# Supplementary figures and images for: Genetic characterization of outbred Sprague Dawley rats and utility for genome-wide association studies
Source: PLoS Genet. 2022 May 31;18(5):e1010234. doi: 10.1371/journal.pgen.1010234 (PMC9187121; doi:10.1371/journal.pgen.1010234)

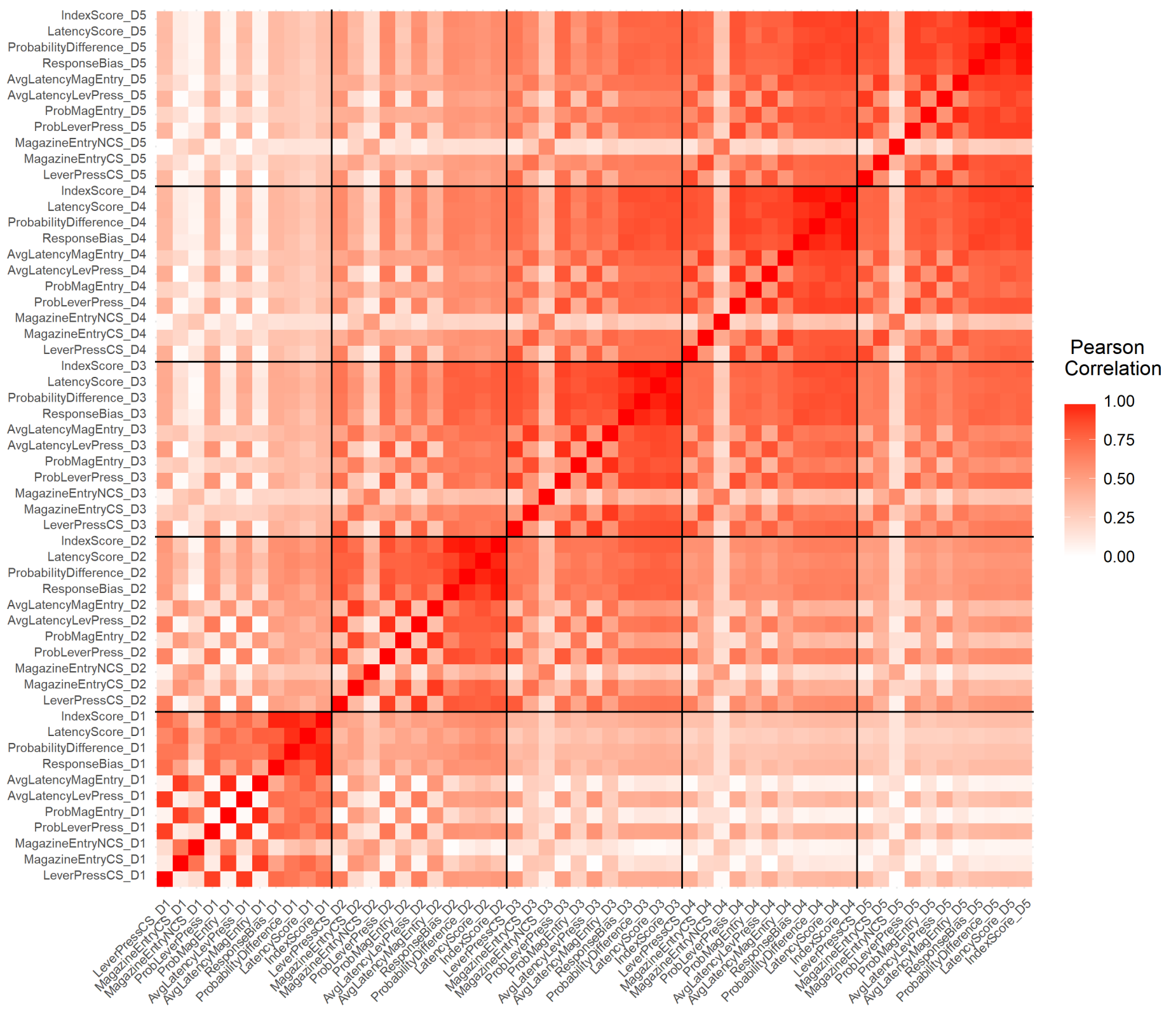

Supplement: S1 Fig — The heatmap displays the absolute value of the Pearson correlation coefficient between each pair of PavCA metrics across all 5 days of testing. (PDF) [file pgen.1010234.s001.pdf]

**S6 Fig. SNP Counts after pruning by pairwise  $r^2$  LD estimates.**

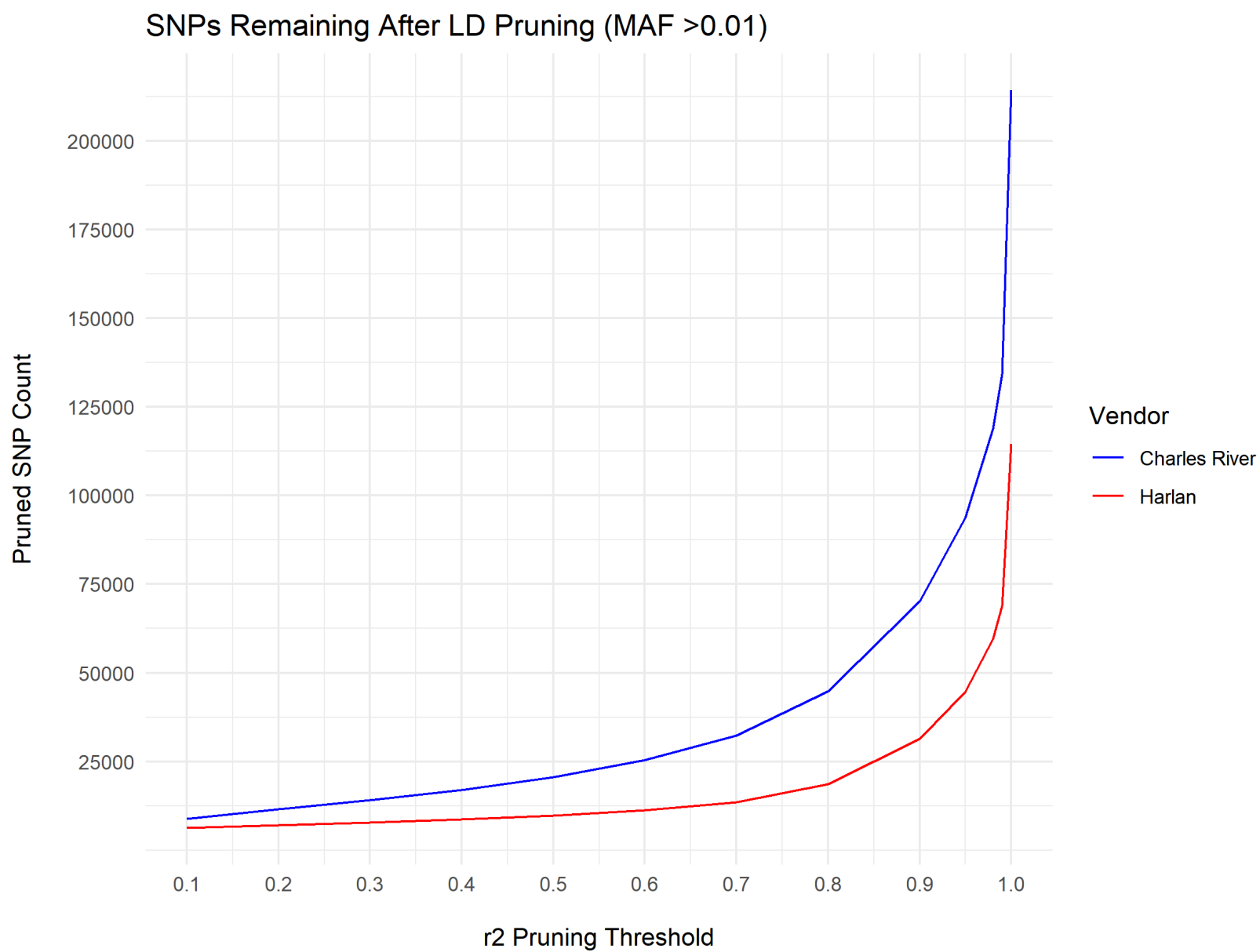

Supplement: S6 Fig — The unfiltered SNP counts represented in this plot are indicated at the x-tick for 1; ~214k for Charles River and 114k for Harlan. Only SNPs with MAF > 0.01 were considered. The stringency of the SNP pruning for the pairwise r2 measurement of LD increases from right to left. For example, 0.95 on the x-axis would indicate that only SNPs with a pairwise correlation of 0.95 or greater has one of the pair removed from the SNP set. (PDF) [file pgen.1010234.s006.pdf]

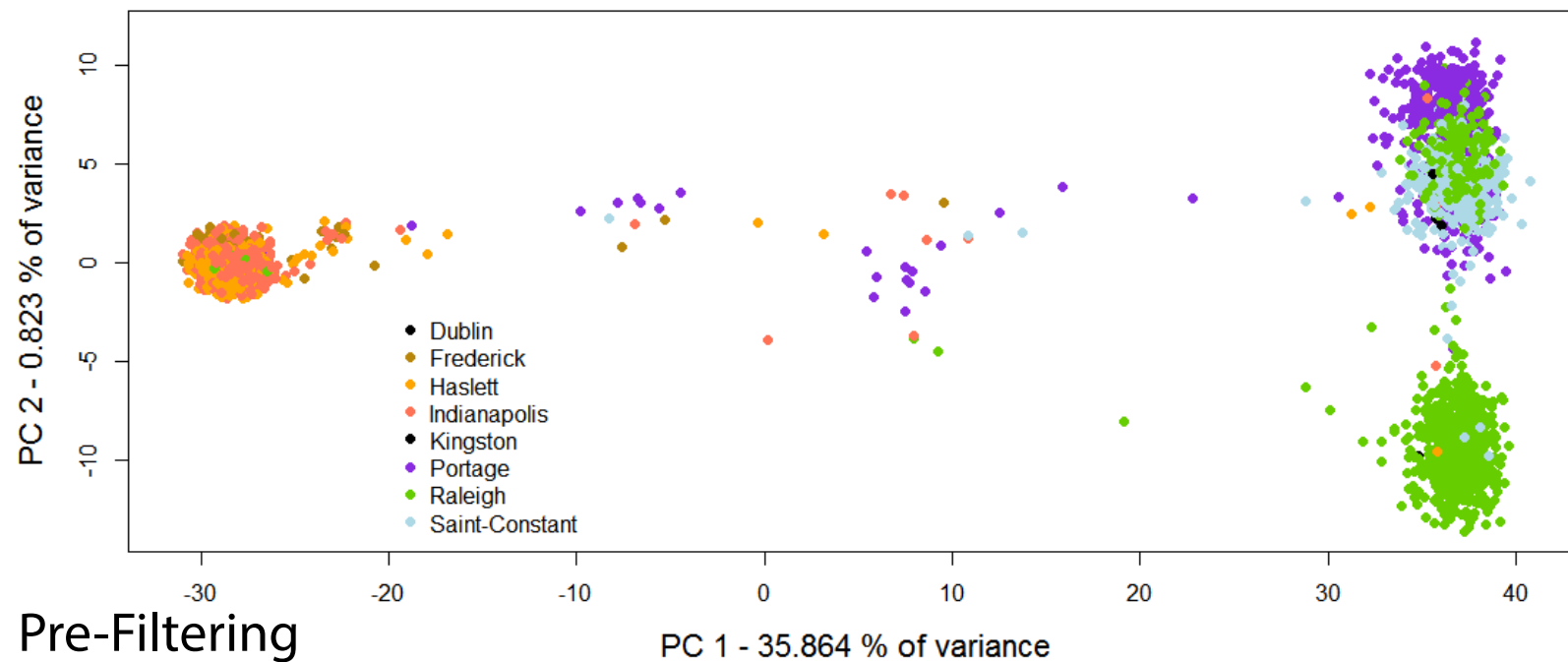

## Post-Filtering

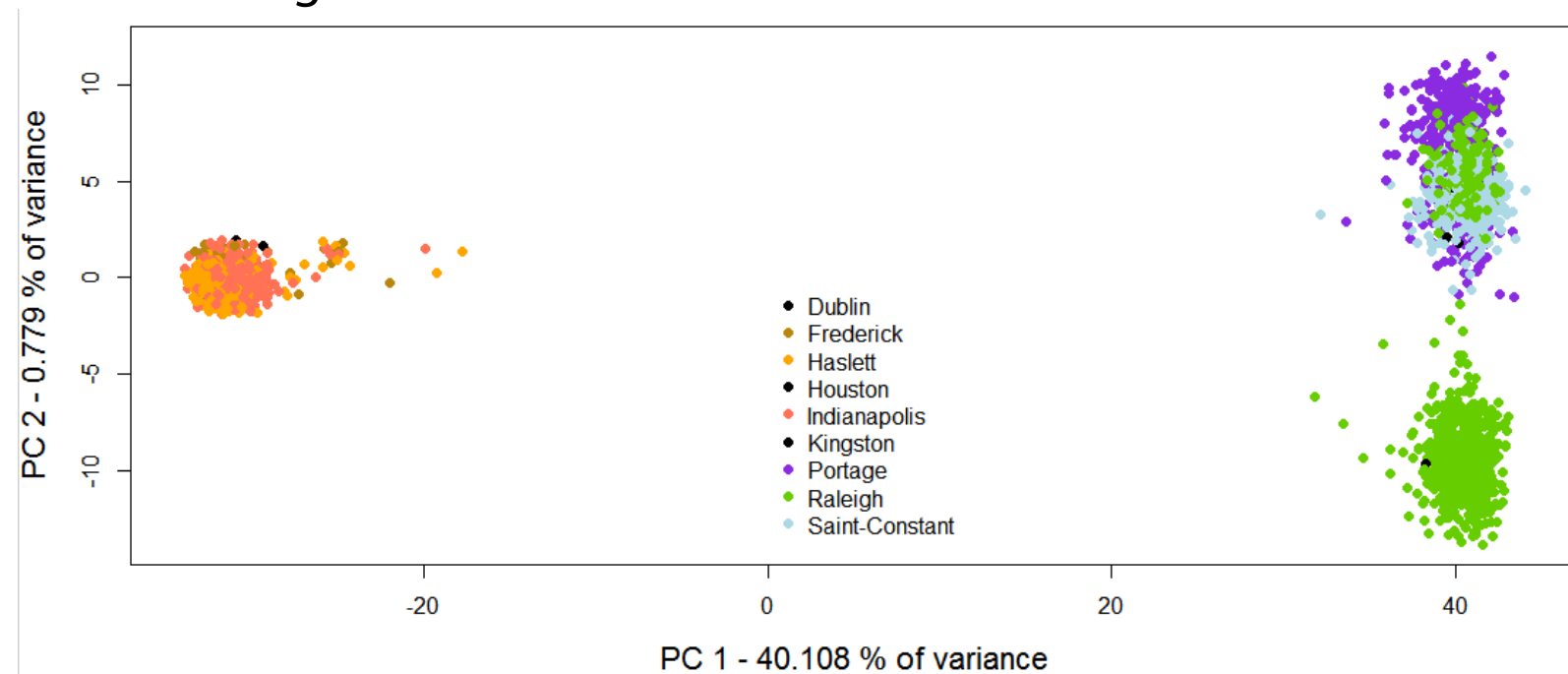

Supplement: S7 Fig — The top plot shows PCA results prior to removing individual outliers that fell far outside their expected cluster based on available metadata. The bottom plot shows the results after visual filtering. (PDF) [file pgen.1010234.s007.pdf]

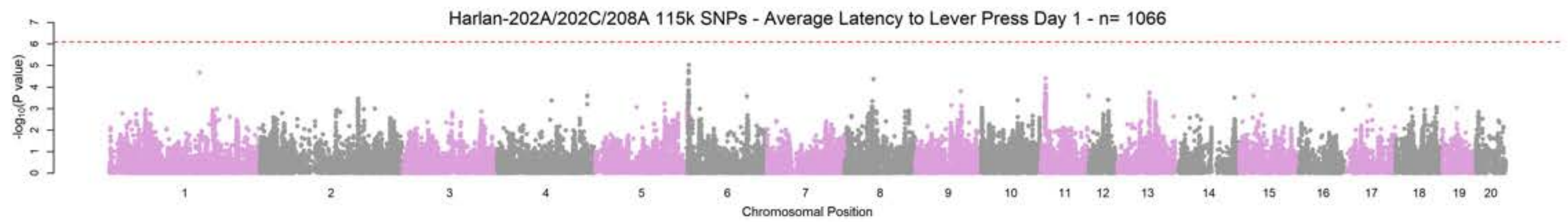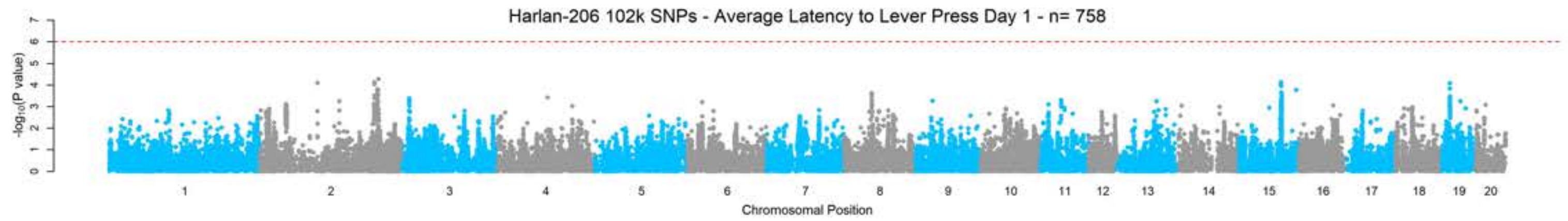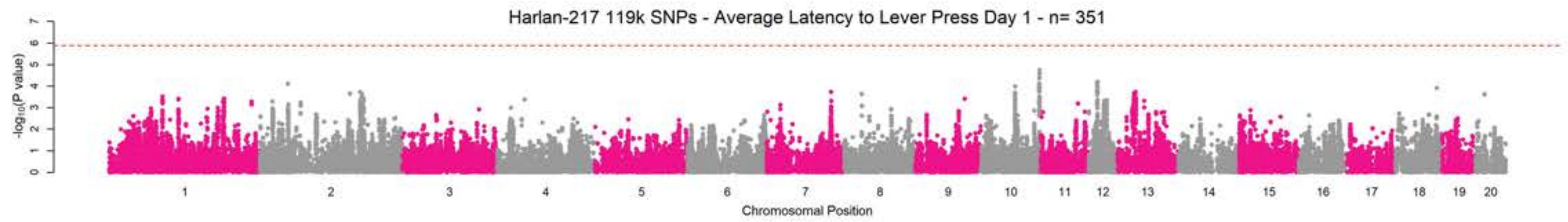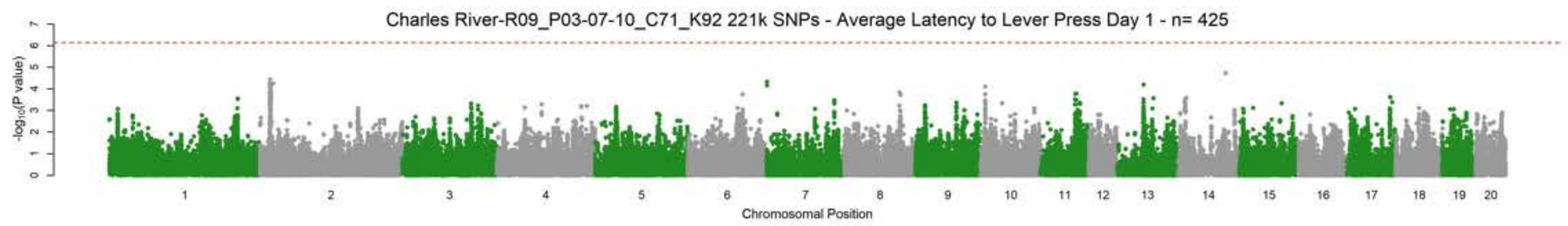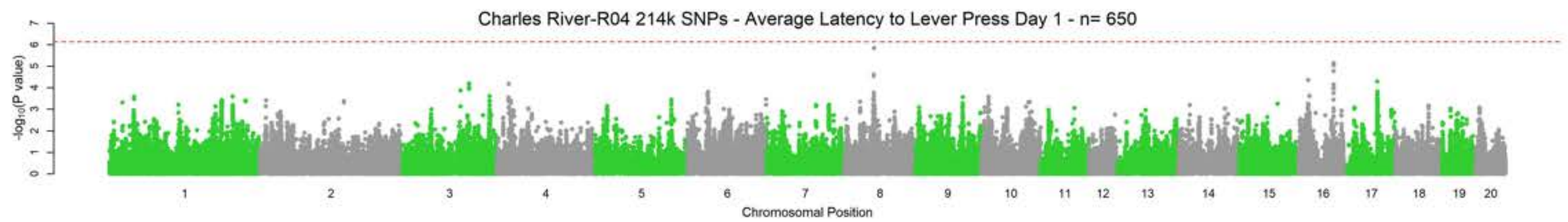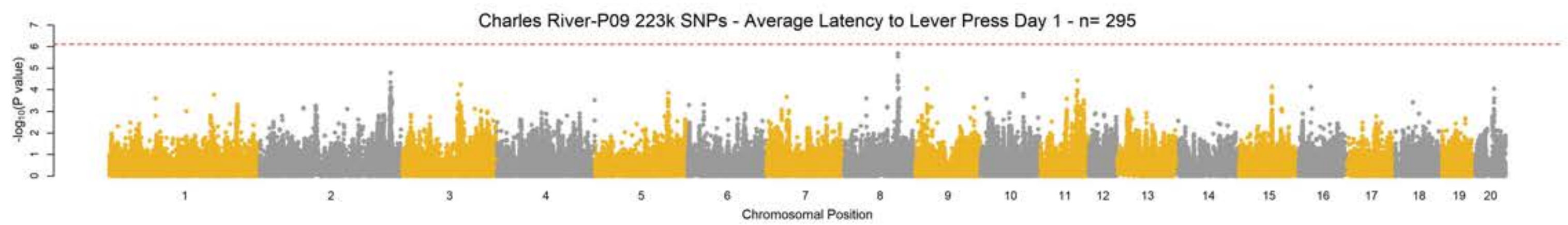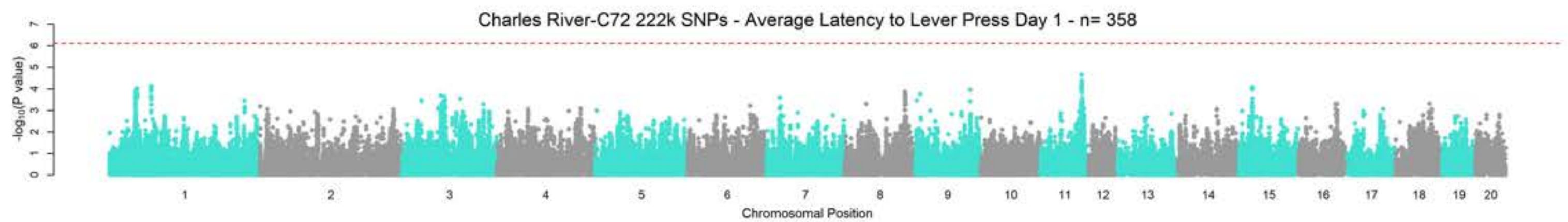

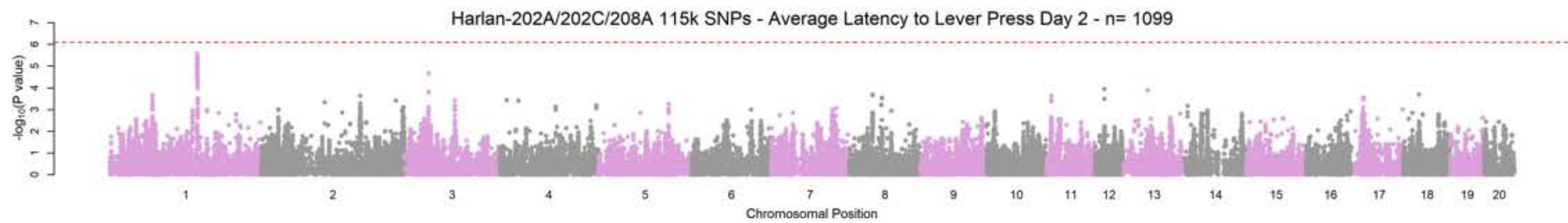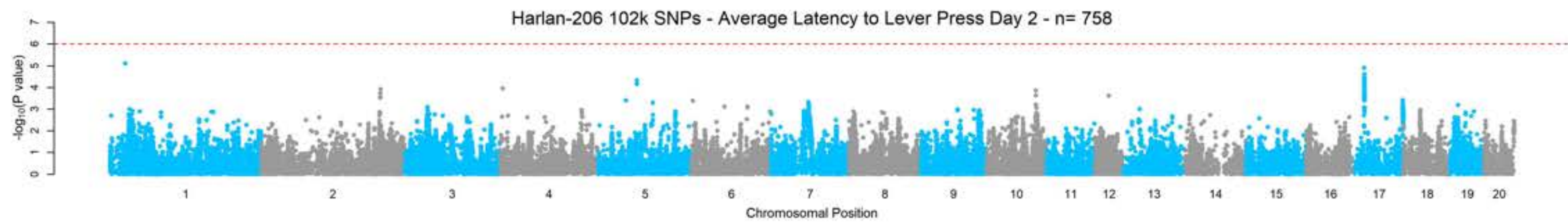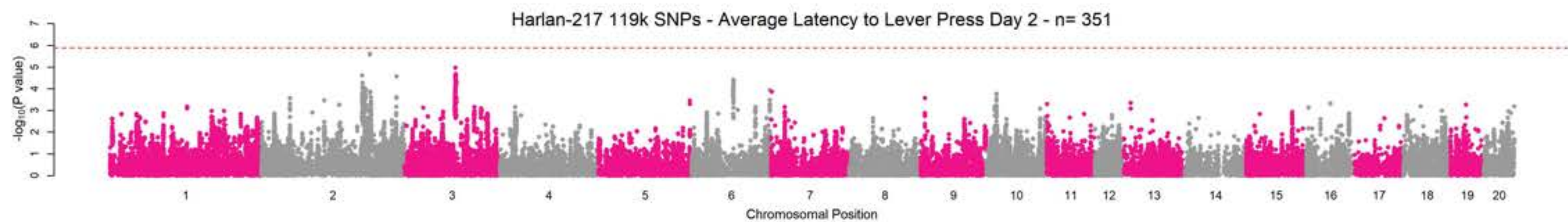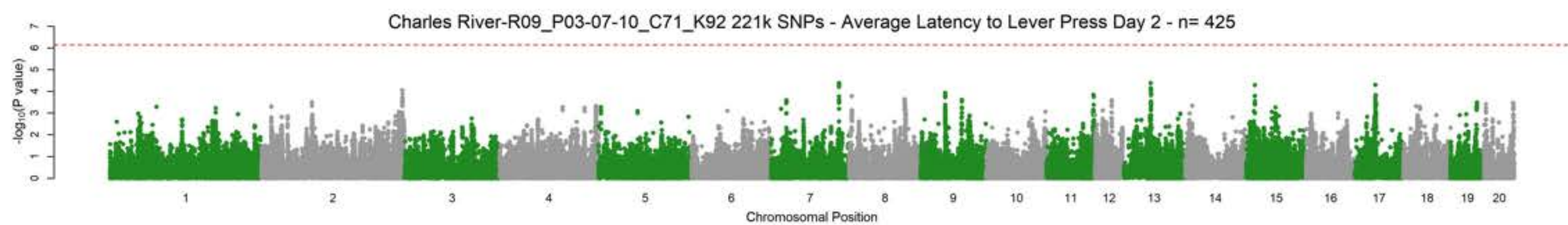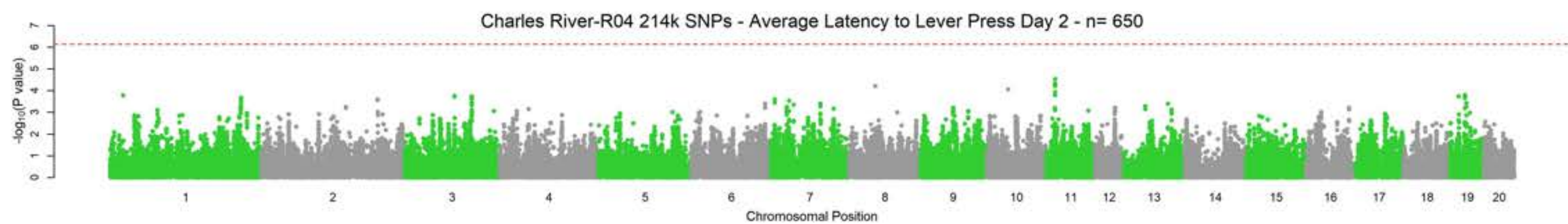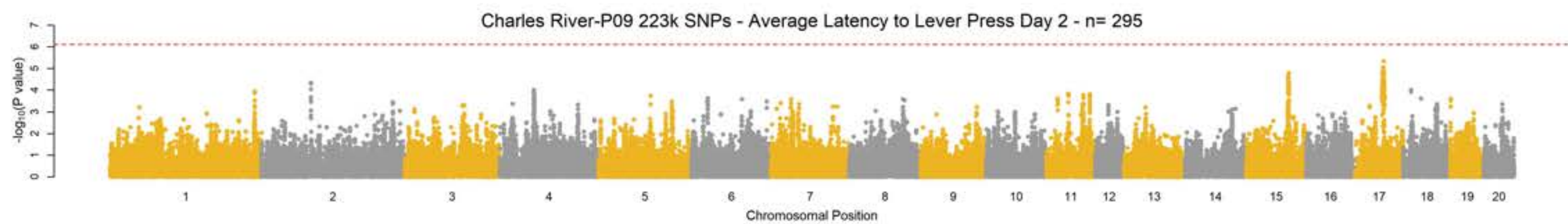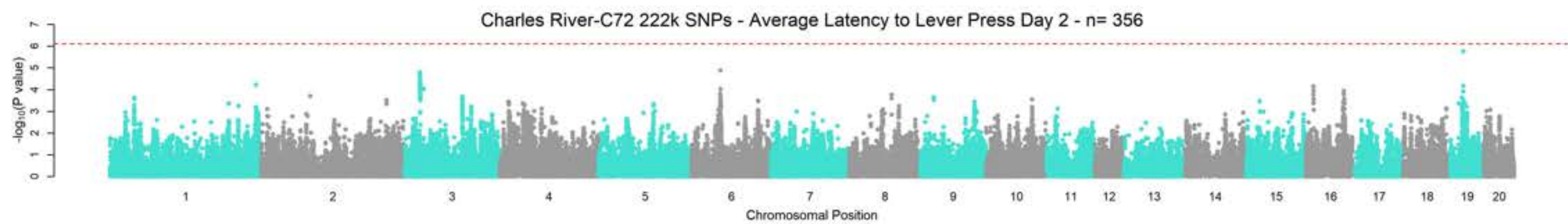

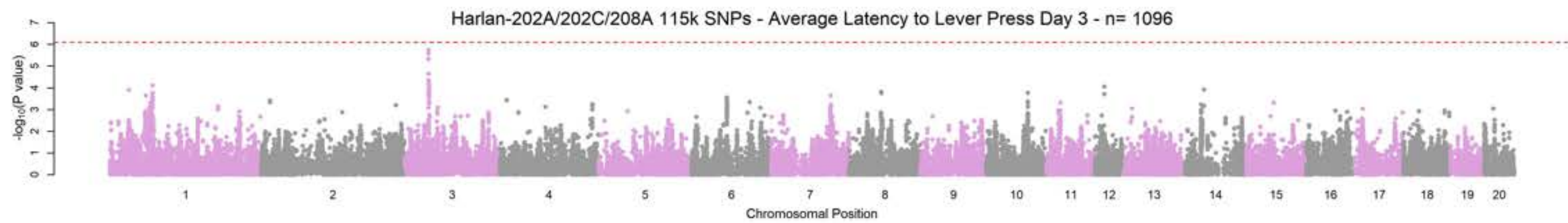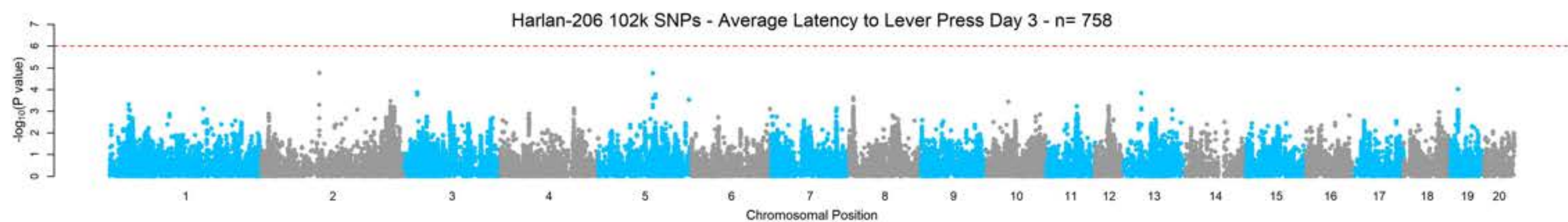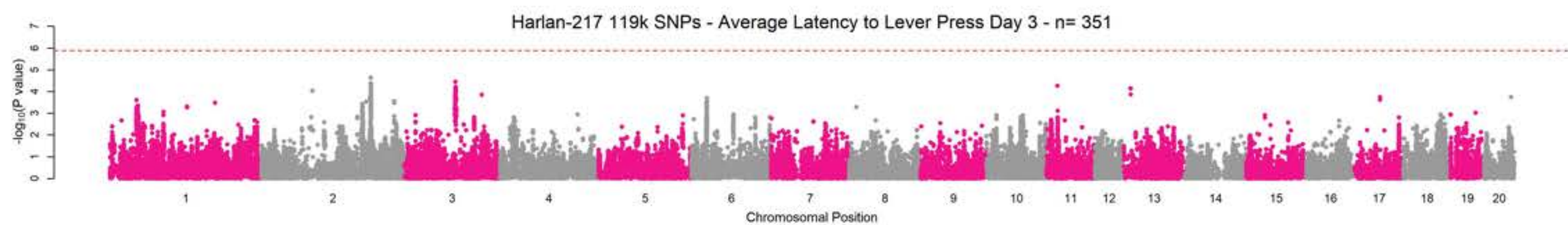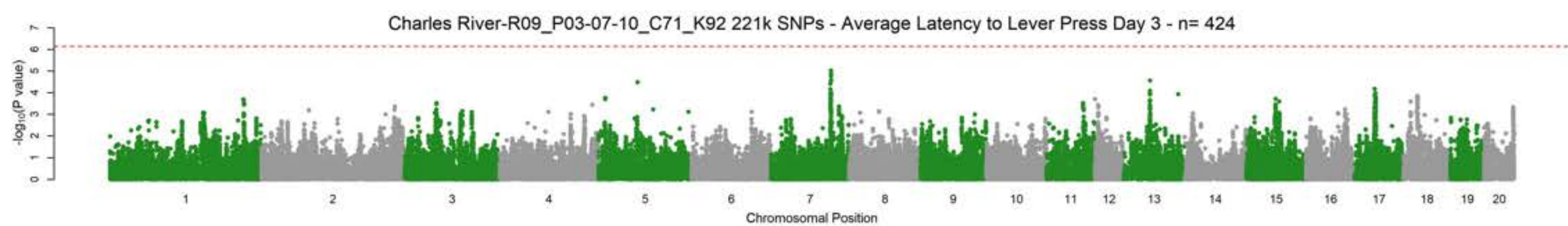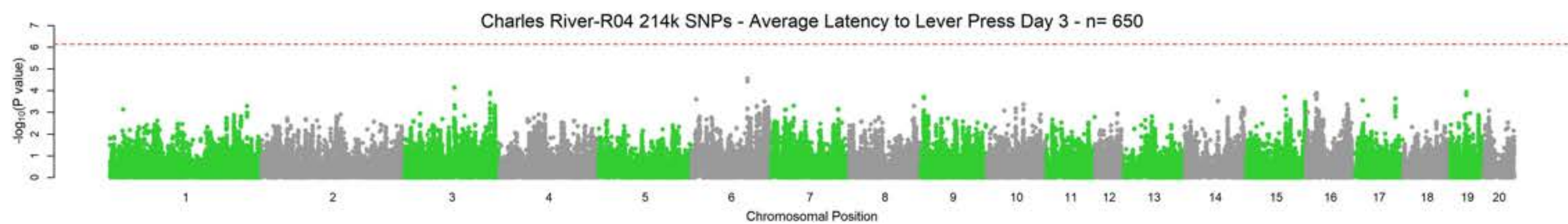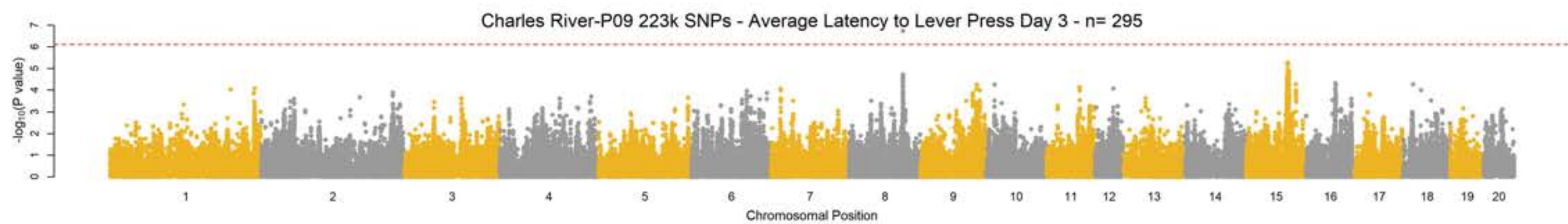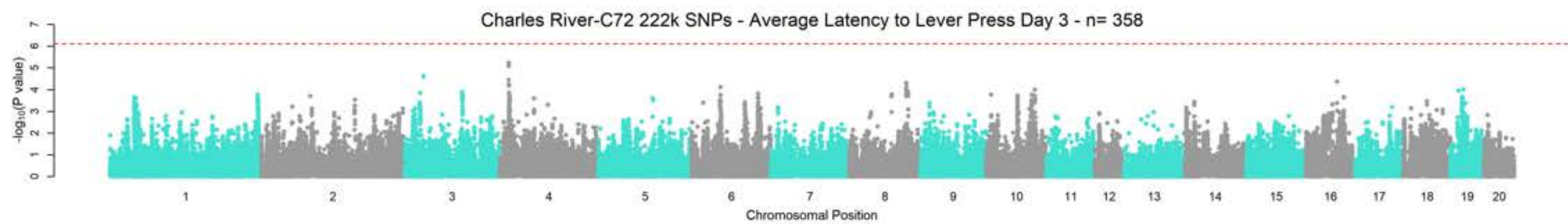

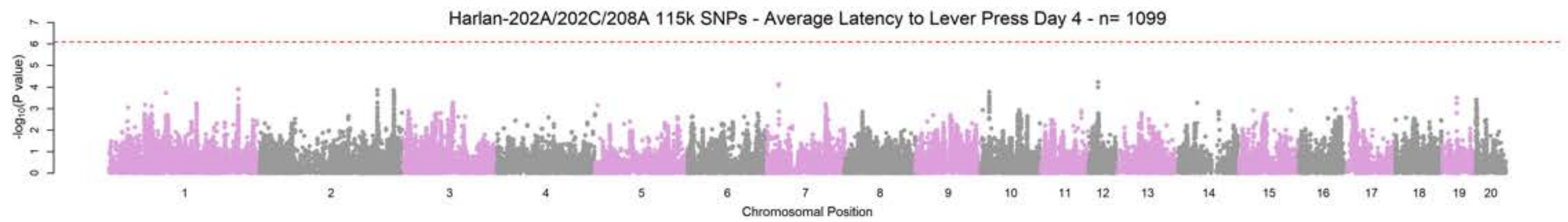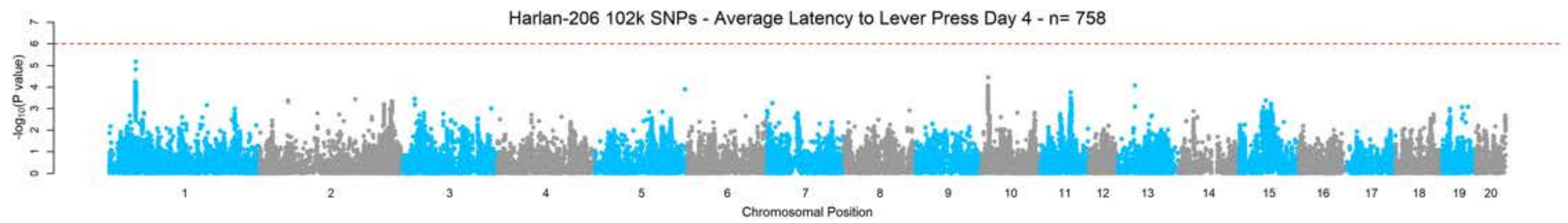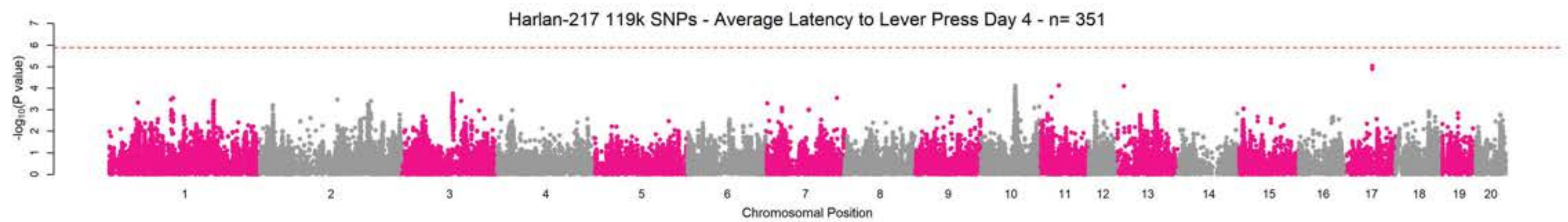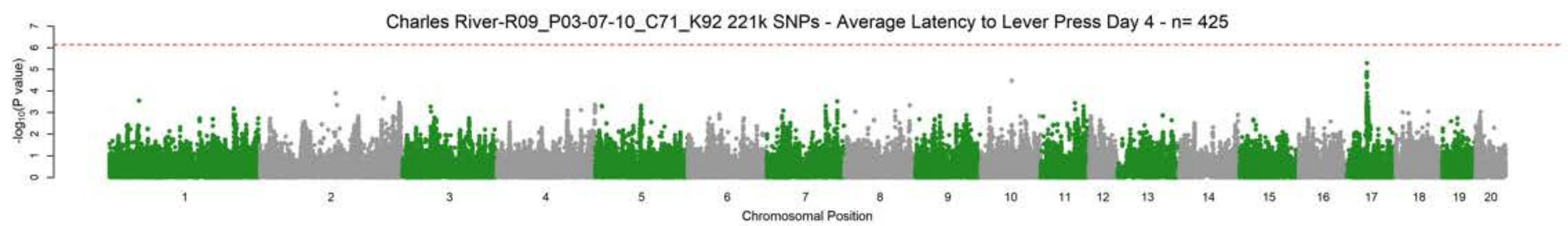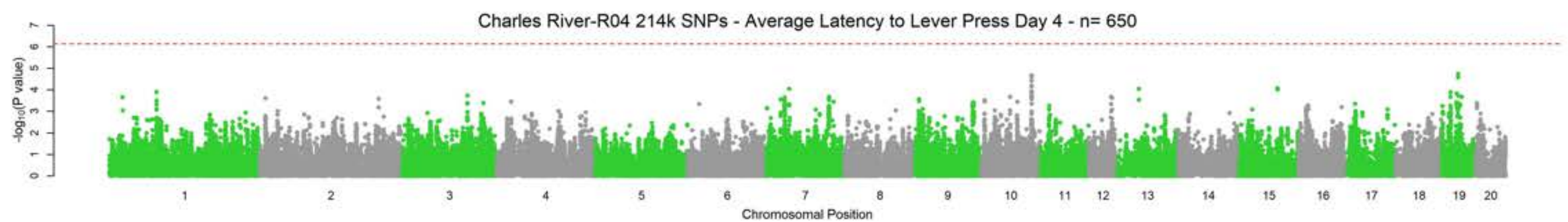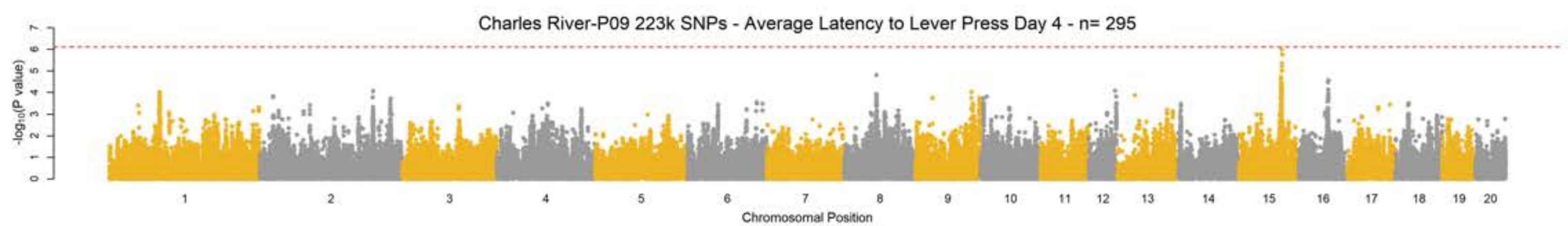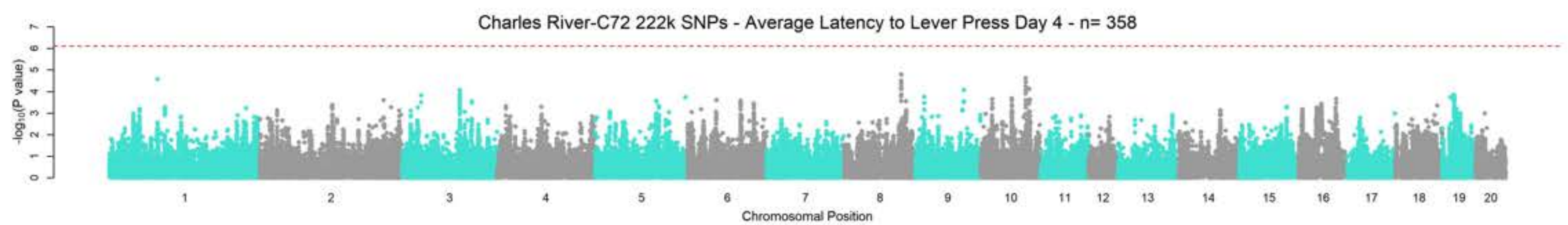

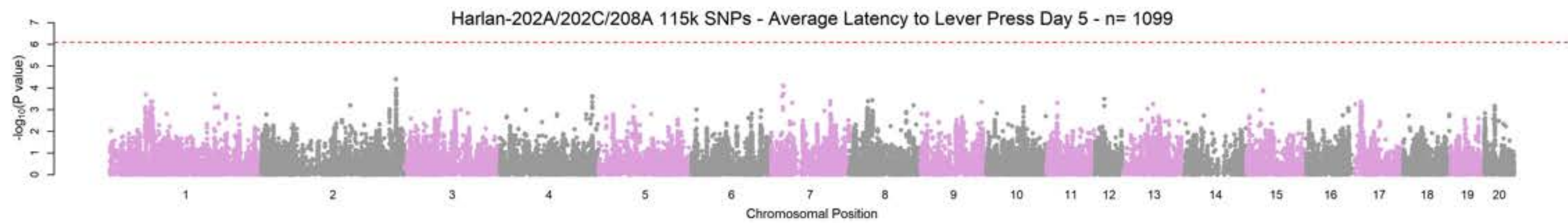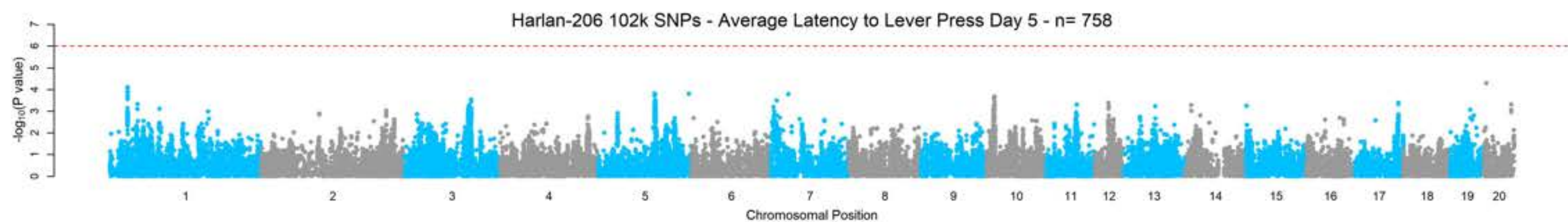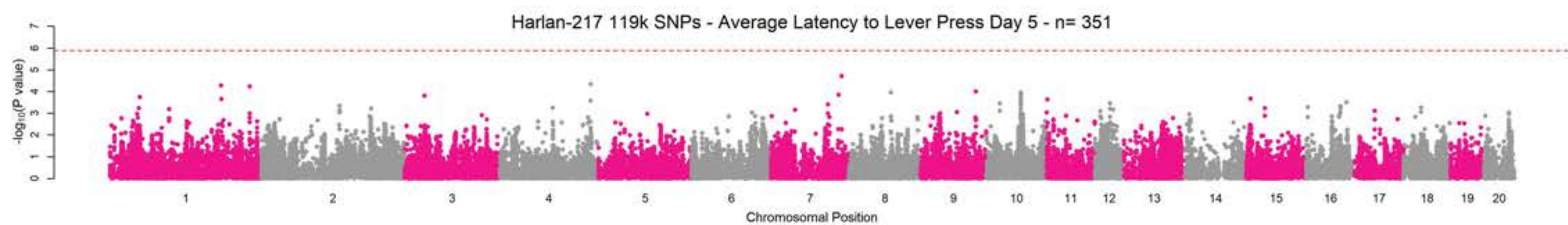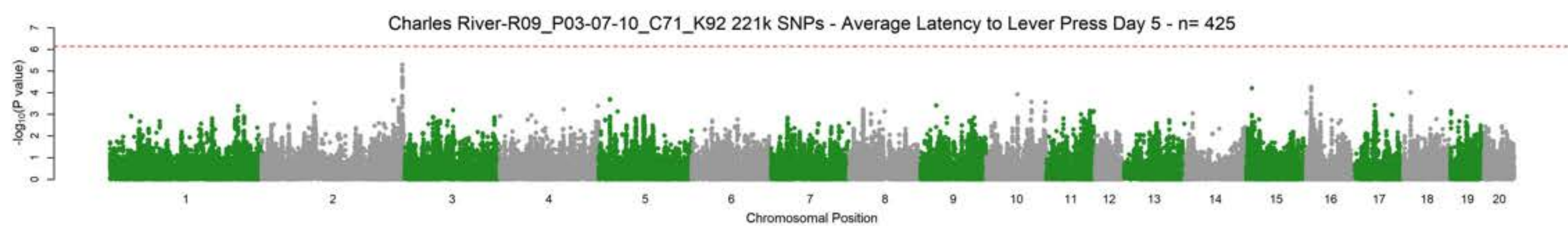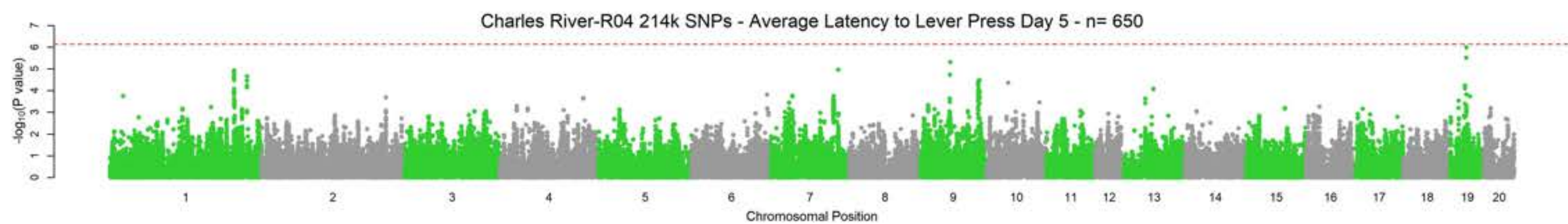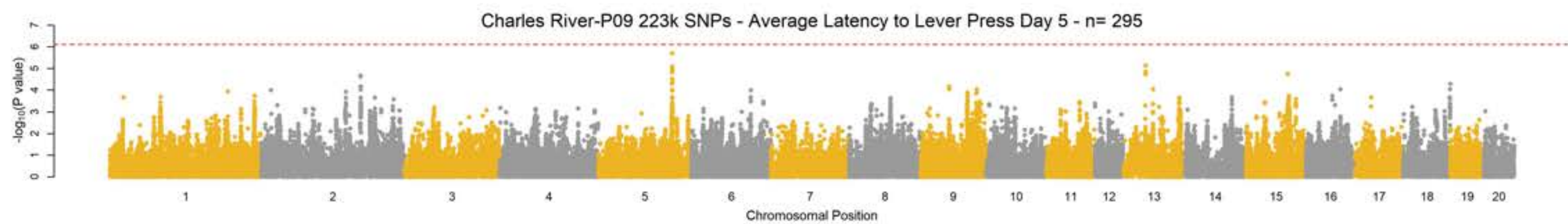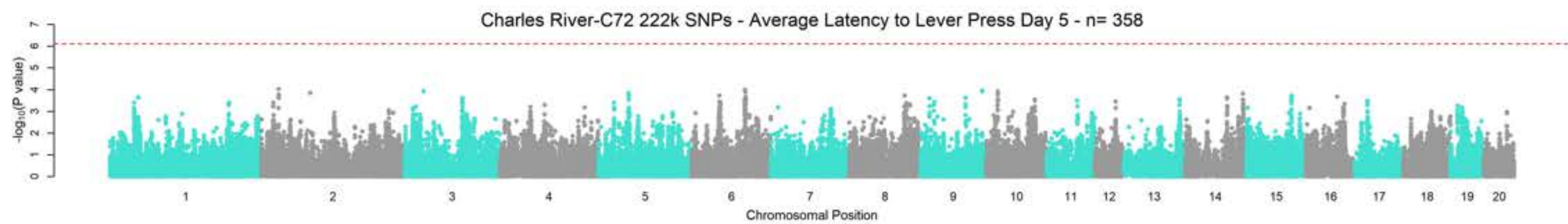

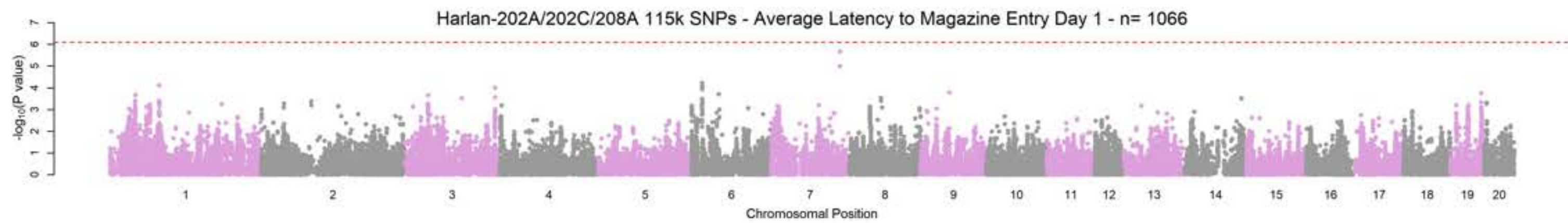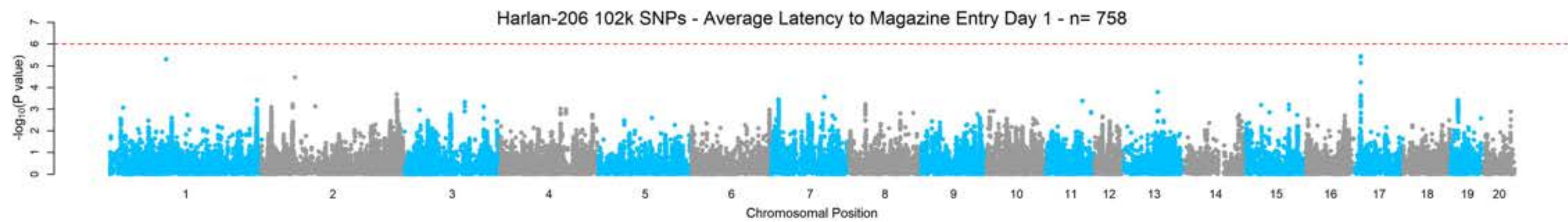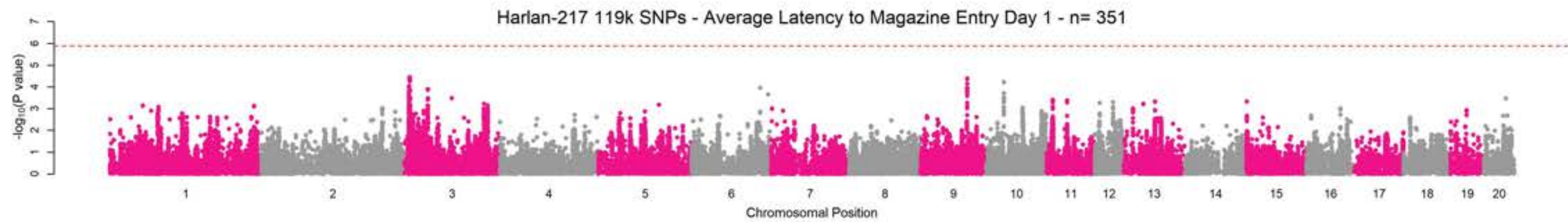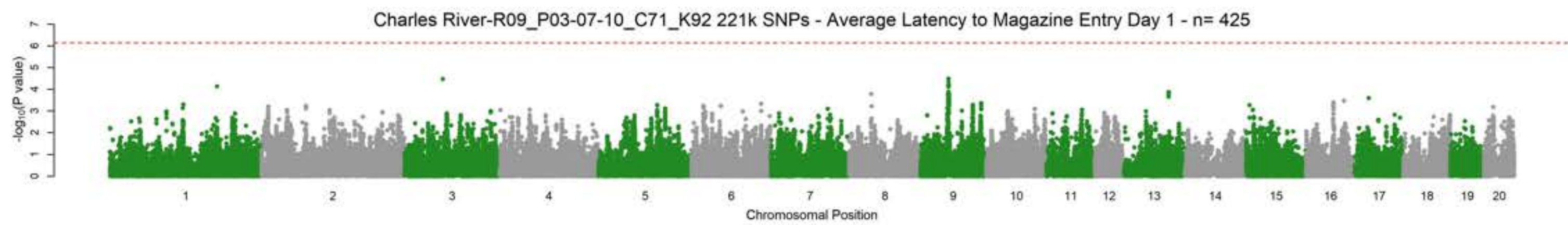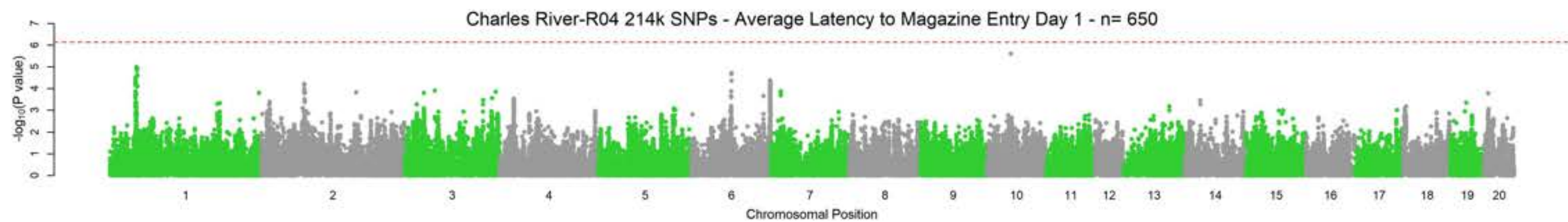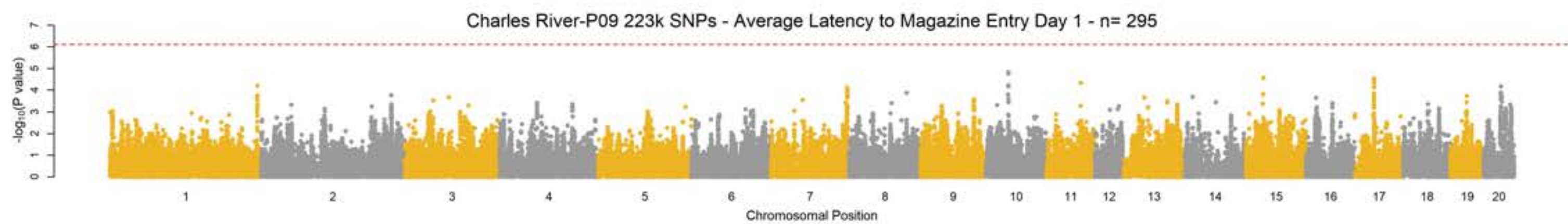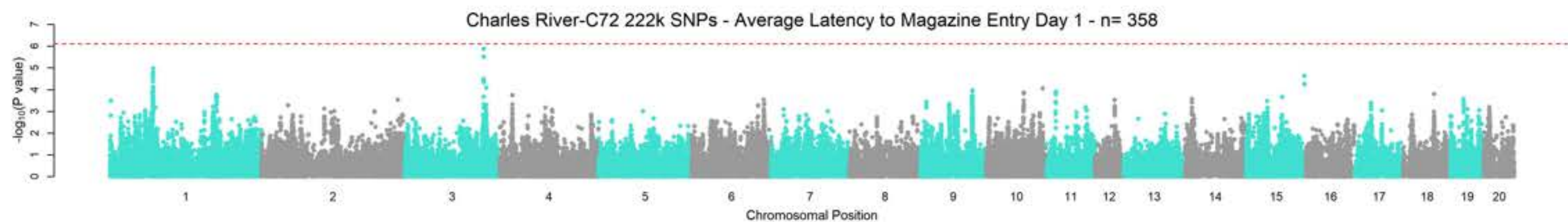

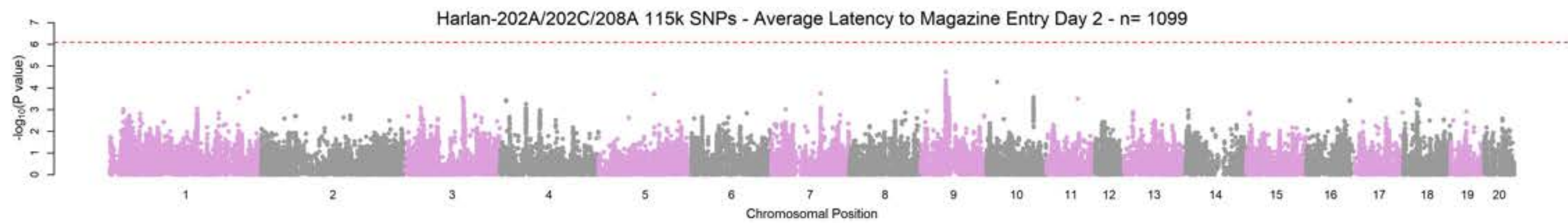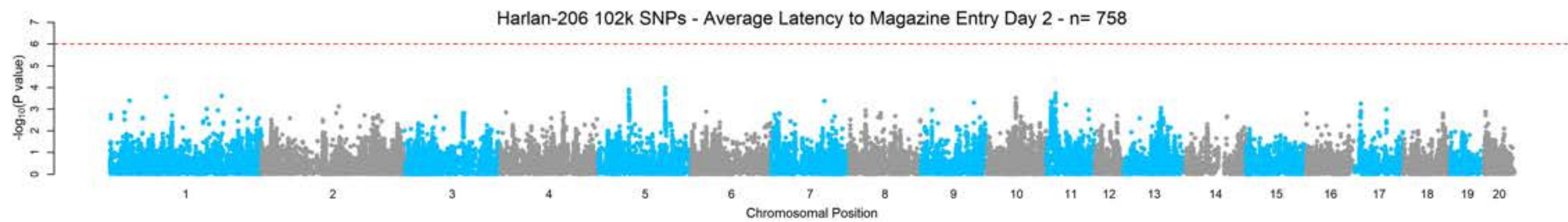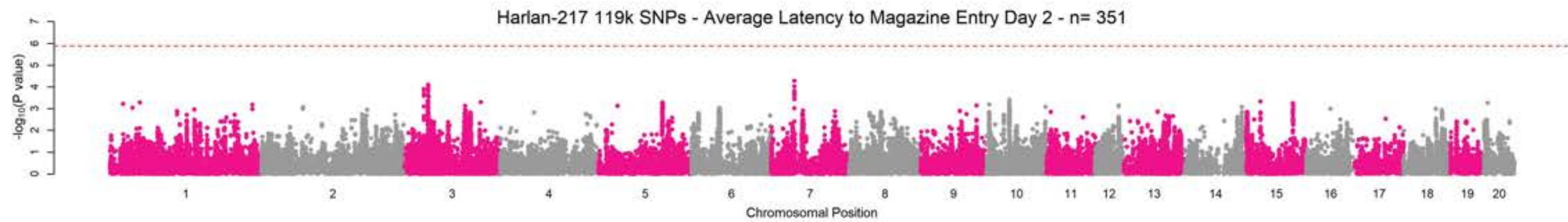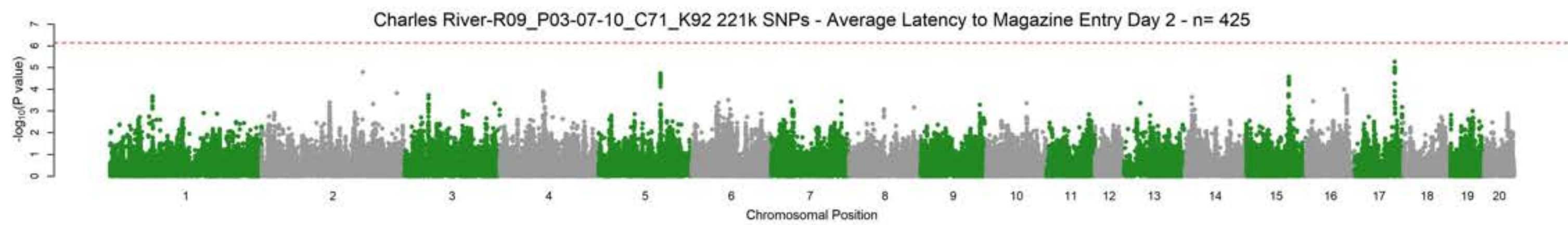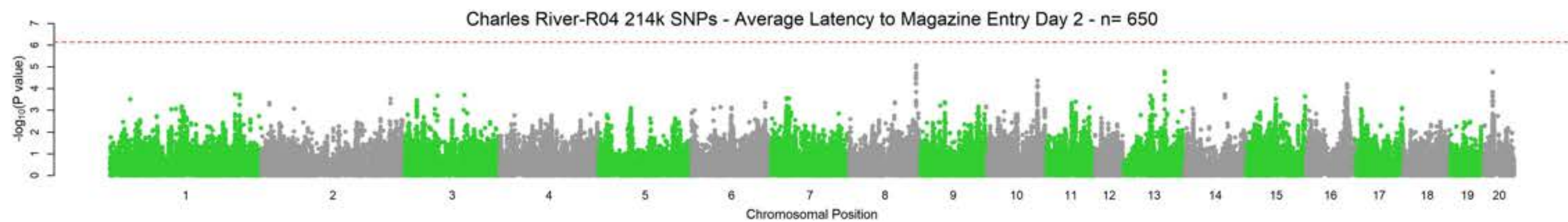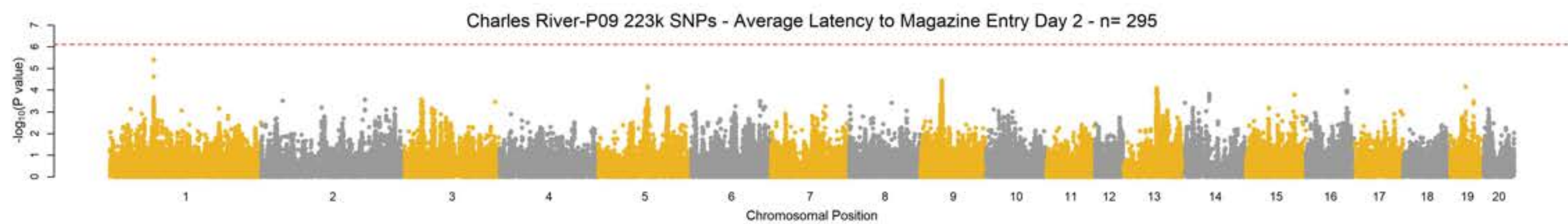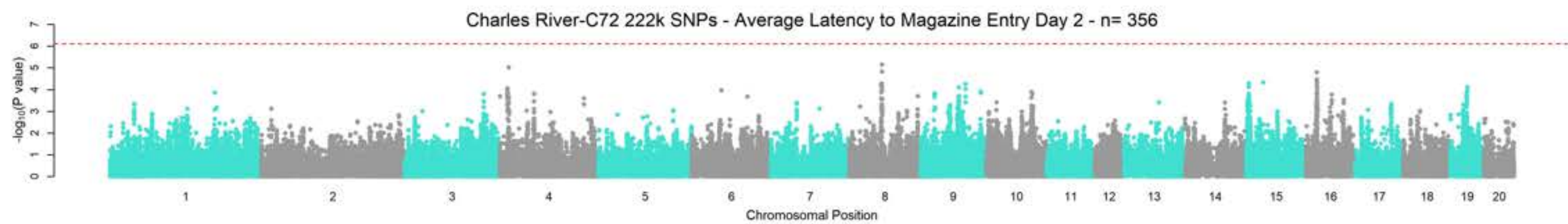

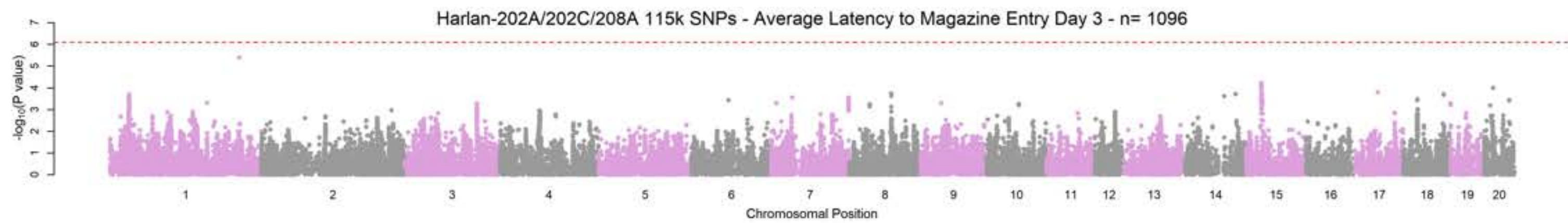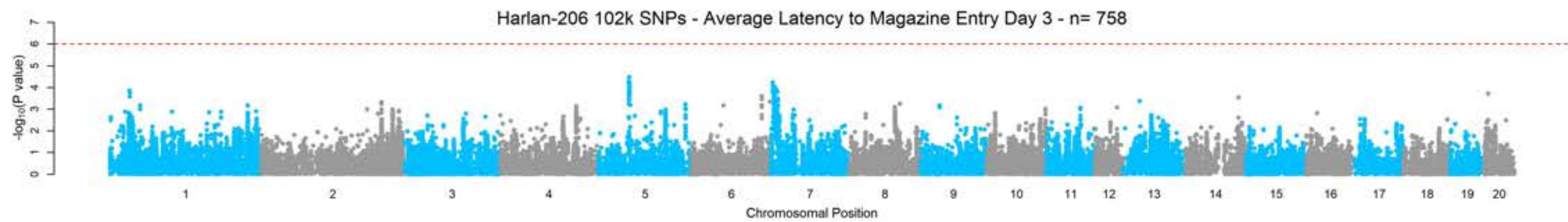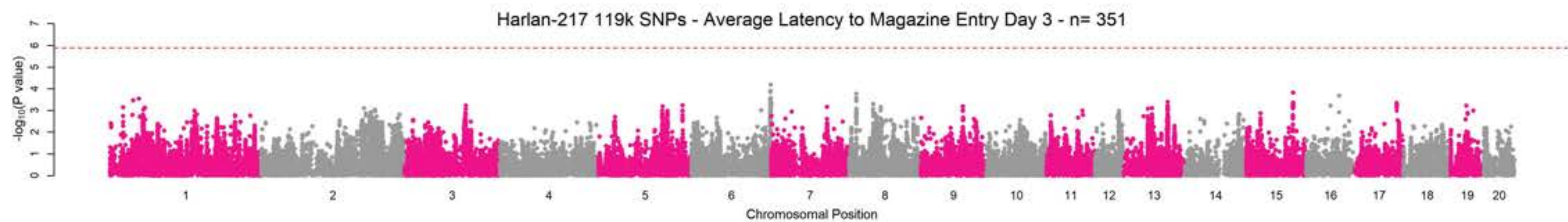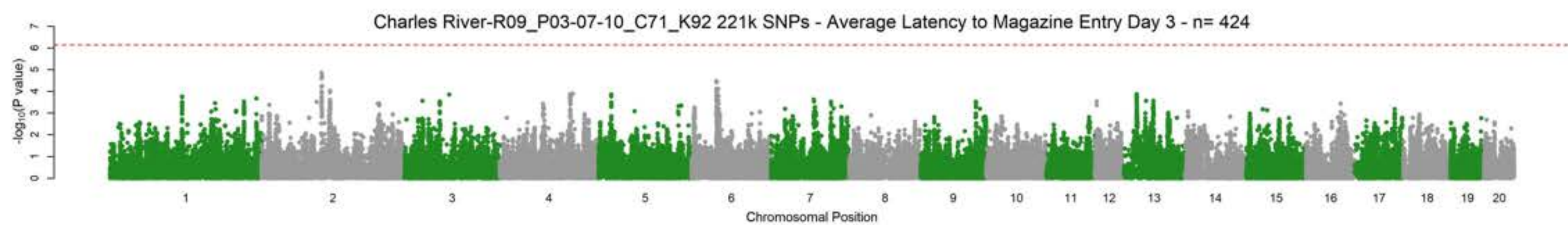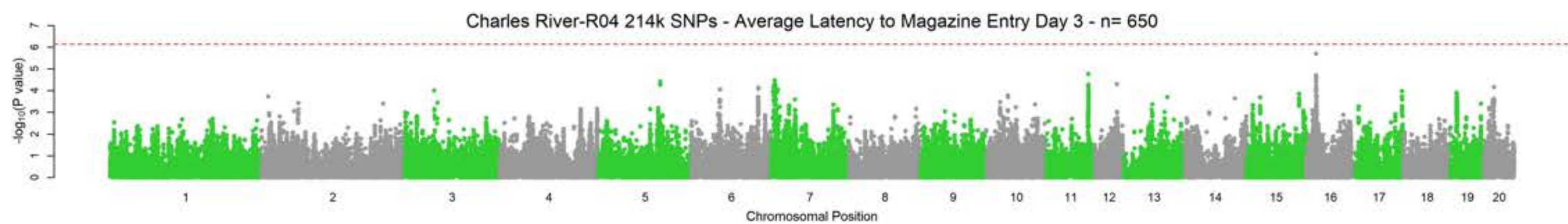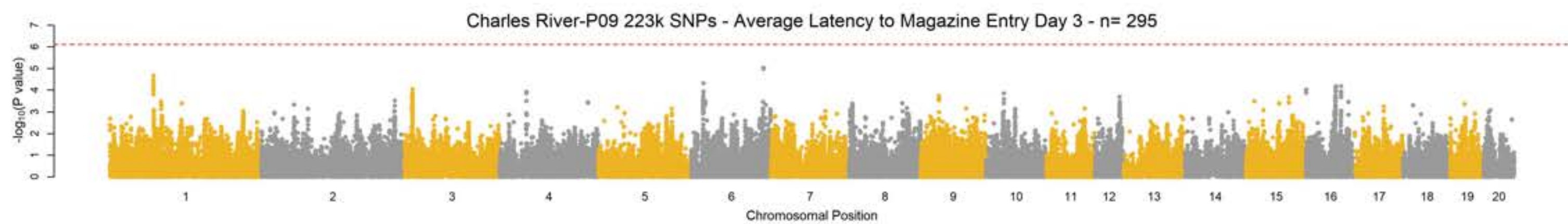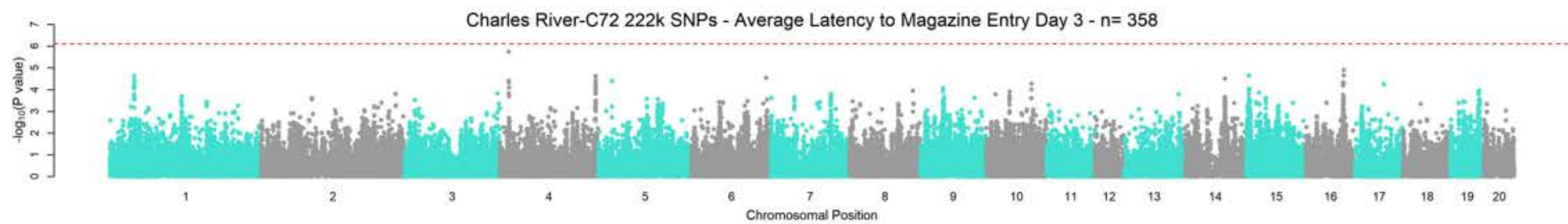

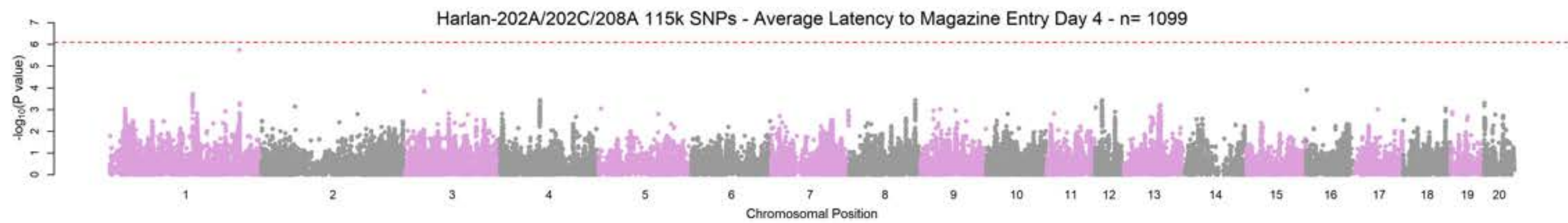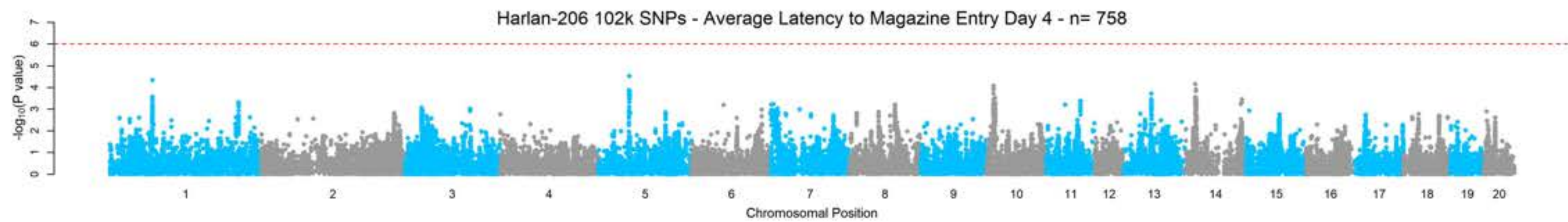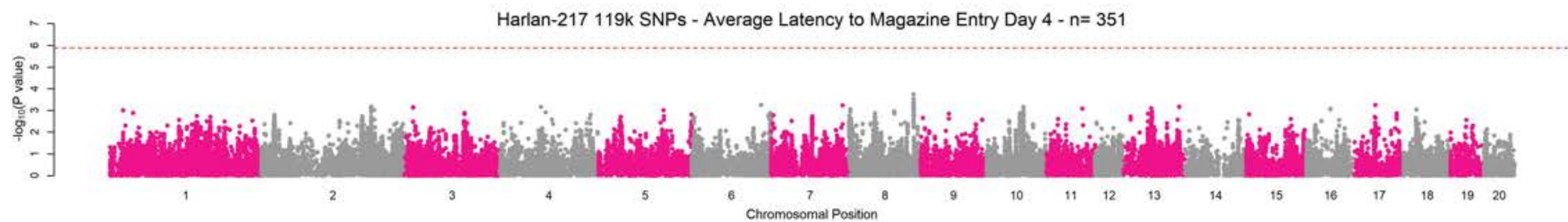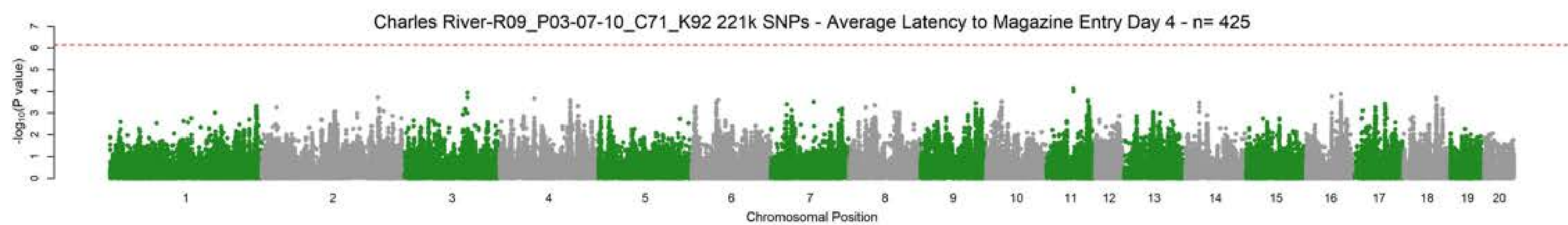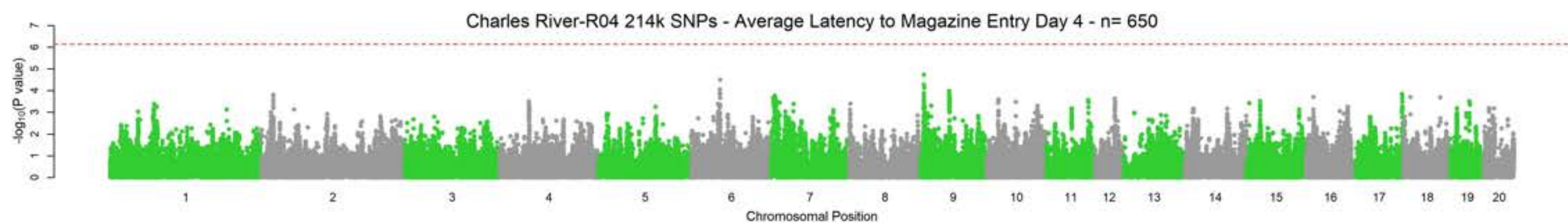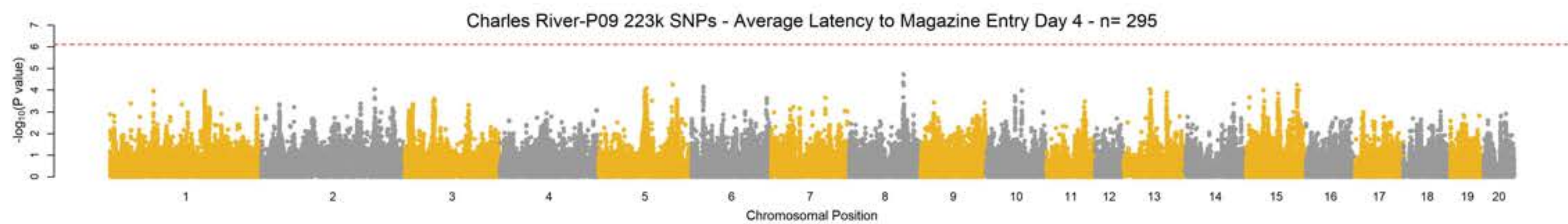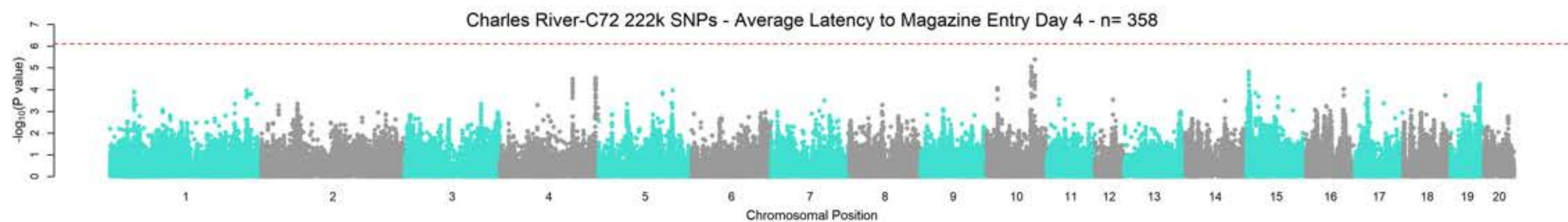

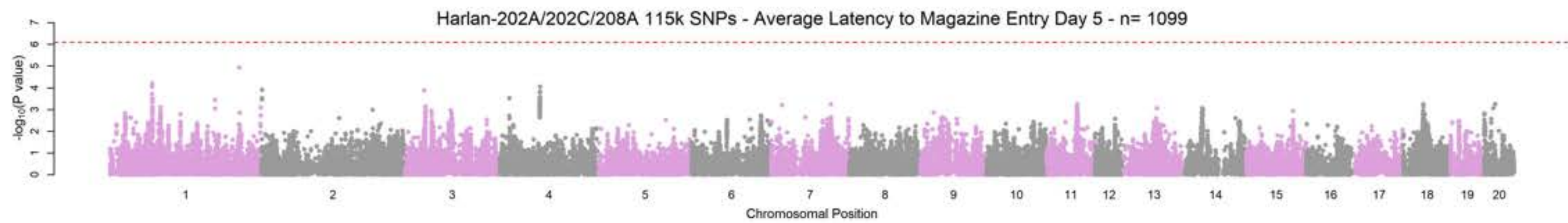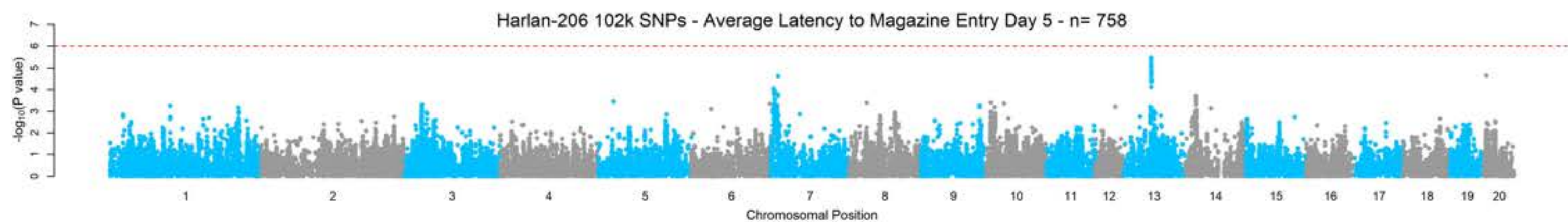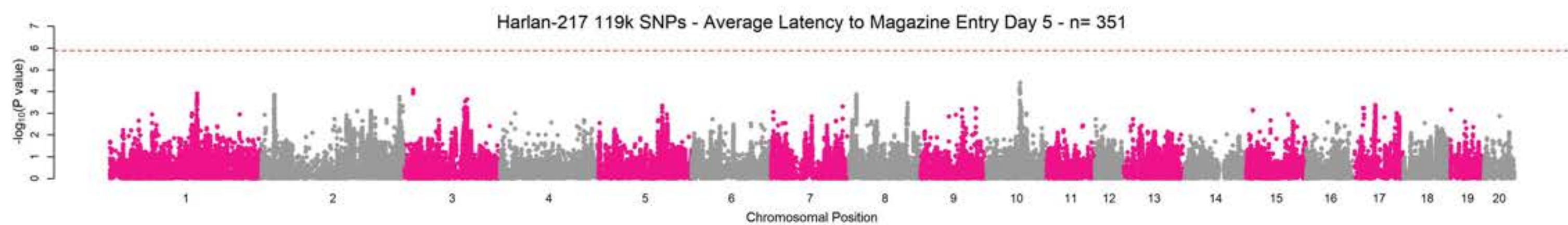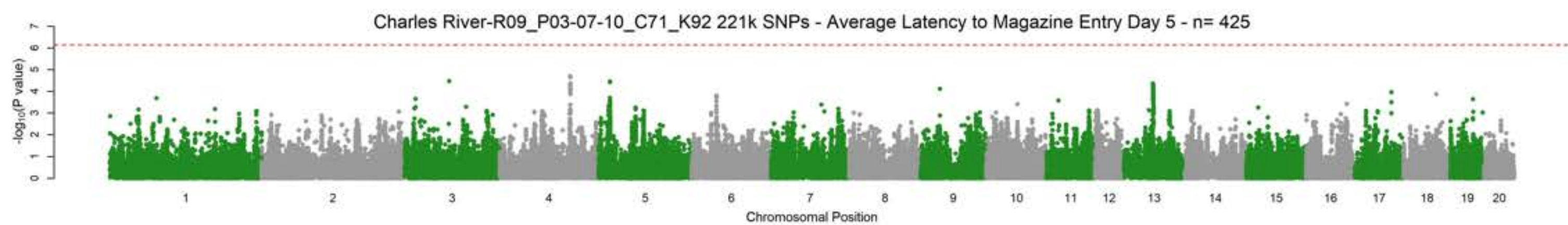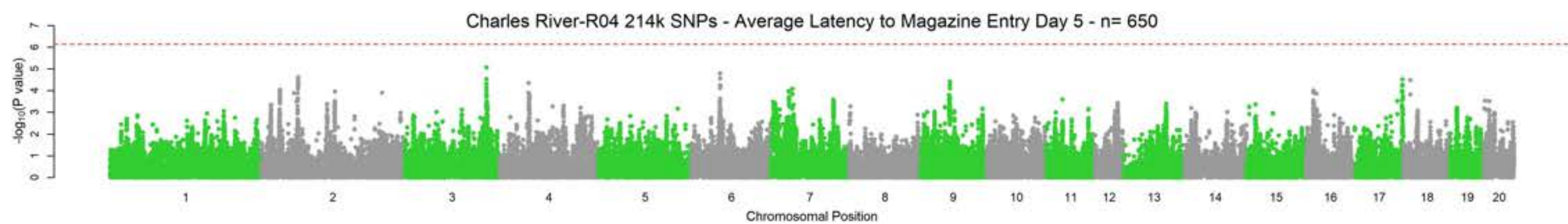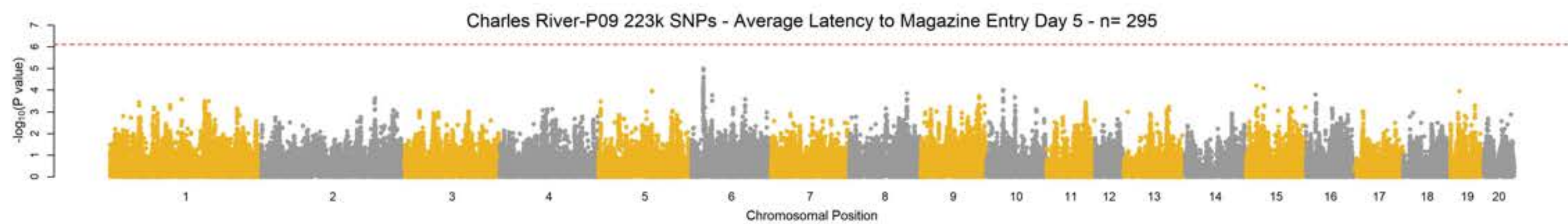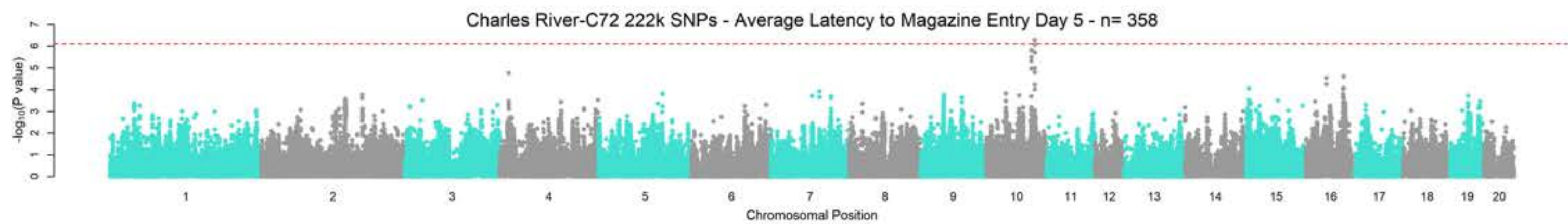

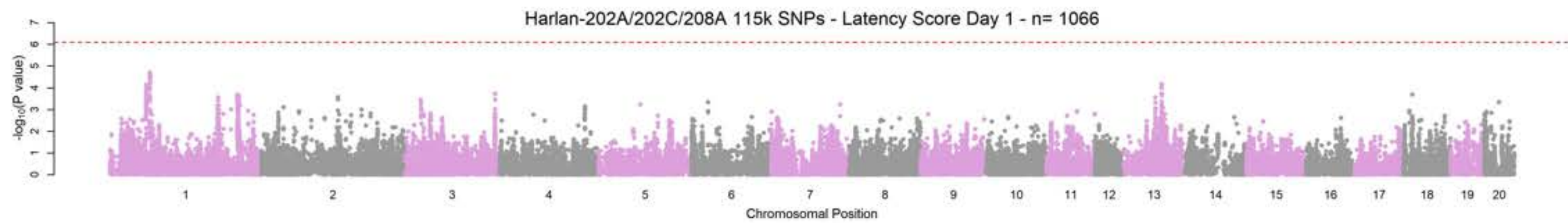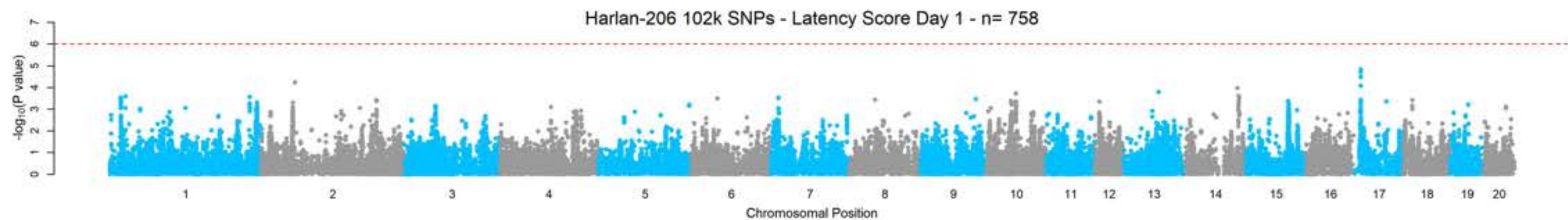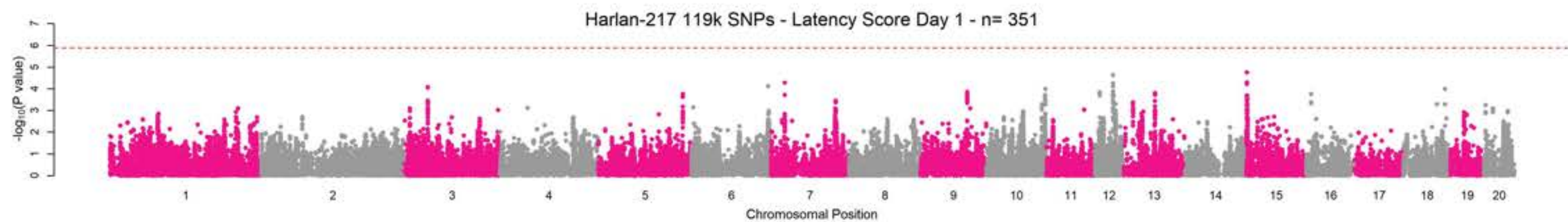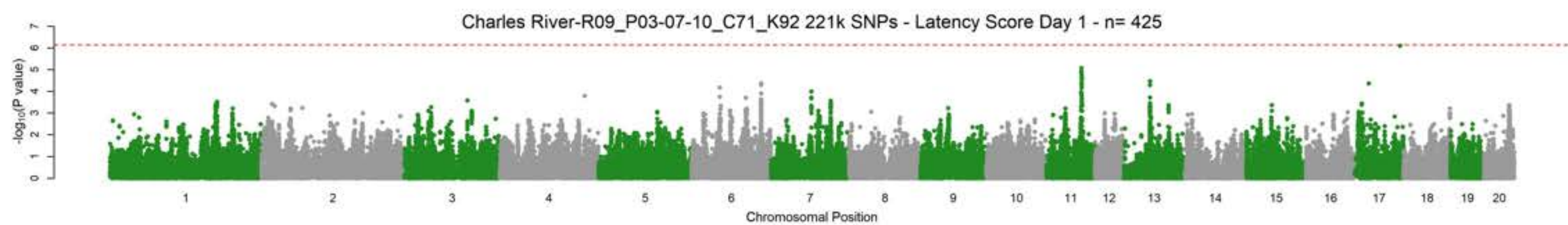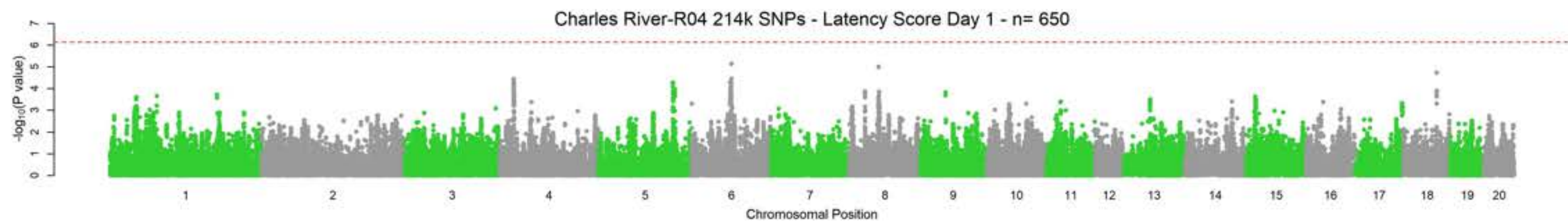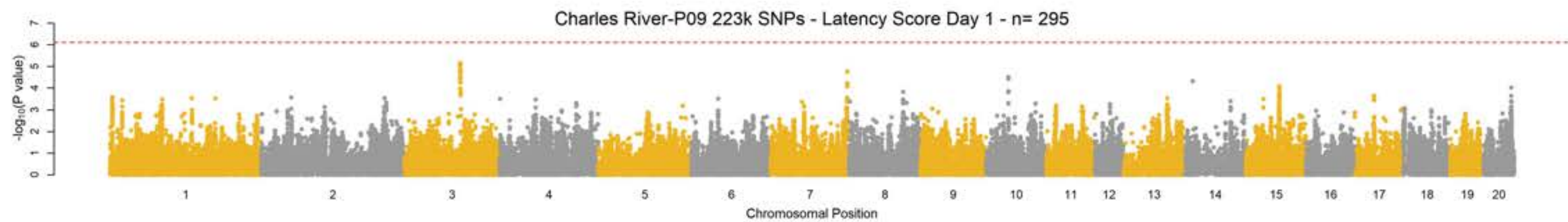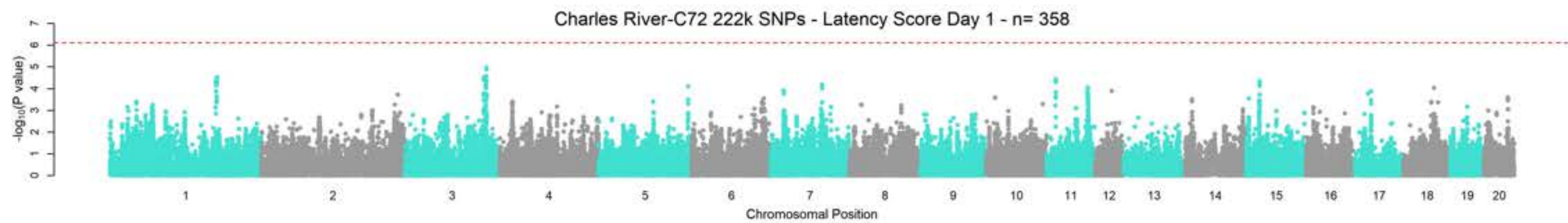

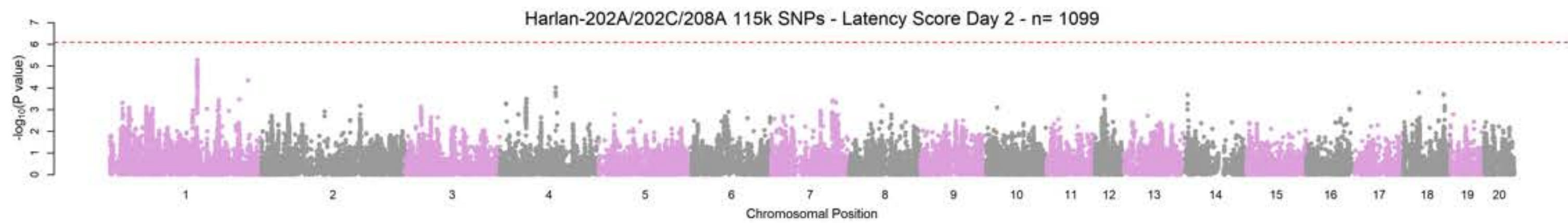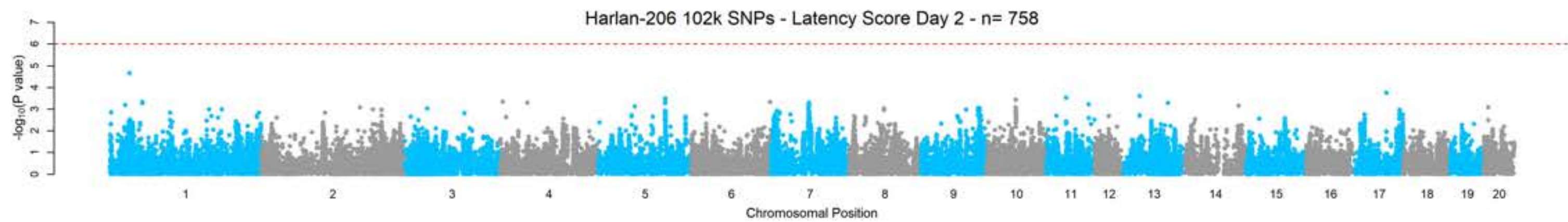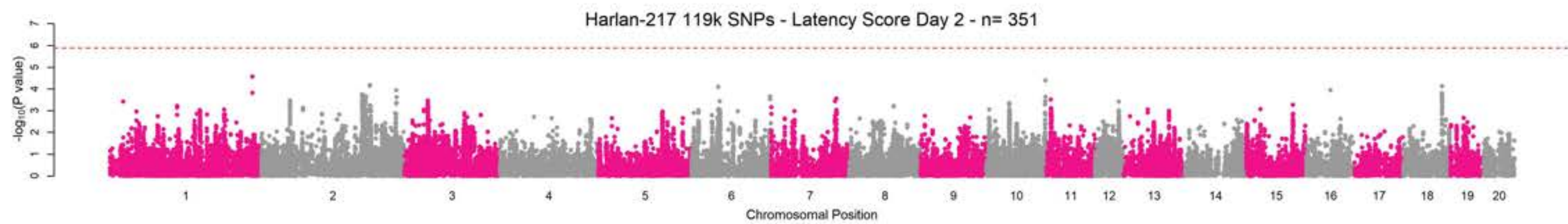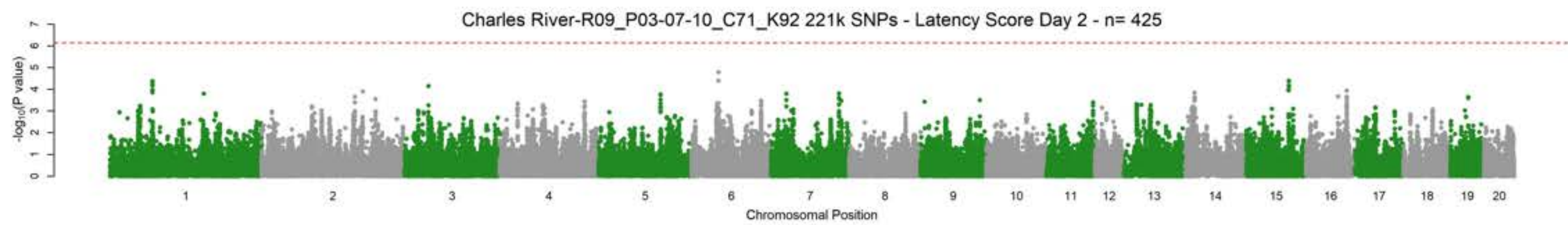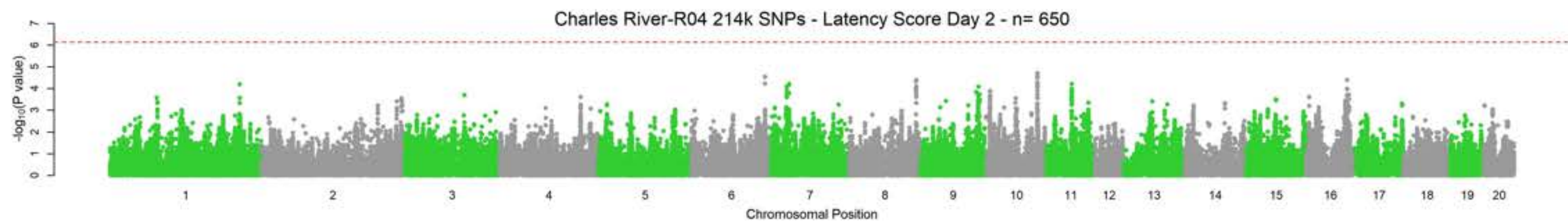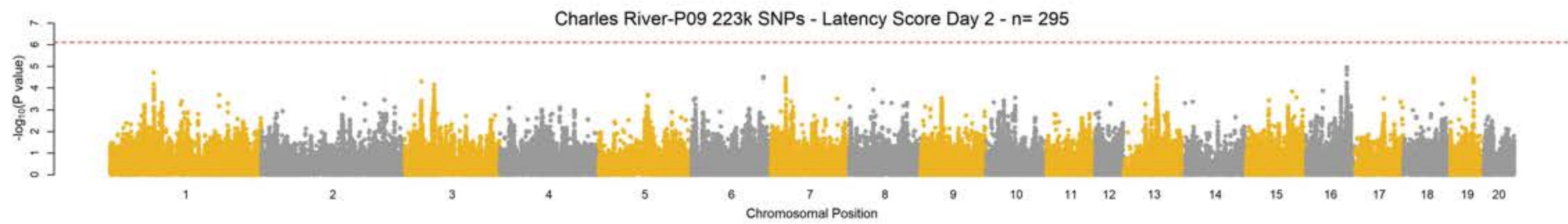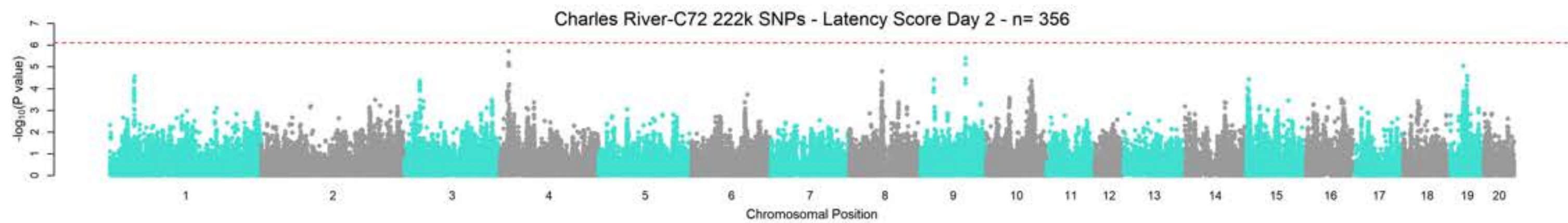

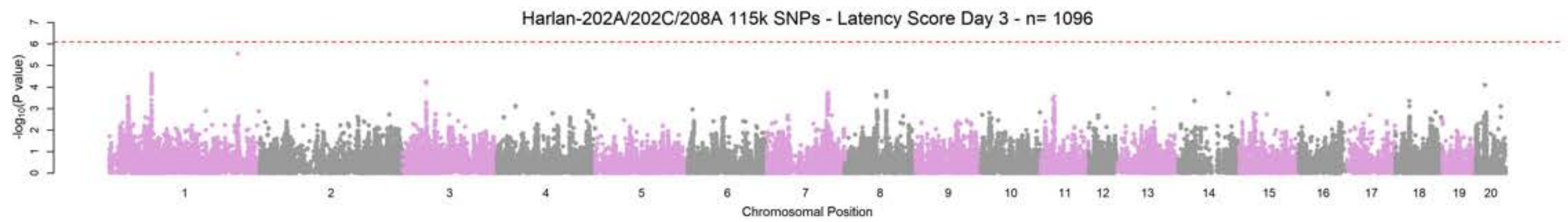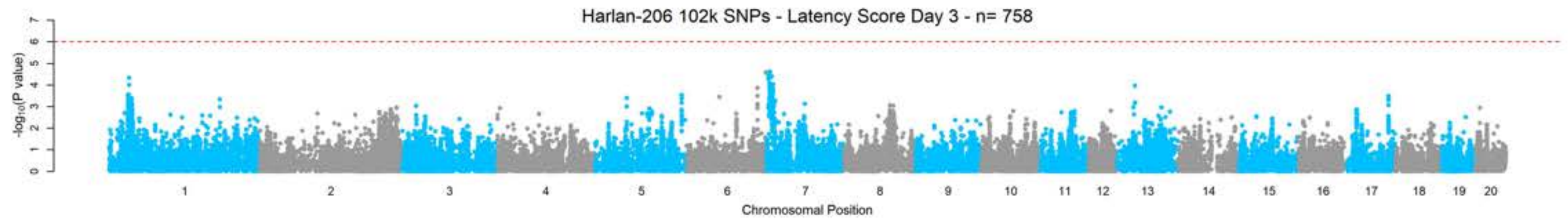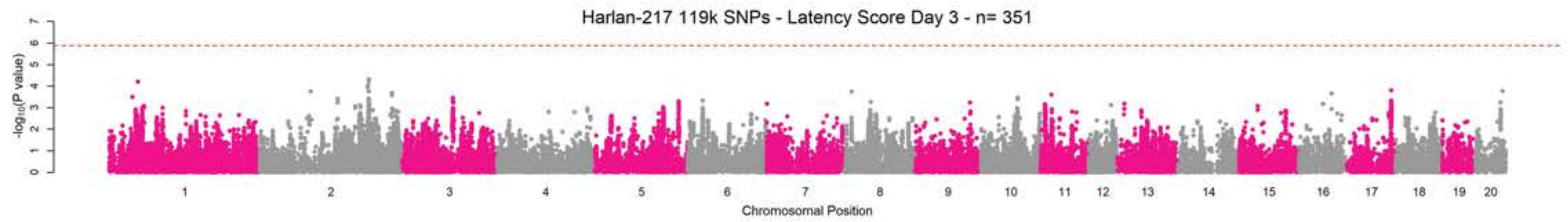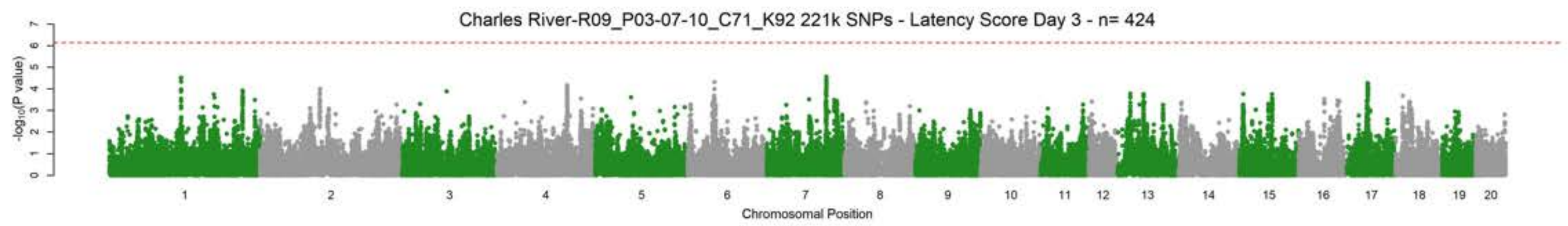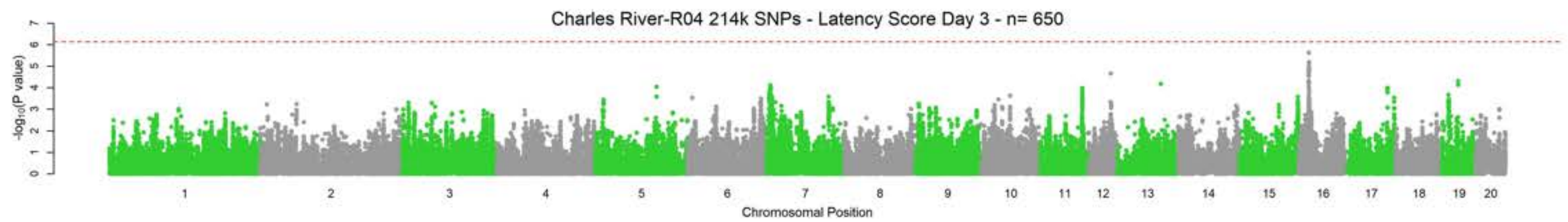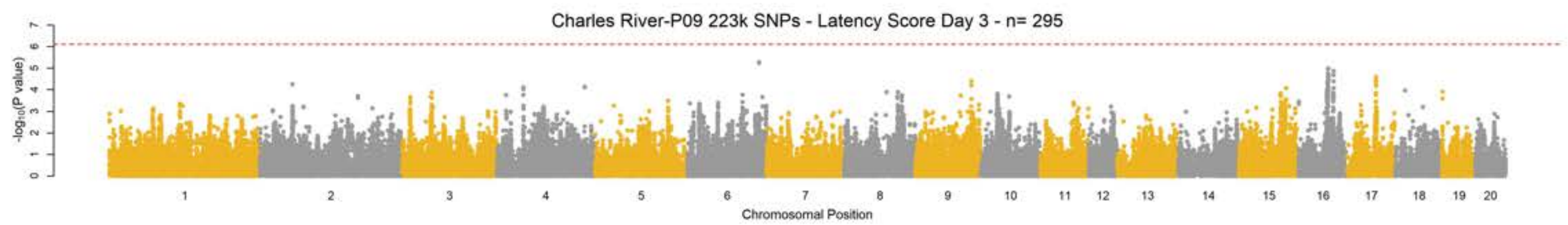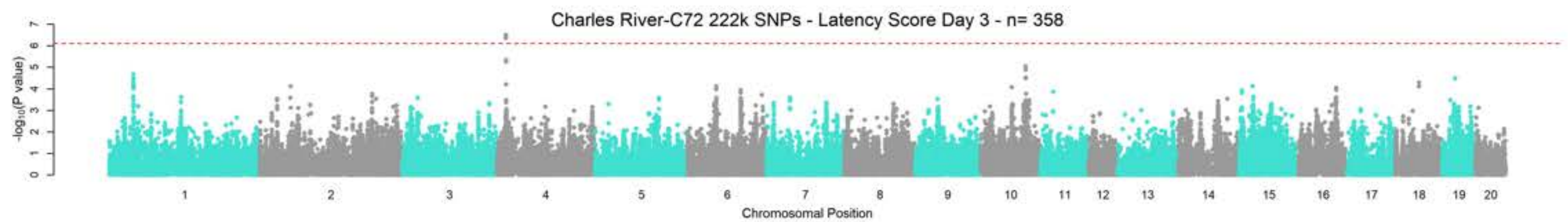

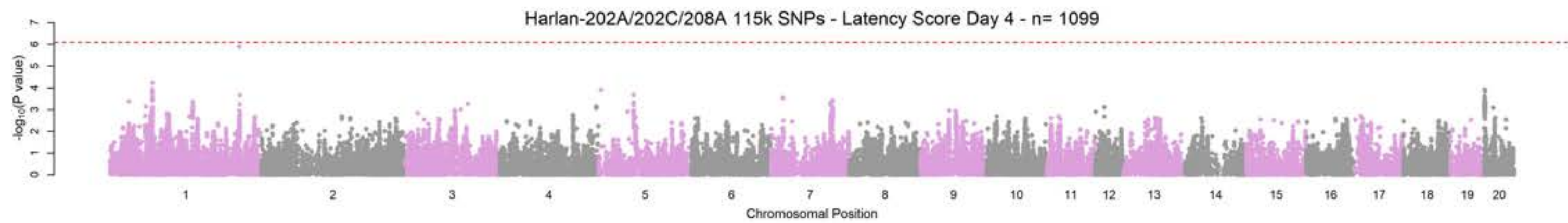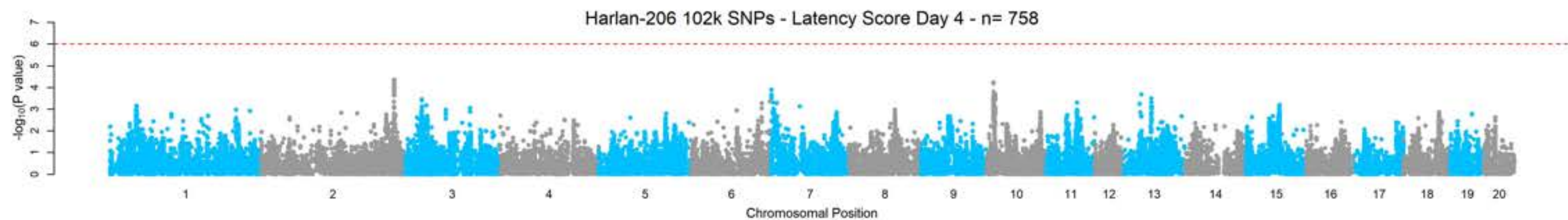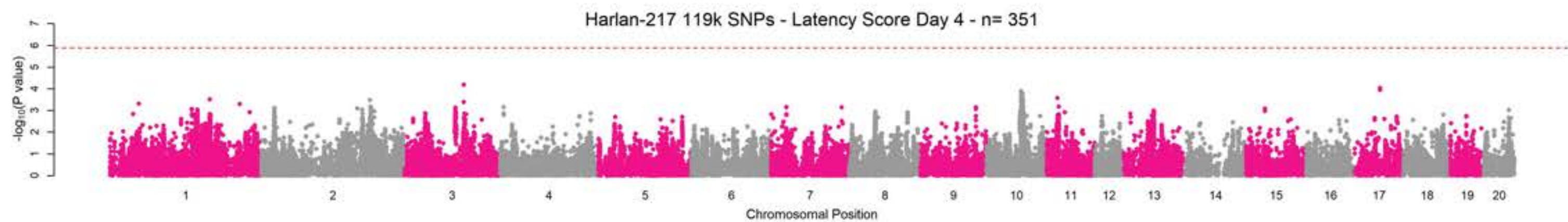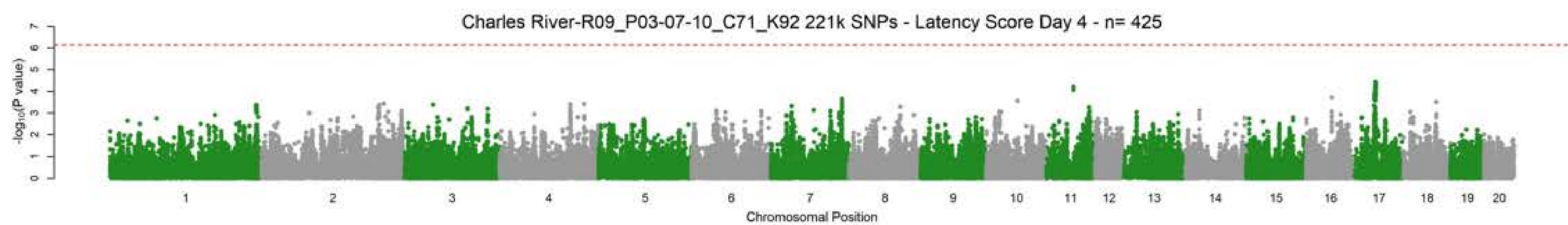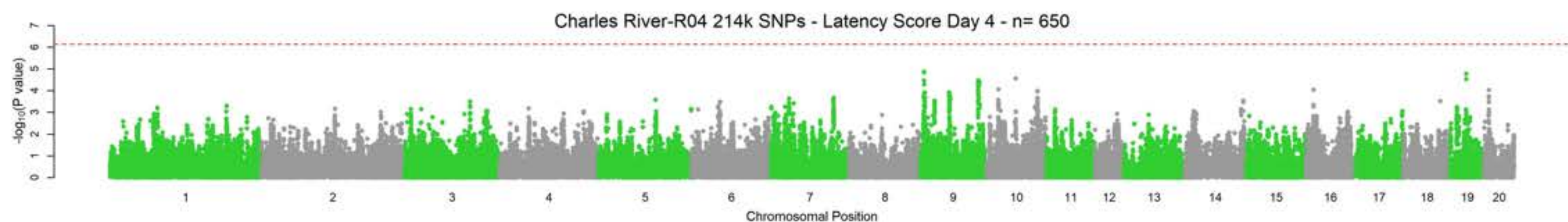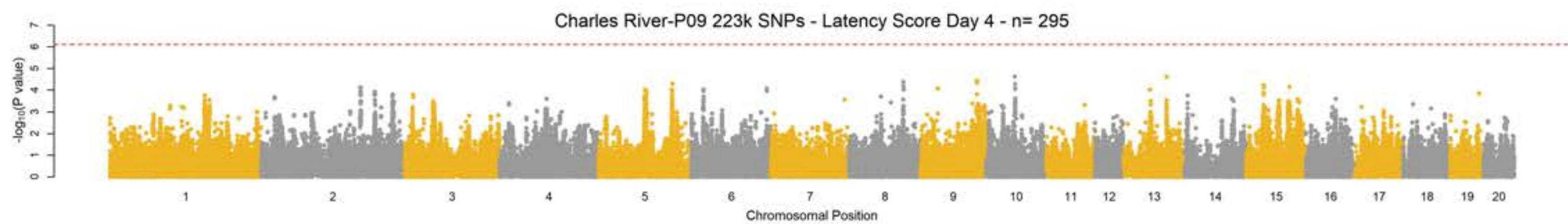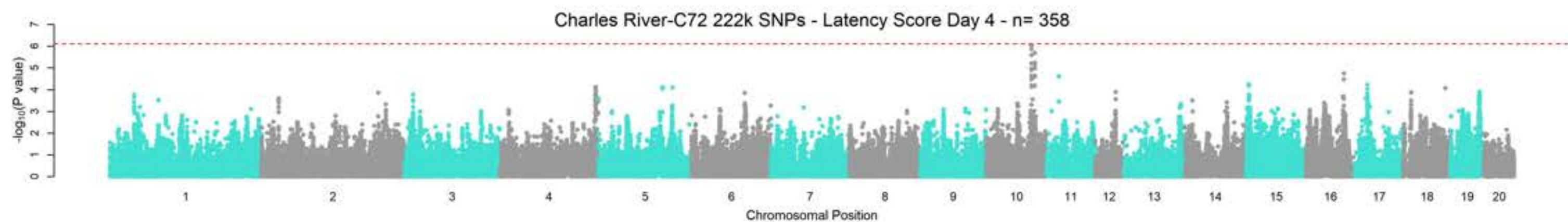

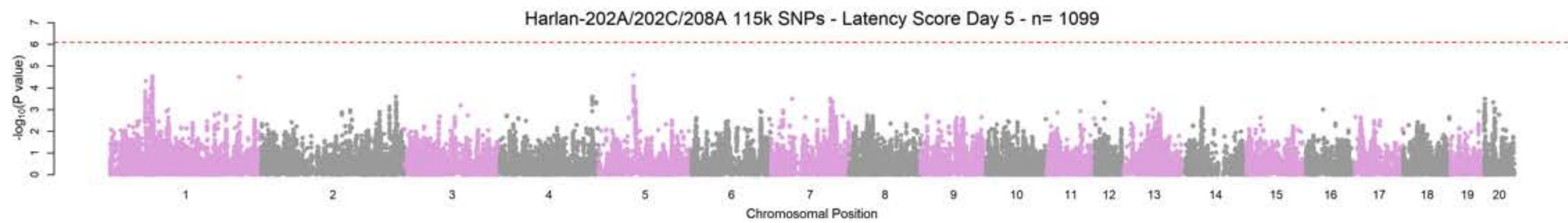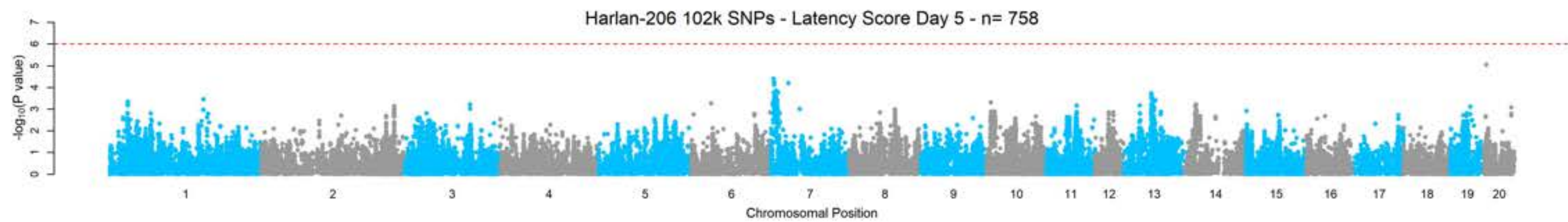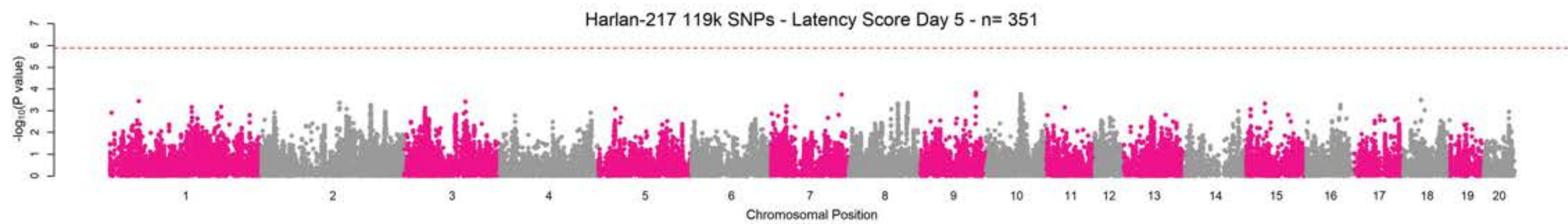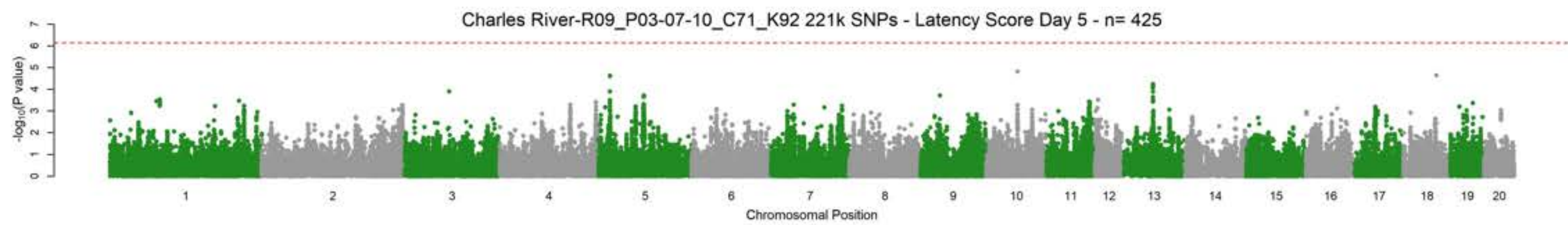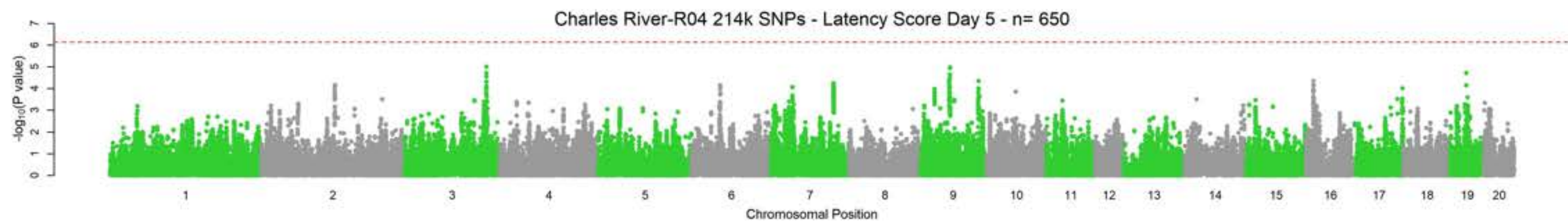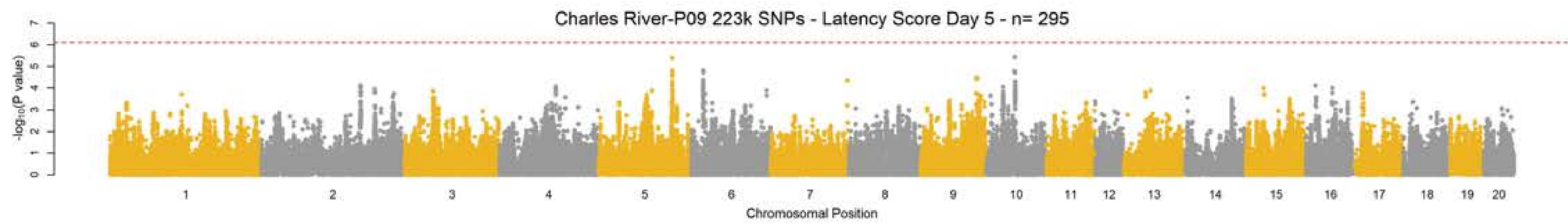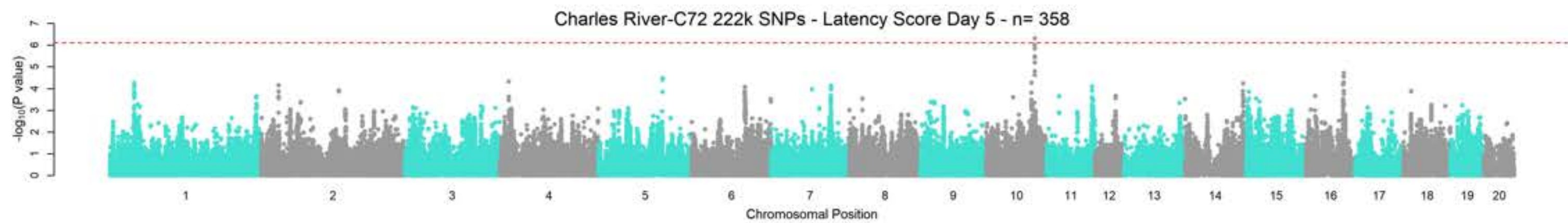

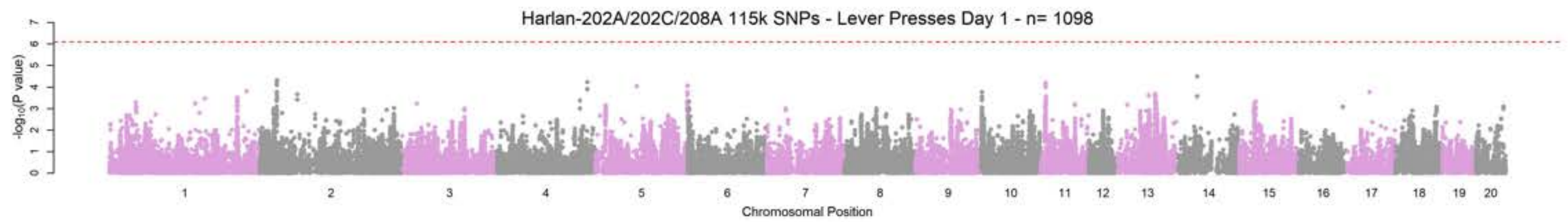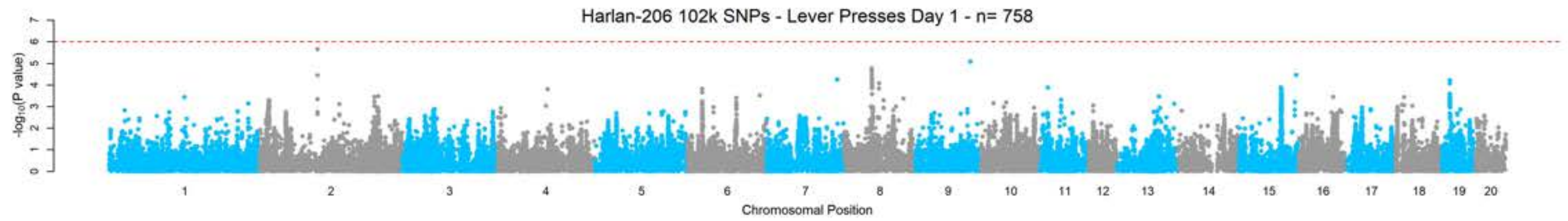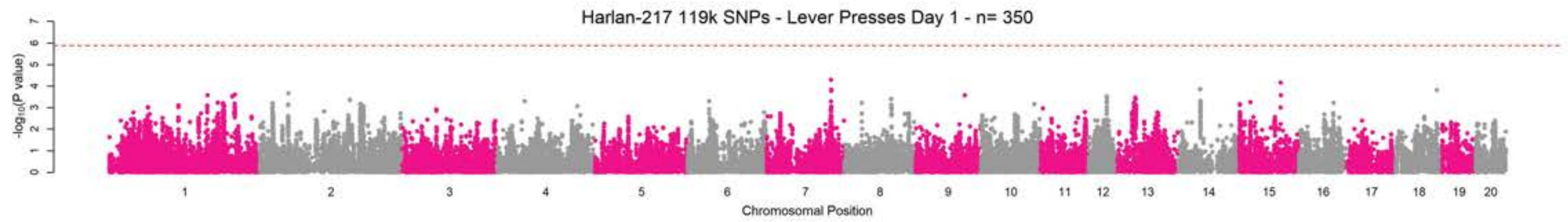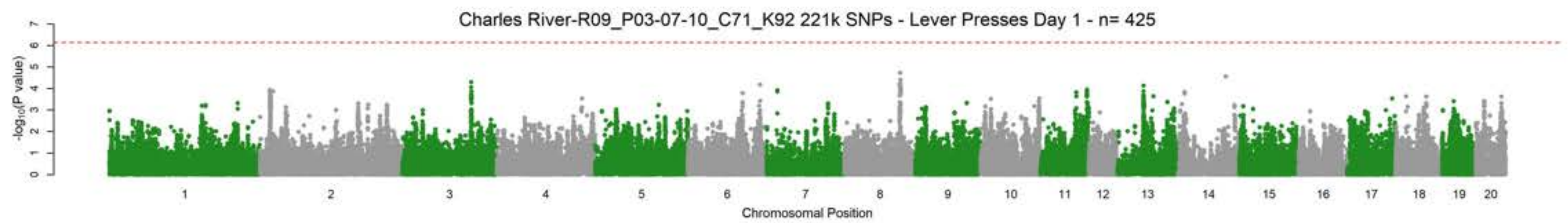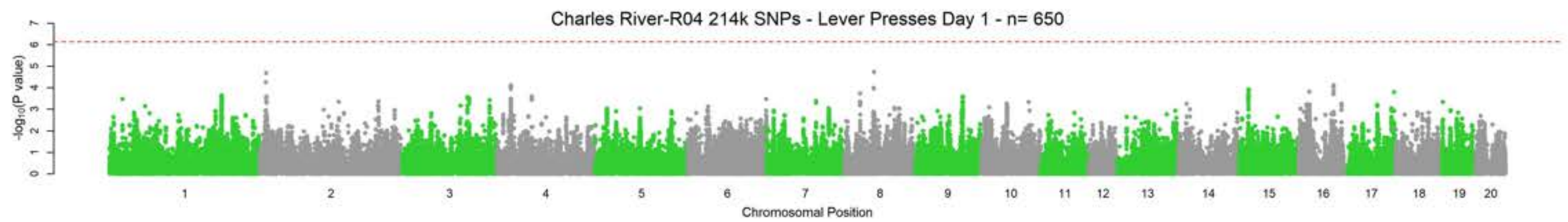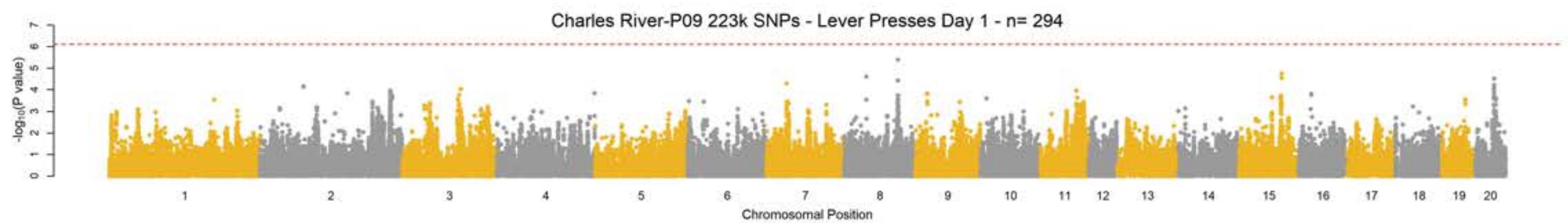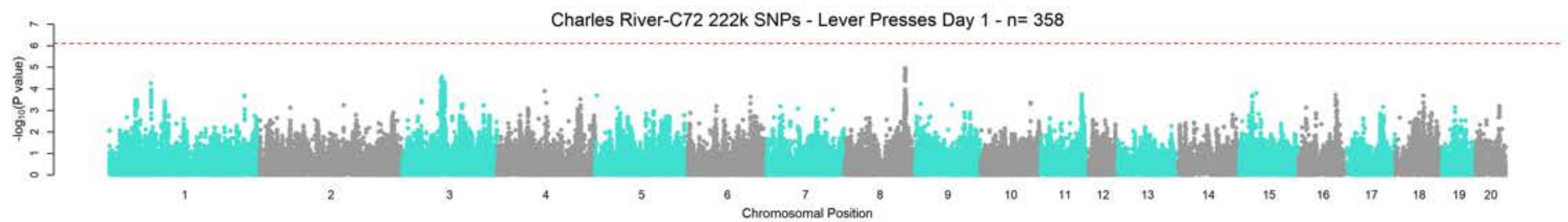

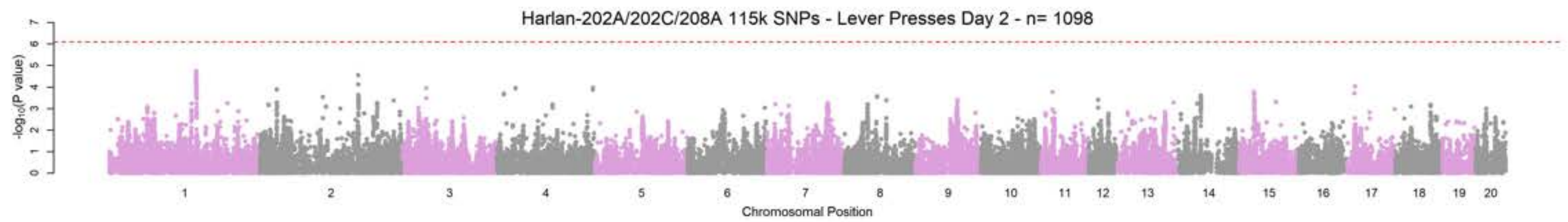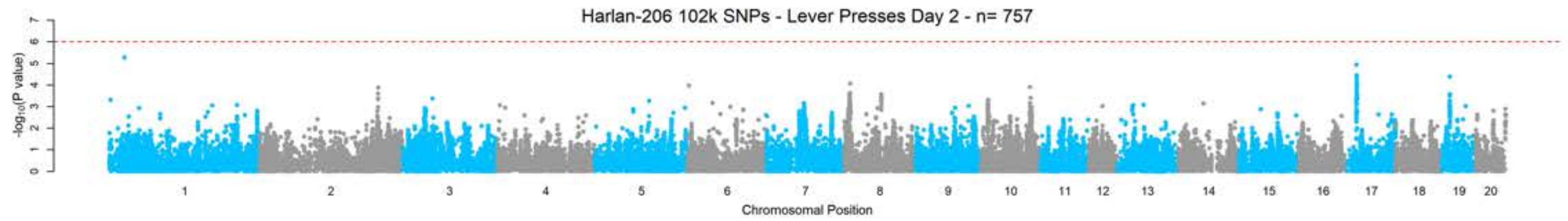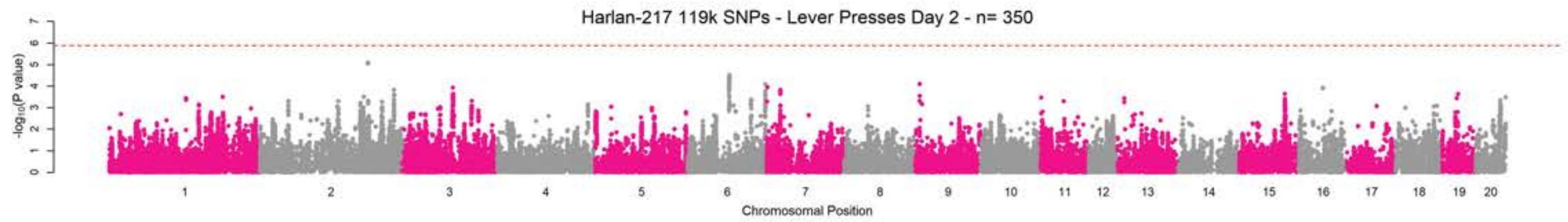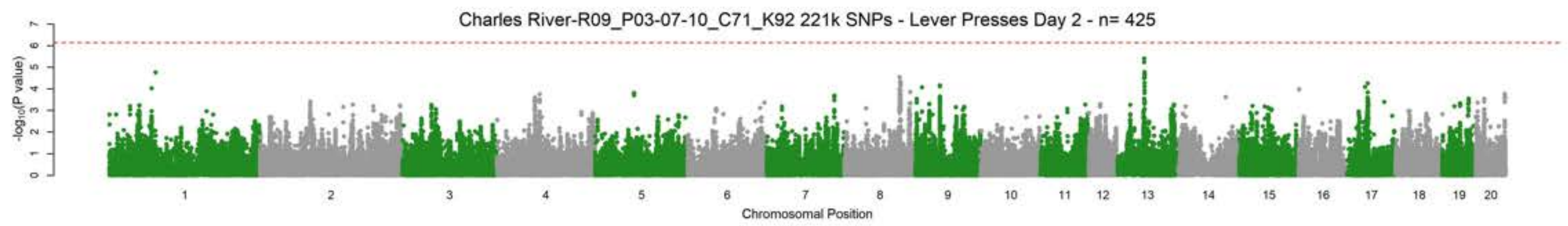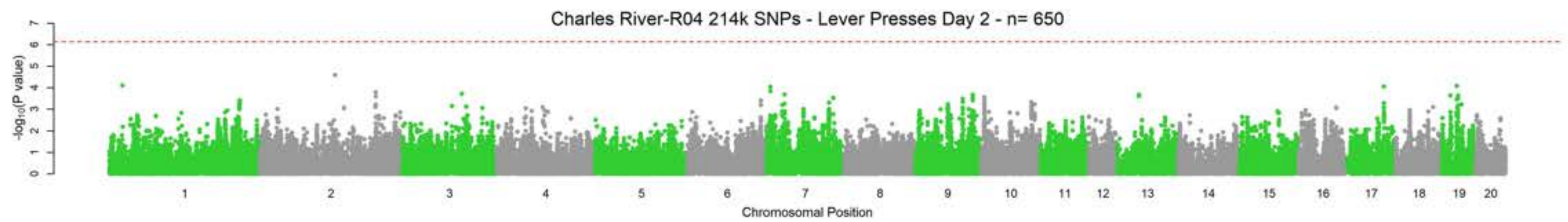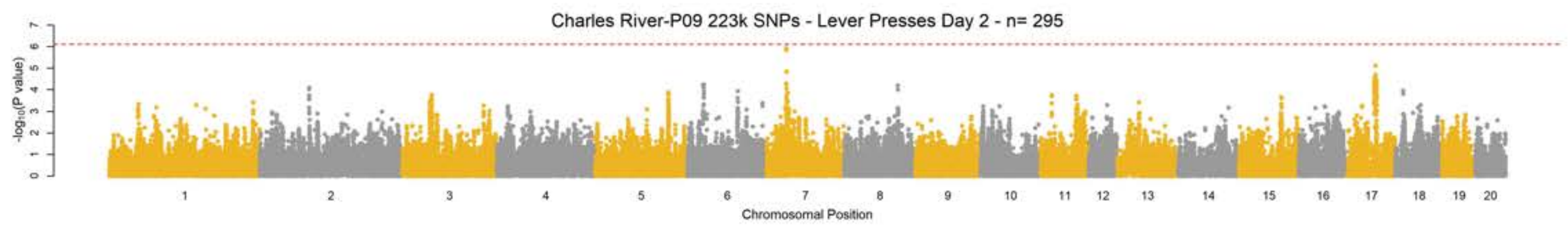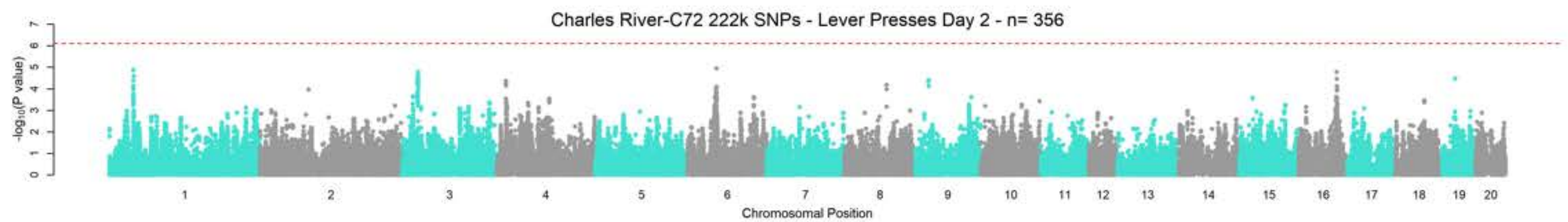

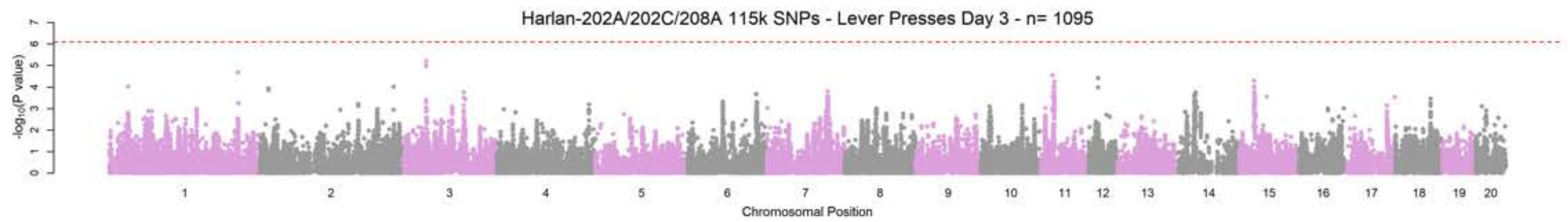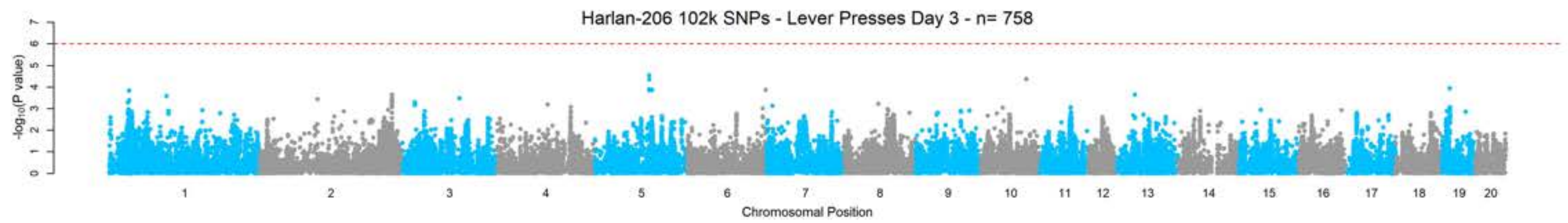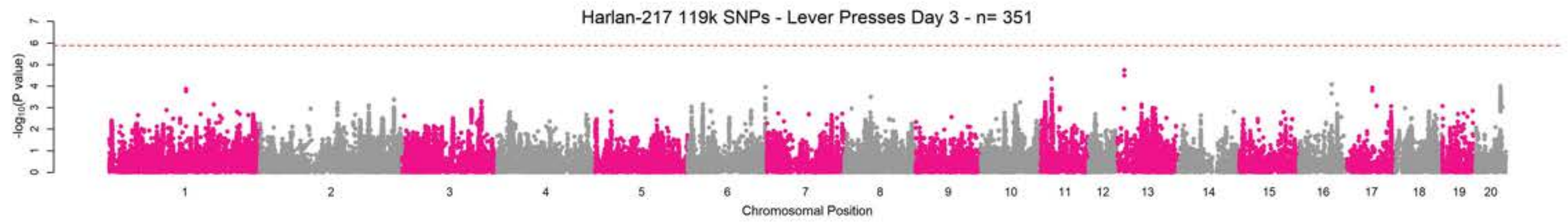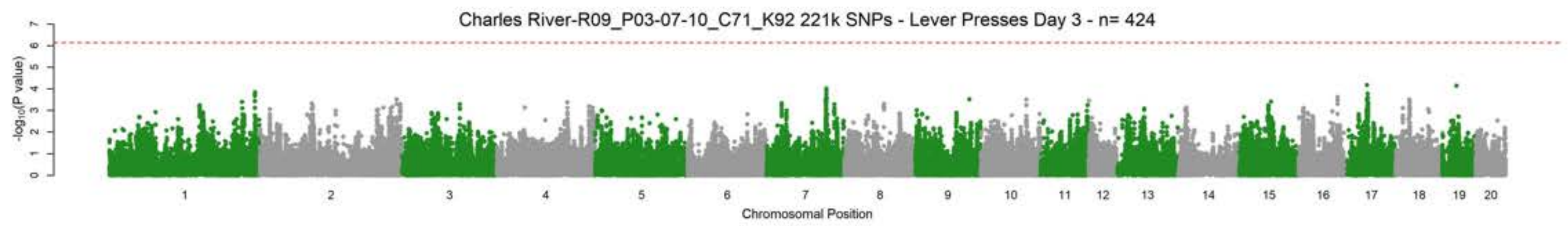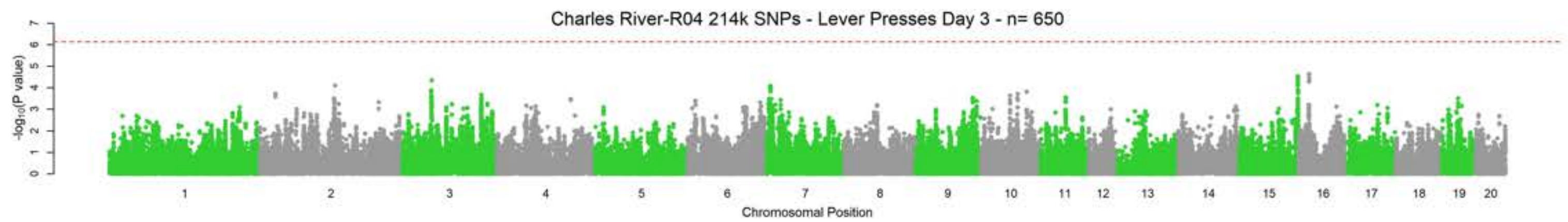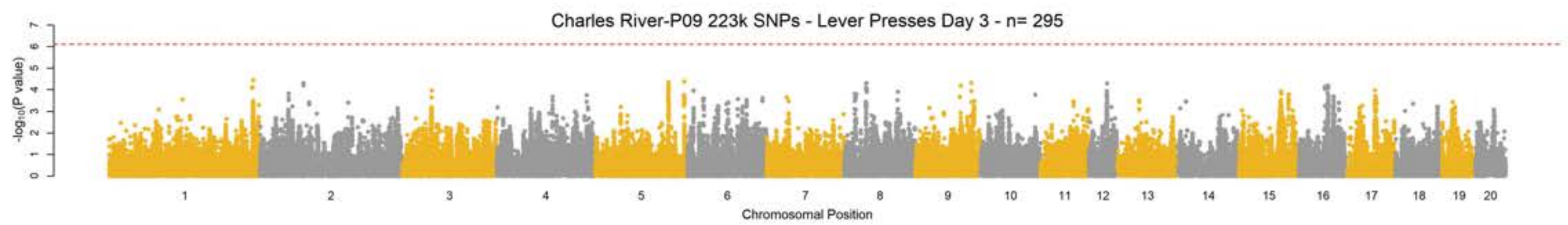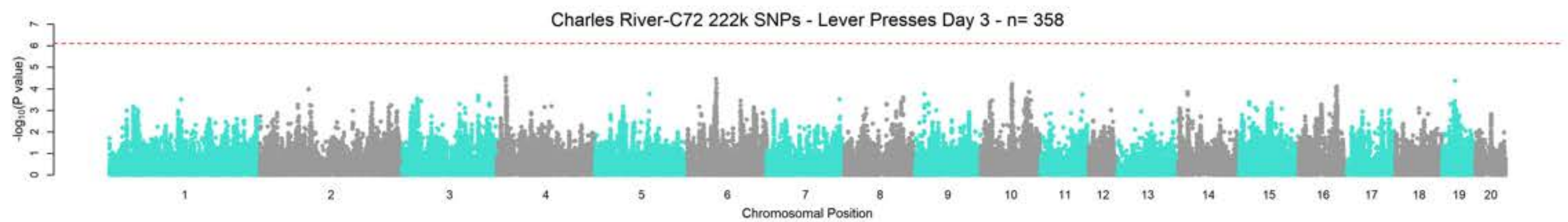

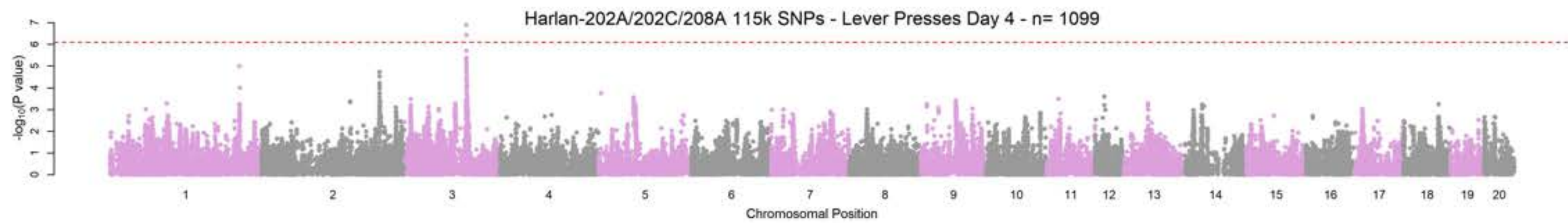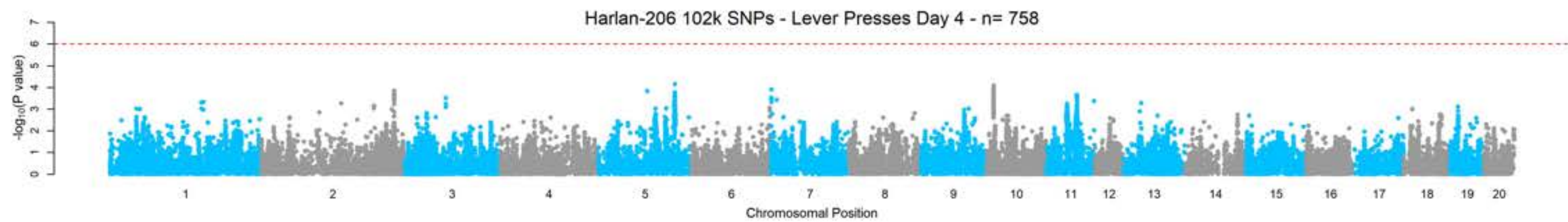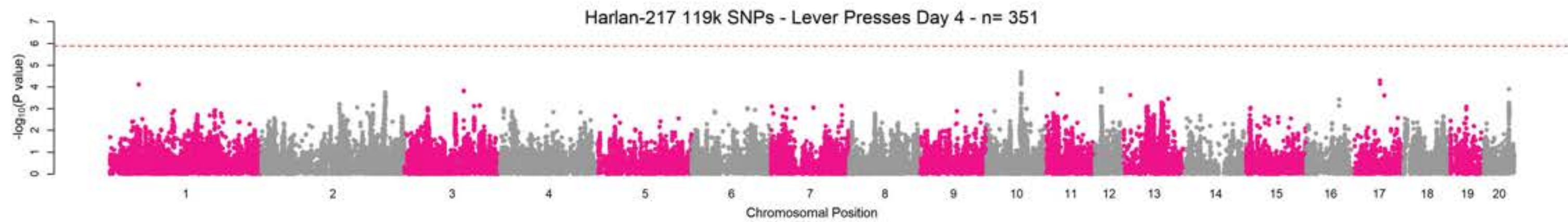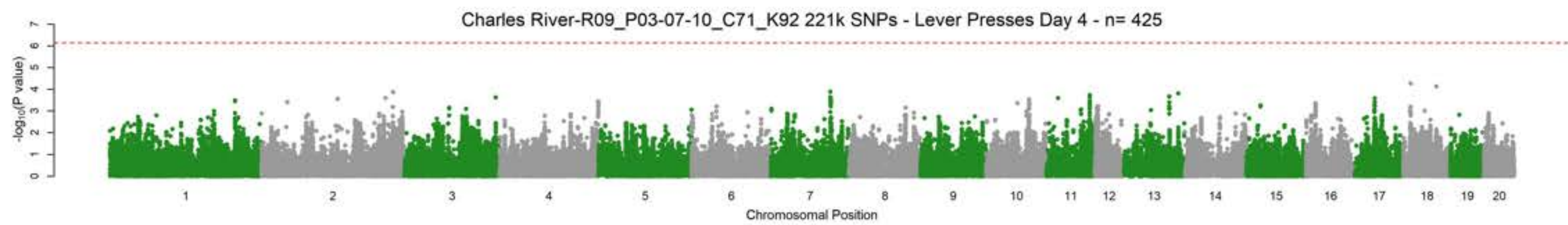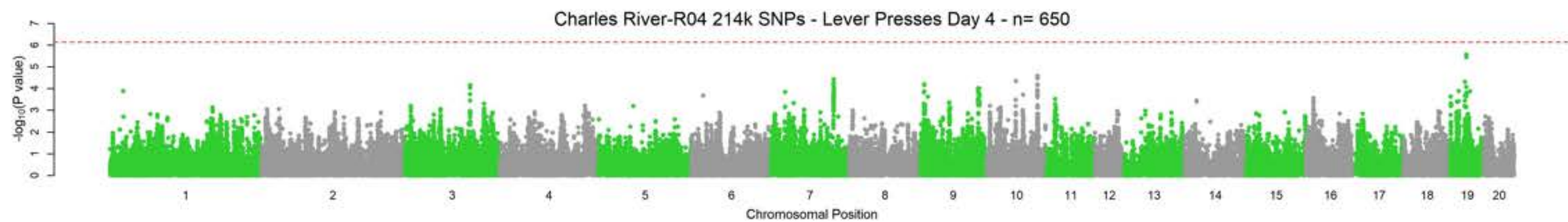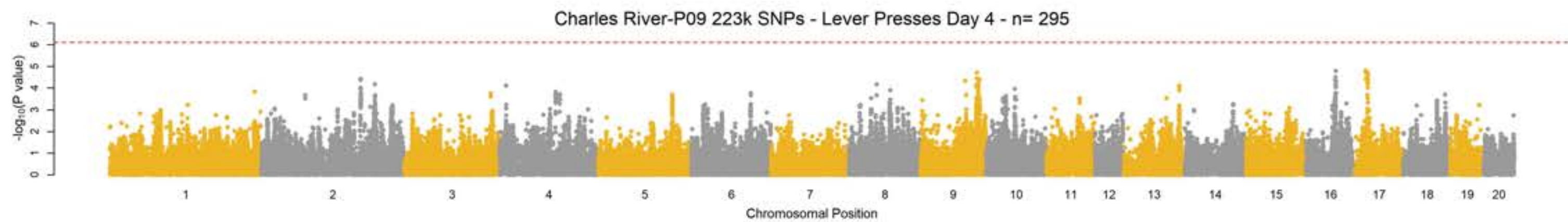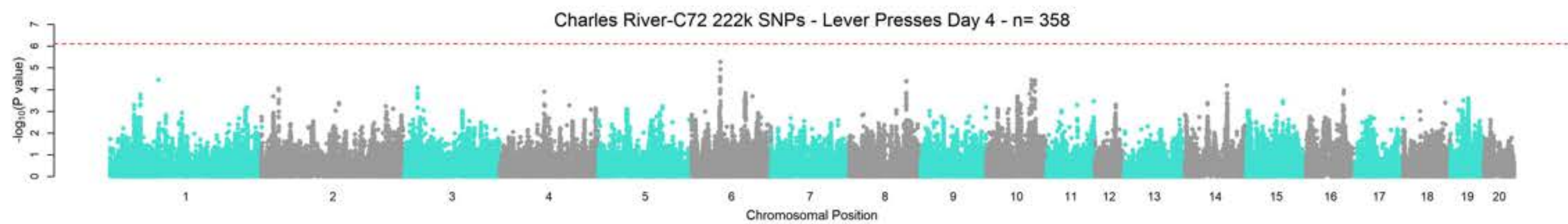

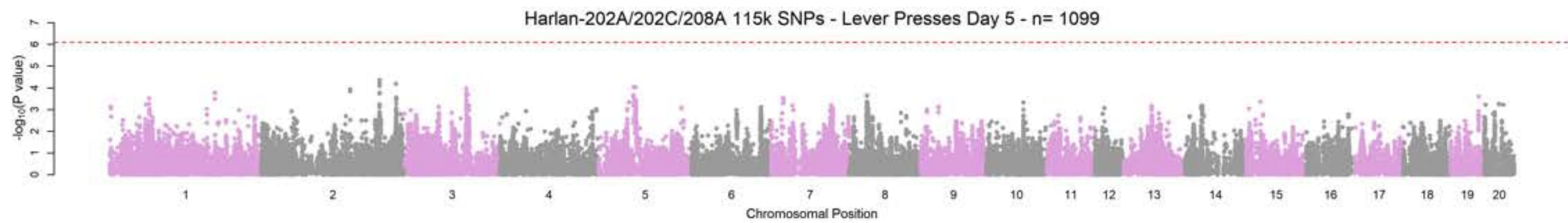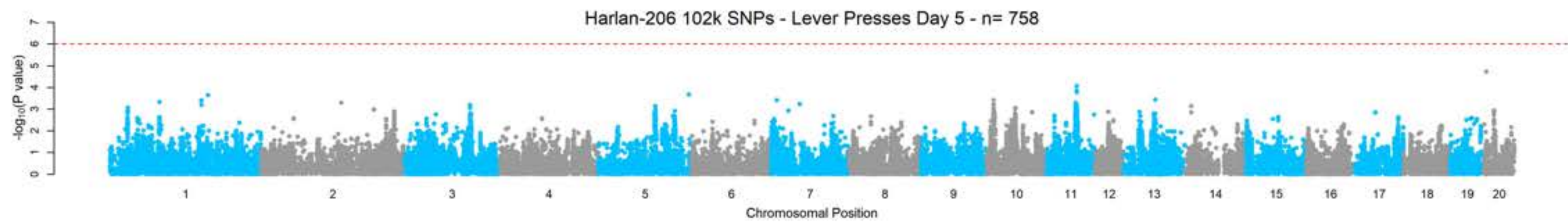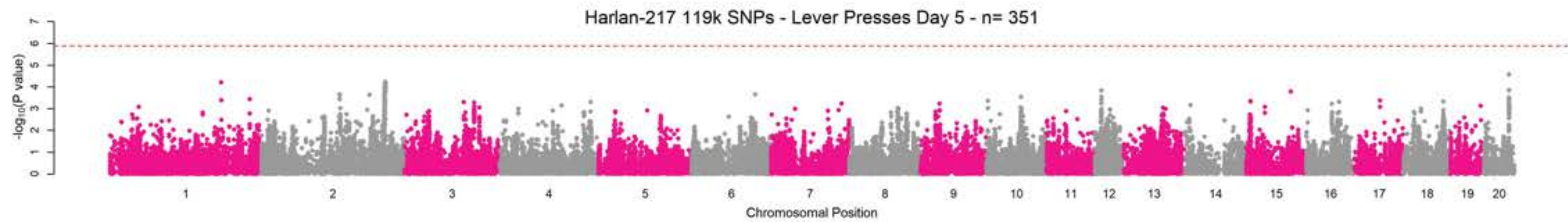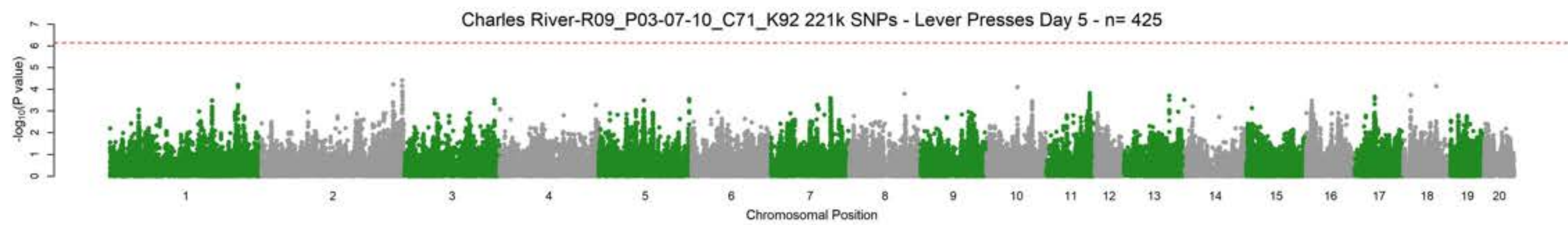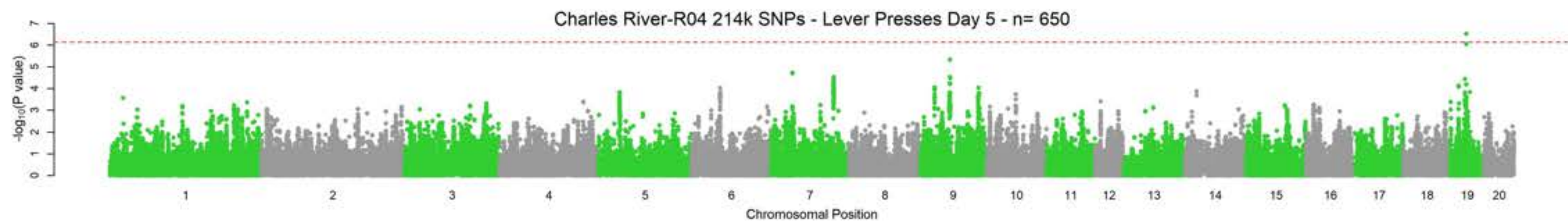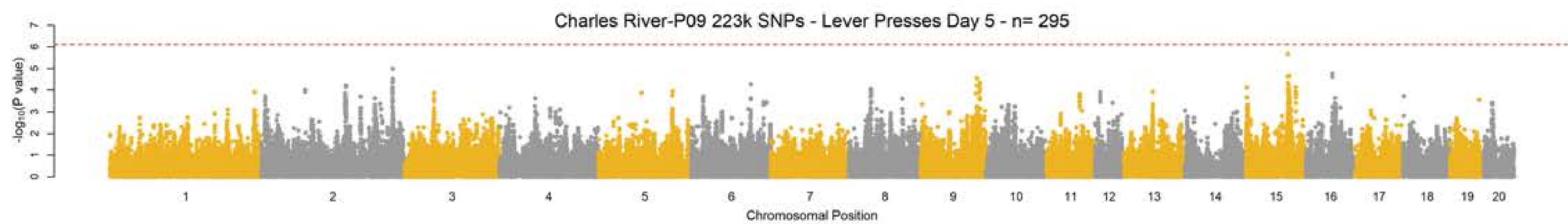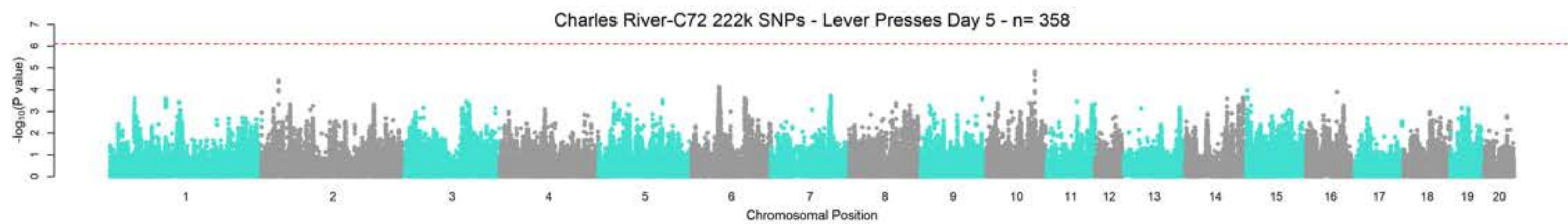

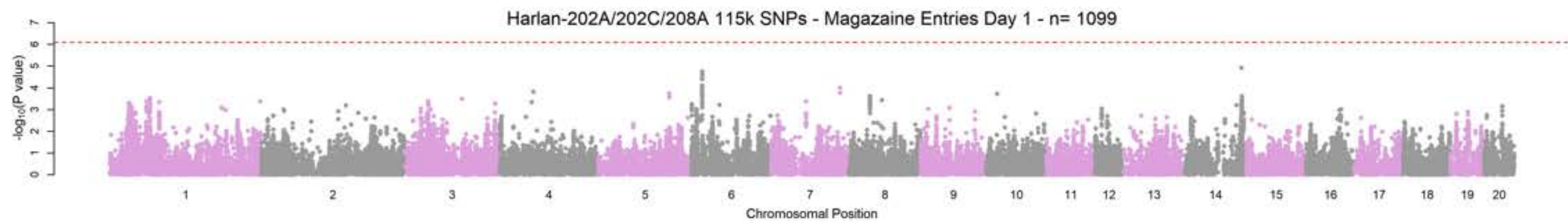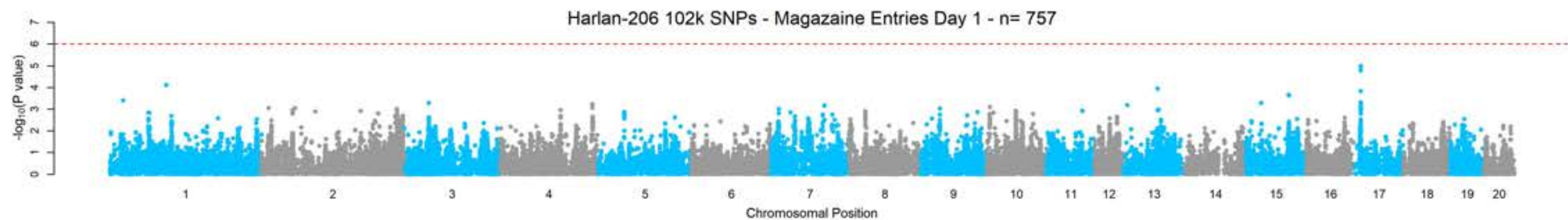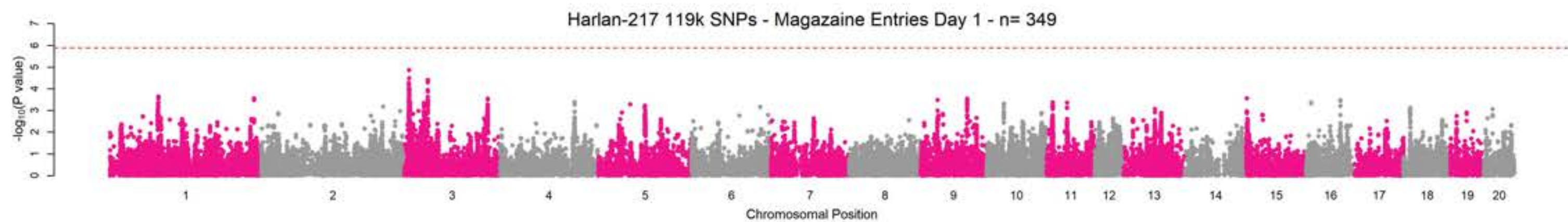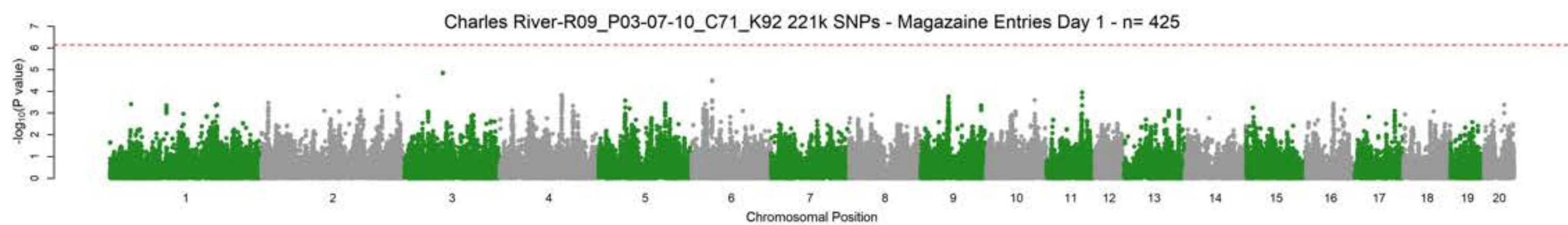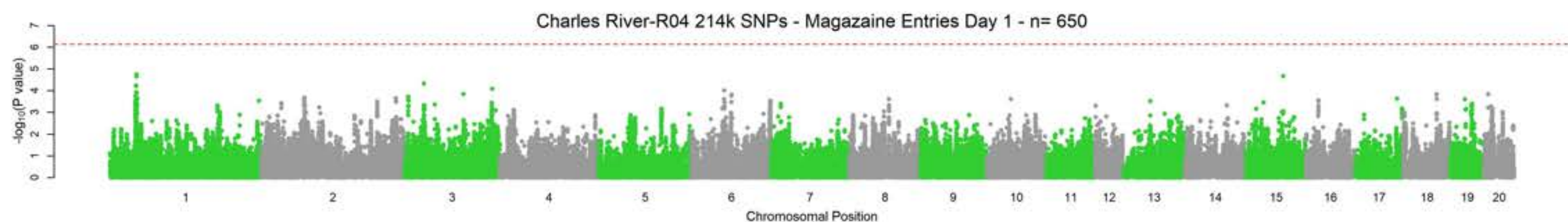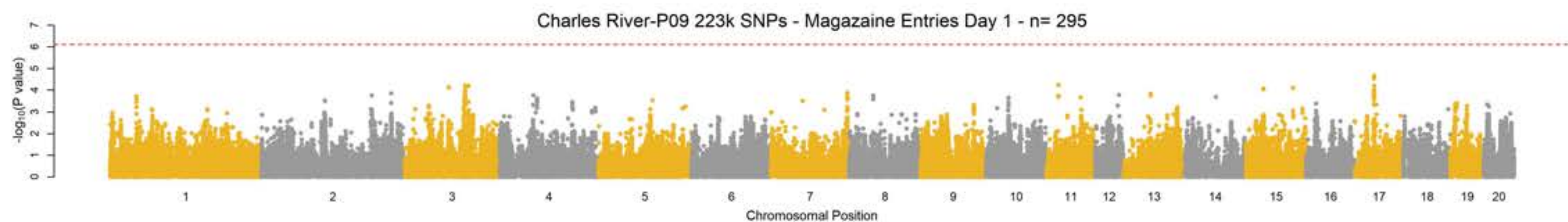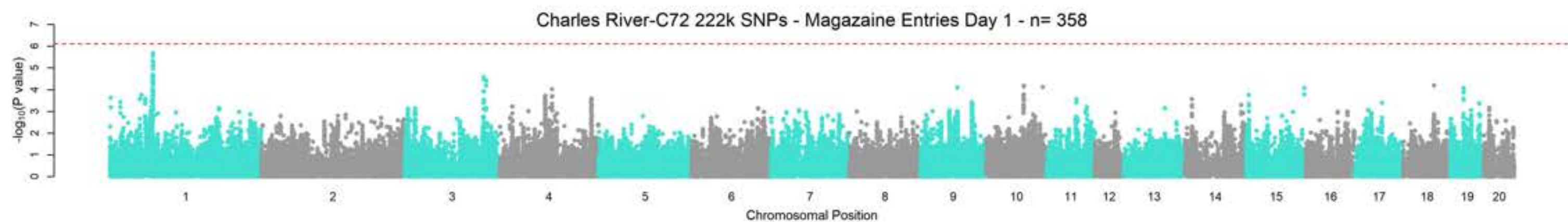

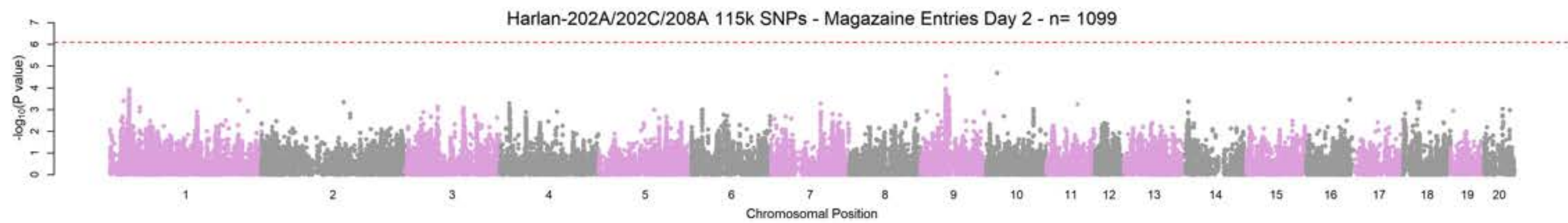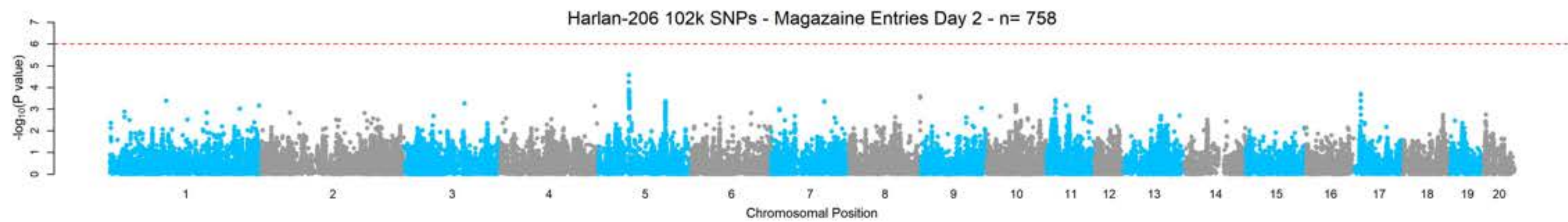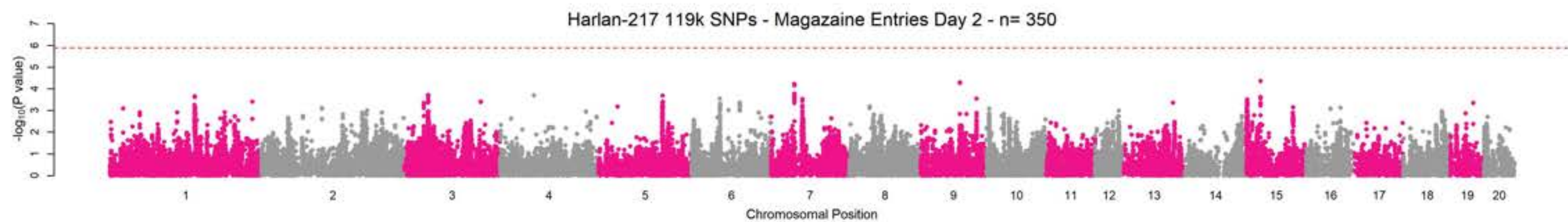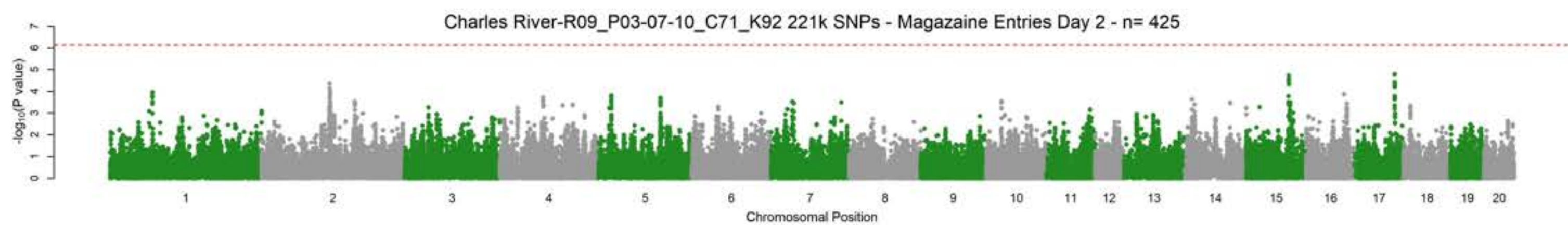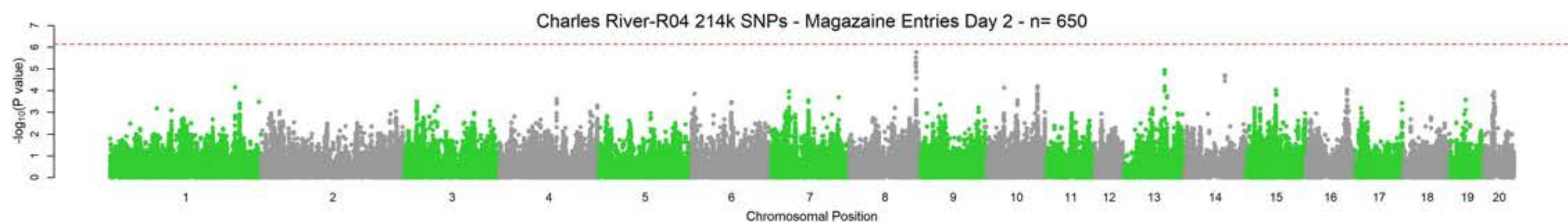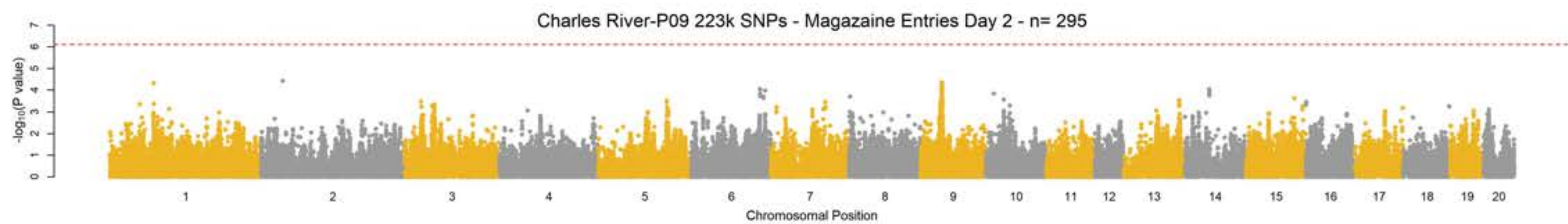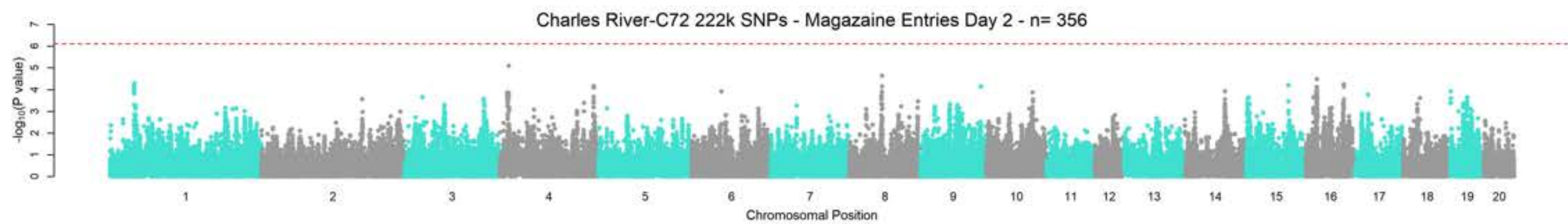

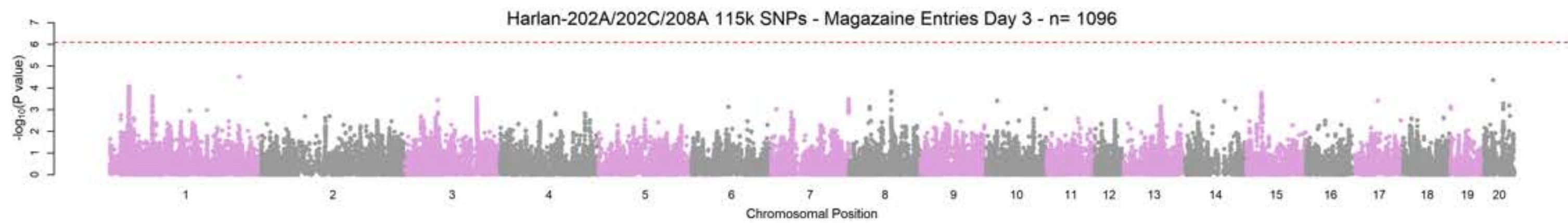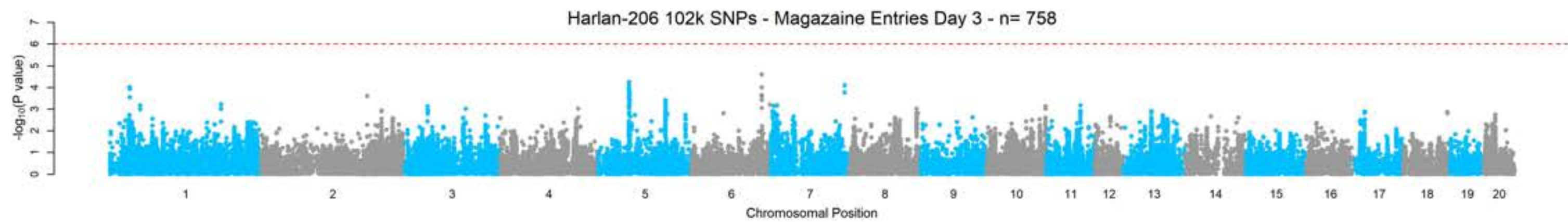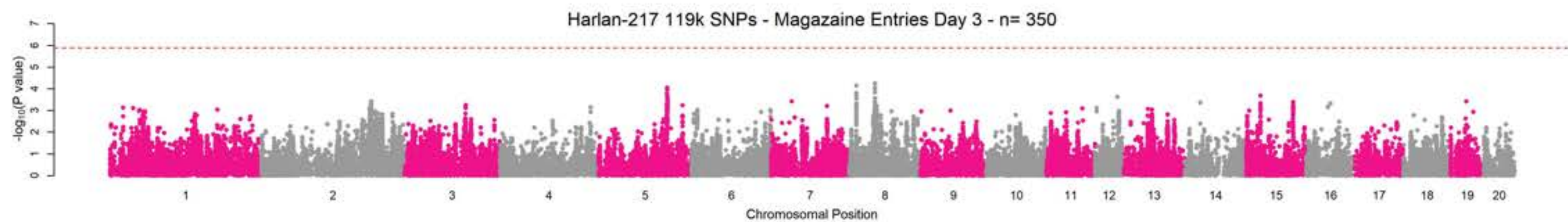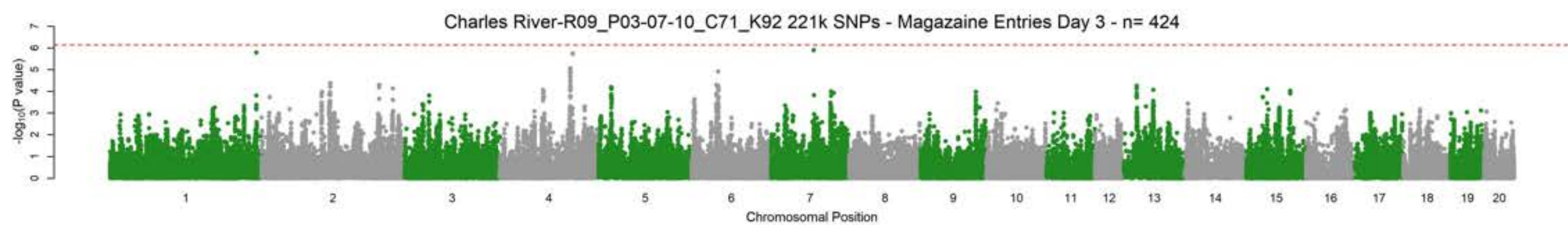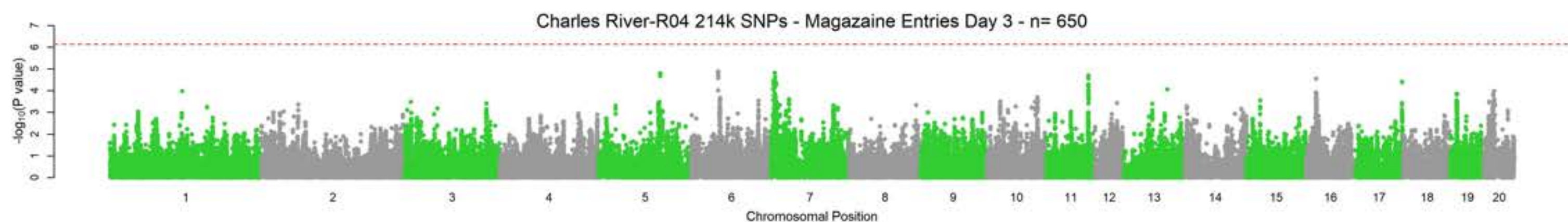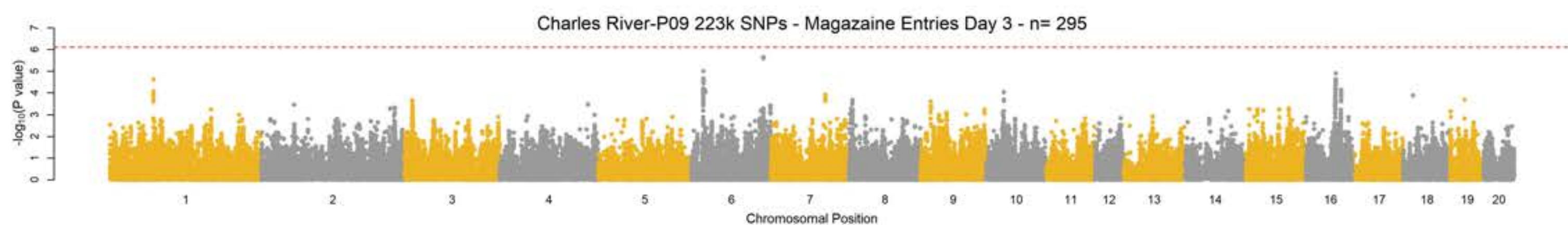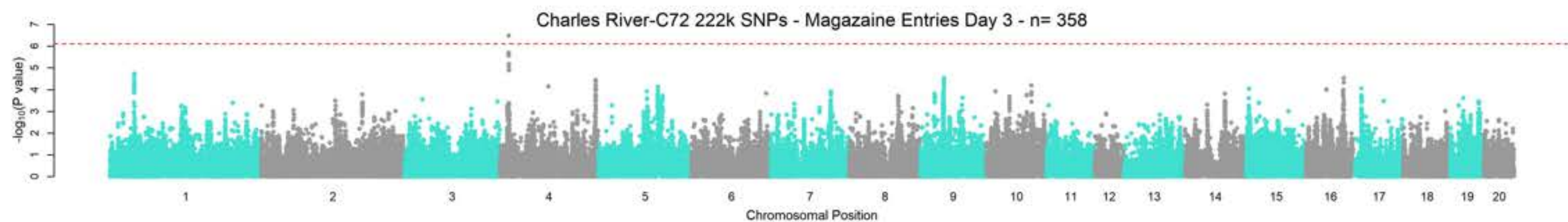

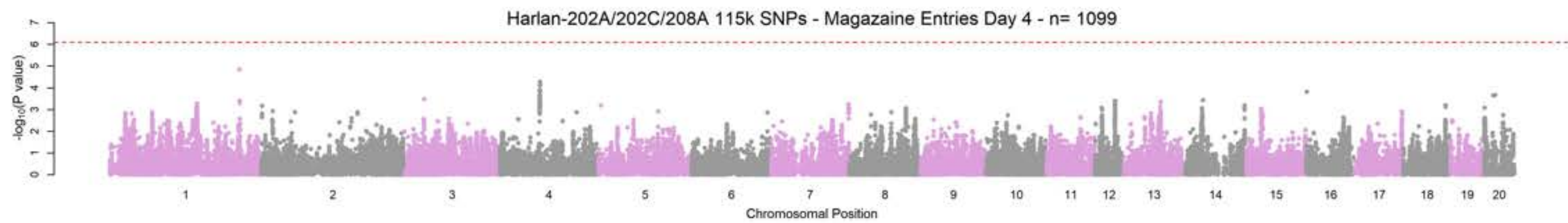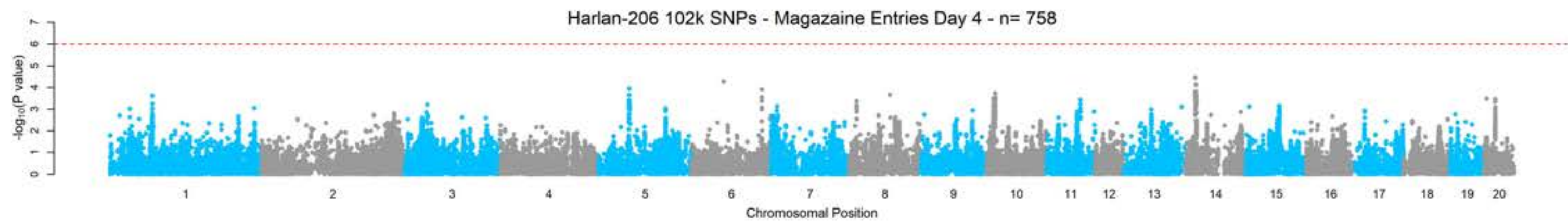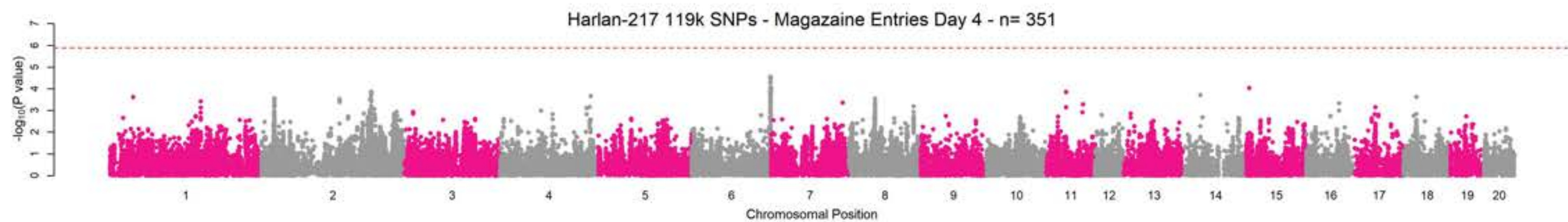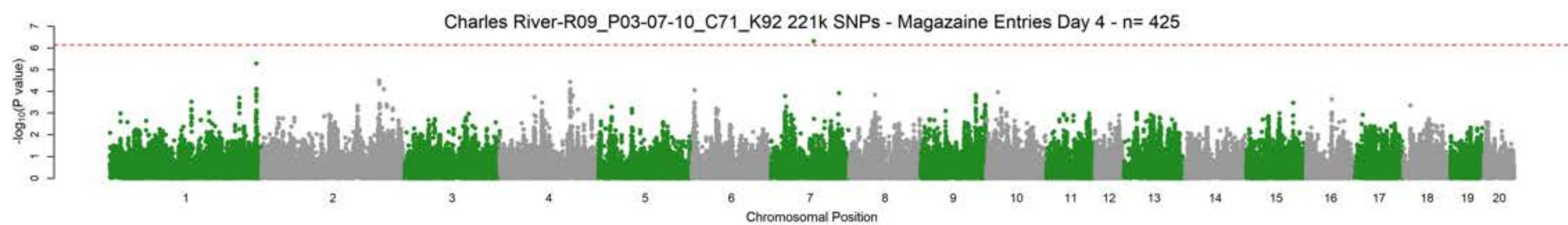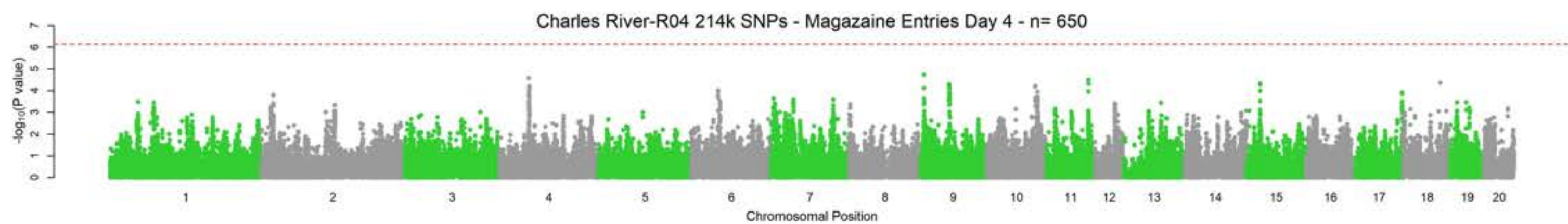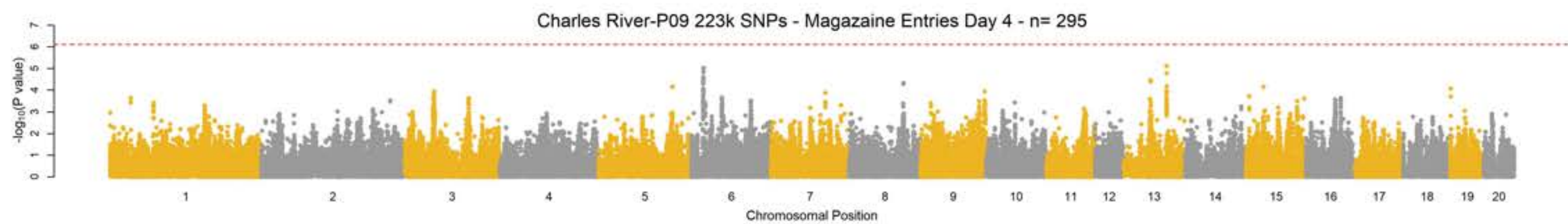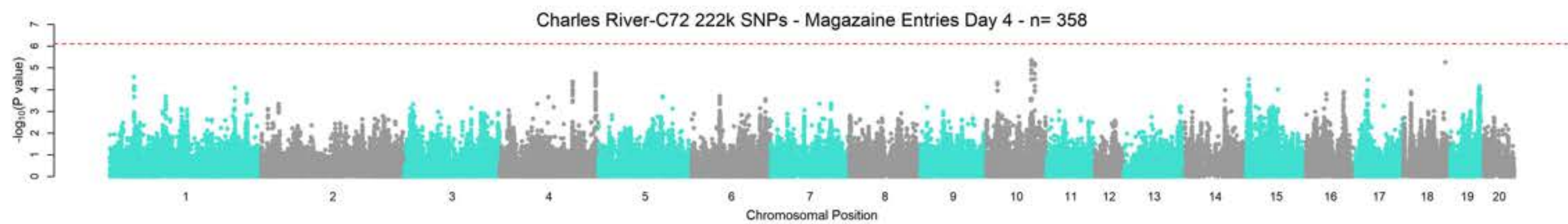

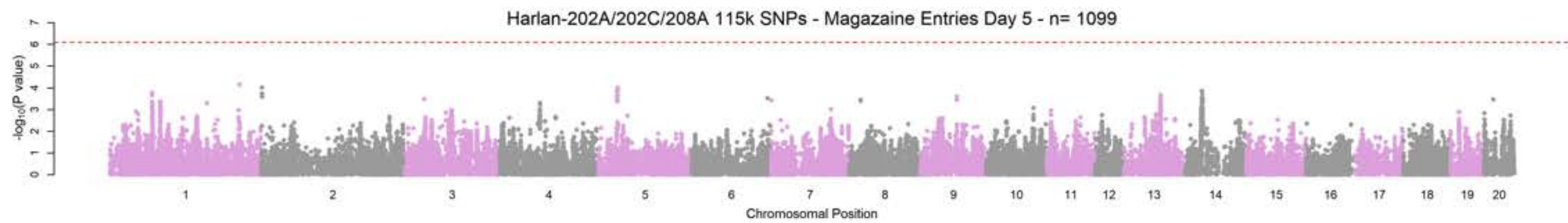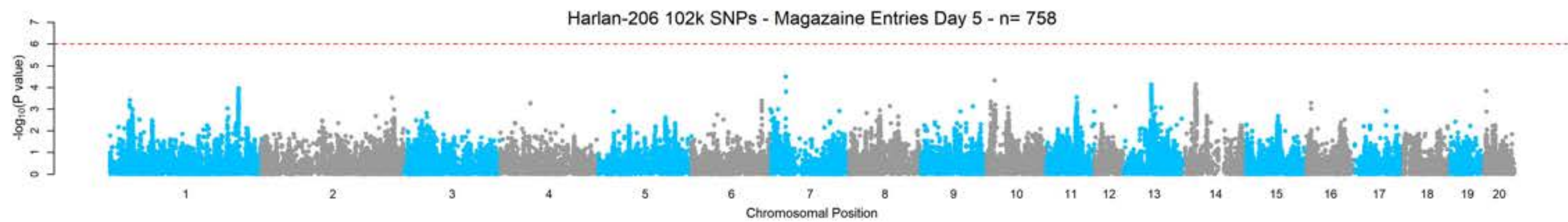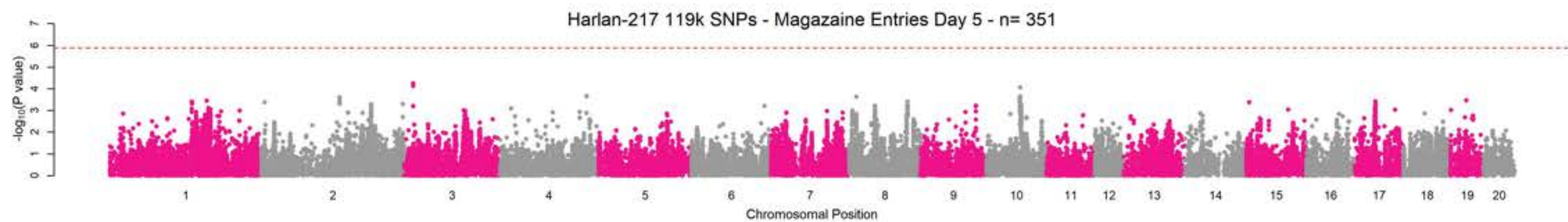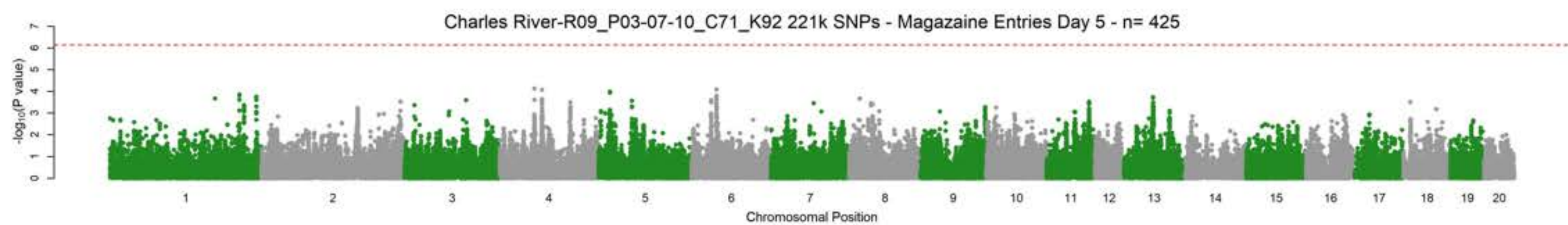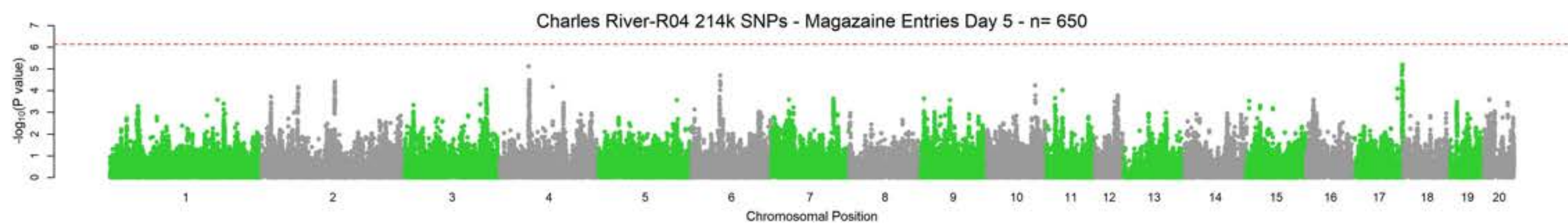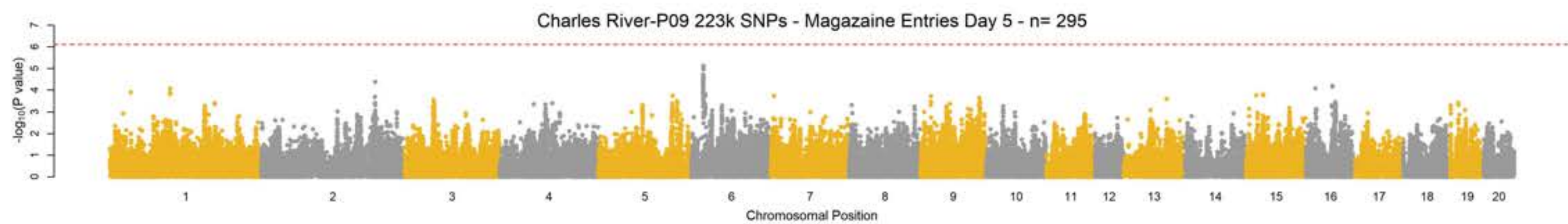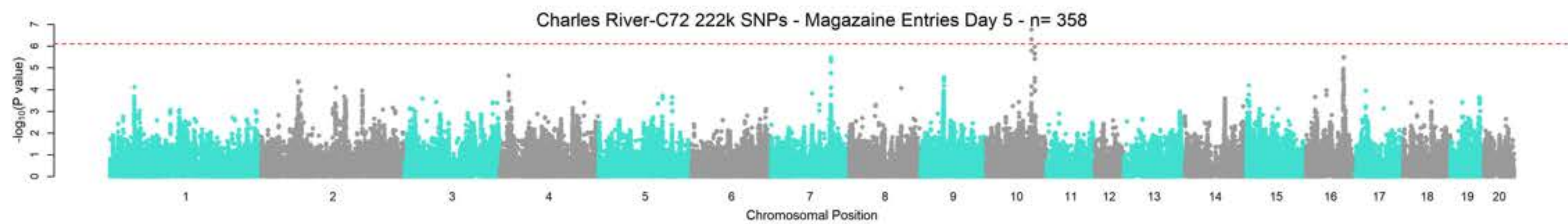

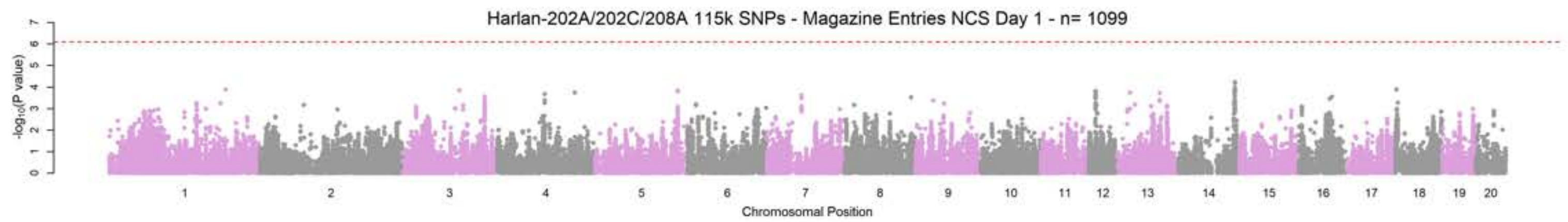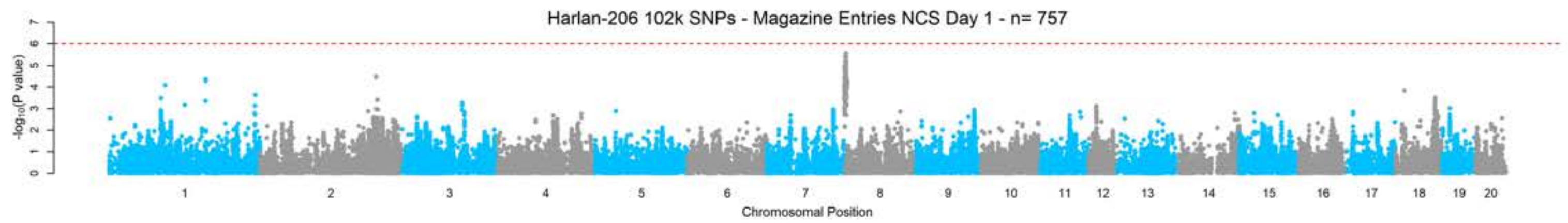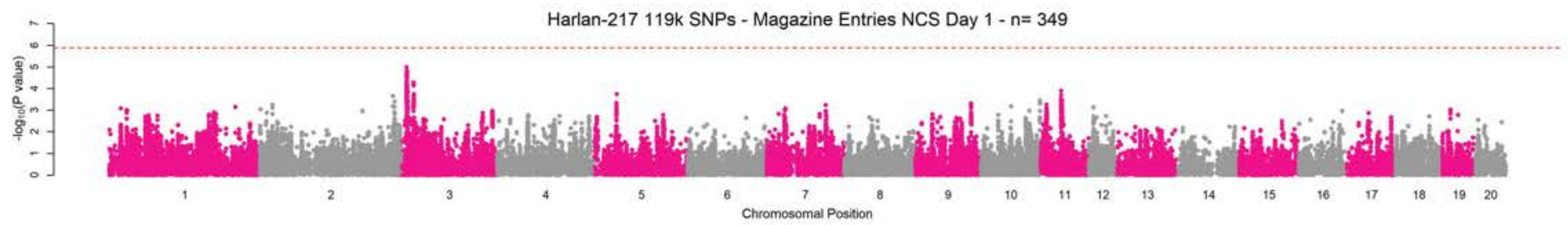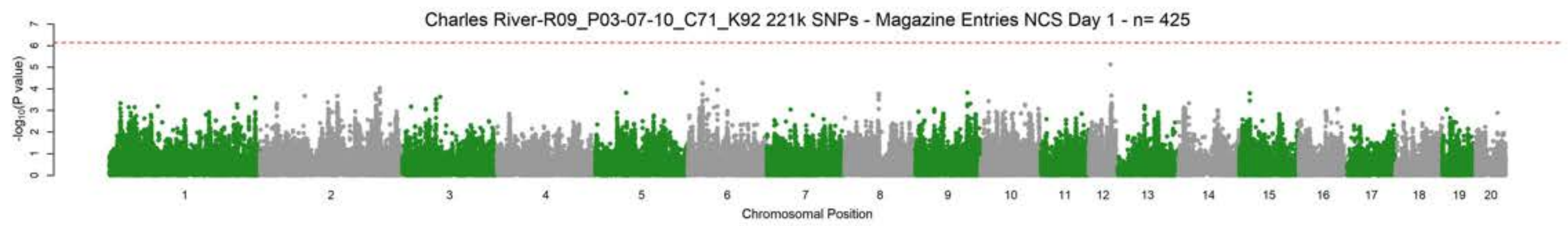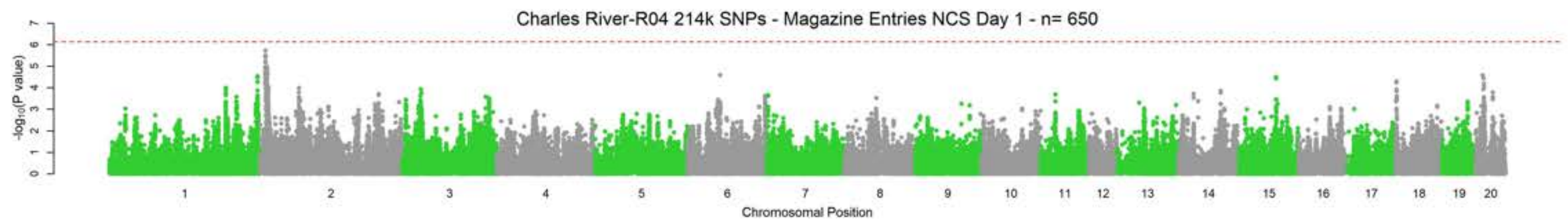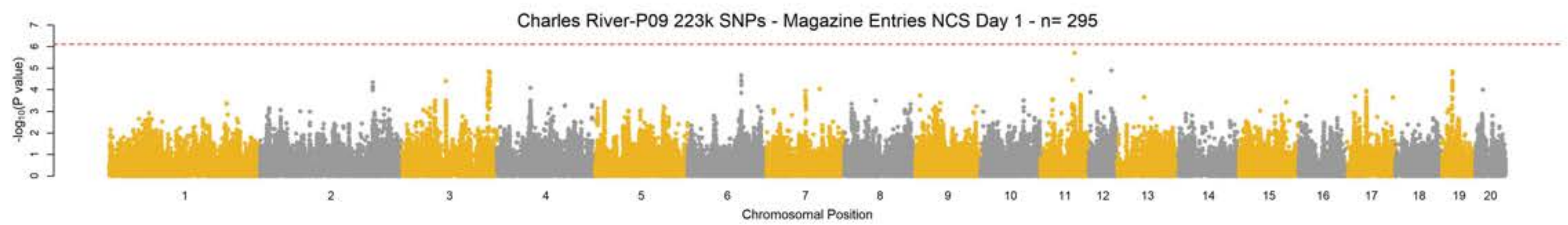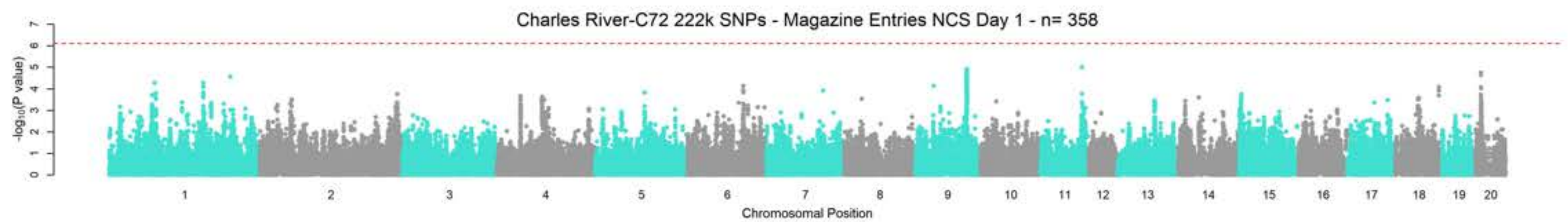

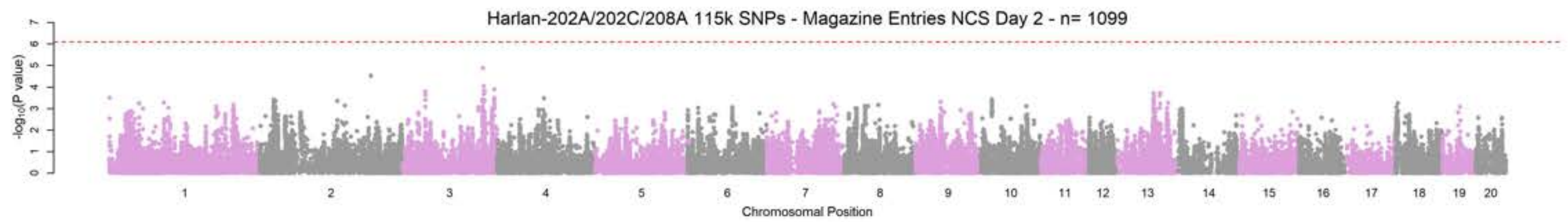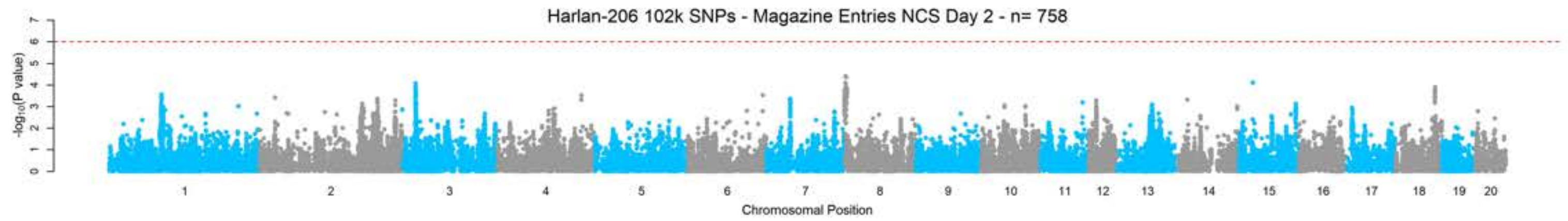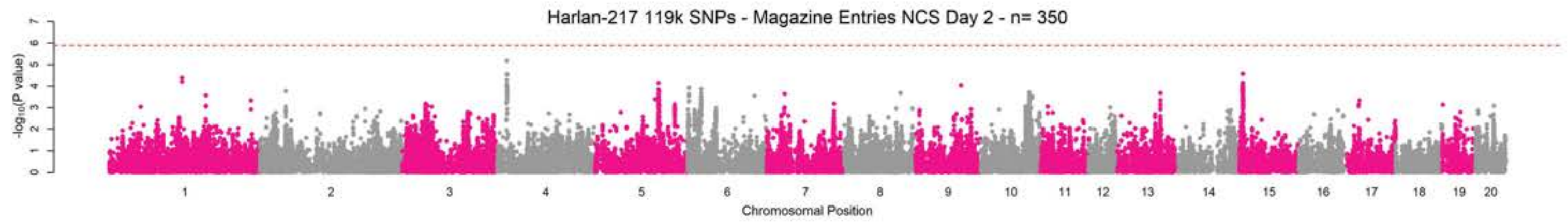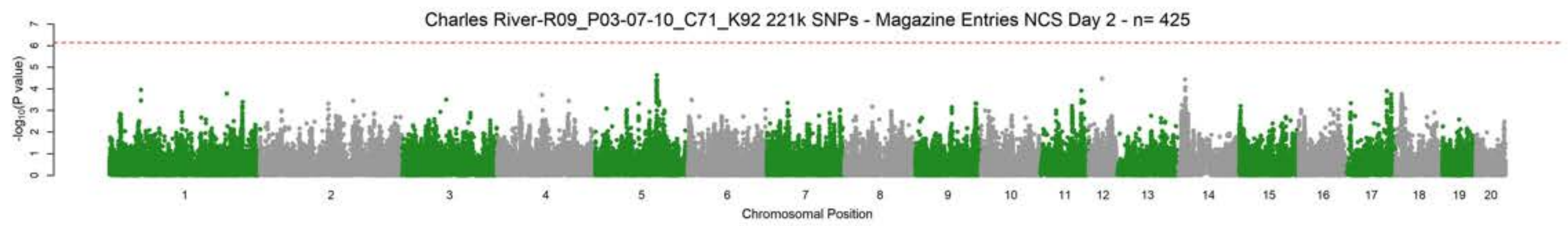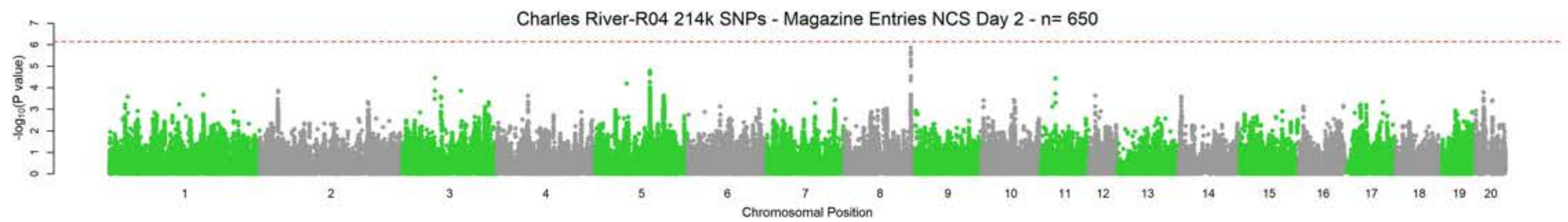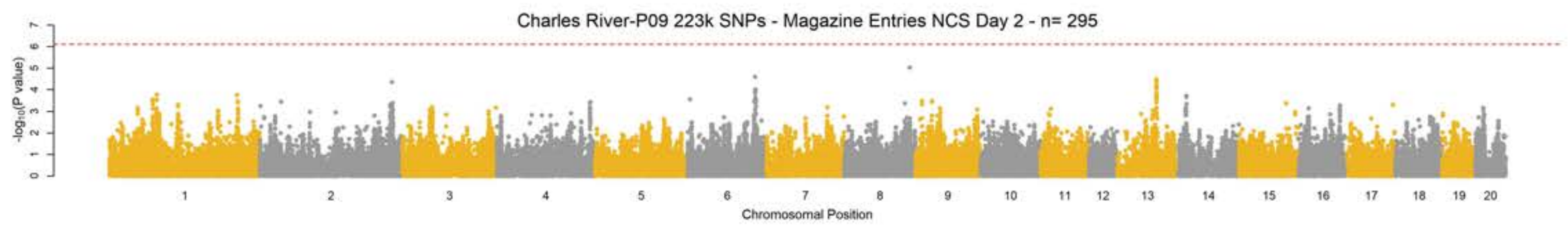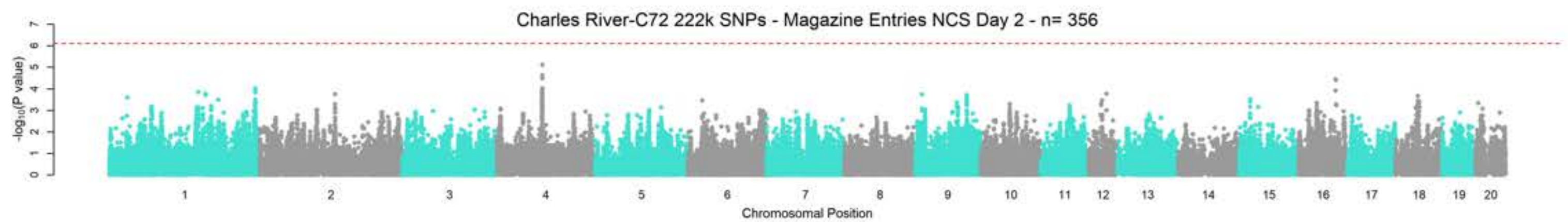

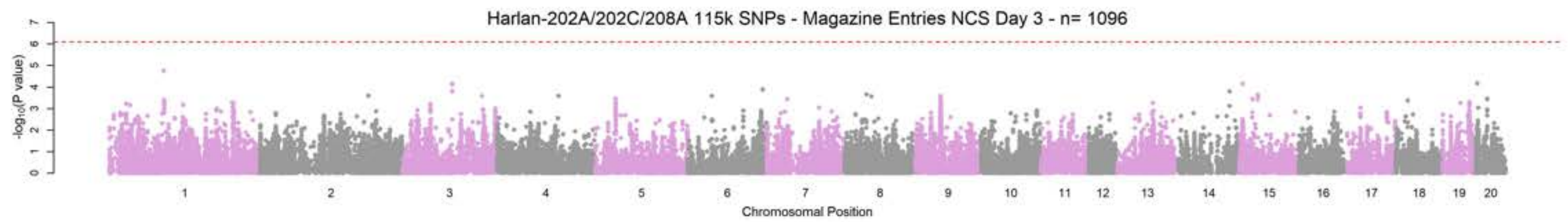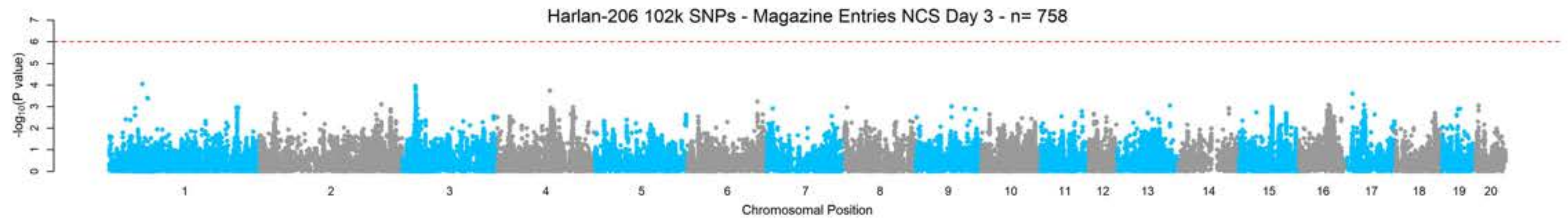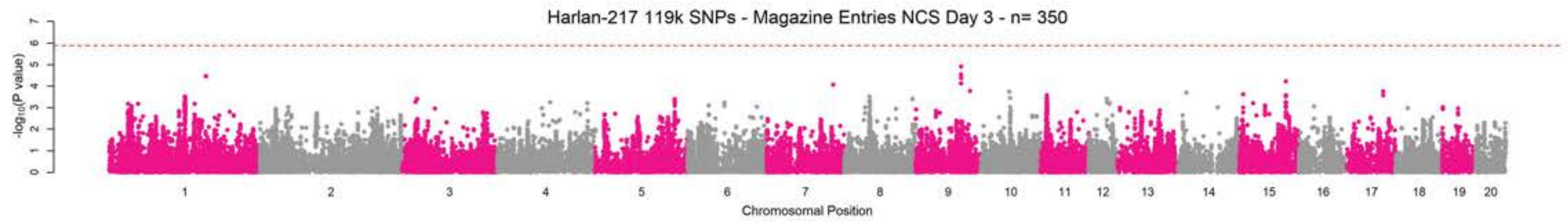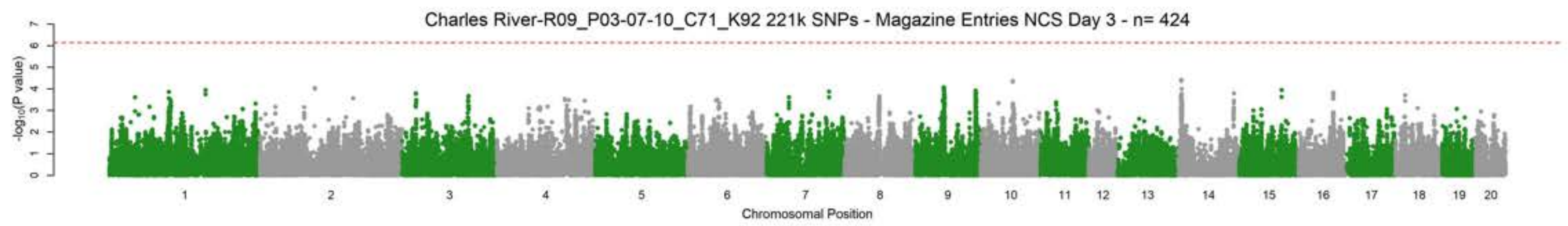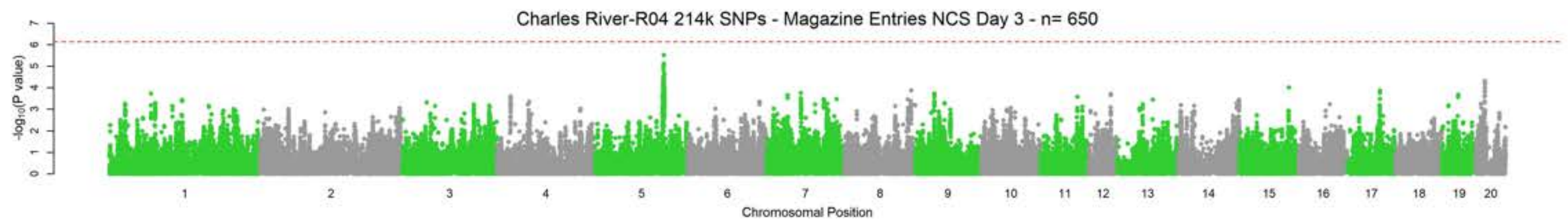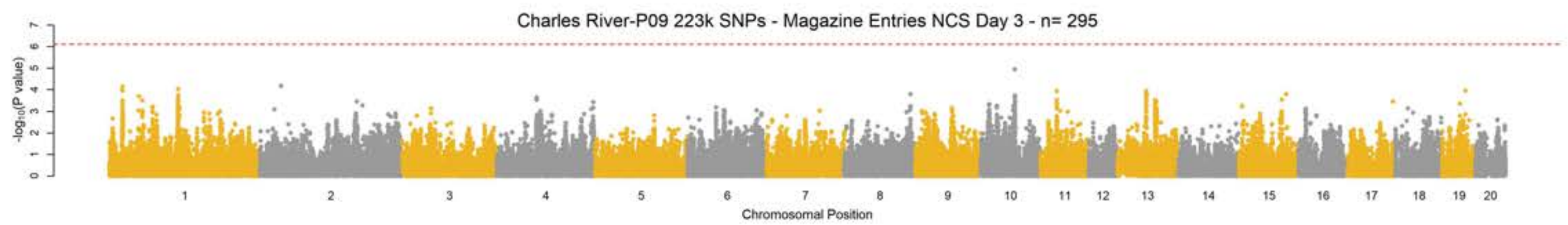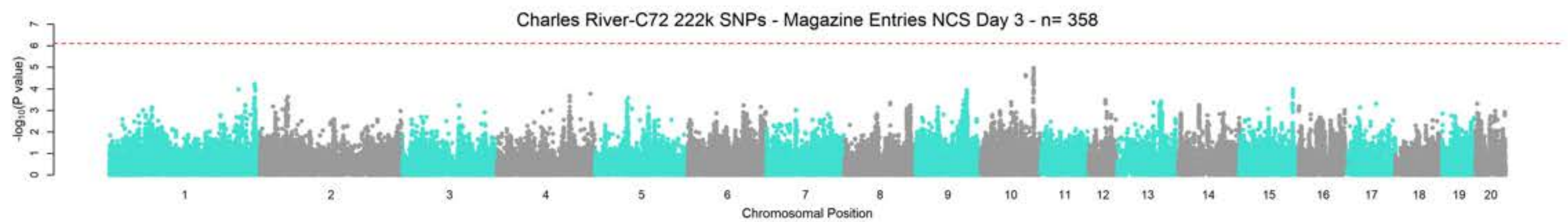

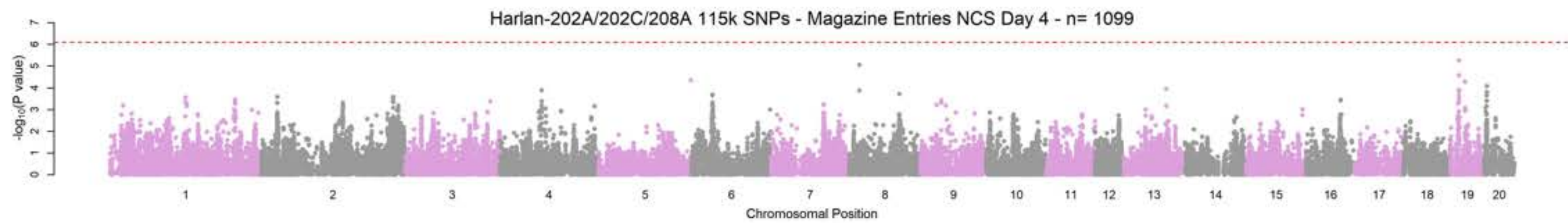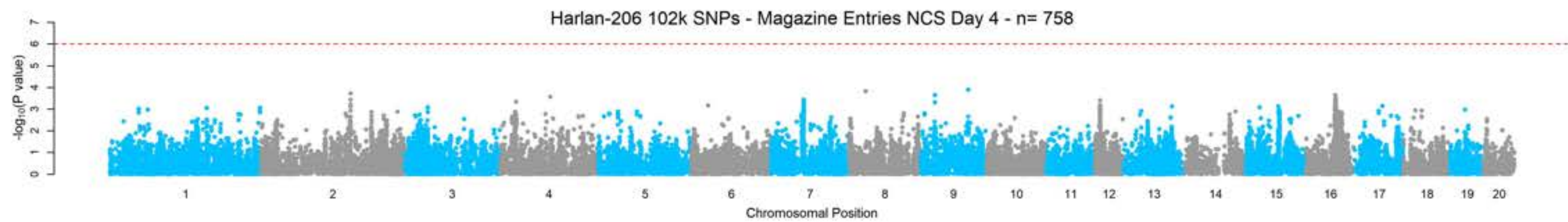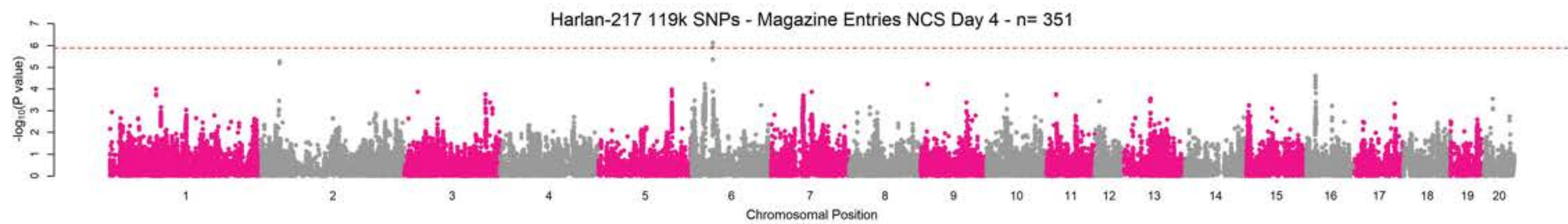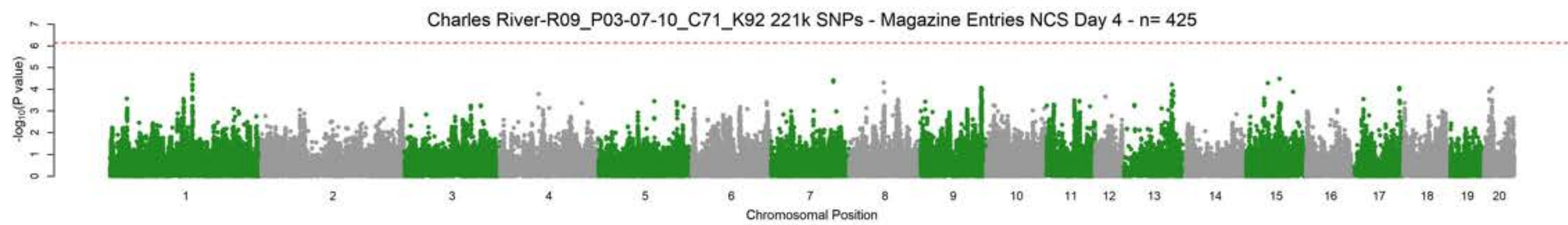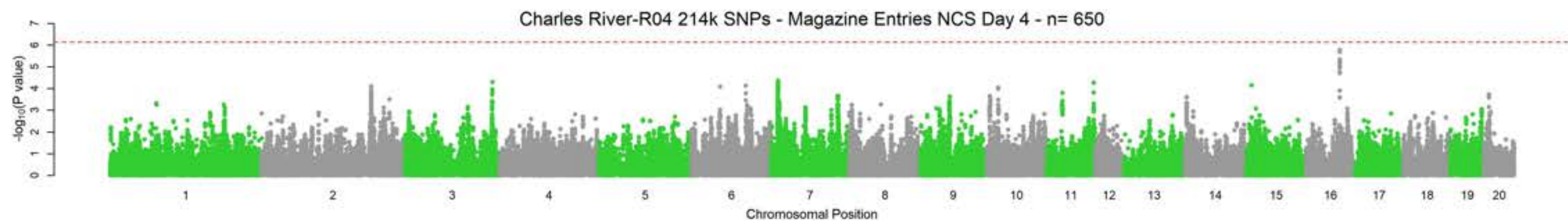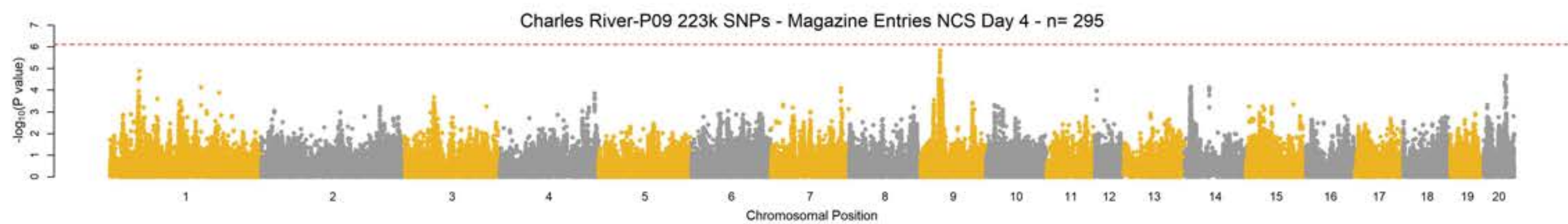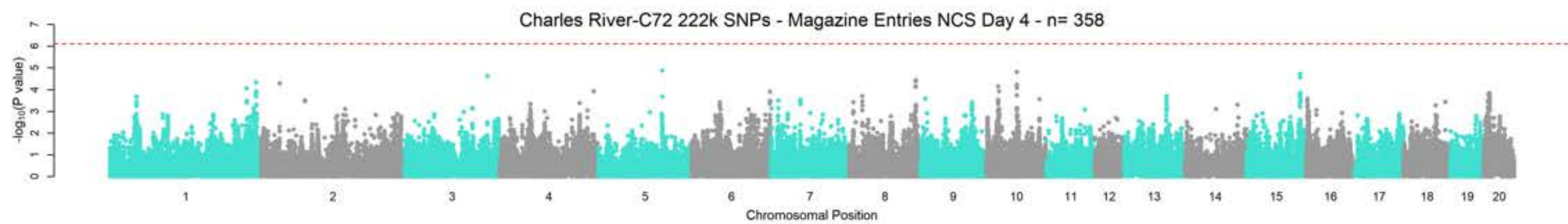

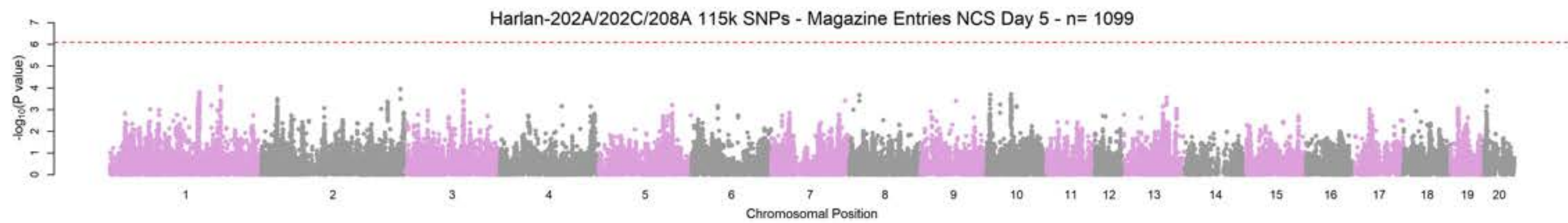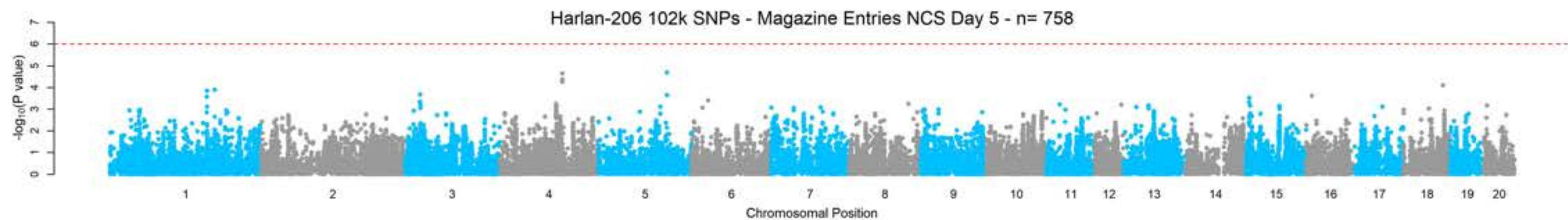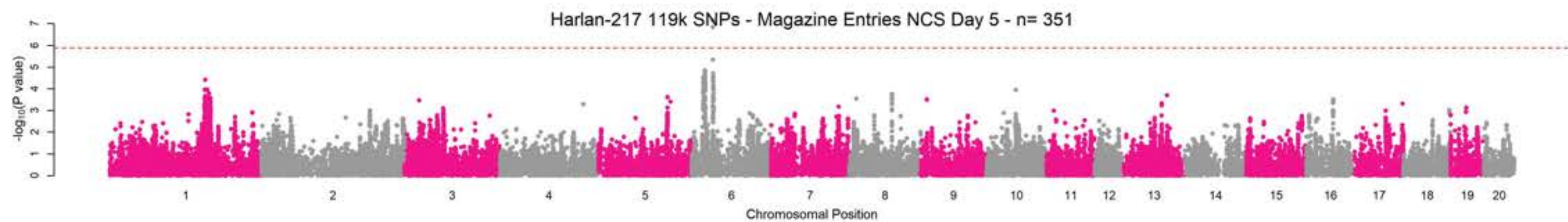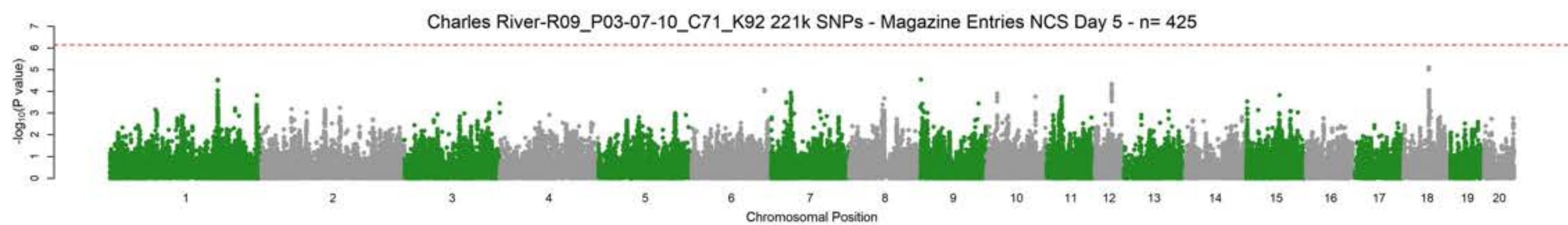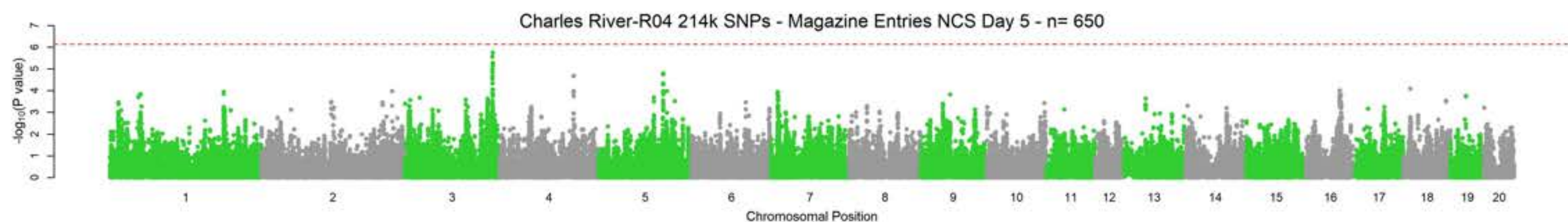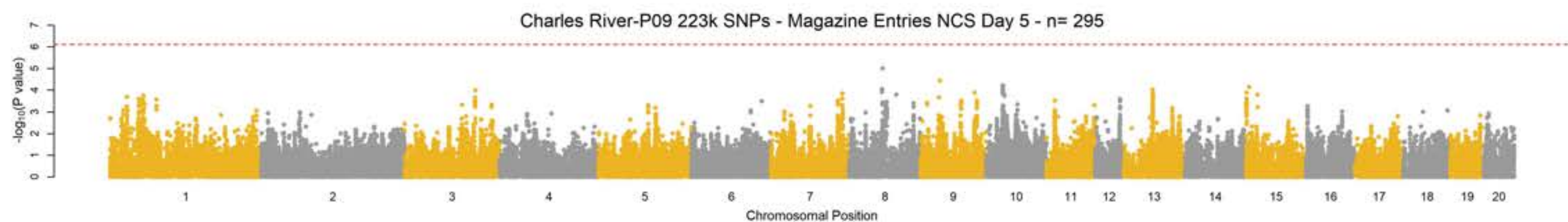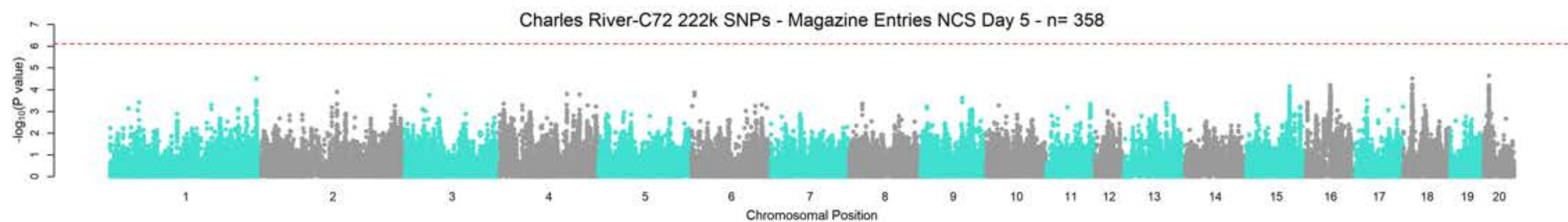

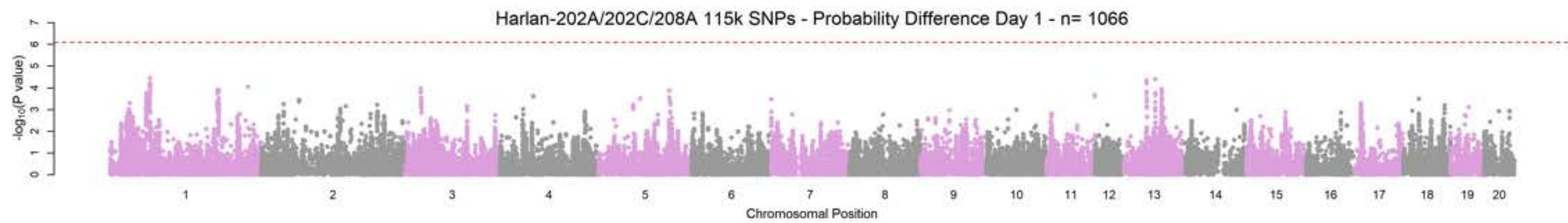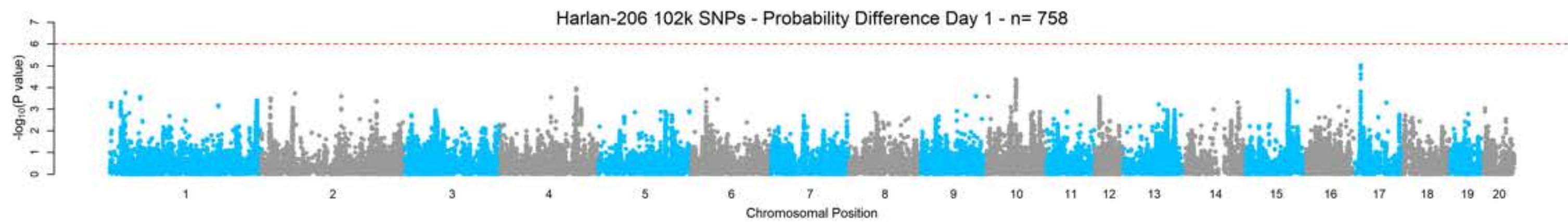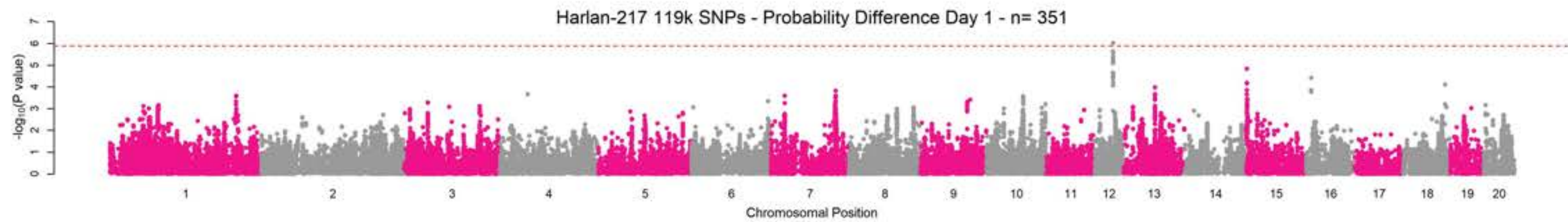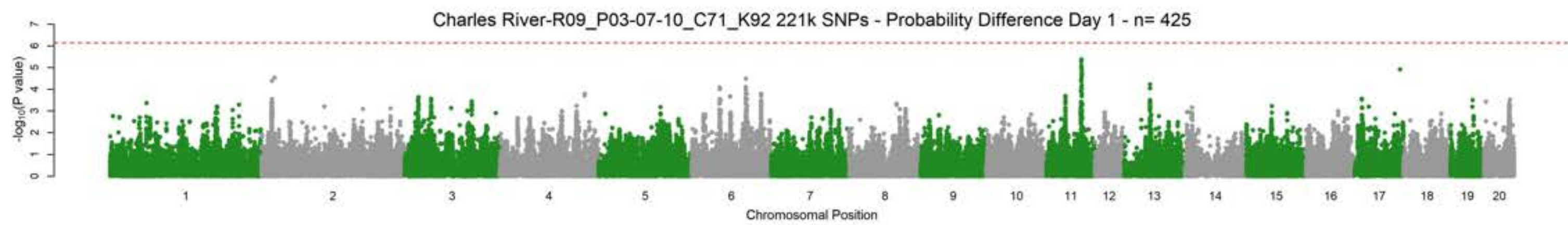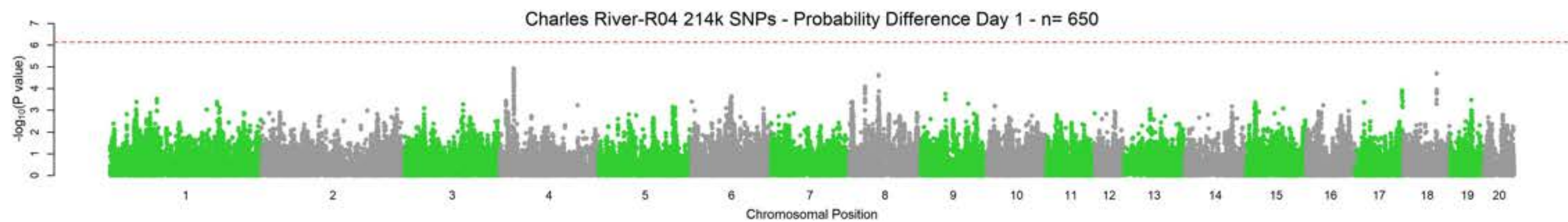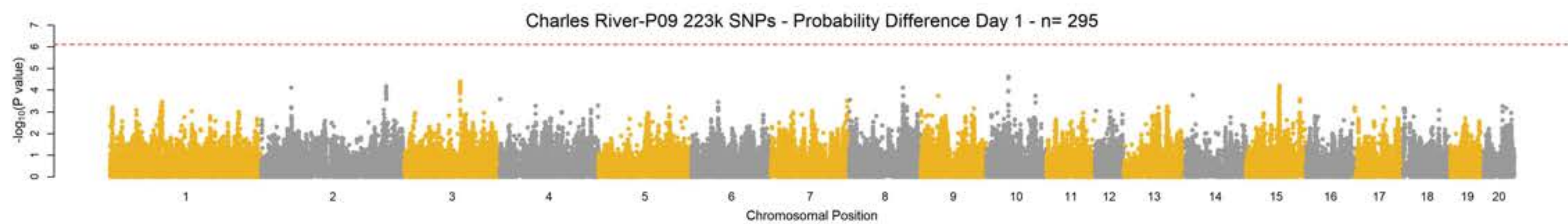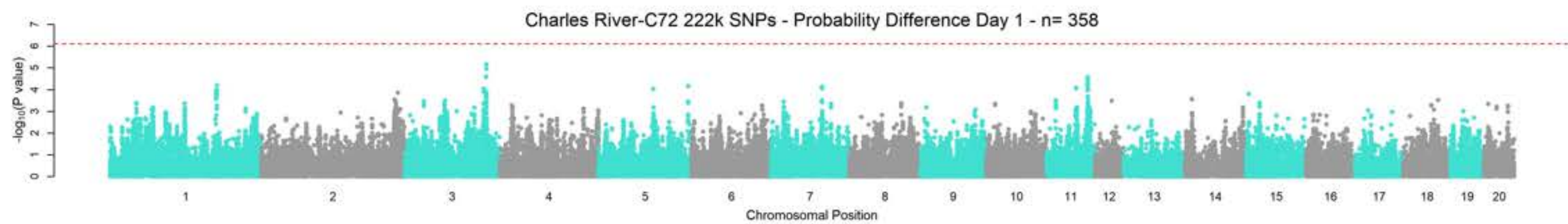

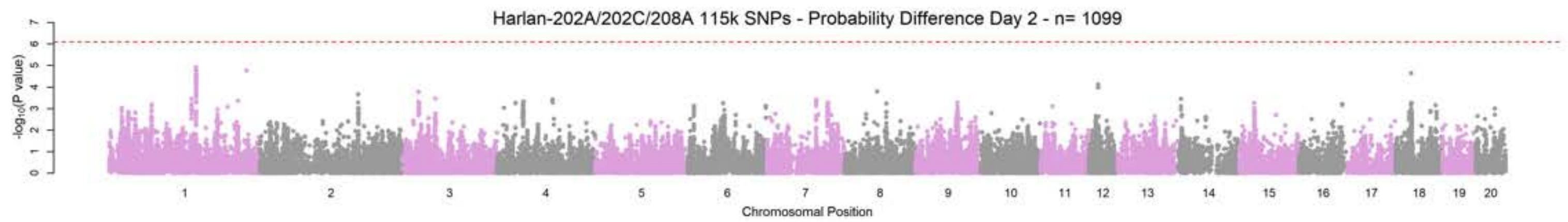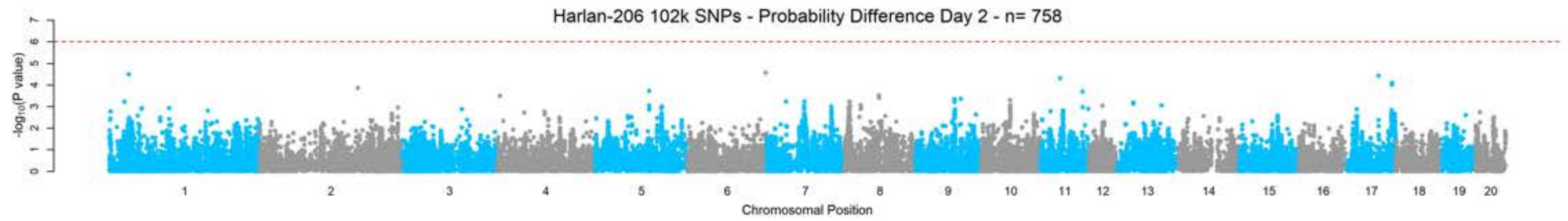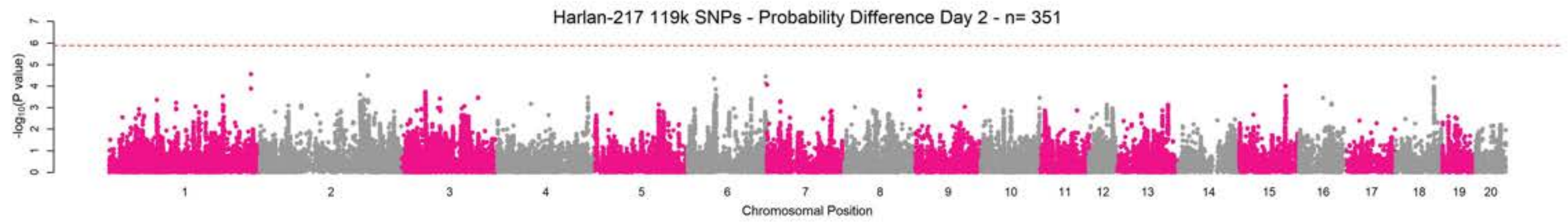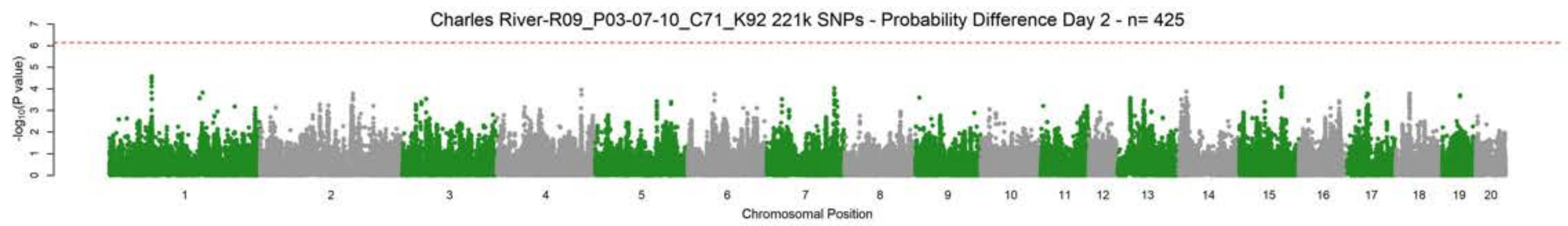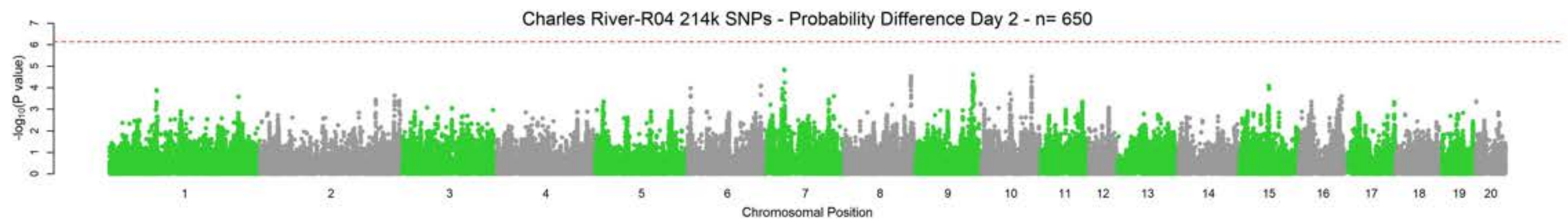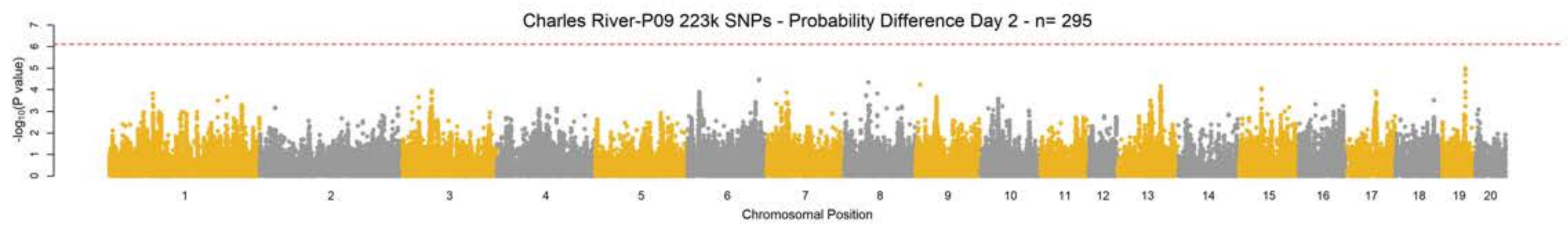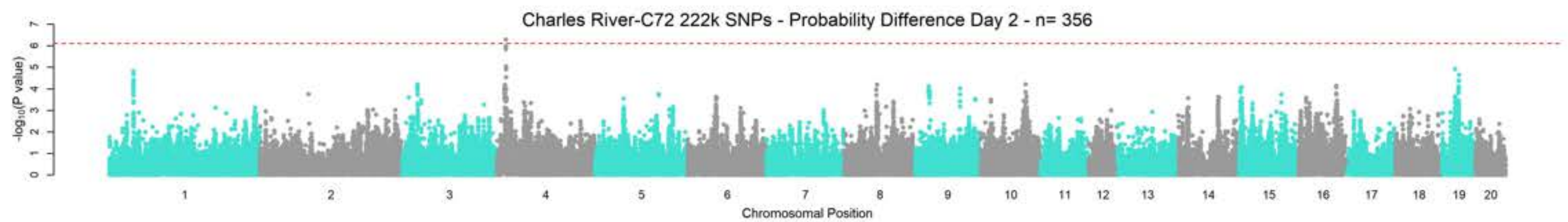

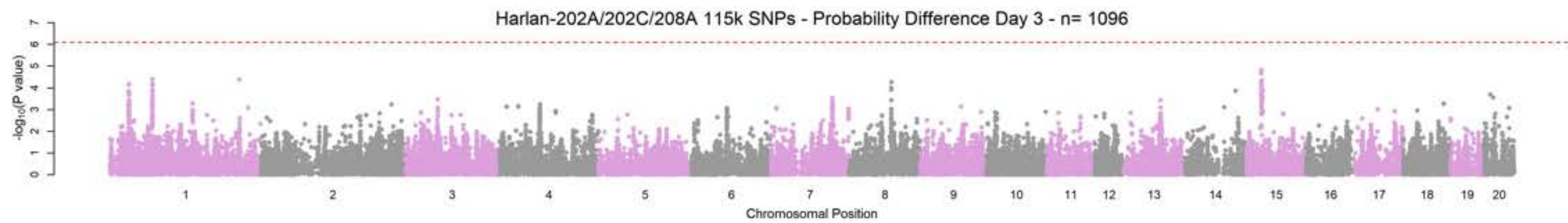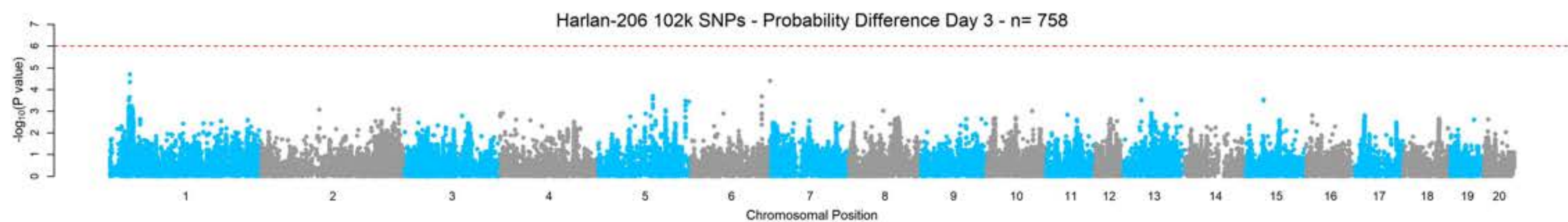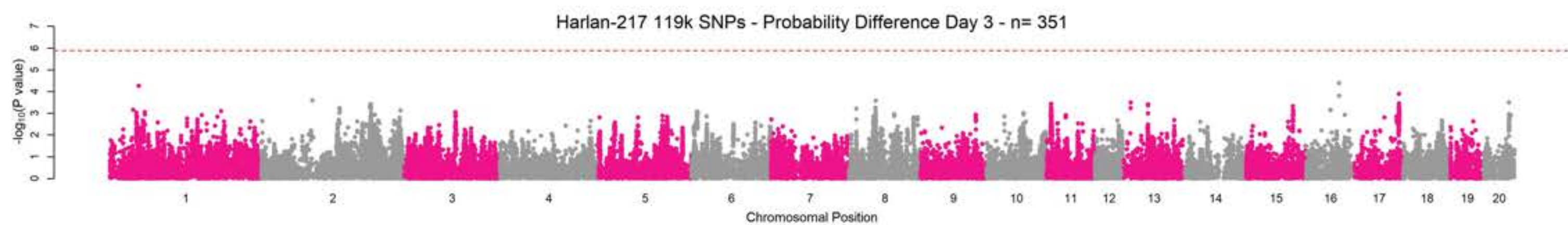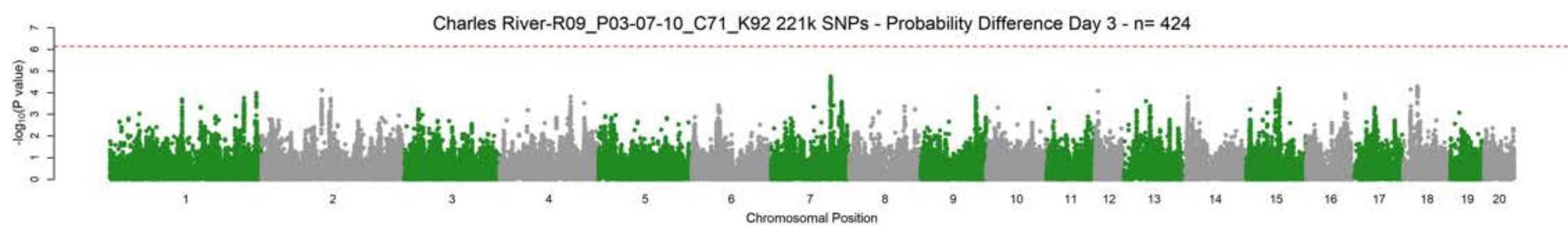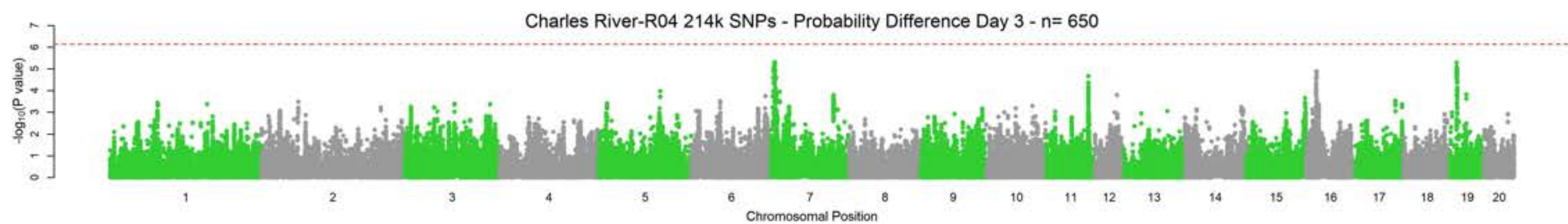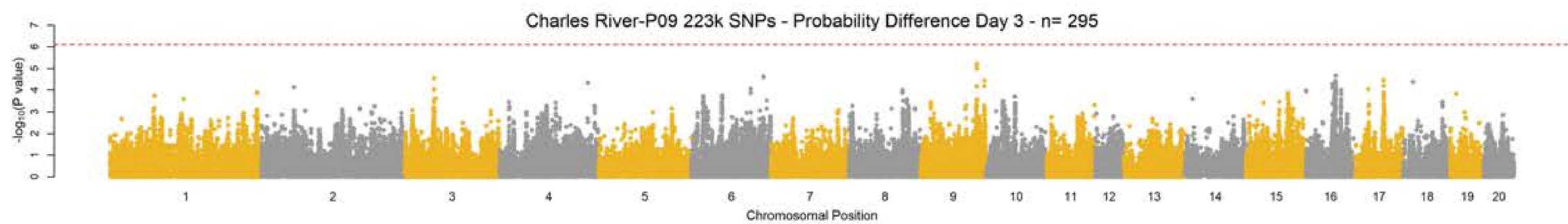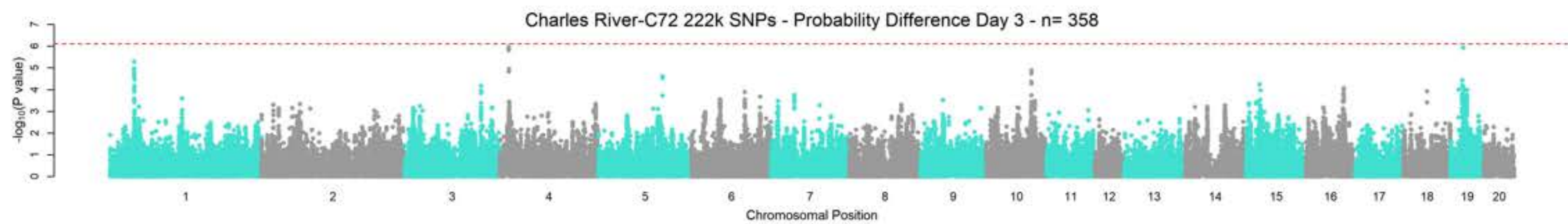

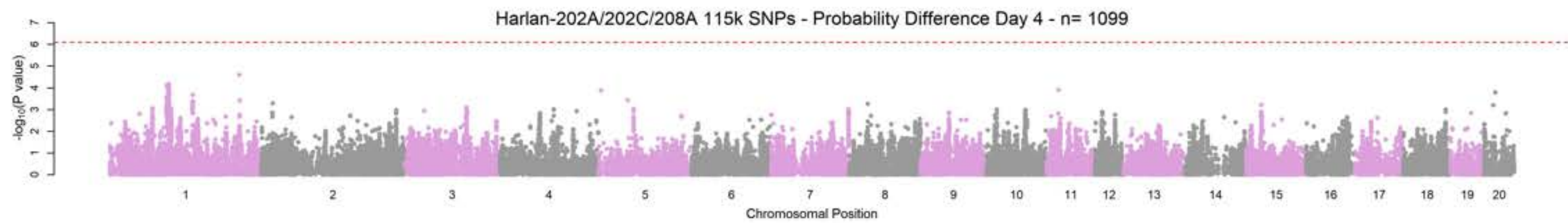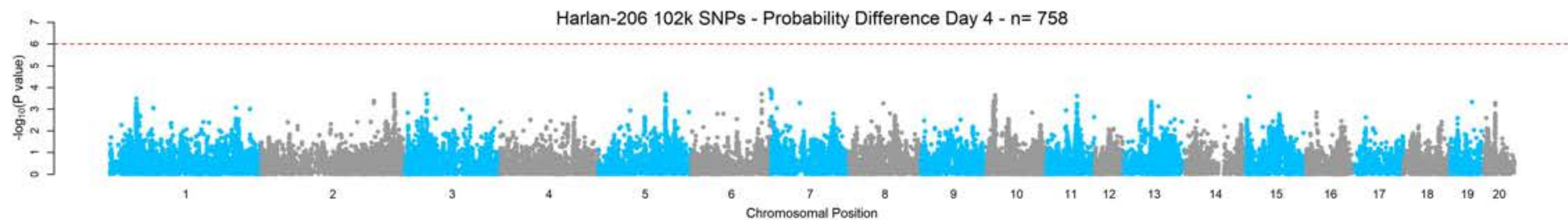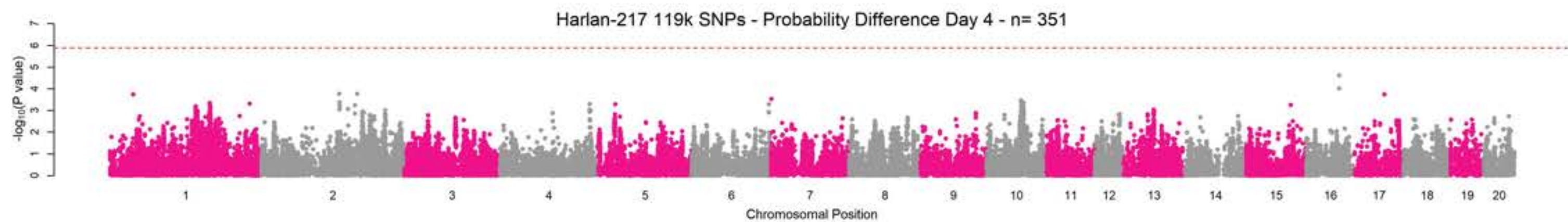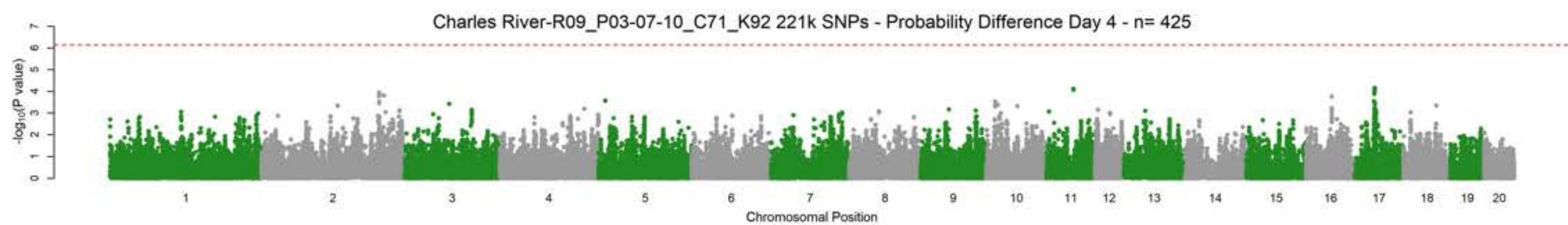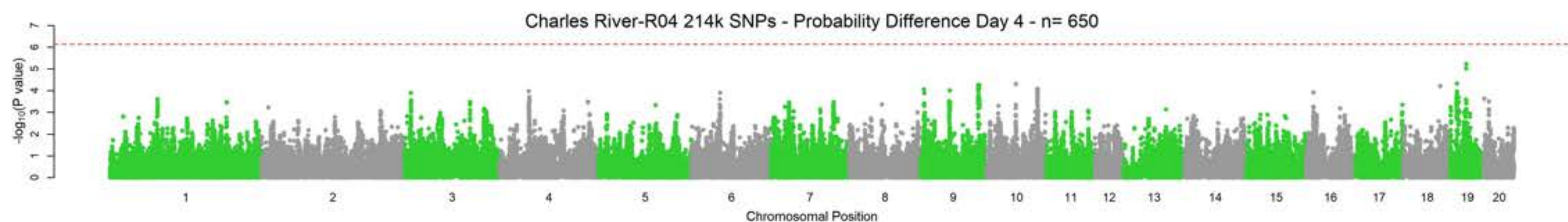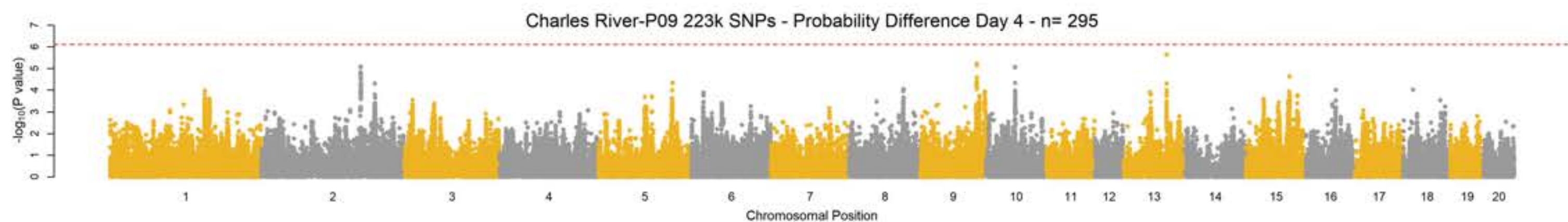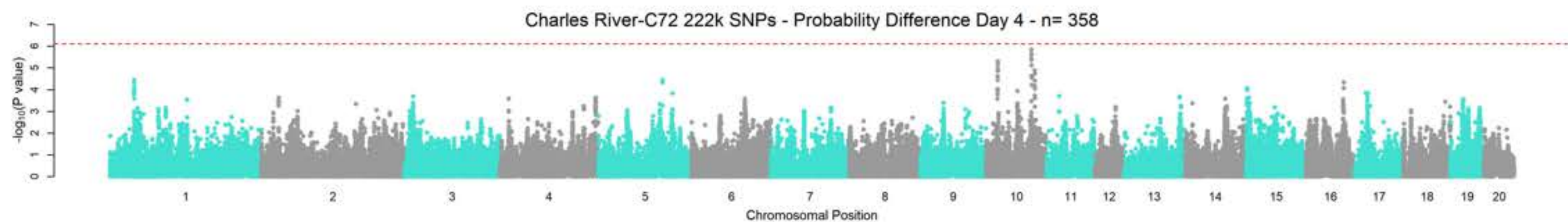

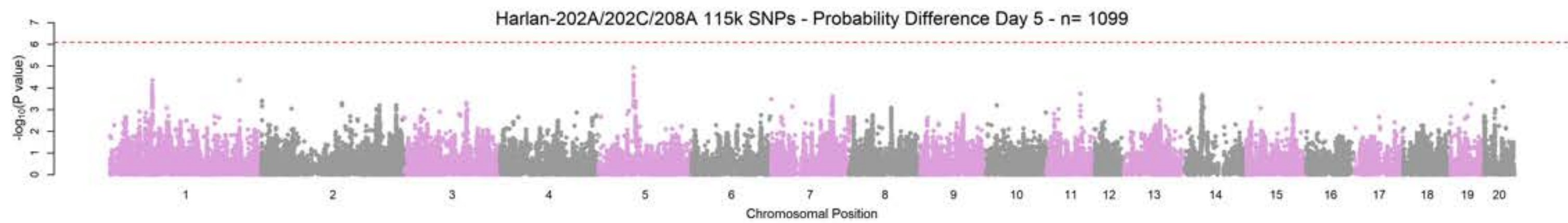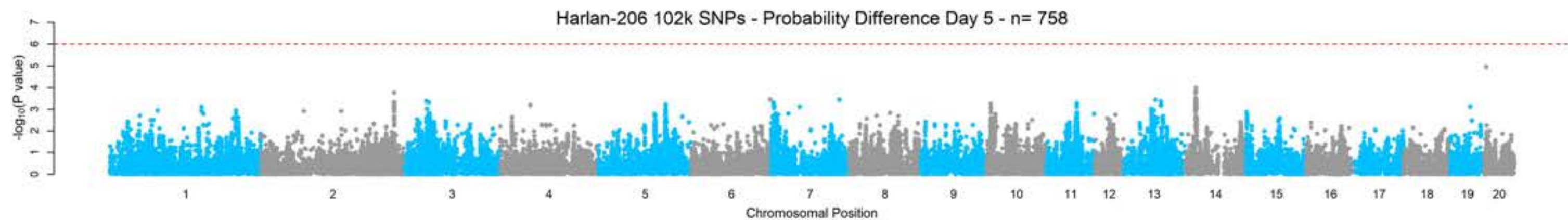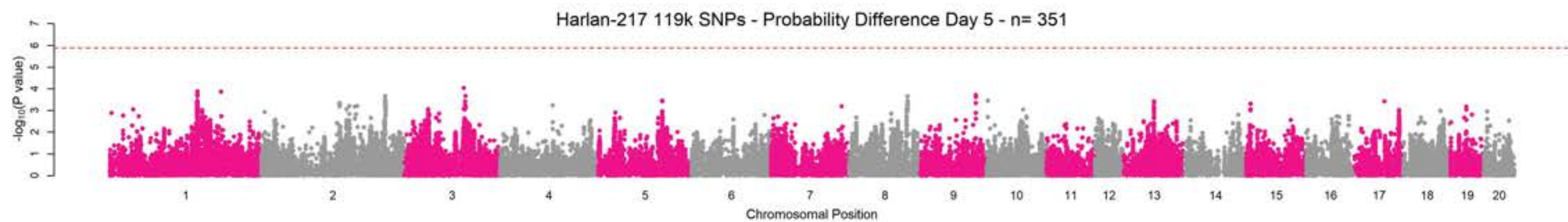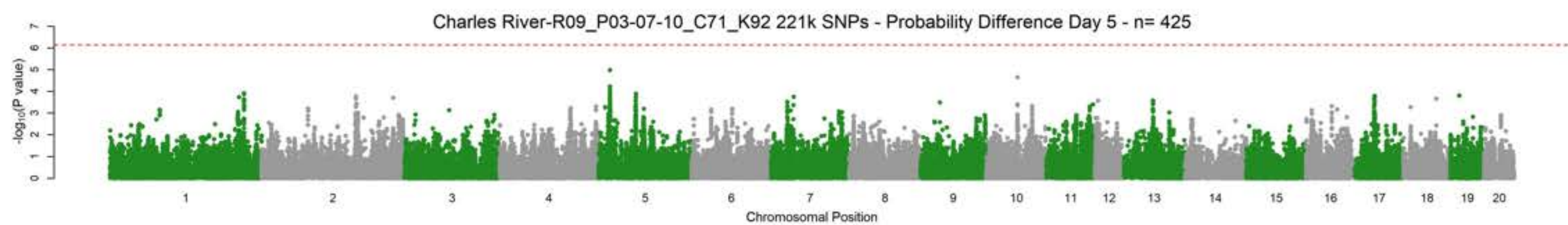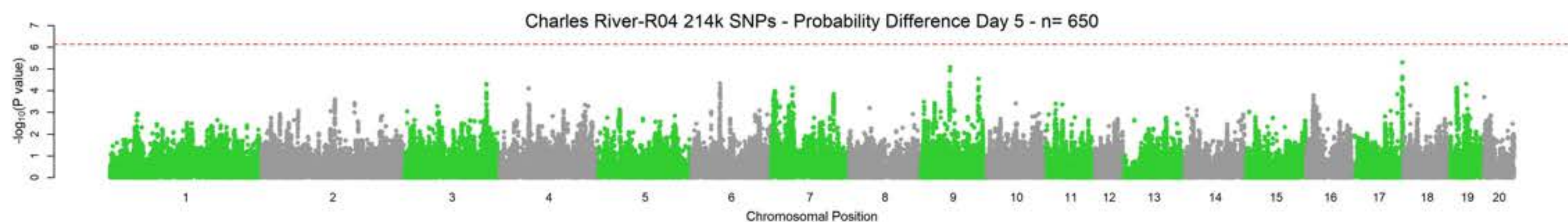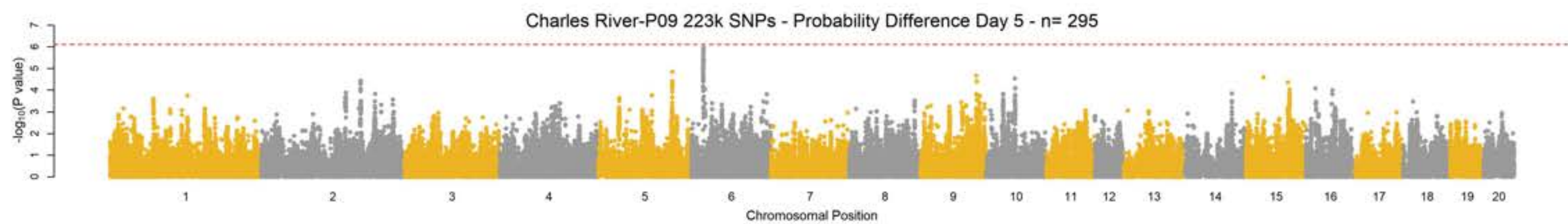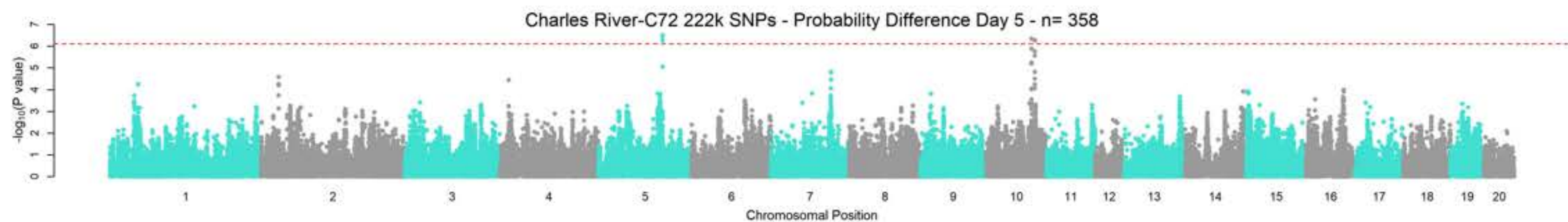

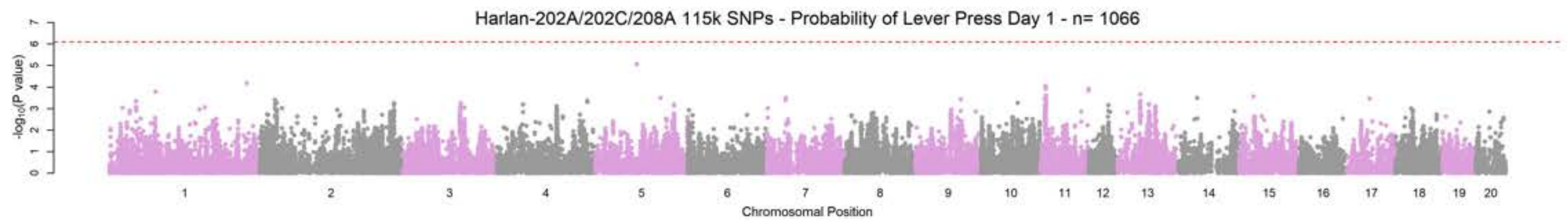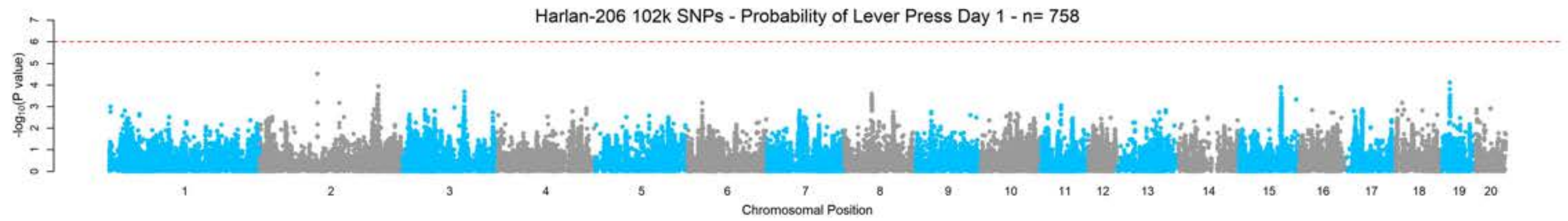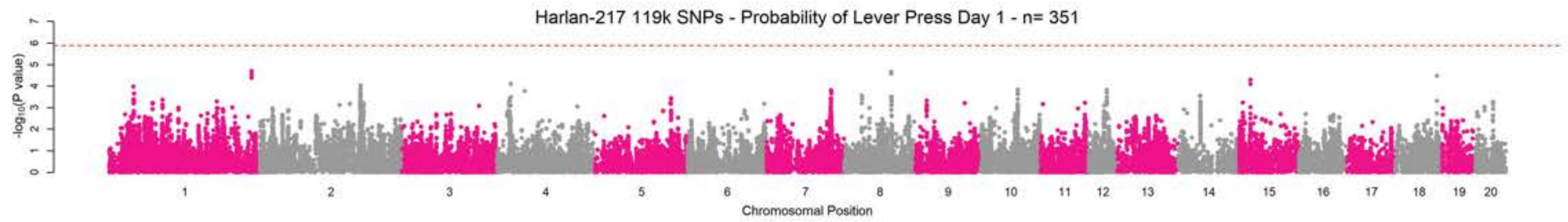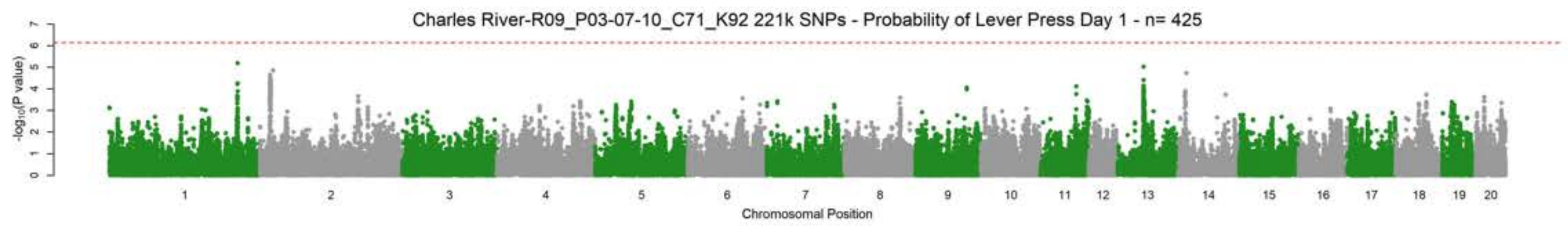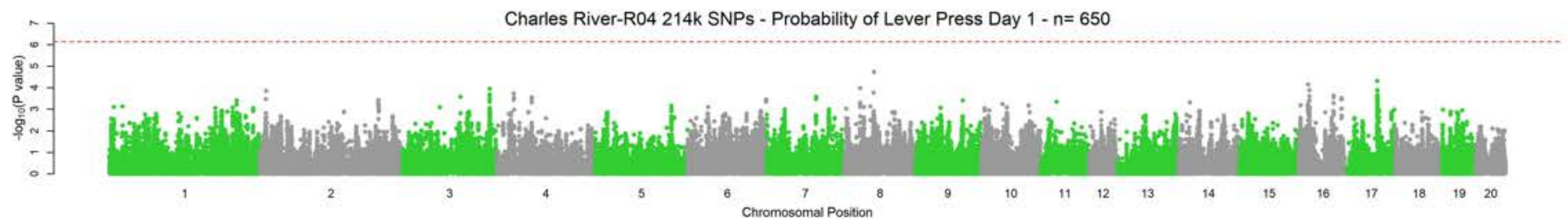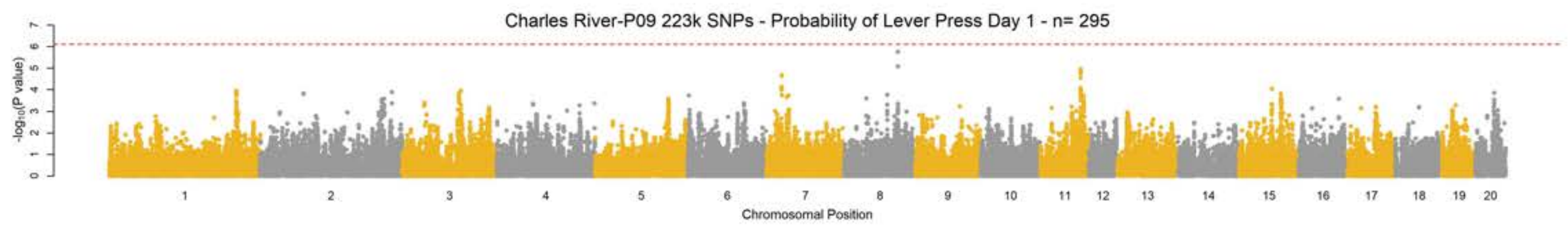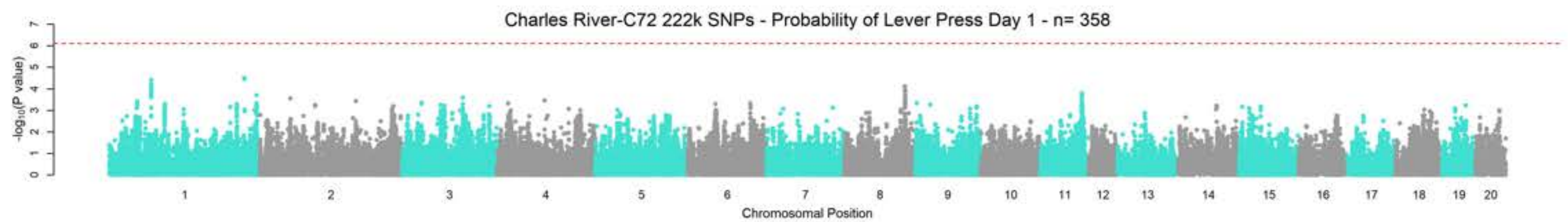

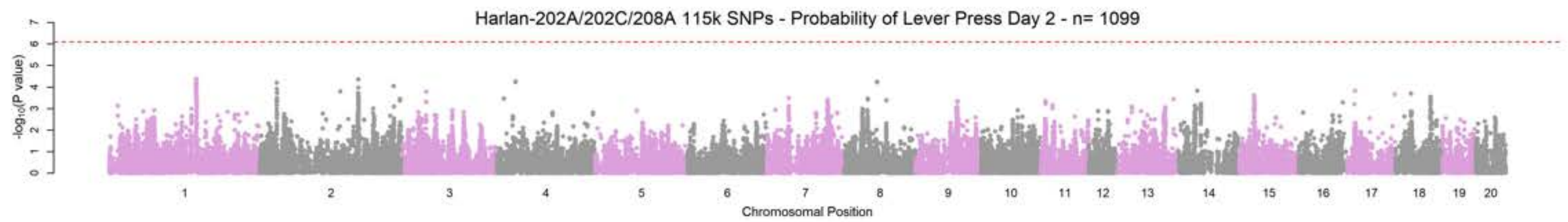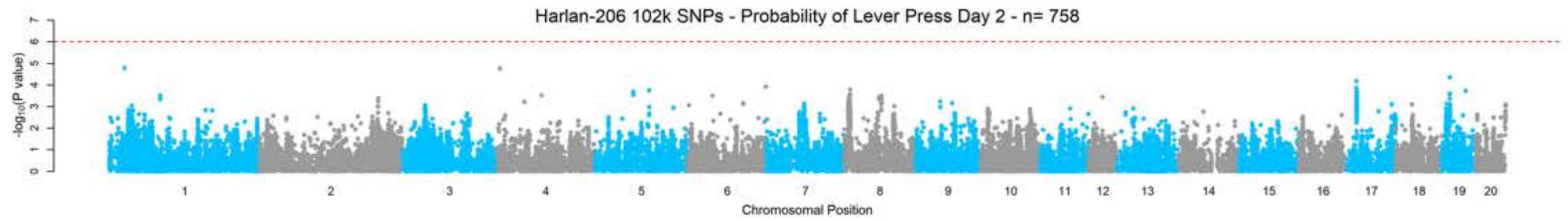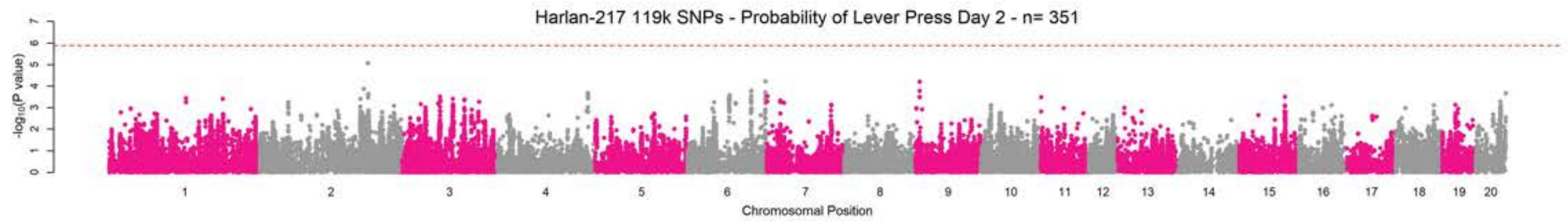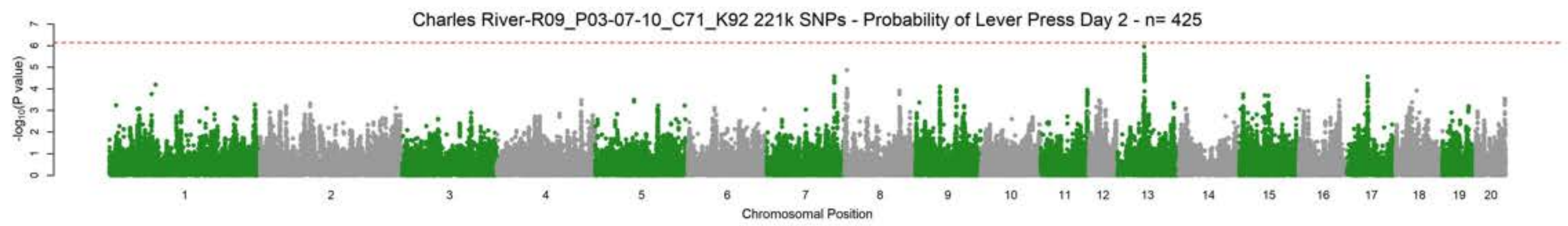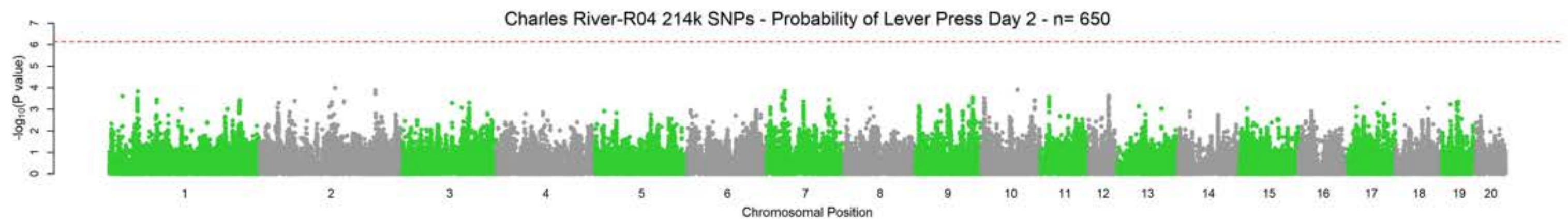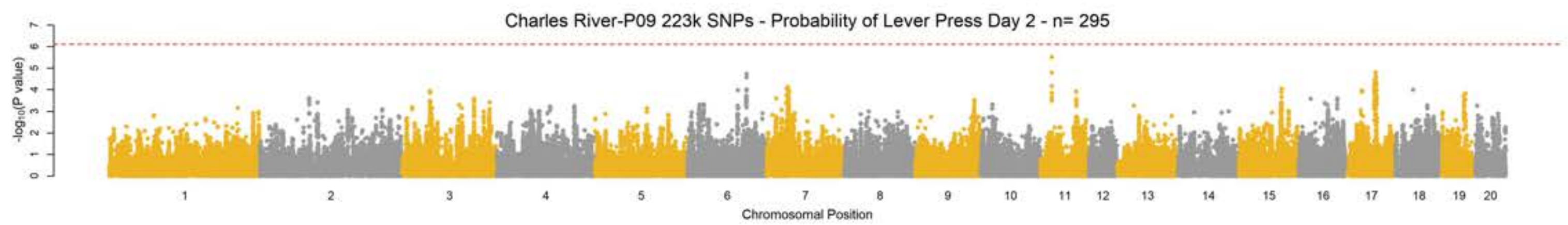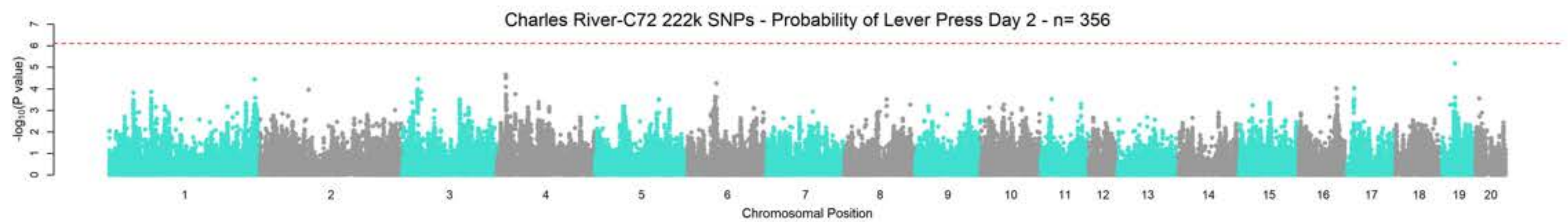

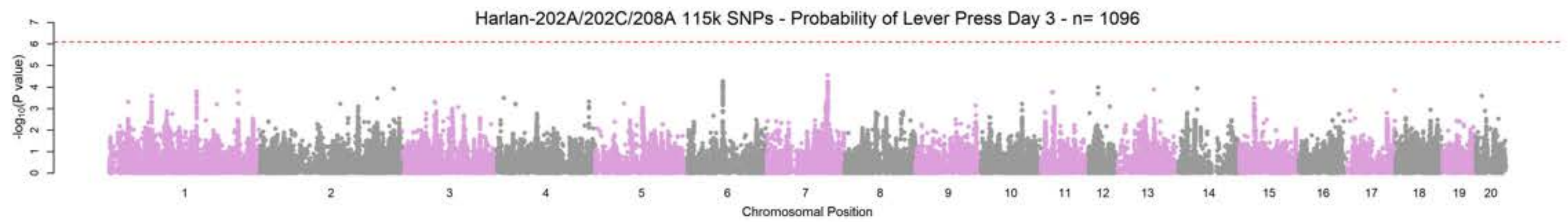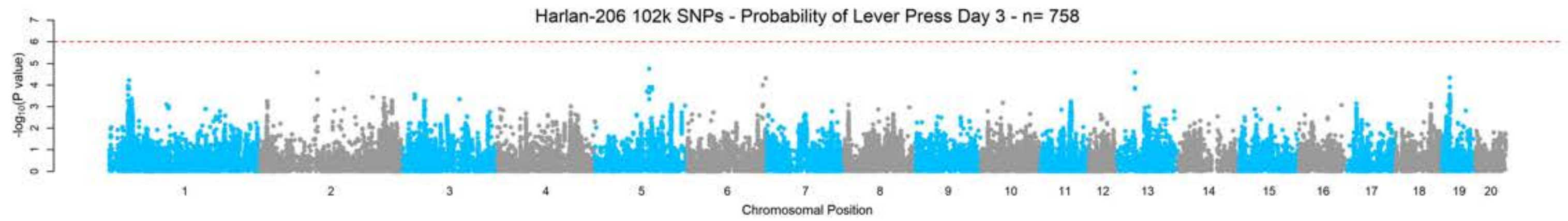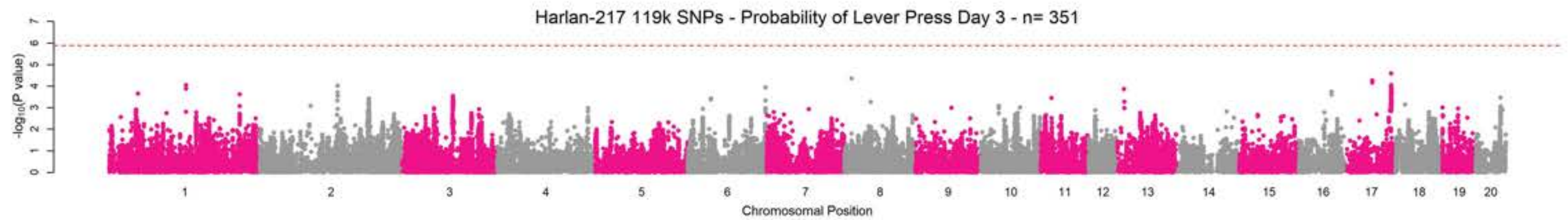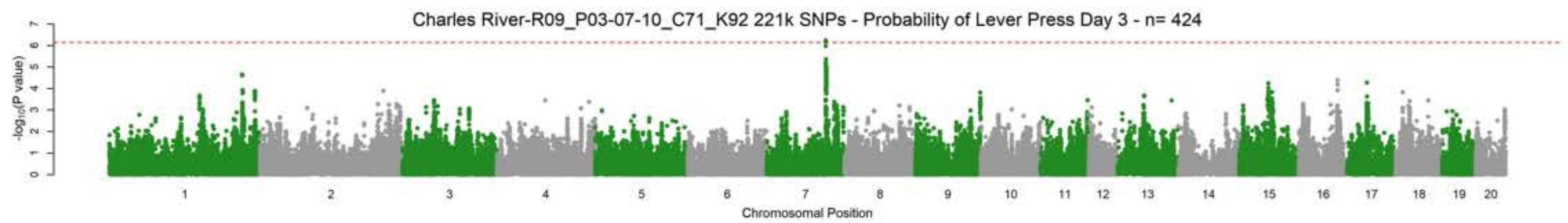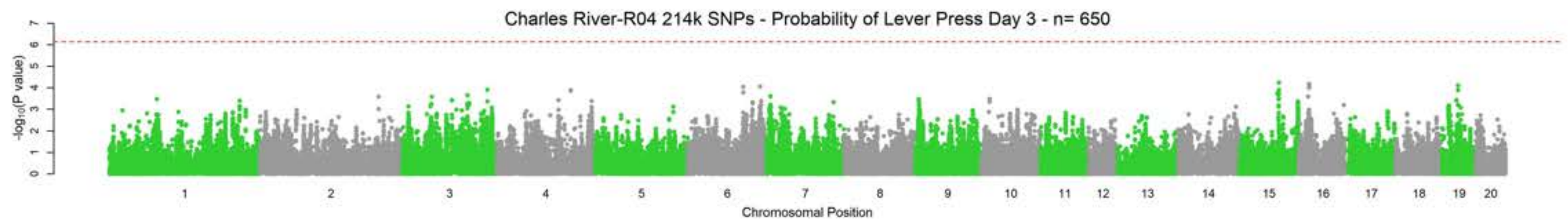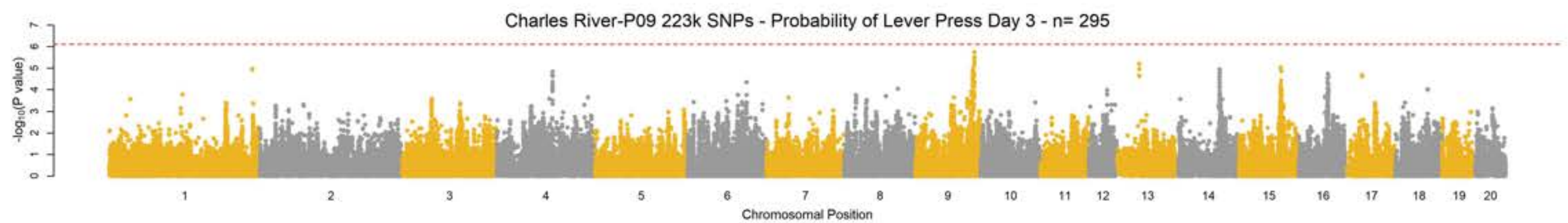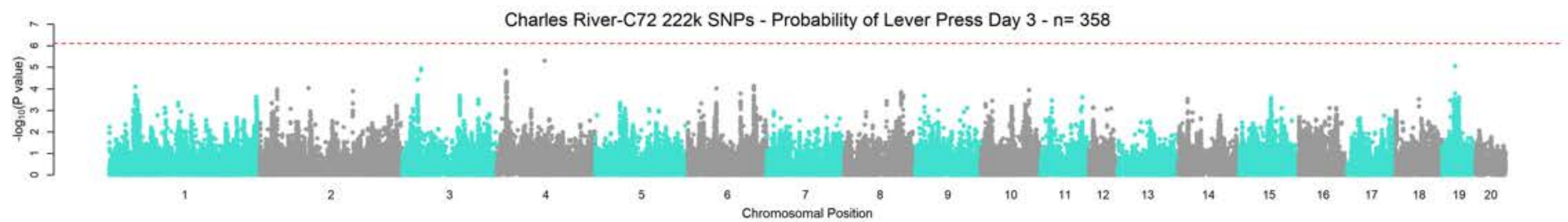

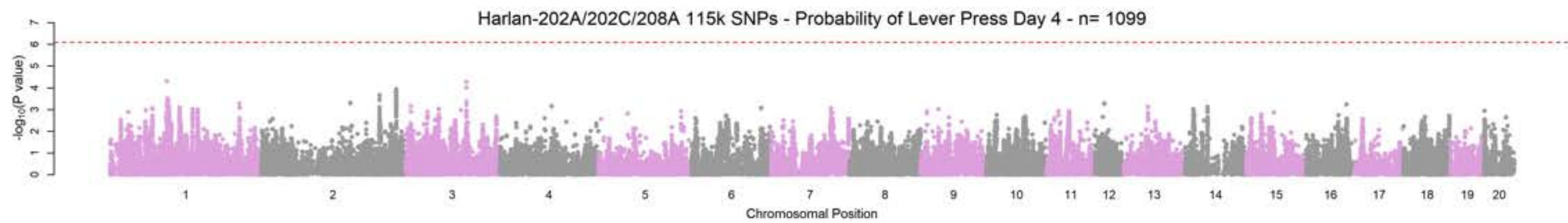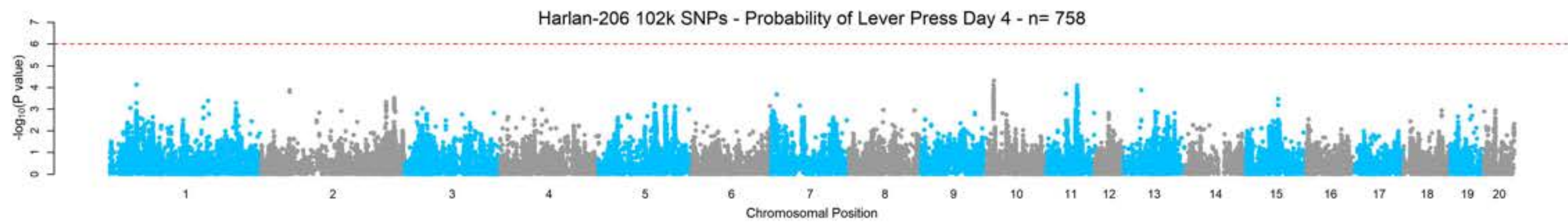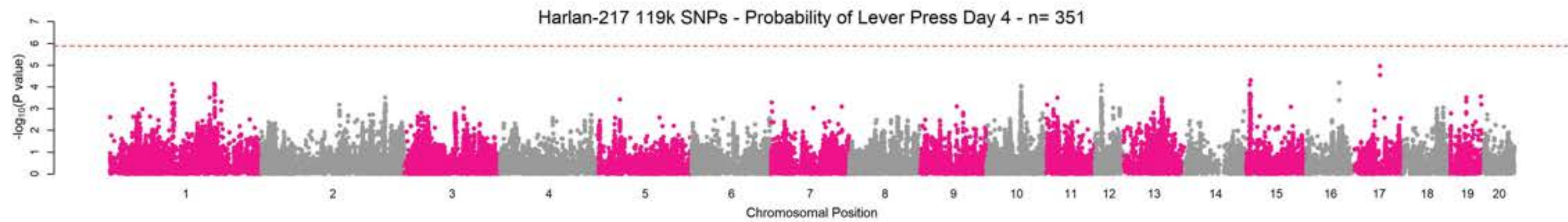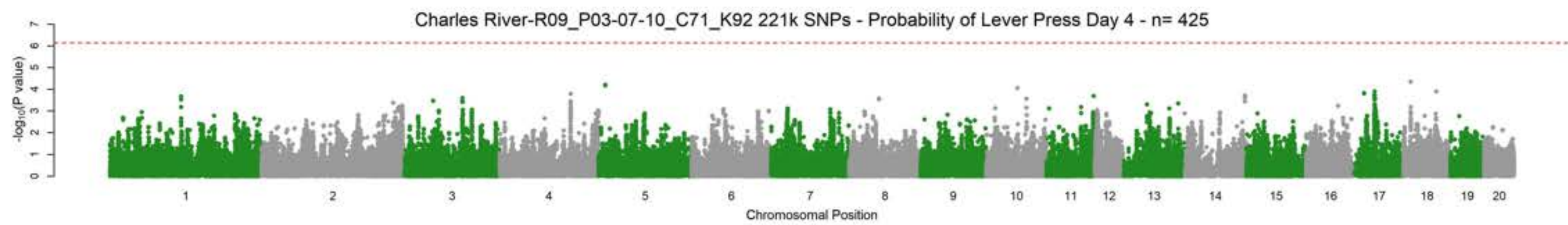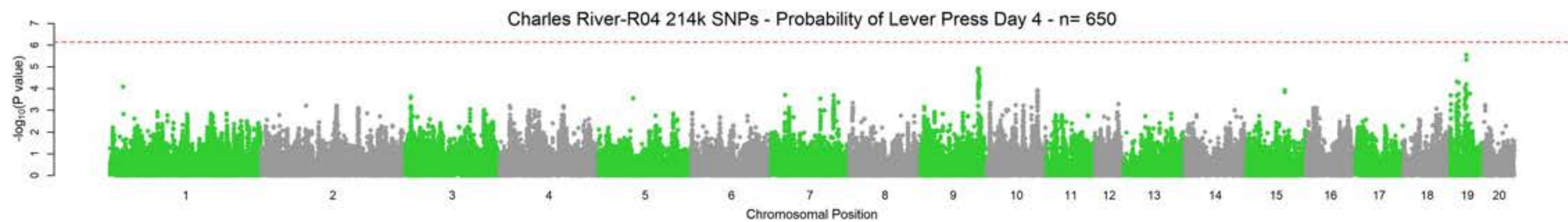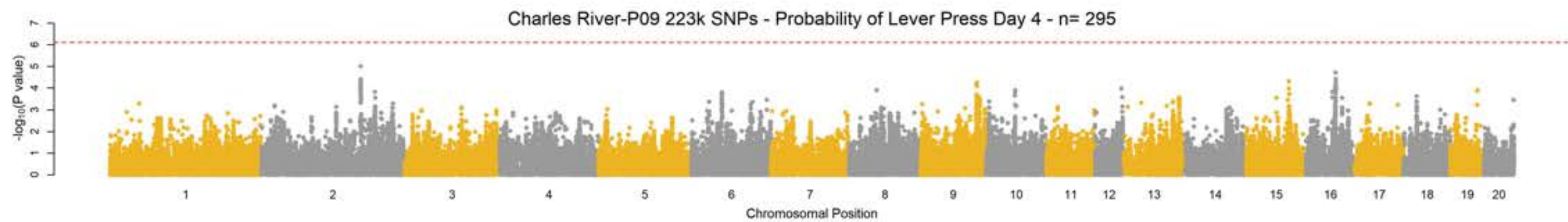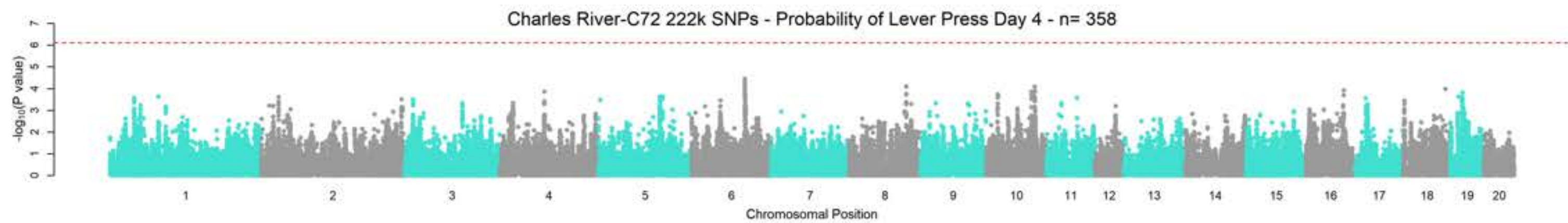

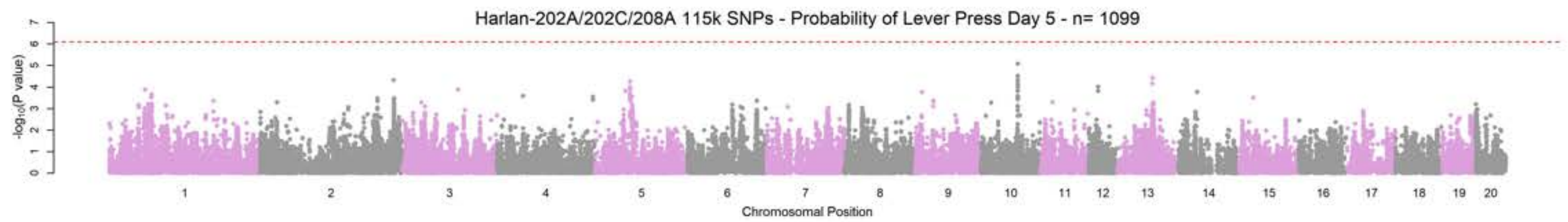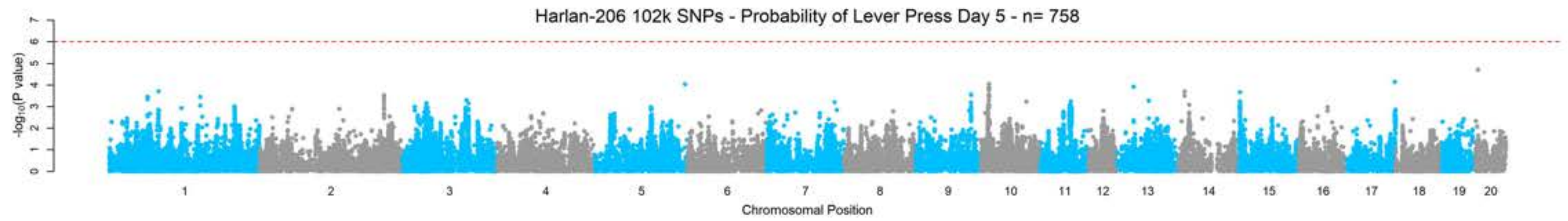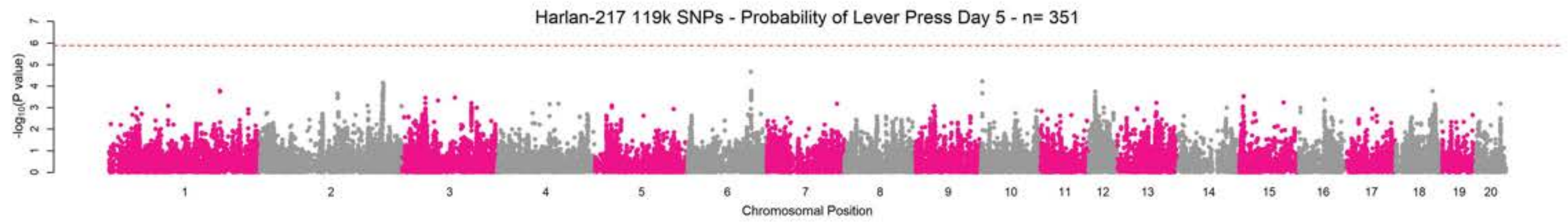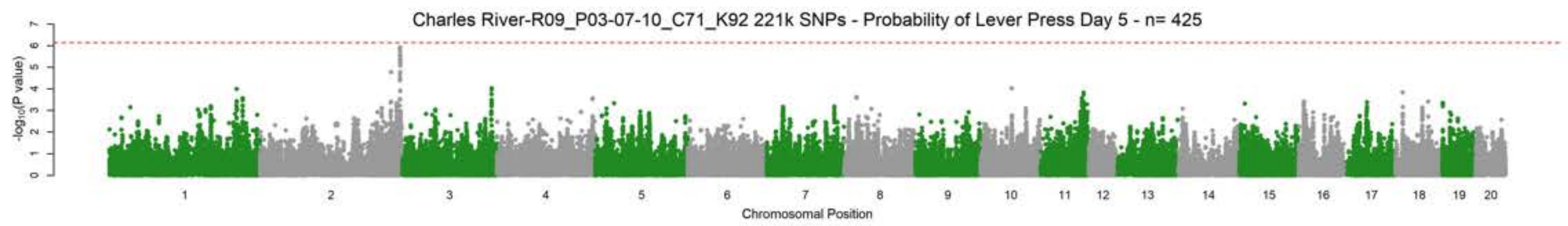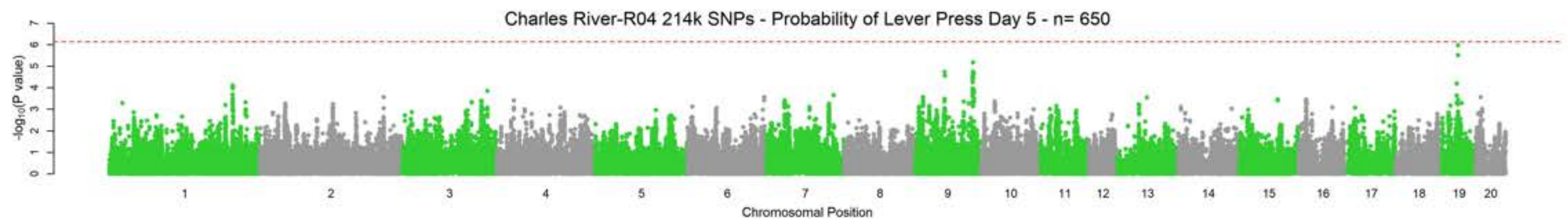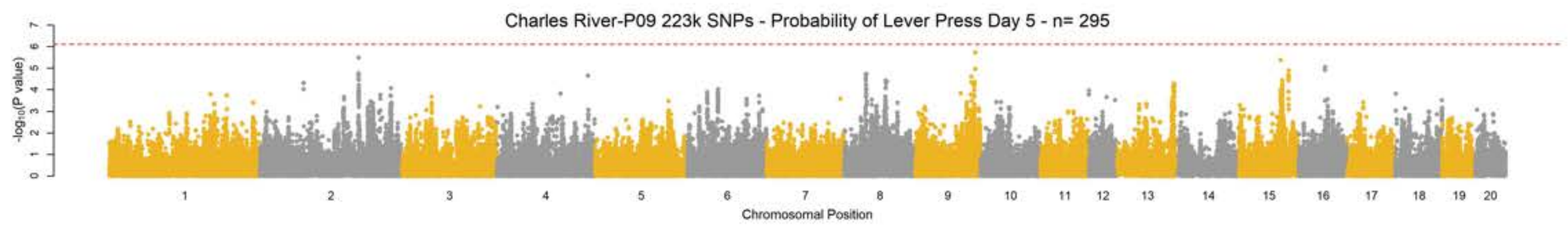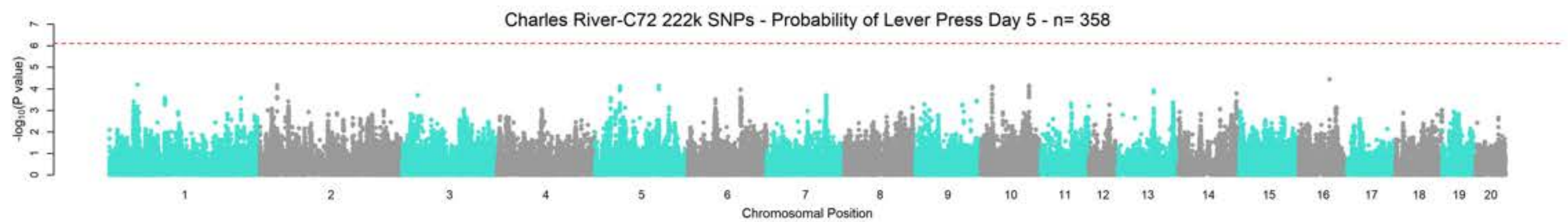

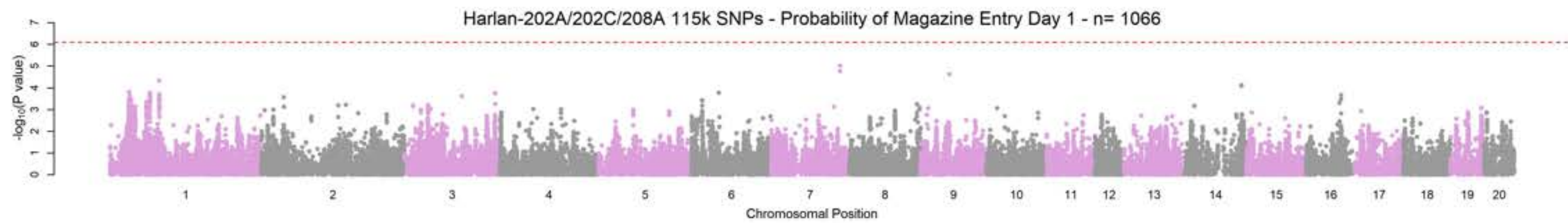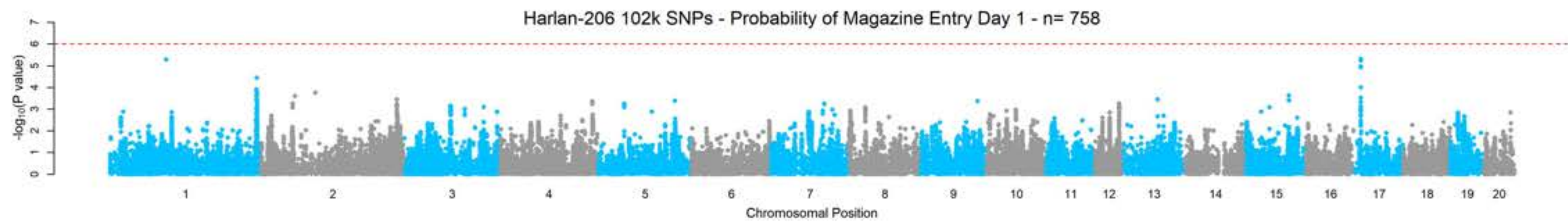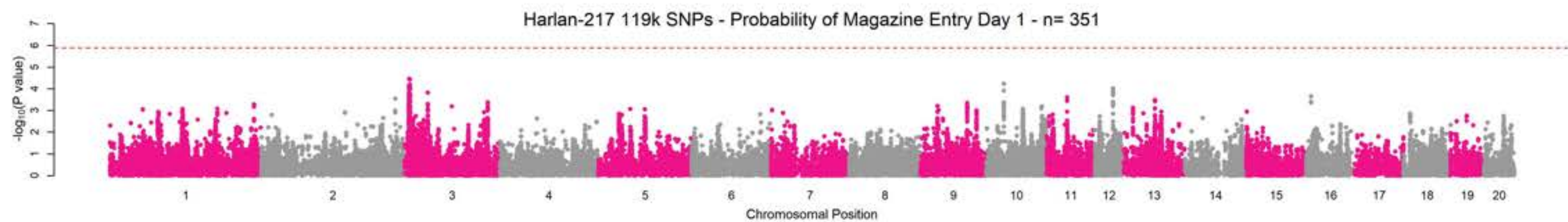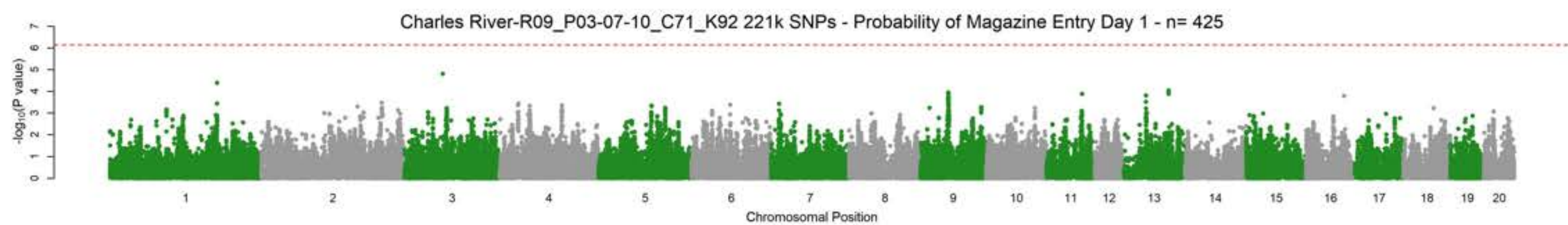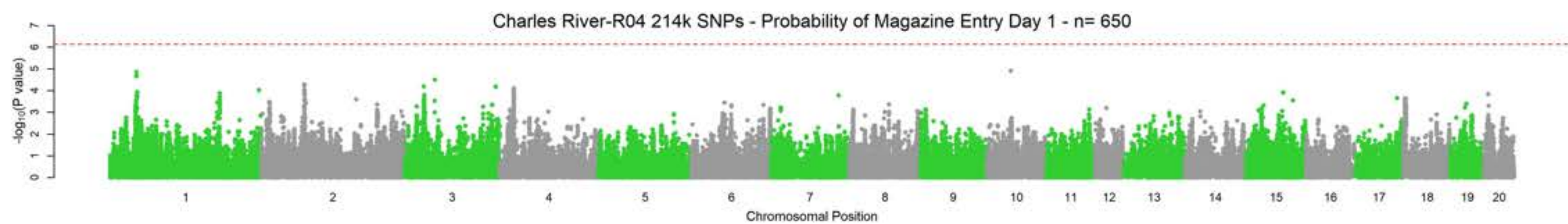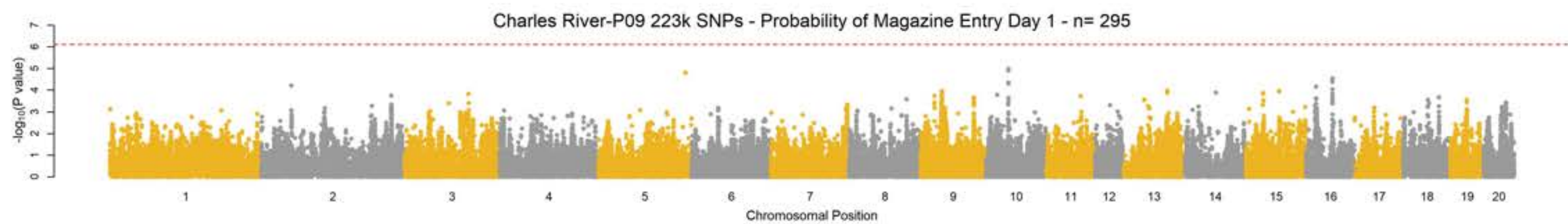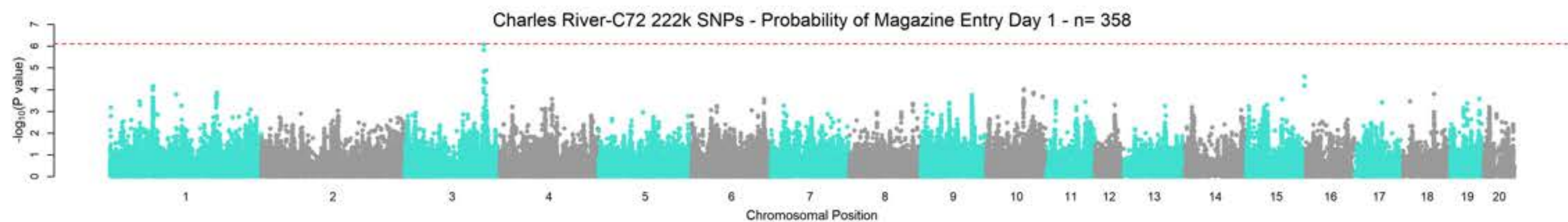

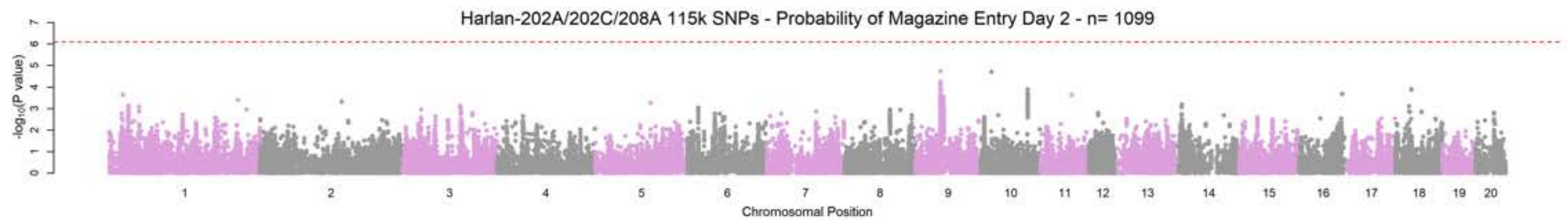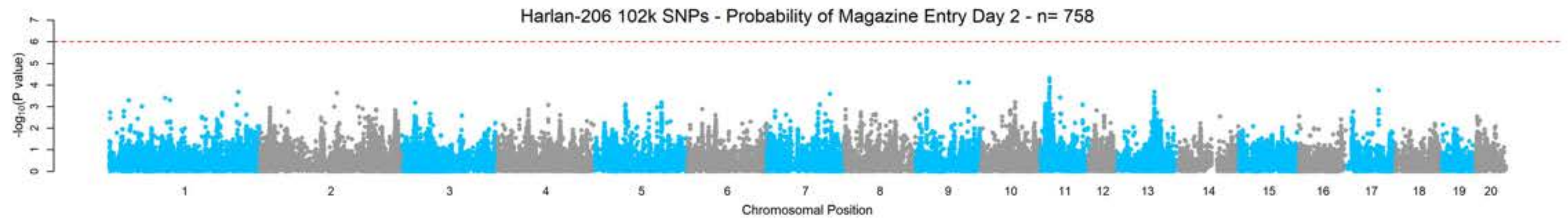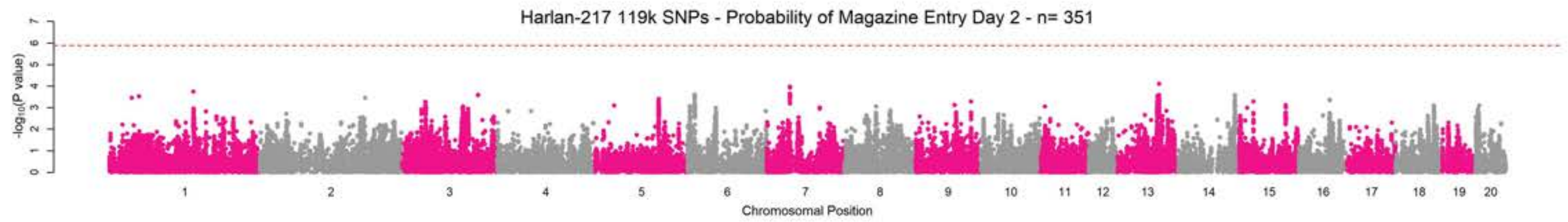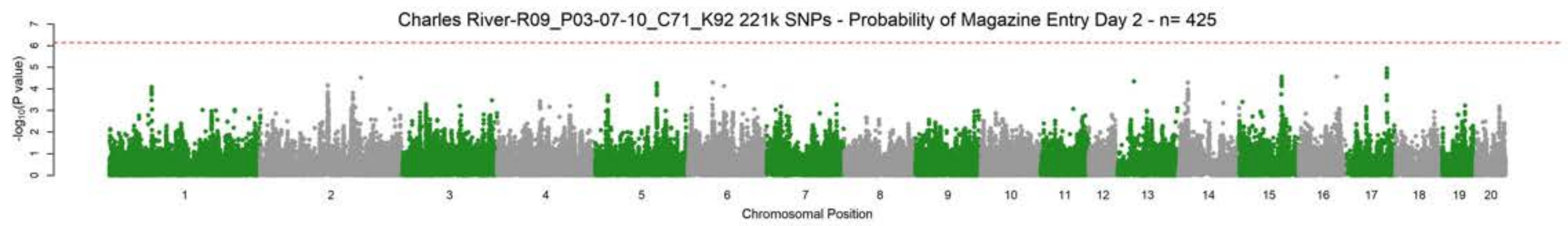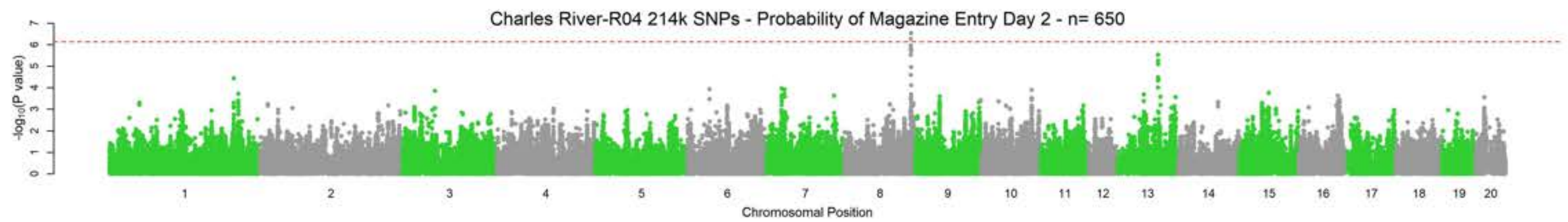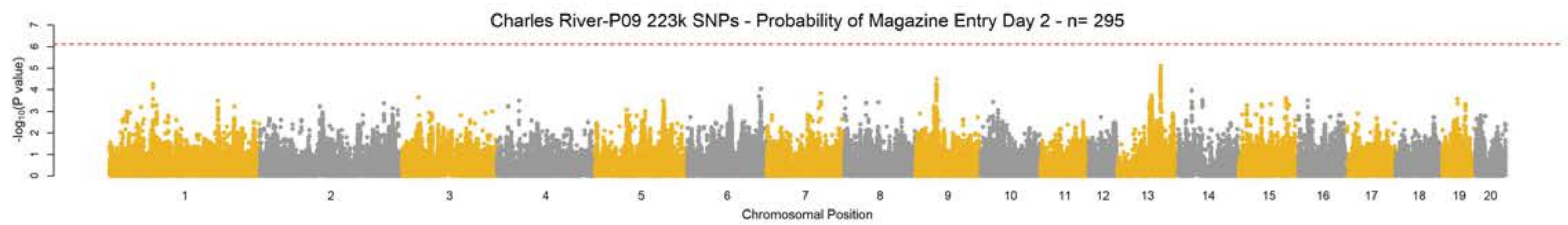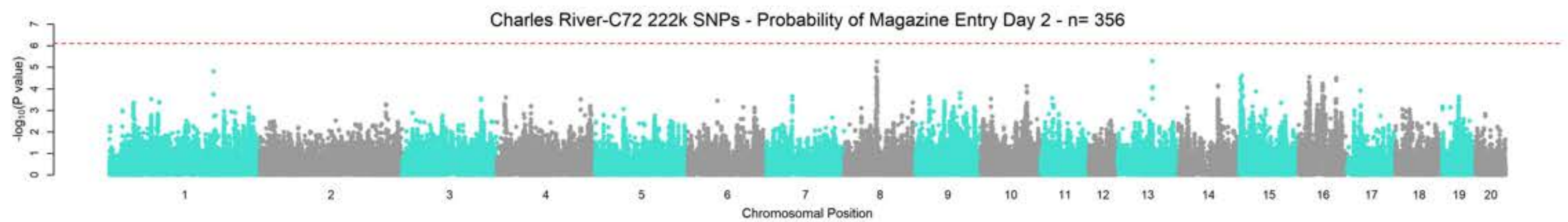

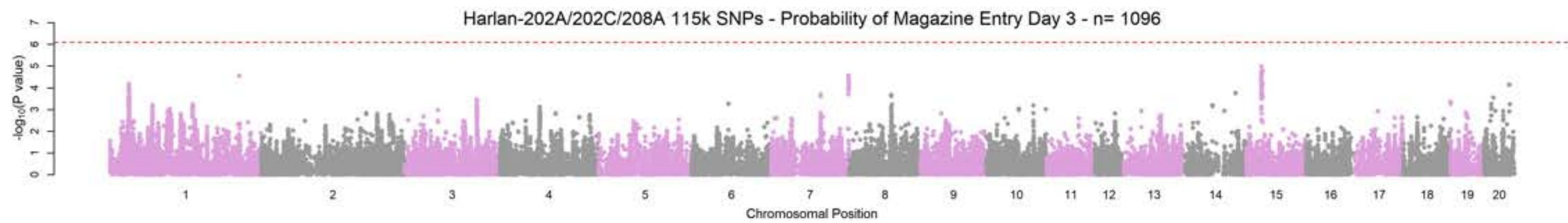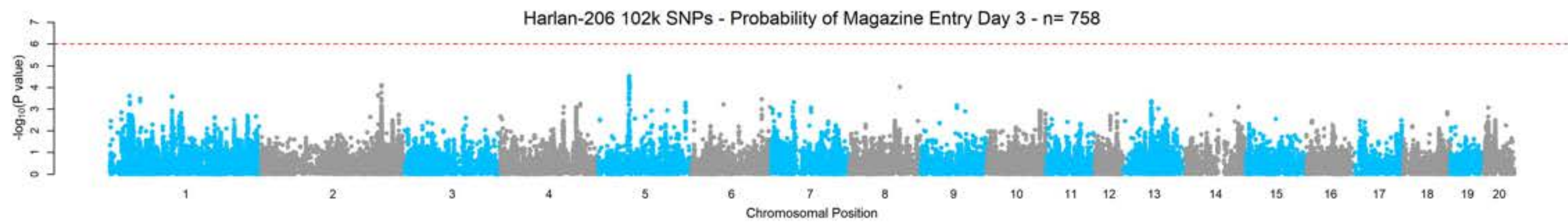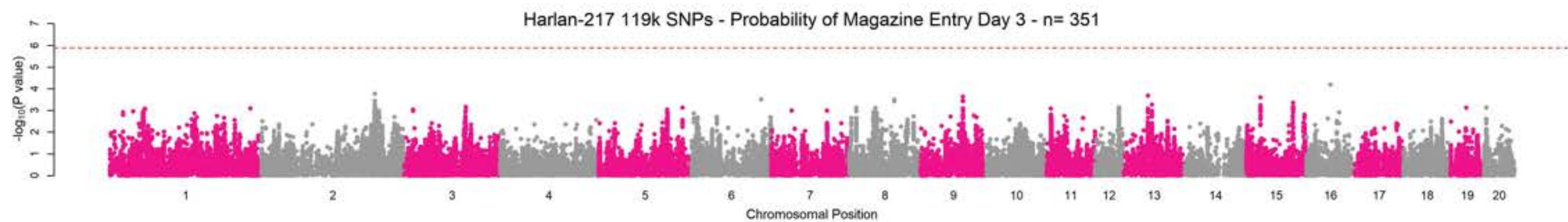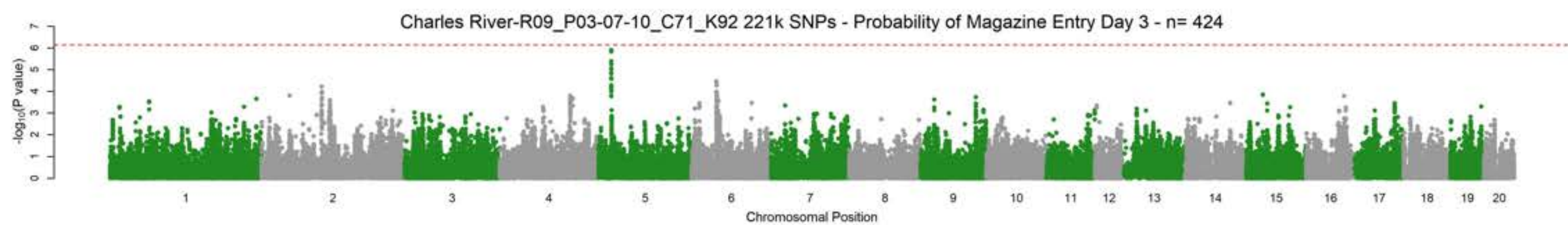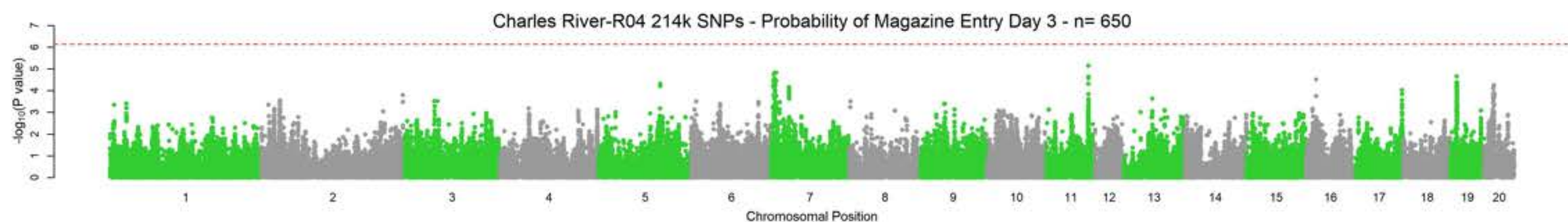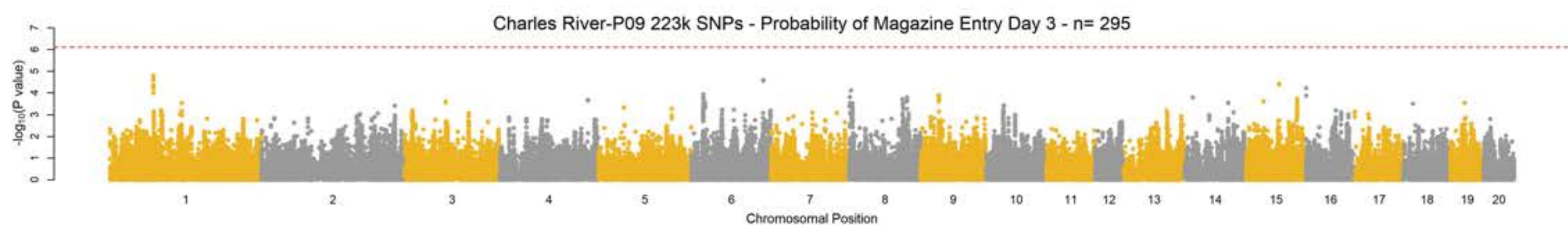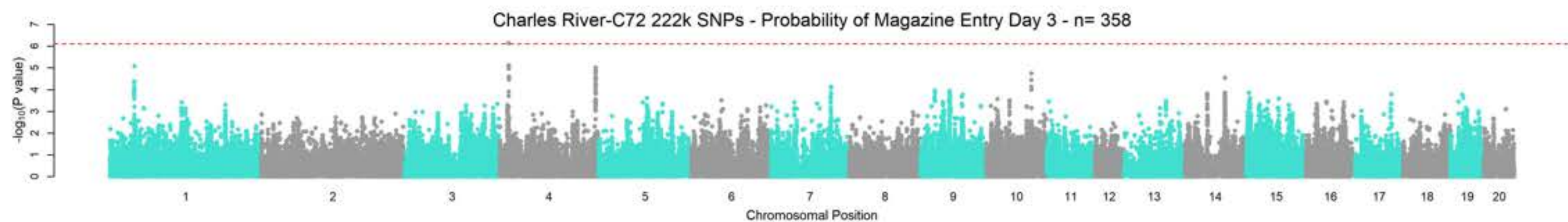

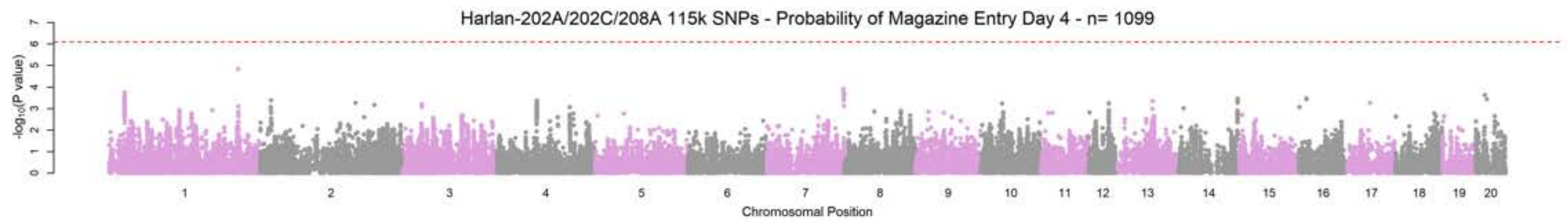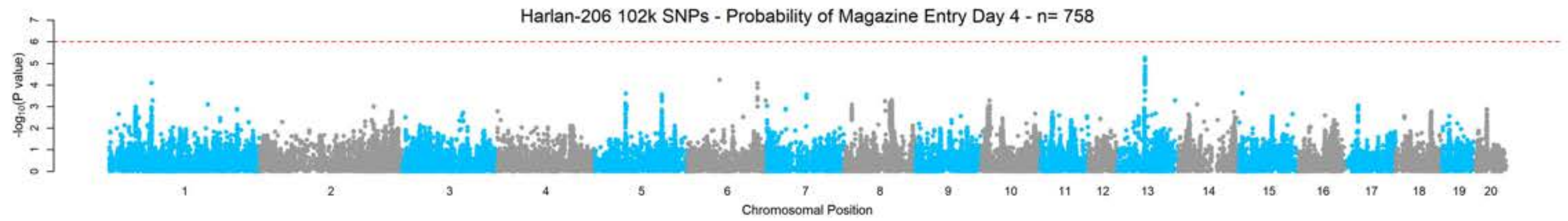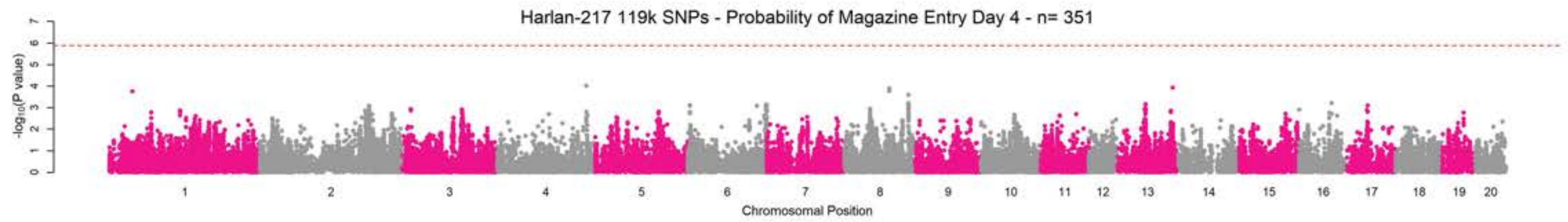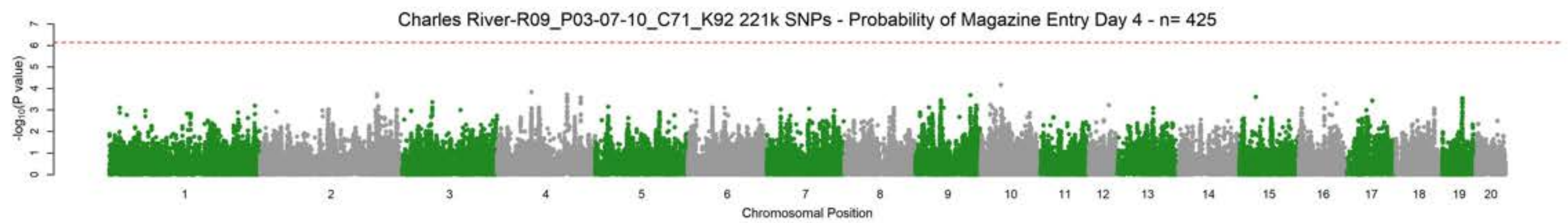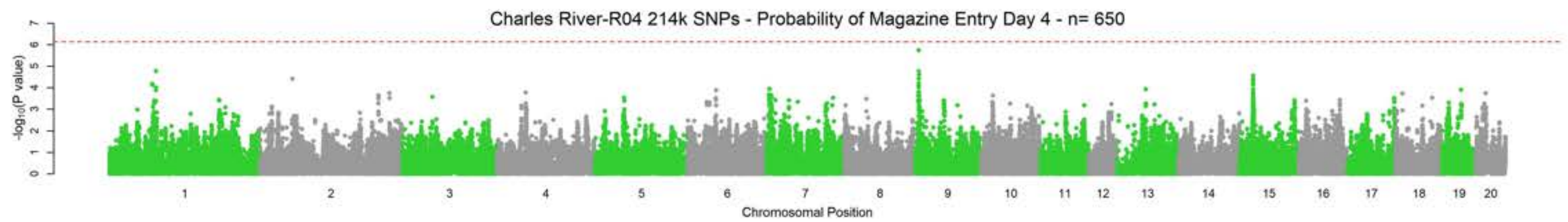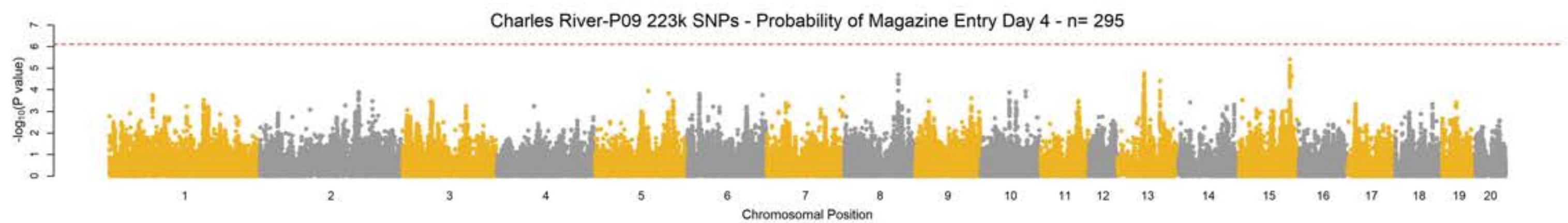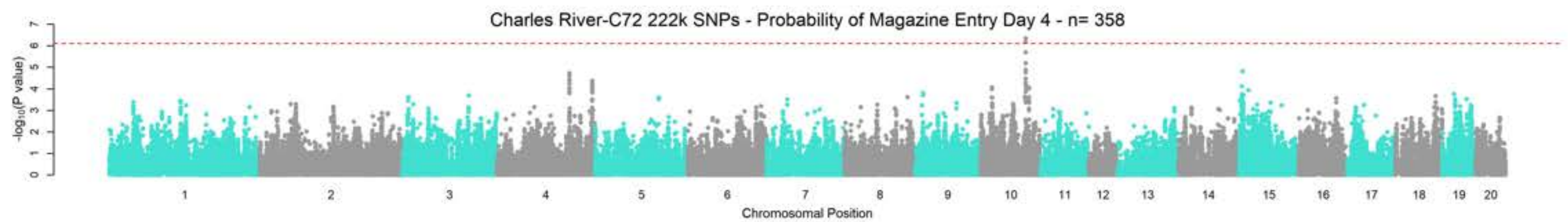

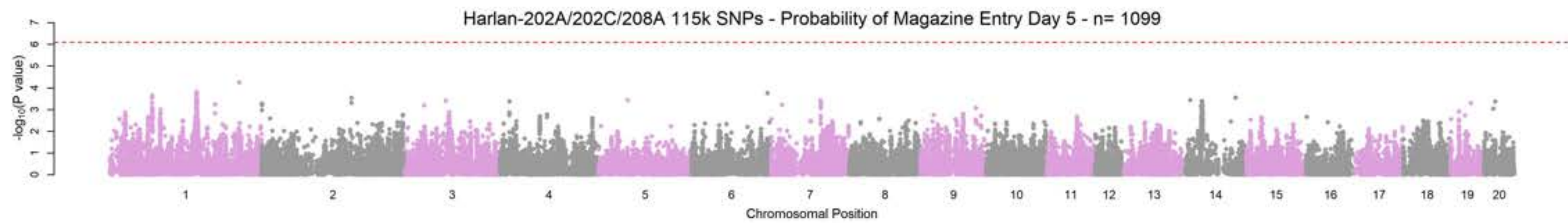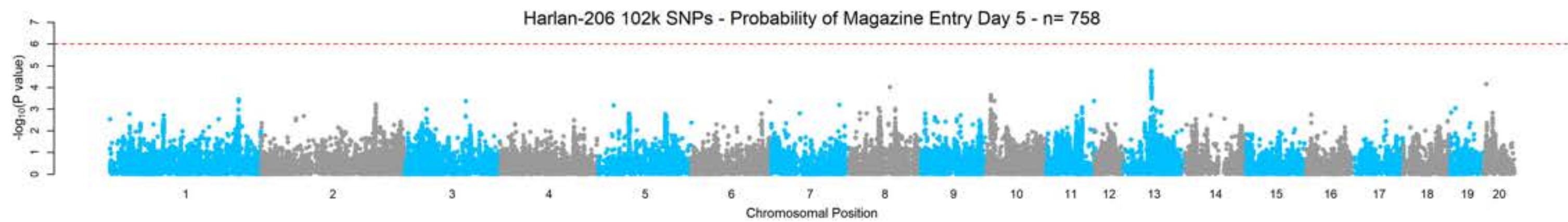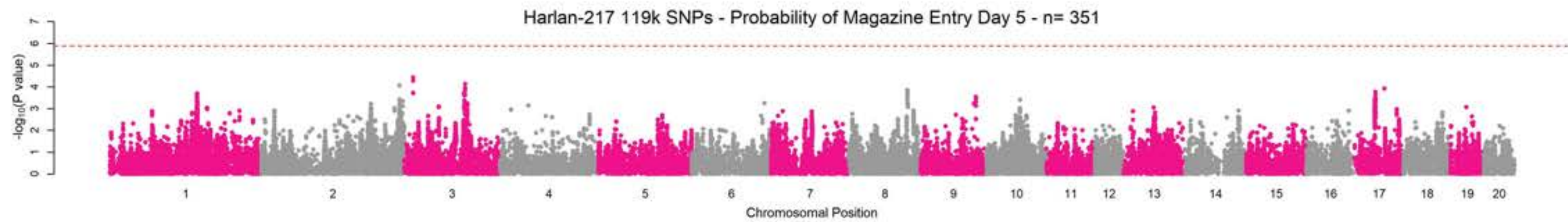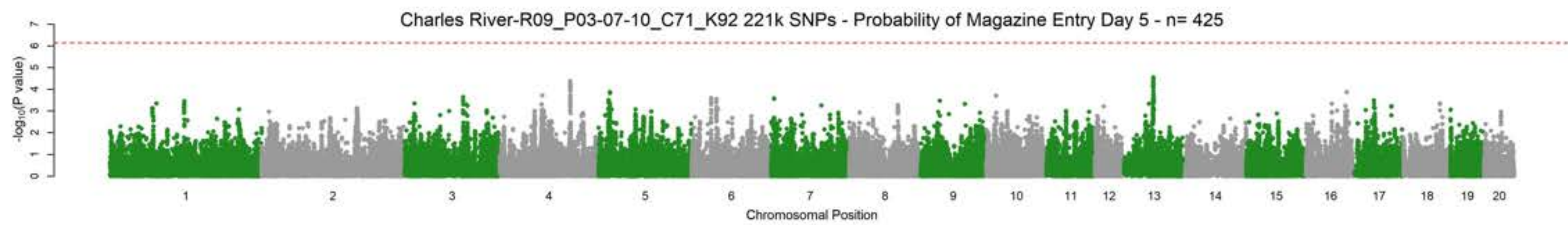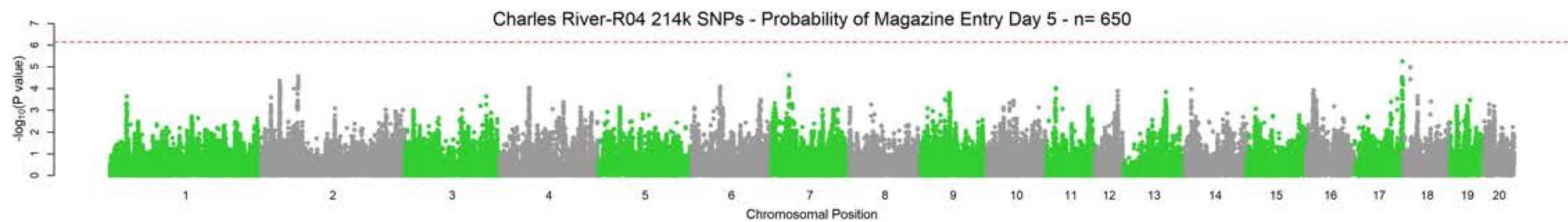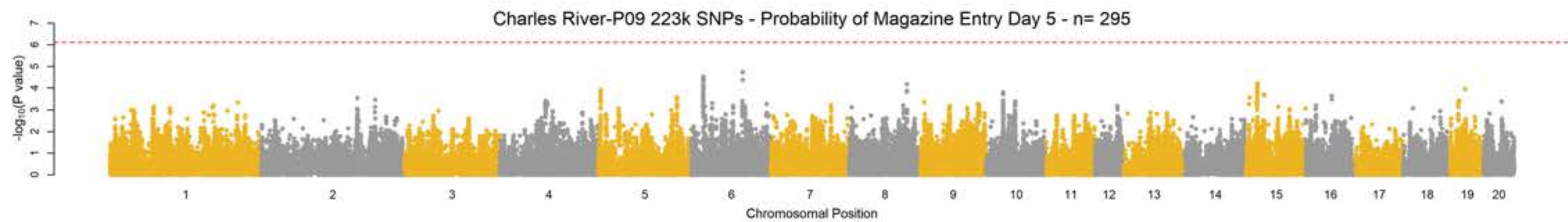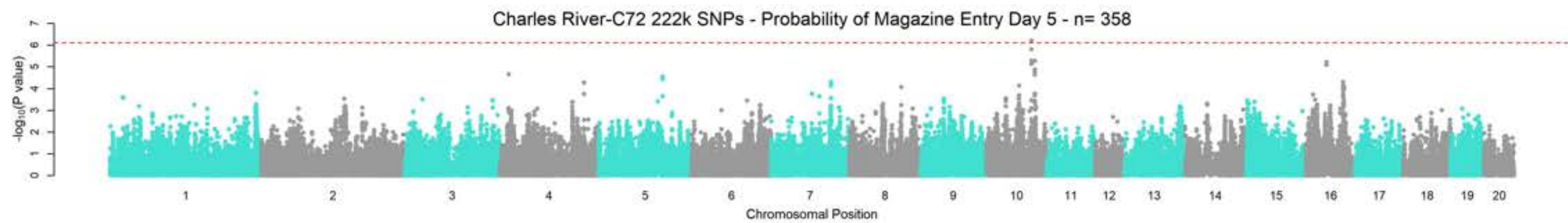

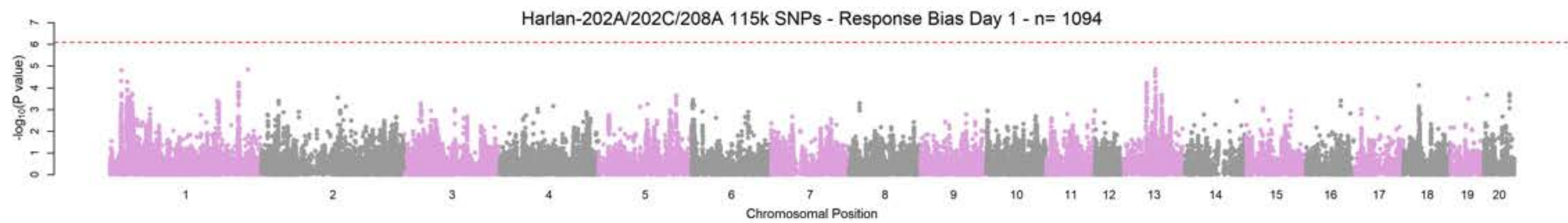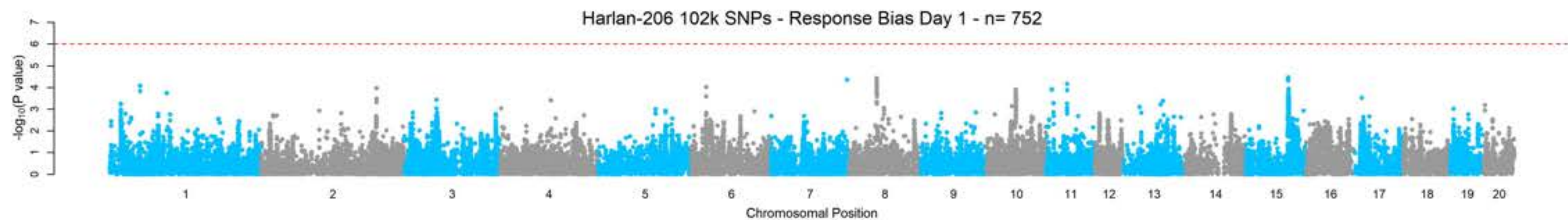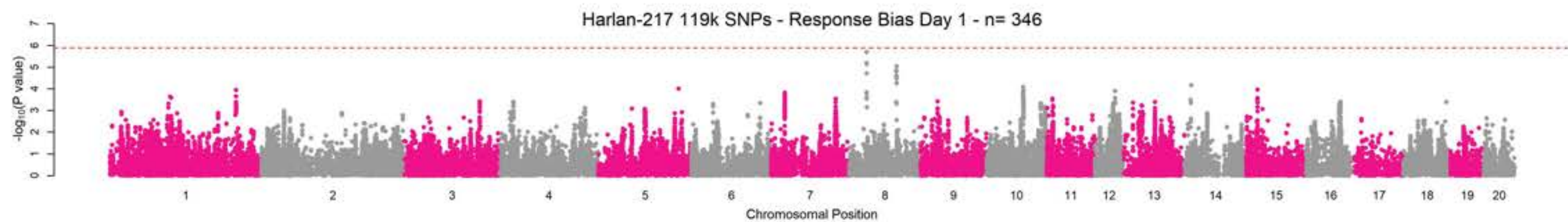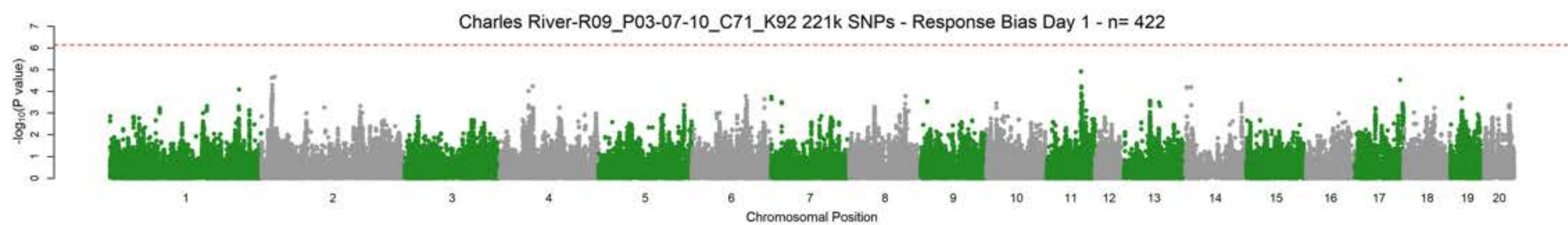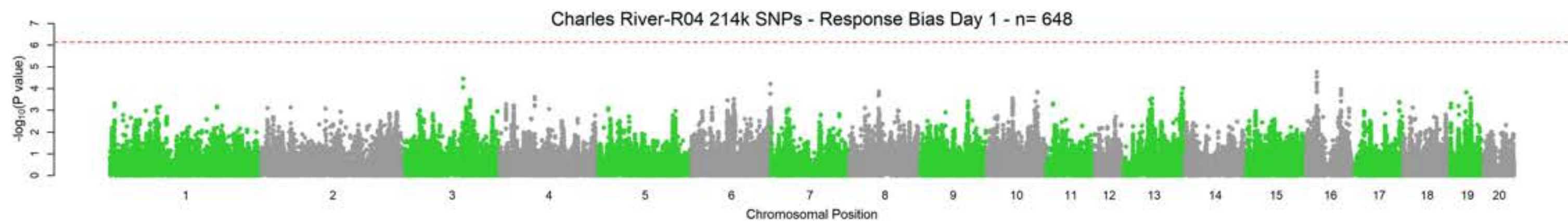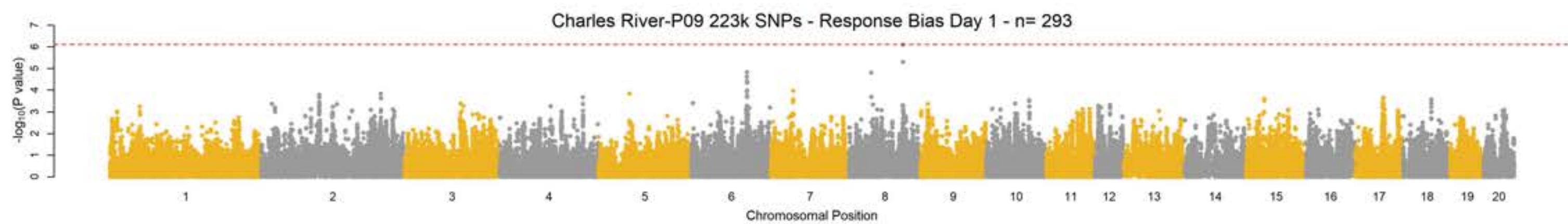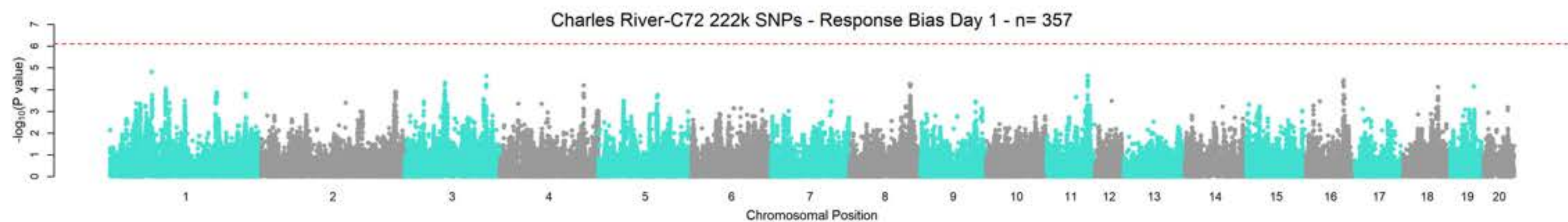

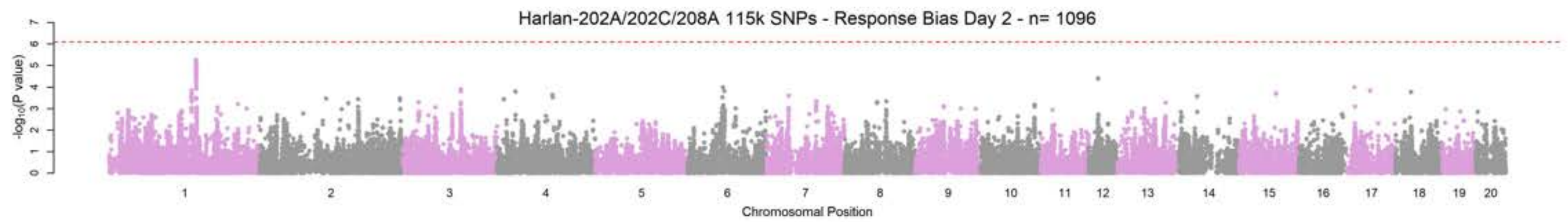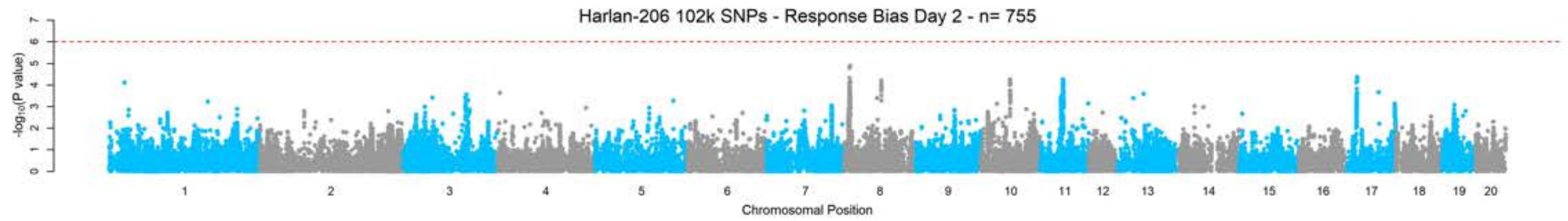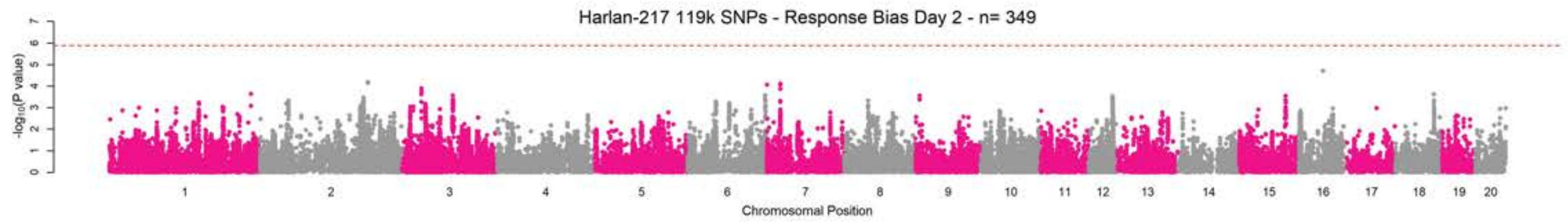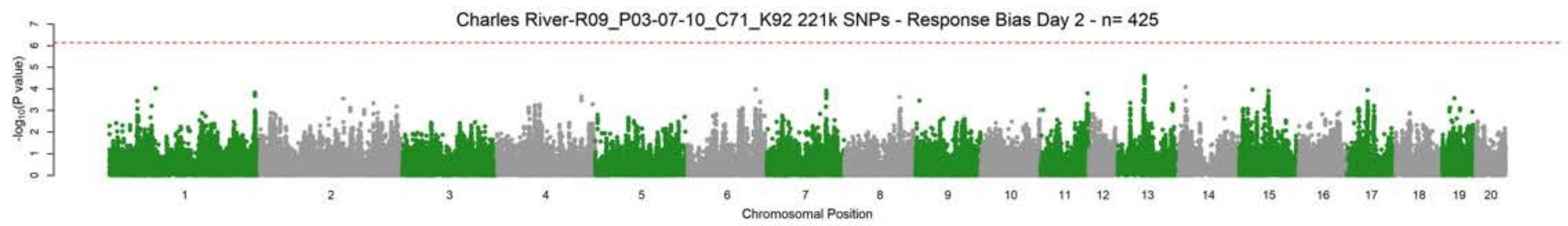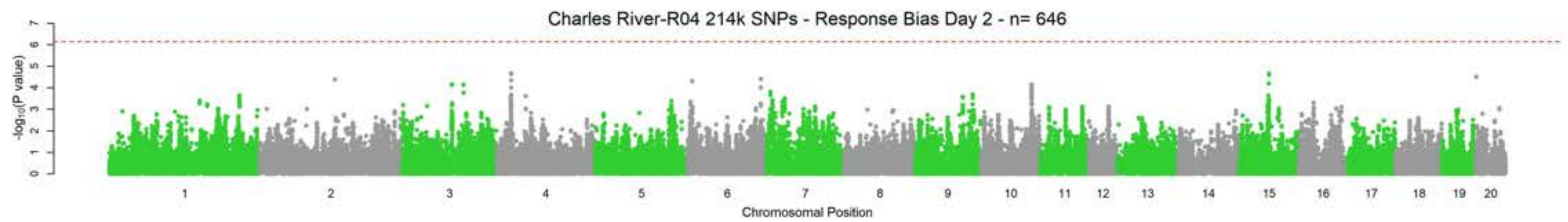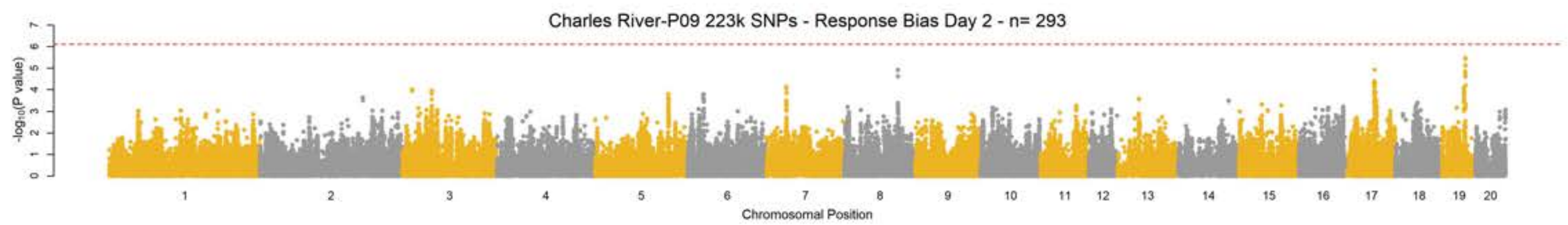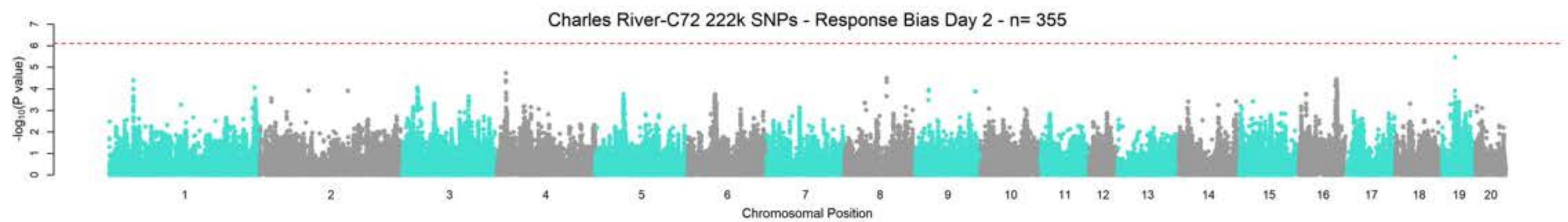

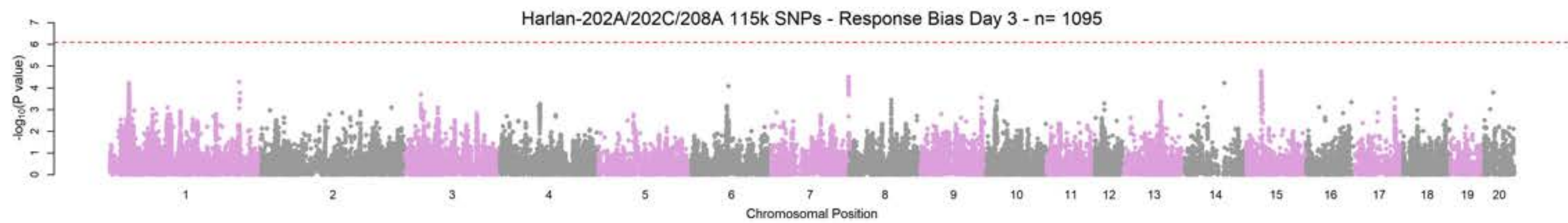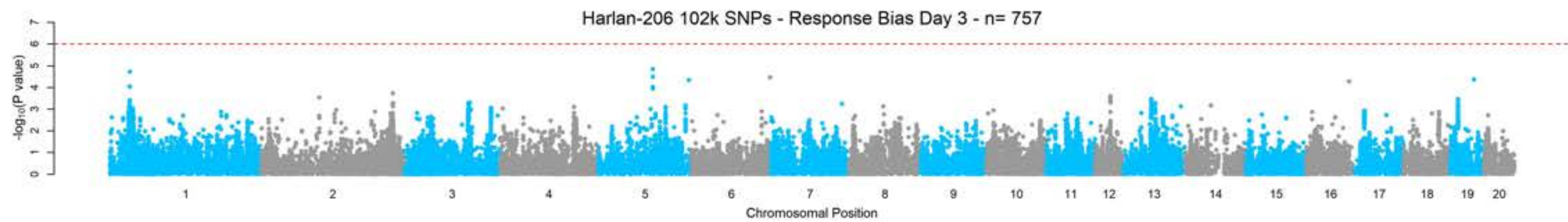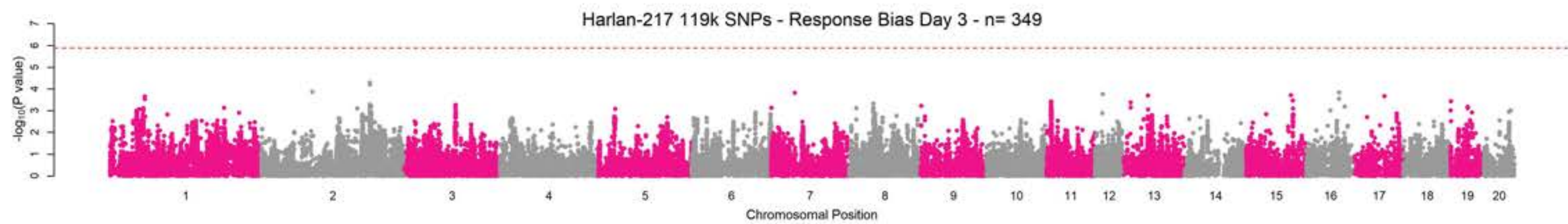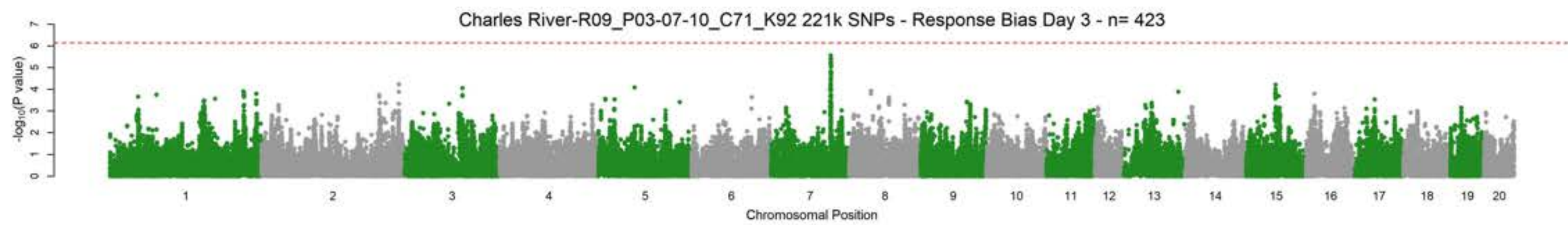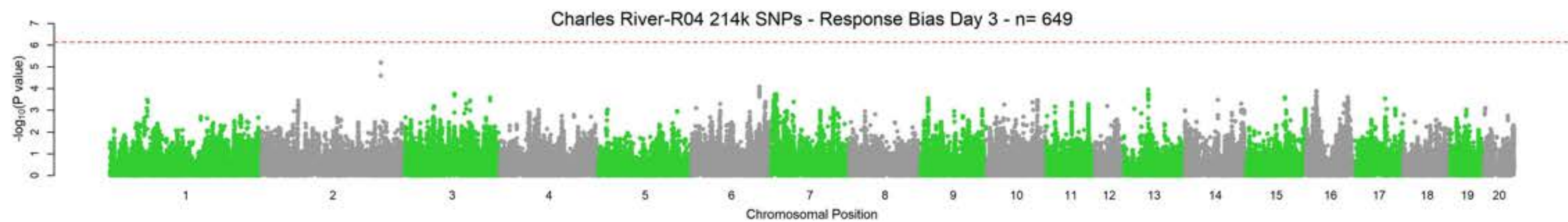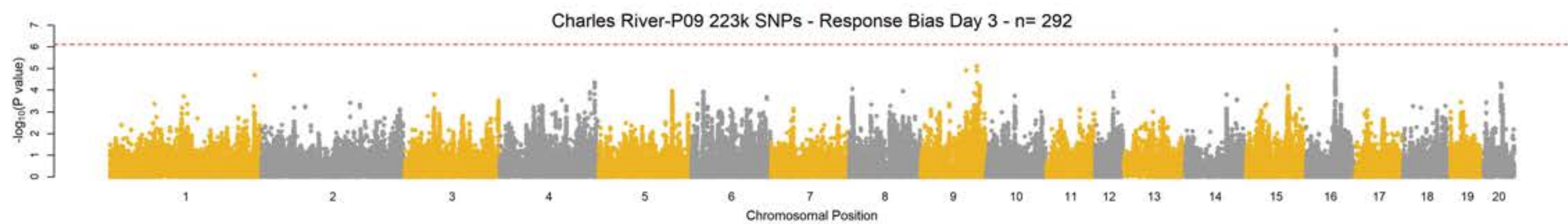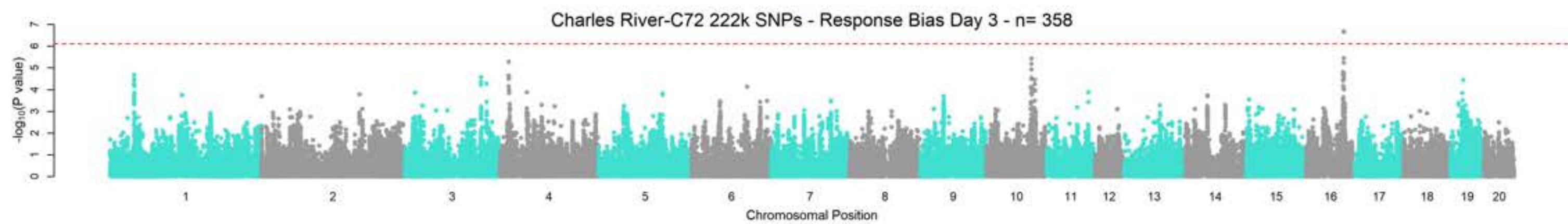

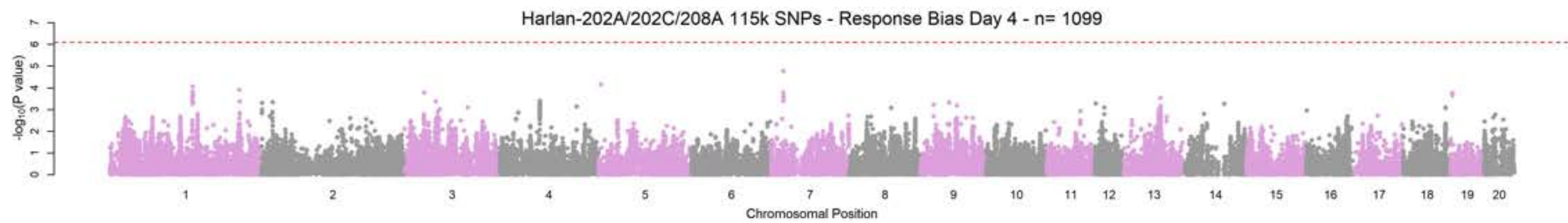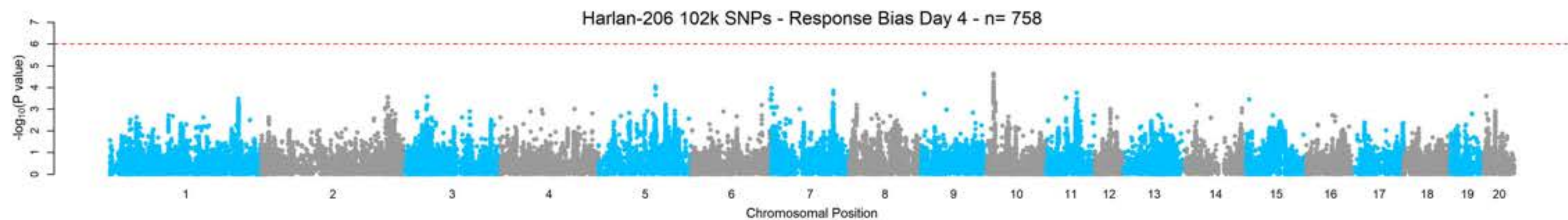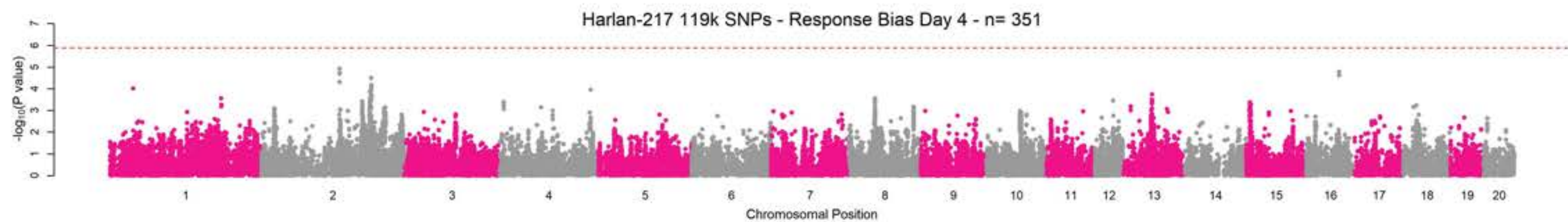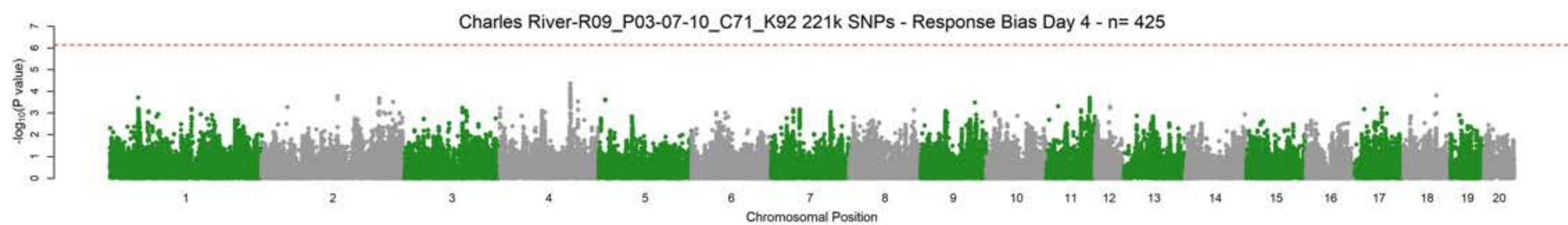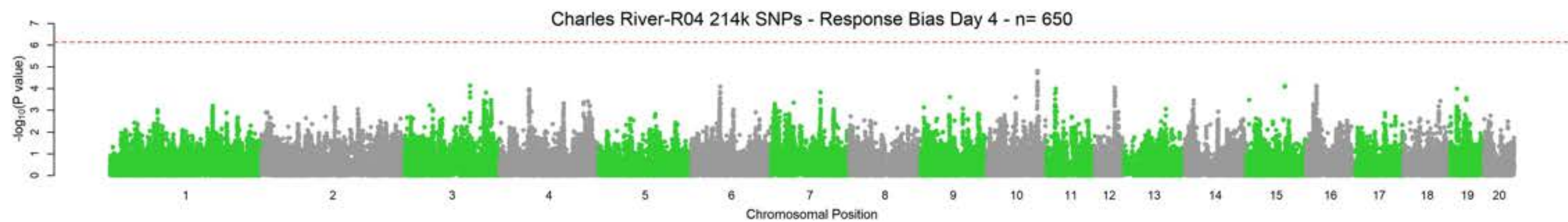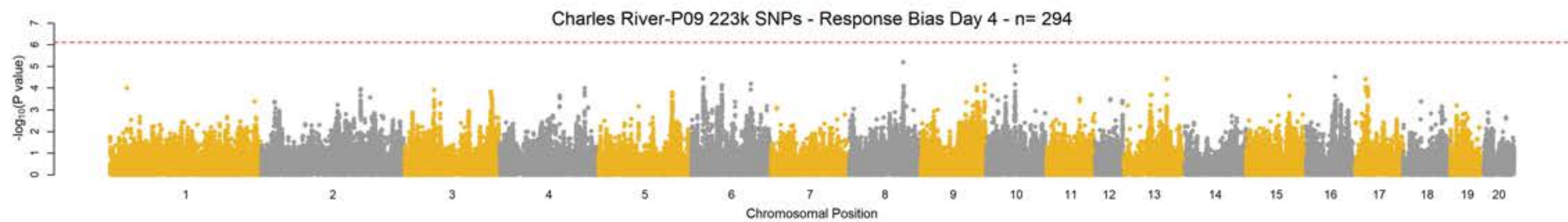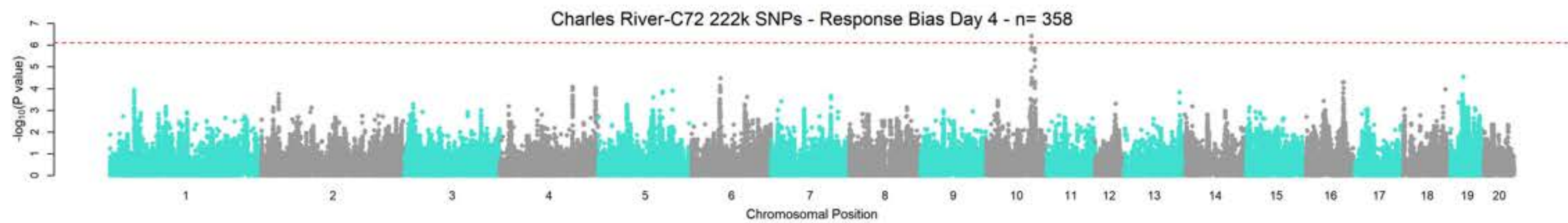

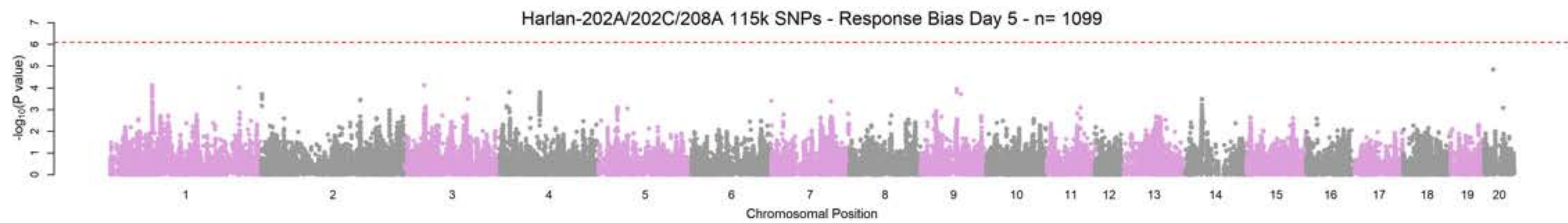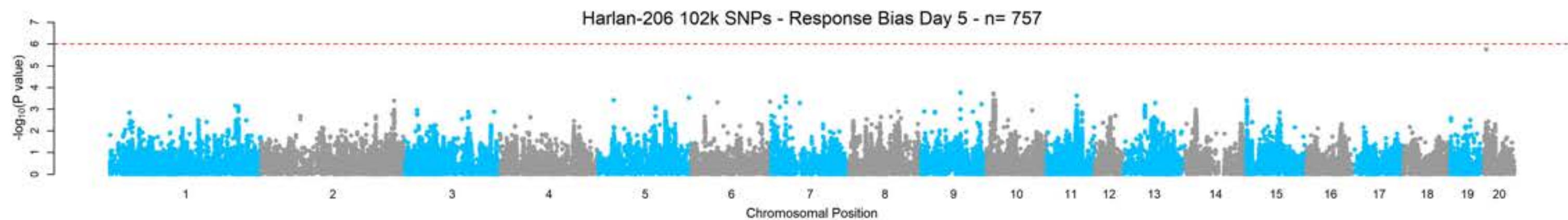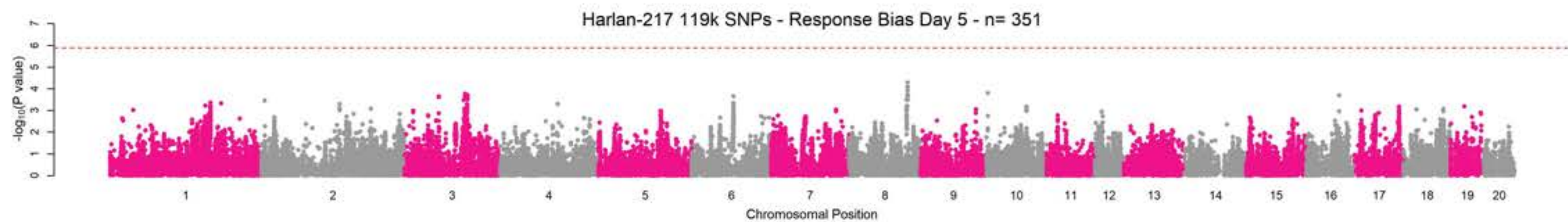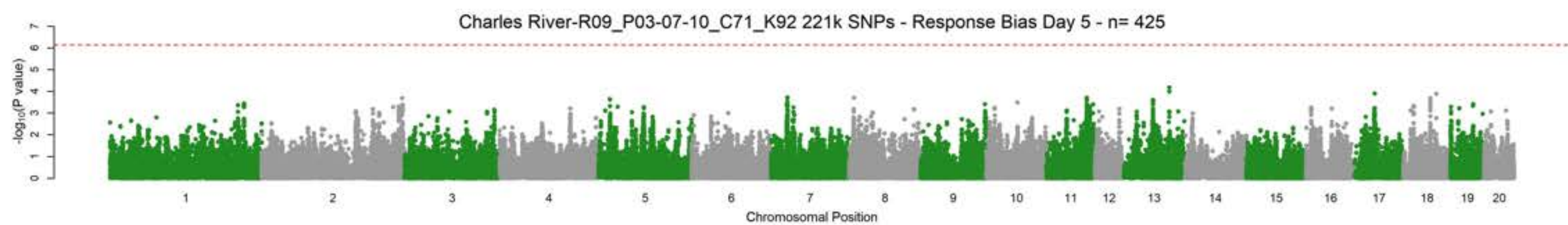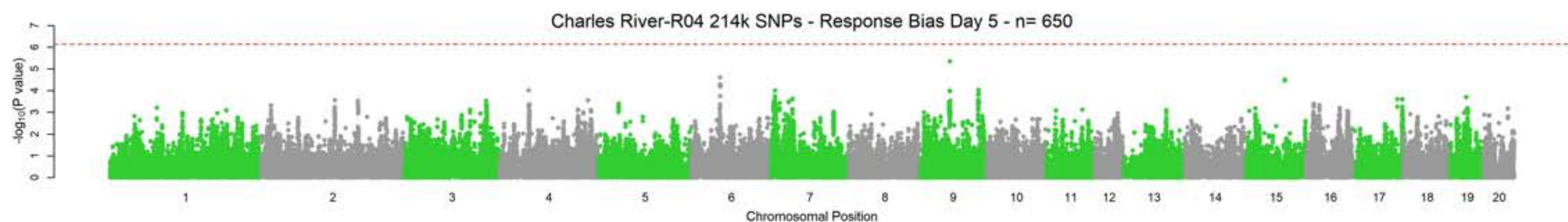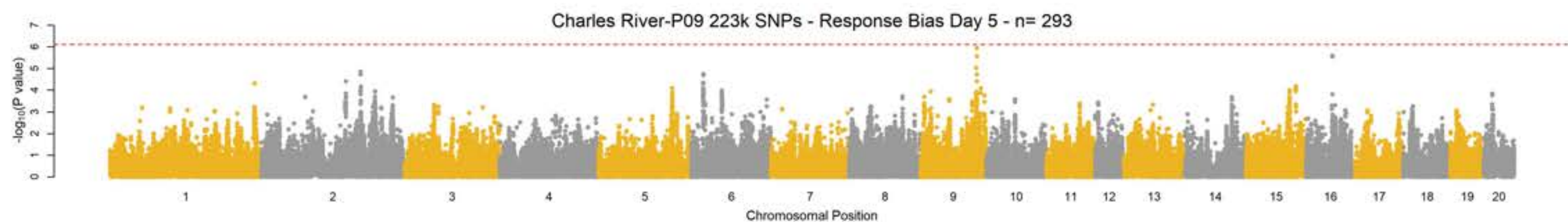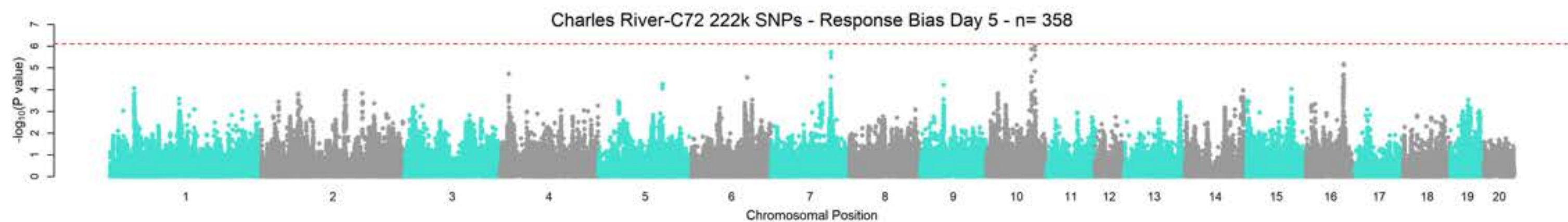

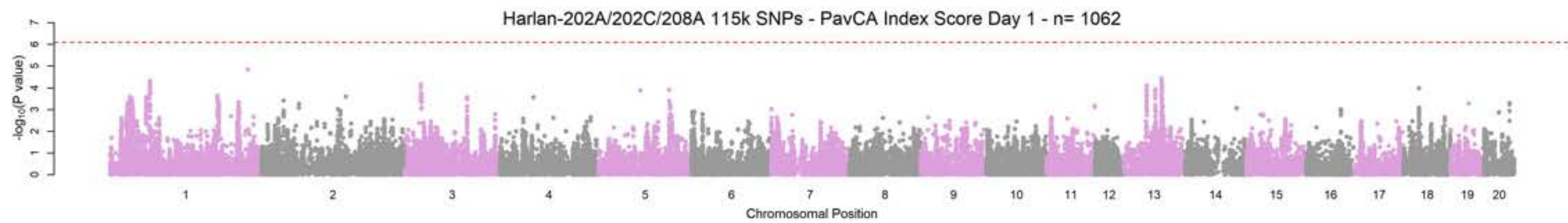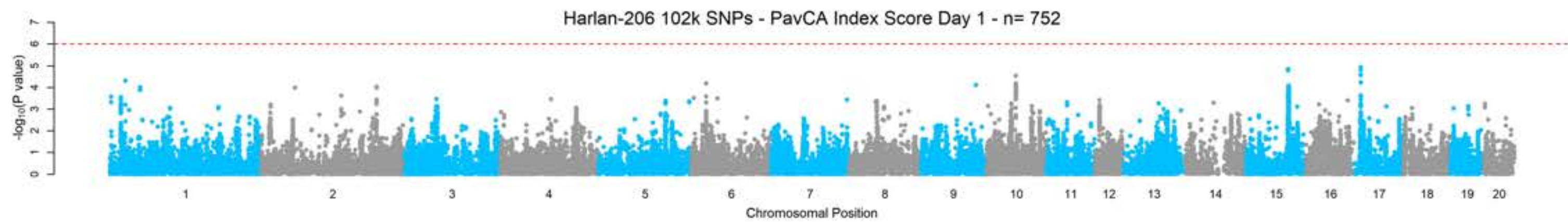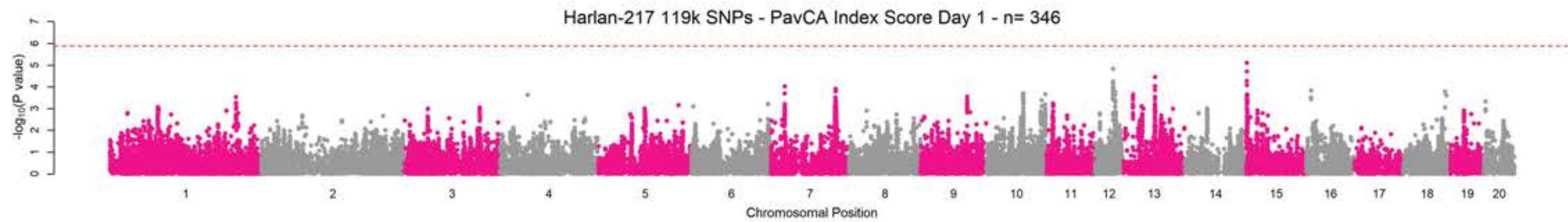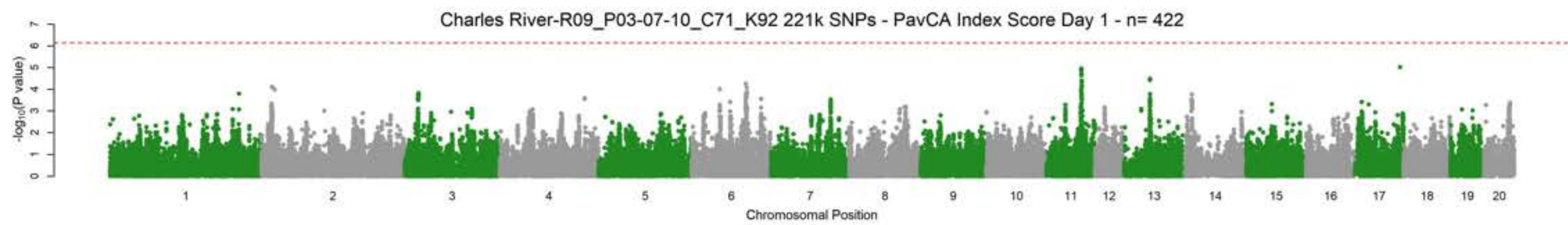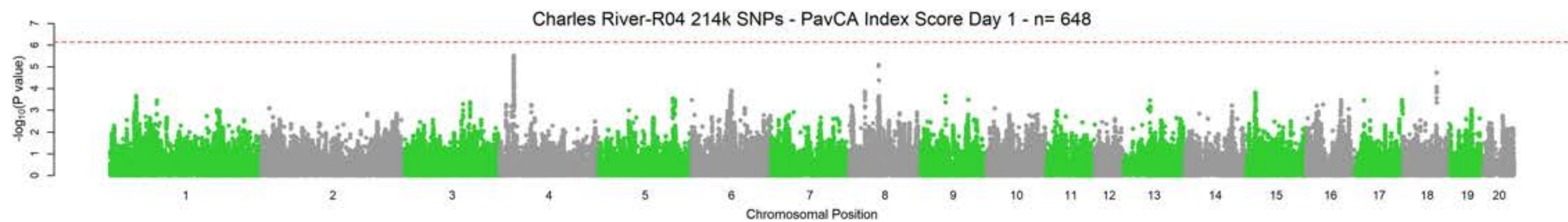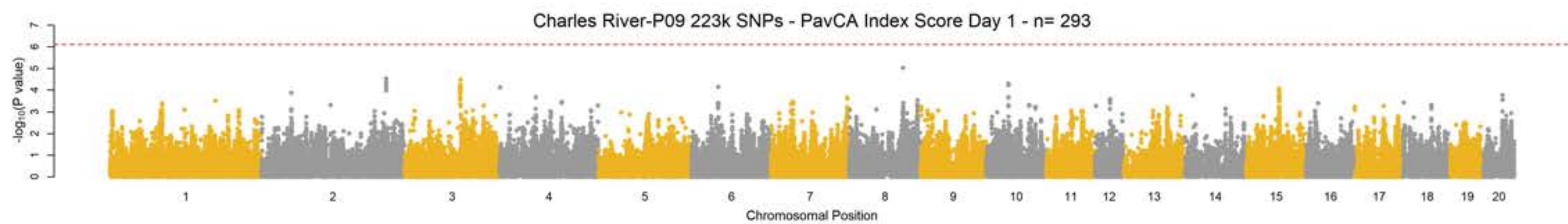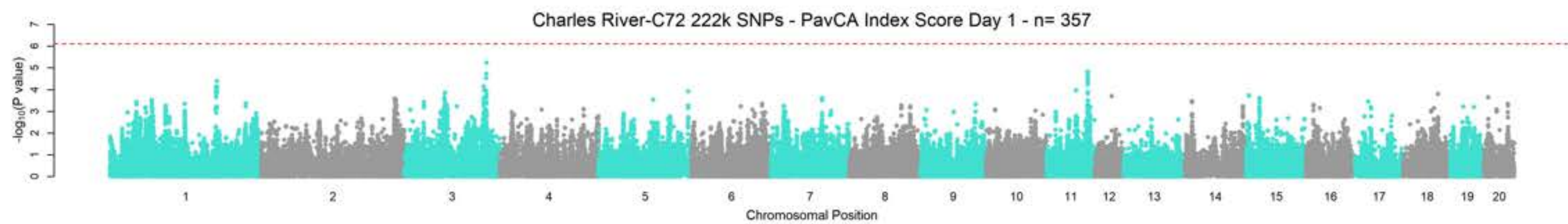

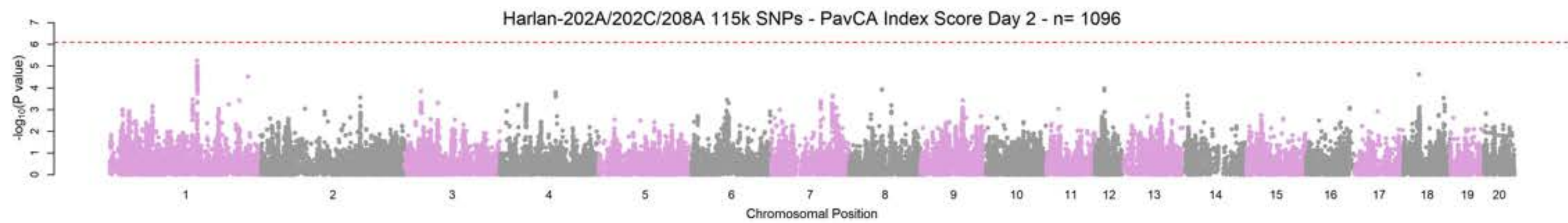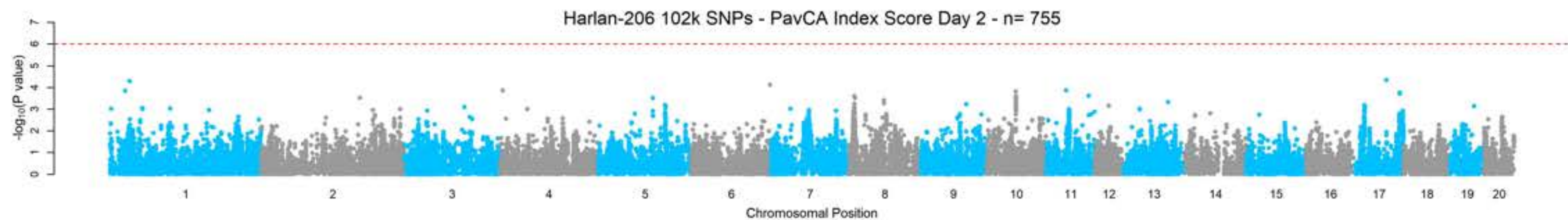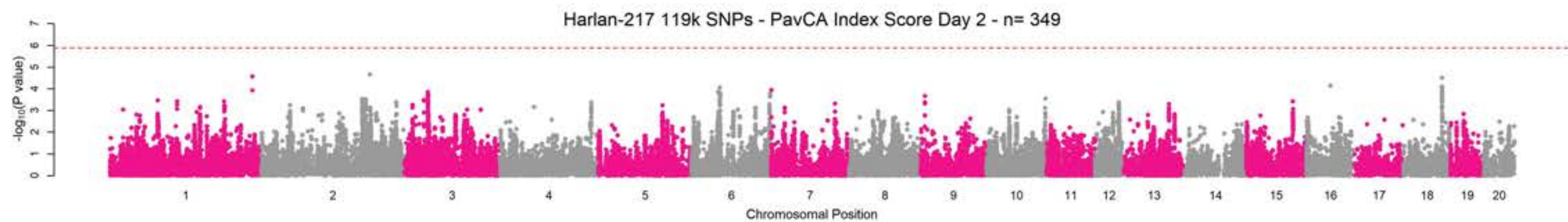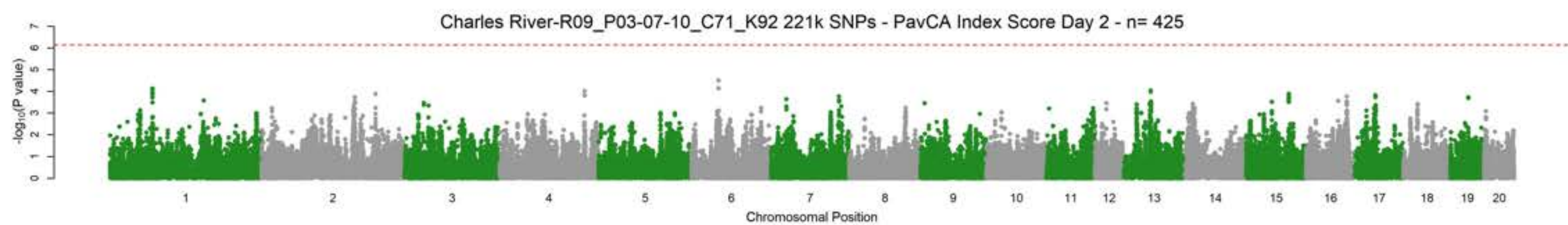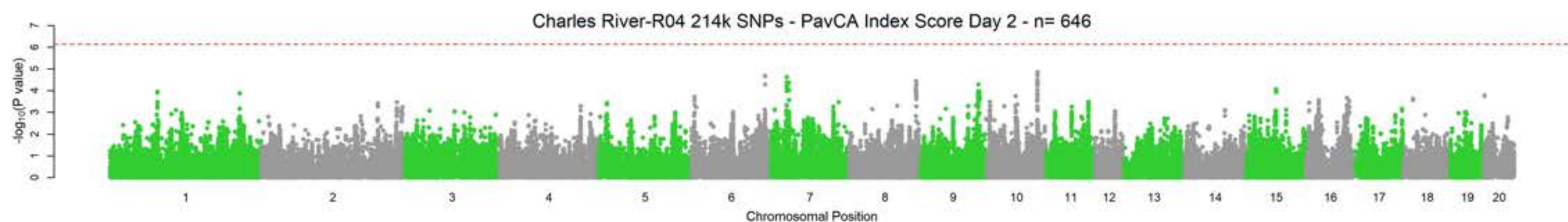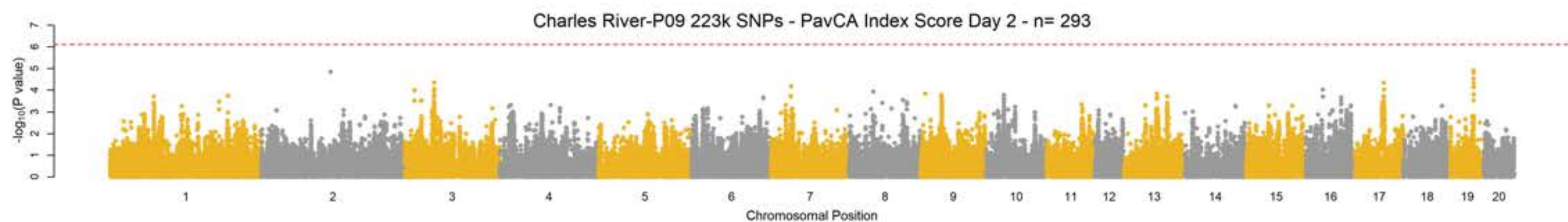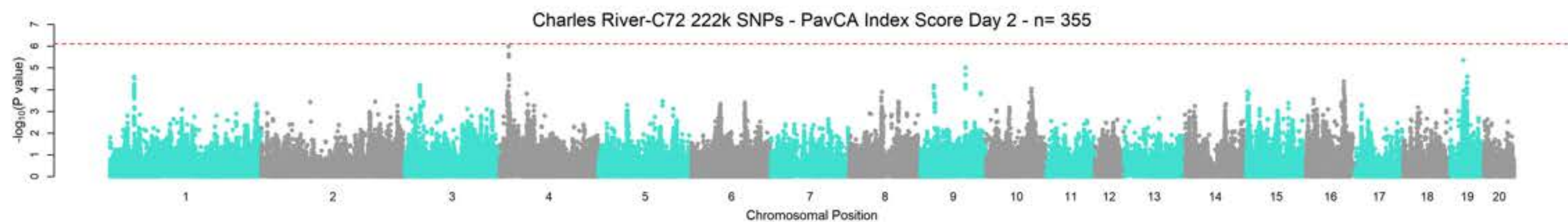

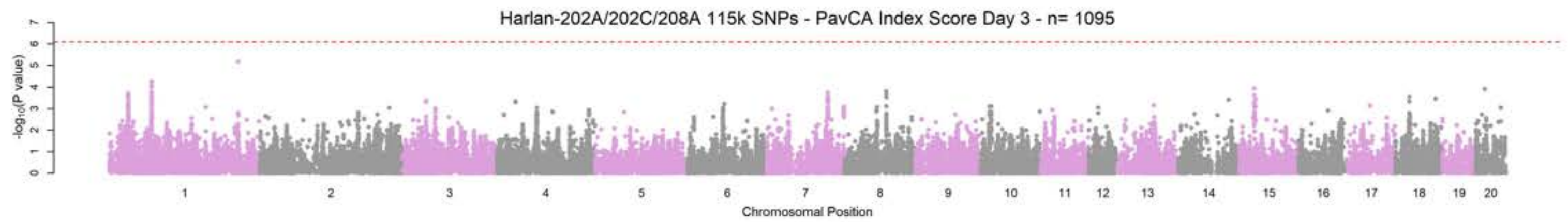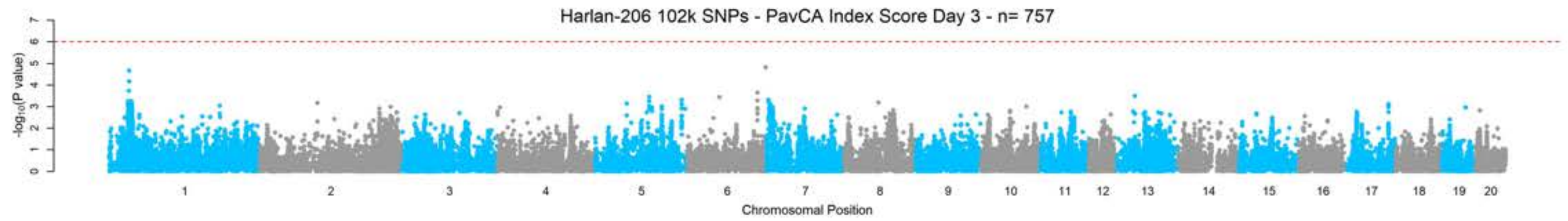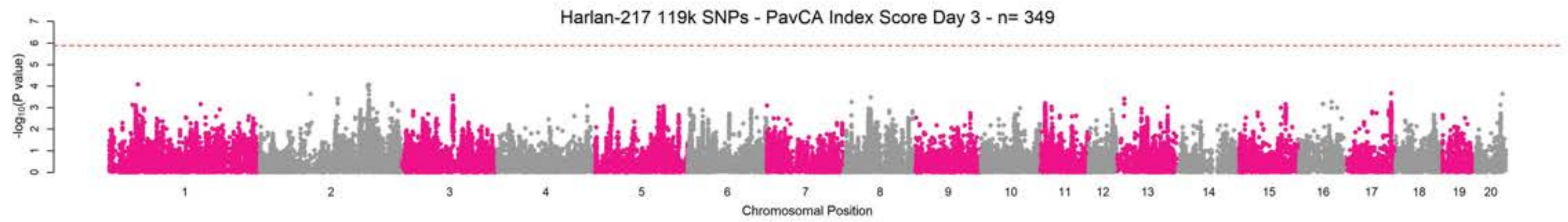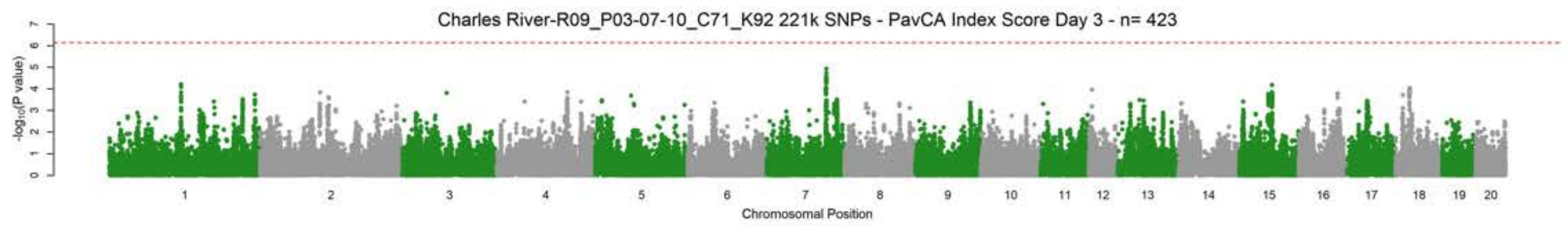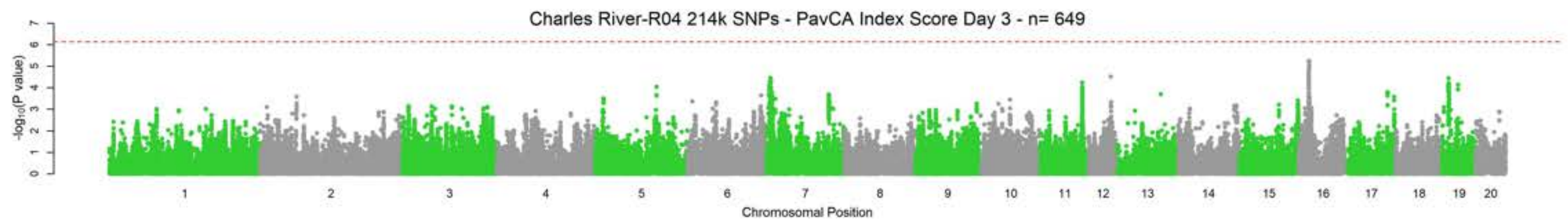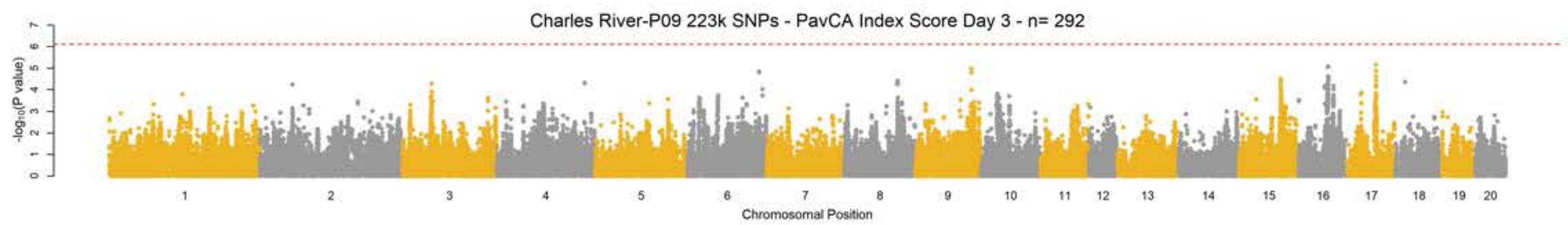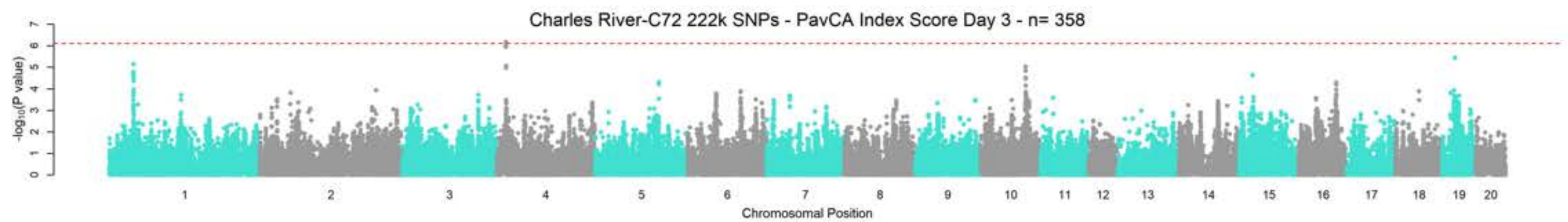

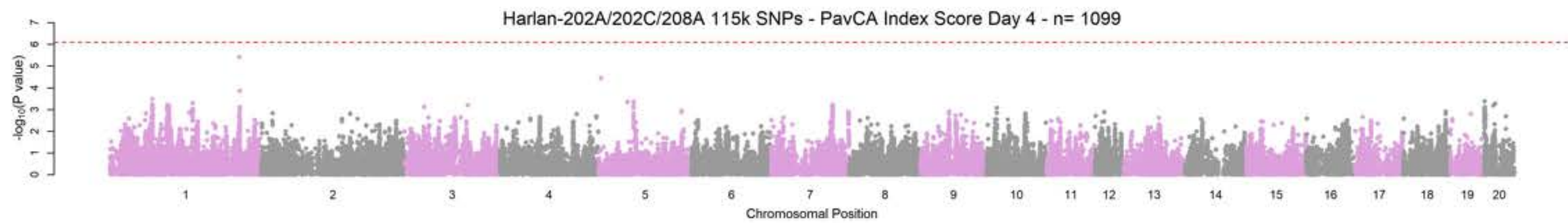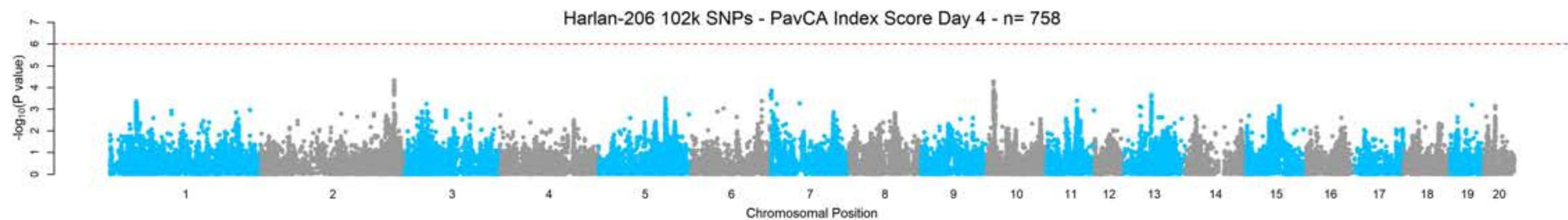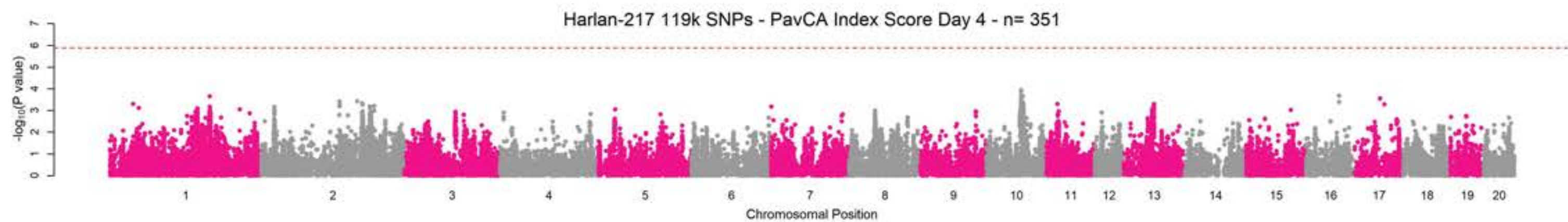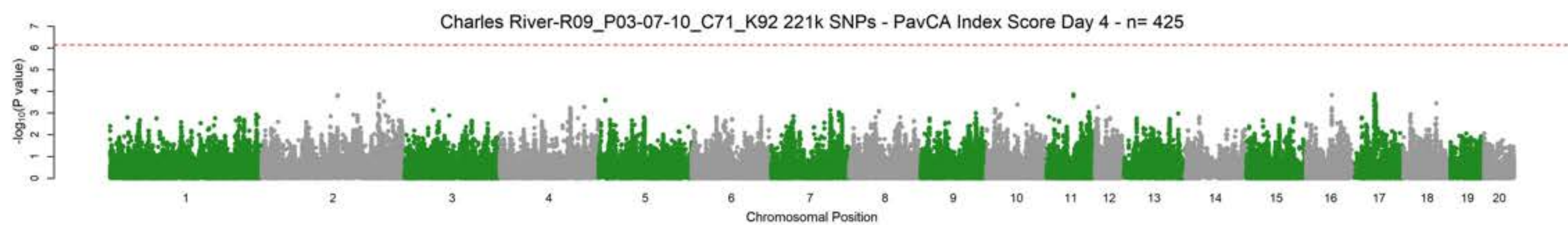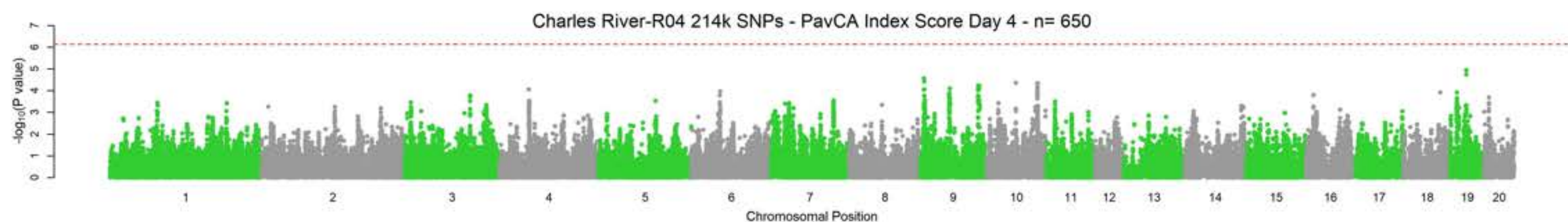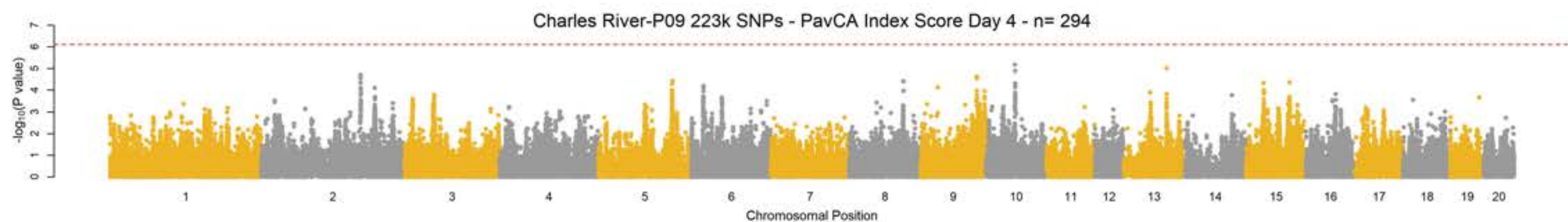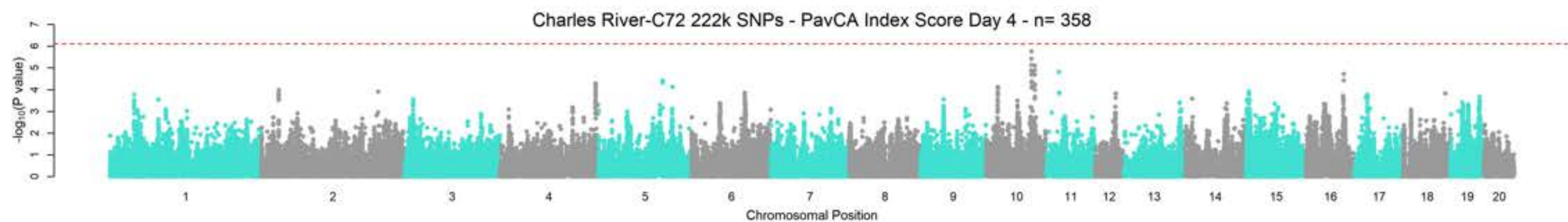

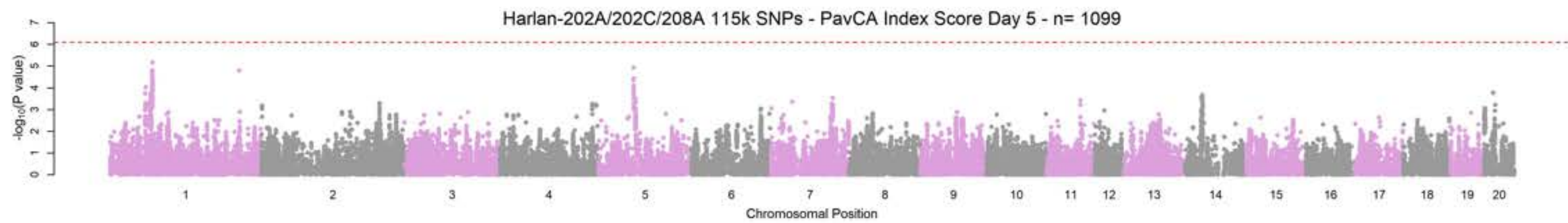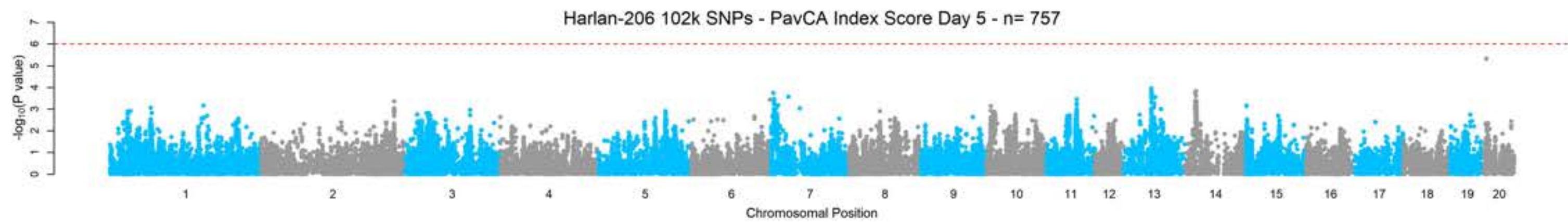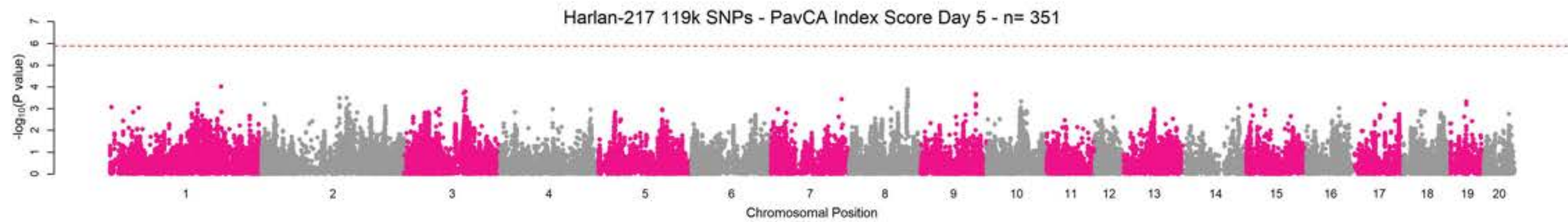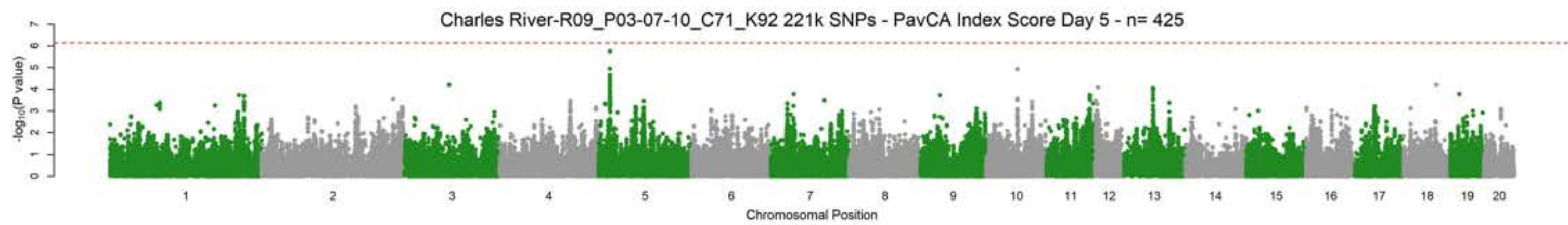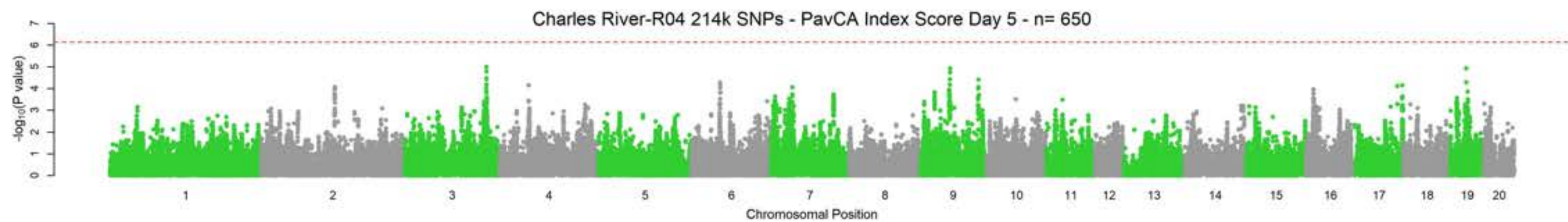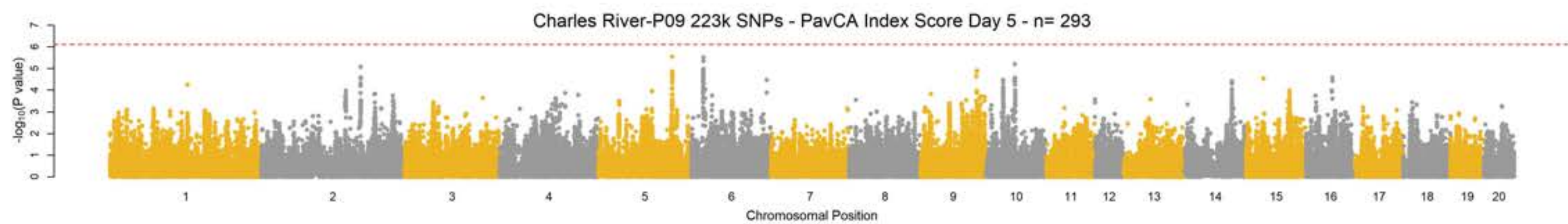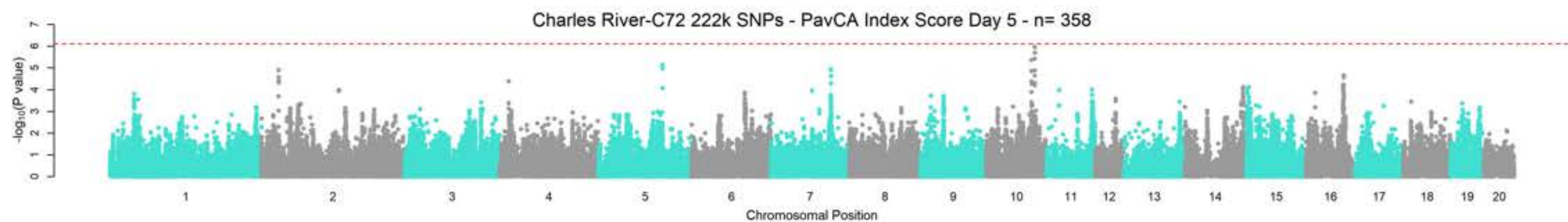

Supplement: S2 File — Each page contains vertically stacked Manhattan plots with the GWAS results for all PavCA metrics in all seven sample subgroups. The plots are in the following order from top to bottom: Harlan 202A/202C/208A (115k SNPs), Harlan 206 (102k SNPs), Harlan 217 (115k SNPs), Charles River R09/P03/P07/P10 (221k SNPs), Charles River R04 (214k SNPs), Charles River P09 (223k SNPs), and Charles River C72 (222k SNPs). (PDF) [file pgen.1010234.s020.pdf]
