# Supplementary material for: Genetic characterization of outbred Sprague Dawley rats and utility for genome-wide association studies
Source: PLoS Genet. 2022 May 31;18(5):e1010234. doi: 10.1371/journal.pgen.1010234 (PMC9187121; doi:10.1371/journal.pgen.1010234)
Supplement: S2 Fig — Histograms were constructed using measurements from the combined Harlan and Charles River sample set. Excluded from this plot is the PavCA index score. (PDF) [file pgen.1010234.s002.pdf]

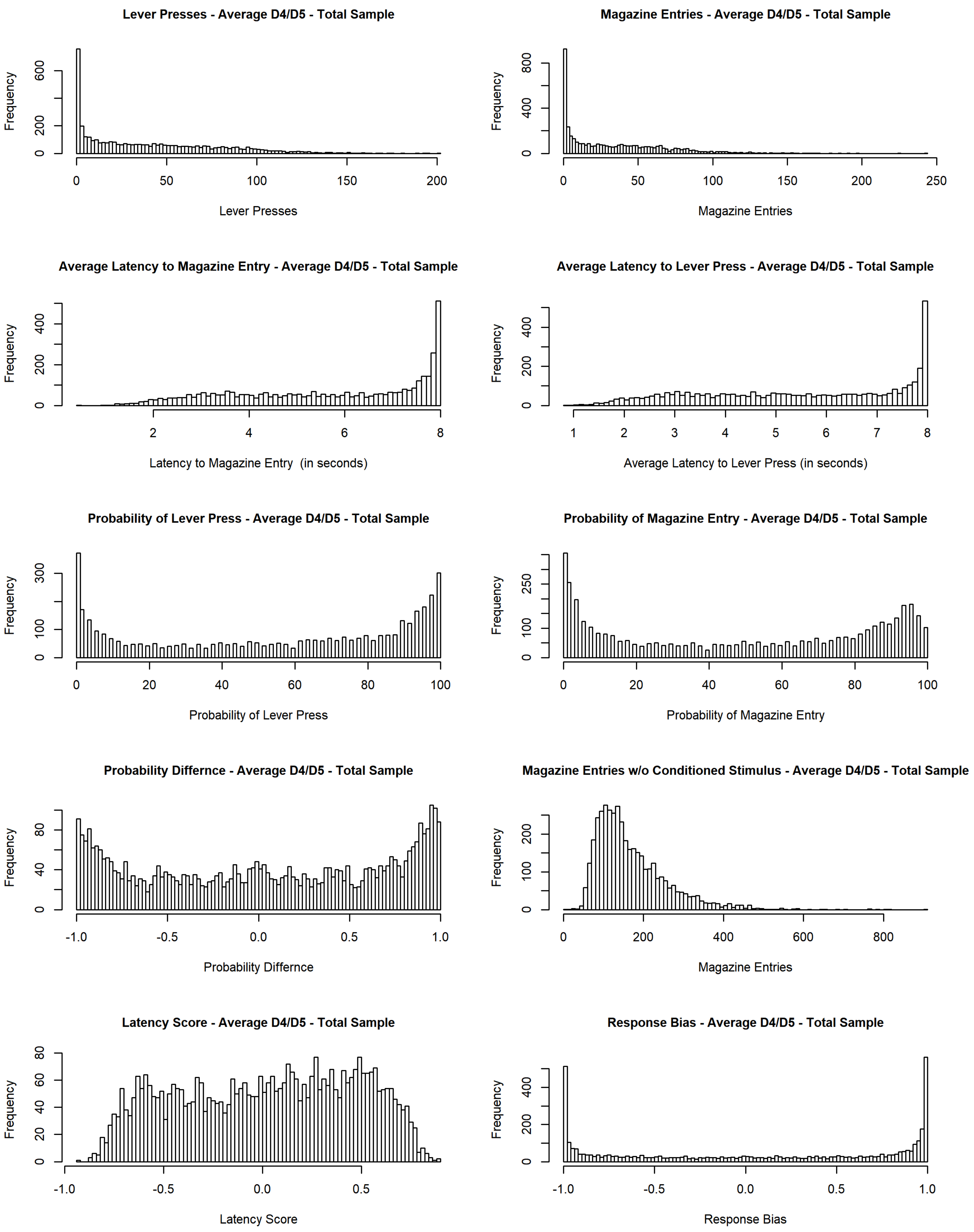
Lever Presses - Average D4/D5 - Total Sample
Frequency
Lever Presses
Magazine Entries - Average D4/D5 - Total Sample
Frequency
Magazine Entries
Average Latency to Magazine Entry - Average D4/D5 - Total Sample
Frequency
Latency to Magazine Entry (in seconds)
Average Latency to Lever Press - Average D4/D5 - Total Sample
Frequency
Average Latency to Lever Press (in seconds)
Probability of Lever Press - Average D4/D5 - Total Sample
Frequency
Probability of Lever Press
Probability of Magazine Entry - Average D4/D5 - Total Sample
Frequency
Probability of Magazine Entry
Probability Differnce - Average D4/D5 - Total Sample
Frequency
Probability Differnce
Magazine Entries w/o Conditioned Stimulus - Average D4/D5 - Total Sample
Frequency
Magazine Entries
Latency Score - Average D4/D5 - Total Sample
Frequency
Latency Score
Response Bias - Average D4/D5 - Total Sample
Frequency
Response Bias
