# Supplementary material for: Genetic characterization of outbred Sprague Dawley rats and utility for genome-wide association studies
Source: PLoS Genet. 2022 May 31;18(5):e1010234. doi: 10.1371/journal.pgen.1010234 (PMC9187121; doi:10.1371/journal.pgen.1010234)
Supplement: S3 Fig — Sample numbers for each breeding location can be found in S1 Table. The three locations in the left column are from Harlan, and the three locations in the right column belong to Charles River. (PDF) [file pgen.1010234.s003.pdf]

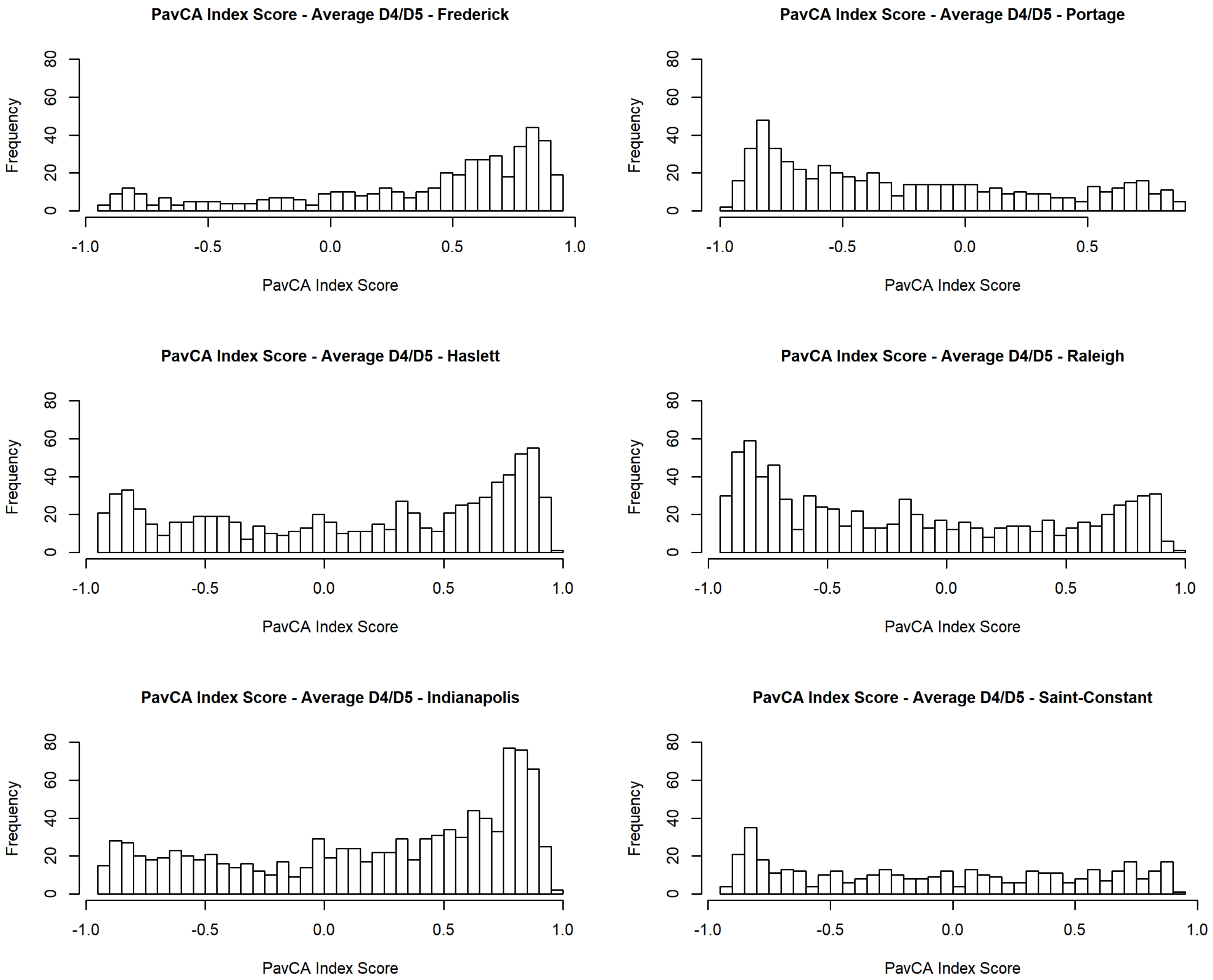

Breeding Location	Frederick	Haslett	Indianapolis	Portage	Raleigh	St. Constant
Frederick	1	1.955e-10	4.645e-06	<2.2e-16	<2.2e-16	<2.2e-16
Haslett		1	0.0131	<2.2e-16	<2.2e-16	<2.2e-16
Indianapolis			1	<2.2e-16	<2.2e-16	<2.2e-16
Portage				1	0.004155	6.657e-06
Raleigh					1	0.03225
St. Constant						1

Welch's 2-sample t-test p-values from pairwise comparisons of day4 and day 5 average PavCA index score distributions between different breeding locations.
