# Supplementary material for: Genetic characterization of outbred Sprague Dawley rats and utility for genome-wide association studies
Source: PLoS Genet. 2022 May 31;18(5):e1010234. doi: 10.1371/journal.pgen.1010234 (PMC9187121; doi:10.1371/journal.pgen.1010234)
Supplement: S4 Fig — Panels A and C show pre-filtering values of P(IBD) = 0 plotted against P(IBD) = 1 for Harlan and Charles River. Unrelated samples cluster in the lower right corner. Samples along the diagonal have 2nd and 3rd degree levels of relatedness, while those clustering around (0.5,0.25) are full-siblings. Panels B and D demonstrate that the sample filtering steps removed several spurious relations from the sample. (PDF) [file pgen.1010234.s004.pdf]

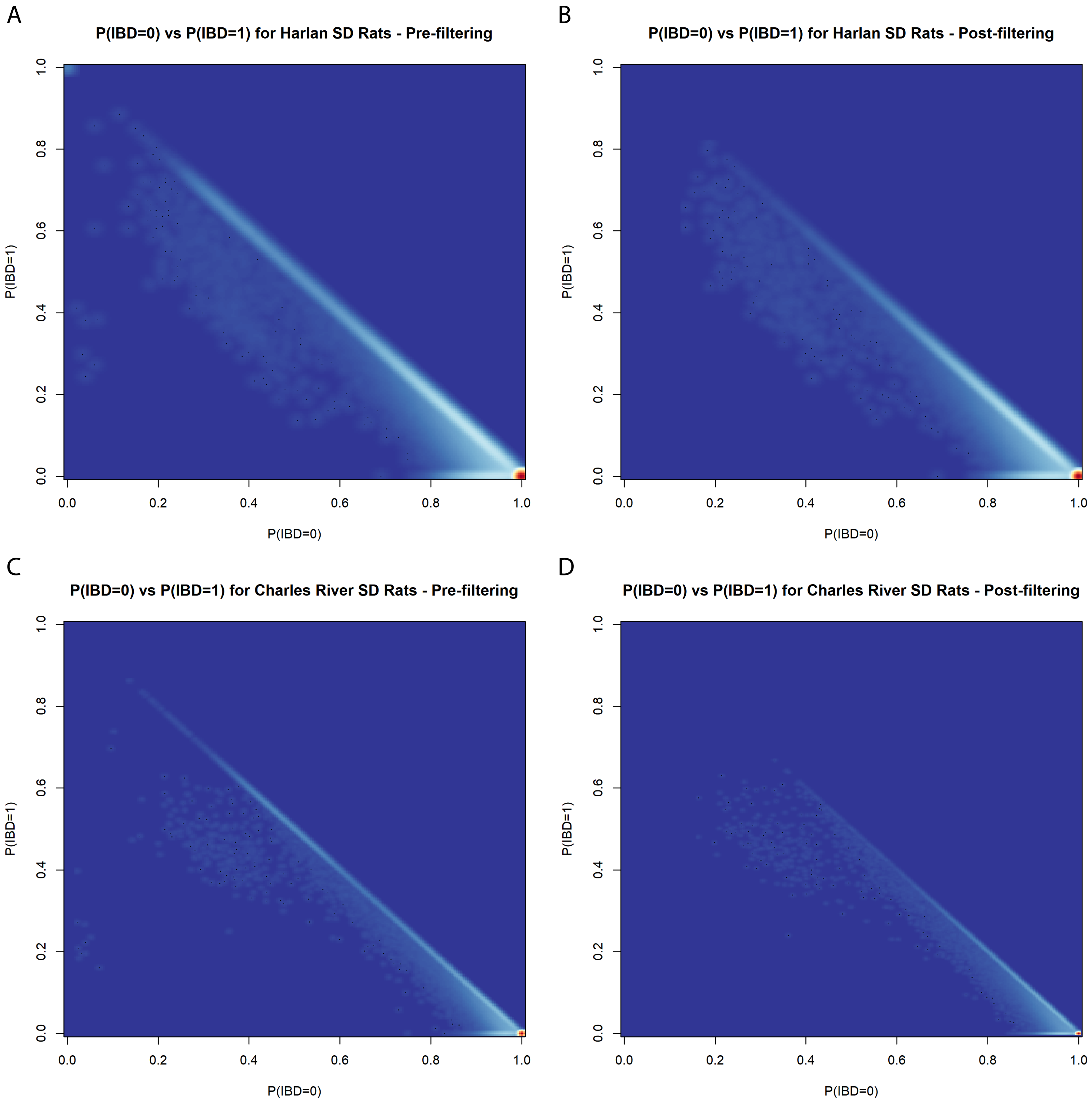
A
P(IBD=0) vs P(IBD=1) for Harlan SD Rats - Pre-filtering
B
P(IBD=0) vs P(IBD=1) for Harlan SD Rats - Post-filtering
C
P(IBD=0) vs P(IBD=1) for Charles River SD Rats - Pre-filtering
D
P(IBD=0) vs P(IBD=1) for Charles River SD Rats - Post-filtering
P(IBD=1)
P(IBD=0)
0.0
0.2
0.4
0.6
0.8
1.0
