# Supplementary material for: Genetic characterization of outbred Sprague Dawley rats and utility for genome-wide association studies
Source: PLoS Genet. 2022 May 31;18(5):e1010234. doi: 10.1371/journal.pgen.1010234 (PMC9187121; doi:10.1371/journal.pgen.1010234)

S5 Fig. QQ-plot inflation observed in vendor-level GWAS

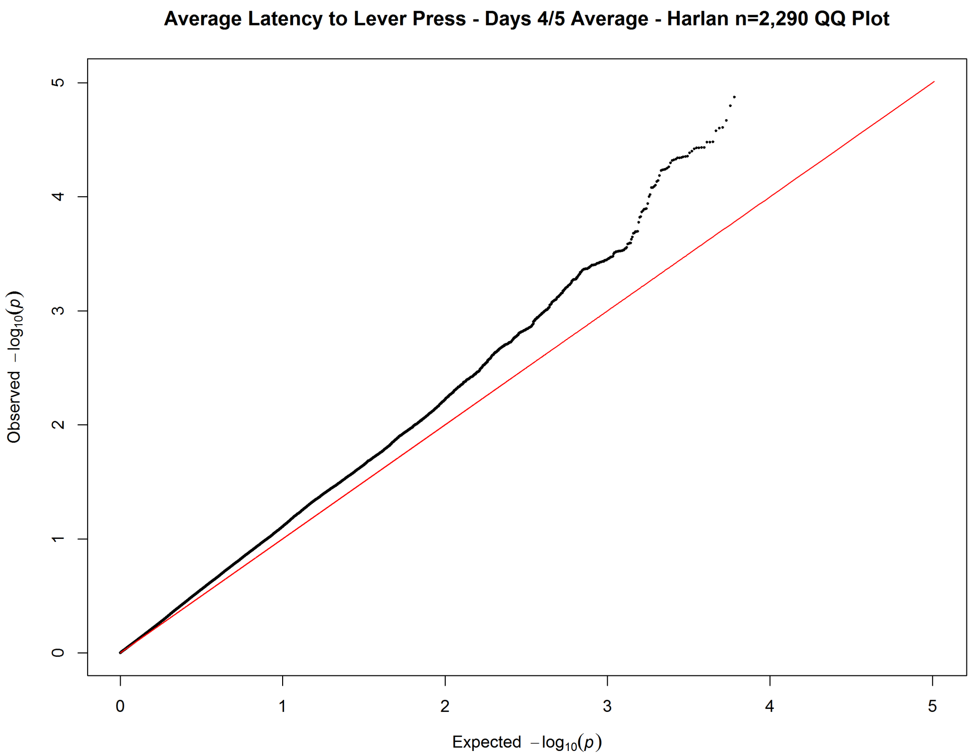

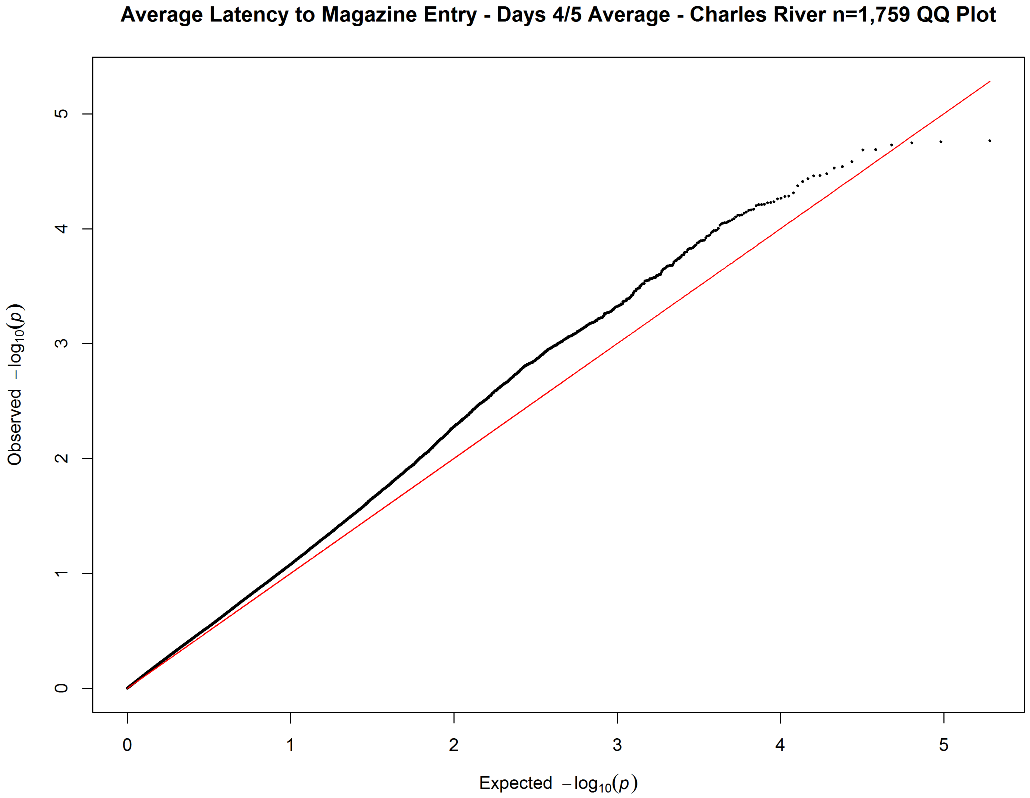

Supplement: S5 Fig — (PDF) [file pgen.1010234.s005.pdf]
