# Supplementary material for: Genetic characterization of outbred Sprague Dawley rats and utility for genome-wide association studies
Source: PLoS Genet. 2022 May 31;18(5):e1010234. doi: 10.1371/journal.pgen.1010234 (PMC9187121; doi:10.1371/journal.pgen.1010234)
Supplement: S8 Fig — Panels A and C show pre-filtering distributions of heterozygosity in Harlan and Charles River, as measured by the method-of-moments F coefficient. Panels B and D show the same distributions post-filtering. A value above 0 indicates a deflation of heterozygosity, whereas a value below 0 would be an inflation. (PDF) [file pgen.1010234.s008.pdf]

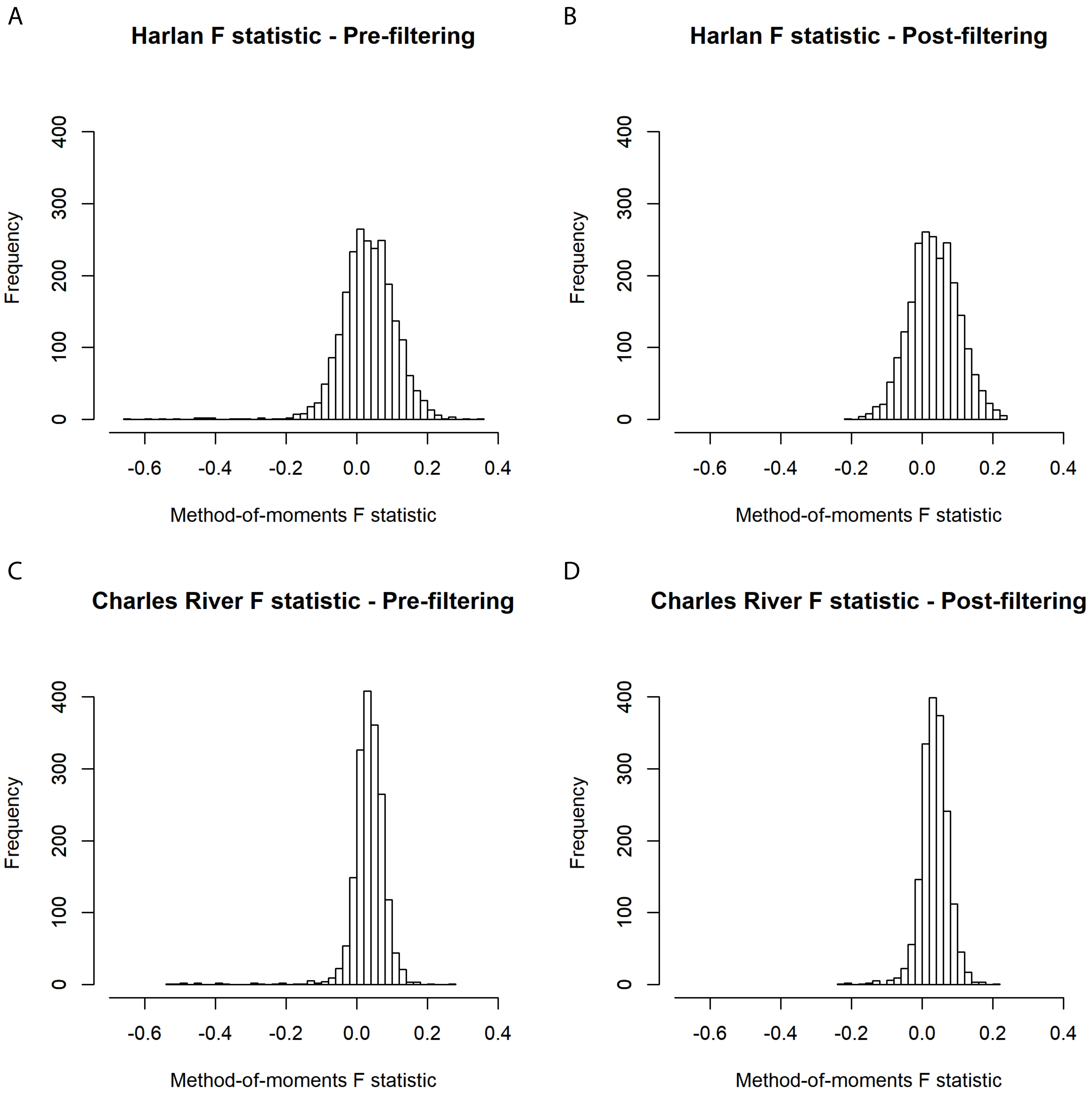
A
Harlan F statistic - Pre-filtering
Frequency
0
100
200
300
400
-0.6
-0.4
-0.2
0.0
0.2
0.4
Method-of-moments F statistic
B
Harlan F statistic - Post-filtering
Frequency
0
100
200
300
400
-0.6
-0.4
-0.2
0.0
0.2
0.4
Method-of-moments F statistic
C
Charles River F statistic - Pre-filtering
Frequency
0
100
200
300
400
-0.6
-0.4
-0.2
0.0
0.2
0.4
Method-of-moments F statistic
D
Charles River F statistic - Post-filtering
Frequency
0
100
200
300
400
-0.6
-0.4
-0.2
0.0
0.2
0.4
Method-of-moments F statistic
