# Supplementary material for: Genetic characterization of outbred Sprague Dawley rats and utility for genome-wide association studies
Source: PLoS Genet. 2022 May 31;18(5):e1010234. doi: 10.1371/journal.pgen.1010234 (PMC9187121; doi:10.1371/journal.pgen.1010234)
Supplement: S1 Table — (PDF) [file pgen.1010234.s011.pdf]

Table S1. Sample origins for all 4,061 SD rats in final filtered set

	# Samples (Barrier Facility)	# Samples (Breeding Location)
**Total samples post-filtering**	4,061	4,061
Harlan – Dublin, VA – Barrier 231	5	5
Harlan – Frederick, MD – Barrier 208A	465	465
Harlan – Haslett, MI – Barrier 206	762	762
Harlan – Houston, TX – Barrier 211	14	14
Harlan – Indianapolis, IN – Barrier 202A	546	994
Harlan – Indianapolis, IN – Barrier 202C	97	
Harlan – Indianapolis, IN – Barrier 217	351	
Charles River – Kingston, NY – Barrier K92	4	4
Charles River – Portage, MI – Barrier P03	32	558
Charles River – Portage, MI – Barrier P07	121	
Charles River – Portage, MI – Barrier P09	327	
Charles River – Portage, MI – Barrier P10	78	
Charles River – Raleigh, NC – Barrier R04	650	794
Charles River – Raleigh, NC – Barrier R09	144	
Charles River – Saint Constant, CAN – Barrier C71	46	404
Charles River – Saint Constant, CAN – Barrier C72	358	
Unknown Barrier	61	61
