# Supplementary material for: Genetic characterization of outbred Sprague Dawley rats and utility for genome-wide association studies
Source: PLoS Genet. 2022 May 31;18(5):e1010234. doi: 10.1371/journal.pgen.1010234 (PMC9187121; doi:10.1371/journal.pgen.1010234)
Supplement: S2 Table — Rates of concordance and genotyping error were calculated by comparing genotypes for 381 duplicate samples called in parallel. Each of the two replicates of the sample was assumed to contribute half the discordant genotypes. Therefore, the per sample genotyping error rate was calculated as half of the observed rate of discordance. (PDF) [file pgen.1010234.s012.pdf]

S2 Table. Concordance and error rates for ANGSD/Beagle genotype calls at different dosage r^2 thresholds

	DR^2>=0.9	DR^2>=0.8	DR^2>=0.7
Total called and imputed by ANGSD/Beagle	2,274,118	2,274,118	2,274,118
SNPs passing dosage r^2 filter	305,779	427,123	541,622
SNPs passing MAF >= 0.01 filter	244,586	326,550	393,569
SNPs passing HWE 10^{-7} filter	204,104	259,111	305,660
Mean Pearson **correlation of dosages** (n=381)	0.983	0.969	0.957
**Rate of concordance** of hard calls (n=381)	0.983	0.972	0.963
**Rate of discordance** (1 – concordance)	0.017	0.028	0.037
**Error rate** (Rate of discordance/2)*100	0.85%	1.4%	1.85%
