# Supplementary material for: Genetic characterization of outbred Sprague Dawley rats and utility for genome-wide association studies
Source: PLoS Genet. 2022 May 31;18(5):e1010234. doi: 10.1371/journal.pgen.1010234 (PMC9187121; doi:10.1371/journal.pgen.1010234)
Supplement: S3 Table — (PDF) [file pgen.1010234.s013.pdf]

S3 Table. List of filtered variant counts and sample numbers in subpopulations.

Subpopulations	Number of rats	Number of SNPs
**Harlan – All, union SNP set**	2,221	223,467
Harlan – 202A/202C/208A	1,108	197,355
Harlan – 206	762	173,443
Harlan – 217	351	171,648
**Charles River – All, union SNP set**	1,760	273,241
Charles River – R09/P03/P07/P10/C71/K92	425	256,236
Charles River – R04	650	252,798
Charles River – P09	327	253,640
Charles River – C72	358	250,868
