# Supplementary material for: Genetic characterization of outbred Sprague Dawley rats and utility for genome-wide association studies
Source: PLoS Genet. 2022 May 31;18(5):e1010234. doi: 10.1371/journal.pgen.1010234 (PMC9187121; doi:10.1371/journal.pgen.1010234)
Supplement: S4 Table — (PDF) [file pgen.1010234.s014.pdf]

S4 Table. List of sample filtering criteria and number of samples removed

	Number of Samples	
**Total sequenced by ddGBS + WGS**	4,625 (17 WGS only)	
Low read count in FASTQ (< 4 million reads)<br>**performed prior to variant calling	316	
Females samples (only females in sample set)	77	
Purchased from Taconic	4	
Lack of phenotype information	10	
Poor clustering in principal component analysis	54	
Putative sample mix-up	18	
Duplicate sample	7	
Inflated/deflated heterozygosity	34	
>= 30 samples with $\hat{\pi}$ >= 0.1875 in sample	12	
$\hat{\pi}$ > 0.6 with another sample	32	
**Final Sample Set (Harlan + Charles River)**	**4,061**	
	**CR – 1,780**	**Har – 2,281**
