# Supplementary material for: Genetic characterization of outbred Sprague Dawley rats and utility for genome-wide association studies
Source: PLoS Genet. 2022 May 31;18(5):e1010234. doi: 10.1371/journal.pgen.1010234 (PMC9187121; doi:10.1371/journal.pgen.1010234)
Supplement: S7 Table — There are 11 total metrics across 5 days of training. The first 7 metrics are direct measurements made during the training periods. The following 3 metrics are calculated from the base measurements. The final metric, PavCA index score, is a composite score from the previous 3 metrics. (PDF) [file pgen.1010234.s017.pdf]

S7 Table. List of all PavCA metrics collected on SD rats.

**Day 1** 25 8-second trials	**Day 2** 25 8-second trials	**Day 3** 25 8-second trials	**Day 4** 25 8-second trials	**Day 5** 25 8-second trials	**Data Type**
Lever Presses	Lever Presses	Lever Presses	Lever Presses	Lever Presses	Count (summed over trials)
Magazine Entries CS	Magazine Entries CS	Magazine Entries CS	Magazine Entries CS	Magazine Entries CS	Count (summed over trials)
Magazine Entries NCS	Magazine Entries NCS	Magazine Entries NCS	Magazine Entries NCS	Magazine Entries NCS	Count (summed over trials)
Latency to Lever Press	Latency to Lever Press	Latency to Lever Press	Latency to Lever Press	Latency to Lever Press	Continuous [0,8] secs (averaged over trials)
Latency to Magazine Entry	Latency to Magazine Entry	Latency to Magazine Entry	Latency to Magazine Entry	Latency to Magazine Entry	Continuous [0,8] secs (averaged over trials)
Probability of Lever Press	Probability of Lever Press	Probability of Lever Press	Probability of Lever Press	Probability of Lever Press	Proportion [0-1] (0/25 to 25/25 trials)
Probability of Mag Entry	Probability of Mag Entry	Probability of Mag Entry	Probability of Mag Entry	Probability of Mag Entry	Proportion [0-1] (0/25 to 25/25 trials)
Response Bias	Response Bias	Response Bias	Response Bias	Response Bias	Continuous [-1,1]
Latency Score	Latency Score	Latency Score	Latency Score	Latency Score	Continuous [-1,1]
Probability Difference	Probability Difference	Probability Difference	Probability Difference	Probability Difference	Continuous [-1,1]
PavCA Index Score	PavCA Index Score	PavCA Index Score	PavCA Index Score	PavCA Index Score	Continuous [-1,1]
