# Supplementary material for: Genetic characterization of outbred Sprague Dawley rats and utility for genome-wide association studies
Source: PLoS Genet. 2022 May 31;18(5):e1010234. doi: 10.1371/journal.pgen.1010234 (PMC9187121; doi:10.1371/journal.pgen.1010234)
Supplement: S8 Table — The first three covariates were used in both Charles River and Harlan analyses. The remaining covariates were unique to each population. (PDF) [file pgen.1010234.s018.pdf]

S8 Table – List of covariates used for the GWAS LMMs in the seven subpopulation clusters

All sets of initials in the table below indicate experimenters that worked on testing the rats in this study for PavCA. The covariates were utilized as binary indicator variables with only one experiment or combination of experimenters being responsible for testing a given rat.

Har 202A	Har 206	Har 217	CR R04	CR R09	CR P09	CR C72
Age of rat in days at start of training (continuous integer)						
Housing condition (binary – single or multiple rats per chamber)						
Light cycle (binary – standard or reverse 12h lighting)						
AAK	CJF	AK	AAK	CJF	AK	AAK
BFS.AA	JC.JDM	BFS	CJF	CJF.JDM	BFS.AA	BFS
CJF	JJ	BTS	CJF.JDM	EGO	EGO	BFS.AA
CJF.JDM	KP	CJF	JJ		LMY	CJF.JDM
EGO		CJF.JDM			MLR.LMF	
KP		JC.JDM				
LMY		TW.KP				
