# Supplementary material for: Genetic characterization of outbred Sprague Dawley rats and utility for genome-wide association studies
Source: PLoS Genet. 2022 May 31;18(5):e1010234. doi: 10.1371/journal.pgen.1010234 (PMC9187121; doi:10.1371/journal.pgen.1010234)

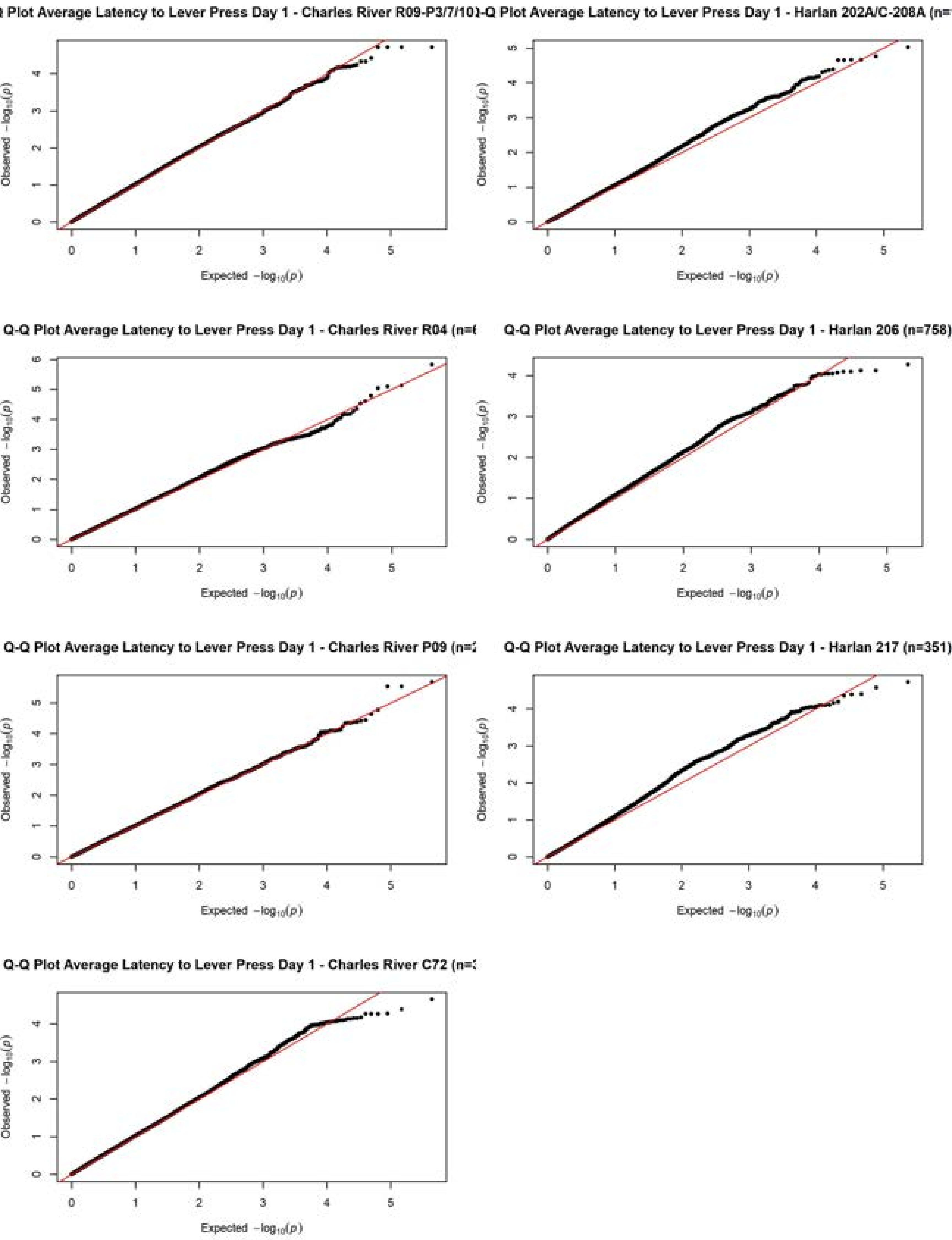
Plot Average Latency to Lever Press Day 1 - Charles River R09-P3/7/10
Q-Q Plot Average Latency to Lever Press Day 1 - Harlan 202A/C-208A (n=
Q-Q Plot Average Latency to Lever Press Day 1 - Charles River R04 (n=
Q-Q Plot Average Latency to Lever Press Day 1 - Harlan 206 (n=758)
Q-Q Plot Average Latency to Lever Press Day 1 - Charles River P09 (n=
Q-Q Plot Average Latency to Lever Press Day 1 - Harlan 217 (n=351)
Q-Q Plot Average Latency to Lever Press Day 1 - Charles River C72 (n=
Expected $-\log_{10}(p)$
Observed $-\log_{10}(p)$

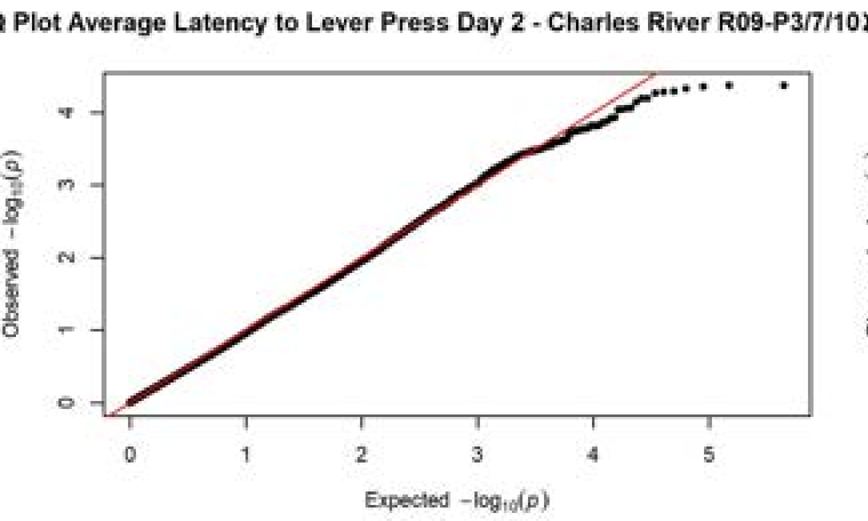
Q Plot Average Latency to Lever Press Day 2 - Charles River R09-P3/7/10
Observed −log10(p)
Expected −log10(p)

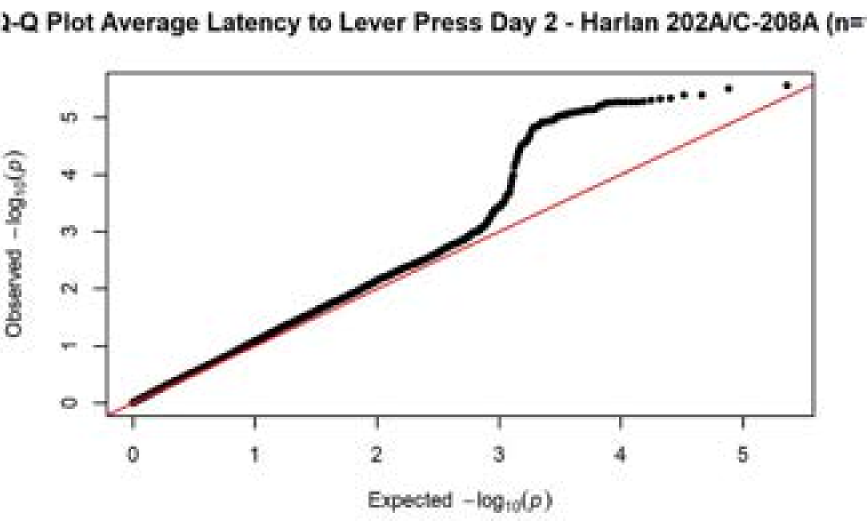
Q-Q Plot Average Latency to Lever Press Day 2 - Harlan 202A/C-208A (n=
Observed −log10(p)
Expected −log10(p)

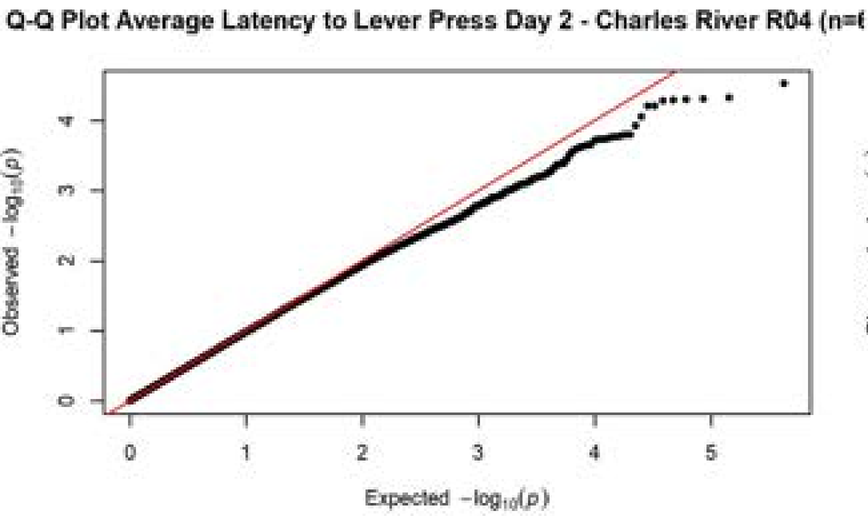
Q-Q Plot Average Latency to Lever Press Day 2 - Charles River R04 (n=
Observed −log10(p)
Expected −log10(p)

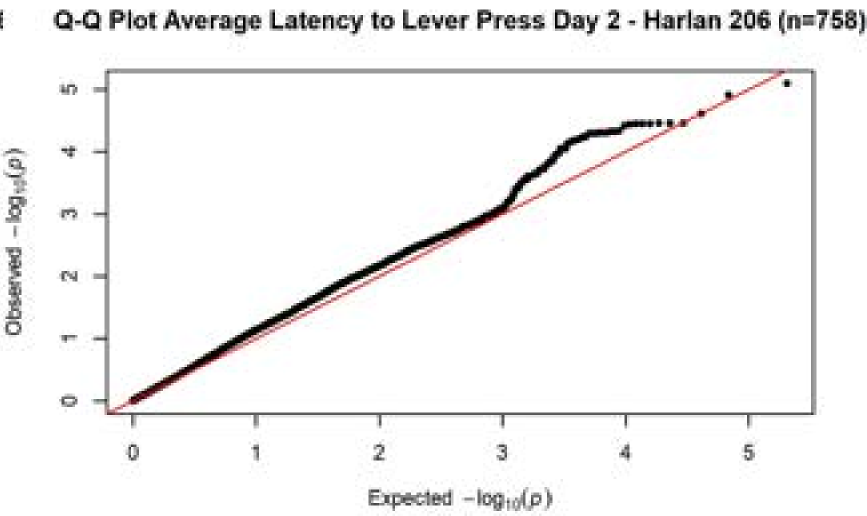
Q-Q Plot Average Latency to Lever Press Day 2 - Harlan 206 (n=758)
Observed −log10(p)
Expected −log10(p)

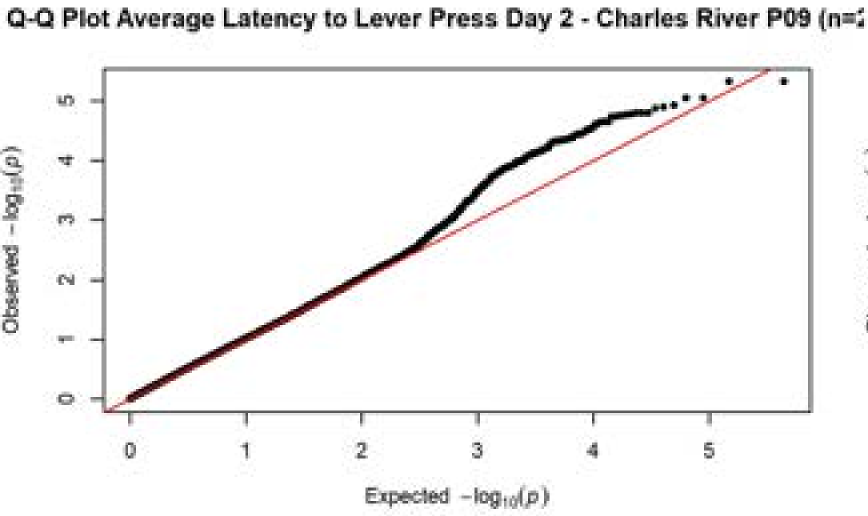
Q-Q Plot Average Latency to Lever Press Day 2 - Charles River P09 (n=
Observed −log10(p)
Expected −log10(p)

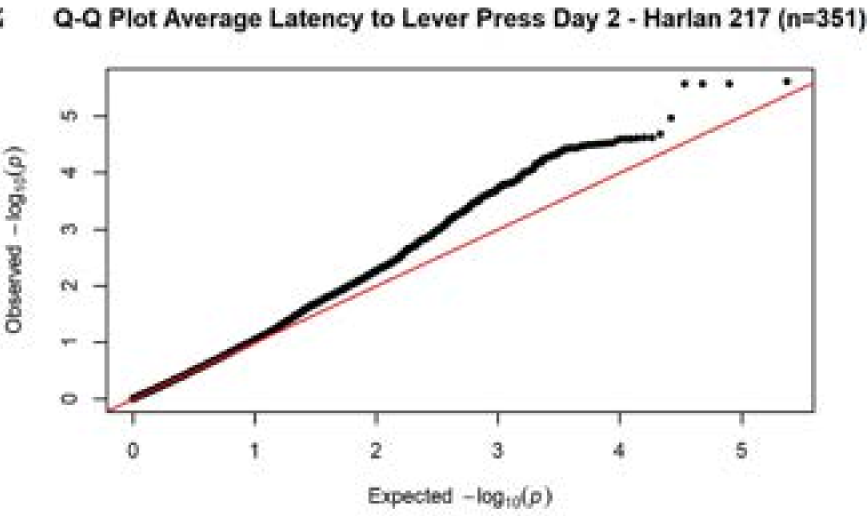
Q-Q Plot Average Latency to Lever Press Day 2 - Harlan 217 (n=351)
Observed −log10(p)
Expected −log10(p)

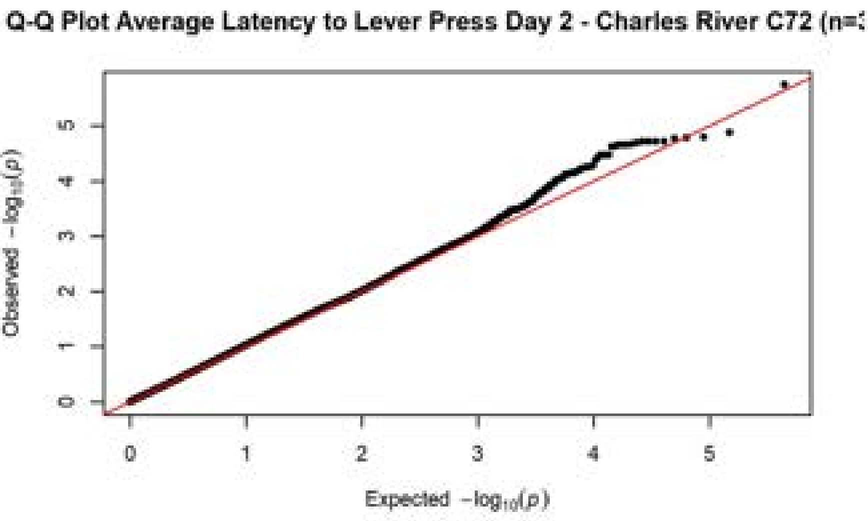
Q-Q Plot Average Latency to Lever Press Day 2 - Charles River C72 (n=
Observed −log10(p)
Expected −log10(p)

Q Plot Average Latency to Lever Press Day 3 - Charles River R09-P3/7/10

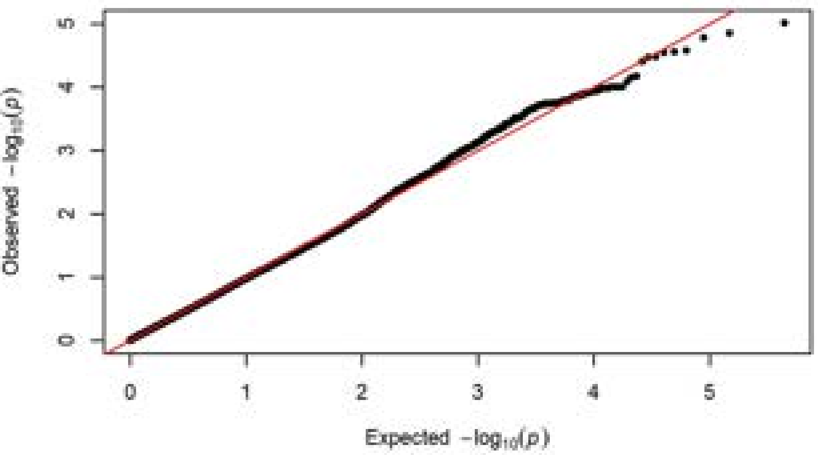

Q-Q Plot Average Latency to Lever Press Day 3 - Harlan 202A/C-208A (n=

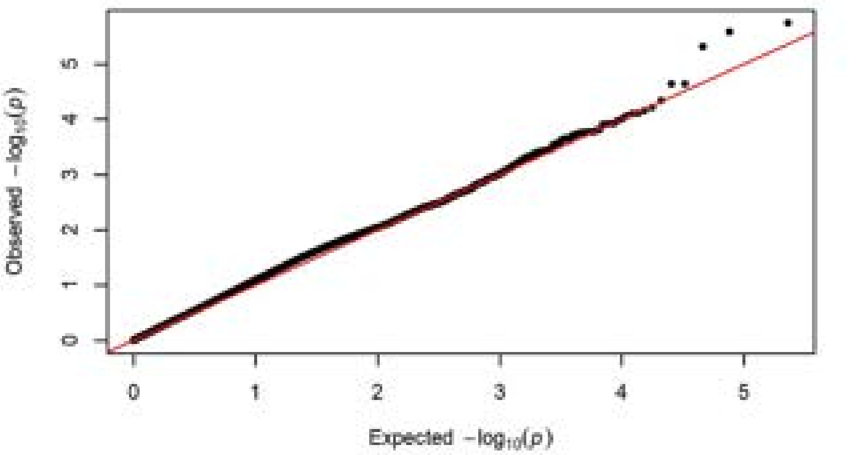

Q-Q Plot Average Latency to Lever Press Day 3 - Charles River R04 (n=

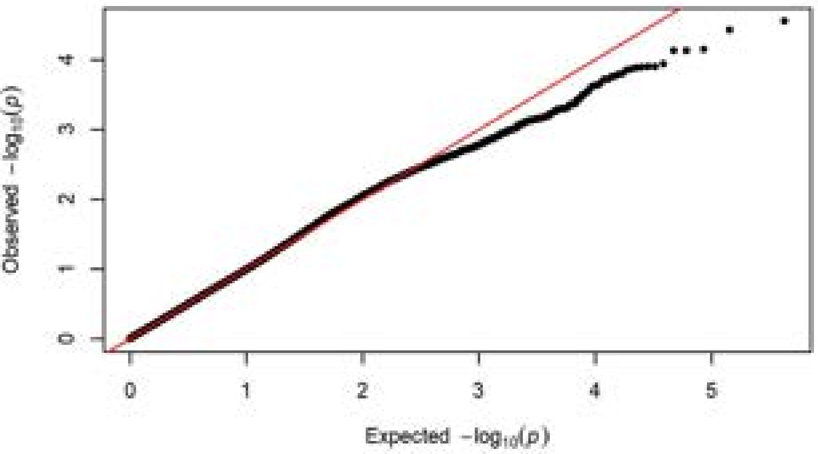

Q-Q Plot Average Latency to Lever Press Day 3 - Harlan 206 (n=758)

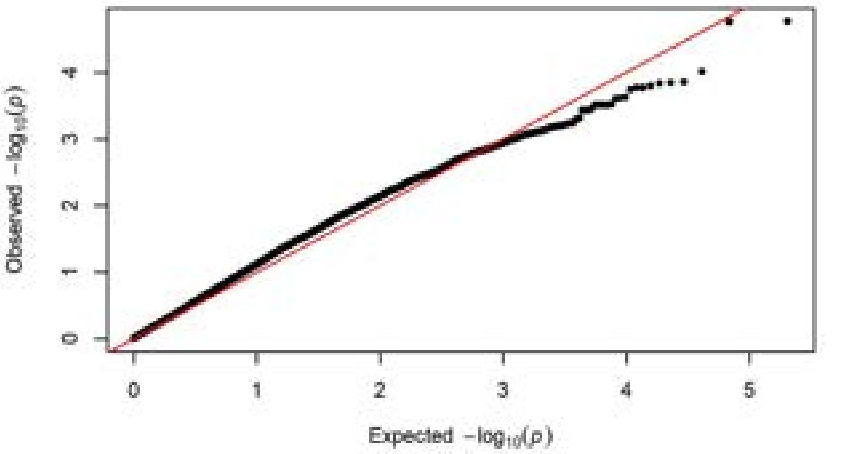

Q-Q Plot Average Latency to Lever Press Day 3 - Charles River P09 (n=

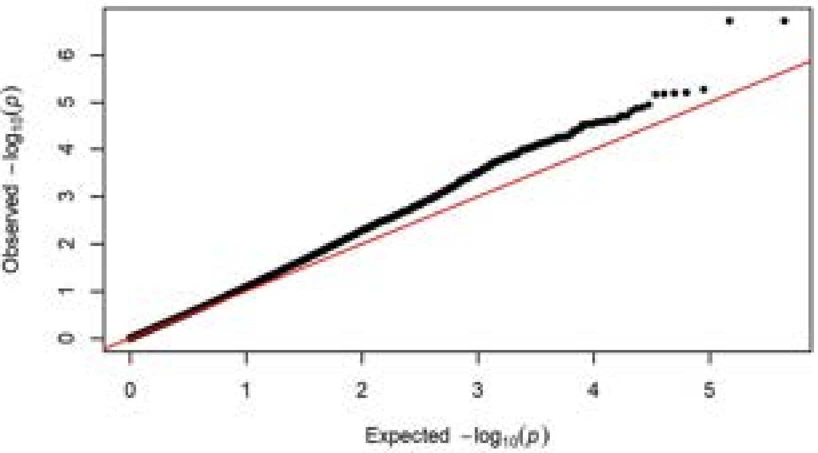

Q-Q Plot Average Latency to Lever Press Day 3 - Harlan 217 (n=351)

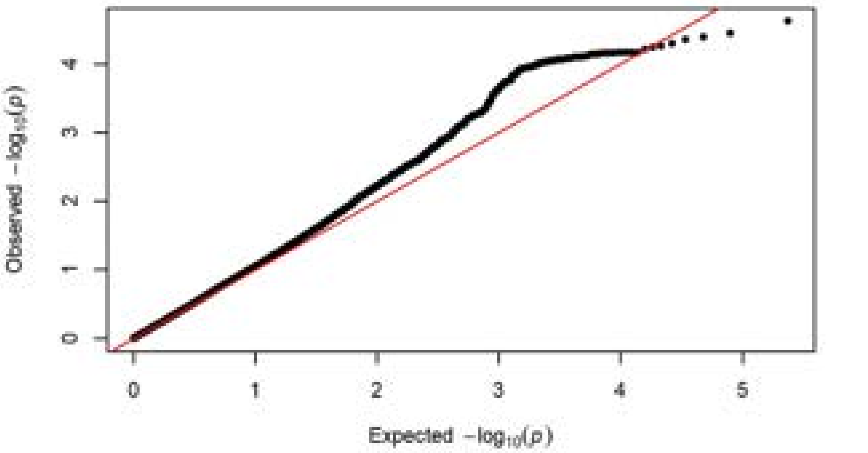

Q-Q Plot Average Latency to Lever Press Day 3 - Charles River C72 (n=

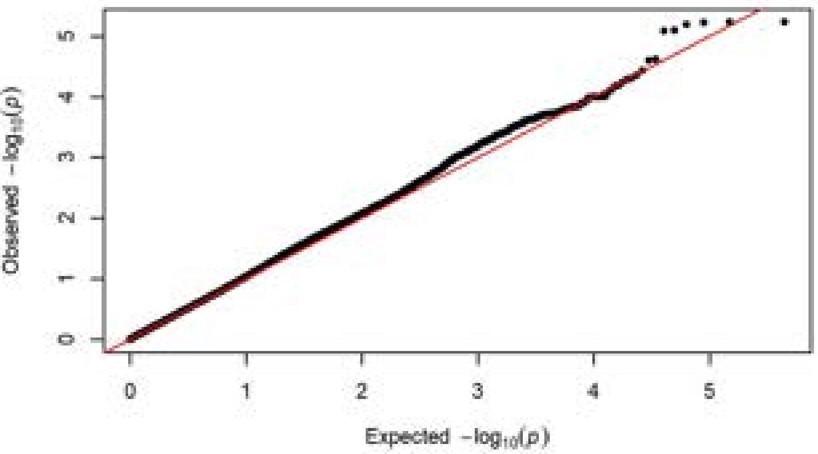

Q Plot Average Latency to Lever Press Day 4 - Charles River R09-P3/7/10

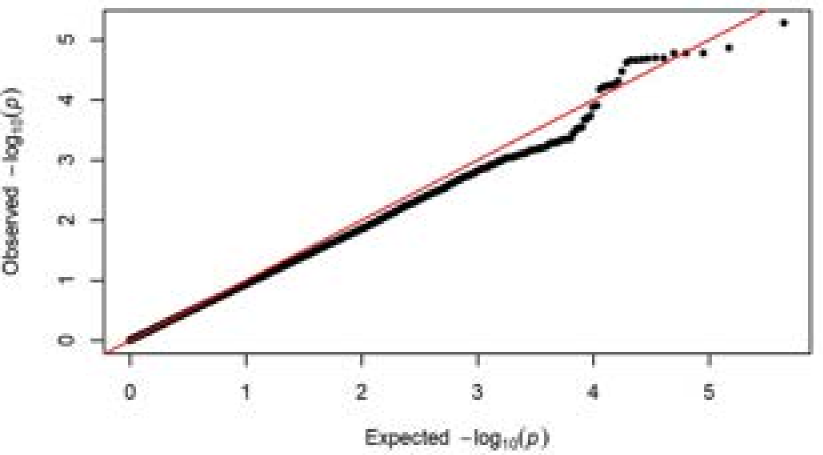

Q-Q Plot Average Latency to Lever Press Day 4 - Harlan 202A/C-208A (n=

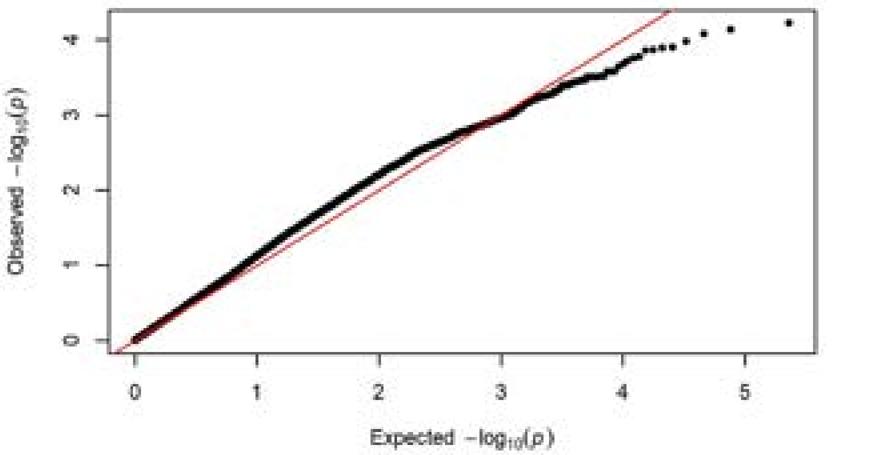

Q-Q Plot Average Latency to Lever Press Day 4 - Charles River R04 (n=

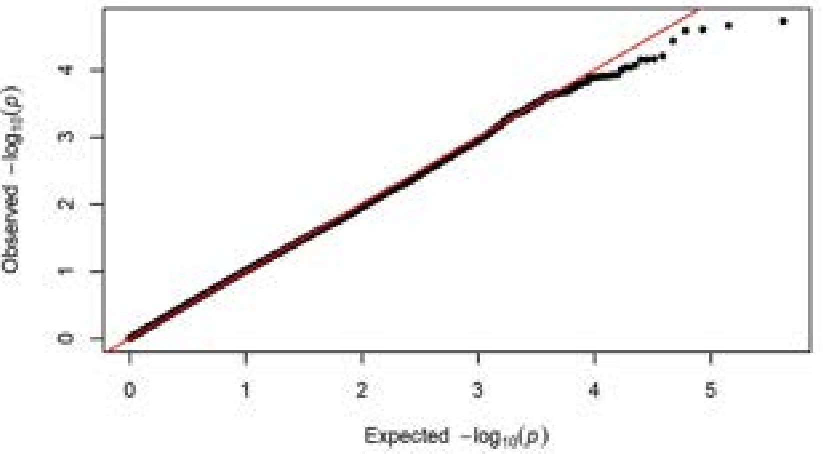

Q-Q Plot Average Latency to Lever Press Day 4 - Harlan 206 (n=758)

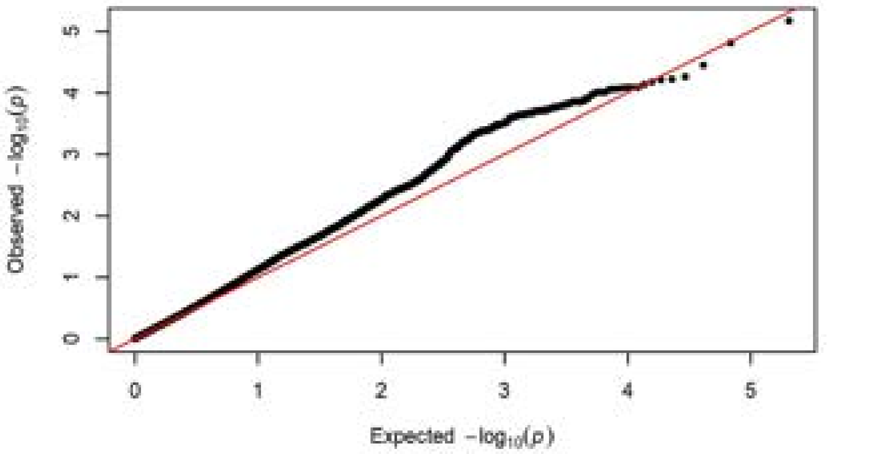

Q-Q Plot Average Latency to Lever Press Day 4 - Charles River P09 (n=

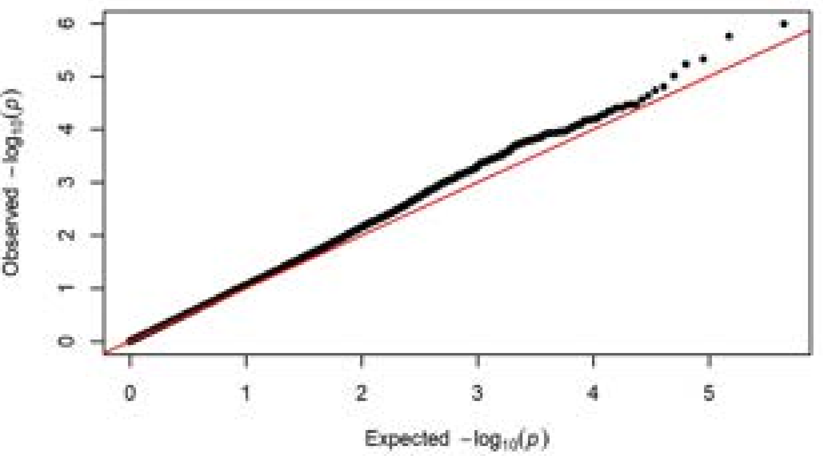

Q-Q Plot Average Latency to Lever Press Day 4 - Harlan 217 (n=351)

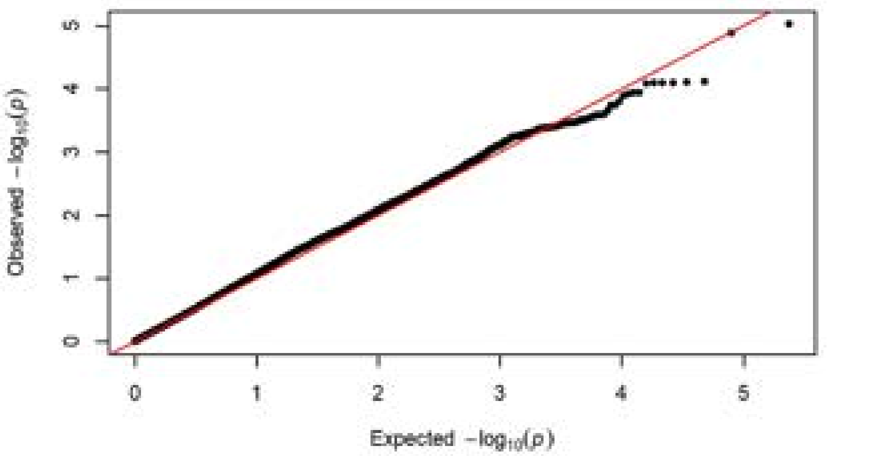

Q-Q Plot Average Latency to Lever Press Day 4 - Charles River C72 (n=

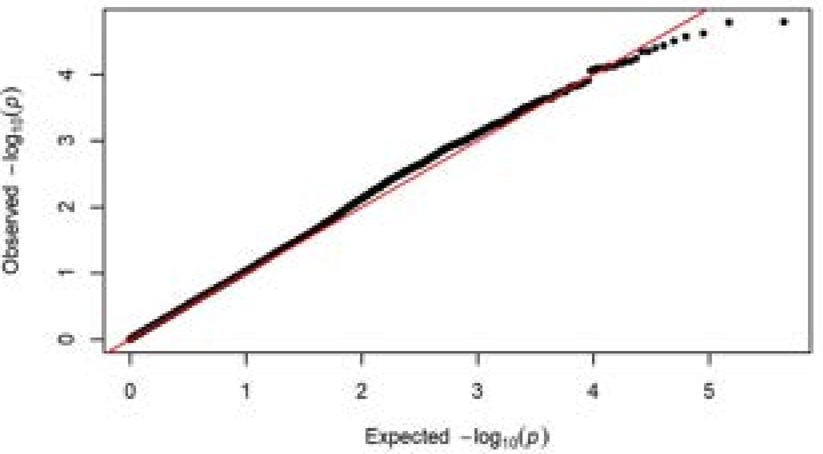

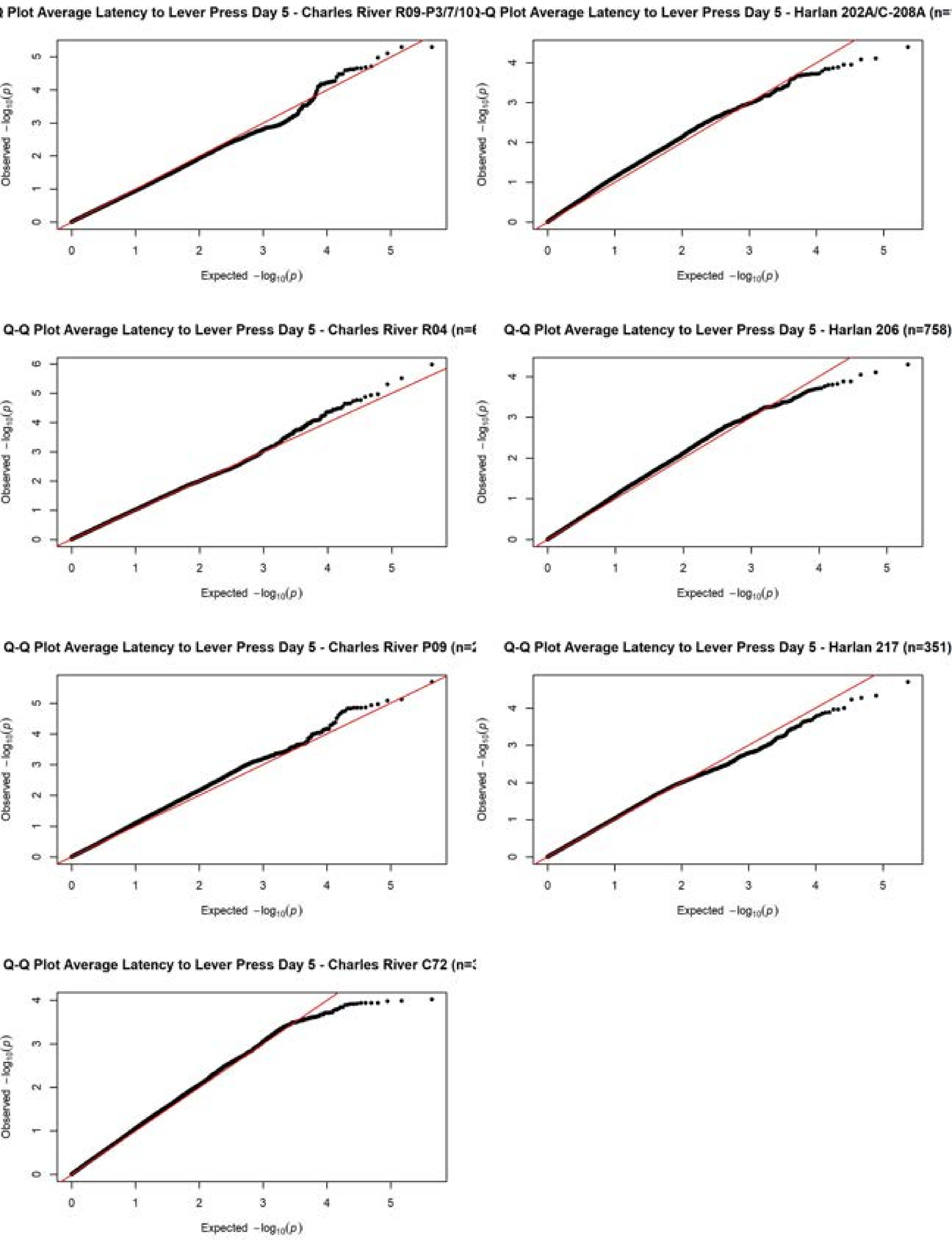
Plot Average Latency to Lever Press Day 5 - Charles River R09-P3/7/10
Q-Q Plot Average Latency to Lever Press Day 5 - Harlan 202A/C-208A (n=
Q-Q Plot Average Latency to Lever Press Day 5 - Charles River R04 (n=
Q-Q Plot Average Latency to Lever Press Day 5 - Harlan 206 (n=758)
Q-Q Plot Average Latency to Lever Press Day 5 - Charles River P09 (n=
Q-Q Plot Average Latency to Lever Press Day 5 - Harlan 217 (n=351)
Q-Q Plot Average Latency to Lever Press Day 5 - Charles River C72 (n=
Observed $-\log_{10}(p)$
Expected $-\log_{10}(p)$

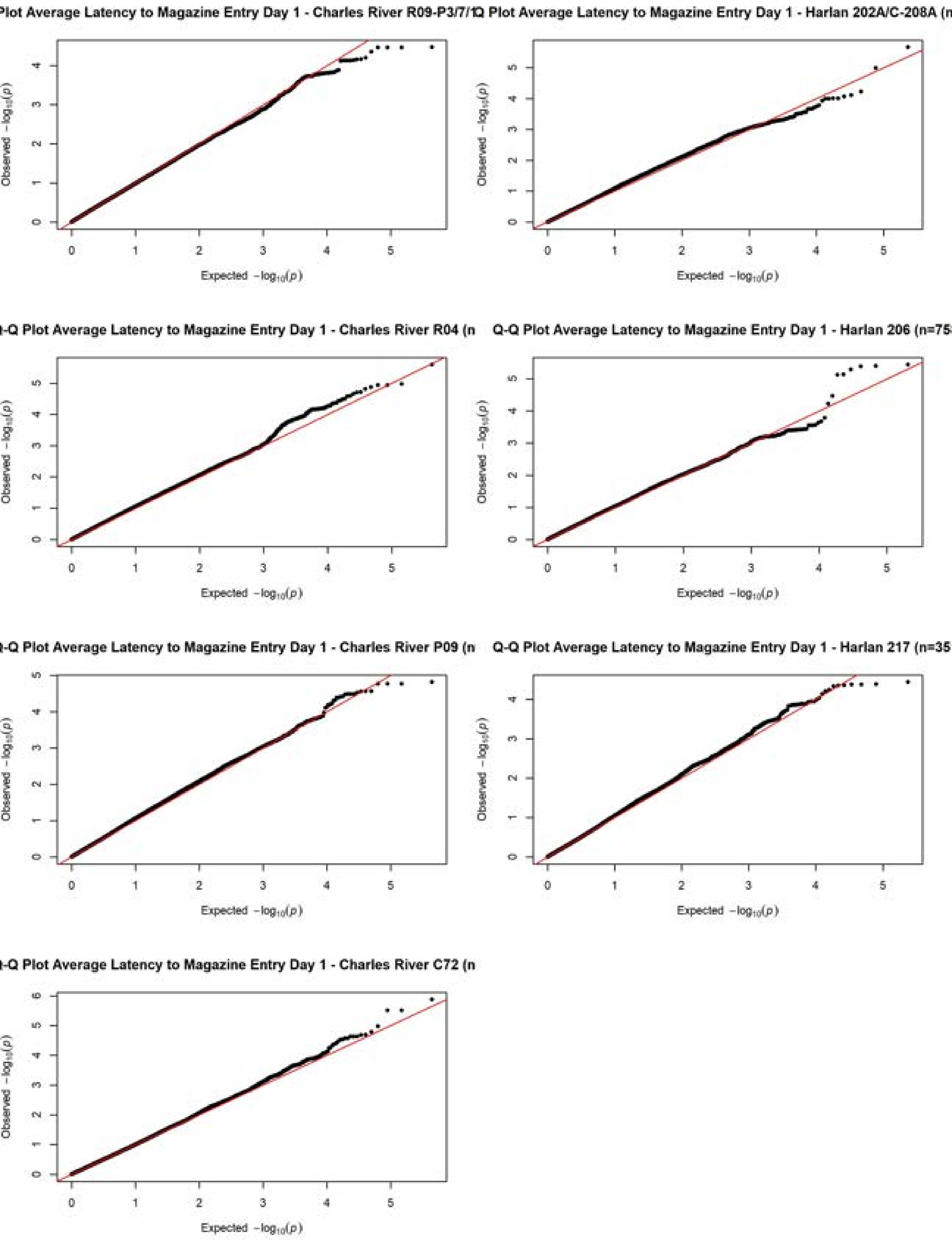

Plot Average Latency to Magazine Entry Day 1 - Charles River R09-P3/7/1Q Plot Average Latency to Magazine Entry Day 1 - Harlan 202A/C-208A (n
-Q Plot Average Latency to Magazine Entry Day 1 - Charles River R04 (n
Q-Q Plot Average Latency to Magazine Entry Day 1 - Harlan 206 (n=75
-Q Plot Average Latency to Magazine Entry Day 1 - Charles River P09 (n
Q-Q Plot Average Latency to Magazine Entry Day 1 - Harlan 217 (n=35
-Q Plot Average Latency to Magazine Entry Day 1 - Charles River C72 (n
Observed $-\log_{10}(p)$
Expected $-\log_{10}(p)$

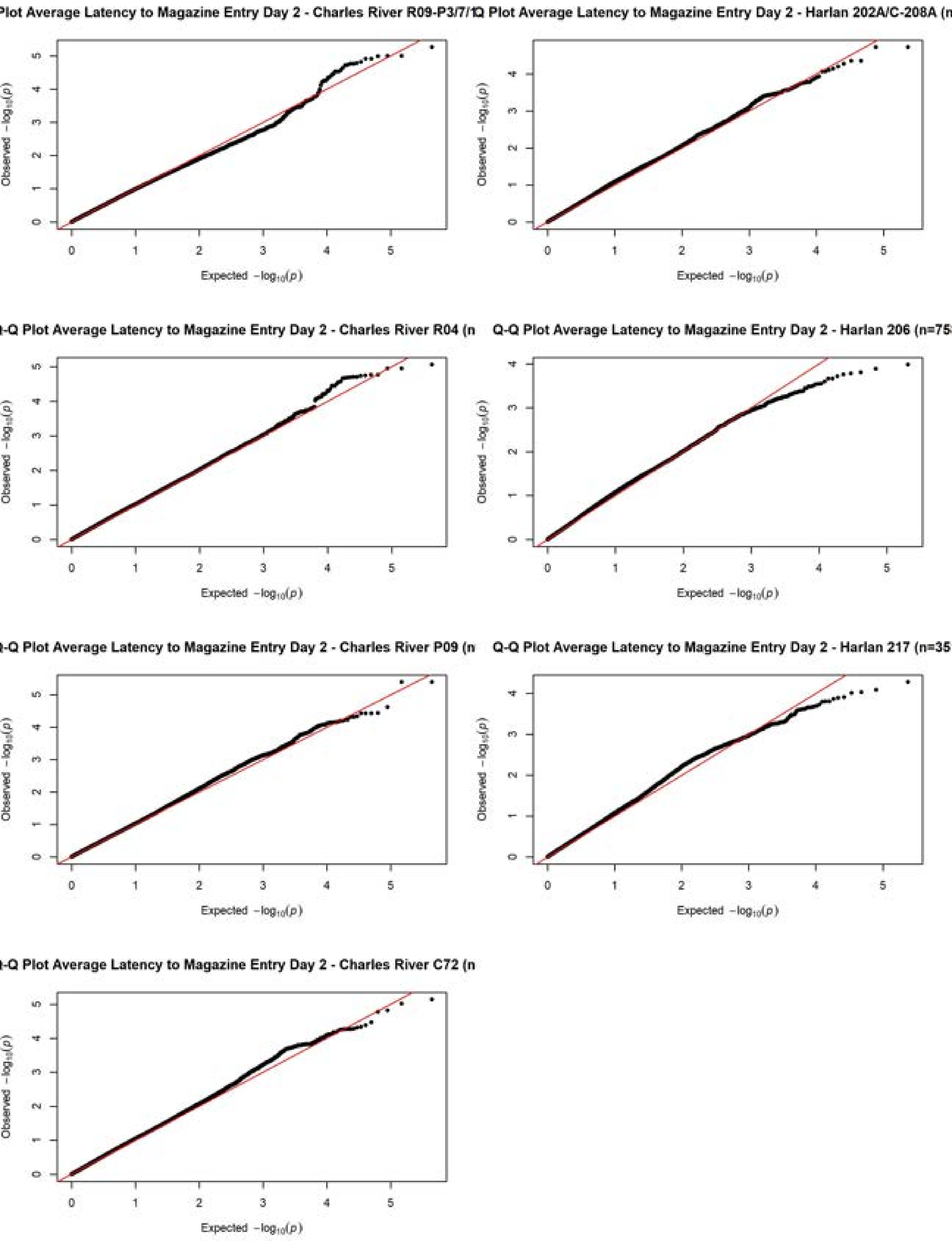
Plot Average Latency to Magazine Entry Day 2 - Charles River R09-P3/7/1
Q Plot Average Latency to Magazine Entry Day 2 - Harlan 202A/C-208A (n
Q-Q Plot Average Latency to Magazine Entry Day 2 - Charles River R04 (n
Q-Q Plot Average Latency to Magazine Entry Day 2 - Harlan 206 (n=75
Q-Q Plot Average Latency to Magazine Entry Day 2 - Charles River P09 (n
Q-Q Plot Average Latency to Magazine Entry Day 2 - Harlan 217 (n=35
Q-Q Plot Average Latency to Magazine Entry Day 2 - Charles River C72 (n
Observed $-\log_{10}(p)$
Expected $-\log_{10}(p)$

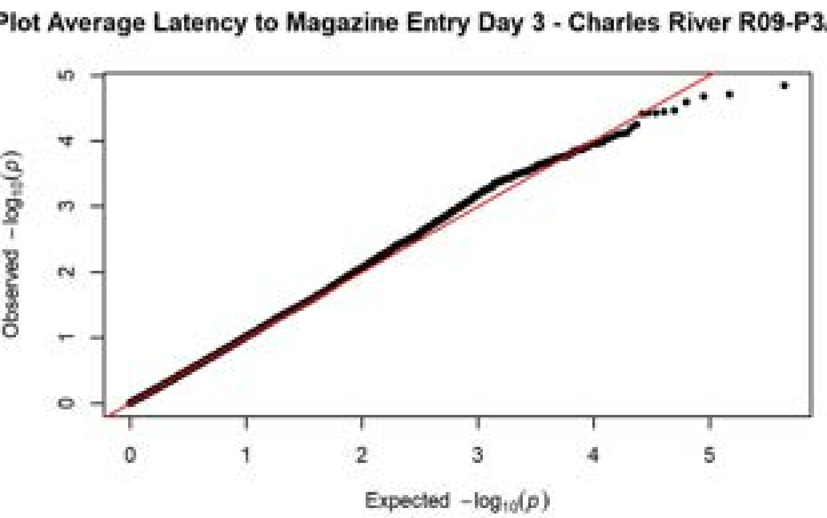
Plot Average Latency to Magazine Entry Day 3 - Charles River R09-P3/7/1
Observed −log10(p)
Expected −log10(p)

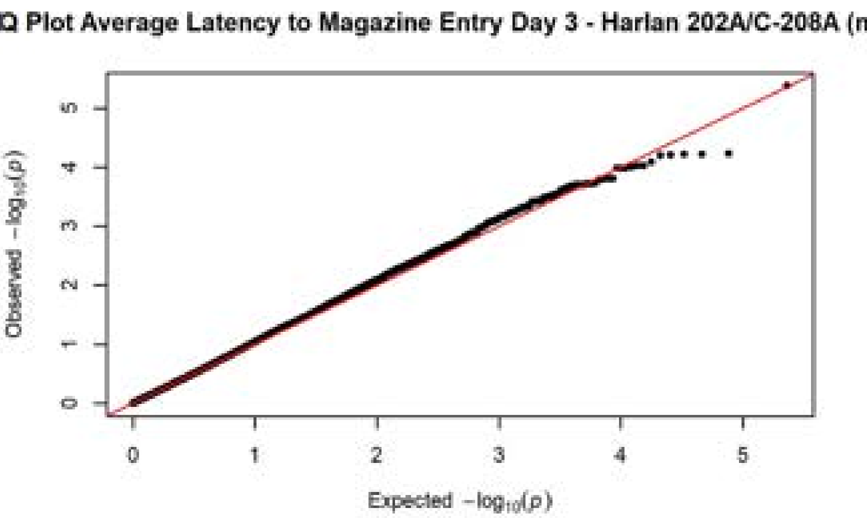
Q Plot Average Latency to Magazine Entry Day 3 - Harlan 202A/C-208A (n
Observed −log10(p)
Expected −log10(p)

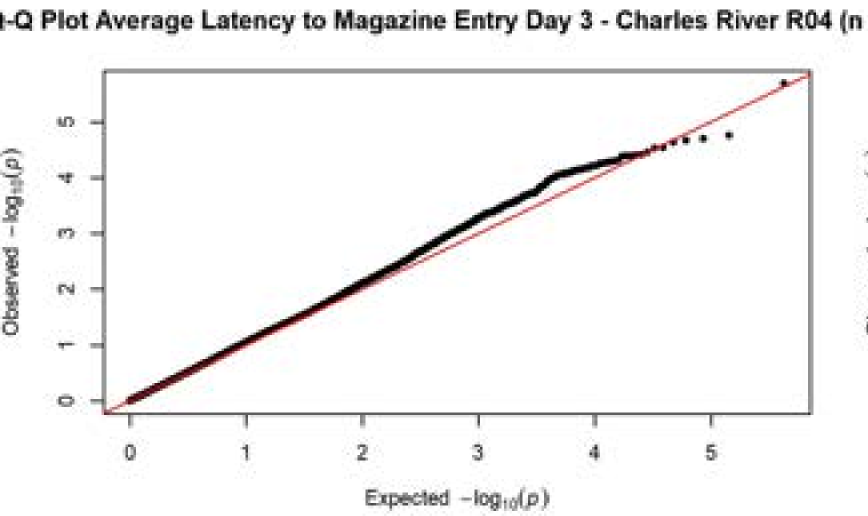
-Q Plot Average Latency to Magazine Entry Day 3 - Charles River R04 (n
Observed −log10(p)
Expected −log10(p)

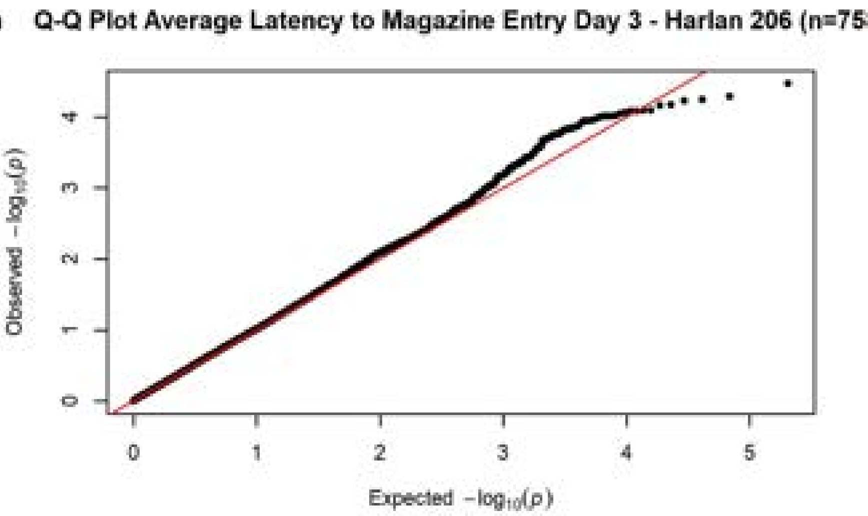
Q-Q Plot Average Latency to Magazine Entry Day 3 - Harlan 206 (n=75
Observed −log10(p)
Expected −log10(p)

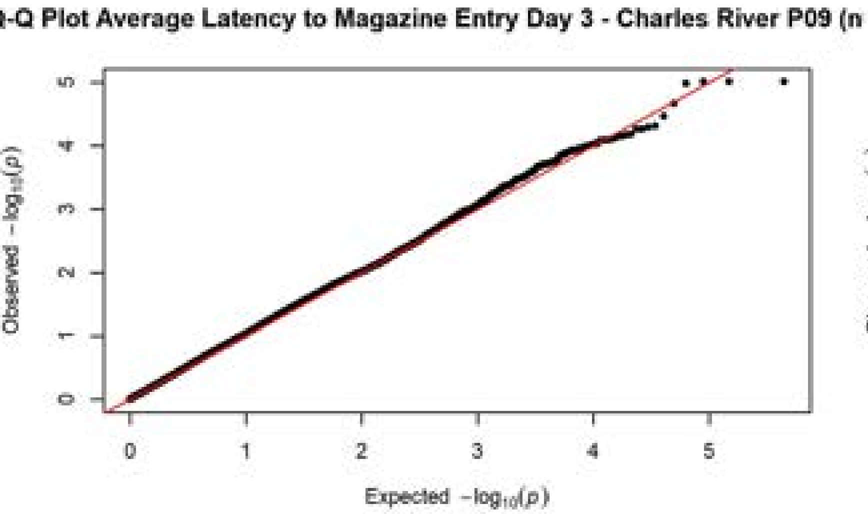
-Q Plot Average Latency to Magazine Entry Day 3 - Charles River P09 (n
Observed −log10(p)
Expected −log10(p)

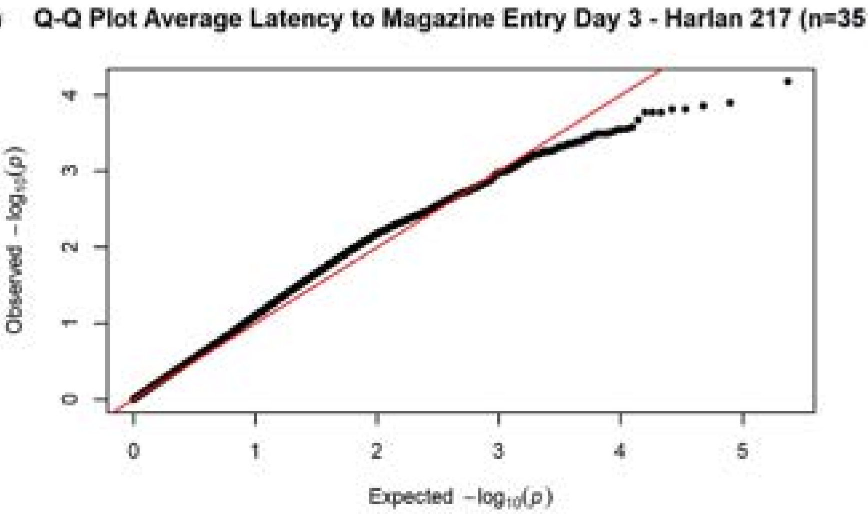
Q-Q Plot Average Latency to Magazine Entry Day 3 - Harlan 217 (n=35
Observed −log10(p)
Expected −log10(p)

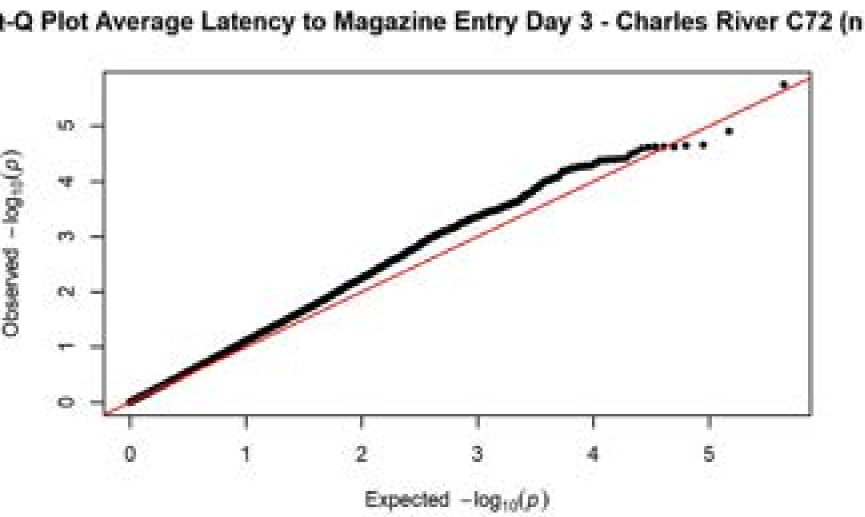
-Q Plot Average Latency to Magazine Entry Day 3 - Charles River C72 (n
Observed −log10(p)
Expected −log10(p)

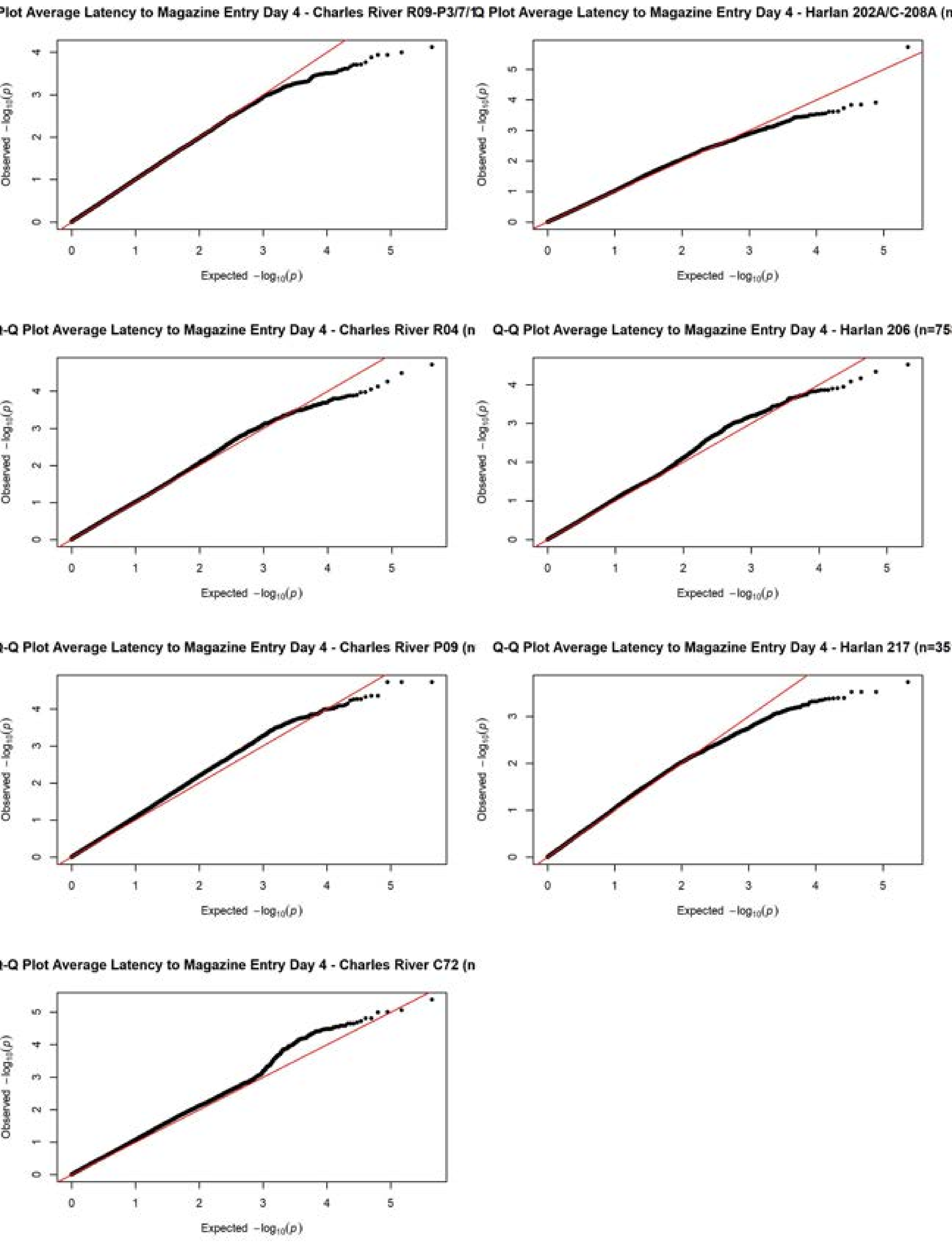
Plot Average Latency to Magazine Entry Day 4 - Charles River R09-P3/7/1Q
Observed −log10(p)
Expected −log10(p)
Plot Average Latency to Magazine Entry Day 4 - Harlan 202A/C-208A (n
Observed −log10(p)
Expected −log10(p)
-Q Plot Average Latency to Magazine Entry Day 4 - Charles River R04 (n
Observed −log10(p)
Expected −log10(p)
Q-Q Plot Average Latency to Magazine Entry Day 4 - Harlan 206 (n=75
Observed −log10(p)
Expected −log10(p)
-Q Plot Average Latency to Magazine Entry Day 4 - Charles River P09 (n
Observed −log10(p)
Expected −log10(p)
Q-Q Plot Average Latency to Magazine Entry Day 4 - Harlan 217 (n=35
Observed −log10(p)
Expected −log10(p)
-Q Plot Average Latency to Magazine Entry Day 4 - Charles River C72 (n
Observed −log10(p)
Expected −log10(p)

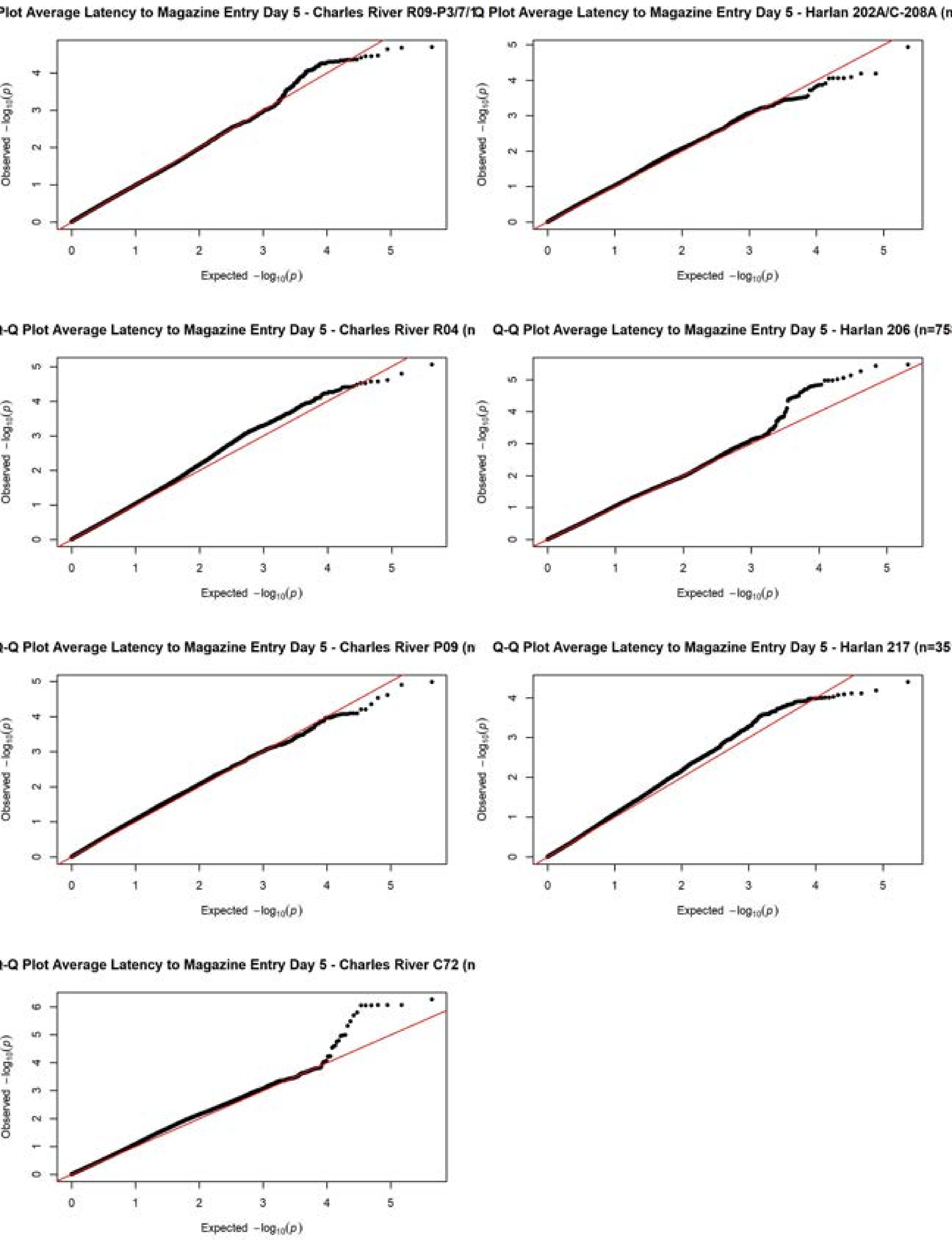
Plot Average Latency to Magazine Entry Day 5 - Charles River R09-P3/7/1
Q Plot Average Latency to Magazine Entry Day 5 - Harlan 202A/C-208A (n
-Q Plot Average Latency to Magazine Entry Day 5 - Charles River R04 (n
Q-Q Plot Average Latency to Magazine Entry Day 5 - Harlan 206 (n=75
-Q Plot Average Latency to Magazine Entry Day 5 - Charles River P09 (n
Q-Q Plot Average Latency to Magazine Entry Day 5 - Harlan 217 (n=35
-Q Plot Average Latency to Magazine Entry Day 5 - Charles River C72 (n
Observed $-\log_{10}(p)$
Expected $-\log_{10}(p)$

Q-Q Plot PavCA Index Score Day 1 - Charles River R09-P3/7/10 (n=42[illegible]

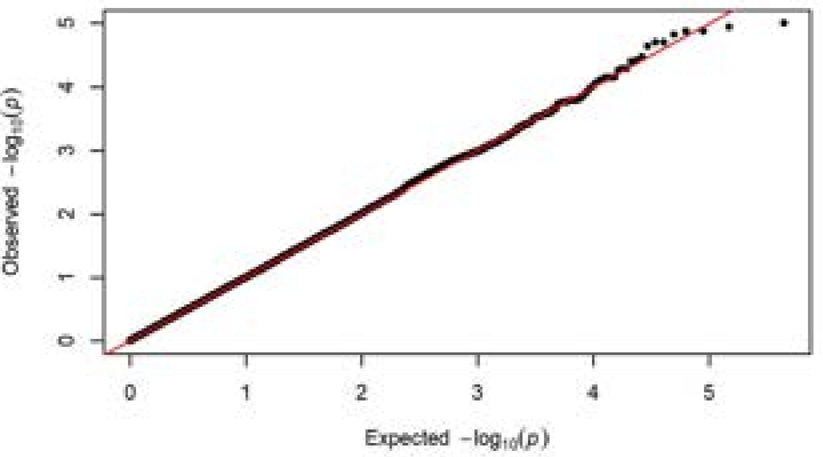

Q-Q Plot PavCA Index Score Day 1 - Harlan 202A/C-208A (n=1062)

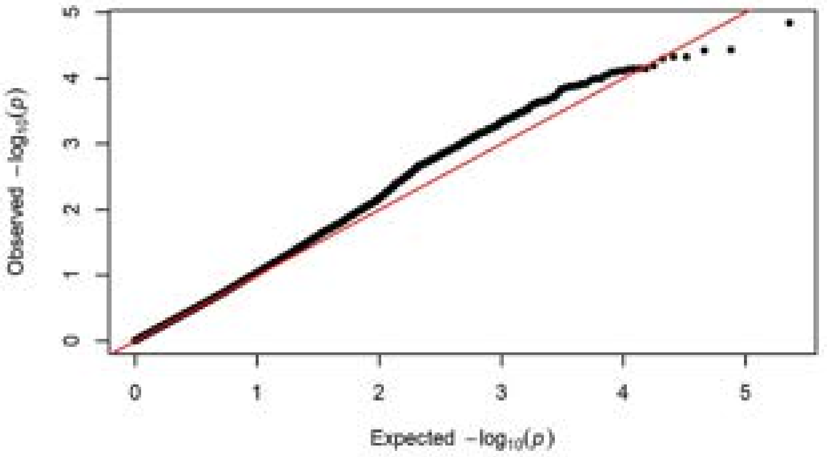

Q-Q Plot PavCA Index Score Day 1 - Charles River R04 (n=648)

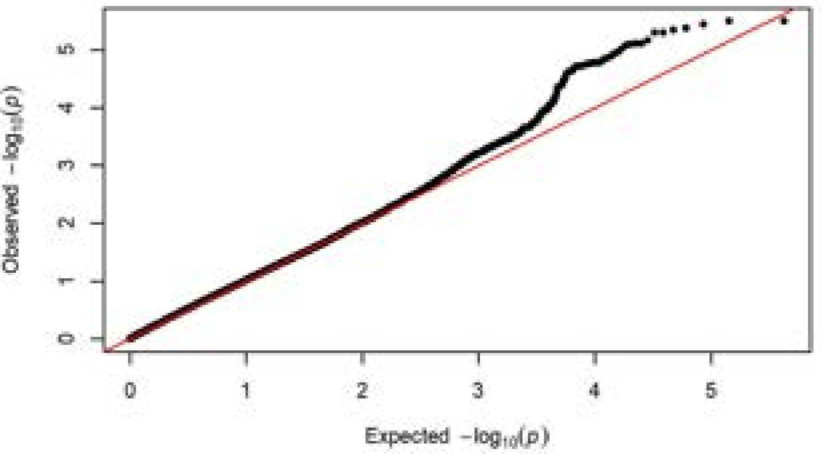

Q-Q Plot PavCA Index Score Day 1 - Harlan 206 (n=752)

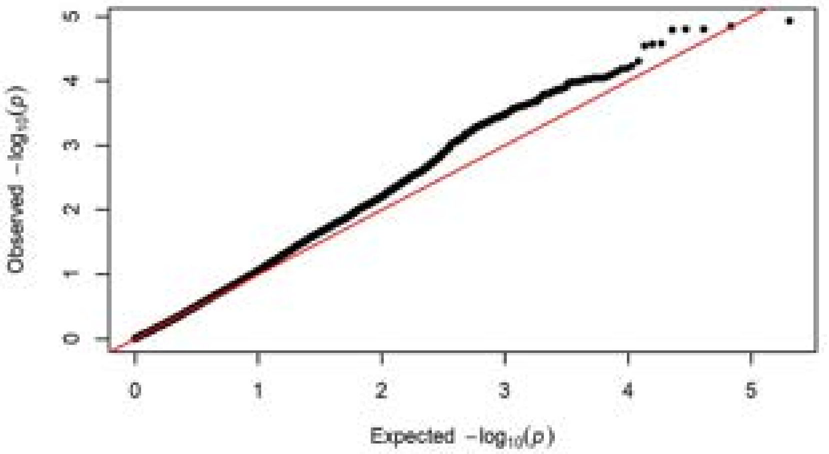

Q-Q Plot PavCA Index Score Day 1 - Charles River P09 (n=293)

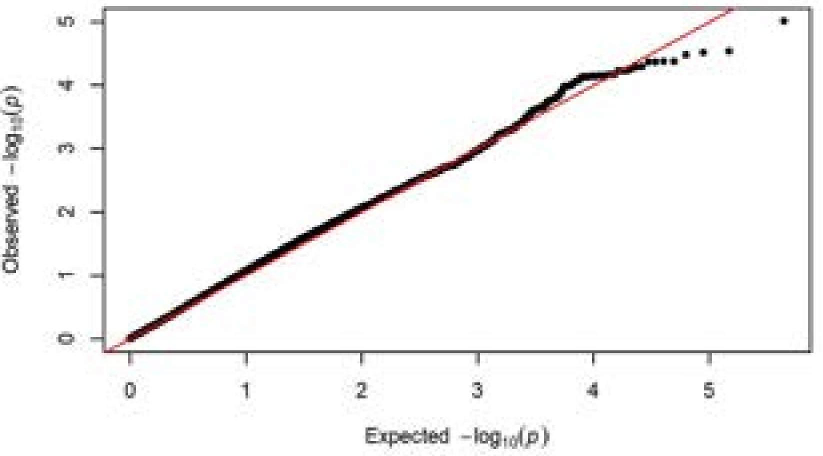

Q-Q Plot PavCA Index Score Day 1 - Harlan 217 (n=346)

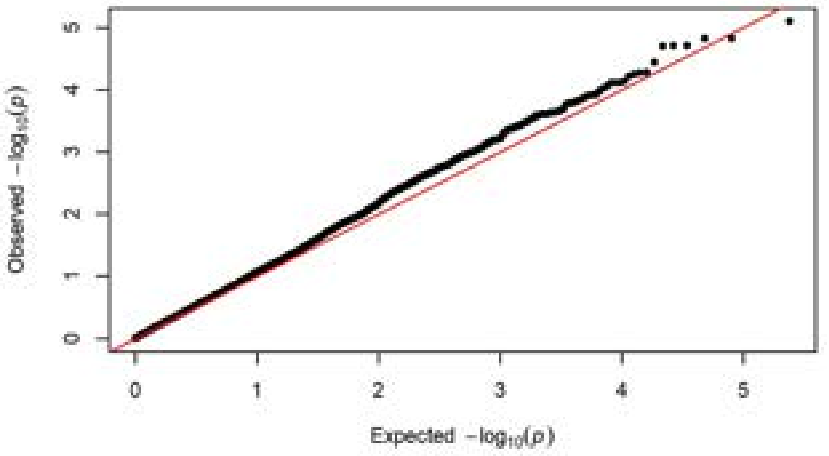

Q-Q Plot PavCA Index Score Day 1 - Charles River C72 (n=357)

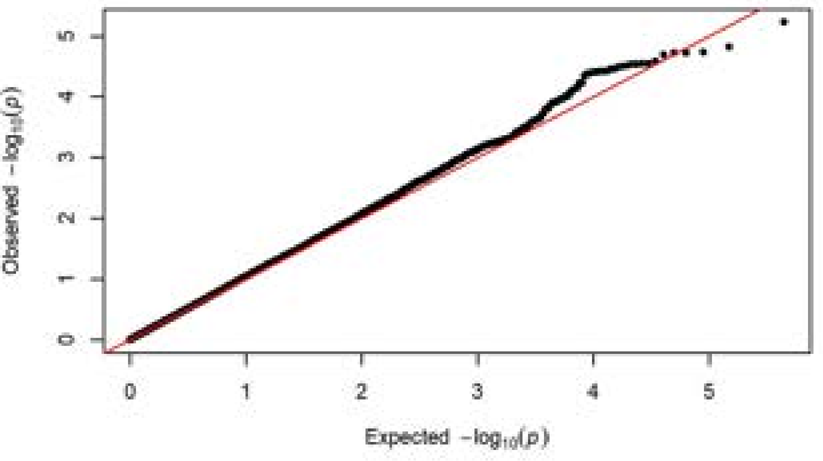

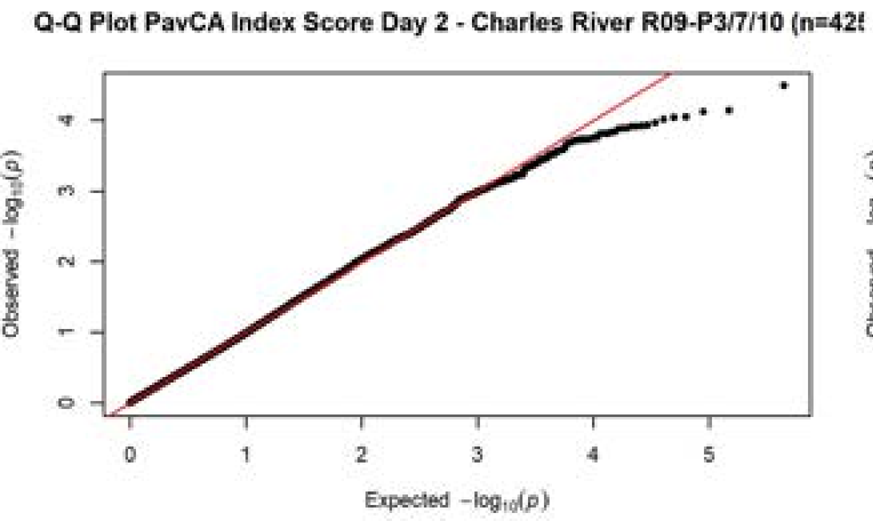
Q-Q Plot PavCA Index Score Day 2 - Charles River R09-P3/7/10 (n=42
Observed $-\log_{10}(p)$
Expected $-\log_{10}(p)$

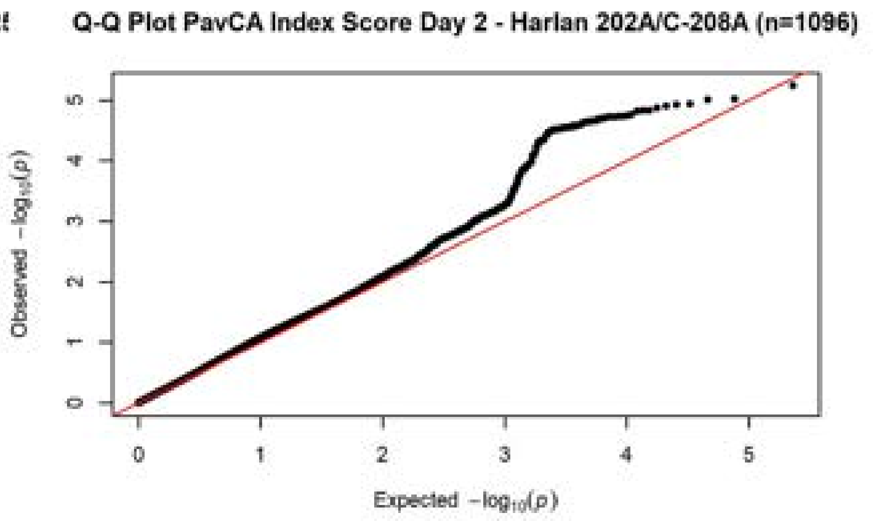
Q-Q Plot PavCA Index Score Day 2 - Harlan 202A/C-208A (n=1096)
Observed $-\log_{10}(p)$
Expected $-\log_{10}(p)$

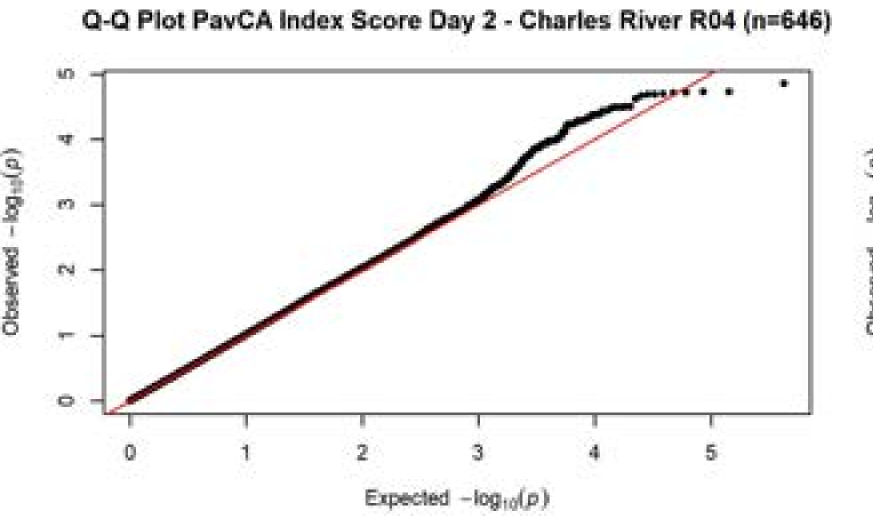
Q-Q Plot PavCA Index Score Day 2 - Charles River R04 (n=646)
Observed $-\log_{10}(p)$
Expected $-\log_{10}(p)$

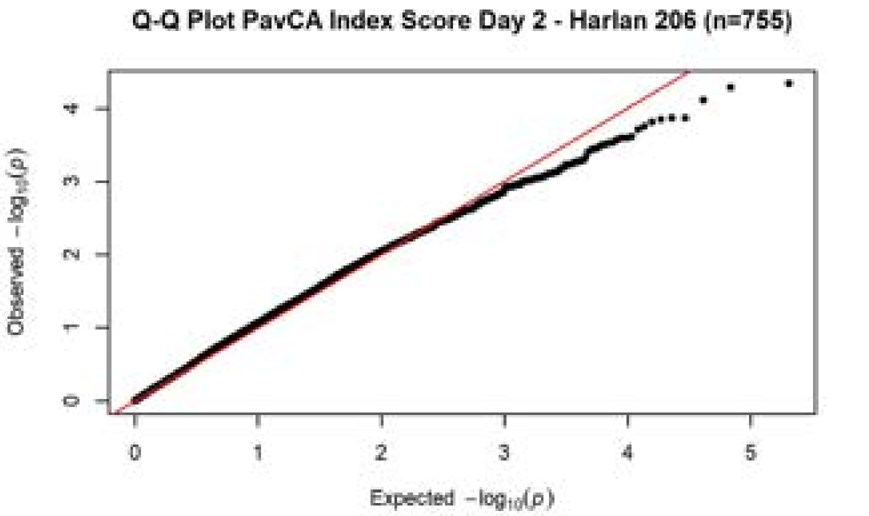
Q-Q Plot PavCA Index Score Day 2 - Harlan 206 (n=755)
Observed $-\log_{10}(p)$
Expected $-\log_{10}(p)$

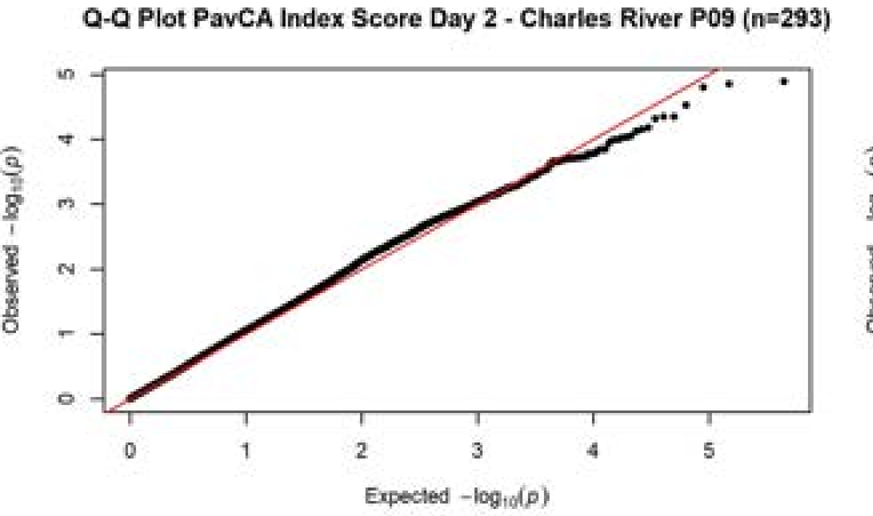
Q-Q Plot PavCA Index Score Day 2 - Charles River P09 (n=293)
Observed $-\log_{10}(p)$
Expected $-\log_{10}(p)$

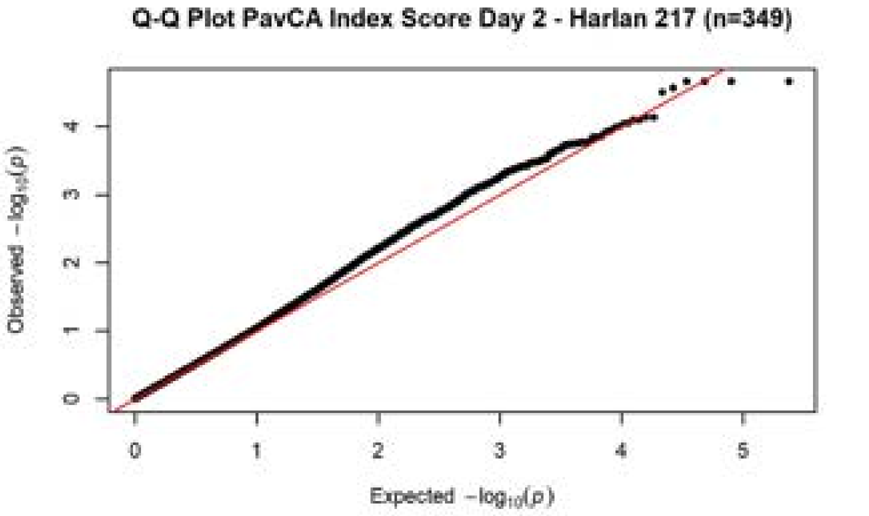
Q-Q Plot PavCA Index Score Day 2 - Harlan 217 (n=349)
Observed $-\log_{10}(p)$
Expected $-\log_{10}(p)$

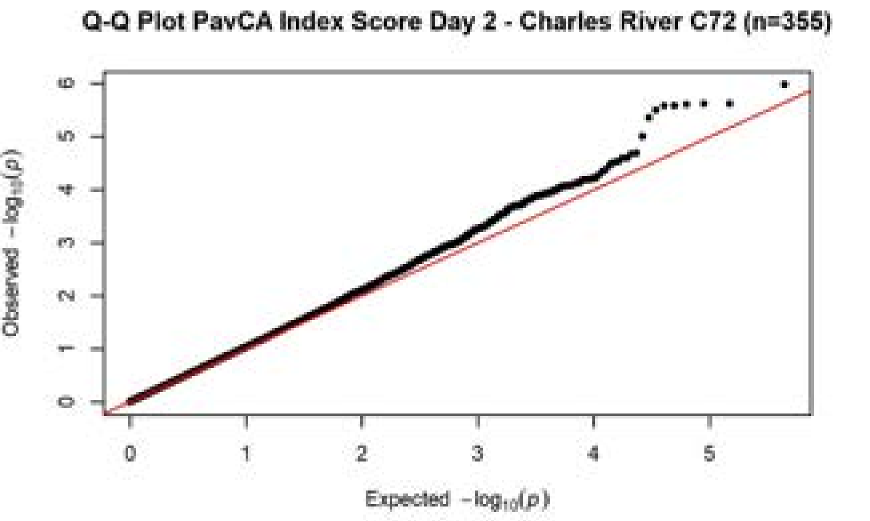
Q-Q Plot PavCA Index Score Day 2 - Charles River C72 (n=355)
Observed $-\log_{10}(p)$
Expected $-\log_{10}(p)$

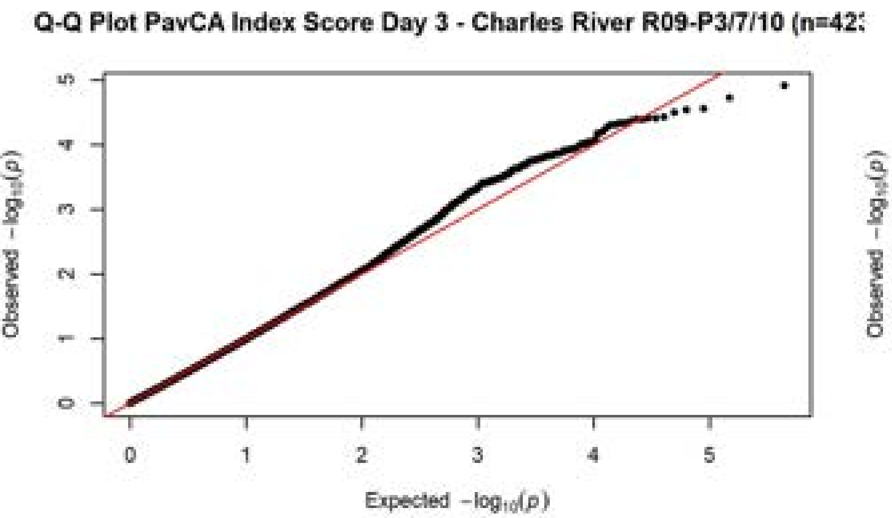
Q-Q Plot PavCA Index Score Day 3 - Charles River R09-P3/7/10 (n=42
Observed $-\log_{10}(p)$
Expected $-\log_{10}(p)$

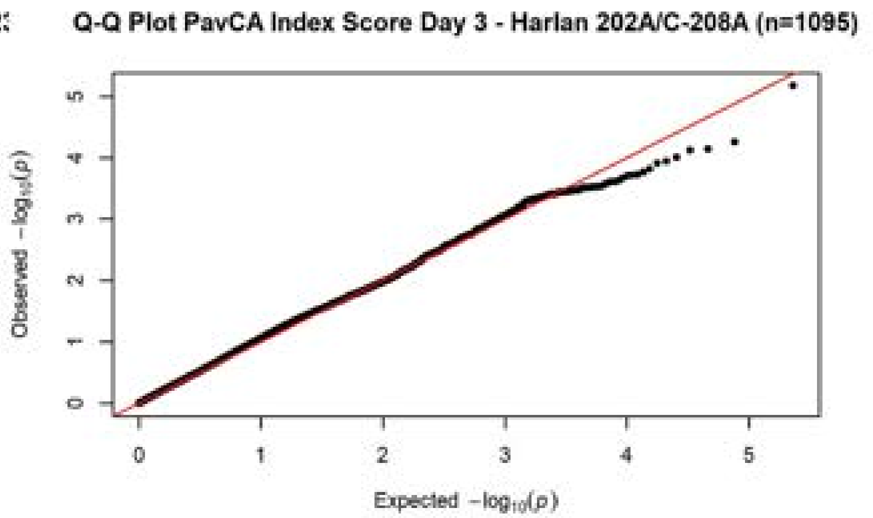
Q-Q Plot PavCA Index Score Day 3 - Harlan 202A/C-208A (n=1095)
Observed $-\log_{10}(p)$
Expected $-\log_{10}(p)$

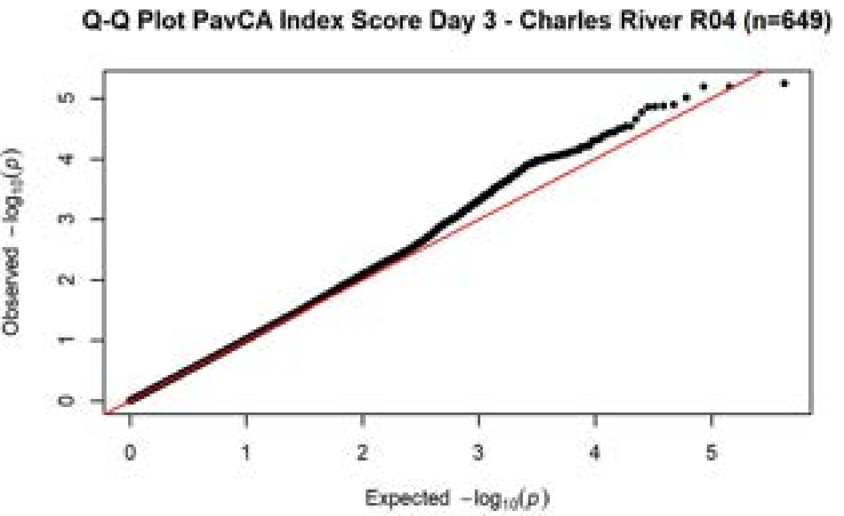
Q-Q Plot PavCA Index Score Day 3 - Charles River R04 (n=649)
Observed $-\log_{10}(p)$
Expected $-\log_{10}(p)$

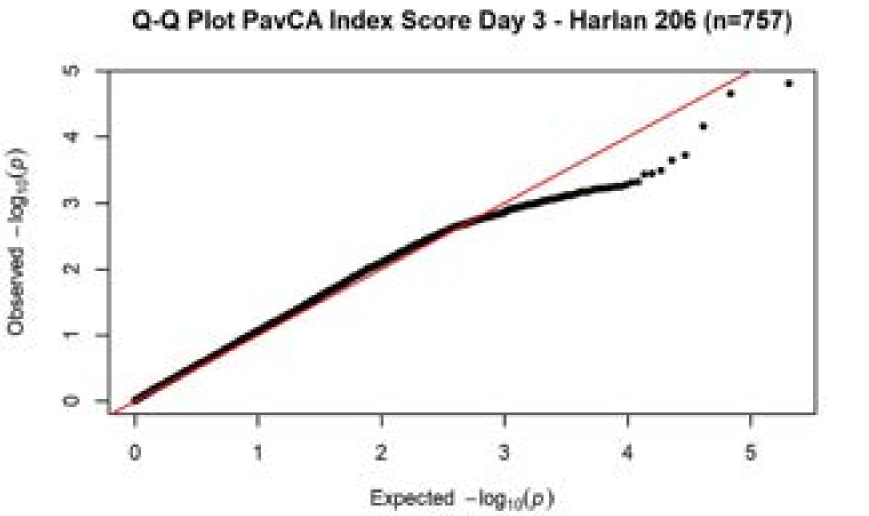
Q-Q Plot PavCA Index Score Day 3 - Harlan 206 (n=757)
Observed $-\log_{10}(p)$
Expected $-\log_{10}(p)$

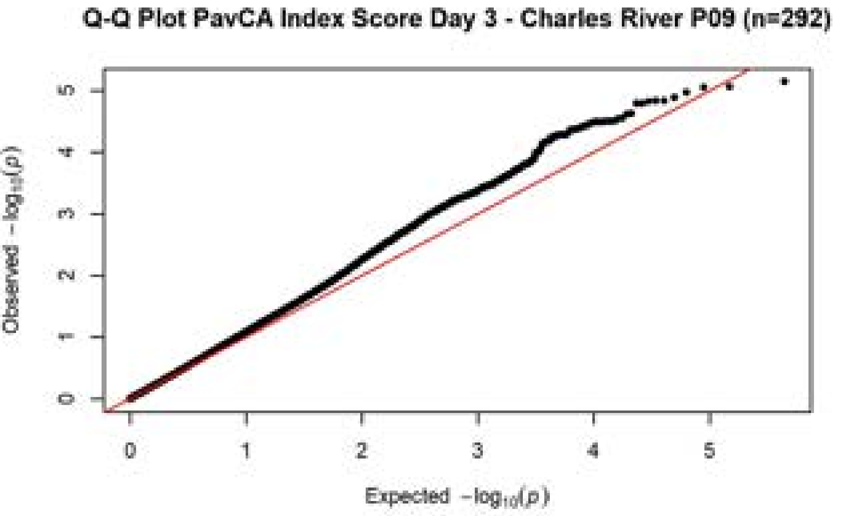
Q-Q Plot PavCA Index Score Day 3 - Charles River P09 (n=292)
Observed $-\log_{10}(p)$
Expected $-\log_{10}(p)$

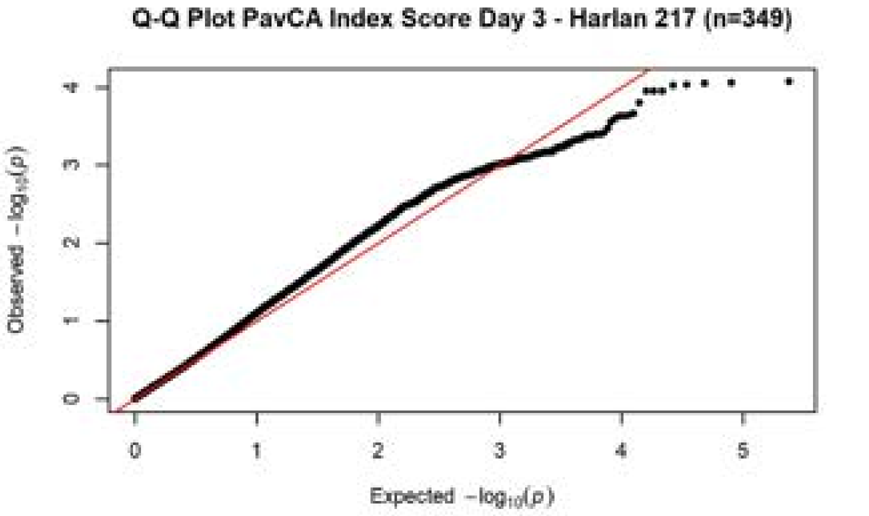
Q-Q Plot PavCA Index Score Day 3 - Harlan 217 (n=349)
Observed $-\log_{10}(p)$
Expected $-\log_{10}(p)$

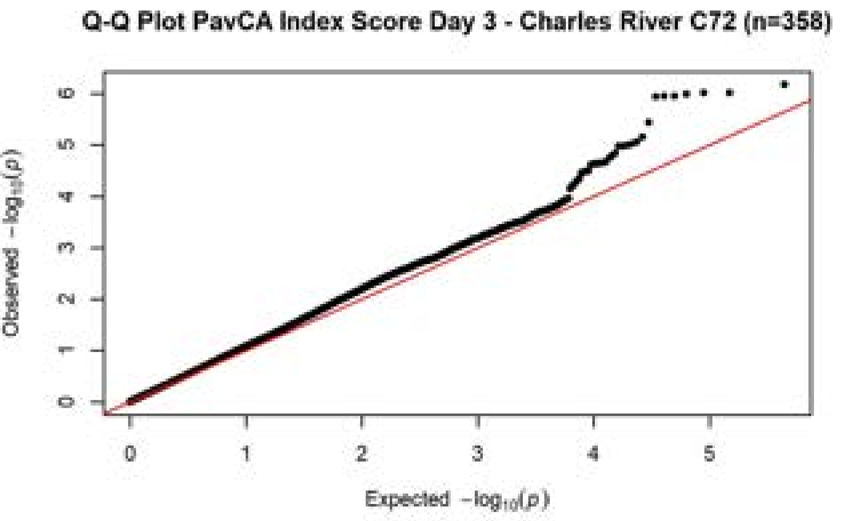
Q-Q Plot PavCA Index Score Day 3 - Charles River C72 (n=358)
Observed $-\log_{10}(p)$
Expected $-\log_{10}(p)$

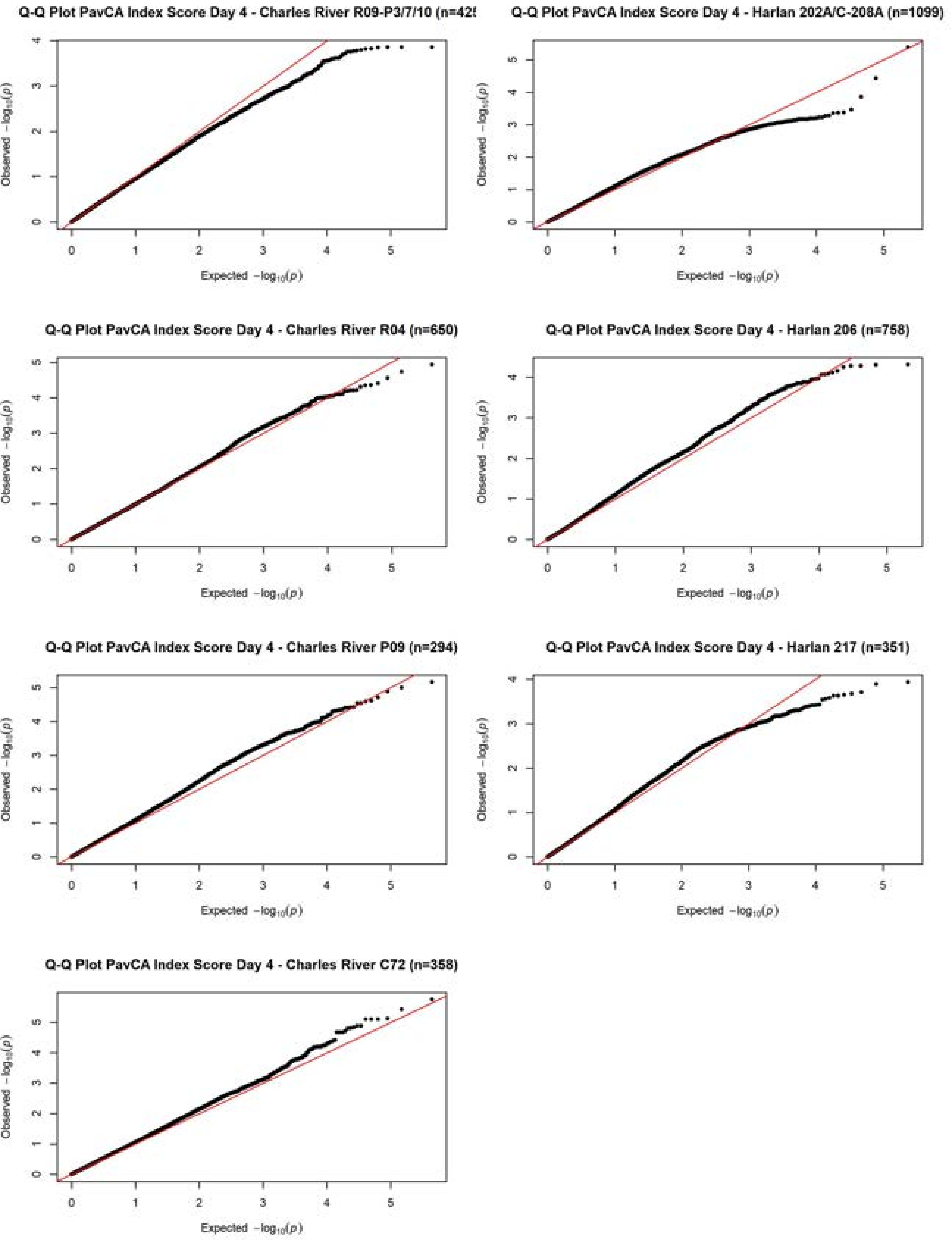
Q-Q Plot PavCA Index Score Day 4 - Charles River R09-P3/7/10 (n=42
Q-Q Plot PavCA Index Score Day 4 - Harlan 202A/C-208A (n=1099)
Q-Q Plot PavCA Index Score Day 4 - Charles River R04 (n=650)
Q-Q Plot PavCA Index Score Day 4 - Harlan 206 (n=758)
Q-Q Plot PavCA Index Score Day 4 - Charles River P09 (n=294)
Q-Q Plot PavCA Index Score Day 4 - Harlan 217 (n=351)
Q-Q Plot PavCA Index Score Day 4 - Charles River C72 (n=358)
Observed $-\log_{10}(p)$
Expected $-\log_{10}(p)$

Q-Q Plot PavCA Index Score Day 5 - Charles River R09-P3/7/10 (n=42[illegible]

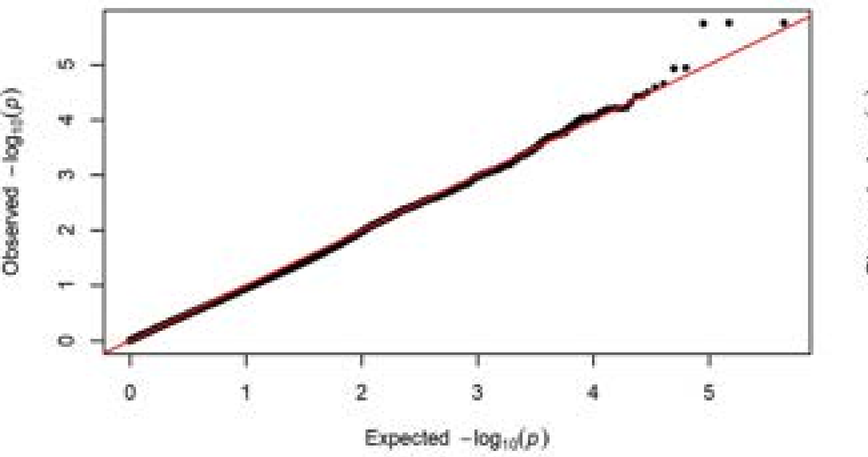

Q-Q Plot PavCA Index Score Day 5 - Harlan 202A/C-208A (n=1099)

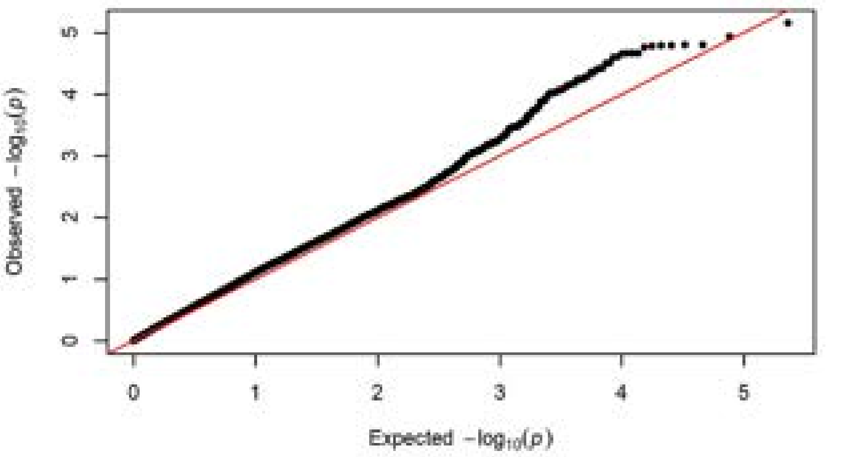

Q-Q Plot PavCA Index Score Day 5 - Charles River R04 (n=650)

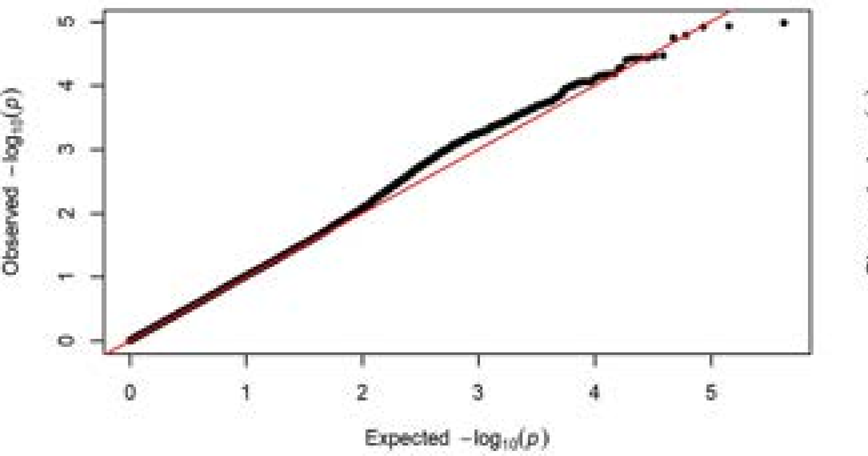

Q-Q Plot PavCA Index Score Day 5 - Harlan 206 (n=757)

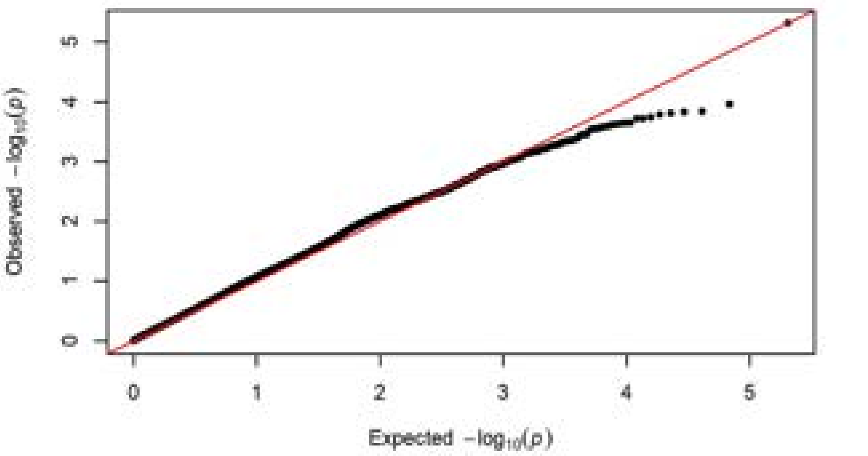

Q-Q Plot PavCA Index Score Day 5 - Charles River P09 (n=293)

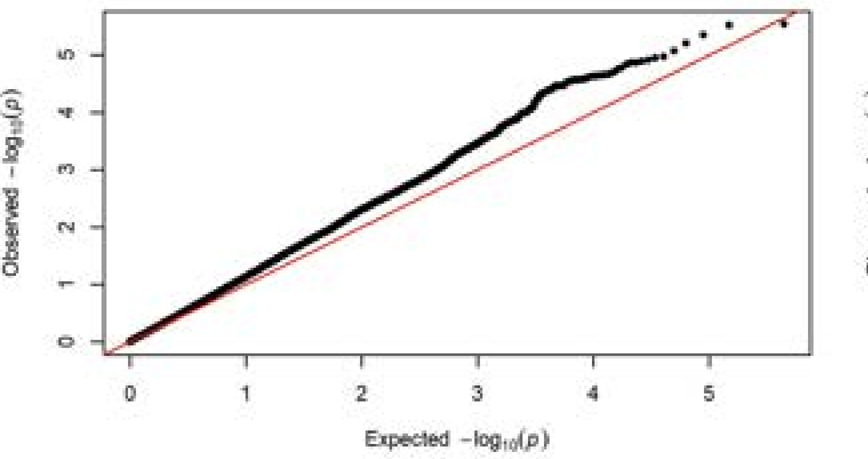

Q-Q Plot PavCA Index Score Day 5 - Harlan 217 (n=351)

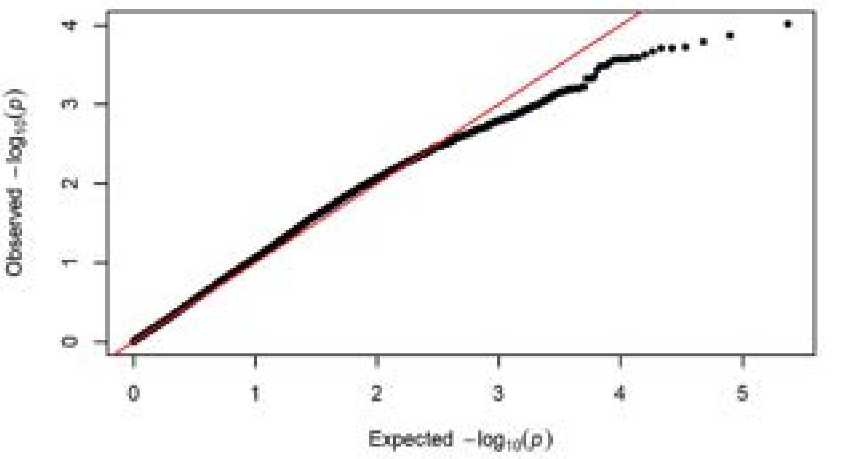

Q-Q Plot PavCA Index Score Day 5 - Charles River C72 (n=358)

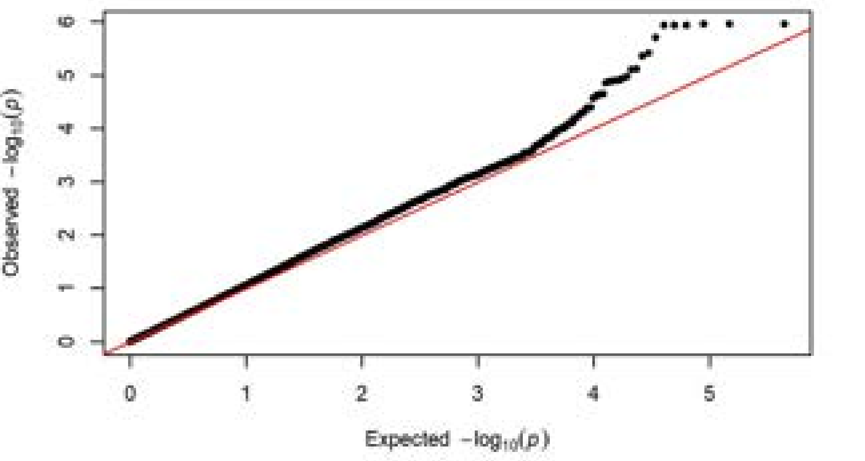

Q-Q Plot Latency Score Day 1 - Charles River R09-P3/7/10 (n=425)

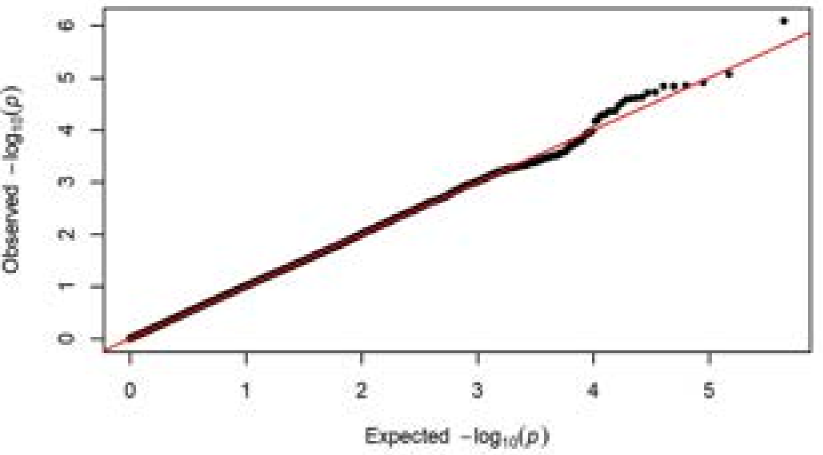

Q-Q Plot Latency Score Day 1 - Harlan 202A/C-208A (n=1066)

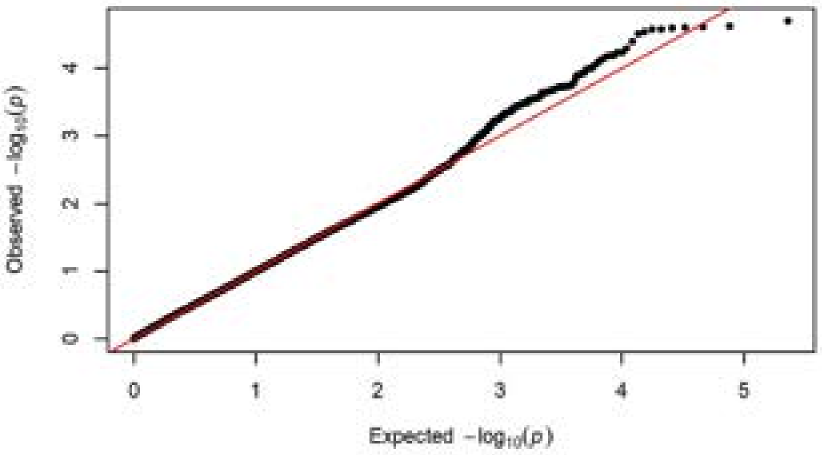

Q-Q Plot Latency Score Day 1 - Charles River R04 (n=650)

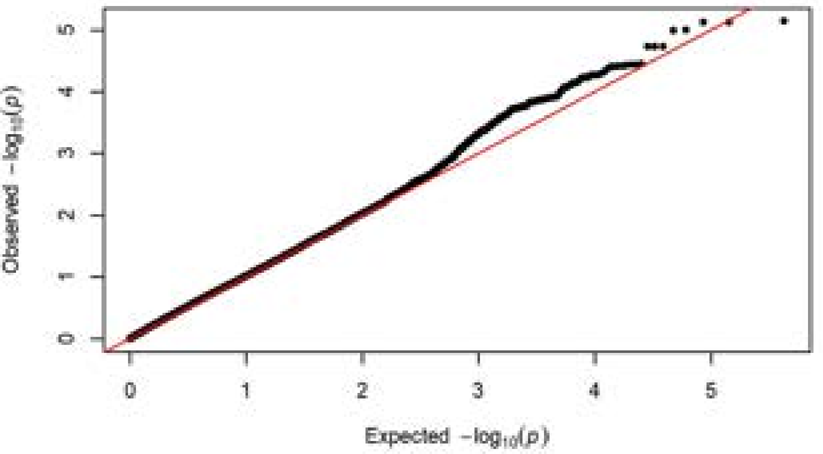

Q-Q Plot Latency Score Day 1 - Harlan 206 (n=758)

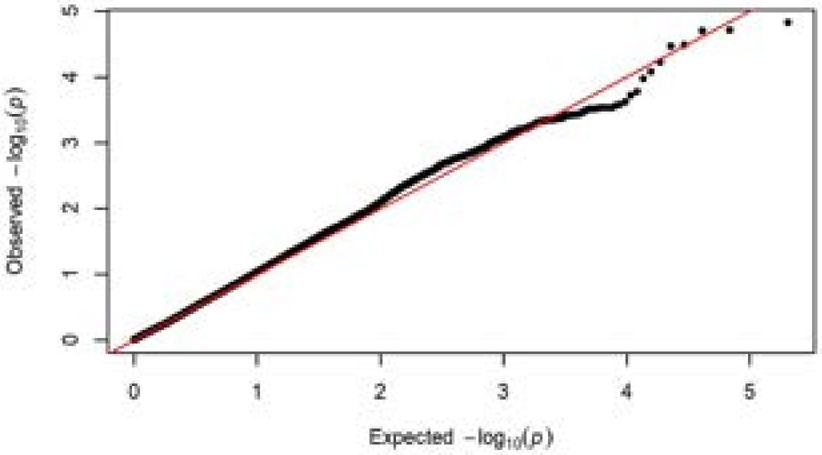

Q-Q Plot Latency Score Day 1 - Charles River P09 (n=295)

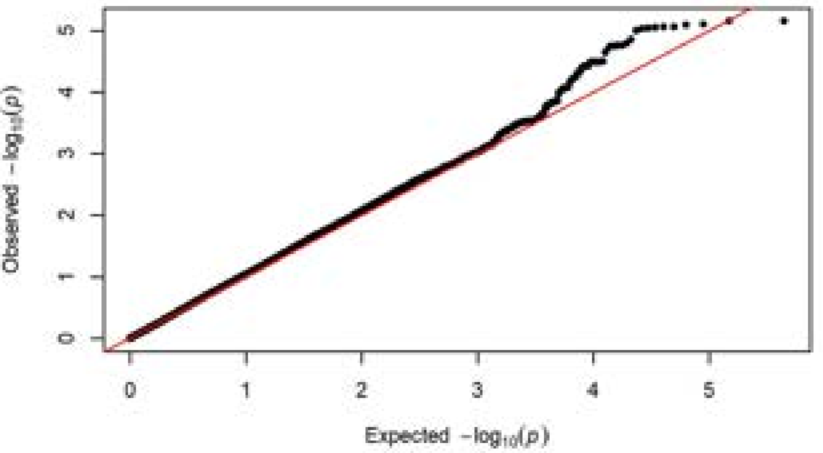

Q-Q Plot Latency Score Day 1 - Harlan 217 (n=351)

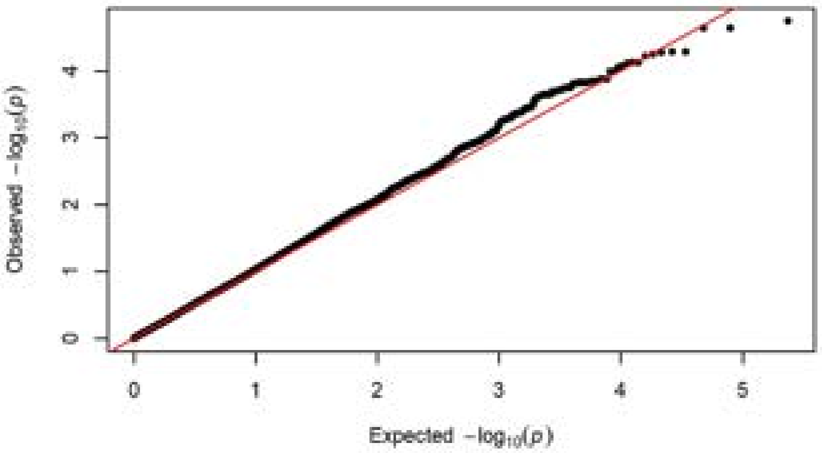

Q-Q Plot Latency Score Day 1 - Charles River C72 (n=358)

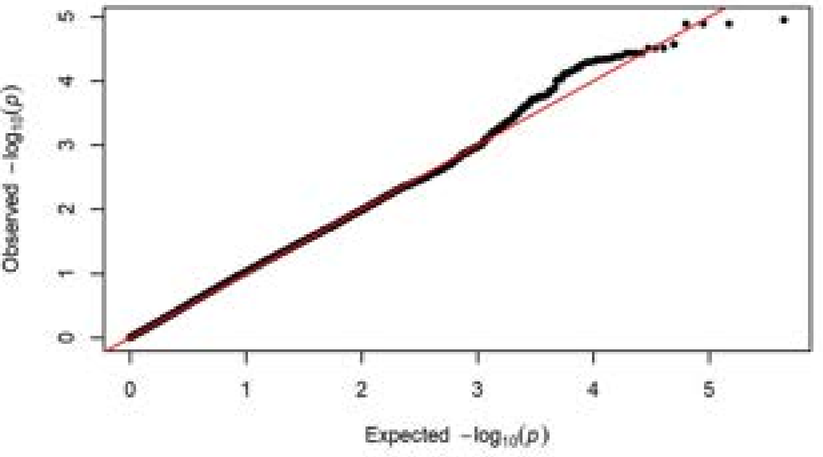

Q-Q Plot Latency Score Day 2 - Charles River R09-P3/7/10 (n=425)

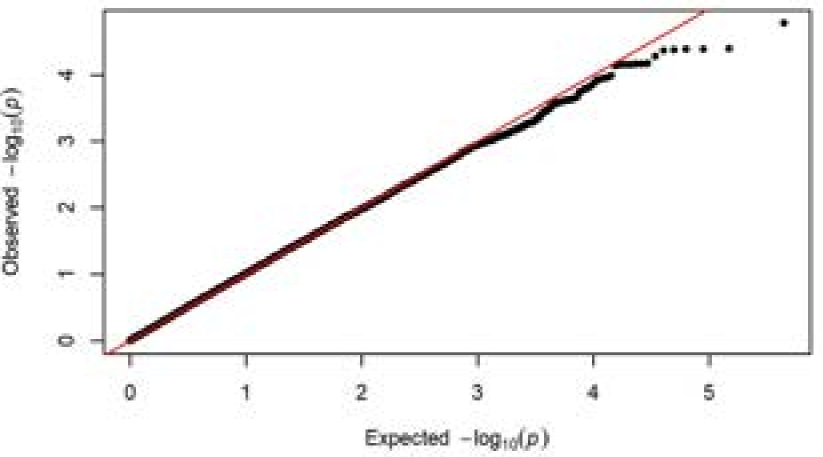

Q-Q Plot Latency Score Day 2 - Harlan 202A/C-208A (n=1099)

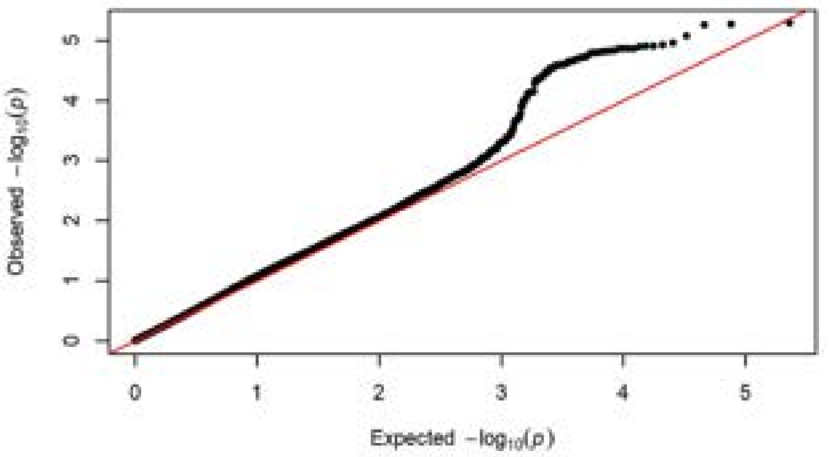

Q-Q Plot Latency Score Day 2 - Charles River R04 (n=650)

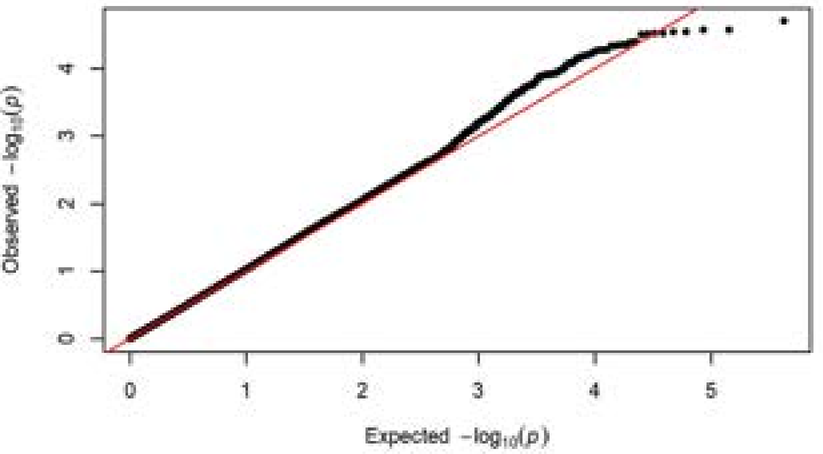

Q-Q Plot Latency Score Day 2 - Harlan 206 (n=758)

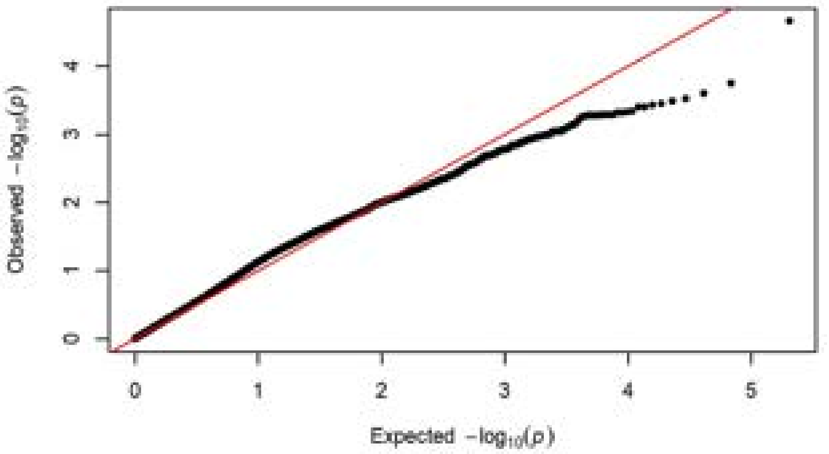

Q-Q Plot Latency Score Day 2 - Charles River P09 (n=295)

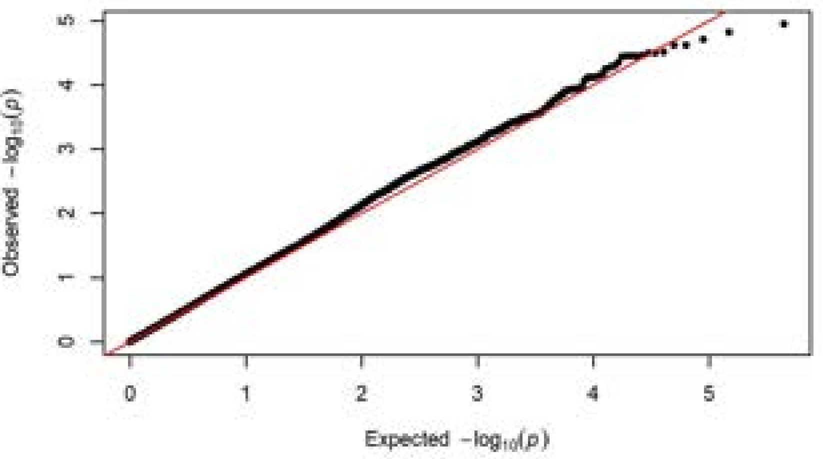

Q-Q Plot Latency Score Day 2 - Harlan 217 (n=351)

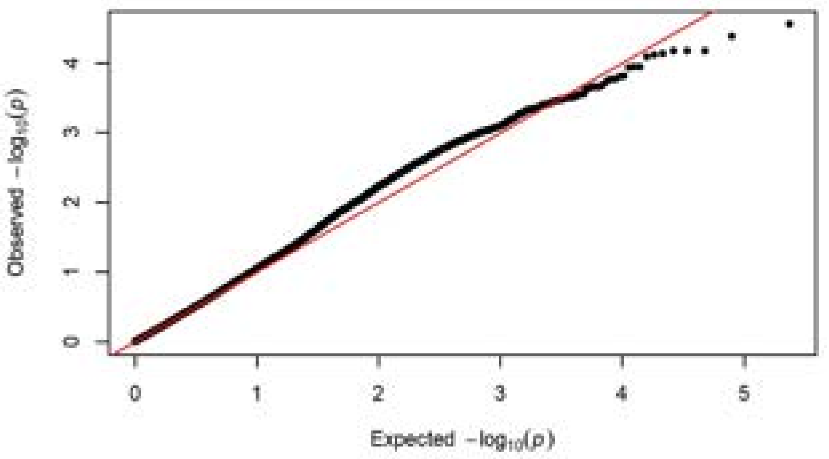

Q-Q Plot Latency Score Day 2 - Charles River C72 (n=356)

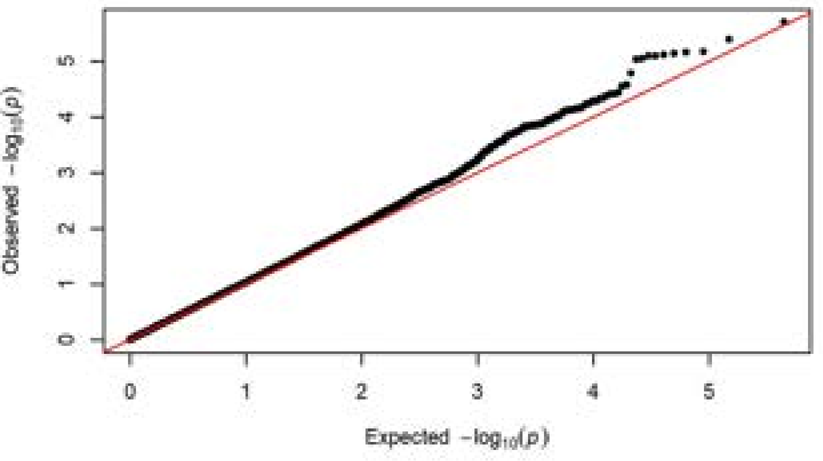

Q-Q Plot Latency Score Day 3 - Charles River R09-P3/7/10 (n=424)

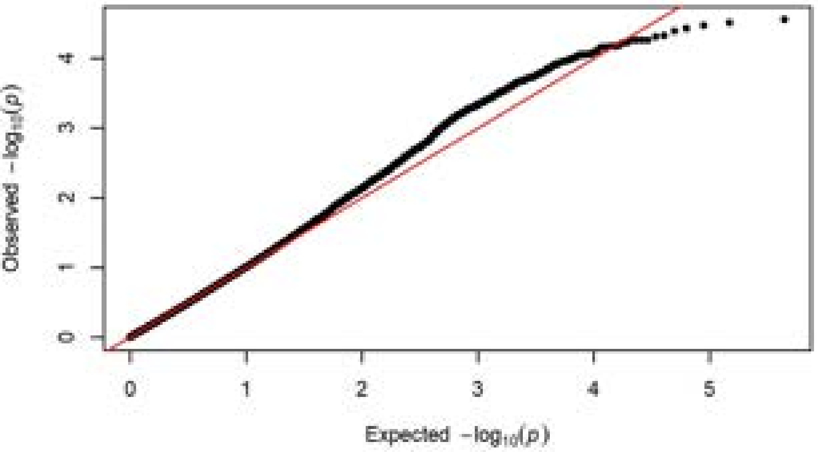

Q-Q Plot Latency Score Day 3 - Harlan 202A/C-208A (n=1096)

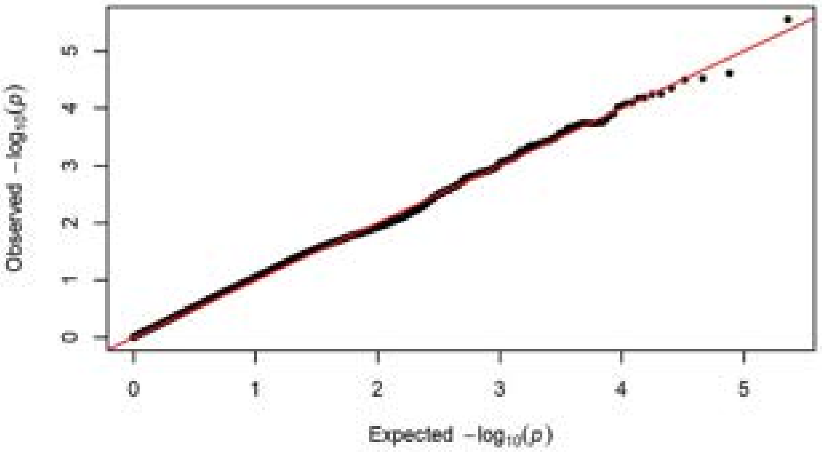

Q-Q Plot Latency Score Day 3 - Charles River R04 (n=650)

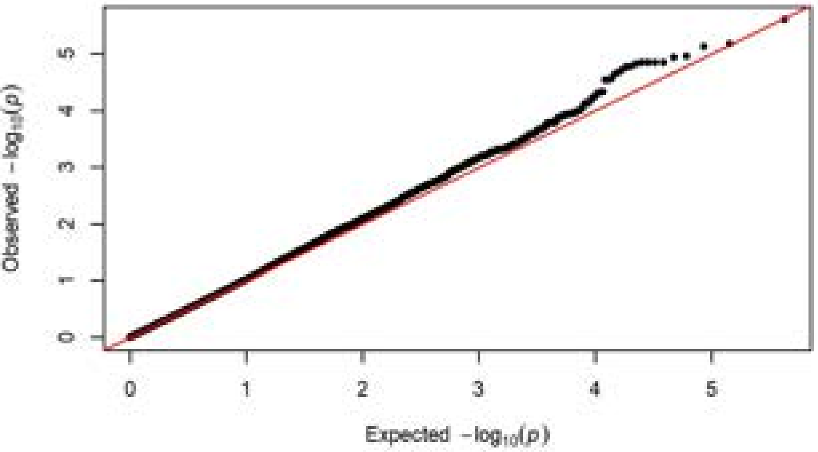

Q-Q Plot Latency Score Day 3 - Harlan 206 (n=758)

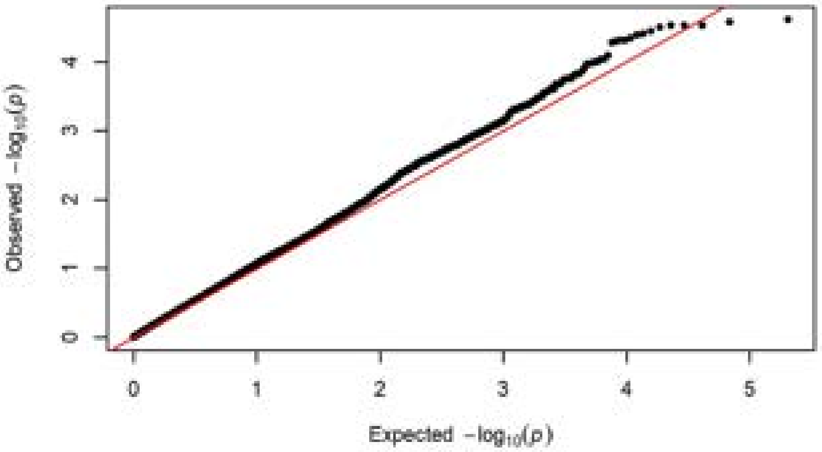

Q-Q Plot Latency Score Day 3 - Charles River P09 (n=295)

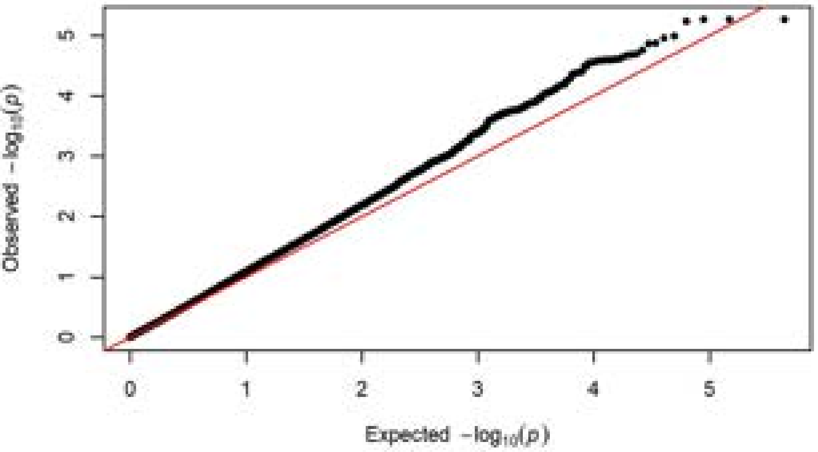

Q-Q Plot Latency Score Day 3 - Harlan 217 (n=351)

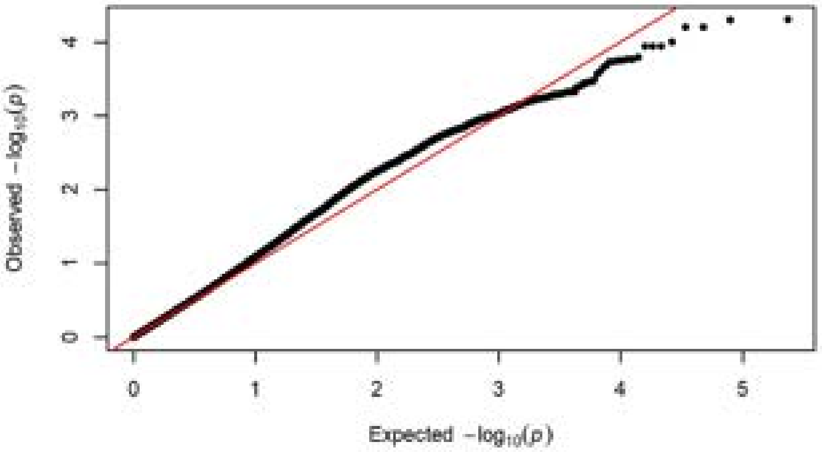

Q-Q Plot Latency Score Day 3 - Charles River C72 (n=358)

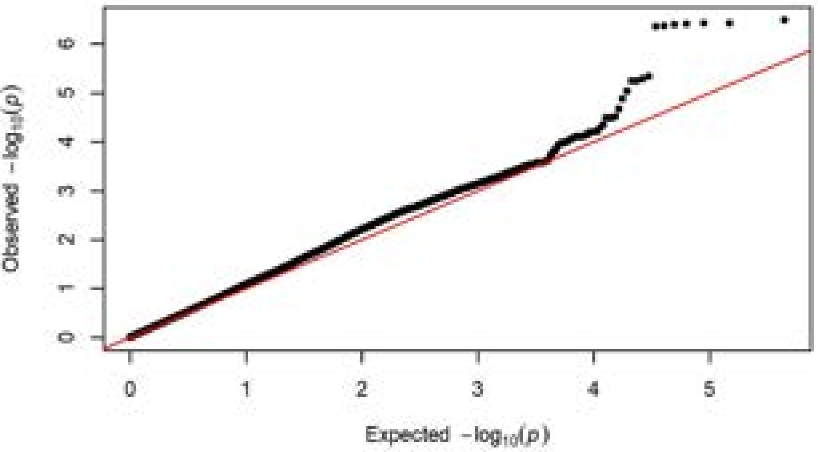

Q-Q Plot Latency Score Day 4 - Charles River R09-P3/7/10 (n=425)

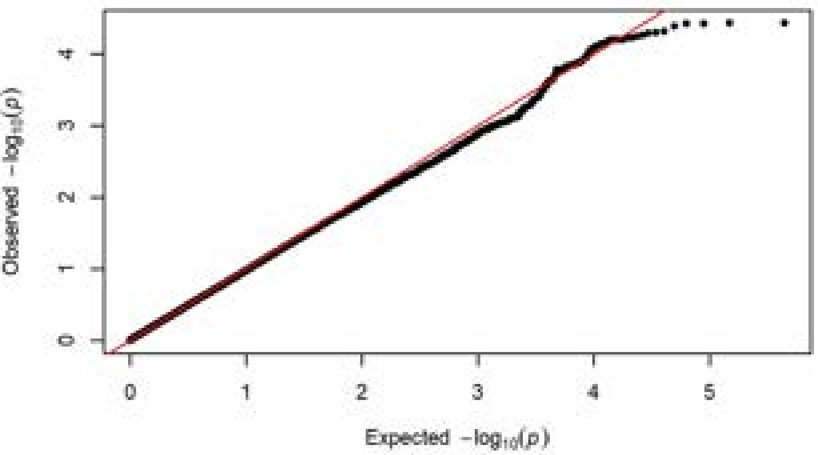

Q-Q Plot Latency Score Day 4 - Harlan 202A/C-208A (n=1099)

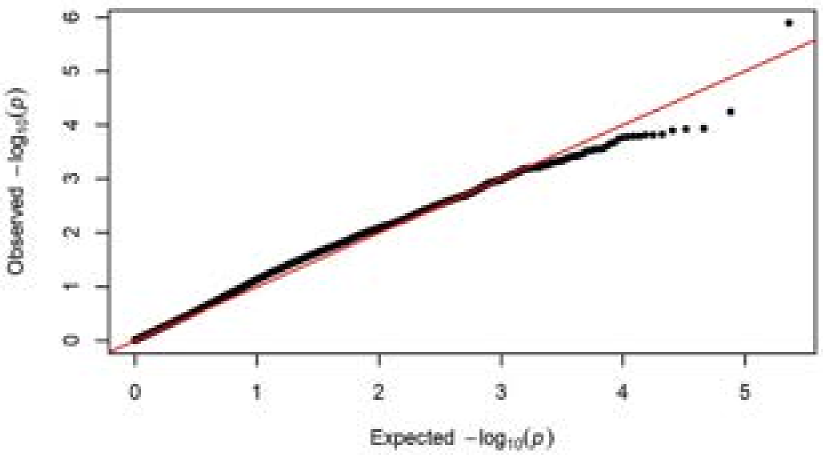

Q-Q Plot Latency Score Day 4 - Charles River R04 (n=650)

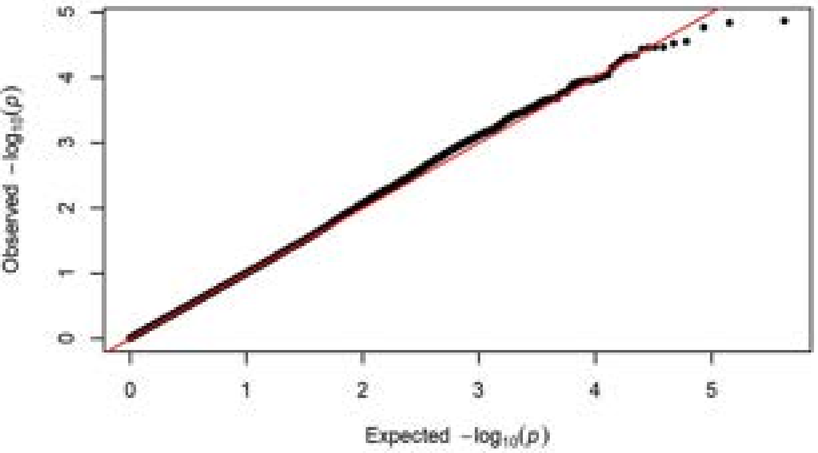

Q-Q Plot Latency Score Day 4 - Harlan 206 (n=758)

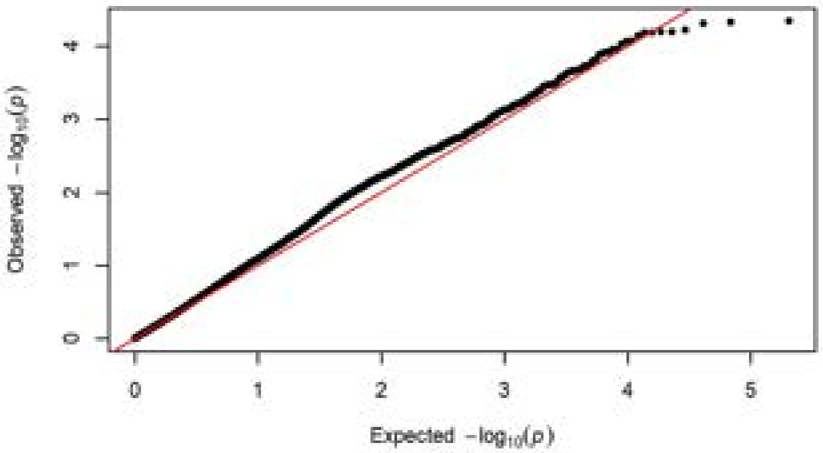

Q-Q Plot Latency Score Day 4 - Charles River P09 (n=295)

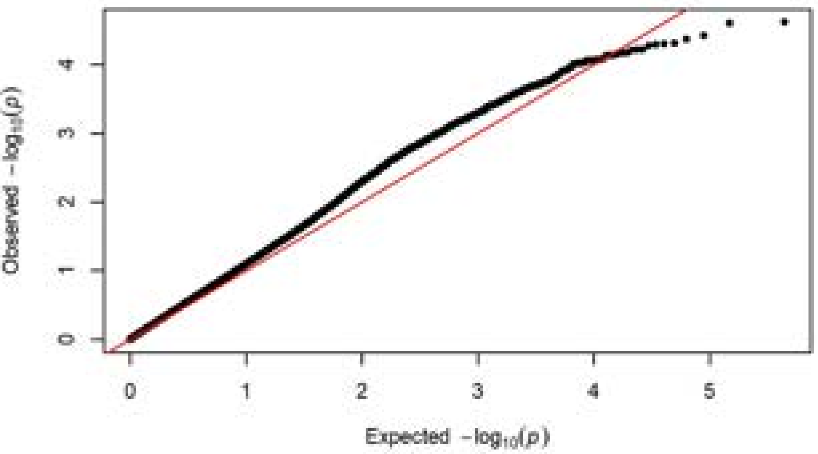

Q-Q Plot Latency Score Day 4 - Harlan 217 (n=351)

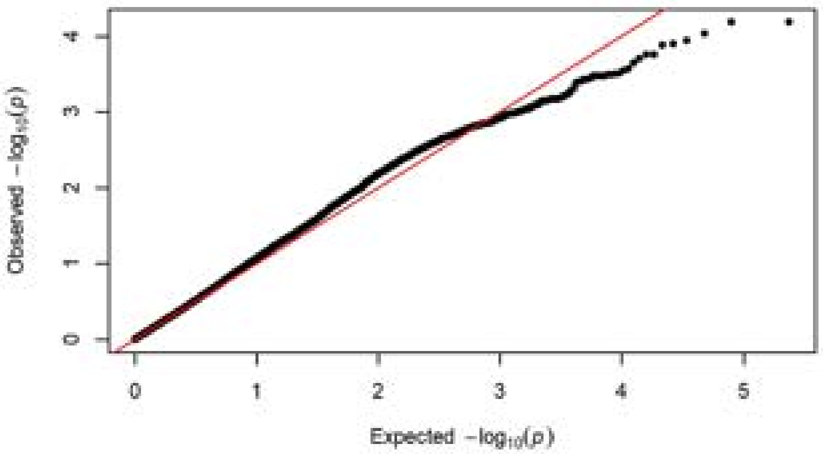

Q-Q Plot Latency Score Day 4 - Charles River C72 (n=358)

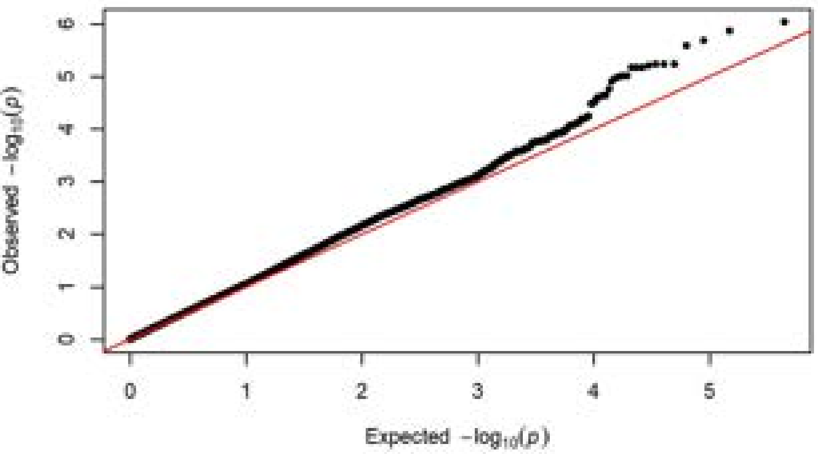

Q-Q Plot Latency Score Day 5 - Charles River R09-P3/7/10 (n=425)

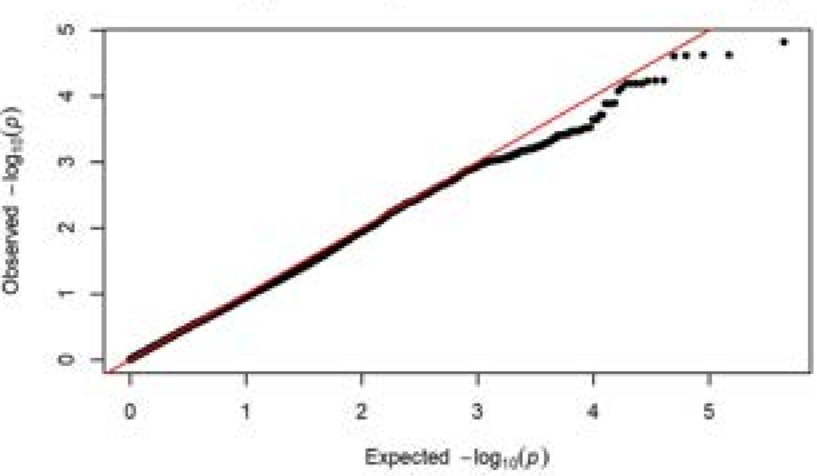

Q-Q Plot Latency Score Day 5 - Harlan 202A/C-208A (n=1099)

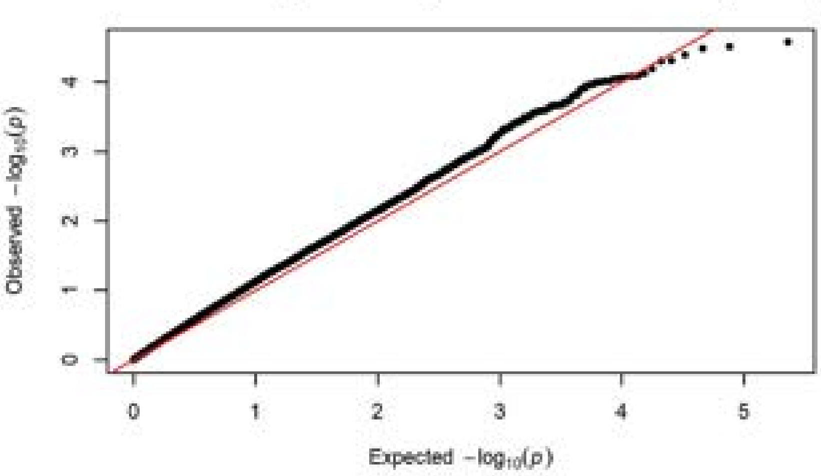

Q-Q Plot Latency Score Day 5 - Charles River R04 (n=650)

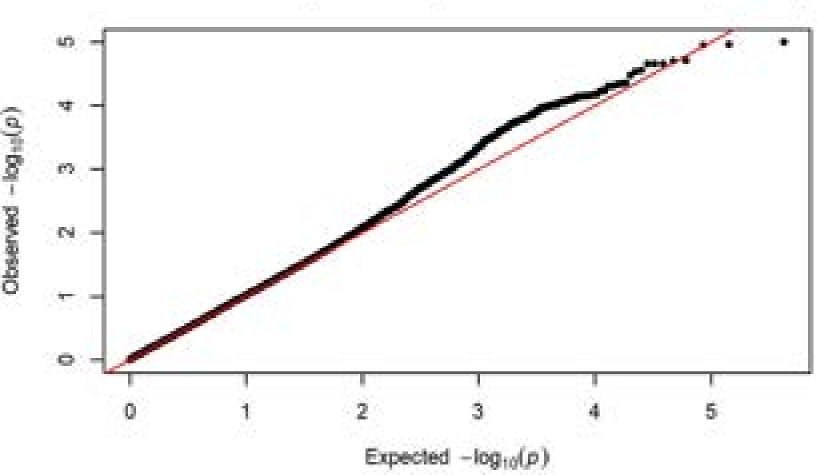

Q-Q Plot Latency Score Day 5 - Harlan 206 (n=758)

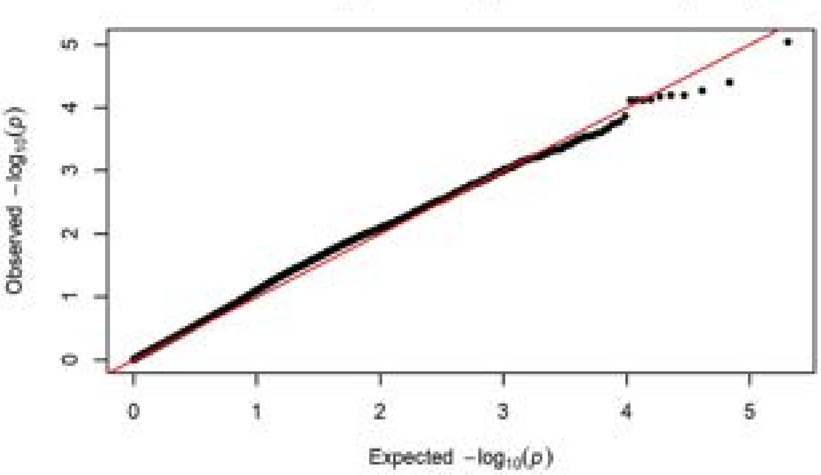

Q-Q Plot Latency Score Day 5 - Charles River P09 (n=295)

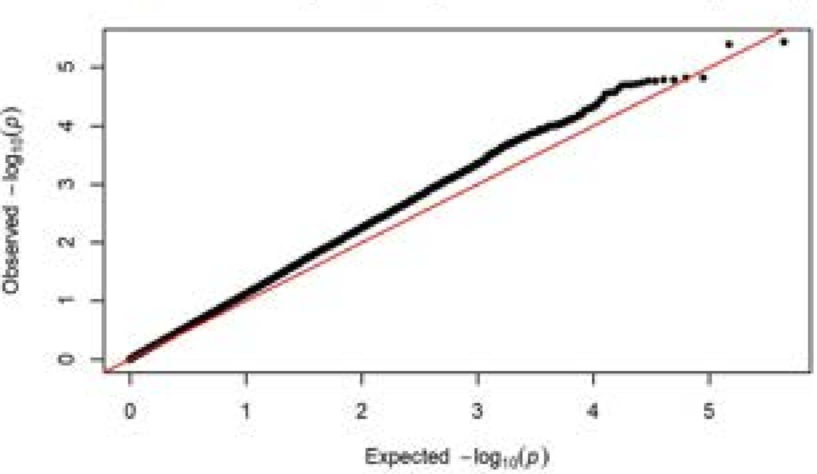

Q-Q Plot Latency Score Day 5 - Harlan 217 (n=351)

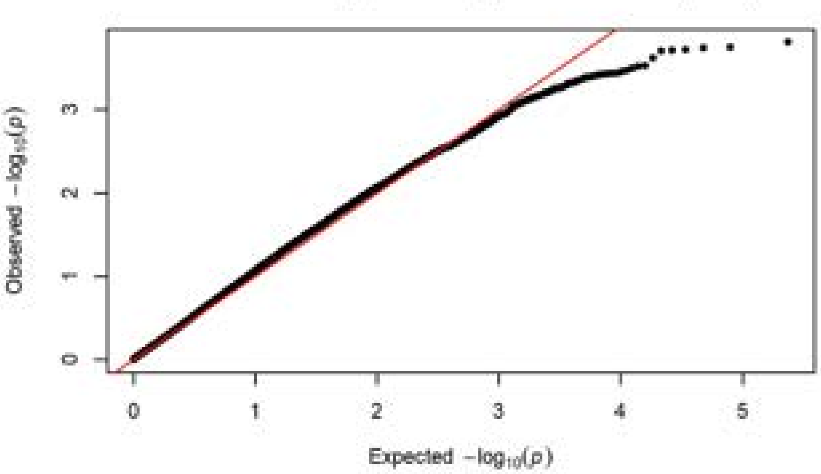

Q-Q Plot Latency Score Day 5 - Charles River C72 (n=358)

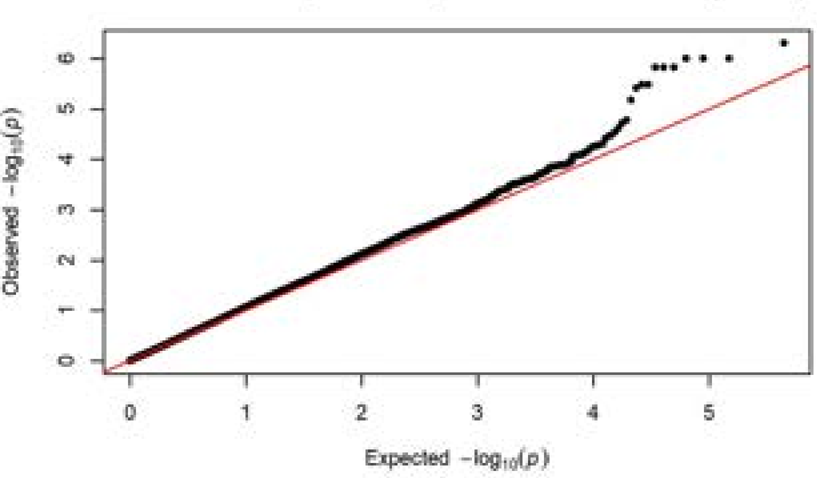

Q-Q Plot Lever Presses Day 1 - Charles River R09-P3/7/10 (n=425)

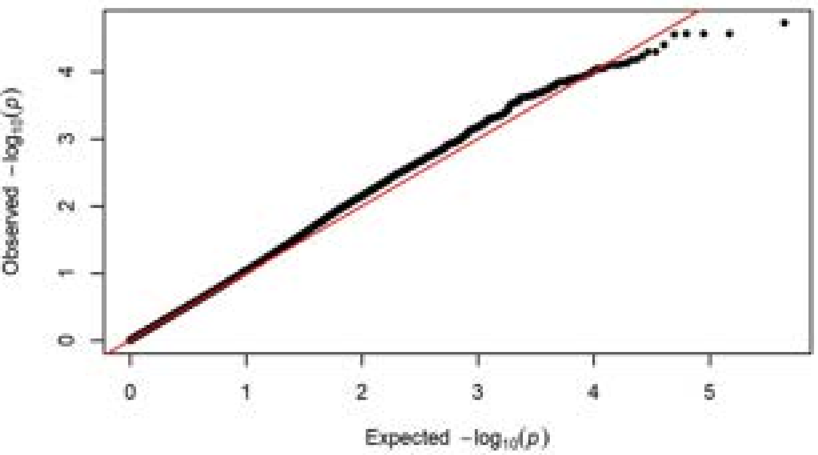

Q-Q Plot Lever Presses Day 1 - Harlan 202A/C-208A (n=1098)

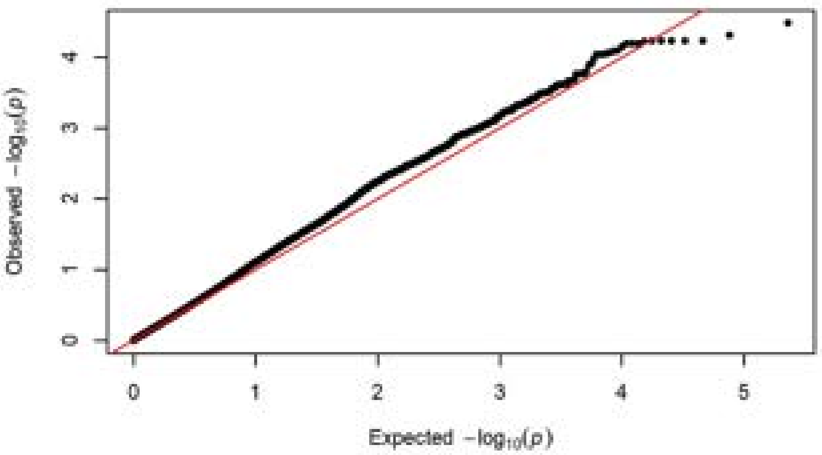

Q-Q Plot Lever Presses Day 1 - Charles River R04 (n=650)

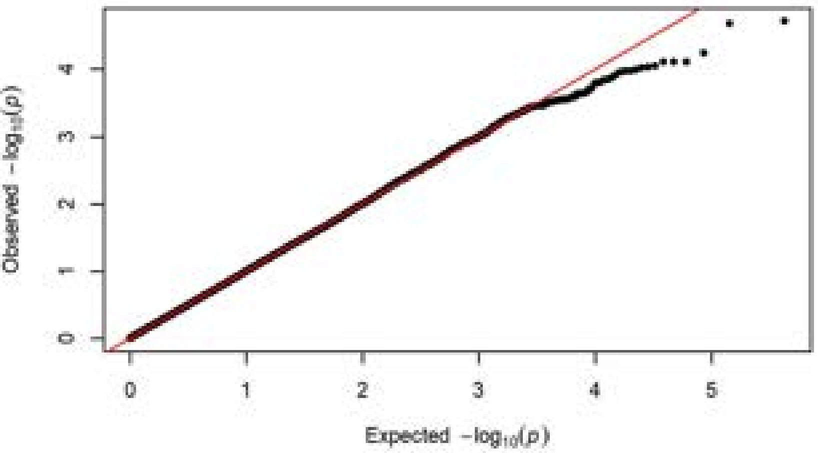

Q-Q Plot Lever Presses Day 1 - Harlan 206 (n=758)

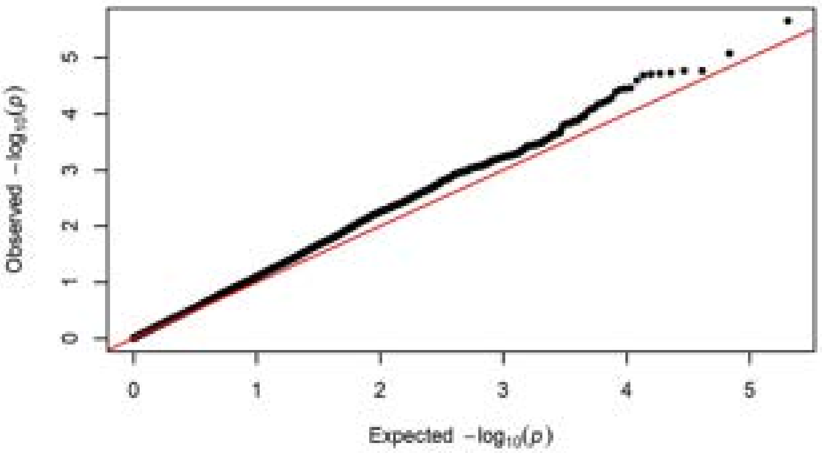

Q-Q Plot Lever Presses Day 1 - Charles River P09 (n=294)

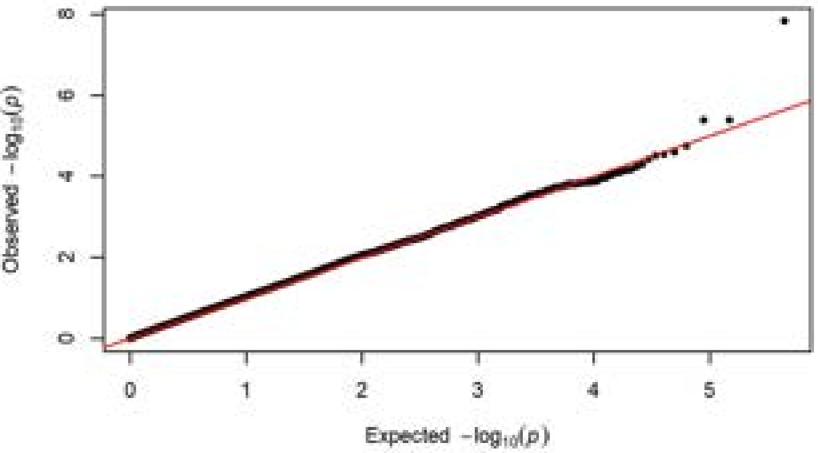

Q-Q Plot Lever Presses Day 1 - Harlan 217 (n=350)

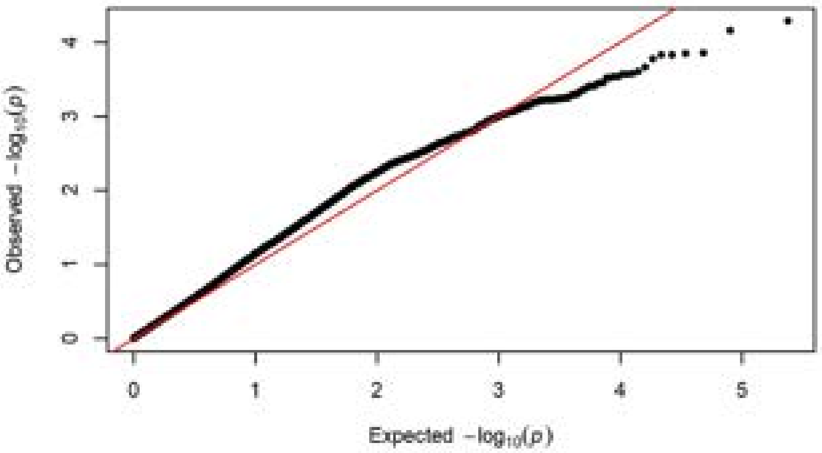

Q-Q Plot Lever Presses Day 1 - Charles River C72 (n=358)

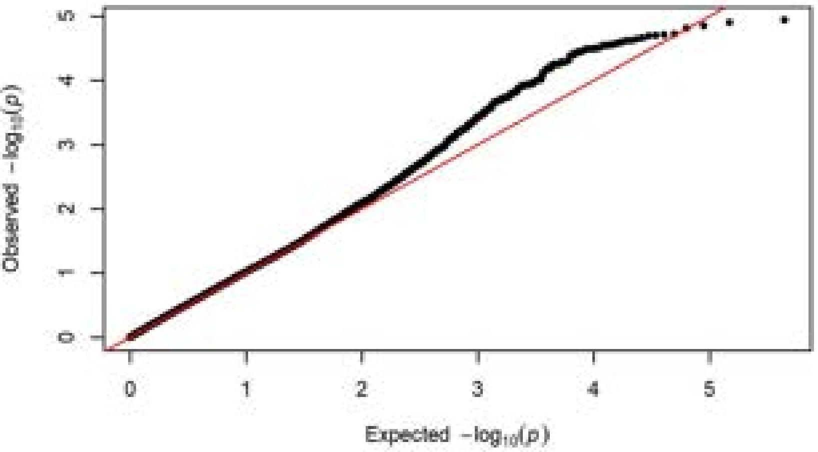

Q-Q Plot Lever Presses Day 2 - Charles River R09-P3/7/10 (n=425)

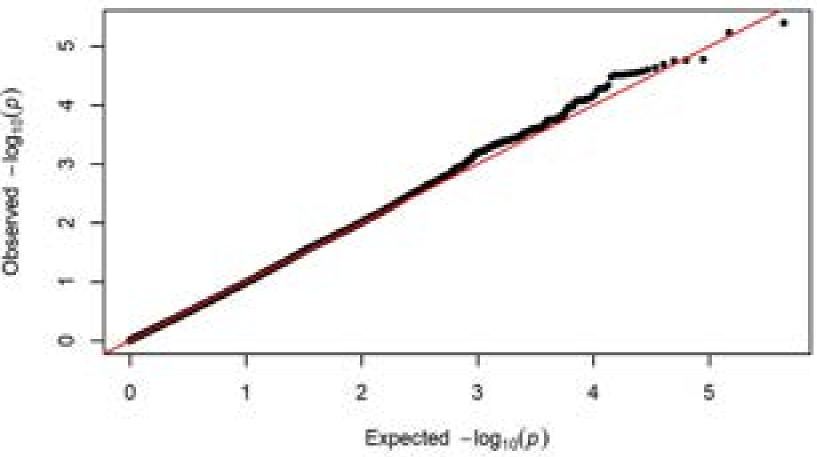

Q-Q Plot Lever Presses Day 2 - Harlan 202A/C-208A (n=1098)

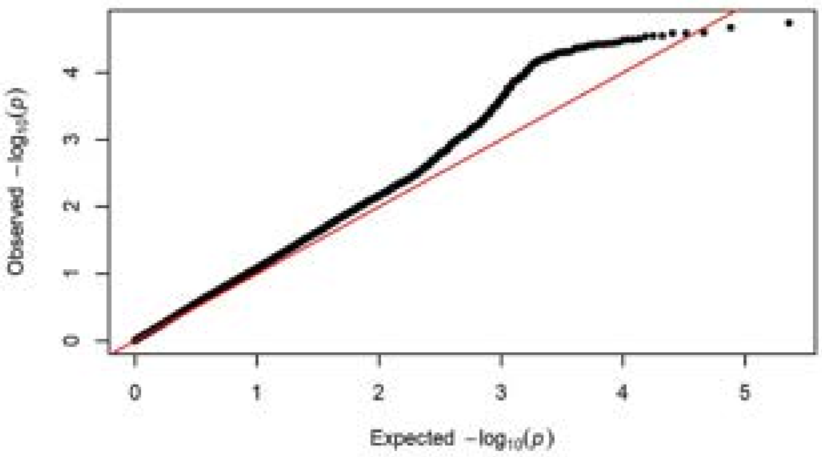

Q-Q Plot Lever Presses Day 2 - Charles River R04 (n=650)

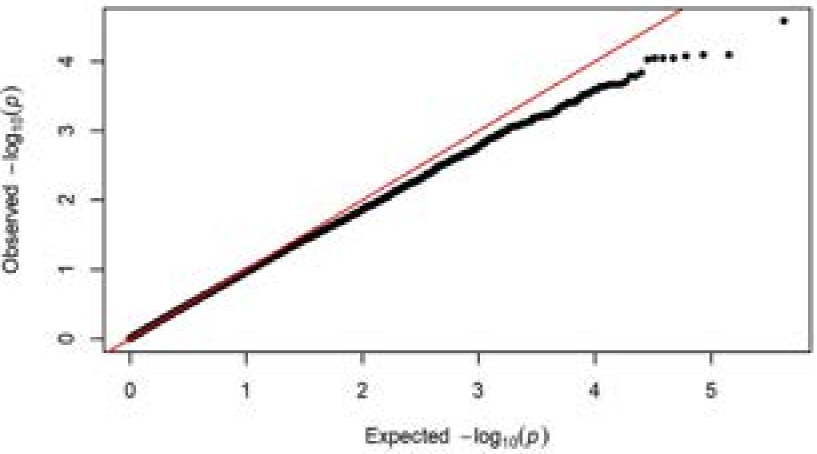

Q-Q Plot Lever Presses Day 2 - Harlan 206 (n=757)

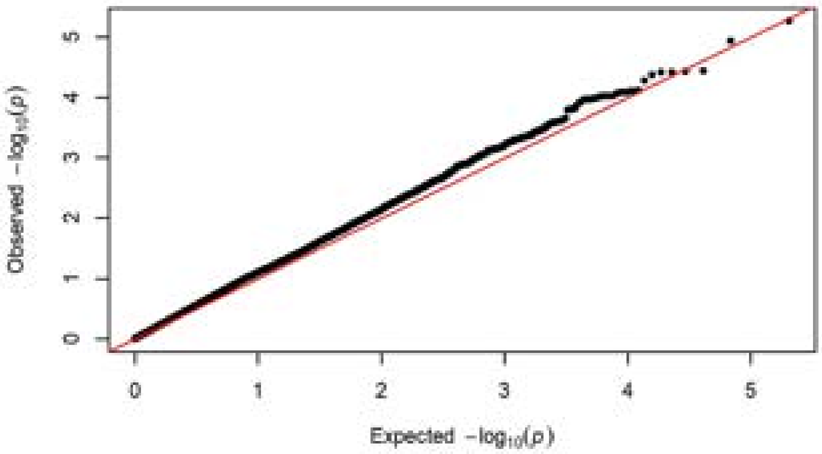

Q-Q Plot Lever Presses Day 2 - Charles River P09 (n=295)

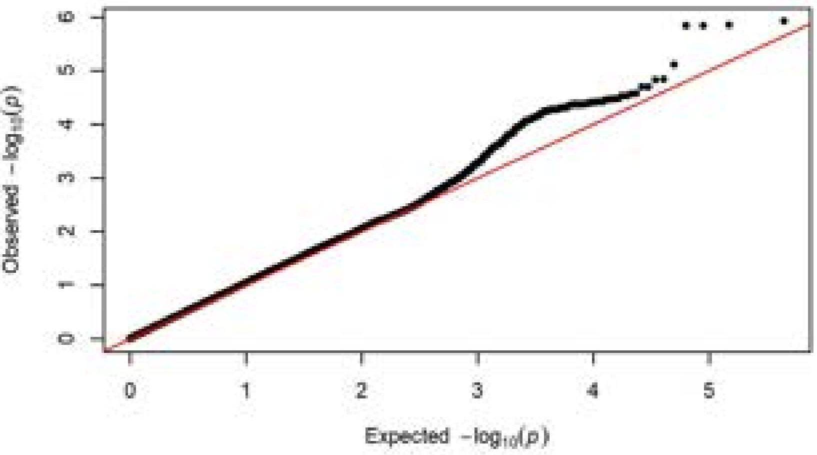

Q-Q Plot Lever Presses Day 2 - Harlan 217 (n=350)

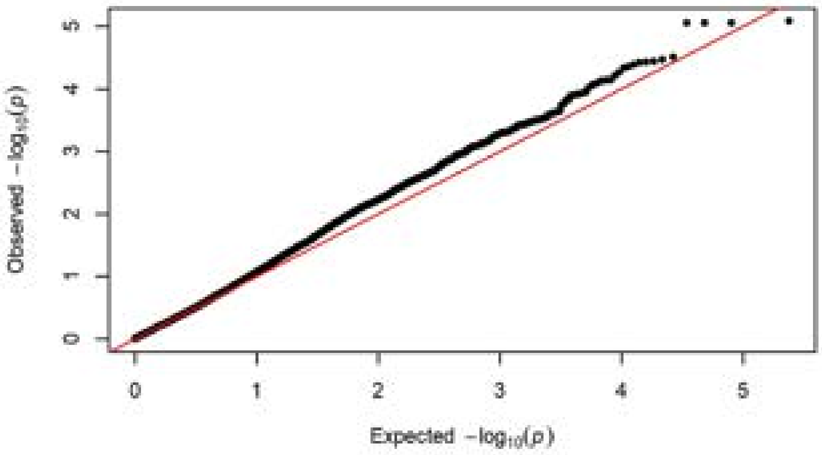

Q-Q Plot Lever Presses Day 2 - Charles River C72 (n=356)

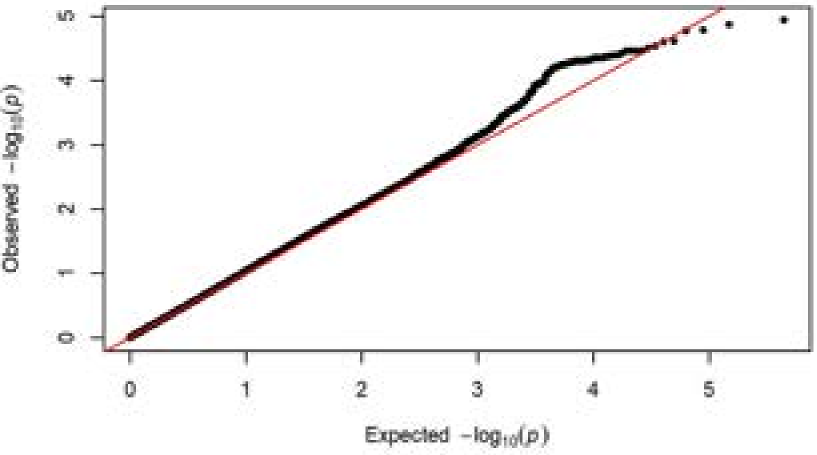

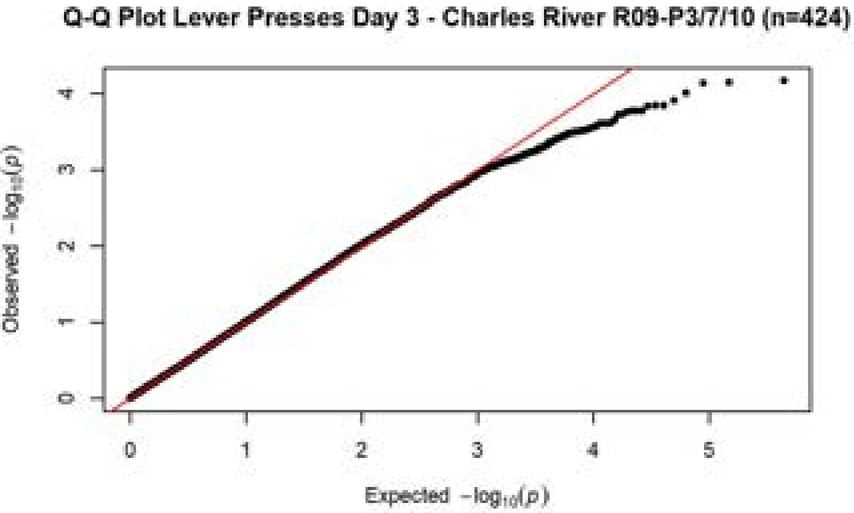
Q-Q Plot Lever Presses Day 3 - Charles River R09-P3/7/10 (n=424)
Observed −log10(p)
Expected −log10(p)

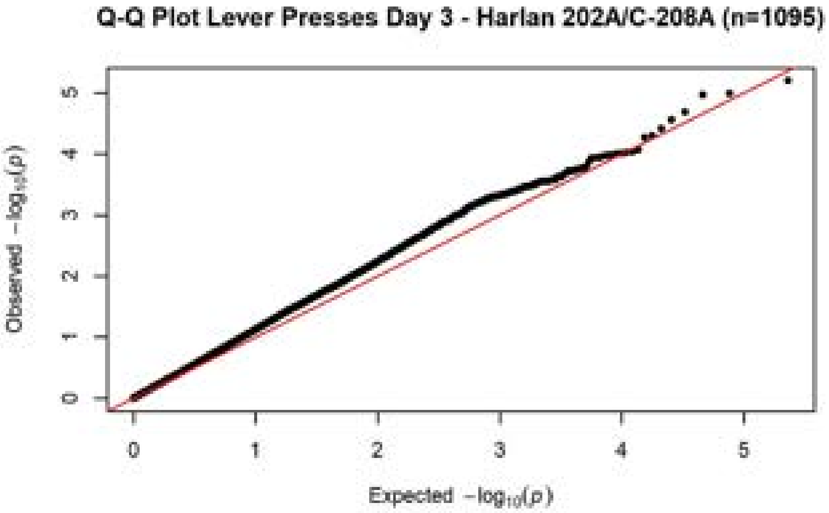
Q-Q Plot Lever Presses Day 3 - Harlan 202A/C-208A (n=1095)
Observed −log10(p)
Expected −log10(p)

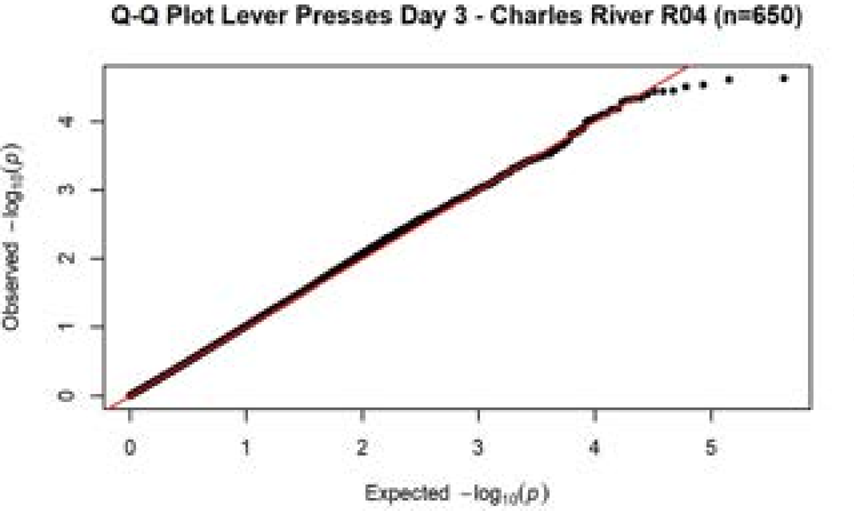
Q-Q Plot Lever Presses Day 3 - Charles River R04 (n=650)
Observed −log10(p)
Expected −log10(p)

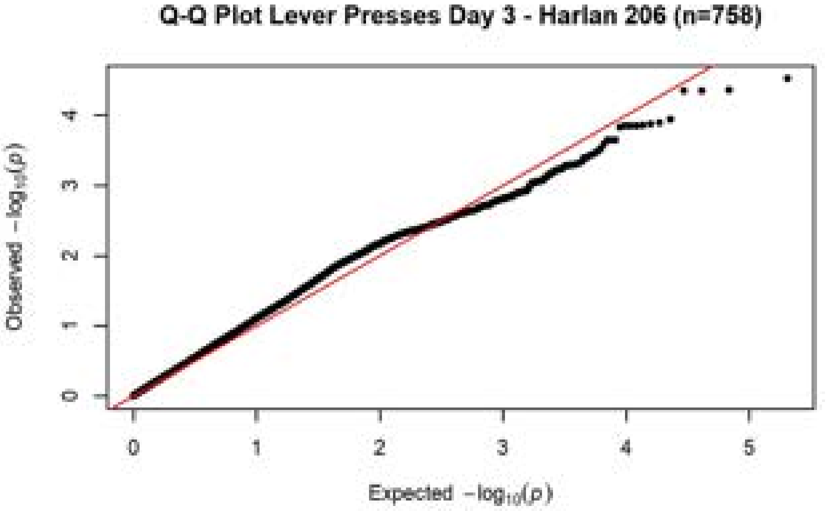
Q-Q Plot Lever Presses Day 3 - Harlan 206 (n=758)
Observed −log10(p)
Expected −log10(p)

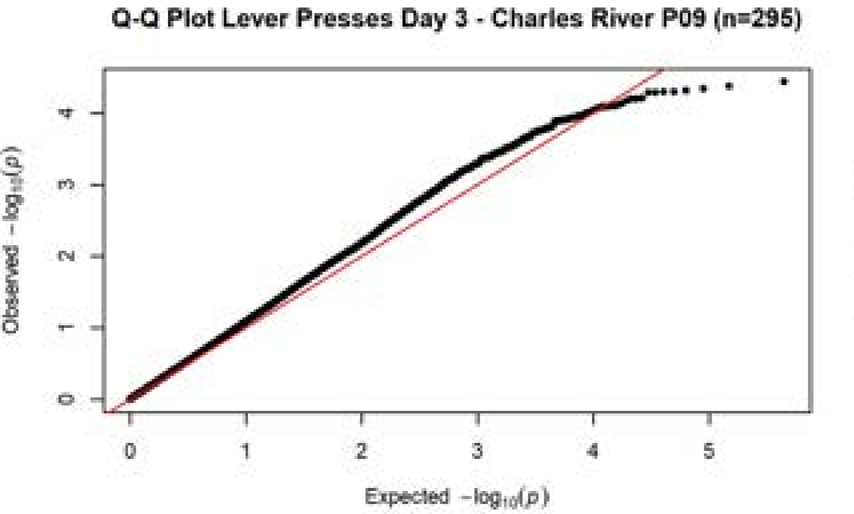
Q-Q Plot Lever Presses Day 3 - Charles River P09 (n=295)
Observed −log10(p)
Expected −log10(p)

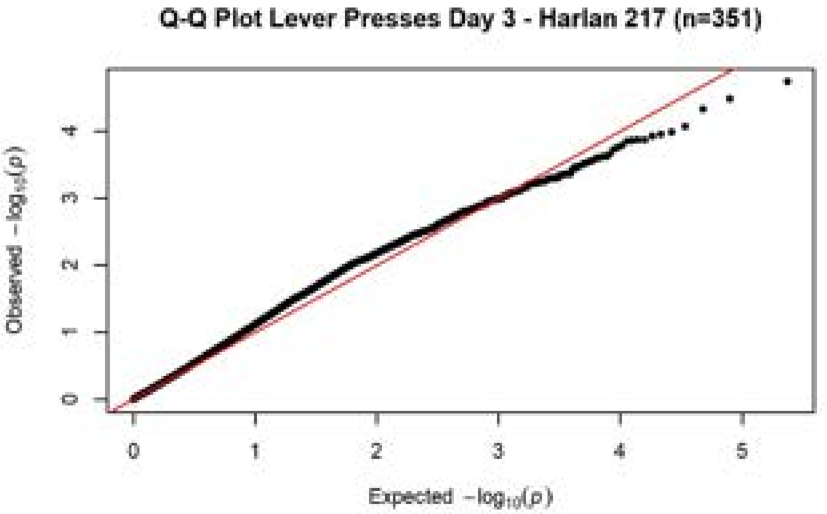
Q-Q Plot Lever Presses Day 3 - Harlan 217 (n=351)
Observed −log10(p)
Expected −log10(p)

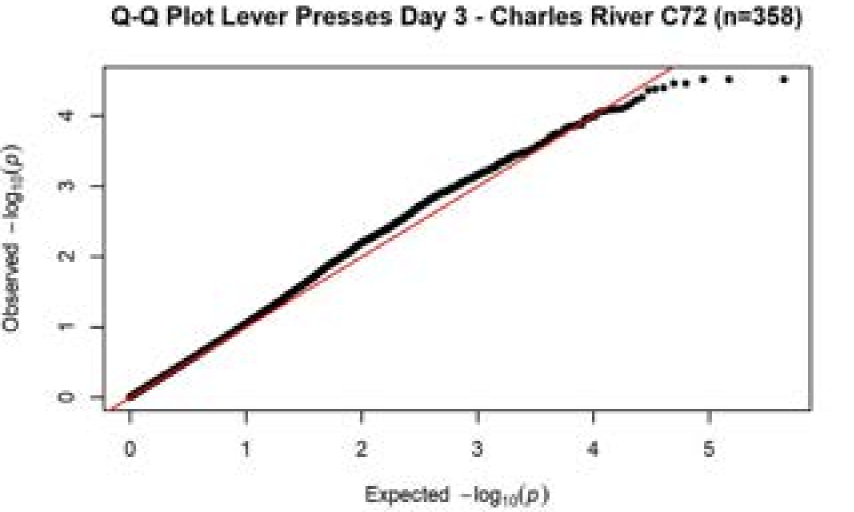
Q-Q Plot Lever Presses Day 3 - Charles River C72 (n=358)
Observed −log10(p)
Expected −log10(p)

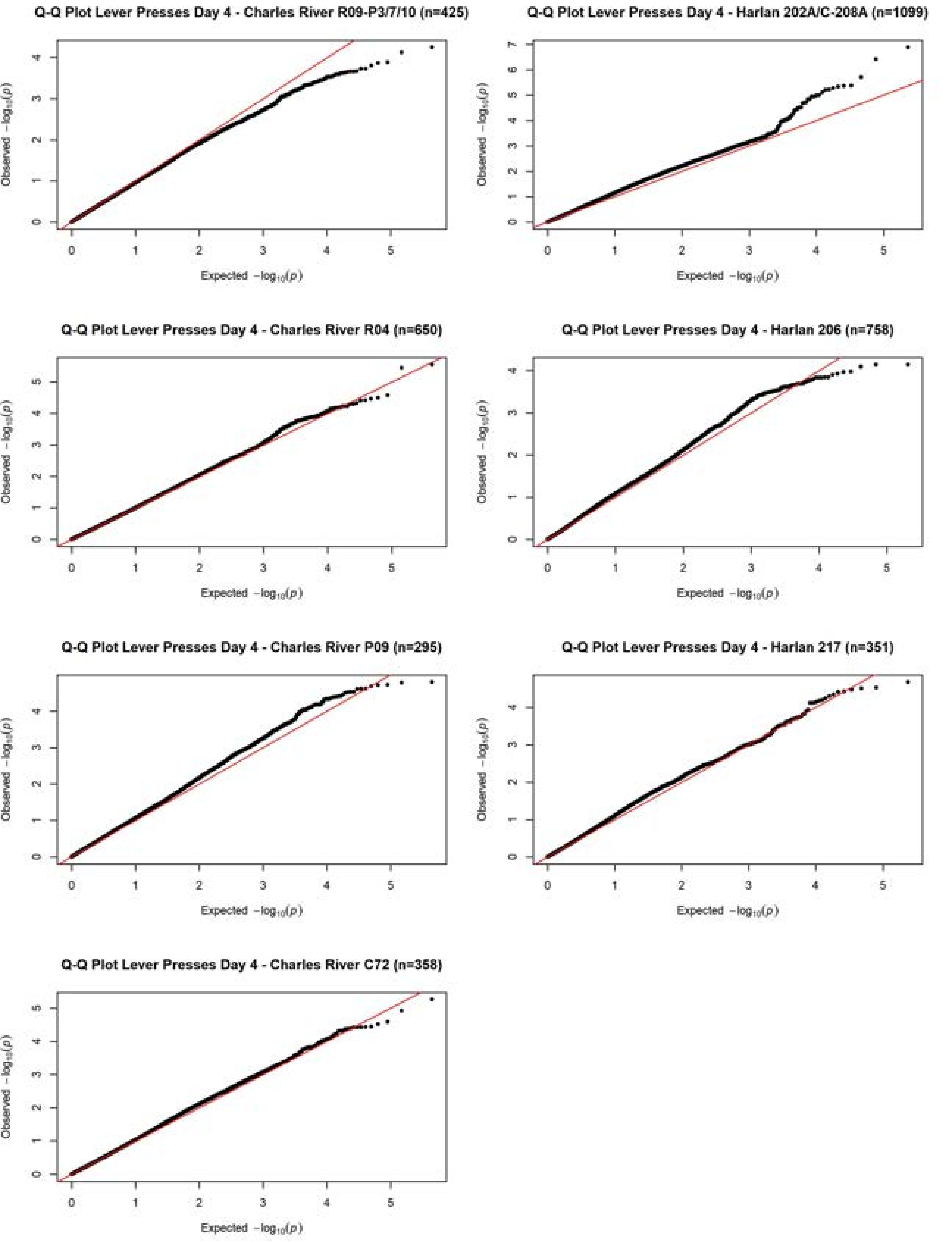
Q-Q Plot Lever Presses Day 4 - Charles River R09-P3/7/10 (n=425)
Q-Q Plot Lever Presses Day 4 - Harlan 202A/C-208A (n=1099)
Q-Q Plot Lever Presses Day 4 - Charles River R04 (n=650)
Q-Q Plot Lever Presses Day 4 - Harlan 206 (n=758)
Q-Q Plot Lever Presses Day 4 - Charles River P09 (n=295)
Q-Q Plot Lever Presses Day 4 - Harlan 217 (n=351)
Q-Q Plot Lever Presses Day 4 - Charles River C72 (n=358)
Observed $-\log_{10}(p)$
Expected $-\log_{10}(p)$

Q-Q Plot Lever Presses Day 5 - Charles River R09-P3/7/10 (n=425)

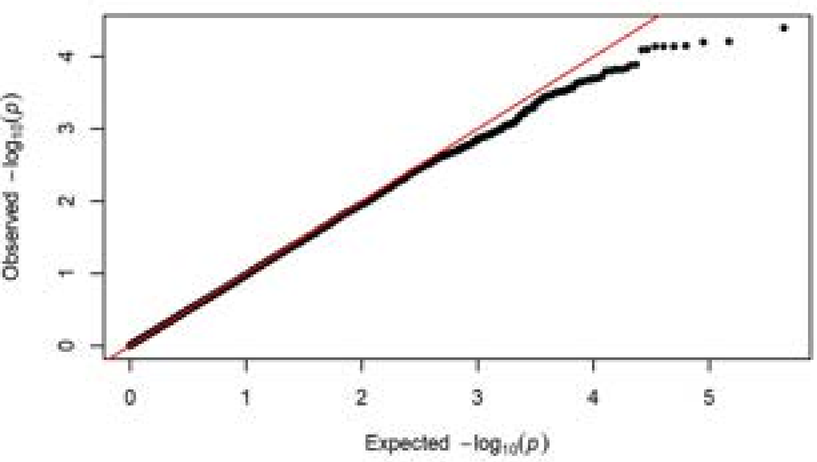

Q-Q Plot Lever Presses Day 5 - Harlan 202A/C-208A (n=1099)

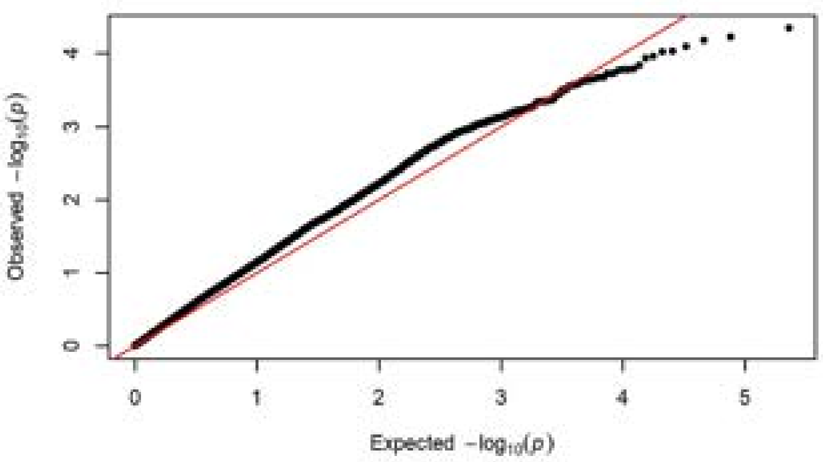

Q-Q Plot Lever Presses Day 5 - Charles River R04 (n=650)

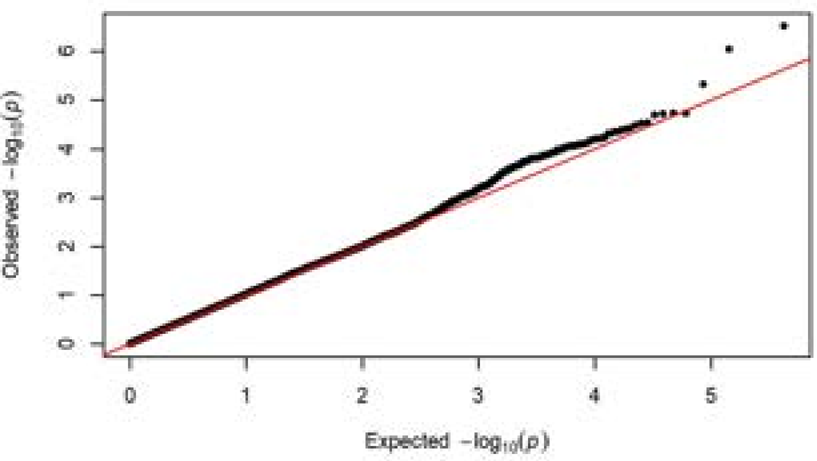

Q-Q Plot Lever Presses Day 5 - Harlan 206 (n=758)

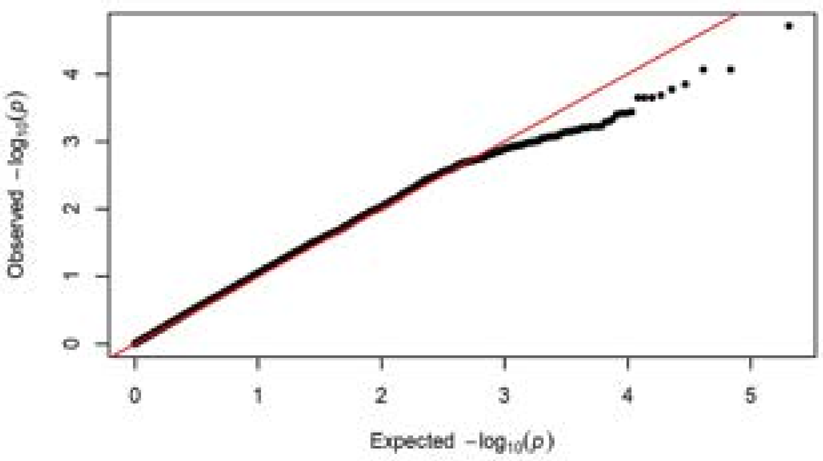

Q-Q Plot Lever Presses Day 5 - Charles River P09 (n=295)

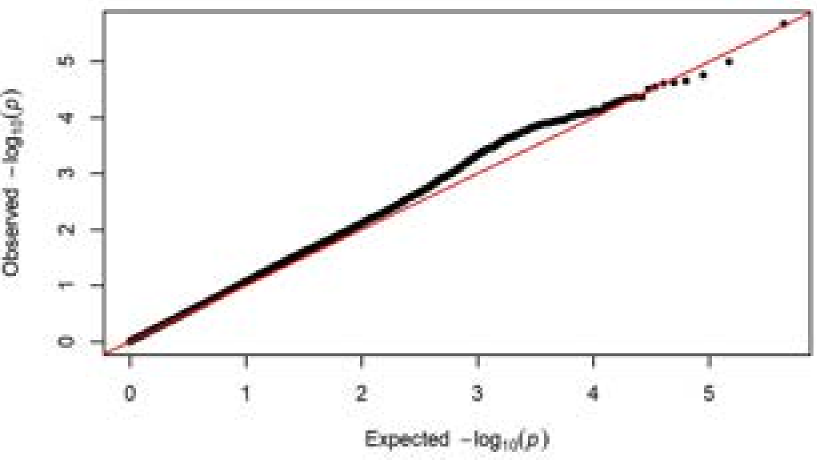

Q-Q Plot Lever Presses Day 5 - Harlan 217 (n=351)

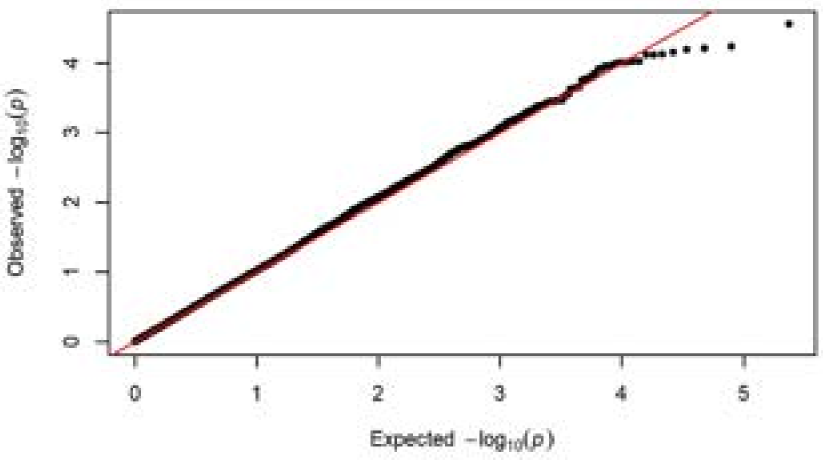

Q-Q Plot Lever Presses Day 5 - Charles River C72 (n=358)

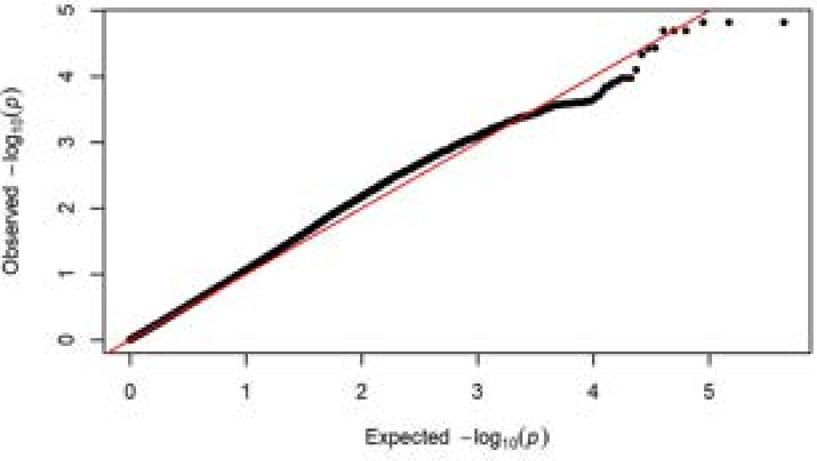

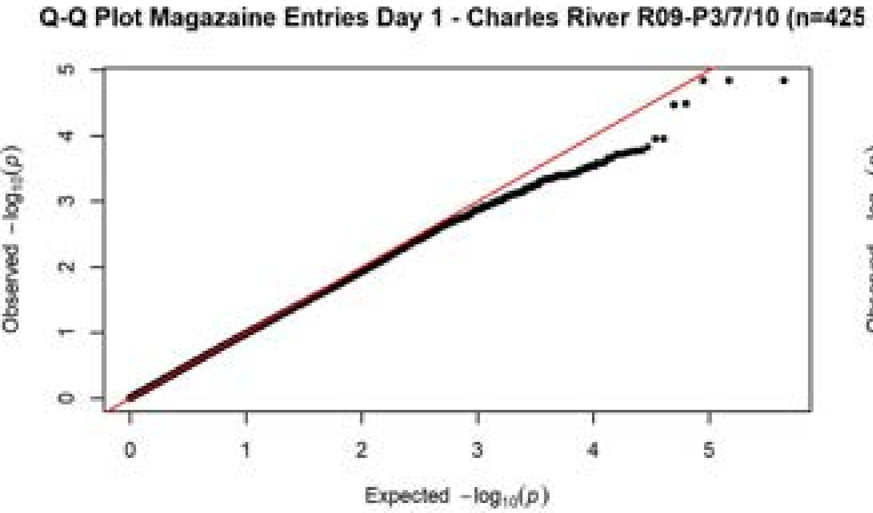
Q-Q Plot Magazaine Entries Day 1 - Charles River R09-P3/7/10 (n=425
Observed −log10(p)
Expected −log10(p)

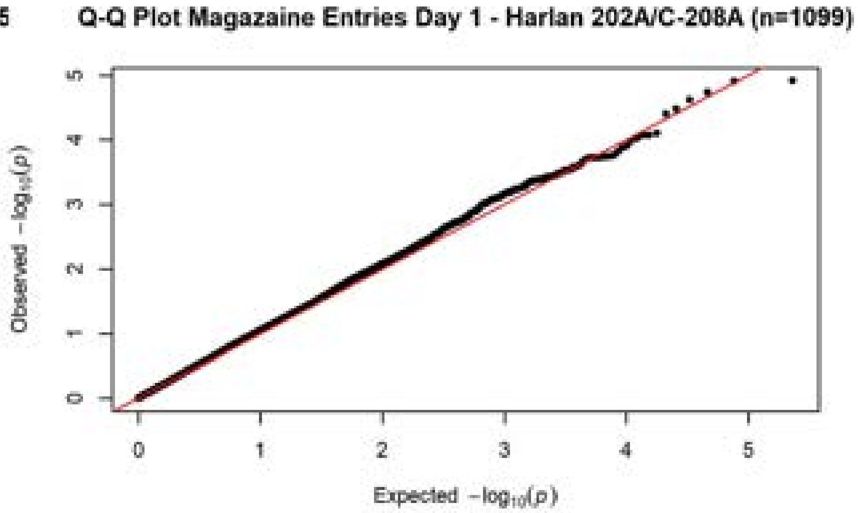
Q-Q Plot Magazaine Entries Day 1 - Harlan 202A/C-208A (n=1099)
Observed −log10(p)
Expected −log10(p)

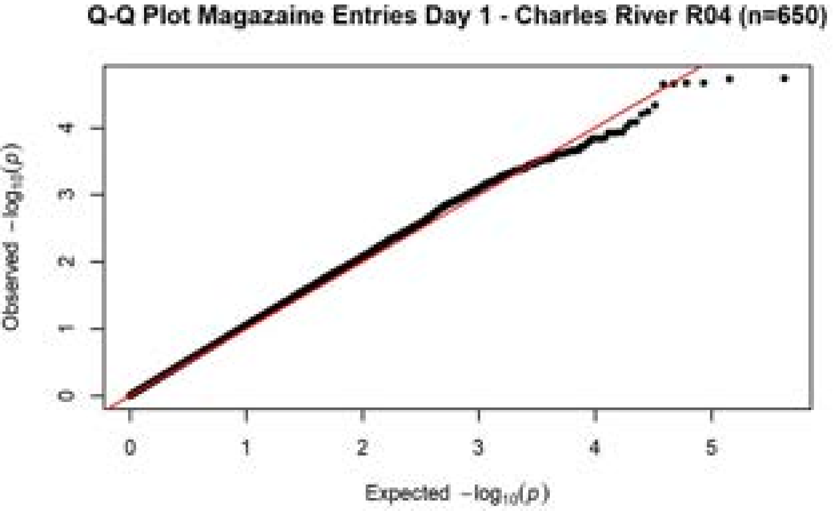
Q-Q Plot Magazaine Entries Day 1 - Charles River R04 (n=650)
Observed −log10(p)
Expected −log10(p)

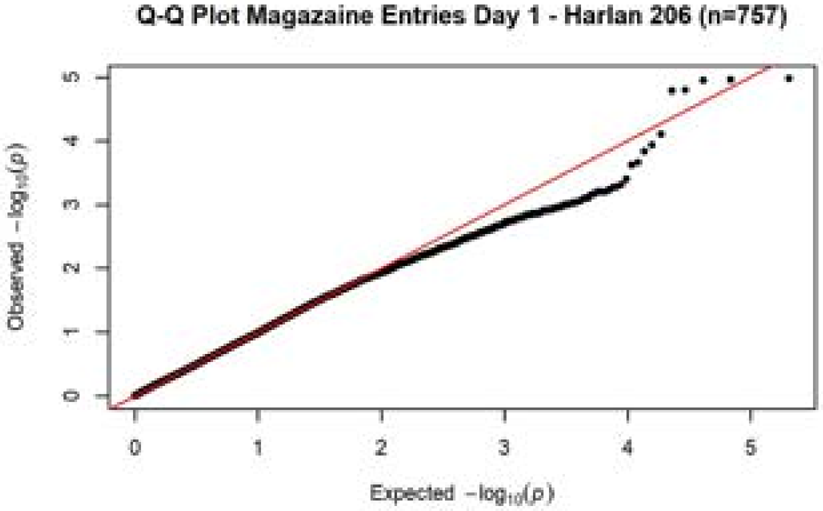
Q-Q Plot Magazaine Entries Day 1 - Harlan 206 (n=757)
Observed −log10(p)
Expected −log10(p)

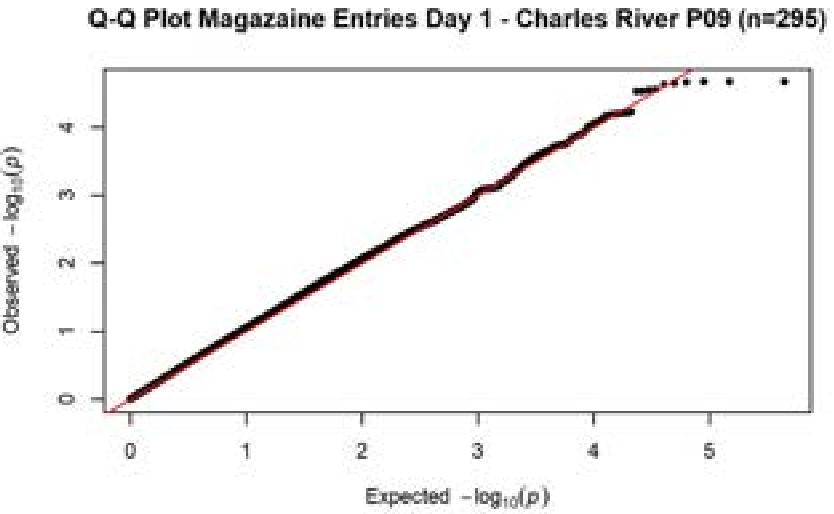
Q-Q Plot Magazaine Entries Day 1 - Charles River P09 (n=295)
Observed −log10(p)
Expected −log10(p)

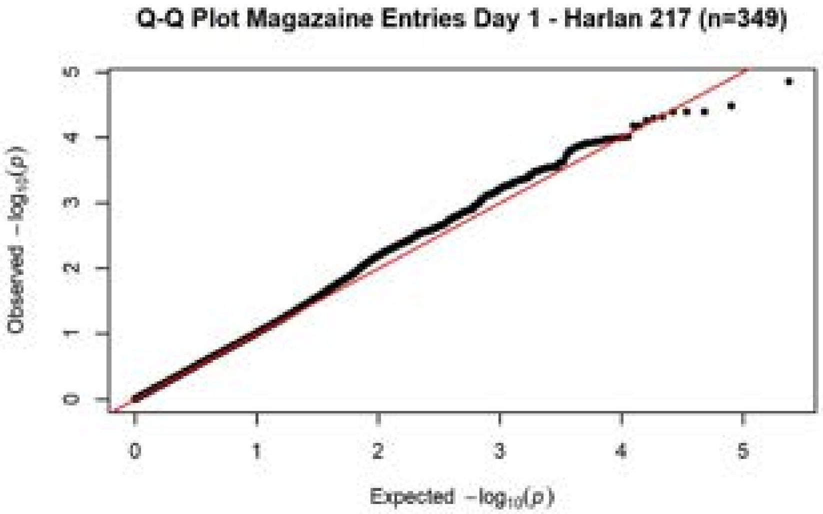
Q-Q Plot Magazaine Entries Day 1 - Harlan 217 (n=349)
Observed −log10(p)
Expected −log10(p)

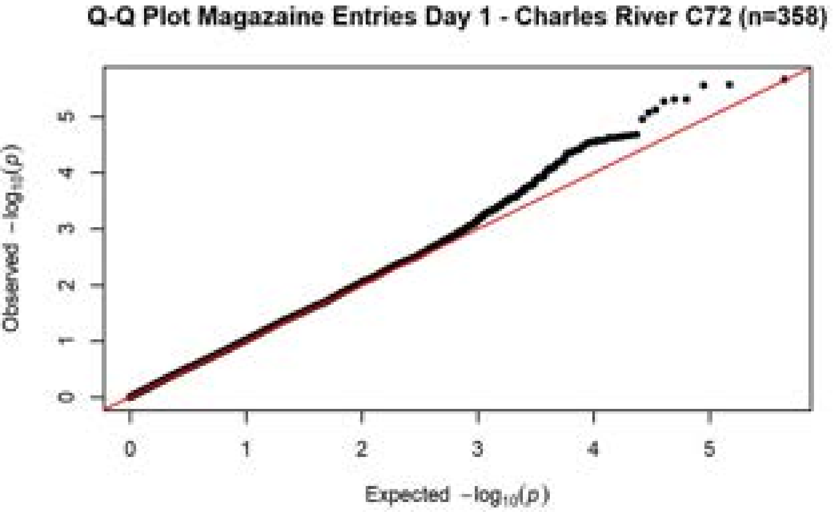
Q-Q Plot Magazaine Entries Day 1 - Charles River C72 (n=358)
Observed −log10(p)
Expected −log10(p)

Q-Q Plot Magazaine Entries Day 2 - Charles River R09-P3/7/10 (n=425

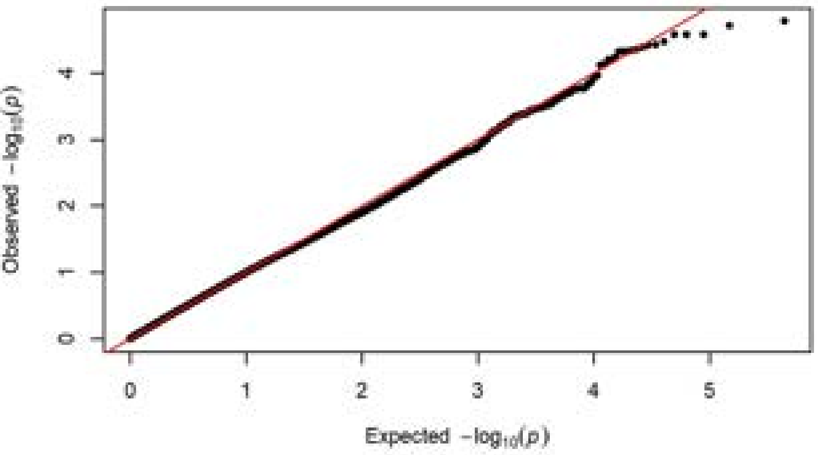

Q-Q Plot Magazaine Entries Day 2 - Harlan 202A/C-208A (n=1099)

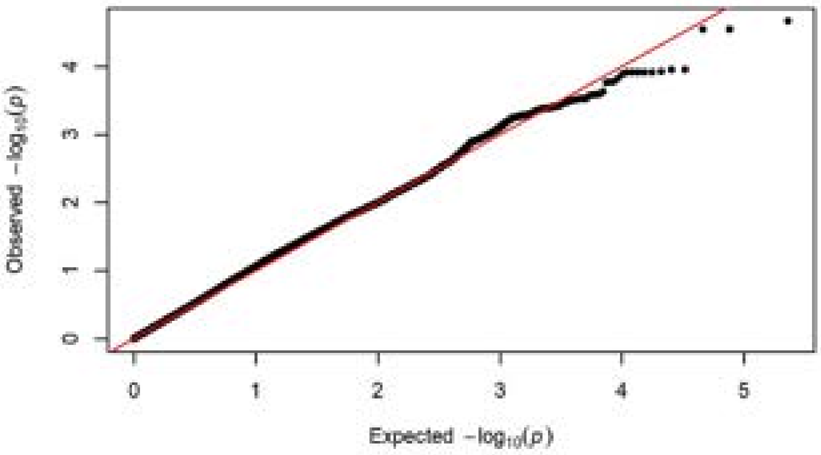

Q-Q Plot Magazaine Entries Day 2 - Charles River R04 (n=650)

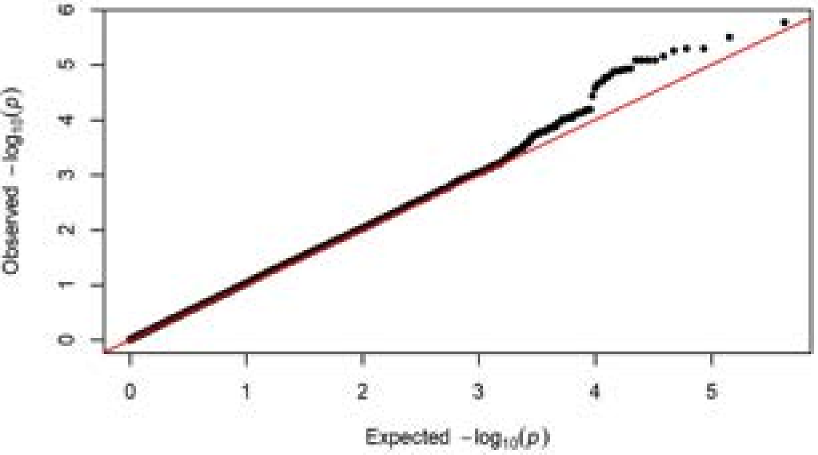

Q-Q Plot Magazaine Entries Day 2 - Harlan 206 (n=758)

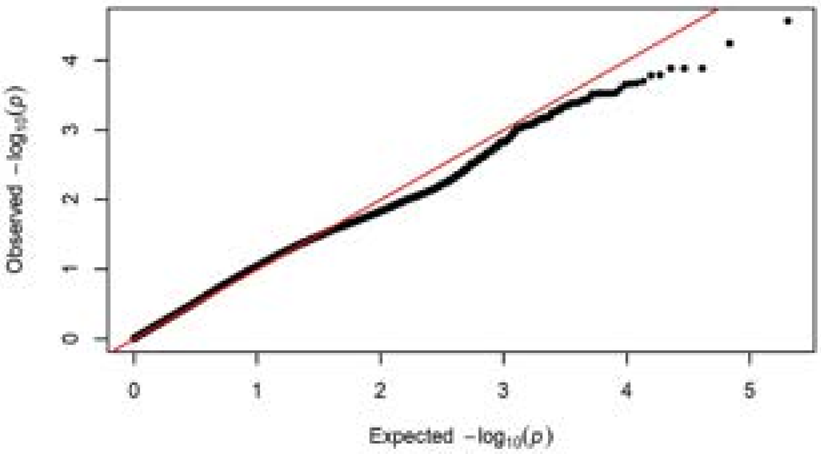

Q-Q Plot Magazaine Entries Day 2 - Charles River P09 (n=295)

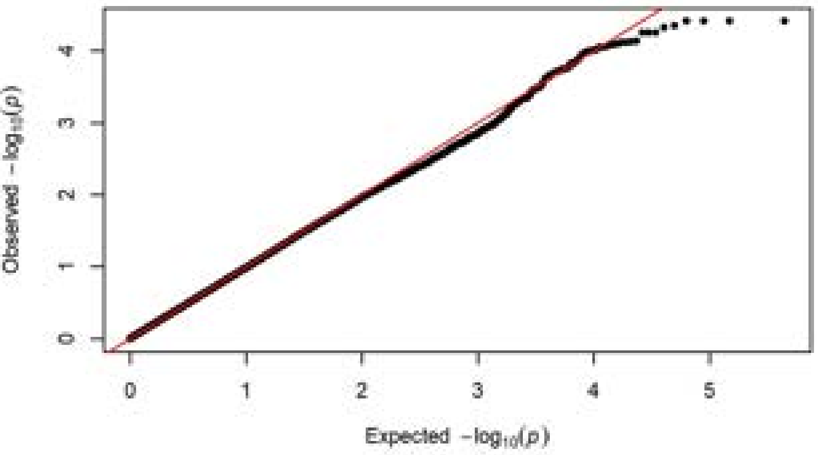

Q-Q Plot Magazaine Entries Day 2 - Harlan 217 (n=350)

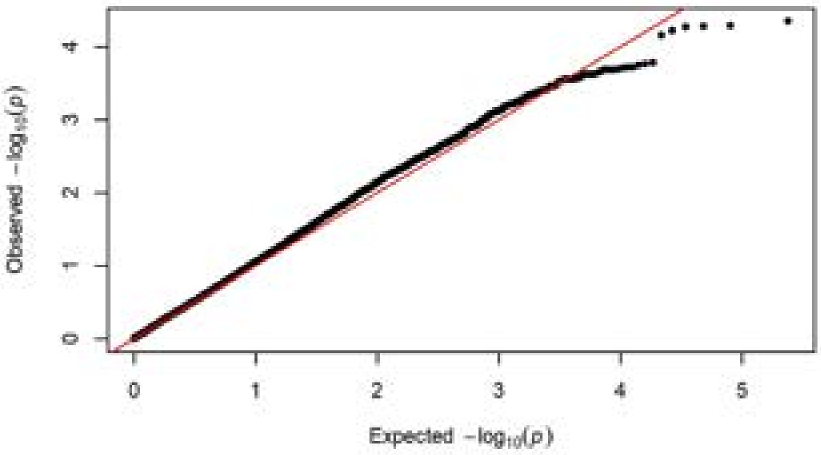

Q-Q Plot Magazaine Entries Day 2 - Charles River C72 (n=356)

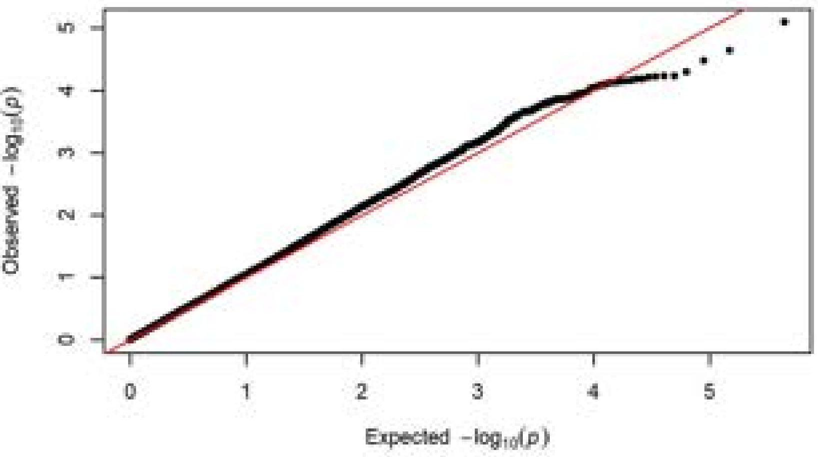

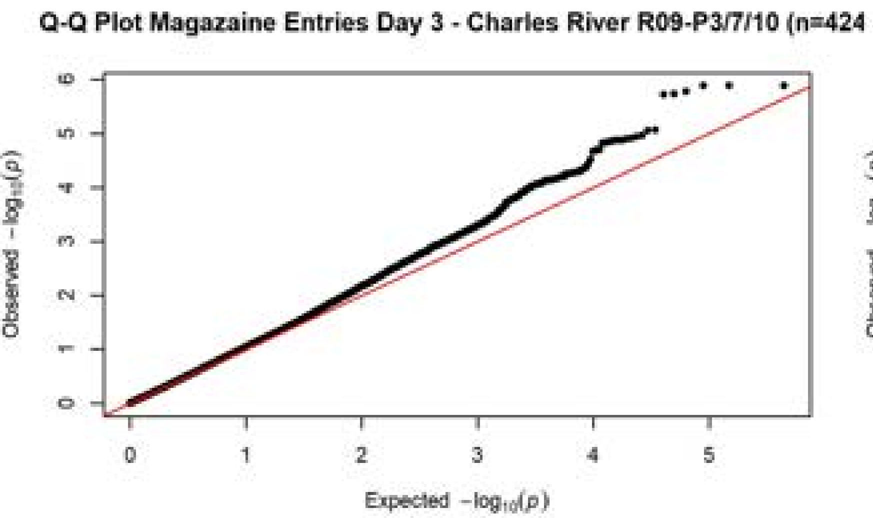
Q-Q Plot Magazaine Entries Day 3 - Charles River R09-P3/7/10 (n=424
Observed $-\log_{10}(p)$
Expected $-\log_{10}(p)$

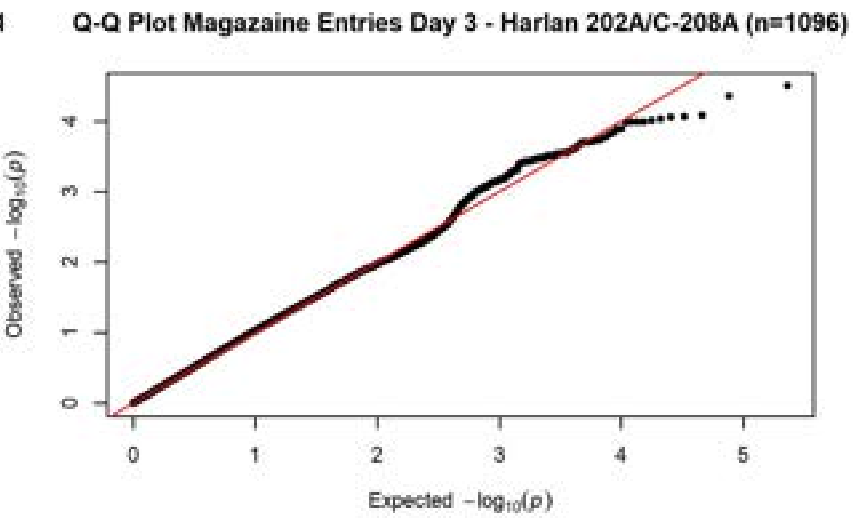
Q-Q Plot Magazaine Entries Day 3 - Harlan 202A/C-208A (n=1096)
Observed $-\log_{10}(p)$
Expected $-\log_{10}(p)$

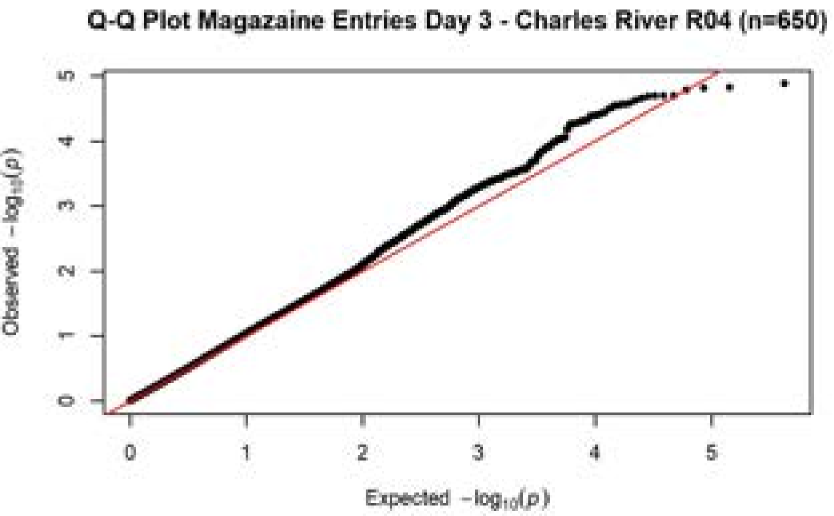
Q-Q Plot Magazaine Entries Day 3 - Charles River R04 (n=650)
Observed $-\log_{10}(p)$
Expected $-\log_{10}(p)$

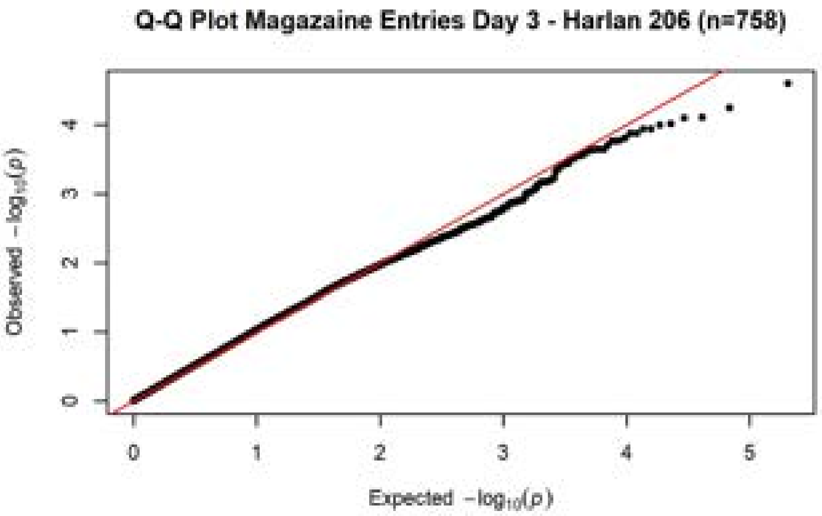
Q-Q Plot Magazaine Entries Day 3 - Harlan 206 (n=758)
Observed $-\log_{10}(p)$
Expected $-\log_{10}(p)$

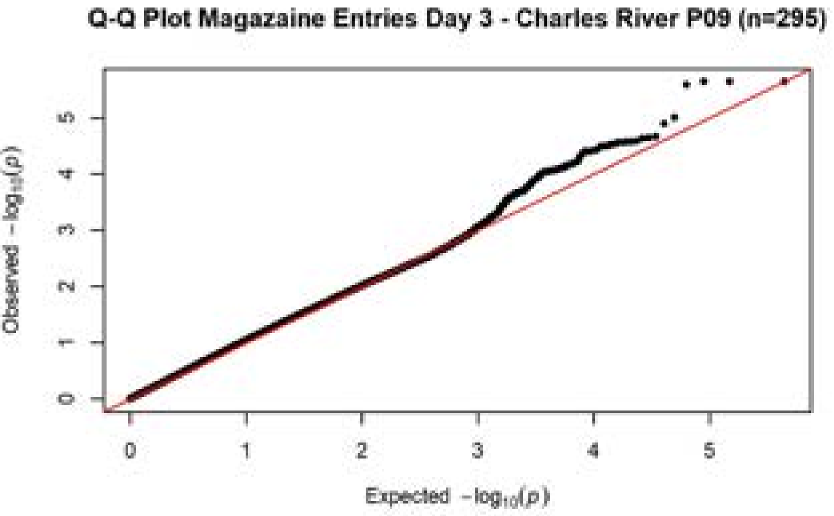
Q-Q Plot Magazaine Entries Day 3 - Charles River P09 (n=295)
Observed $-\log_{10}(p)$
Expected $-\log_{10}(p)$

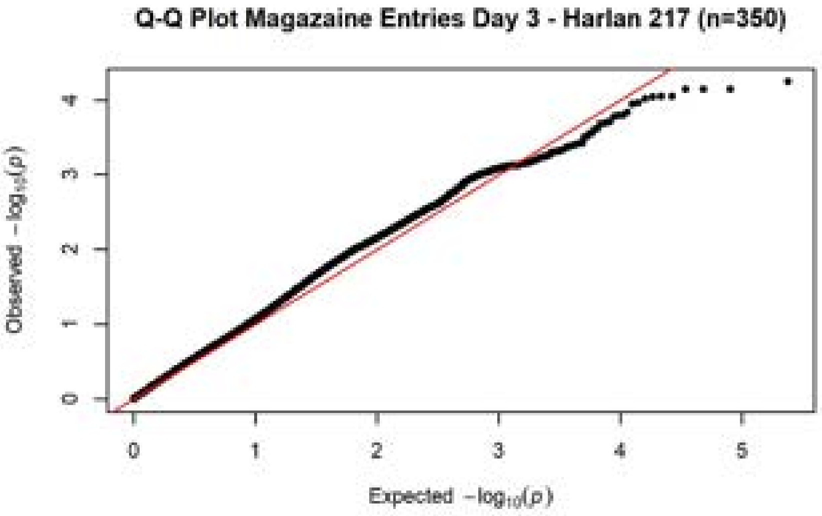
Q-Q Plot Magazaine Entries Day 3 - Harlan 217 (n=350)
Observed $-\log_{10}(p)$
Expected $-\log_{10}(p)$

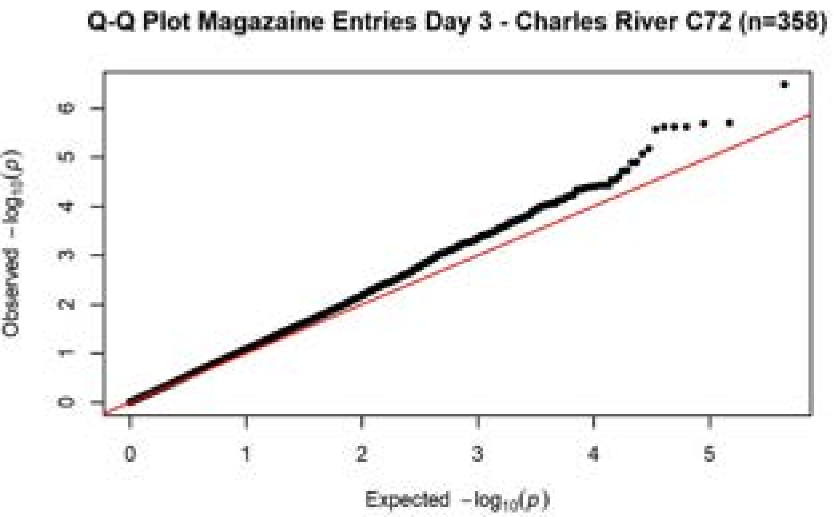
Q-Q Plot Magazaine Entries Day 3 - Charles River C72 (n=358)
Observed $-\log_{10}(p)$
Expected $-\log_{10}(p)$

Q-Q Plot Magazaine Entries Day 4 - Charles River R09-P3/7/10 (n=425

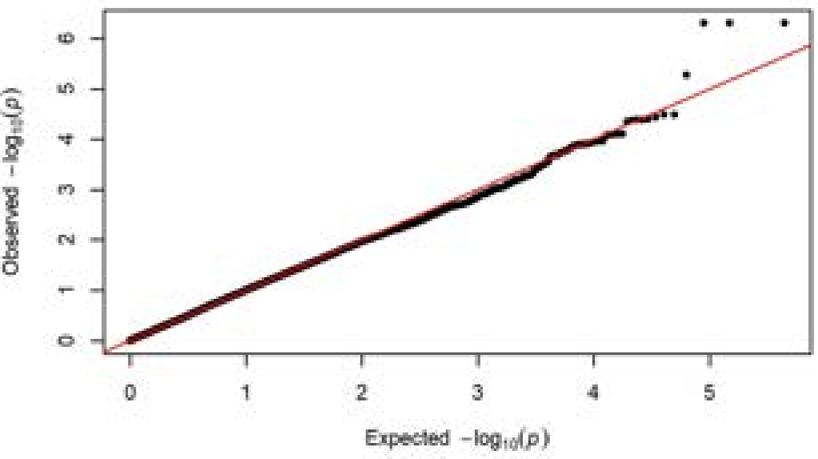

Q-Q Plot Magazaine Entries Day 4 - Harlan 202A/C-208A (n=1099)

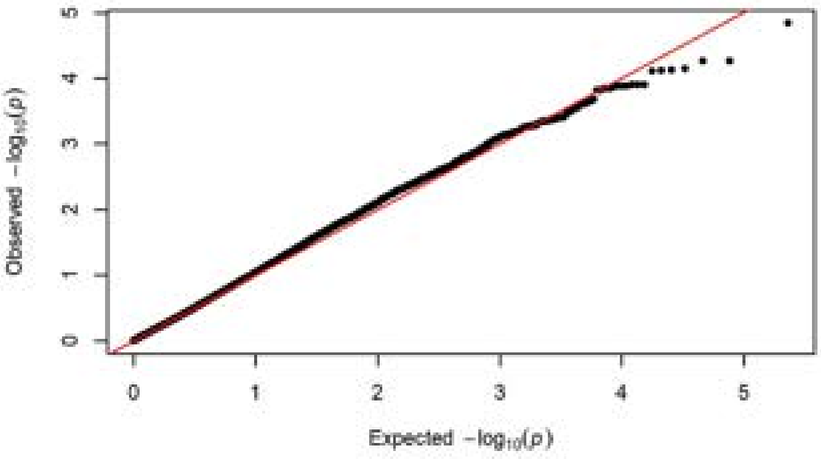

Q-Q Plot Magazaine Entries Day 4 - Charles River R04 (n=650)

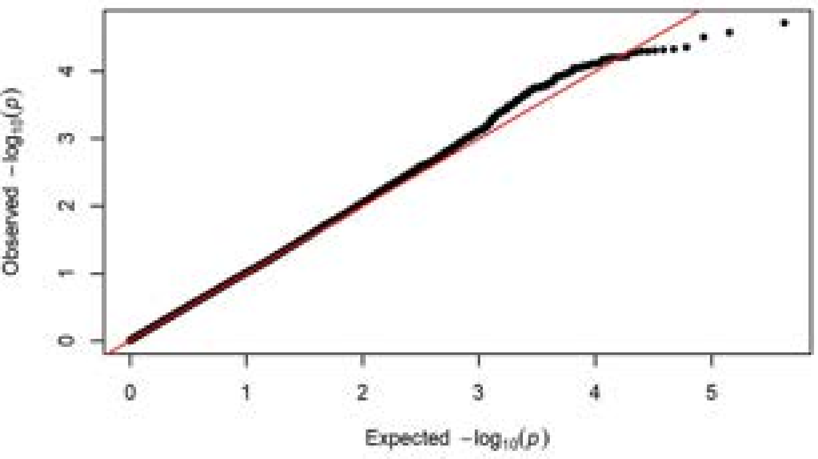

Q-Q Plot Magazaine Entries Day 4 - Harlan 206 (n=758)

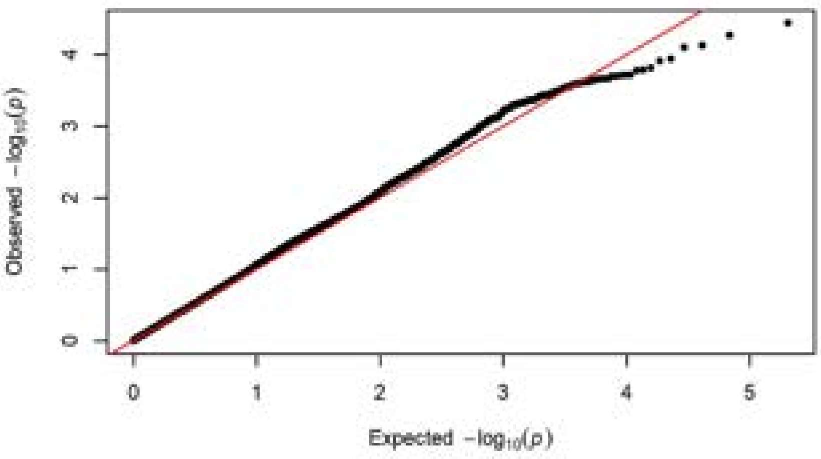

Q-Q Plot Magazaine Entries Day 4 - Charles River P09 (n=295)

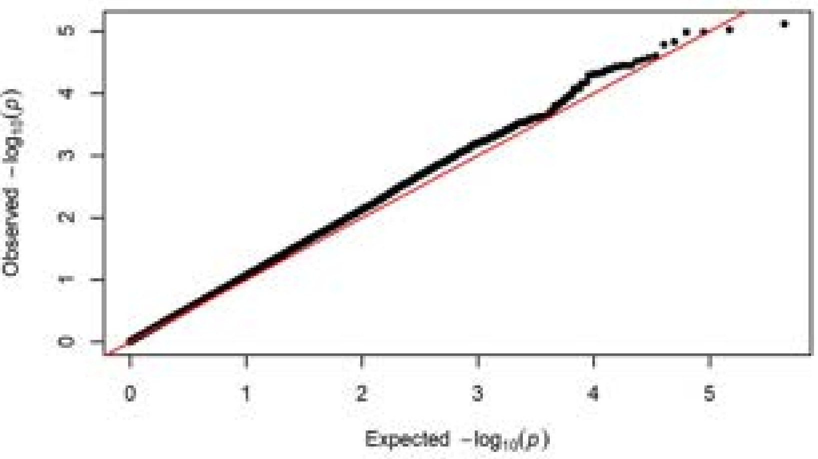

Q-Q Plot Magazaine Entries Day 4 - Harlan 217 (n=351)

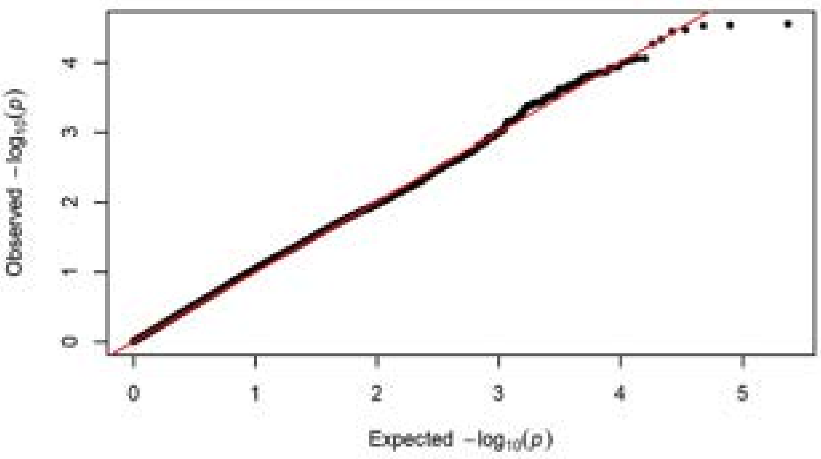

Q-Q Plot Magazaine Entries Day 4 - Charles River C72 (n=358)

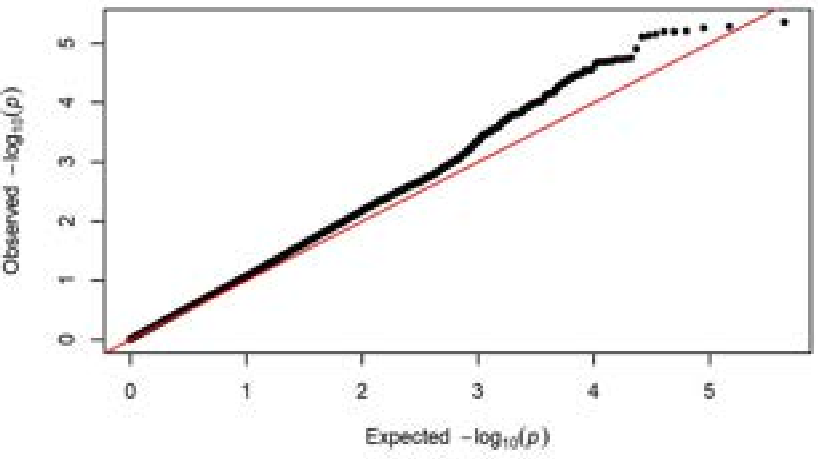

Q-Q Plot Magazaine Entries Day 5 - Charles River R09-P3/7/10 (n=425

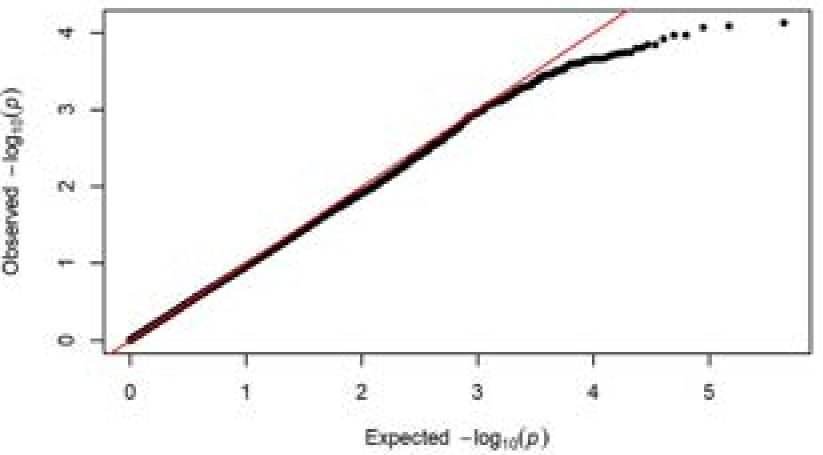

Q-Q Plot Magazaine Entries Day 5 - Harlan 202A/C-208A (n=1099)

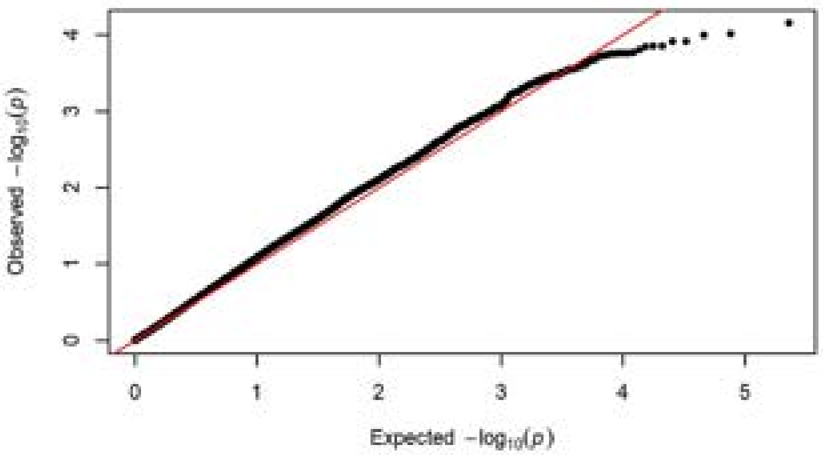

Q-Q Plot Magazaine Entries Day 5 - Charles River R04 (n=650)

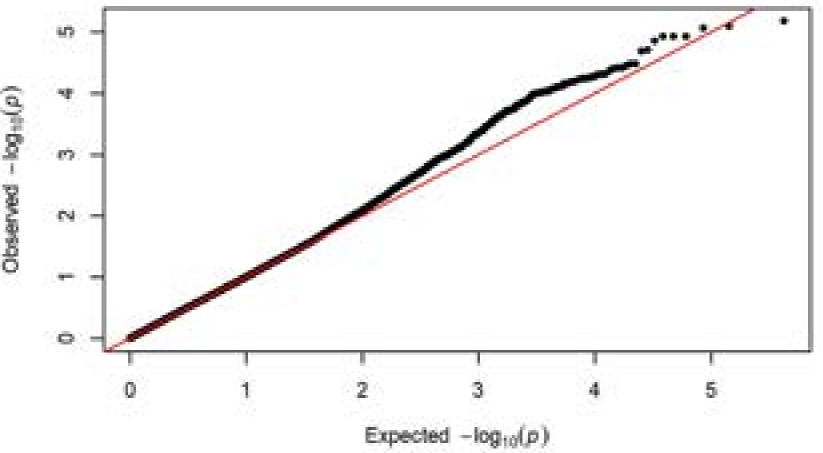

Q-Q Plot Magazaine Entries Day 5 - Harlan 206 (n=758)

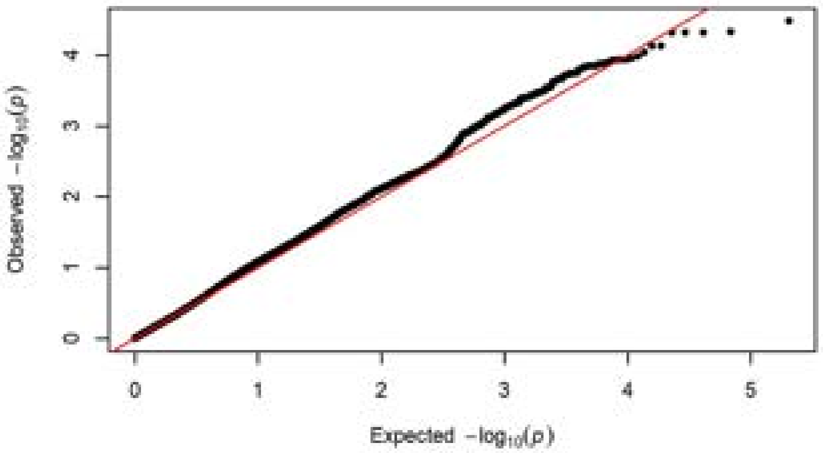

Q-Q Plot Magazaine Entries Day 5 - Charles River P09 (n=295)

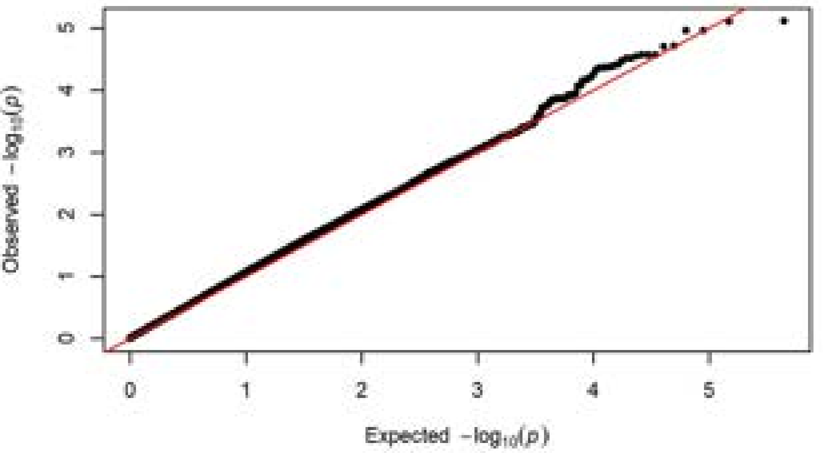

Q-Q Plot Magazaine Entries Day 5 - Harlan 217 (n=351)

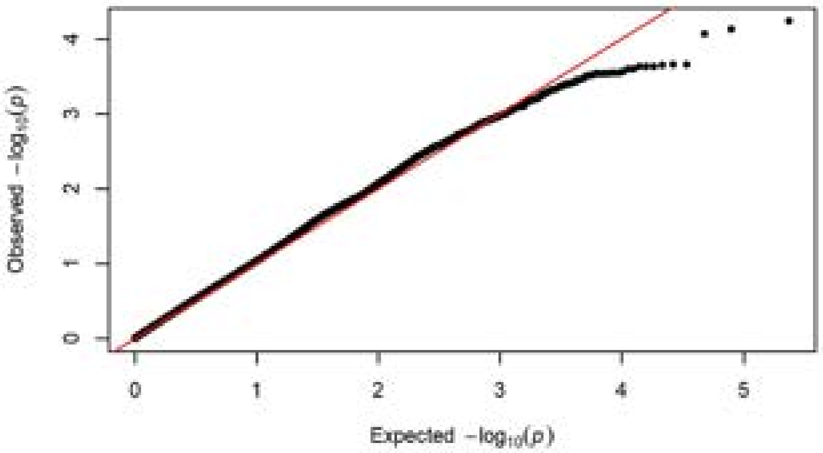

Q-Q Plot Magazaine Entries Day 5 - Charles River C72 (n=358)

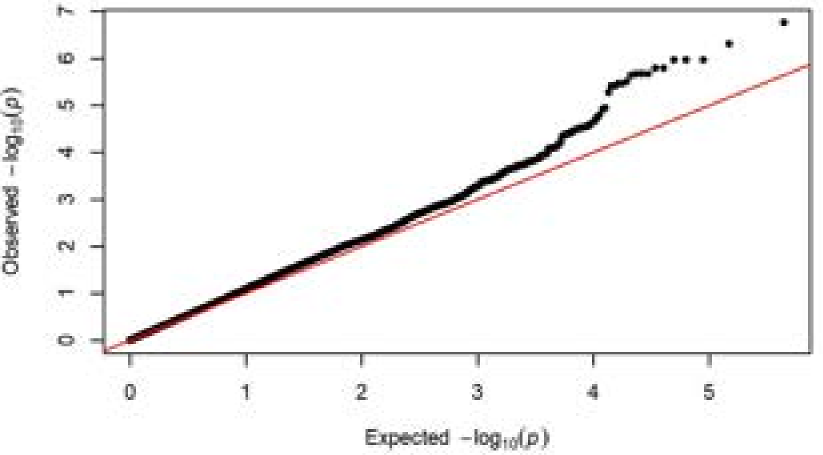

Q-Q Plot Magazine Entries NCS Day 1 - Charles River R09-P3/7/10 (n=4

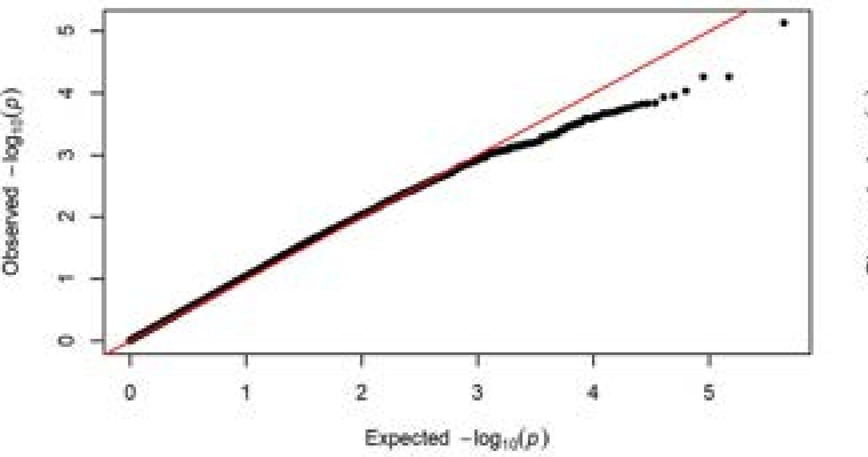

Q-Q Plot Magazine Entries NCS Day 1 - Harlan 202A/C-208A (n=1099)

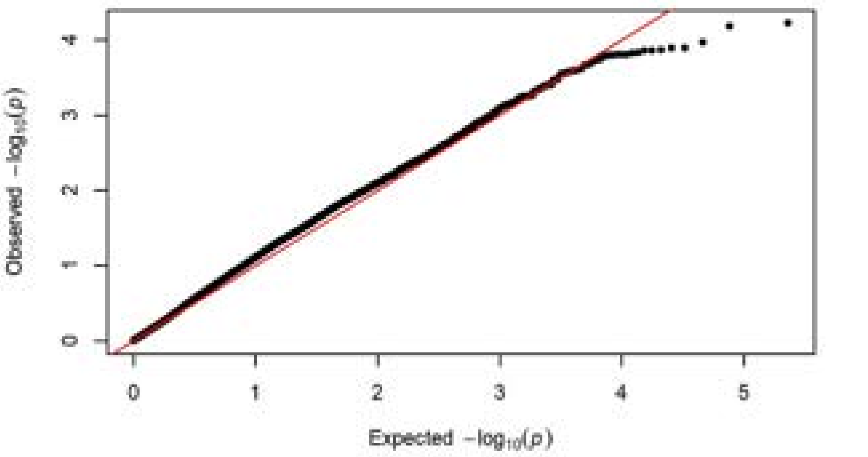

Q-Q Plot Magazine Entries NCS Day 1 - Charles River R04 (n=650)

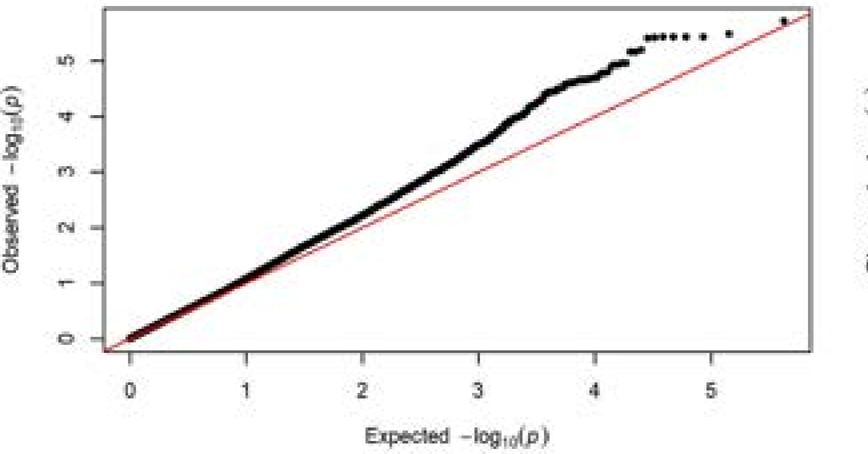

Q-Q Plot Magazine Entries NCS Day 1 - Harlan 206 (n=757)

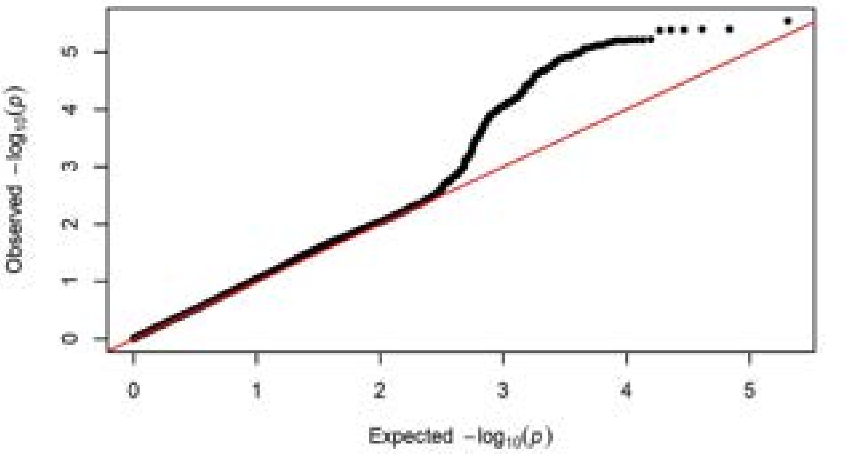

Q-Q Plot Magazine Entries NCS Day 1 - Charles River P09 (n=295)

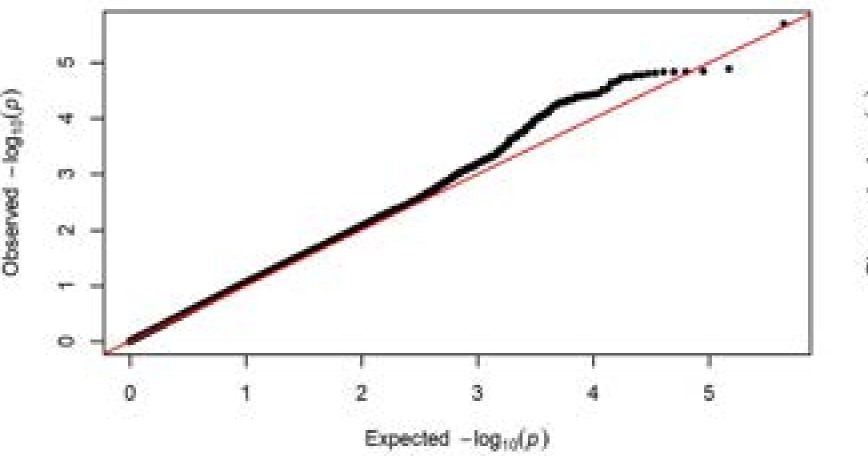

Q-Q Plot Magazine Entries NCS Day 1 - Harlan 217 (n=349)

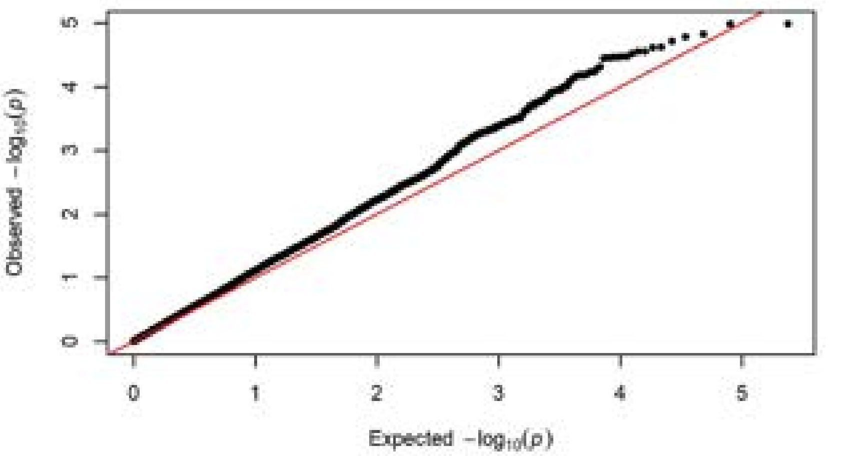

Q-Q Plot Magazine Entries NCS Day 1 - Charles River C72 (n=358)

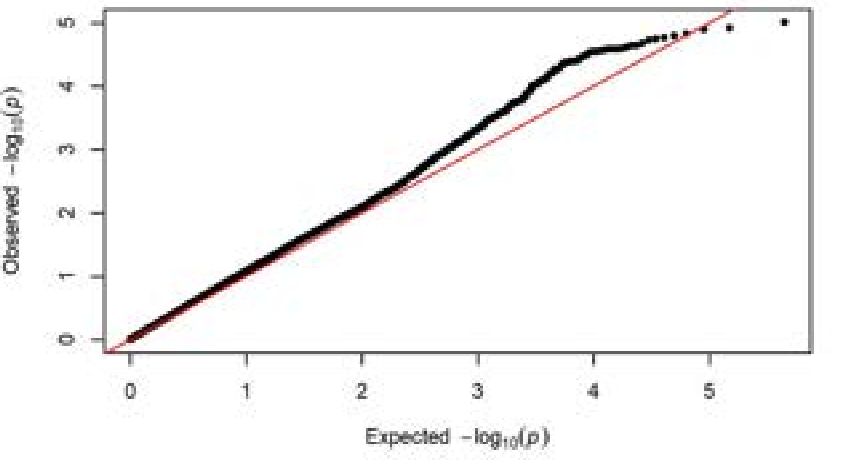

Q-Q Plot Magazine Entries NCS Day 2 - Charles River R09-P3/7/10 (n=4

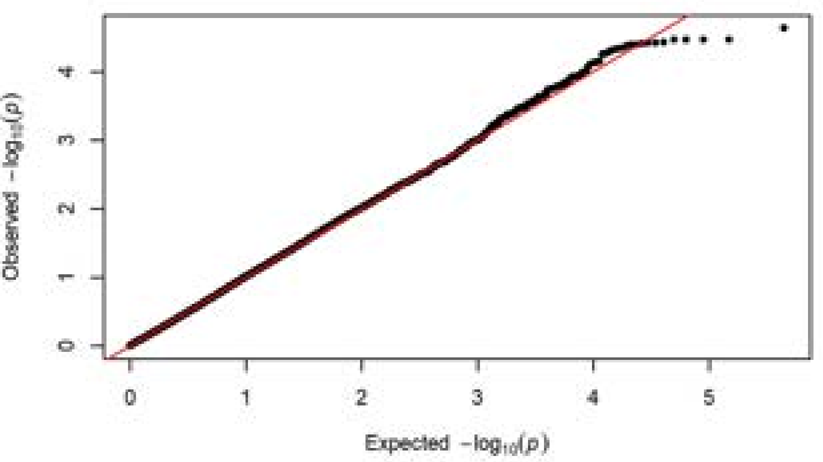

Q-Q Plot Magazine Entries NCS Day 2 - Harlan 202A/C-208A (n=1099)

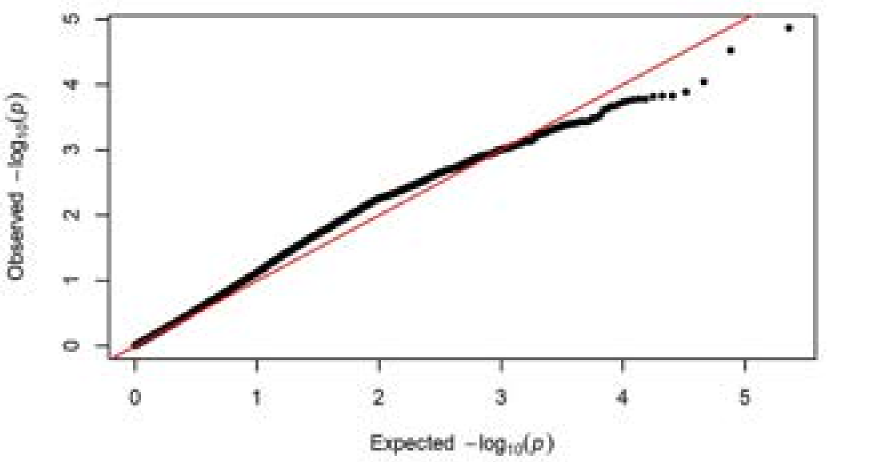

Q-Q Plot Magazine Entries NCS Day 2 - Charles River R04 (n=650)

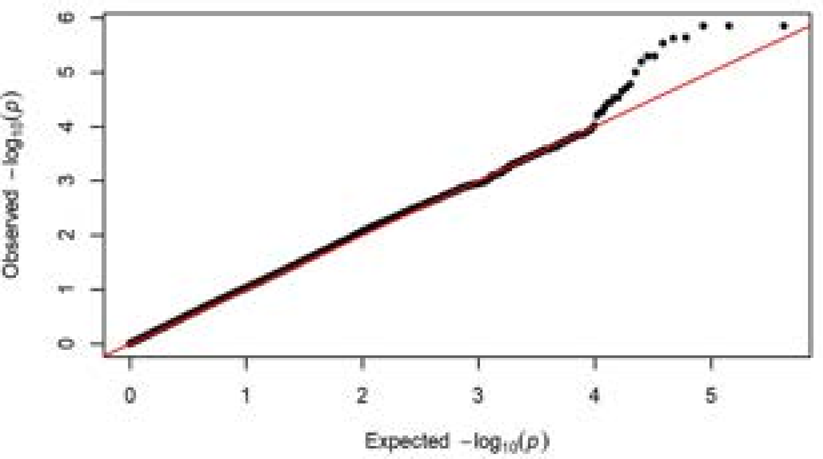

Q-Q Plot Magazine Entries NCS Day 2 - Harlan 206 (n=758)

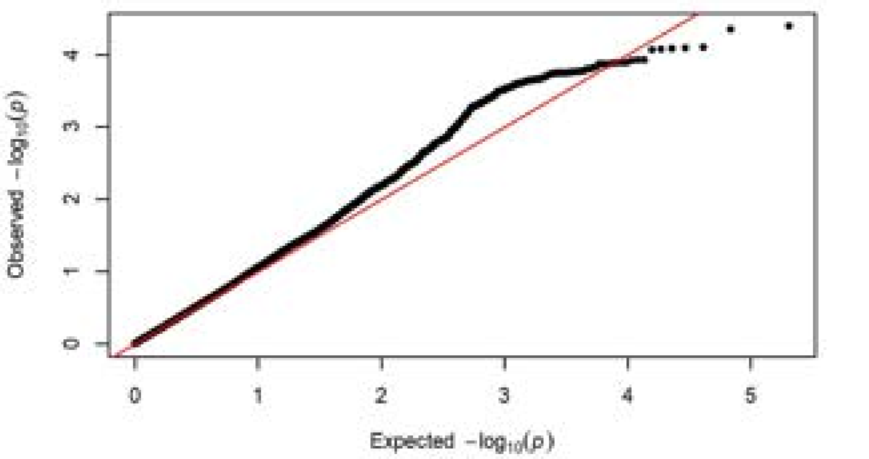

Q-Q Plot Magazine Entries NCS Day 2 - Charles River P09 (n=295)

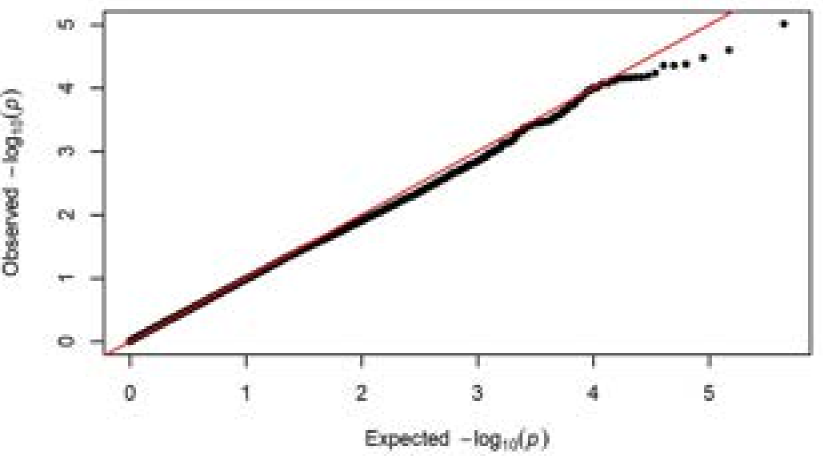

Q-Q Plot Magazine Entries NCS Day 2 - Harlan 217 (n=350)

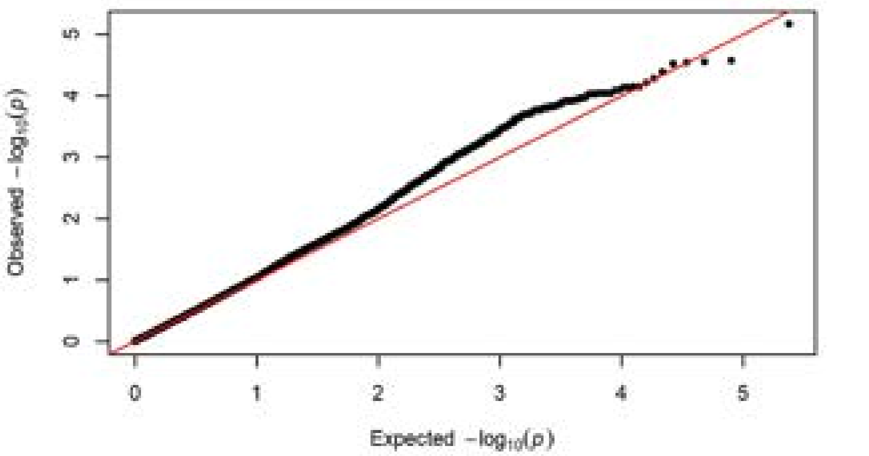

Q-Q Plot Magazine Entries NCS Day 2 - Charles River C72 (n=356)

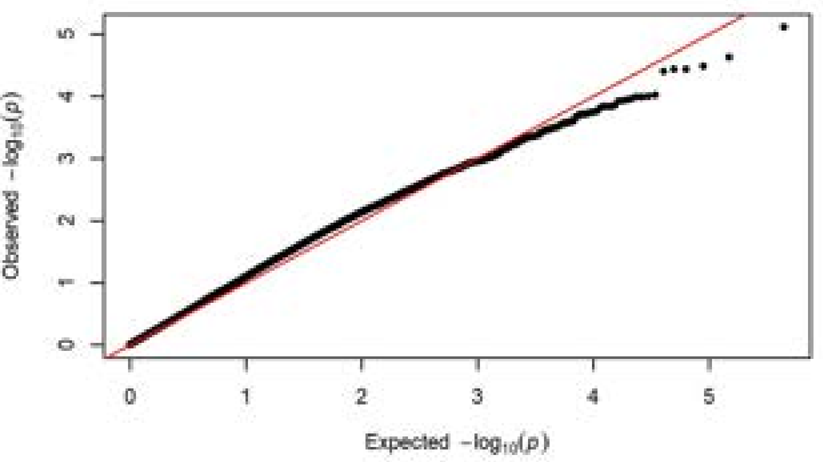

Q-Q Plot Magazine Entries NCS Day 3 - Charles River R09-P3/7/10 (n=4

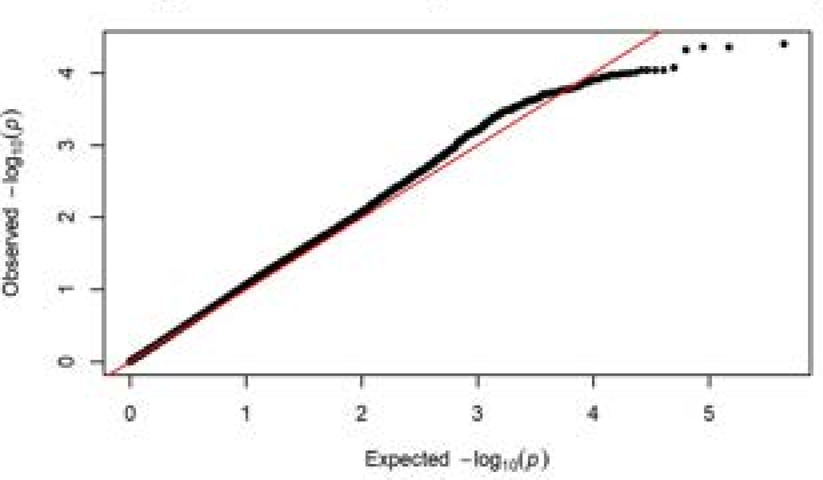

Q-Q Plot Magazine Entries NCS Day 3 - Harlan 202A/C-208A (n=1096)

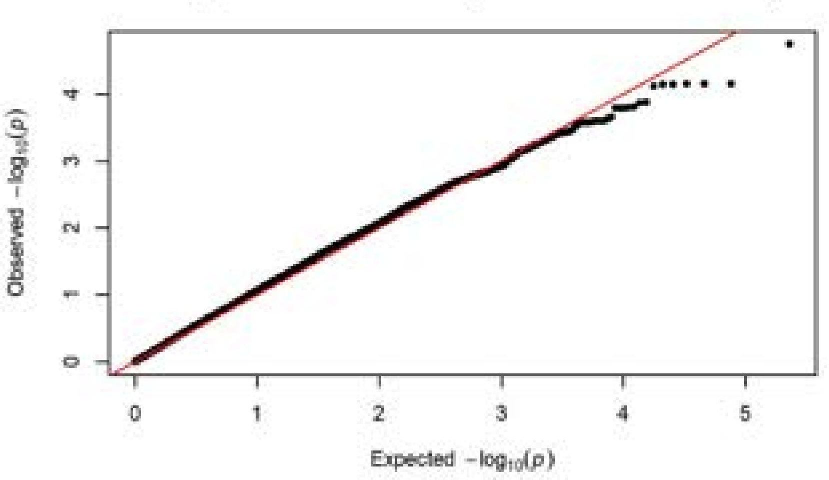

Q-Q Plot Magazine Entries NCS Day 3 - Charles River R04 (n=650)

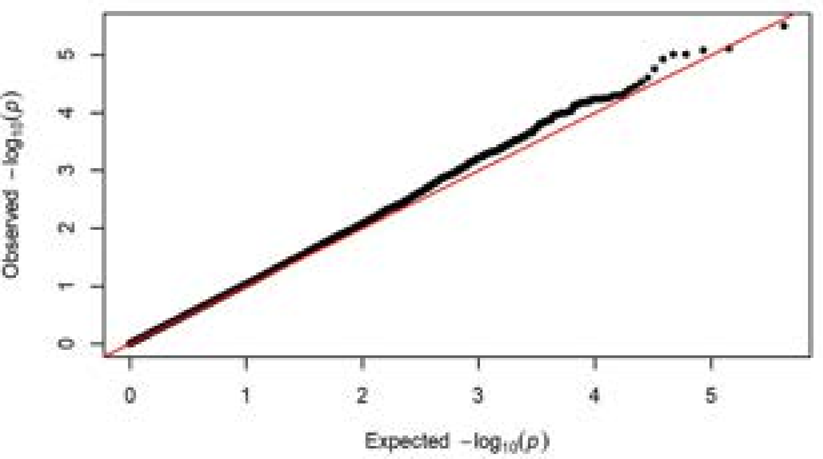

Q-Q Plot Magazine Entries NCS Day 3 - Harlan 206 (n=758)

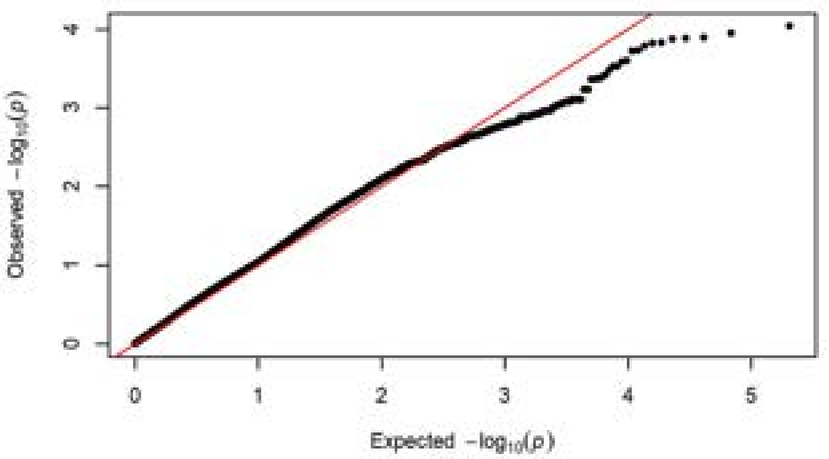

Q-Q Plot Magazine Entries NCS Day 3 - Charles River P09 (n=295)

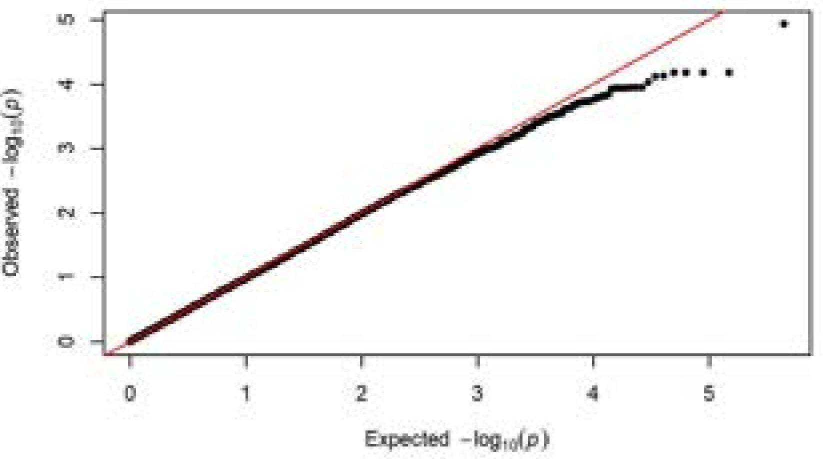

Q-Q Plot Magazine Entries NCS Day 3 - Harlan 217 (n=350)

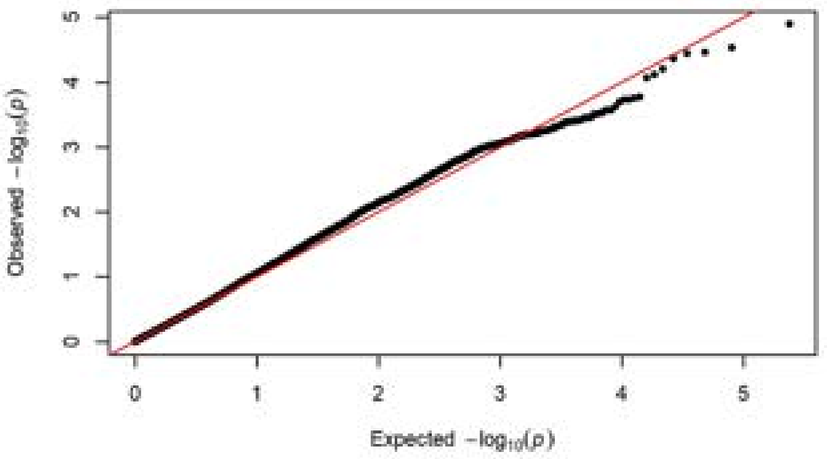

Q-Q Plot Magazine Entries NCS Day 3 - Charles River C72 (n=358)

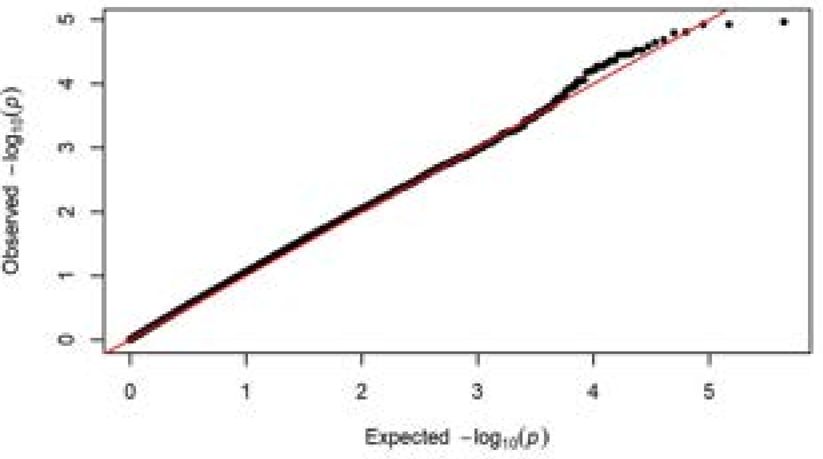

Q-Q Plot Magazine Entries NCS Day 4 - Charles River R09-P3/7/10 (n=4

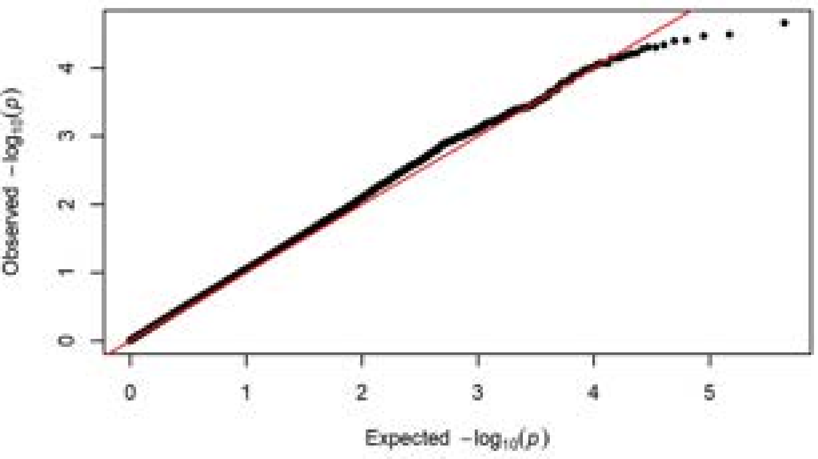

Q-Q Plot Magazine Entries NCS Day 4 - Harlan 202A/C-208A (n=1099)

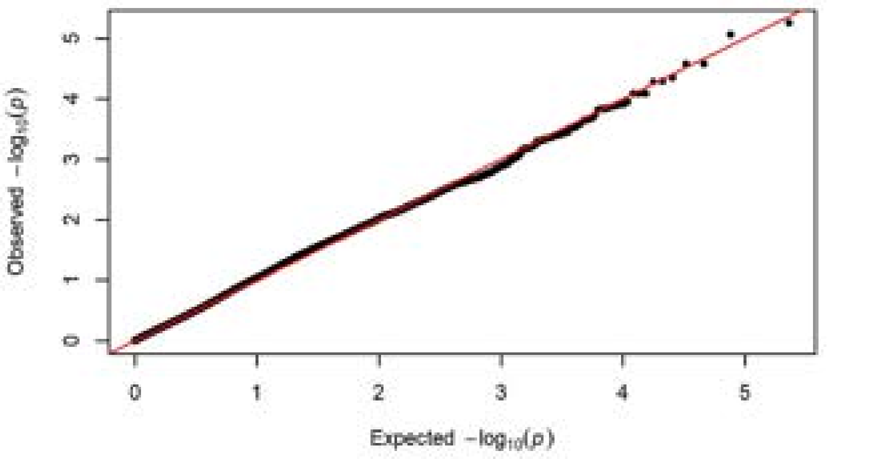

Q-Q Plot Magazine Entries NCS Day 4 - Charles River R04 (n=650)

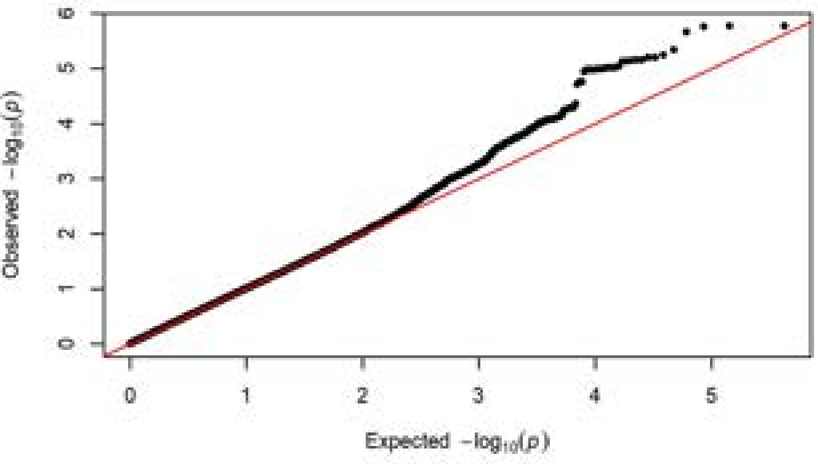

Q-Q Plot Magazine Entries NCS Day 4 - Harlan 206 (n=758)

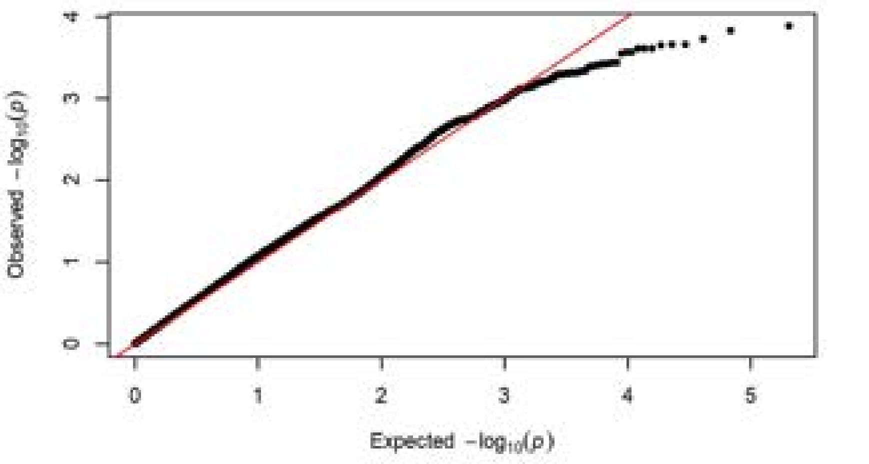

Q-Q Plot Magazine Entries NCS Day 4 - Charles River P09 (n=295)

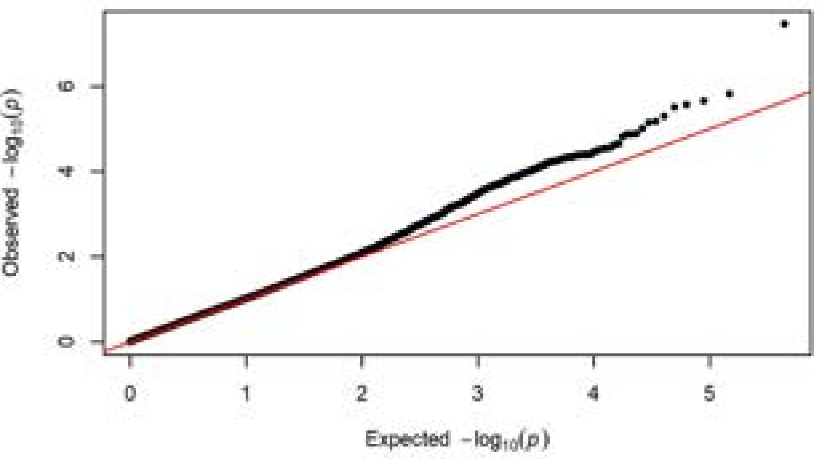

Q-Q Plot Magazine Entries NCS Day 4 - Harlan 217 (n=351)

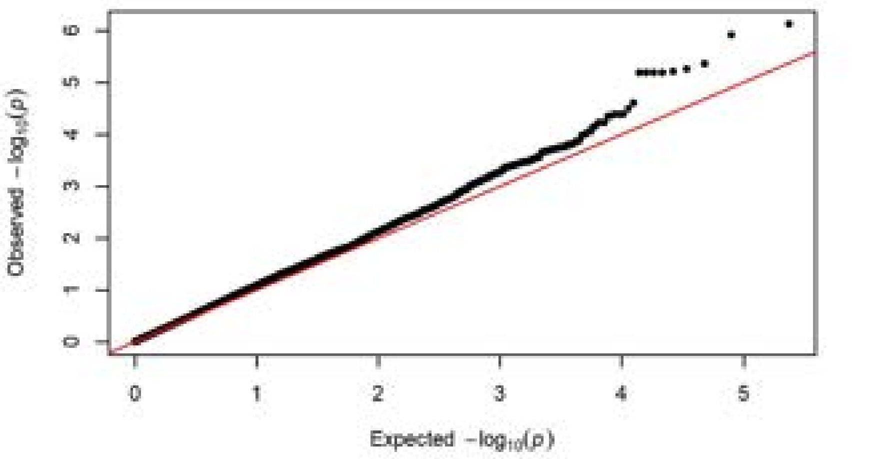

Q-Q Plot Magazine Entries NCS Day 4 - Charles River C72 (n=358)

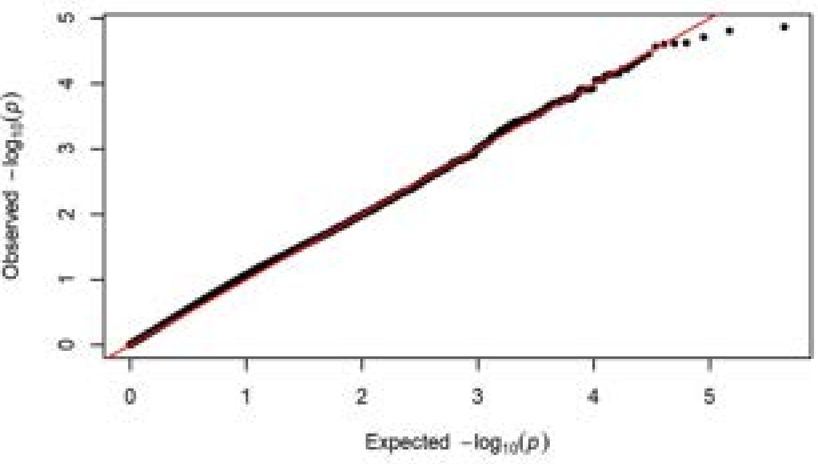

Q-Q Plot Magazine Entries NCS Day 5 - Charles River R09-P3/7/10 (n=4

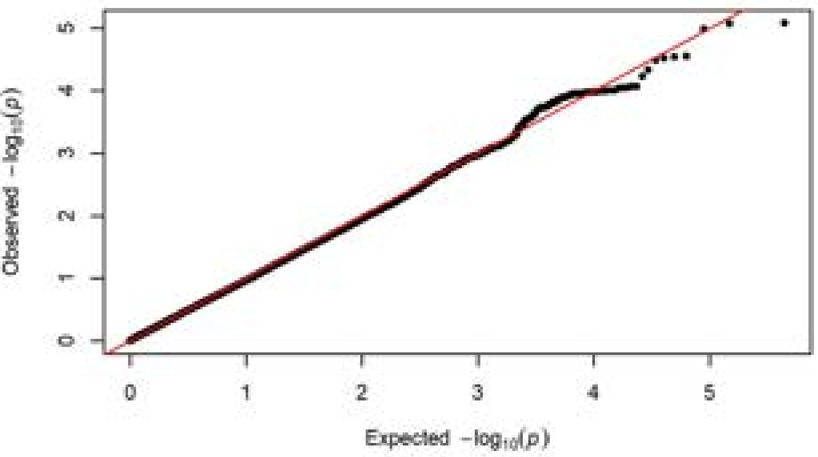

Q-Q Plot Magazine Entries NCS Day 5 - Harlan 202A/C-208A (n=1099)

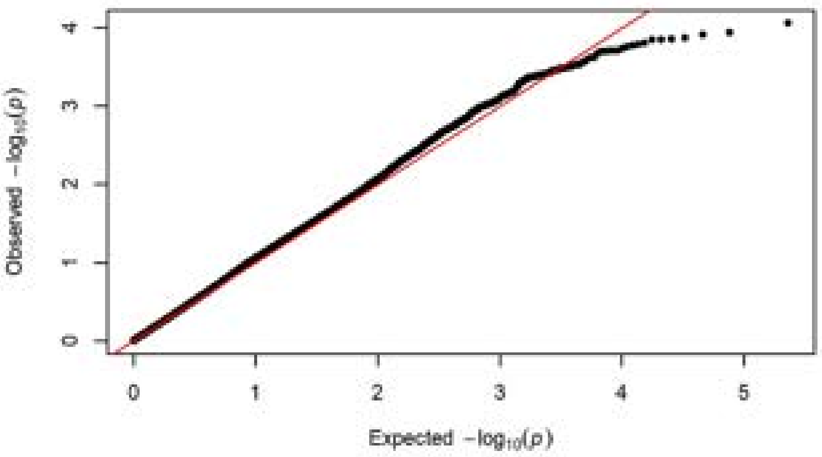

Q-Q Plot Magazine Entries NCS Day 5 - Charles River R04 (n=650)

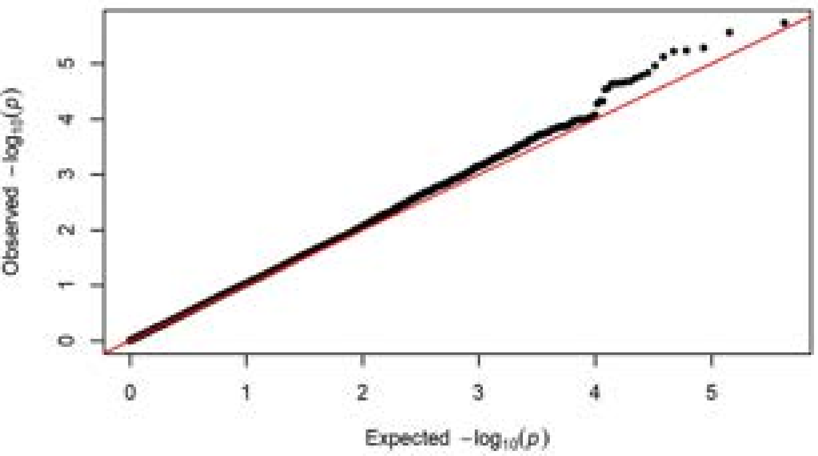

Q-Q Plot Magazine Entries NCS Day 5 - Harlan 206 (n=758)

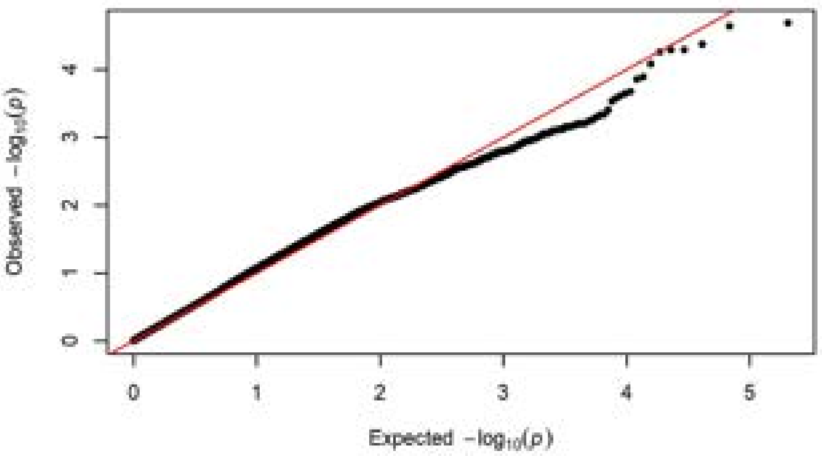

Q-Q Plot Magazine Entries NCS Day 5 - Charles River P09 (n=295)

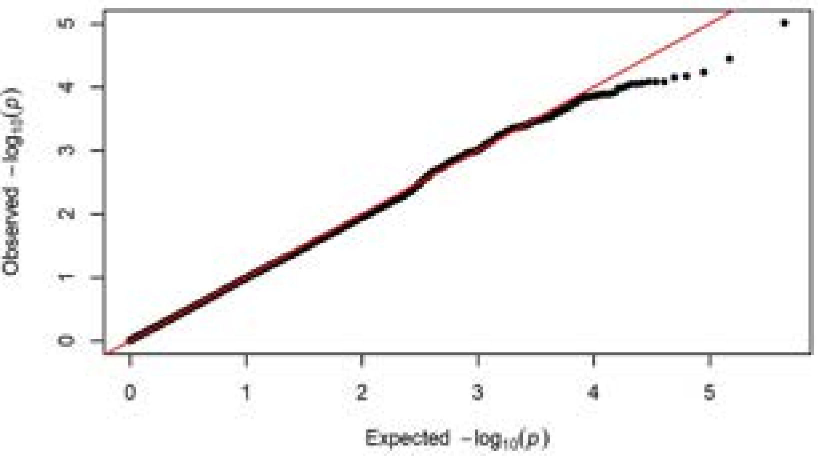

Q-Q Plot Magazine Entries NCS Day 5 - Harlan 217 (n=351)

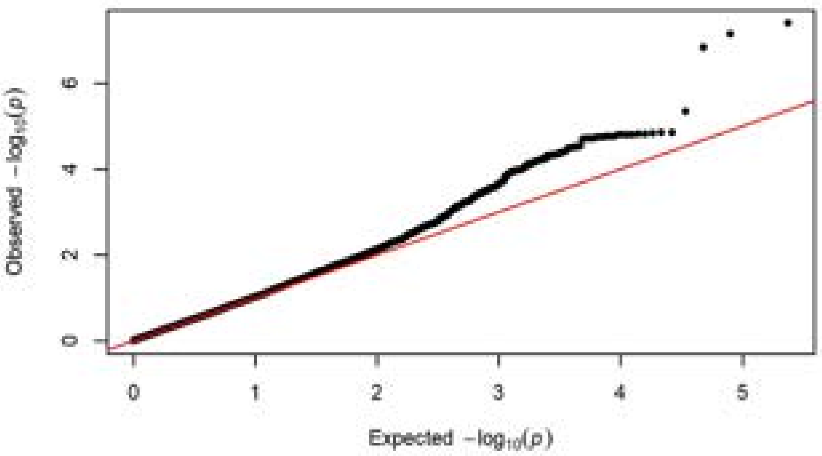

Q-Q Plot Magazine Entries NCS Day 5 - Charles River C72 (n=358)

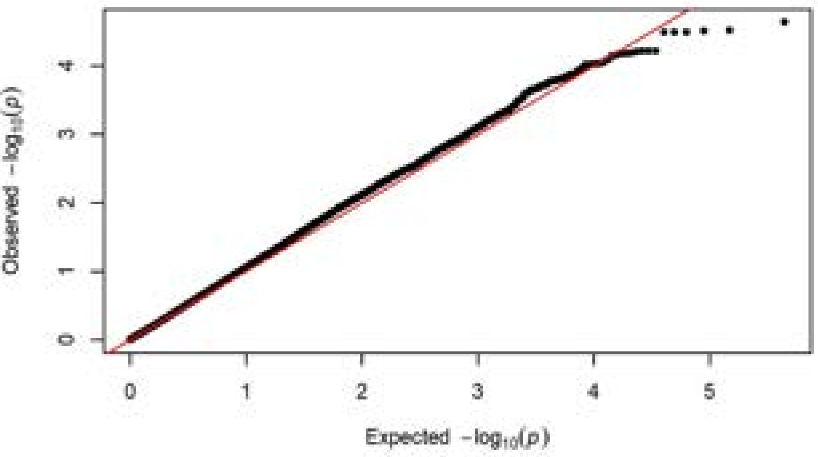

Q-Q Plot Probability Difference Day 1 - Charles River R09-P3/7/10 (n=4[illegible]

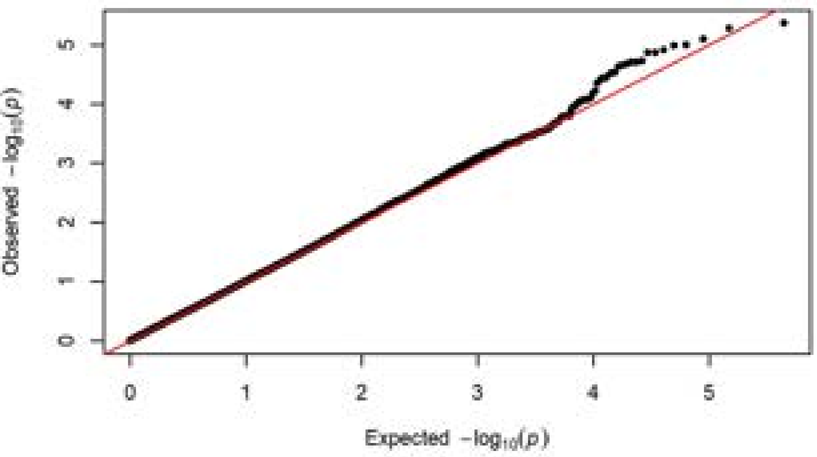

Q-Q Plot Probability Difference Day 1 - Harlan 202A/C-208A (n=1066)

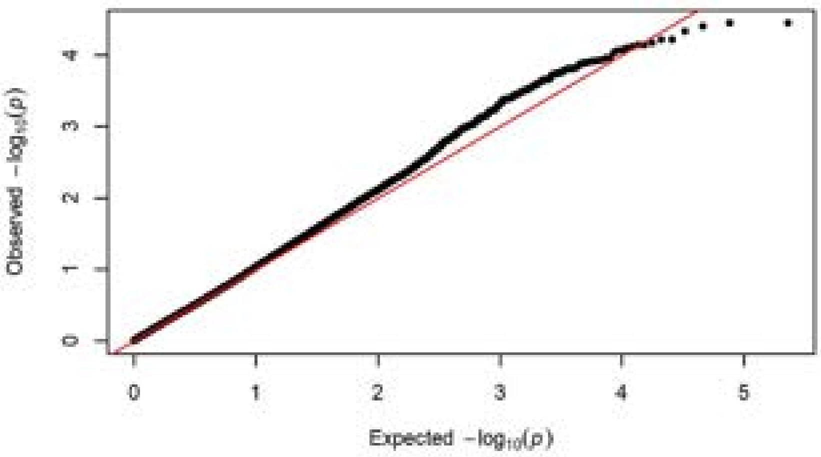

Q-Q Plot Probability Difference Day 1 - Charles River R04 (n=650)

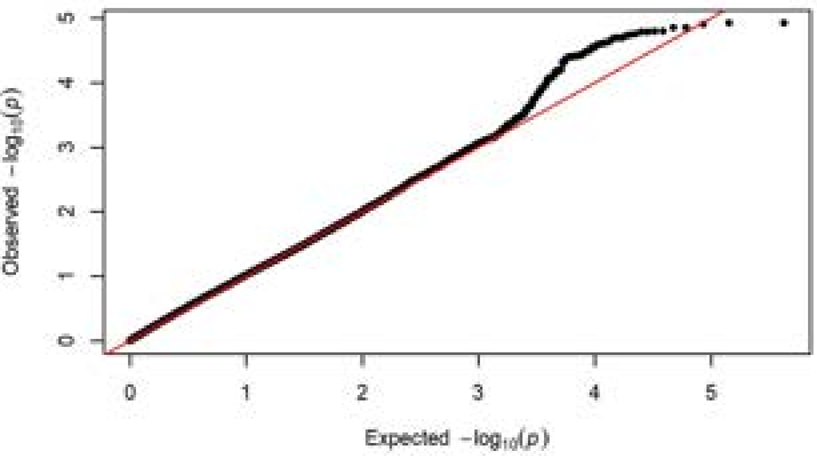

Q-Q Plot Probability Difference Day 1 - Harlan 206 (n=758)

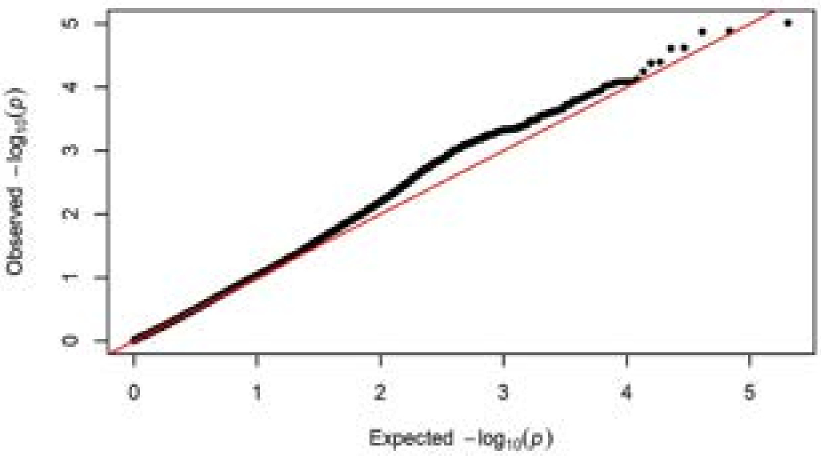

Q-Q Plot Probability Difference Day 1 - Charles River P09 (n=295)

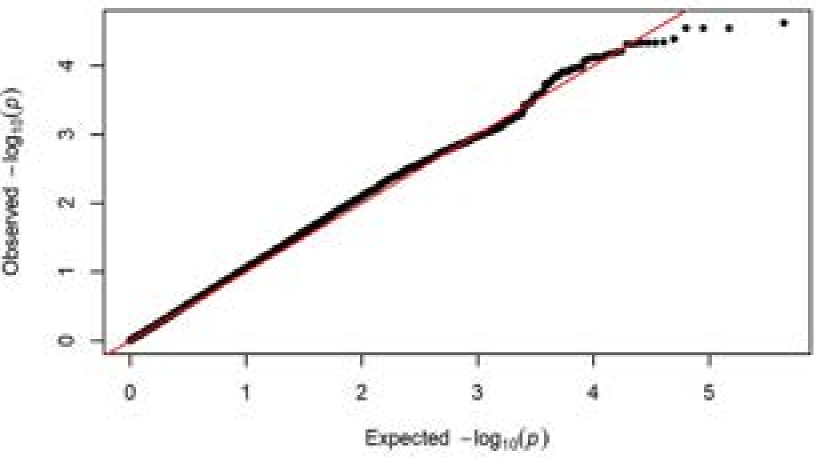

Q-Q Plot Probability Difference Day 1 - Harlan 217 (n=351)

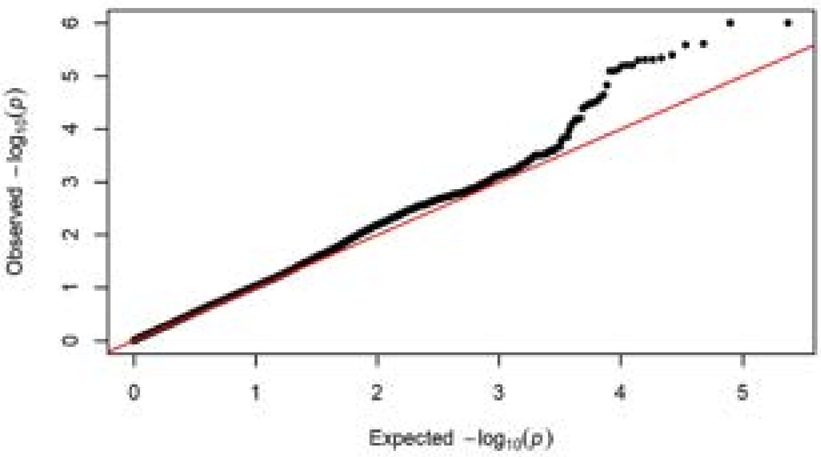

Q-Q Plot Probability Difference Day 1 - Charles River C72 (n=358)

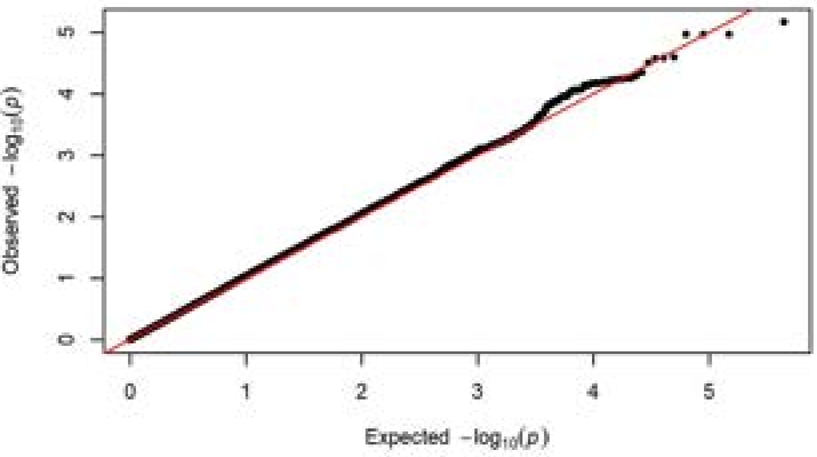

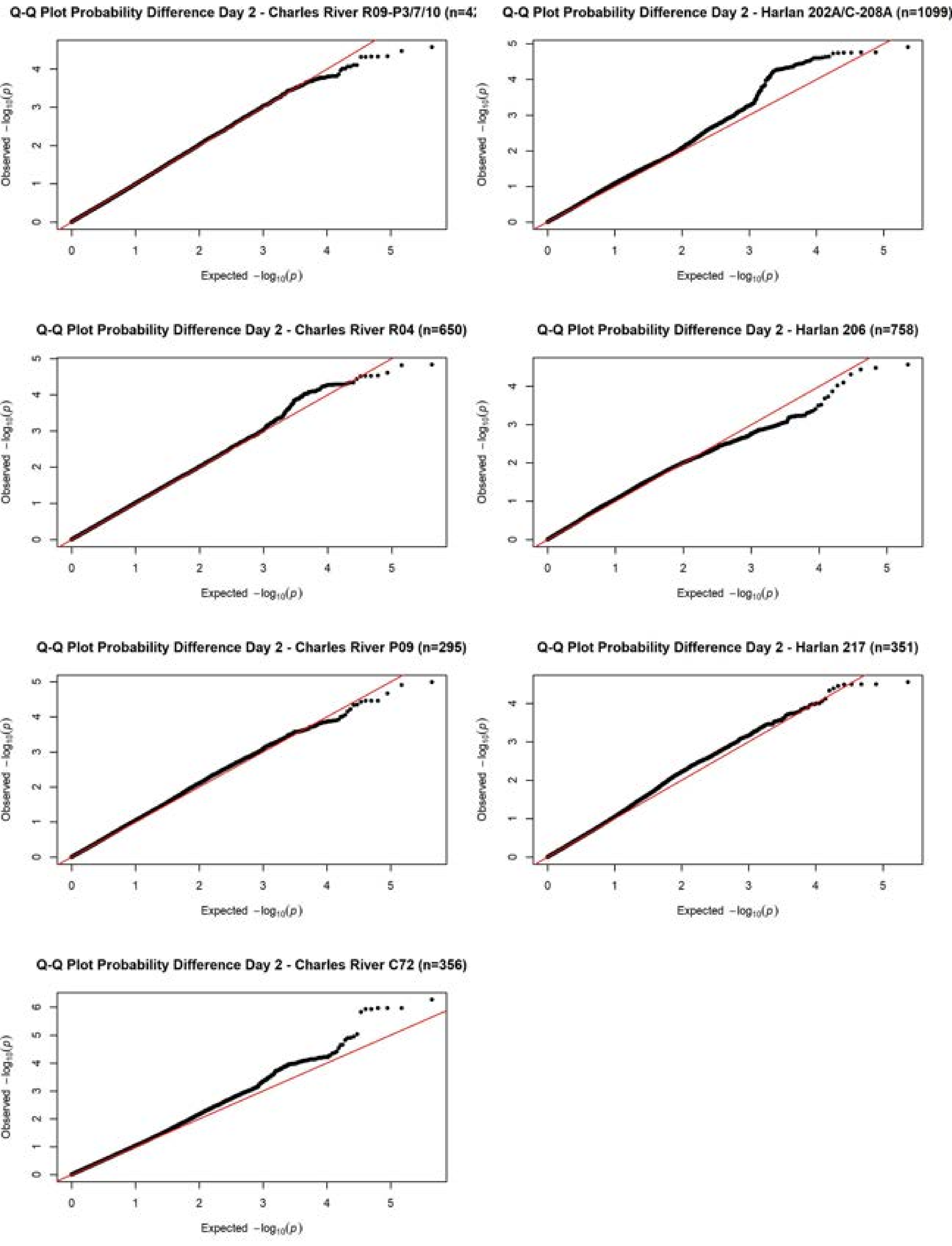
Q-Q Plot Probability Difference Day 2 - Charles River R09-P3/7/10 (n=4
Q-Q Plot Probability Difference Day 2 - Harlan 202A/C-208A (n=1099)
Q-Q Plot Probability Difference Day 2 - Charles River R04 (n=650)
Q-Q Plot Probability Difference Day 2 - Harlan 206 (n=758)
Q-Q Plot Probability Difference Day 2 - Charles River P09 (n=295)
Q-Q Plot Probability Difference Day 2 - Harlan 217 (n=351)
Q-Q Plot Probability Difference Day 2 - Charles River C72 (n=356)
Observed $-\log_{10}(p)$
Expected $-\log_{10}(p)$

Q-Q Plot Probability Difference Day 3 - Charles River R09-P3/7/10 (n=4[illegible]

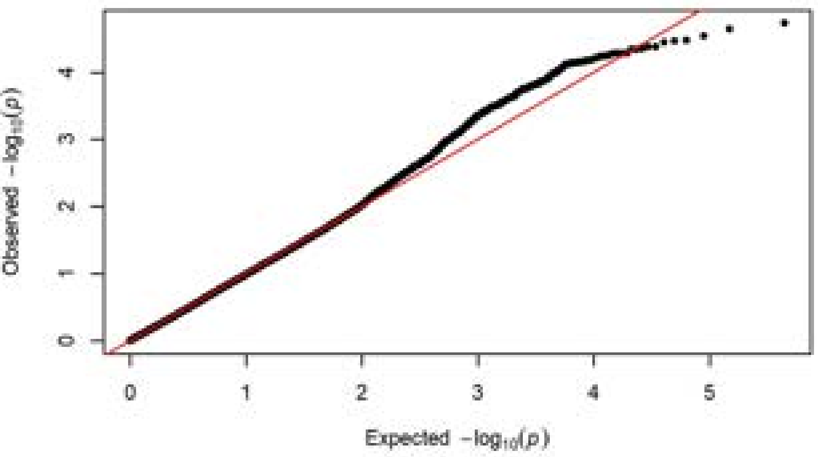

Q-Q Plot Probability Difference Day 3 - Harlan 202A/C-208A (n=1096)

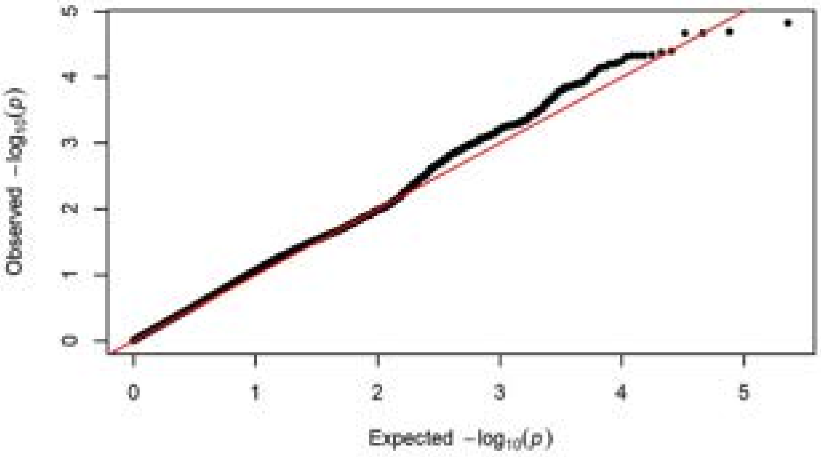

Q-Q Plot Probability Difference Day 3 - Charles River R04 (n=650)

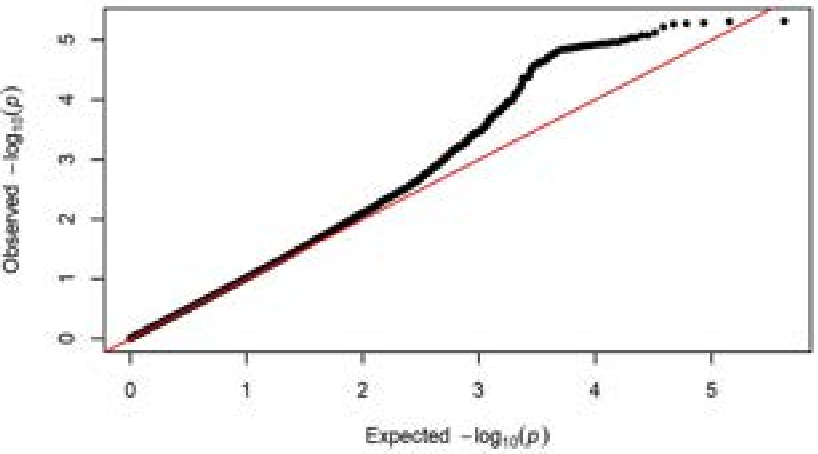

Q-Q Plot Probability Difference Day 3 - Harlan 206 (n=758)

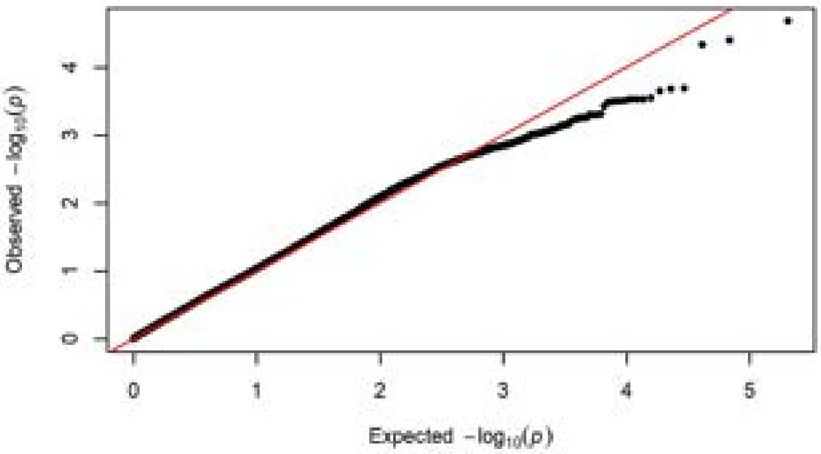

Q-Q Plot Probability Difference Day 3 - Charles River P09 (n=295)

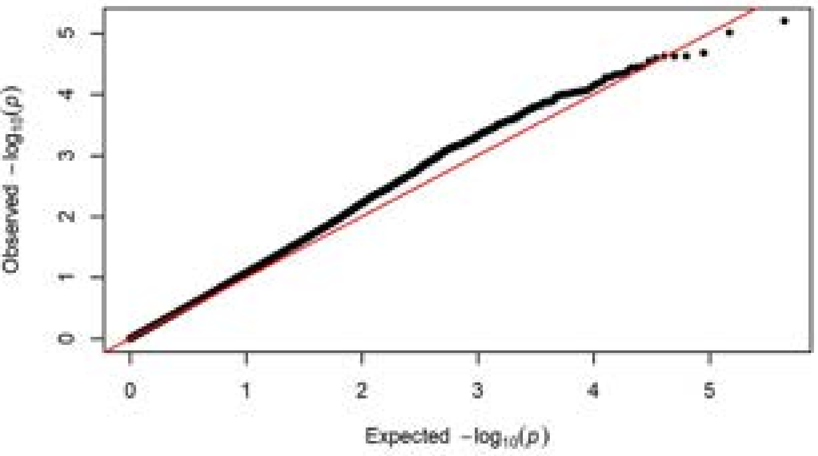

Q-Q Plot Probability Difference Day 3 - Harlan 217 (n=351)

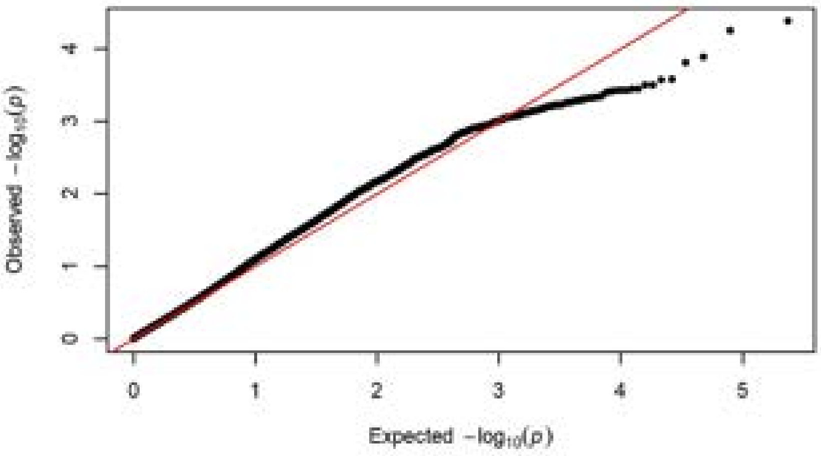

Q-Q Plot Probability Difference Day 3 - Charles River C72 (n=358)

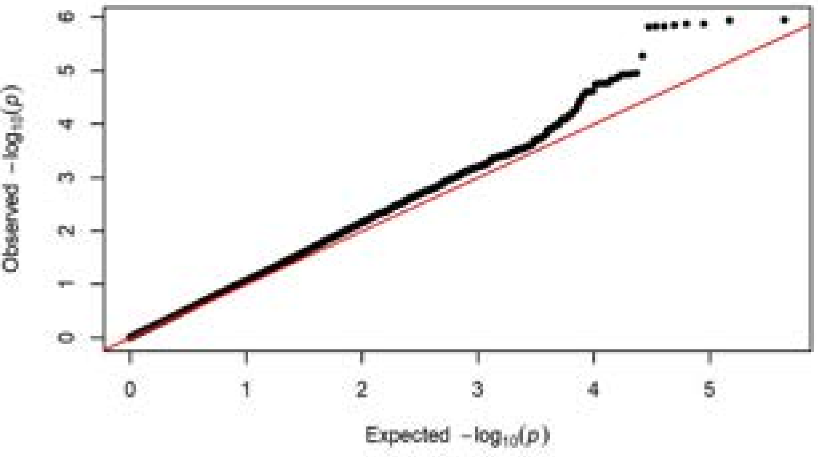

Q-Q Plot Probability Difference Day 4 - Charles River R09-P3/7/10 (n=4[illegible]

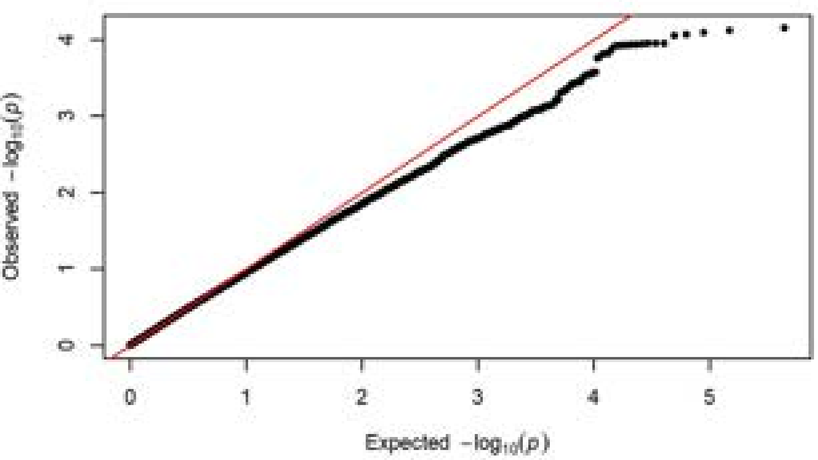

Q-Q Plot Probability Difference Day 4 - Harlan 202A/C-208A (n=1099)

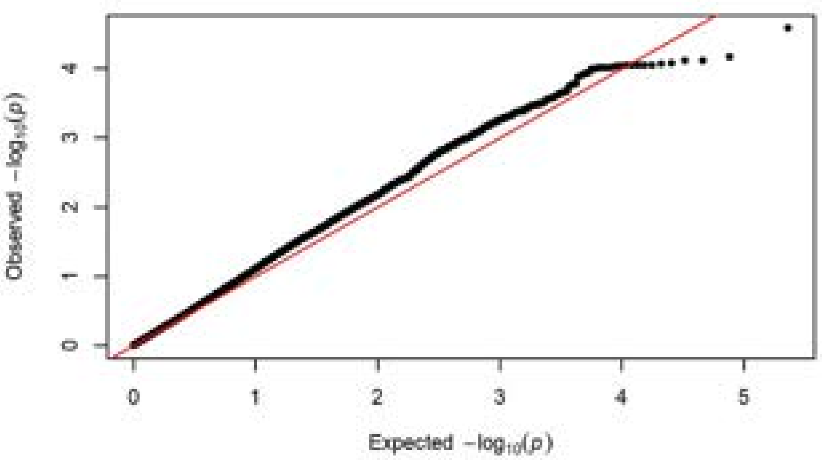

Q-Q Plot Probability Difference Day 4 - Charles River R04 (n=650)

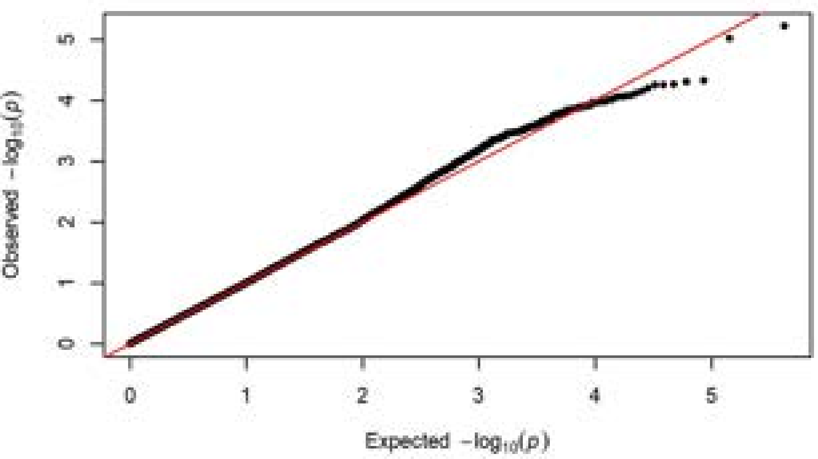

Q-Q Plot Probability Difference Day 4 - Harlan 206 (n=758)

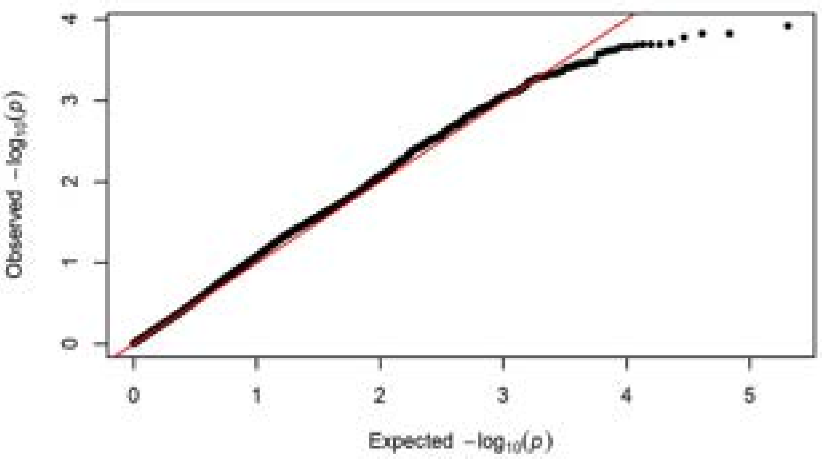

Q-Q Plot Probability Difference Day 4 - Charles River P09 (n=295)

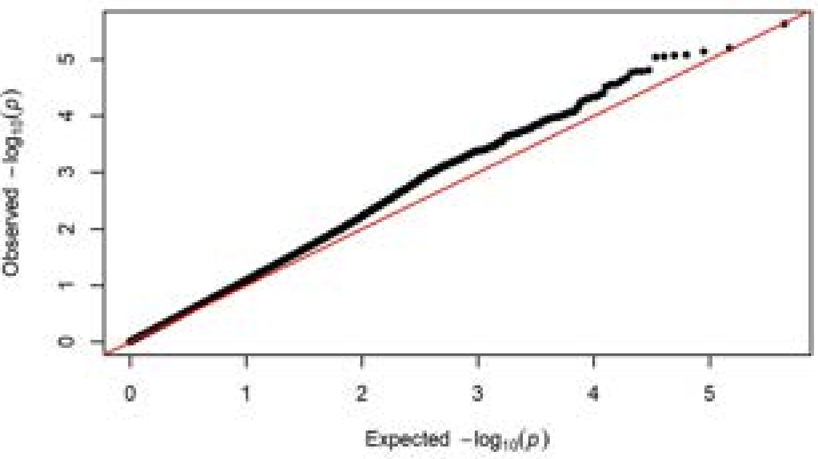

Q-Q Plot Probability Difference Day 4 - Harlan 217 (n=351)

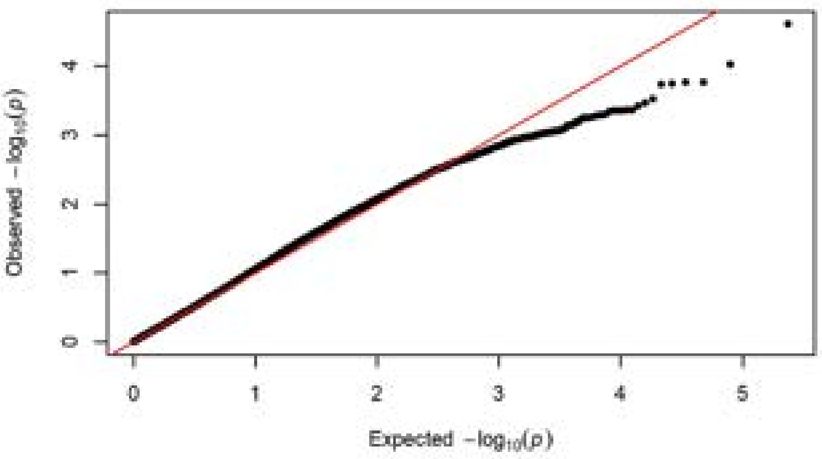

Q-Q Plot Probability Difference Day 4 - Charles River C72 (n=358)

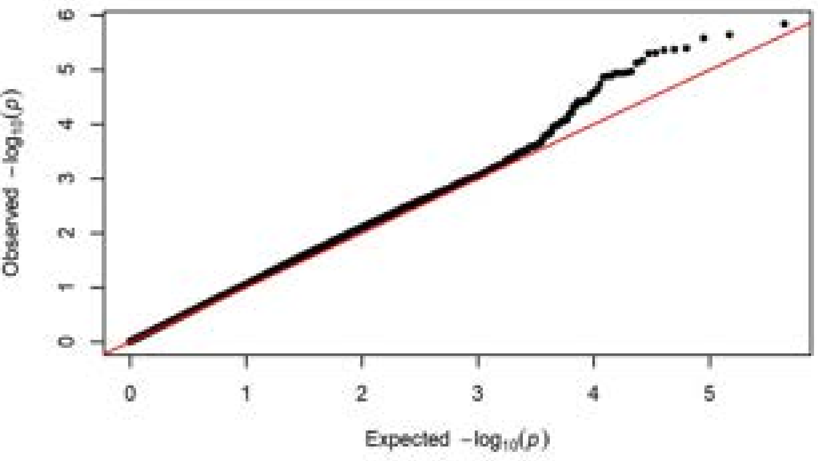

Q-Q Plot Probability Difference Day 5 - Charles River R09-P3/7/10 (n=4

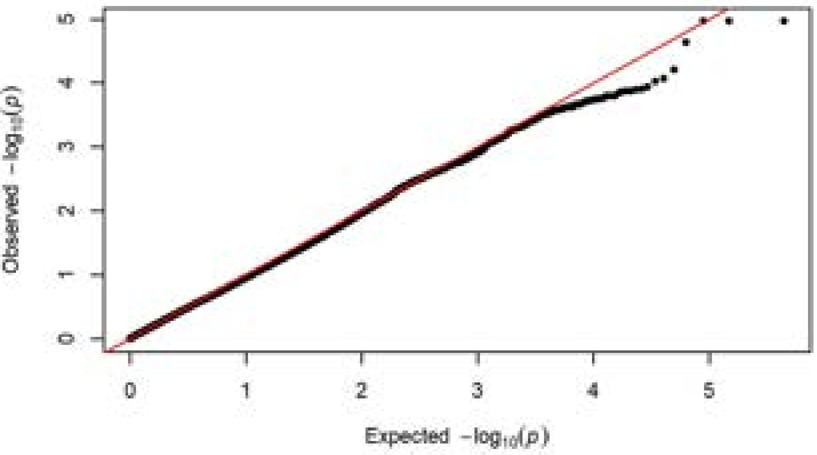

Q-Q Plot Probability Difference Day 5 - Harlan 202A/C-208A (n=1099)

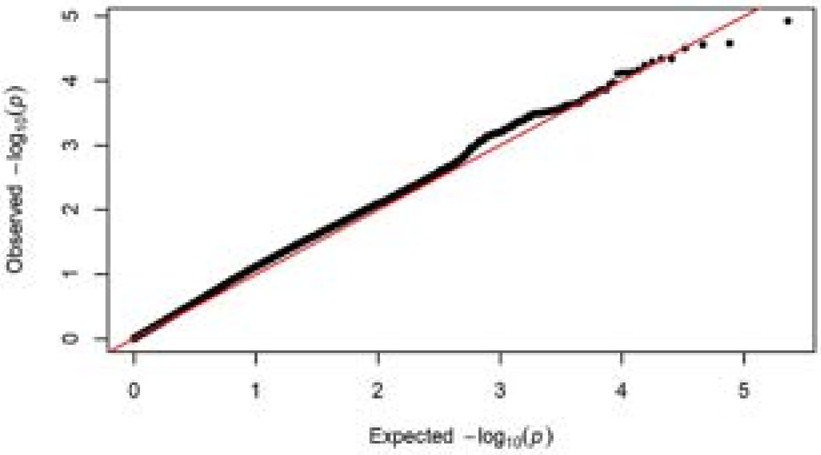

Q-Q Plot Probability Difference Day 5 - Charles River R04 (n=650)

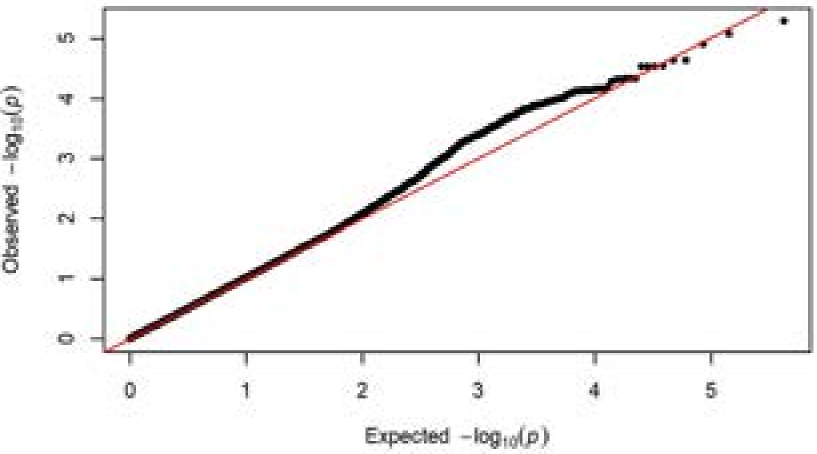

Q-Q Plot Probability Difference Day 5 - Harlan 206 (n=758)

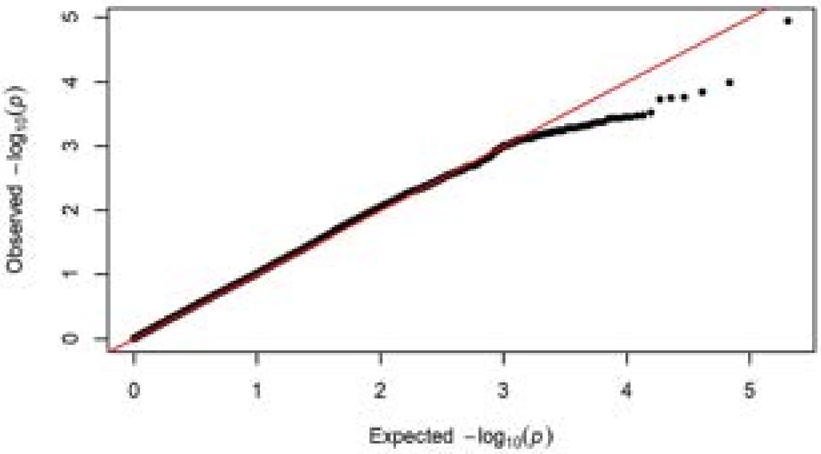

Q-Q Plot Probability Difference Day 5 - Charles River P09 (n=295)

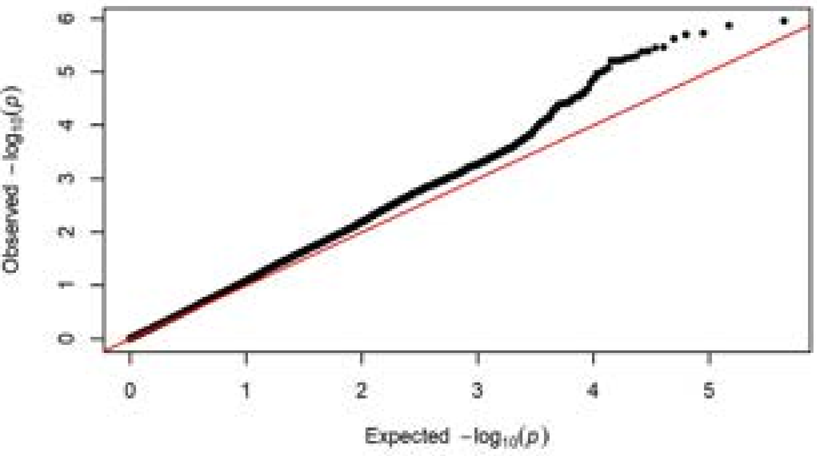

Q-Q Plot Probability Difference Day 5 - Harlan 217 (n=351)

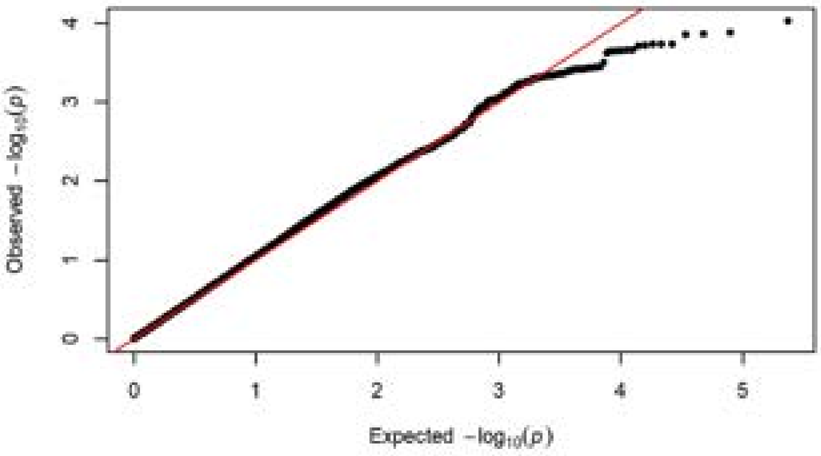

Q-Q Plot Probability Difference Day 5 - Charles River C72 (n=358)

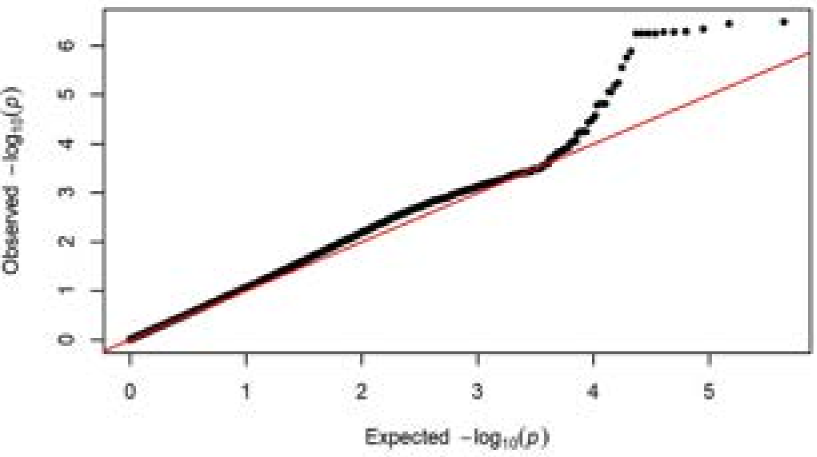

Q-Q Plot Probability of Lever Press Day 1 - Charles River R09-P3/7/10 (n=

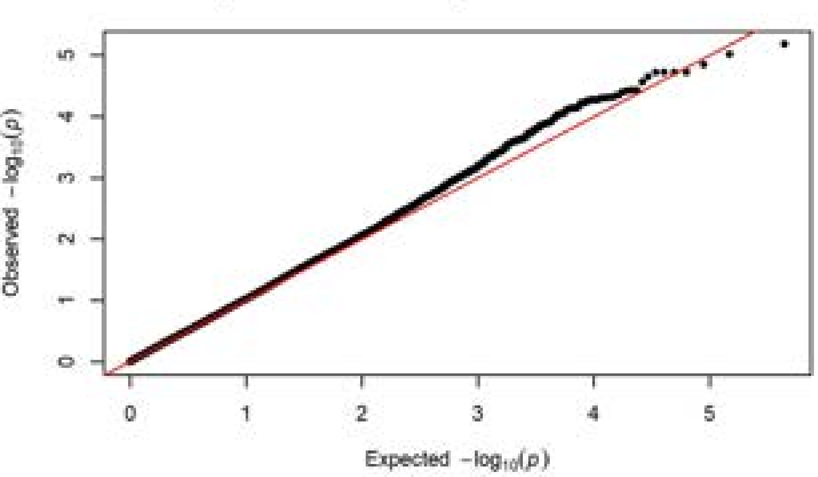

Q-Q Plot Probability of Lever Press Day 1 - Harlan 202A/C-208A (n=106

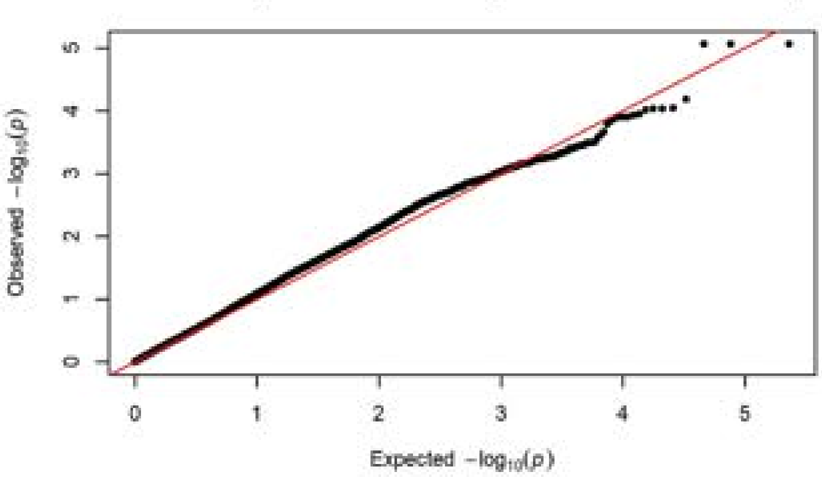

Q-Q Plot Probability of Lever Press Day 1 - Charles River R04 (n=650

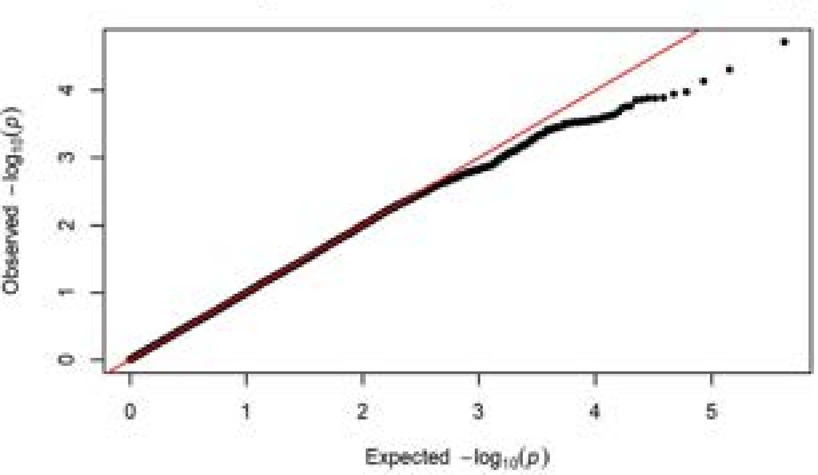

Q-Q Plot Probability of Lever Press Day 1 - Harlan 206 (n=758)

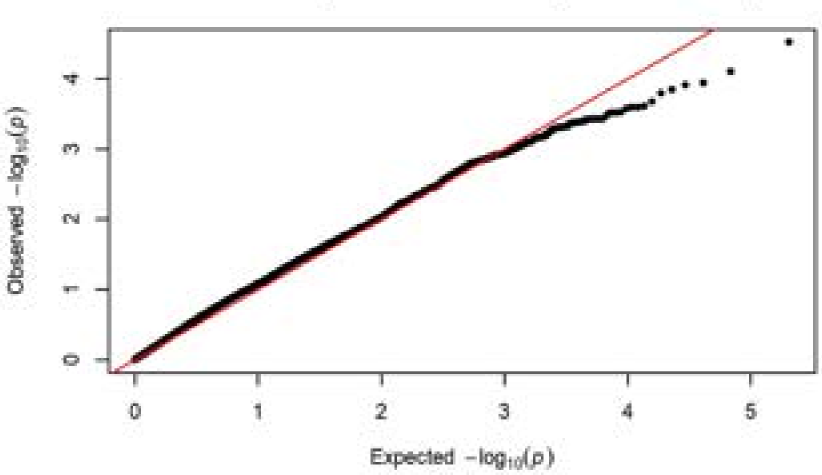

Q-Q Plot Probability of Lever Press Day 1 - Charles River P09 (n=295

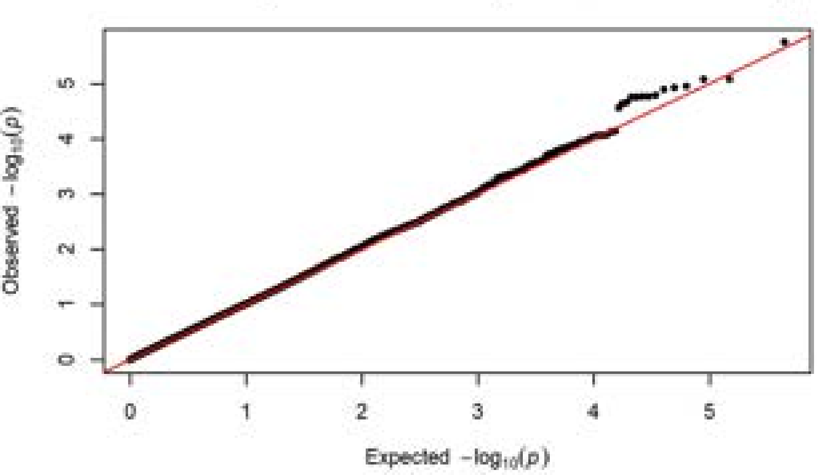

Q-Q Plot Probability of Lever Press Day 1 - Harlan 217 (n=351)

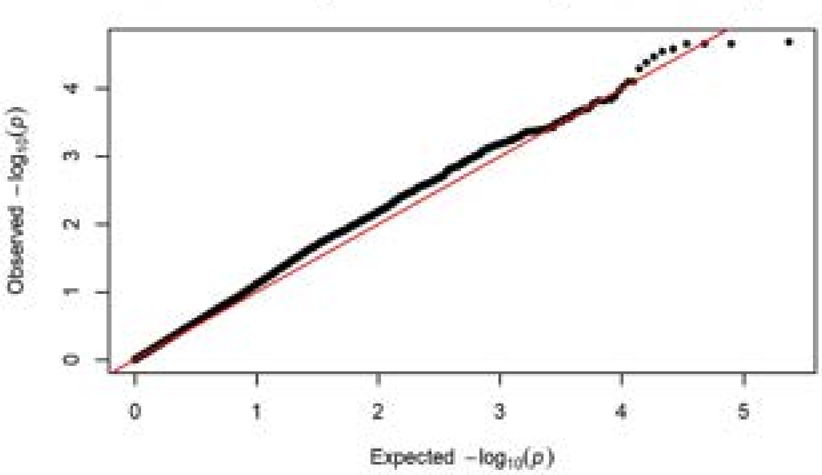

Q-Q Plot Probability of Lever Press Day 1 - Charles River C72 (n=358

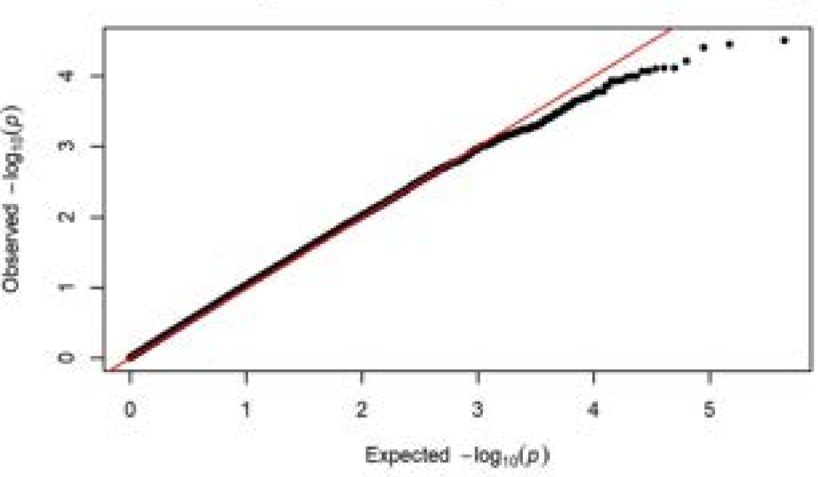

Q-Q Plot Probability of Lever Press Day 2 - Charles River R09-P3/7/10 (n=

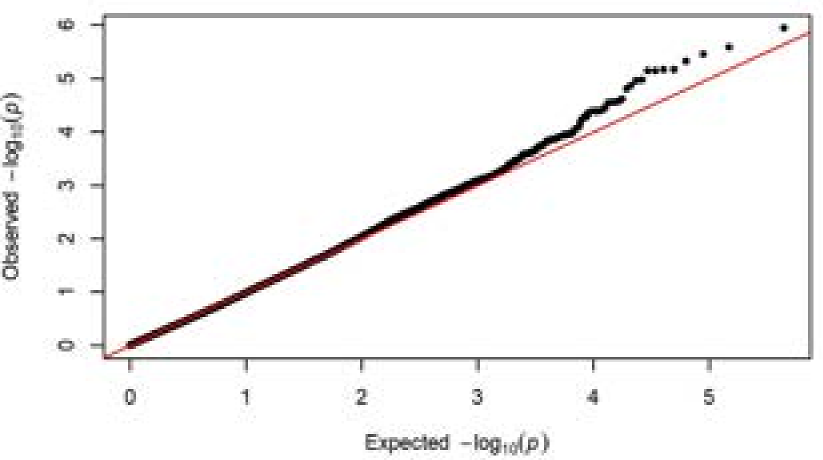

Q-Q Plot Probability of Lever Press Day 2 - Harlan 202A/C-208A (n=109

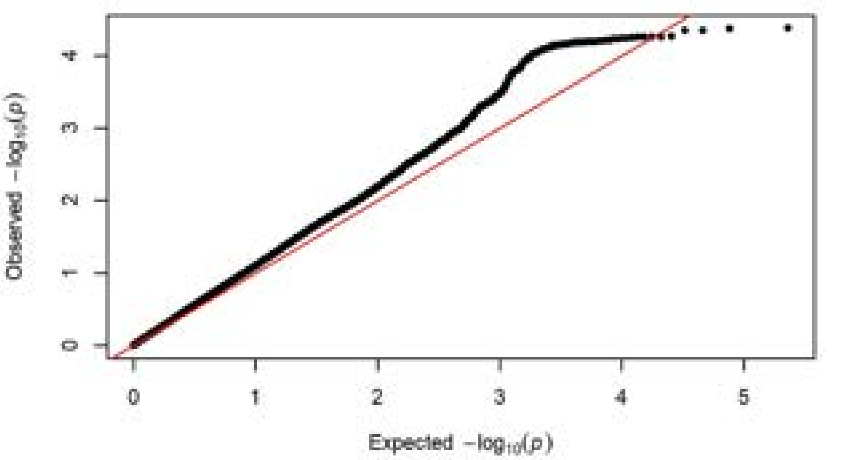

Q-Q Plot Probability of Lever Press Day 2 - Charles River R04 (n=650

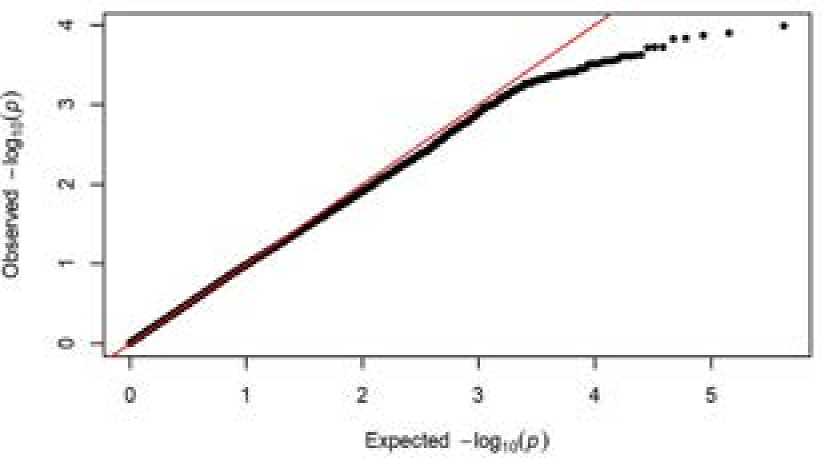

Q-Q Plot Probability of Lever Press Day 2 - Harlan 206 (n=758)

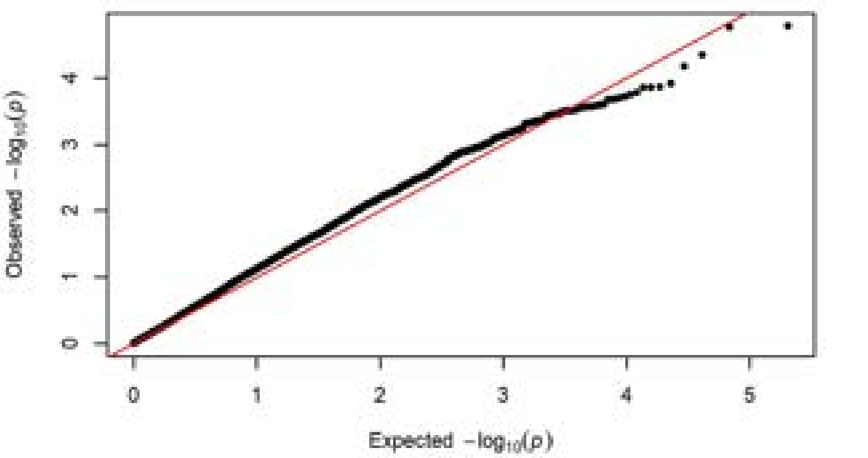

Q-Q Plot Probability of Lever Press Day 2 - Charles River P09 (n=295

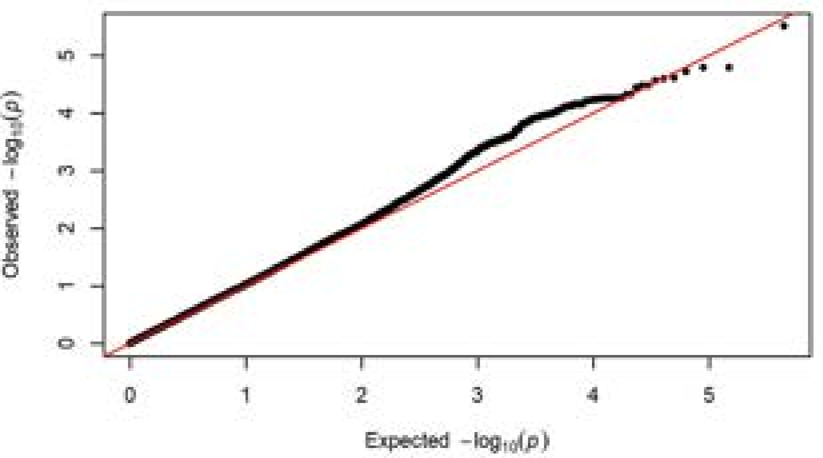

Q-Q Plot Probability of Lever Press Day 2 - Harlan 217 (n=351)

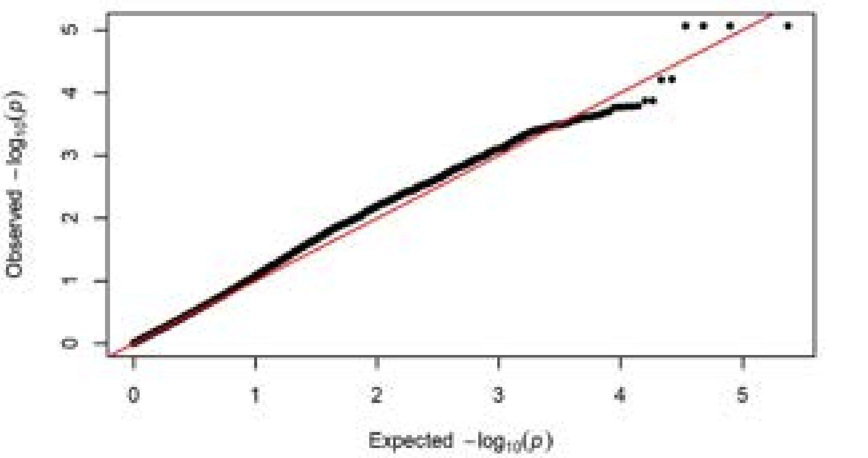

Q-Q Plot Probability of Lever Press Day 2 - Charles River C72 (n=356

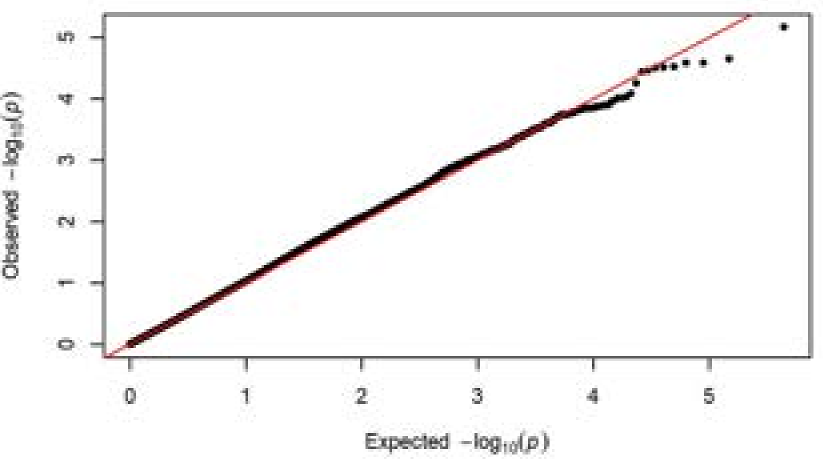

Q-Q Plot Probability of Lever Press Day 3 - Charles River R09-P3/7/10 (n=

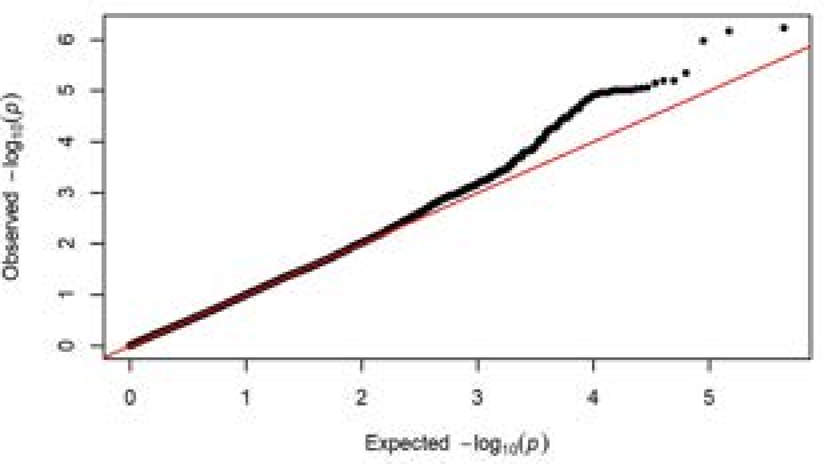

Q-Q Plot Probability of Lever Press Day 3 - Harlan 202A/C-208A (n=109

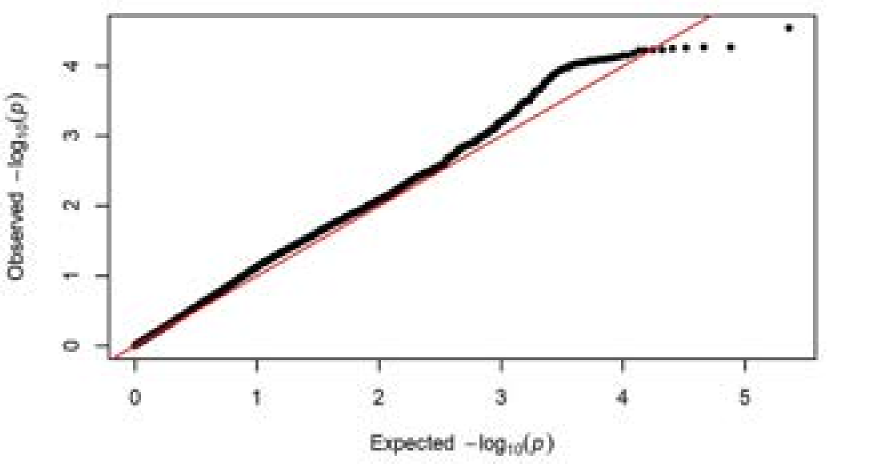

Q-Q Plot Probability of Lever Press Day 3 - Charles River R04 (n=650

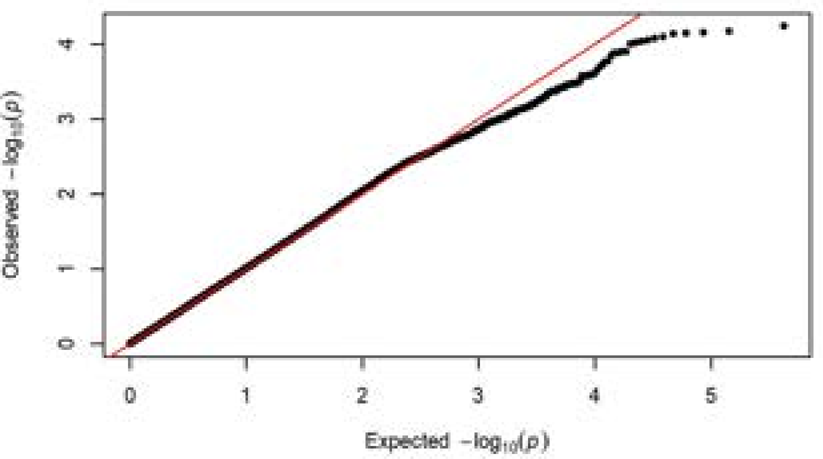

Q-Q Plot Probability of Lever Press Day 3 - Harlan 206 (n=758)

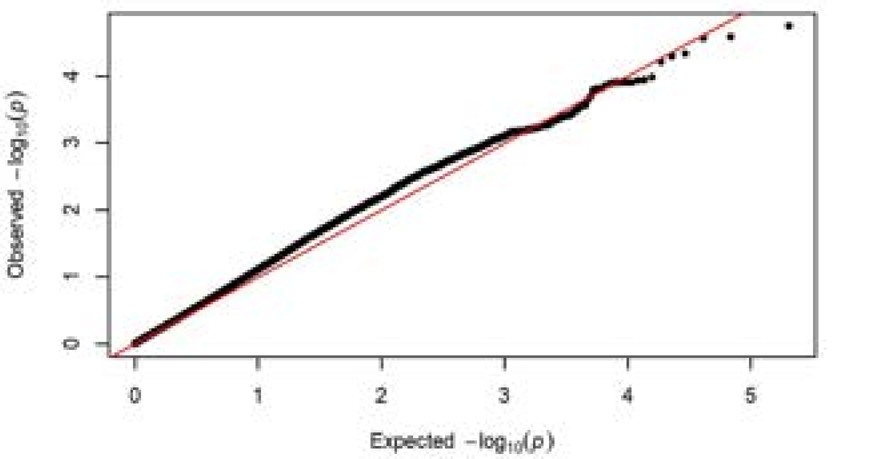

Q-Q Plot Probability of Lever Press Day 3 - Charles River P09 (n=295

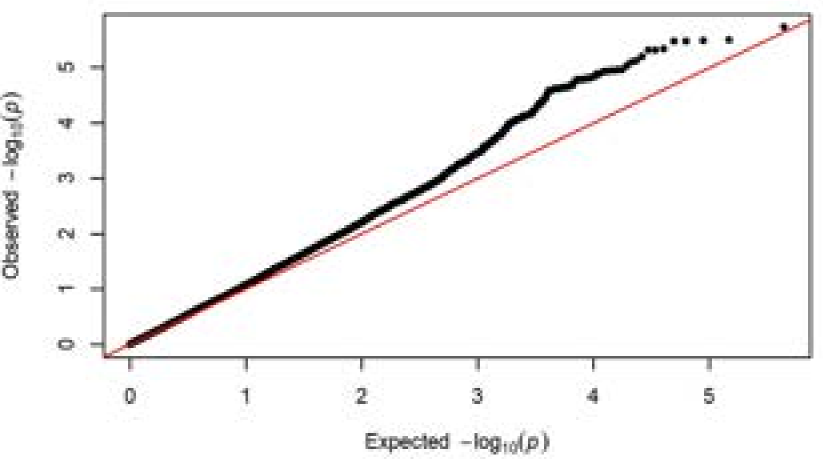

Q-Q Plot Probability of Lever Press Day 3 - Harlan 217 (n=351)

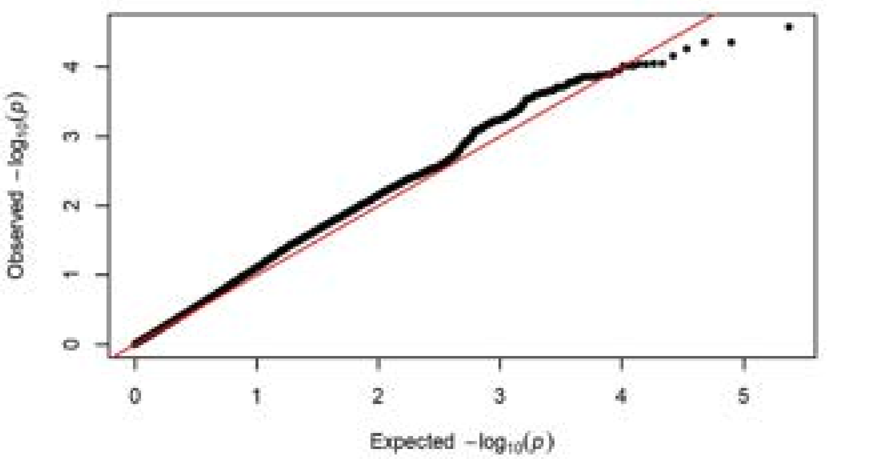

Q-Q Plot Probability of Lever Press Day 3 - Charles River C72 (n=358

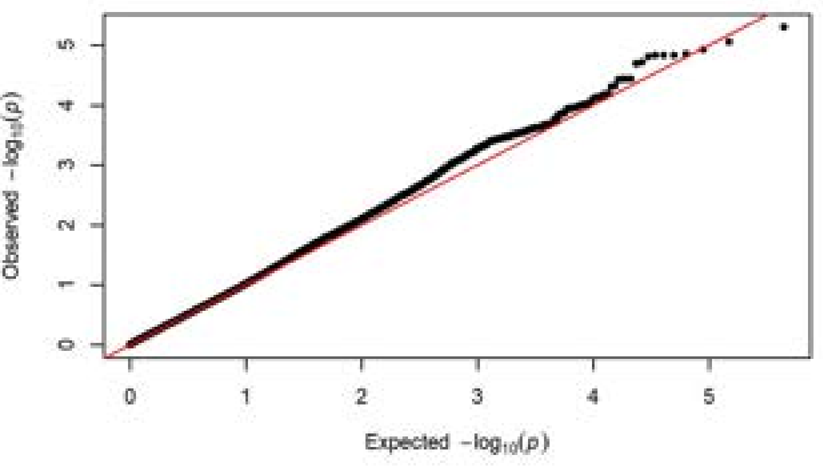

Q-Q Plot Probability of Lever Press Day 4 - Charles River R09-P3/7/10 (n=

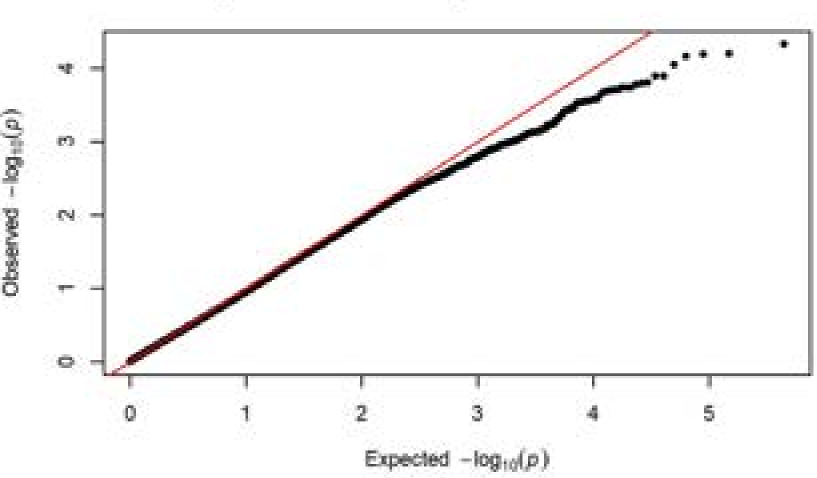

Q-Q Plot Probability of Lever Press Day 4 - Harlan 202A/C-208A (n=109

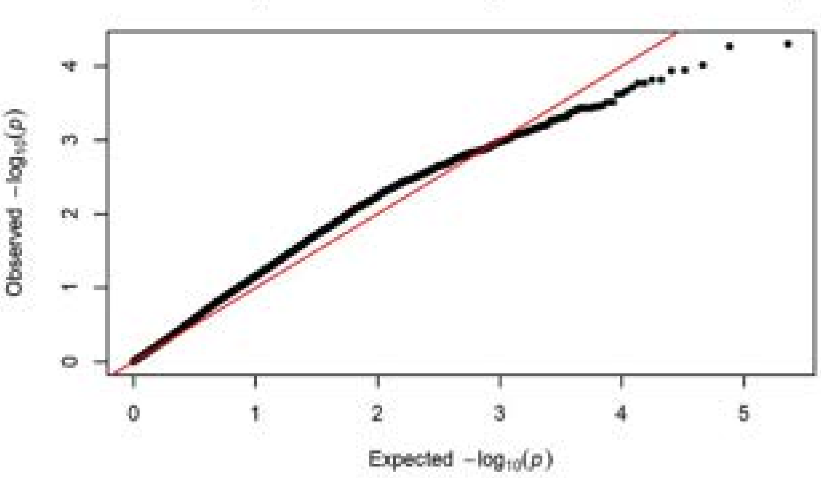

Q-Q Plot Probability of Lever Press Day 4 - Charles River R04 (n=650

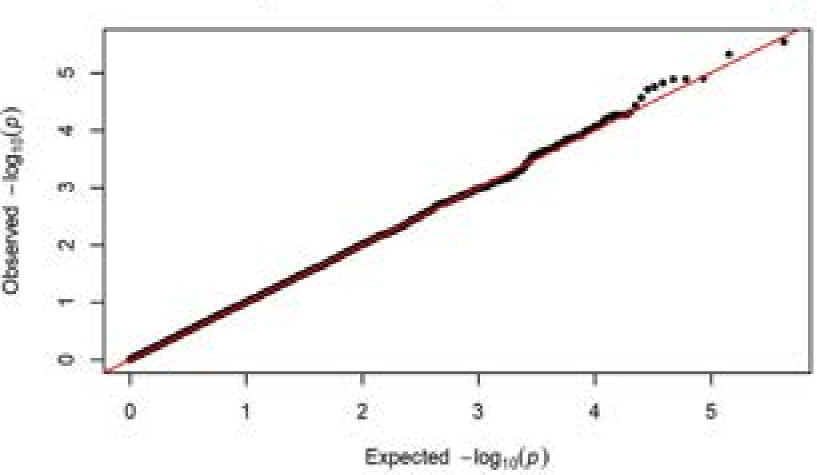

Q-Q Plot Probability of Lever Press Day 4 - Harlan 206 (n=758)

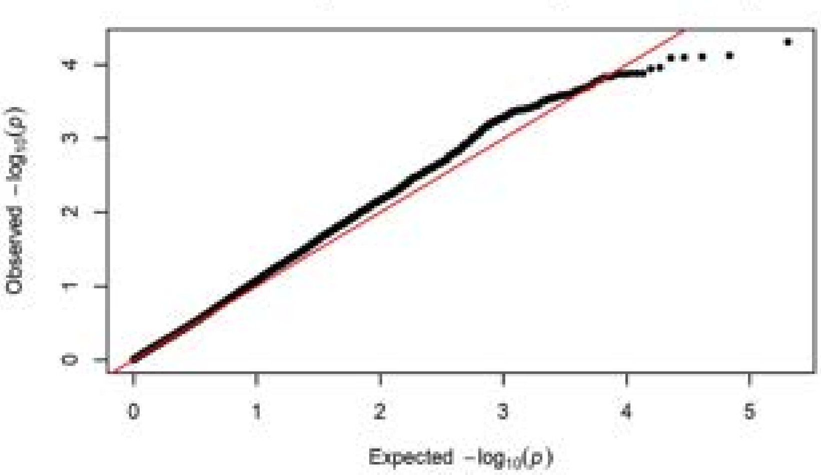

Q-Q Plot Probability of Lever Press Day 4 - Charles River P09 (n=295

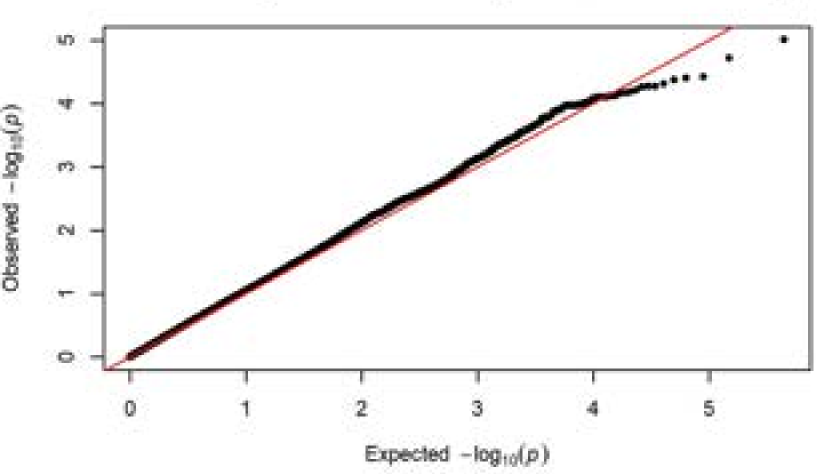

Q-Q Plot Probability of Lever Press Day 4 - Harlan 217 (n=351)

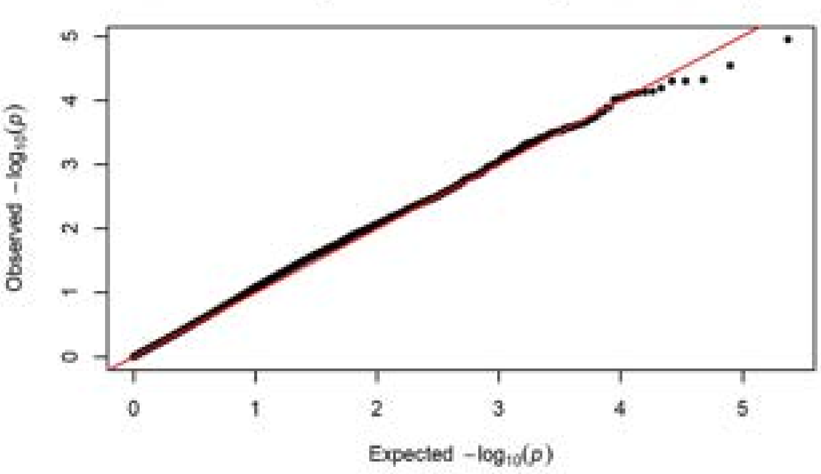

Q-Q Plot Probability of Lever Press Day 4 - Charles River C72 (n=358

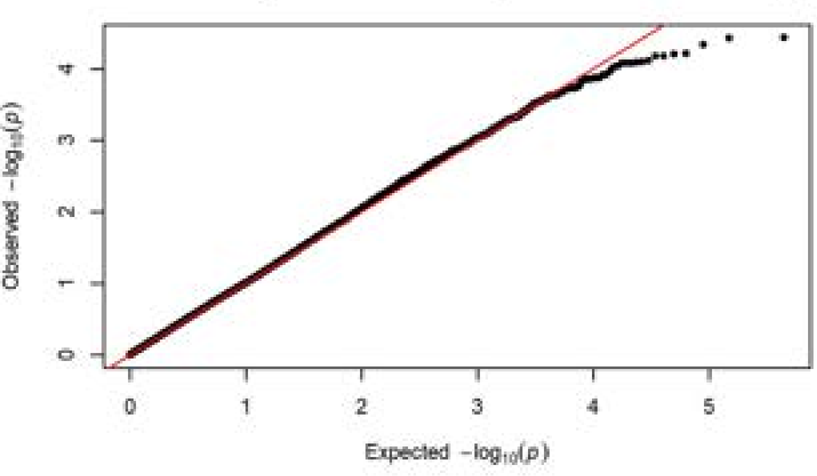

Q-Q Plot Probability of Lever Press Day 5 - Charles River R09-P3/7/10 (n=

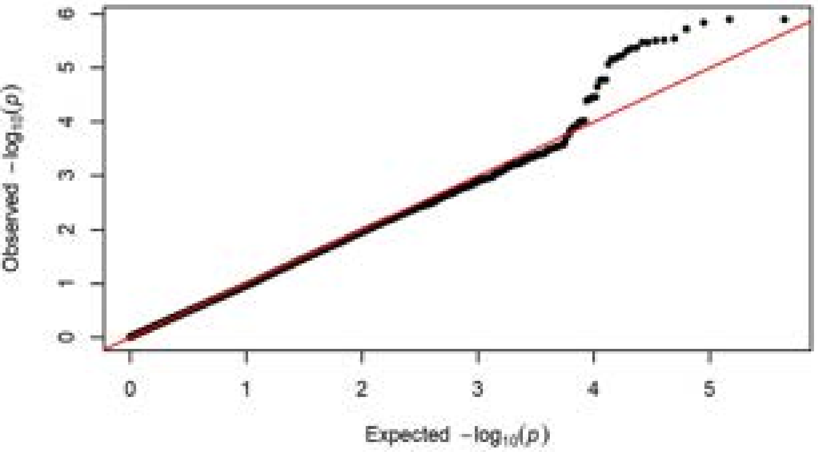

Q-Q Plot Probability of Lever Press Day 5 - Harlan 202A/C-208A (n=10

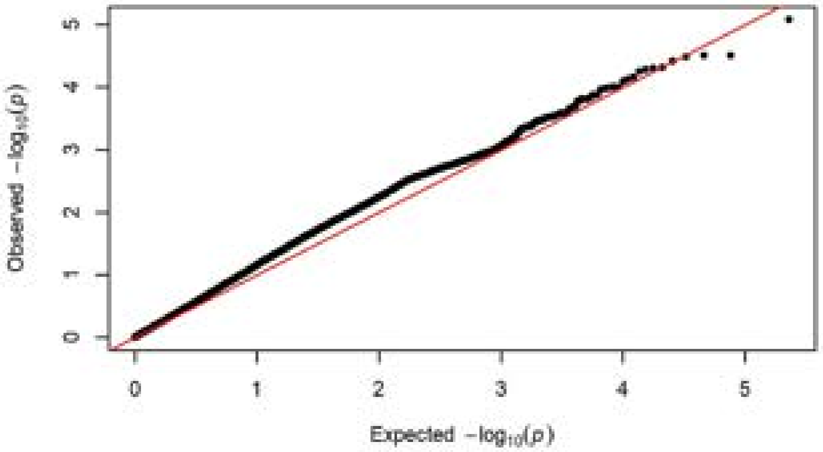

Q-Q Plot Probability of Lever Press Day 5 - Charles River R04 (n=650

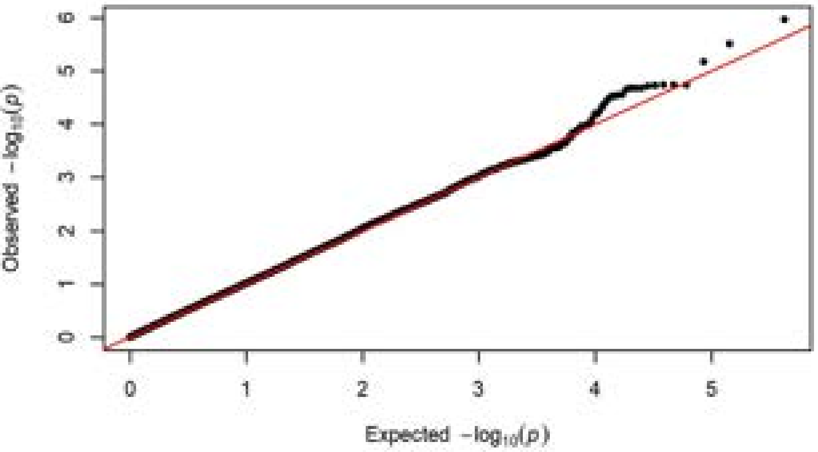

Q-Q Plot Probability of Lever Press Day 5 - Harlan 206 (n=758)

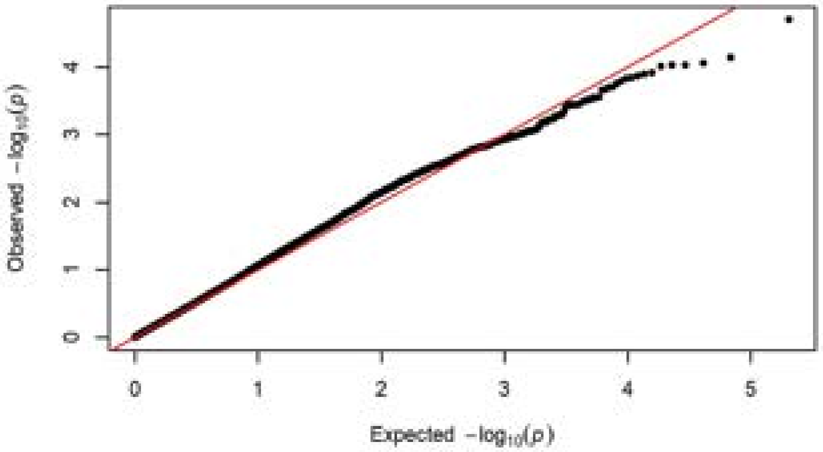

Q-Q Plot Probability of Lever Press Day 5 - Charles River P09 (n=295

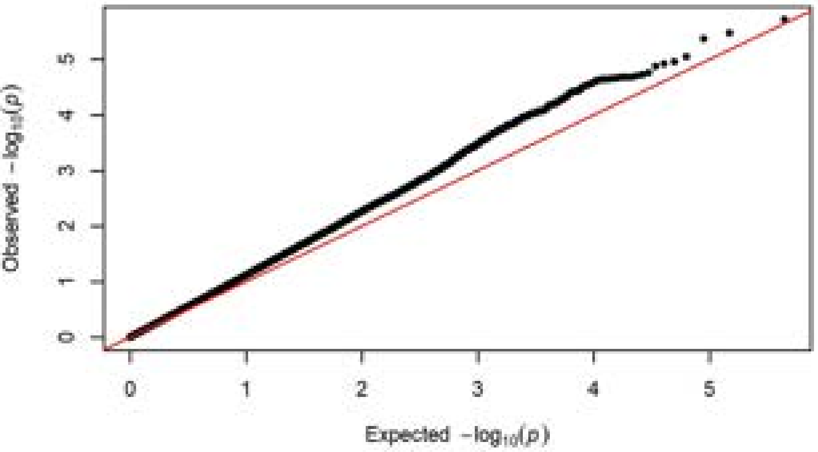

Q-Q Plot Probability of Lever Press Day 5 - Harlan 217 (n=351)

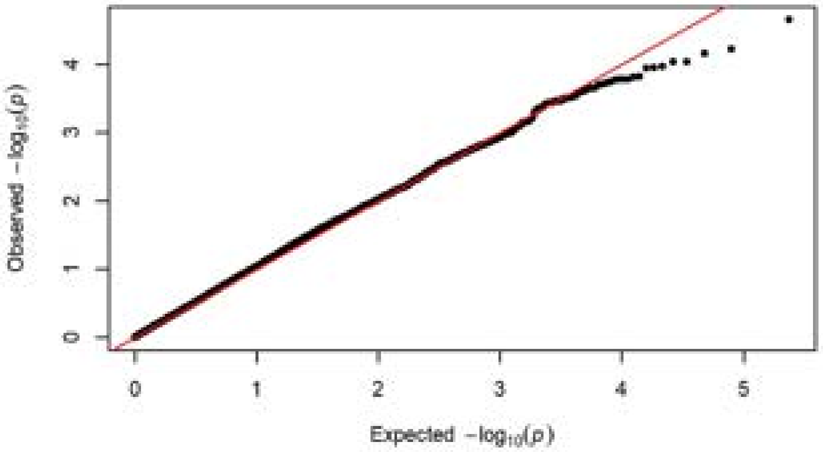

Q-Q Plot Probability of Lever Press Day 5 - Charles River C72 (n=358

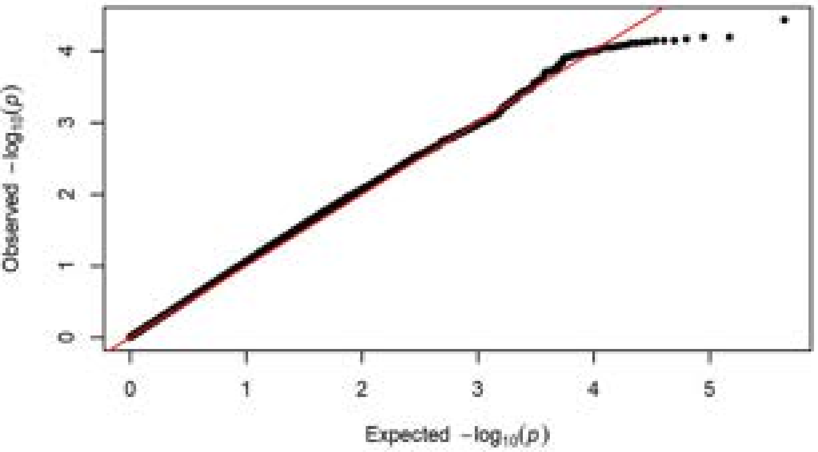

Q-Q Plot Probability of Magazine Entry Day 1 - Charles River R09-P3/7/10 (

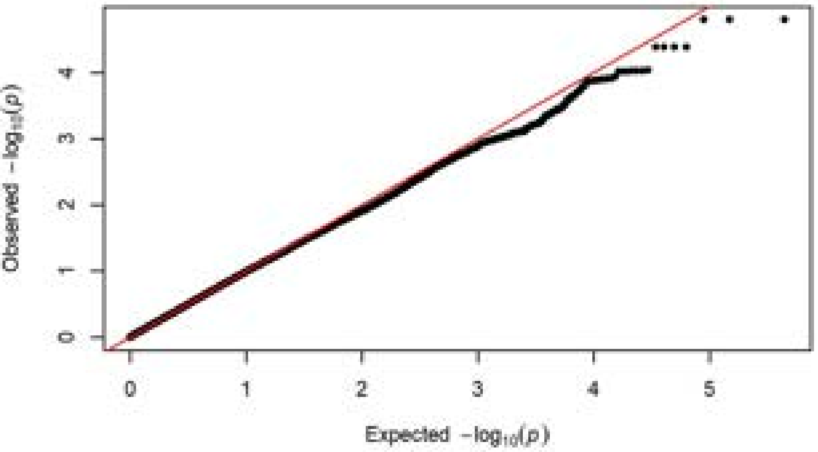

Q-Q Plot Probability of Magazine Entry Day 1 - Harlan 202A/C-208A (n=1

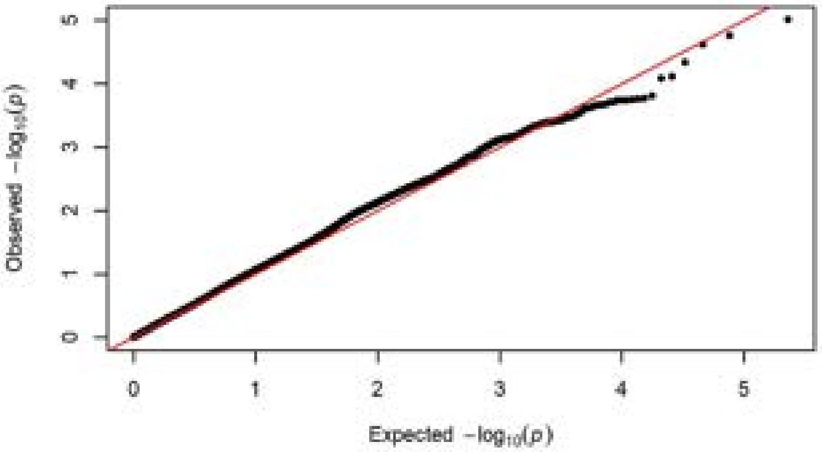

Q-Q Plot Probability of Magazine Entry Day 1 - Charles River R04 (n=6

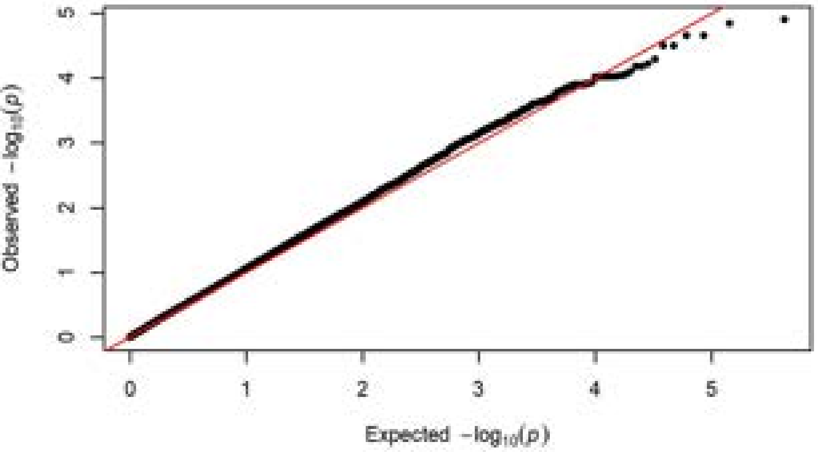

Q-Q Plot Probability of Magazine Entry Day 1 - Harlan 206 (n=758)

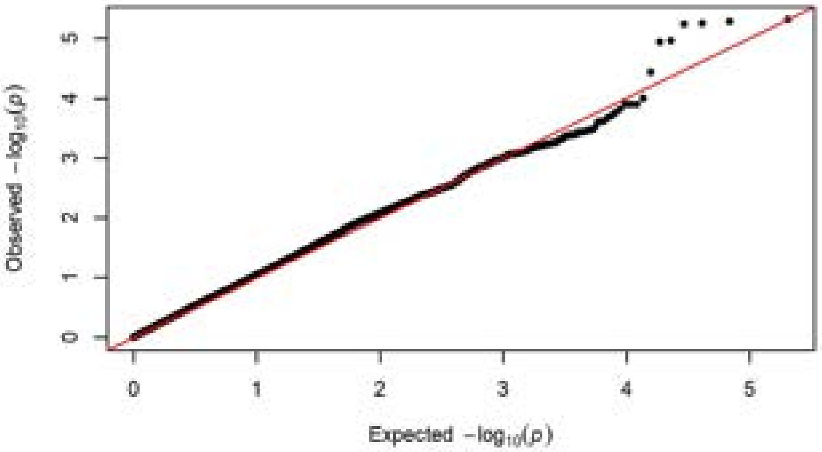

Q-Q Plot Probability of Magazine Entry Day 1 - Charles River P09 (n=2

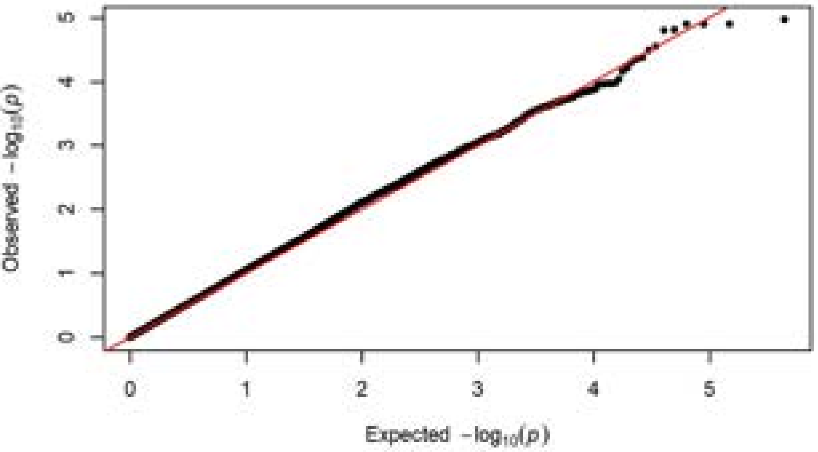

Q-Q Plot Probability of Magazine Entry Day 1 - Harlan 217 (n=351)

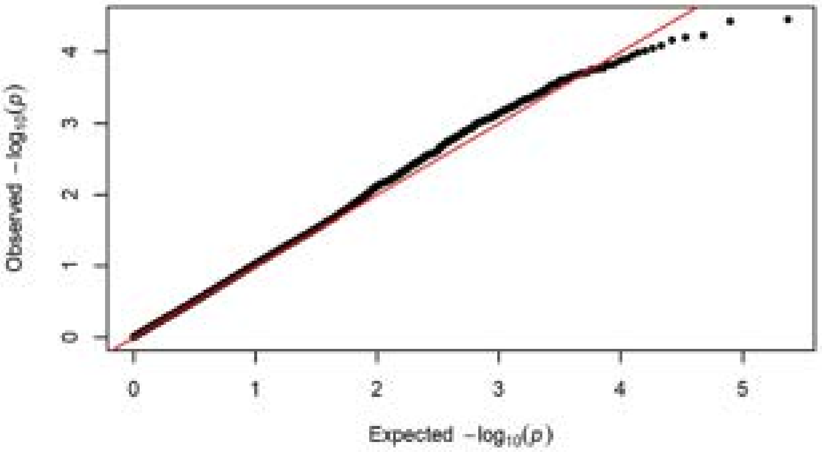

Q-Q Plot Probability of Magazine Entry Day 1 - Charles River C72 (n=3

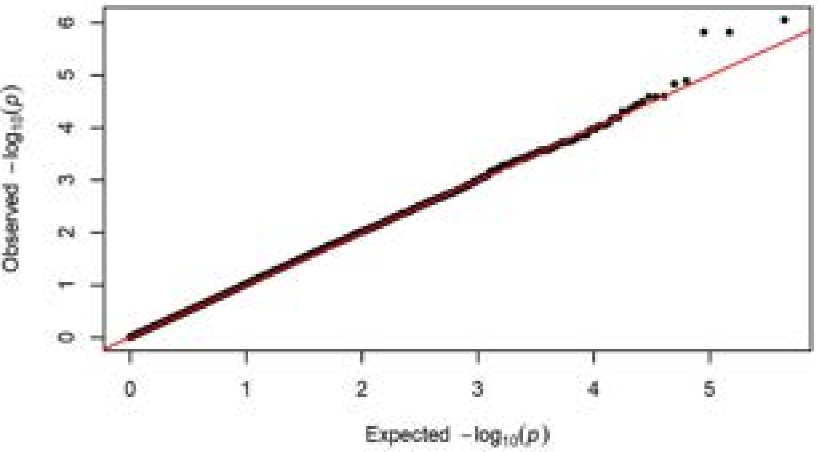

Q Plot Probability of Magazine Entry Day 2 - Charles River R09-P3/7/10 (

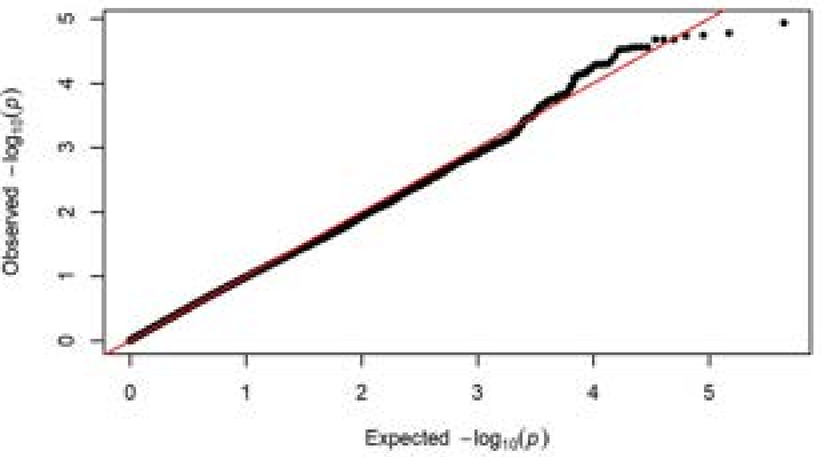

Q-Q Plot Probability of Magazine Entry Day 2 - Harlan 202A/C-208A (n=1

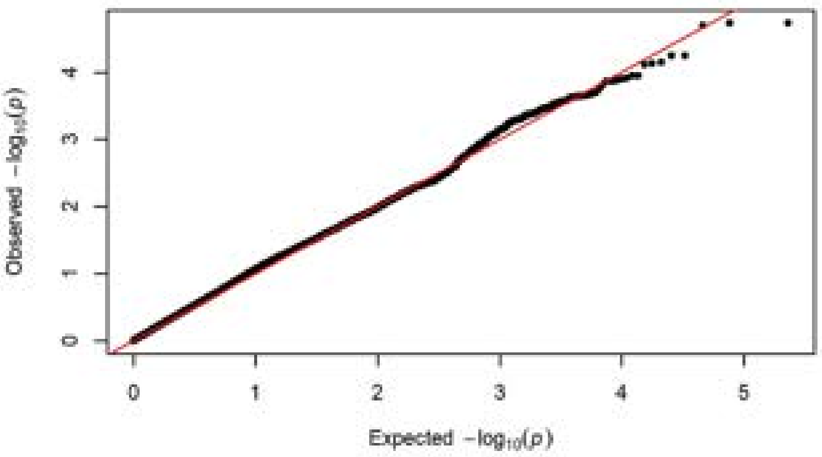

Q-Q Plot Probability of Magazine Entry Day 2 - Charles River R04 (n=6

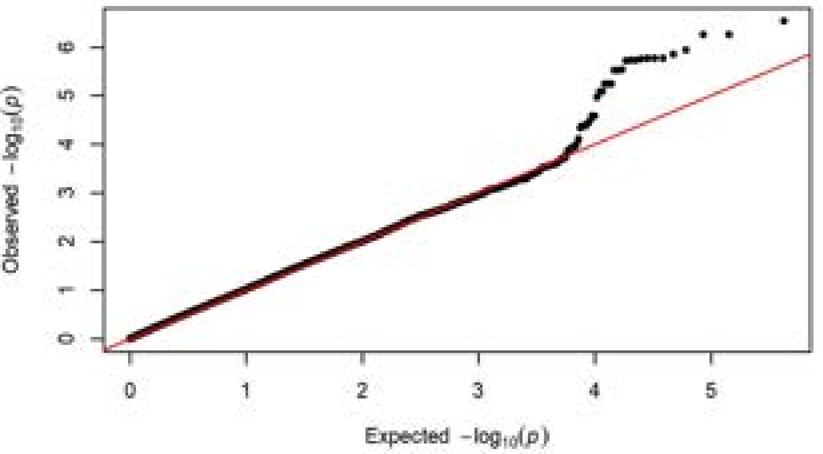

Q-Q Plot Probability of Magazine Entry Day 2 - Harlan 206 (n=758)

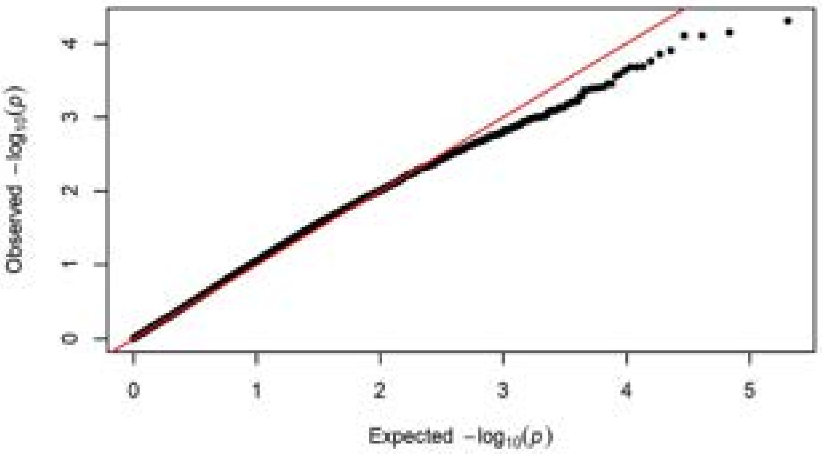

Q-Q Plot Probability of Magazine Entry Day 2 - Charles River P09 (n=2

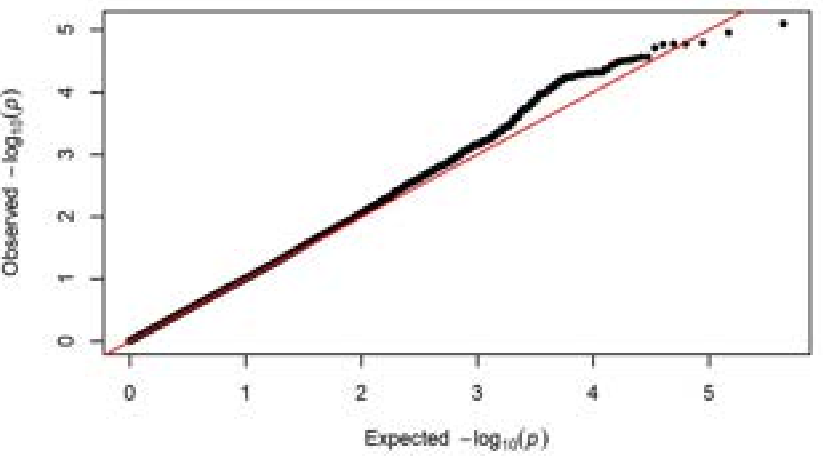

Q-Q Plot Probability of Magazine Entry Day 2 - Harlan 217 (n=351)

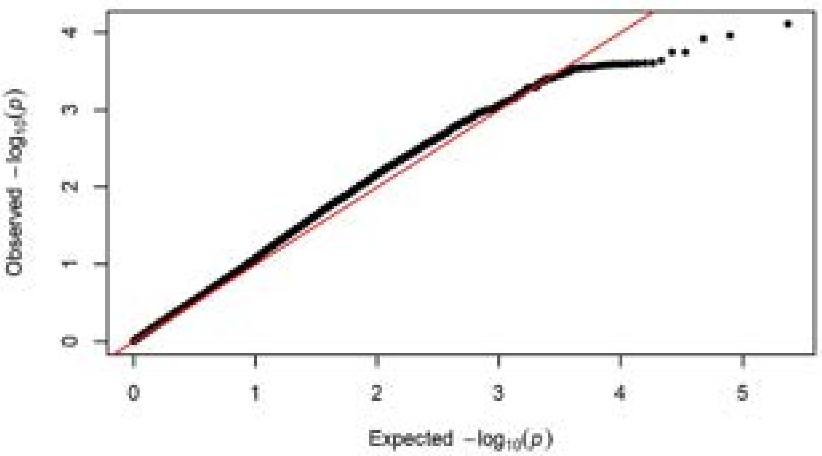

Q-Q Plot Probability of Magazine Entry Day 2 - Charles River C72 (n=3

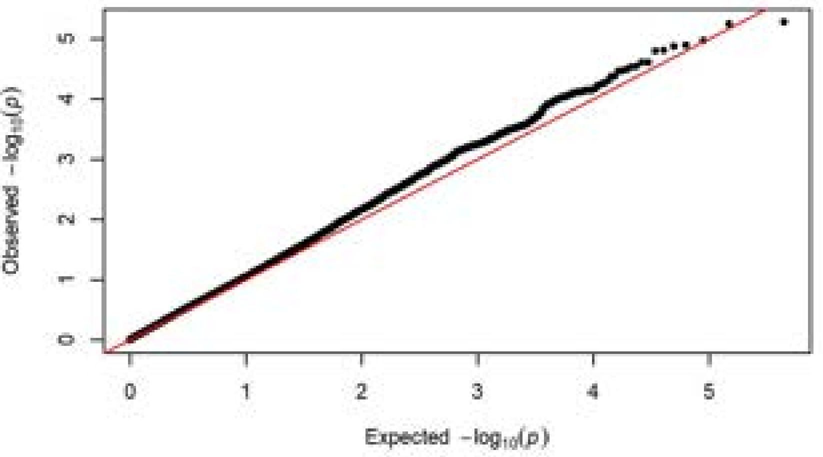

Q Plot Probability of Magazine Entry Day 3 - Charles River R09-P3/7/10 (

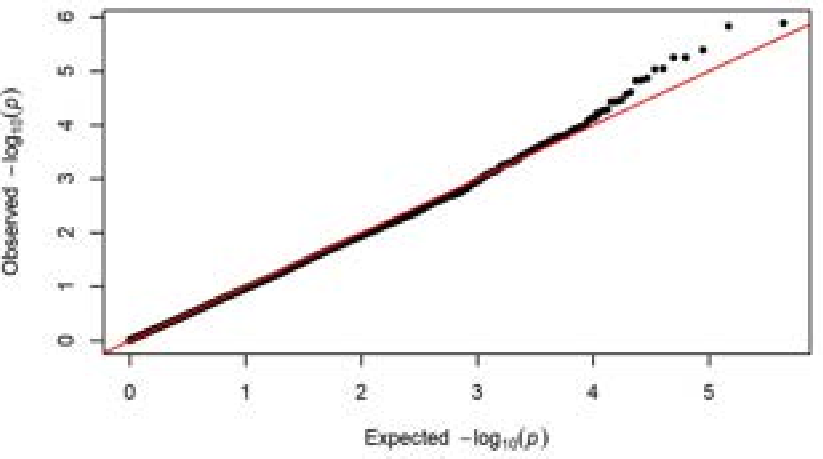

Q-Q Plot Probability of Magazine Entry Day 3 - Harlan 202A/C-208A (n=1

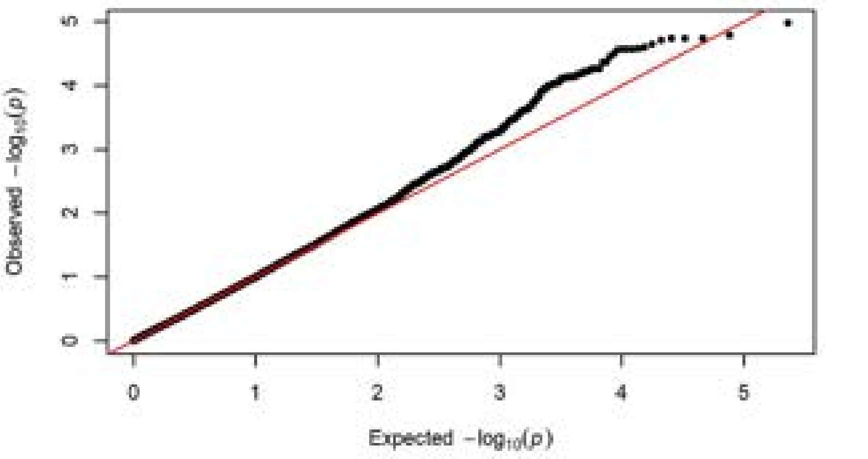

Q-Q Plot Probability of Magazine Entry Day 3 - Charles River R04 (n=6

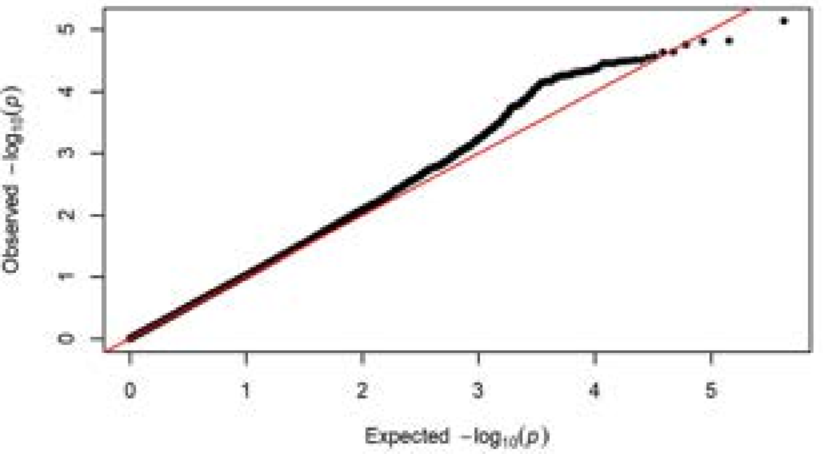

Q-Q Plot Probability of Magazine Entry Day 3 - Harlan 206 (n=758)

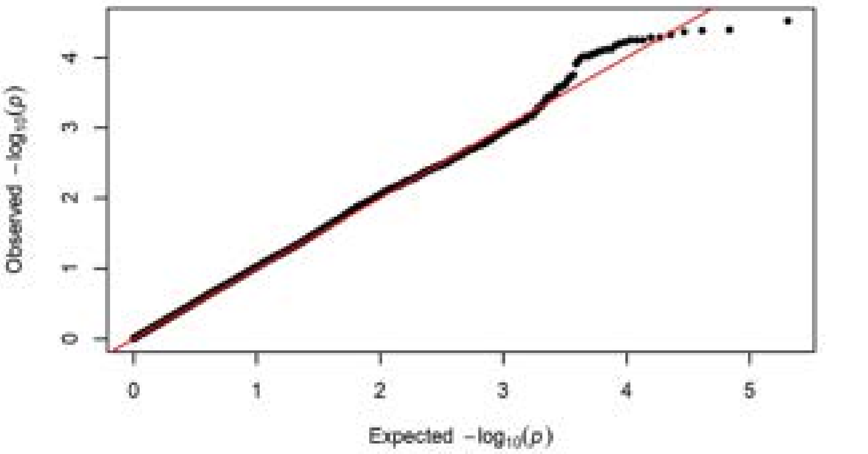

Q-Q Plot Probability of Magazine Entry Day 3 - Charles River P09 (n=2

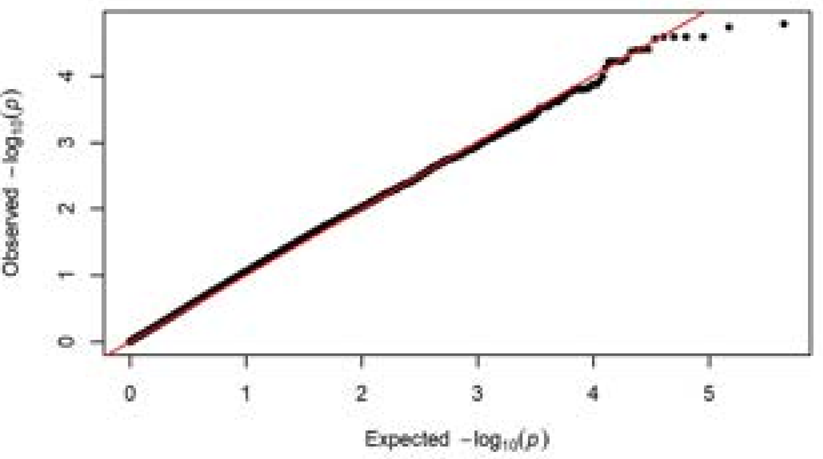

Q-Q Plot Probability of Magazine Entry Day 3 - Harlan 217 (n=351)

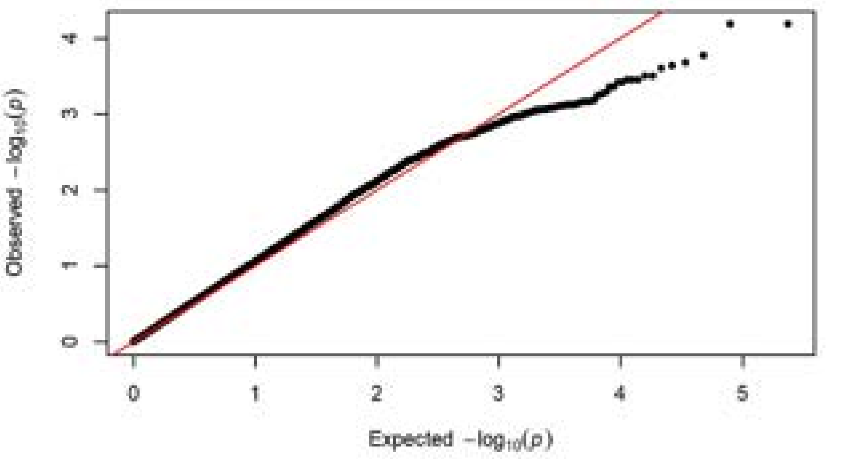

Q-Q Plot Probability of Magazine Entry Day 3 - Charles River C72 (n=3

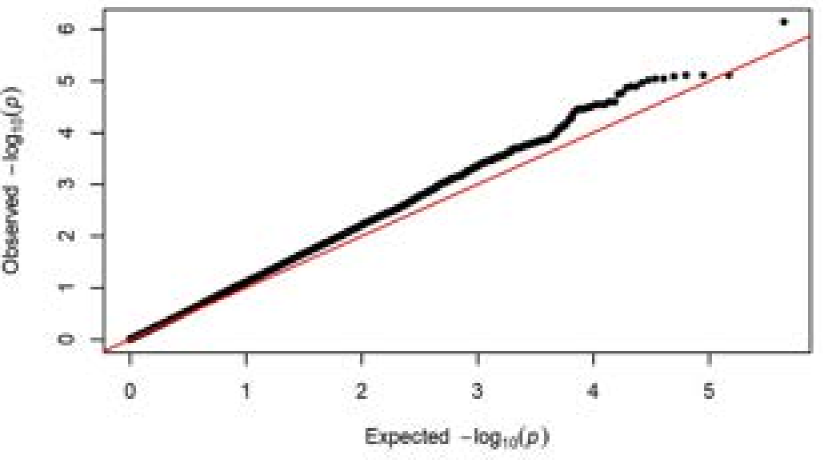

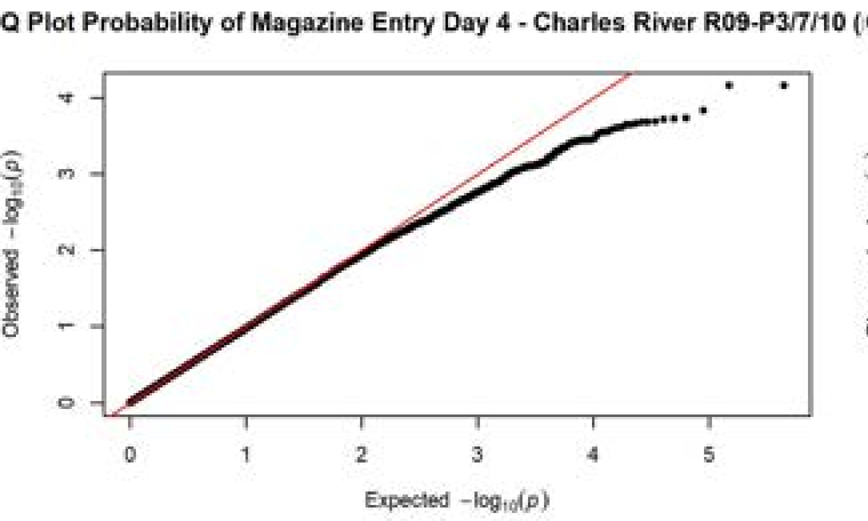
Q Plot Probability of Magazine Entry Day 4 - Charles River R09-P3/7/10 (
Observed −log10(p)
Expected −log10(p)

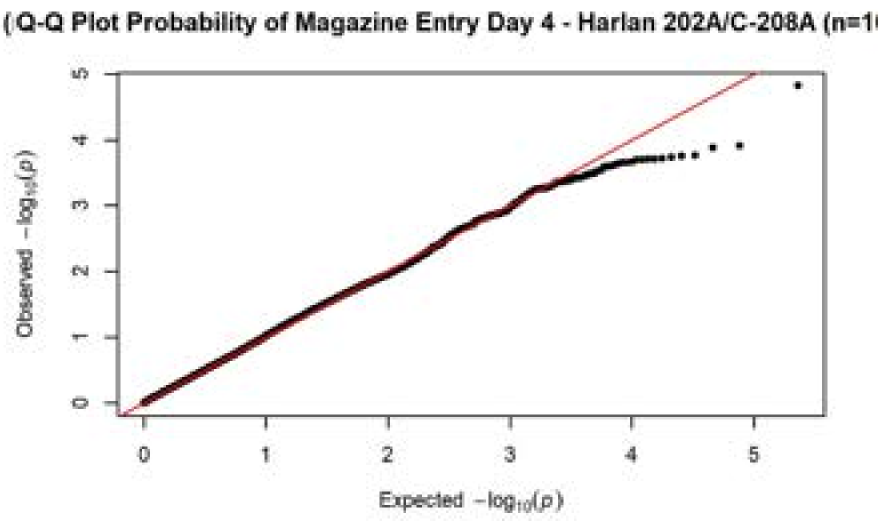
Q-Q Plot Probability of Magazine Entry Day 4 - Harlan 202A/C-208A (n=1
Observed −log10(p)
Expected −log10(p)

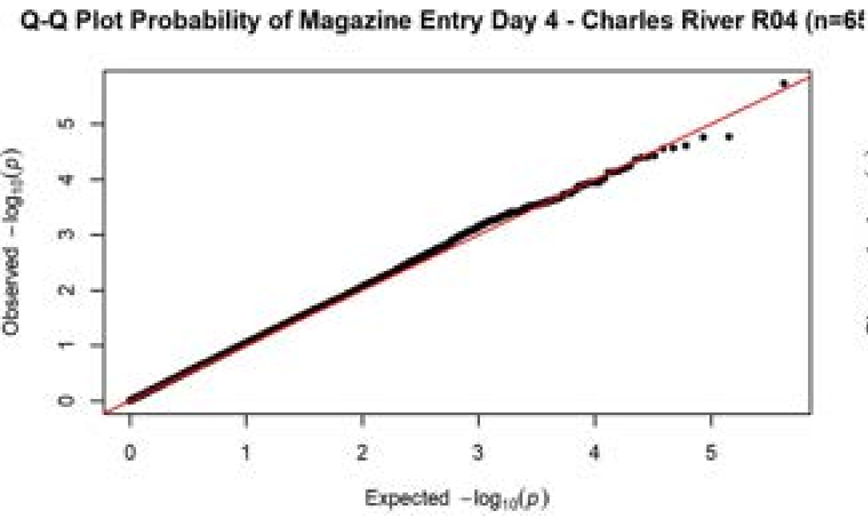
Q-Q Plot Probability of Magazine Entry Day 4 - Charles River R04 (n=6
Observed −log10(p)
Expected −log10(p)

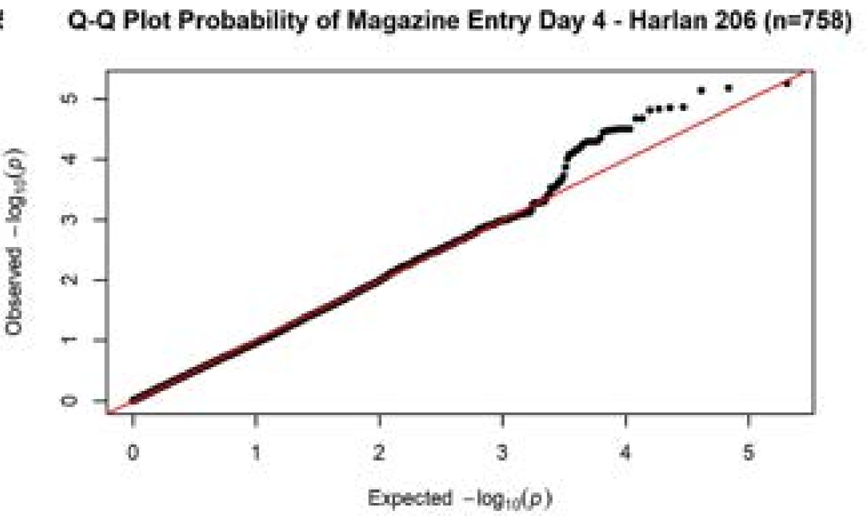
Q-Q Plot Probability of Magazine Entry Day 4 - Harlan 206 (n=758)
Observed −log10(p)
Expected −log10(p)

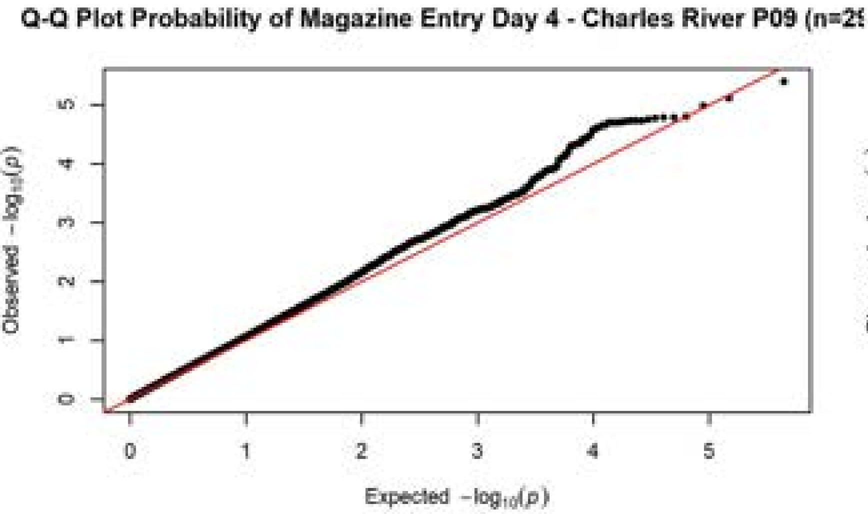
Q-Q Plot Probability of Magazine Entry Day 4 - Charles River P09 (n=2
Observed −log10(p)
Expected −log10(p)

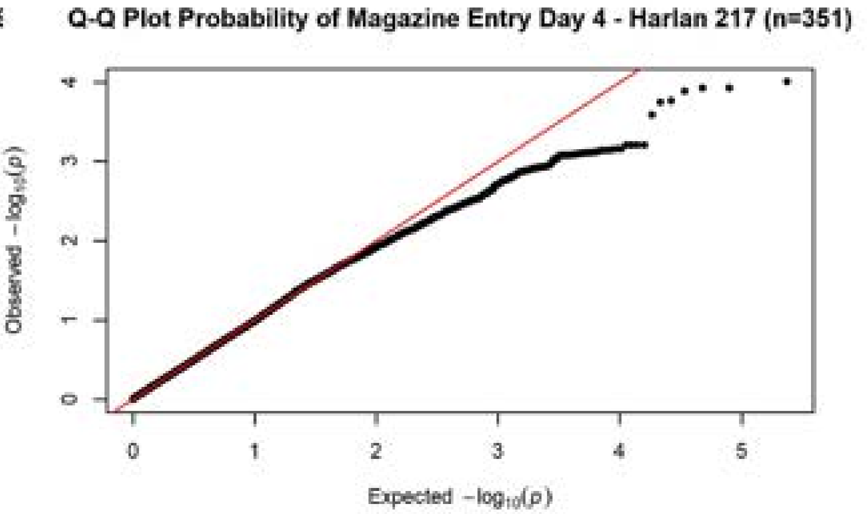
Q-Q Plot Probability of Magazine Entry Day 4 - Harlan 217 (n=351)
Observed −log10(p)
Expected −log10(p)

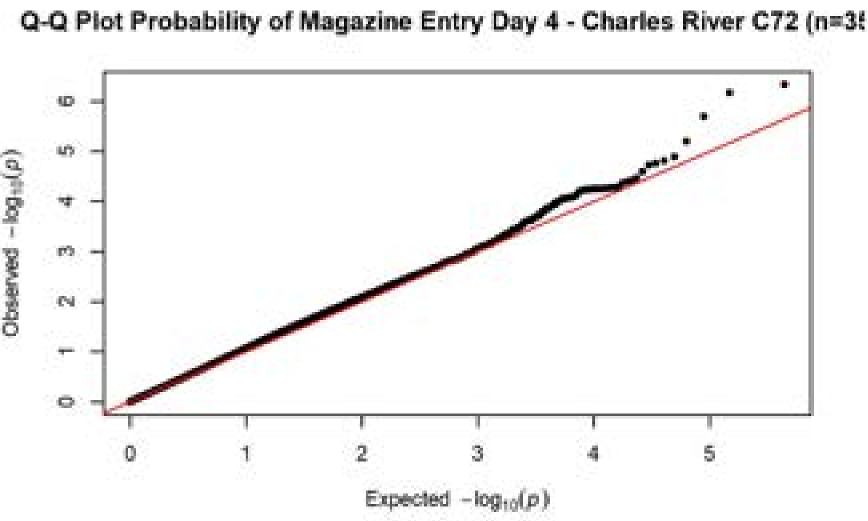
Q-Q Plot Probability of Magazine Entry Day 4 - Charles River C72 (n=3
Observed −log10(p)
Expected −log10(p)

Q Plot Probability of Magazine Entry Day 5 - Charles River R09-P3/7/10 ([illegible]

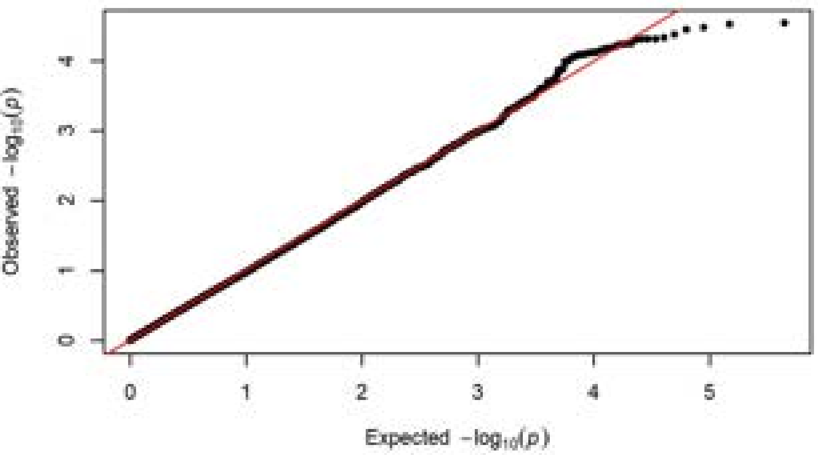

Q-Q Plot Probability of Magazine Entry Day 5 - Harlan 202A/C-208A (n=1[illegible]

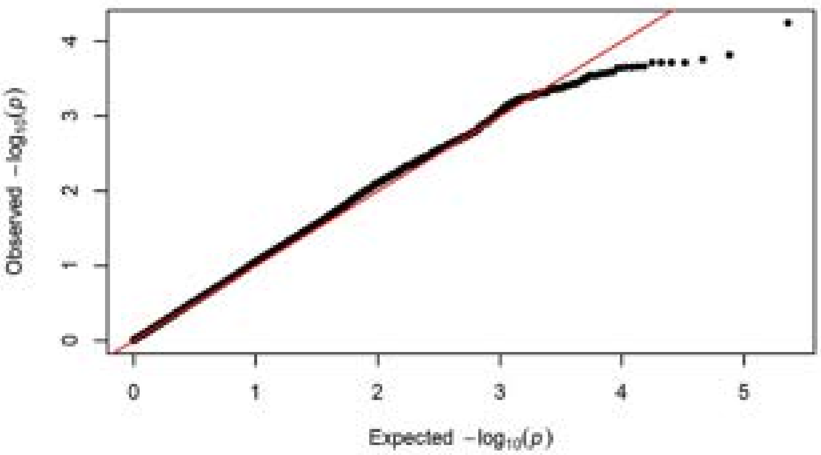

Q-Q Plot Probability of Magazine Entry Day 5 - Charles River R04 (n=6[illegible]

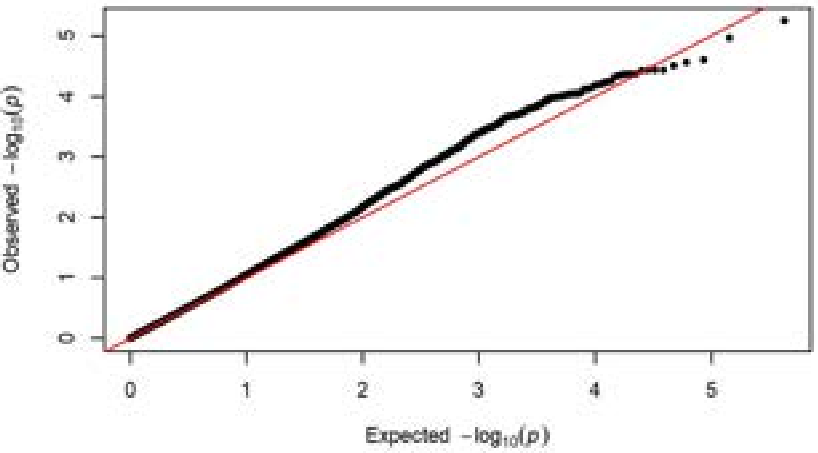

Q-Q Plot Probability of Magazine Entry Day 5 - Harlan 206 (n=758)

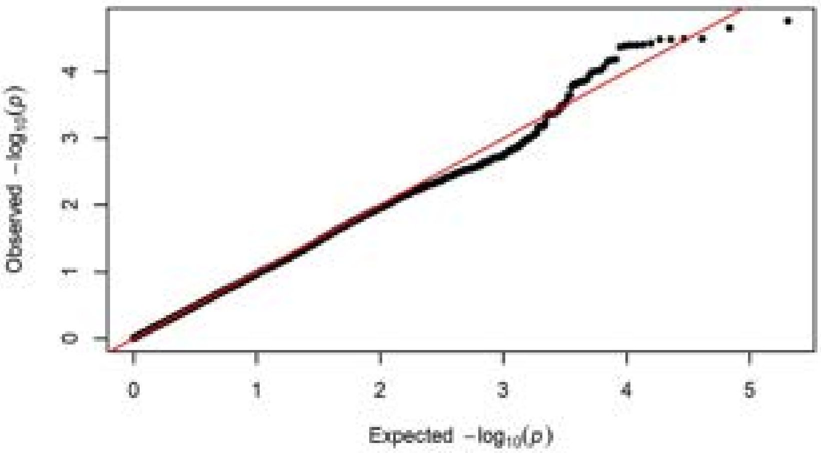

Q-Q Plot Probability of Magazine Entry Day 5 - Charles River P09 (n=2[illegible]

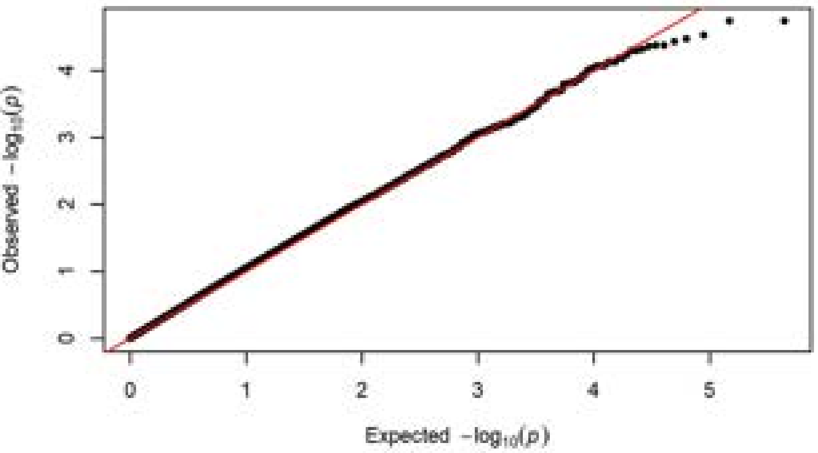

Q-Q Plot Probability of Magazine Entry Day 5 - Harlan 217 (n=351)

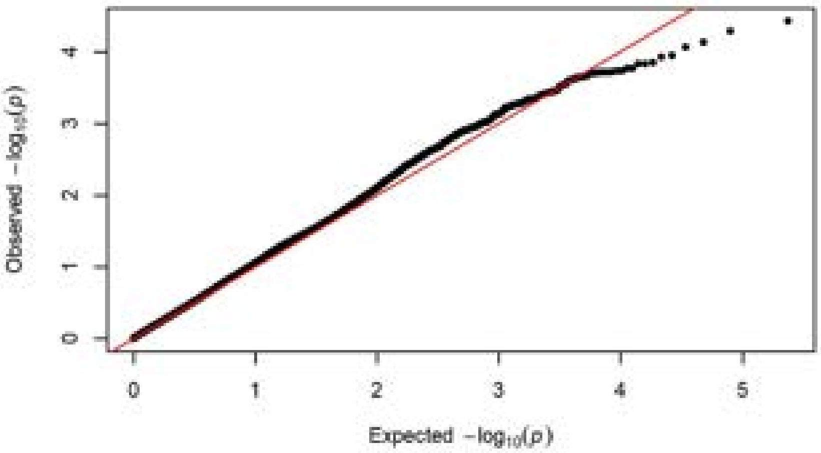

Q-Q Plot Probability of Magazine Entry Day 5 - Charles River C72 (n=3[illegible]

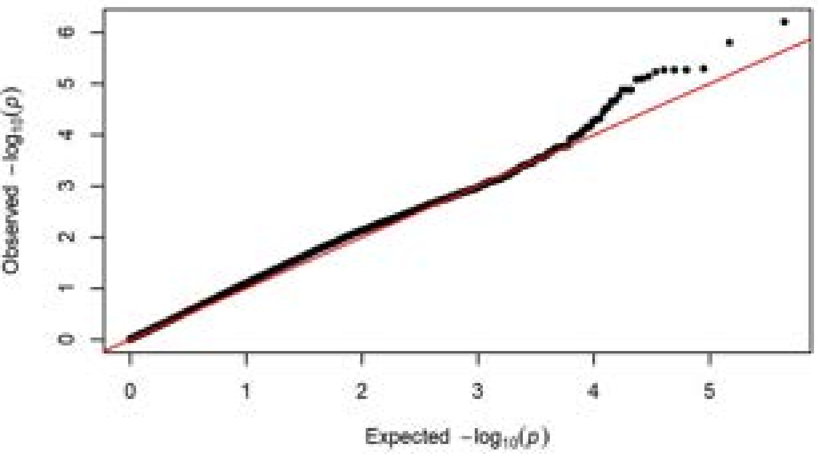

Q-Q Plot Response Bias Day 1 - Charles River R09-P3/7/10 (n=422)

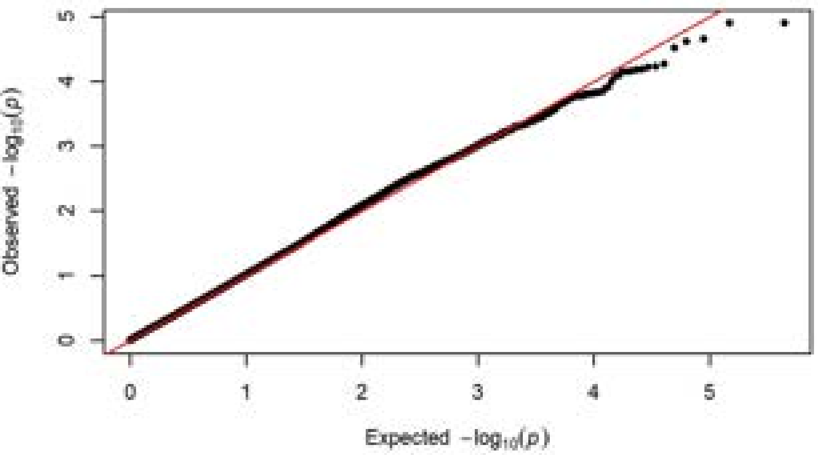

Q-Q Plot Response Bias Day 1 - Harlan 202A/C-208A (n=1094)

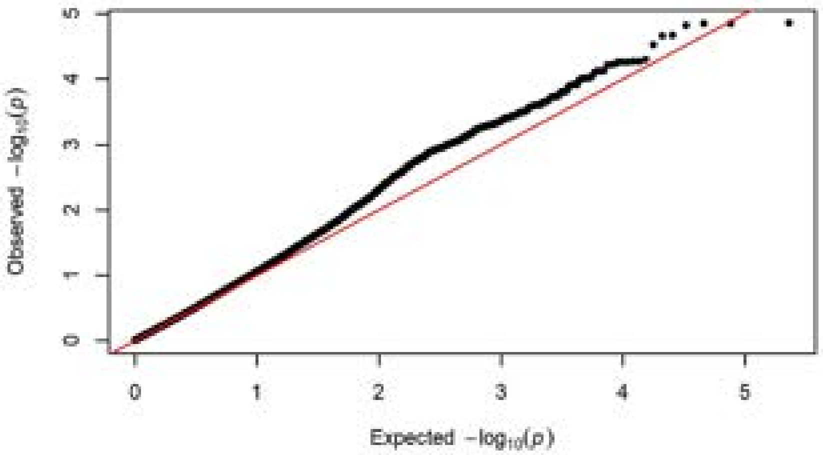

Q-Q Plot Response Bias Day 1 - Charles River R04 (n=648)

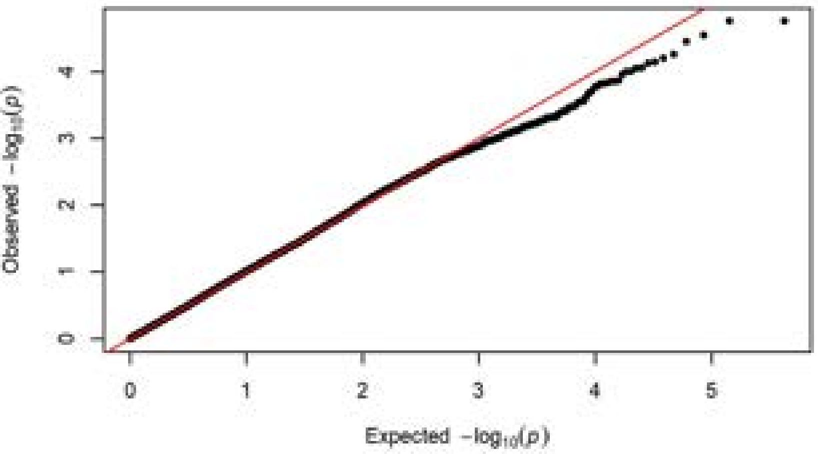

Q-Q Plot Response Bias Day 1 - Harlan 206 (n=752)

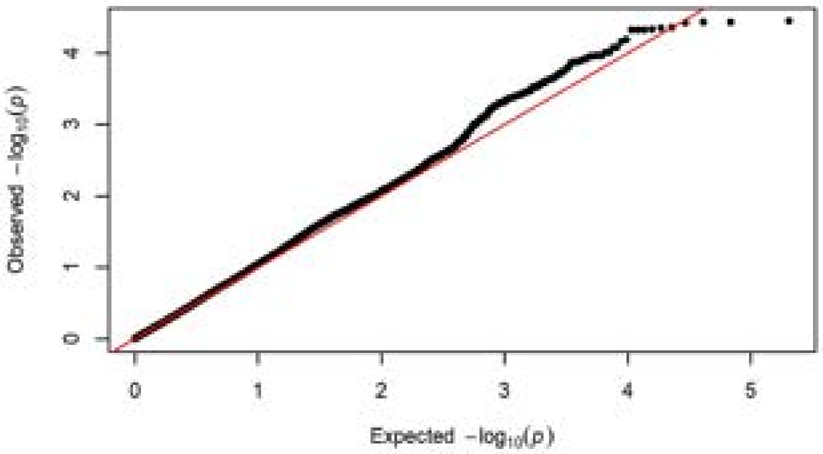

Q-Q Plot Response Bias Day 1 - Charles River P09 (n=293)

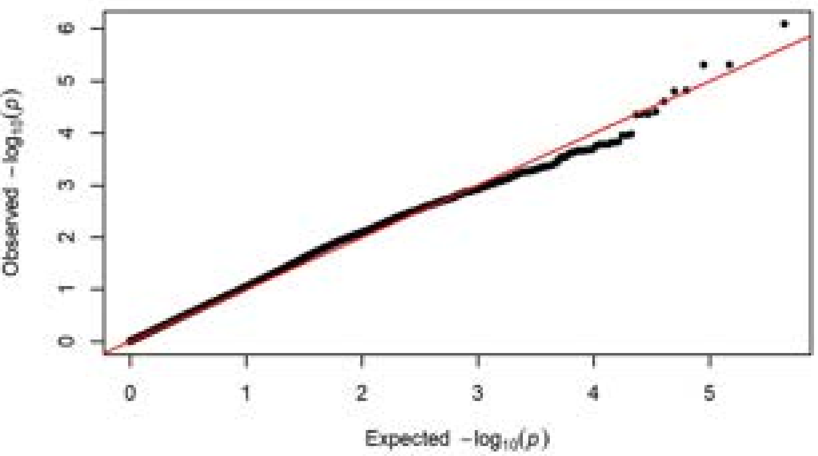

Q-Q Plot Response Bias Day 1 - Harlan 217 (n=346)

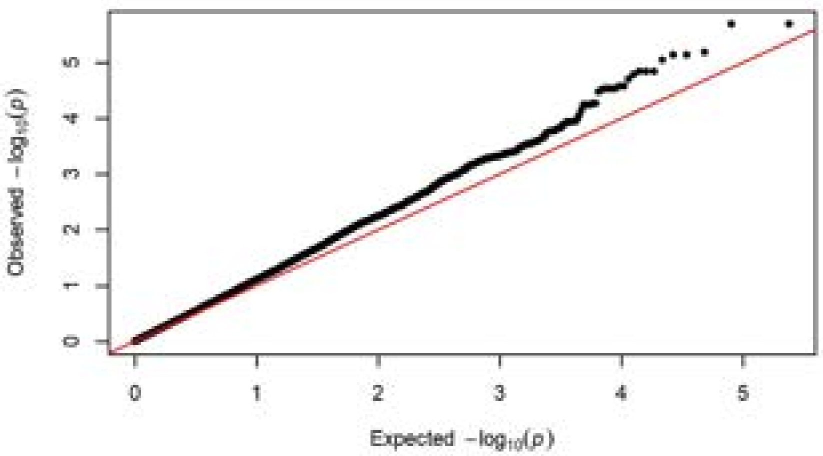

Q-Q Plot Response Bias Day 1 - Charles River C72 (n=357)

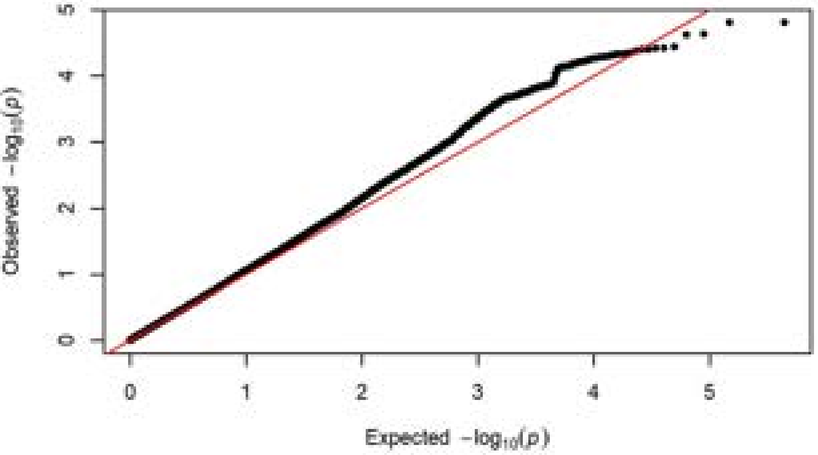

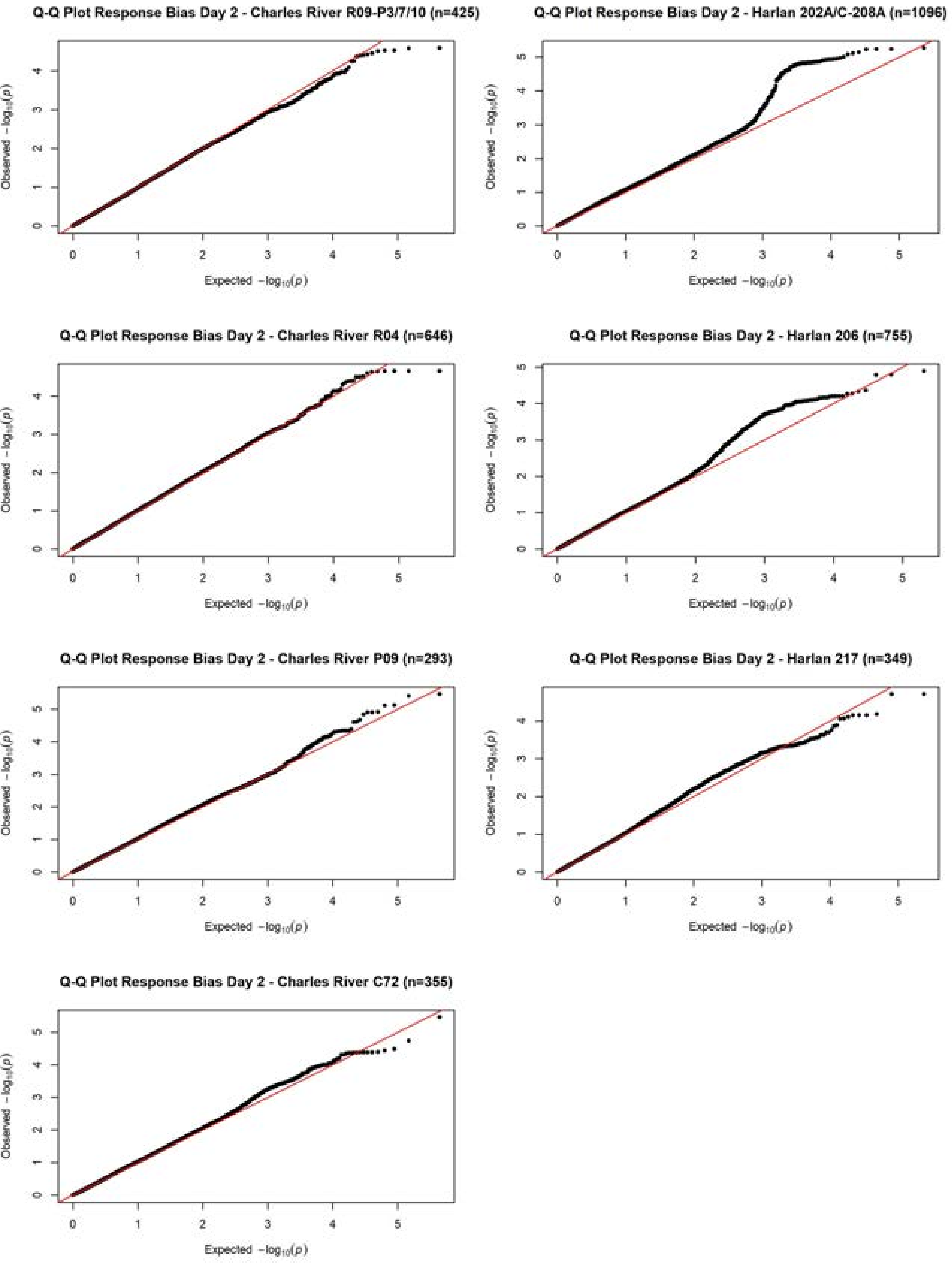
Q-Q Plot Response Bias Day 2 - Charles River R09-P3/7/10 (n=425)
Q-Q Plot Response Bias Day 2 - Harlan 202A/C-208A (n=1096)
Q-Q Plot Response Bias Day 2 - Charles River R04 (n=646)
Q-Q Plot Response Bias Day 2 - Harlan 206 (n=755)
Q-Q Plot Response Bias Day 2 - Charles River P09 (n=293)
Q-Q Plot Response Bias Day 2 - Harlan 217 (n=349)
Q-Q Plot Response Bias Day 2 - Charles River C72 (n=355)
Observed $-\log_{10}(p)$
Expected $-\log_{10}(p)$

Q-Q Plot Response Bias Day 3 - Charles River R09-P3/7/10 (n=423)

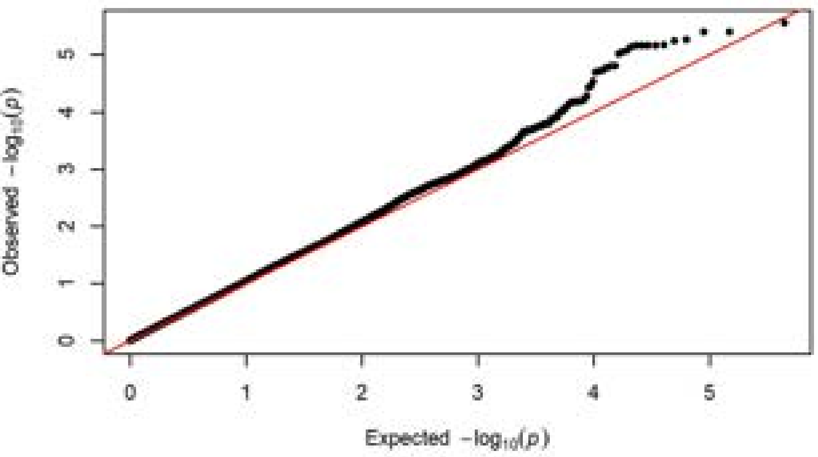

Q-Q Plot Response Bias Day 3 - Harlan 202A/C-208A (n=1095)

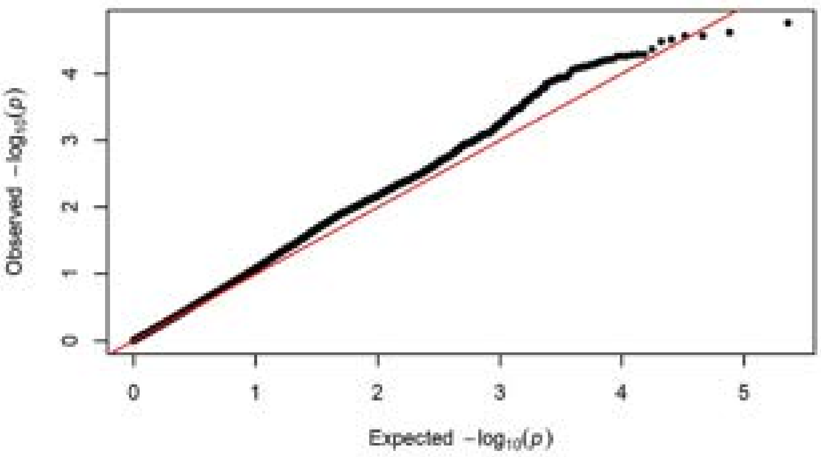

Q-Q Plot Response Bias Day 3 - Charles River R04 (n=649)

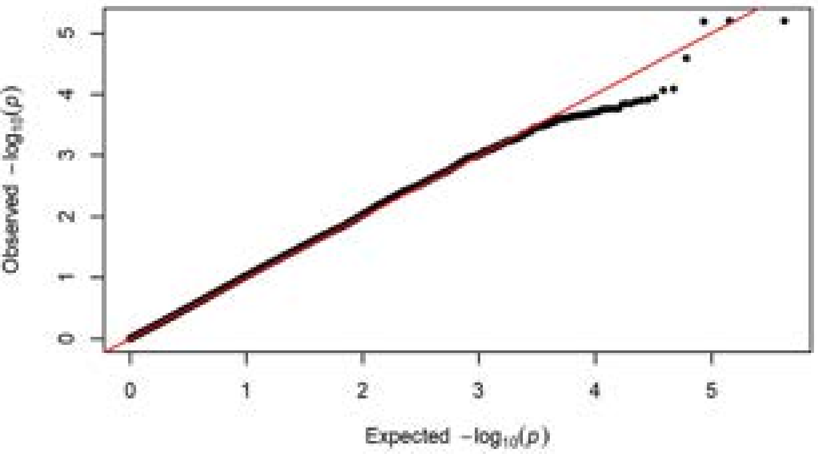

Q-Q Plot Response Bias Day 3 - Harlan 206 (n=757)

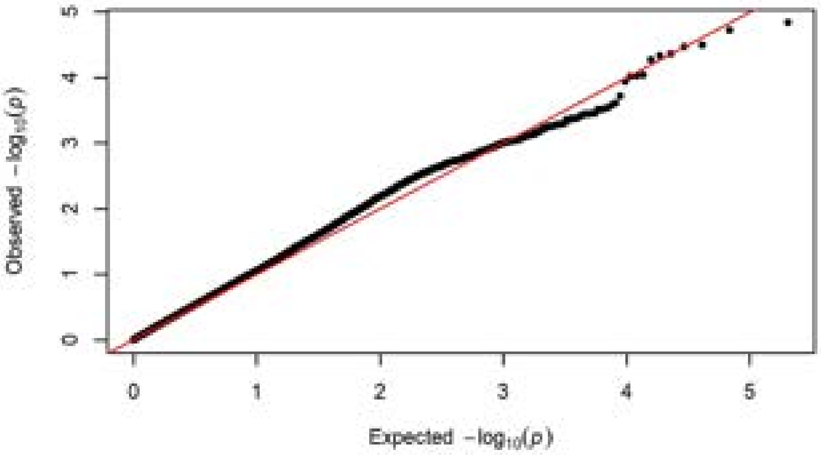

Q-Q Plot Response Bias Day 3 - Charles River P09 (n=292)

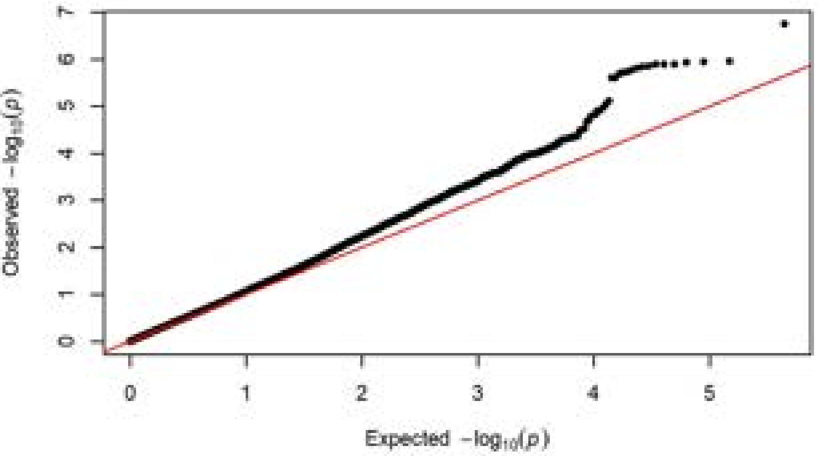

Q-Q Plot Response Bias Day 3 - Harlan 217 (n=349)

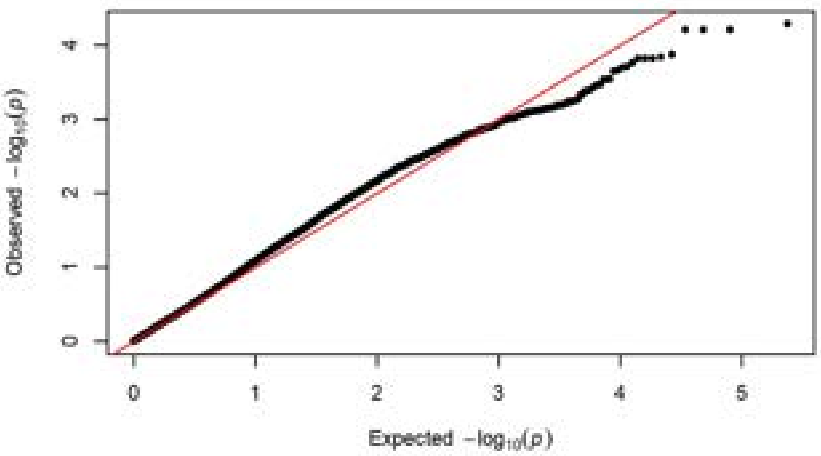

Q-Q Plot Response Bias Day 3 - Charles River C72 (n=358)

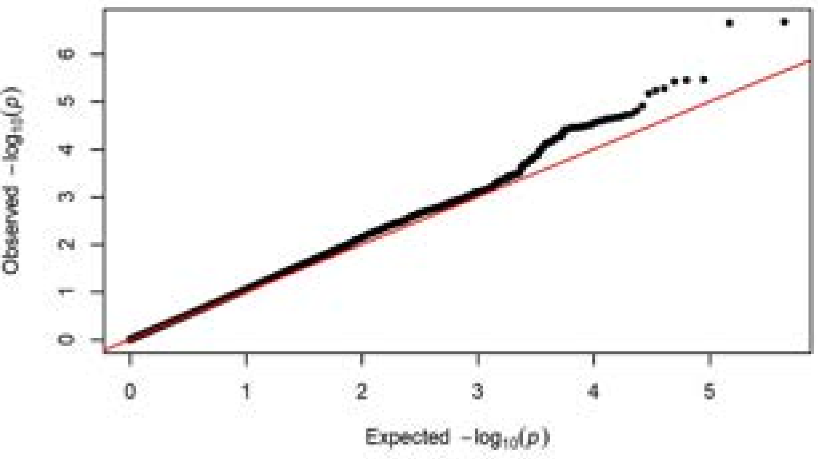

Q-Q Plot Response Bias Day 4 - Charles River R09-P3/7/10 (n=425)

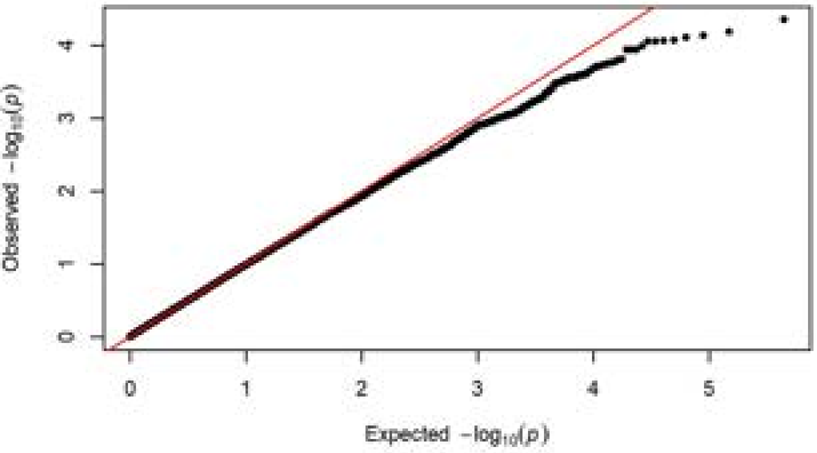

Q-Q Plot Response Bias Day 4 - Harlan 202A/C-208A (n=1099)

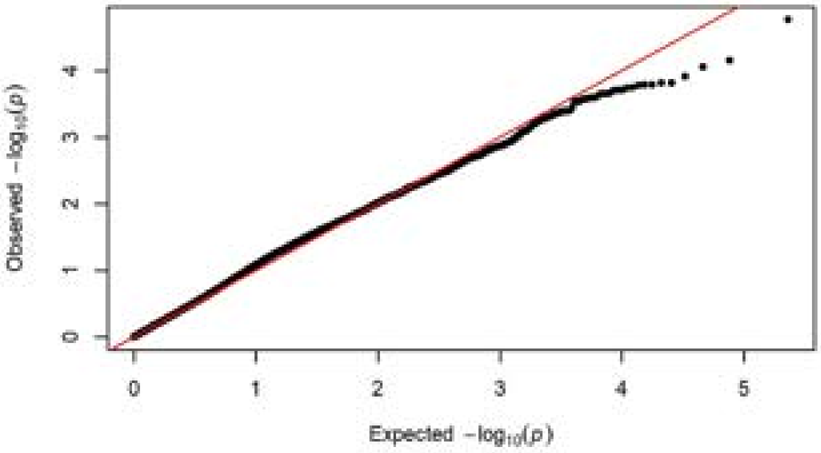

Q-Q Plot Response Bias Day 4 - Charles River R04 (n=650)

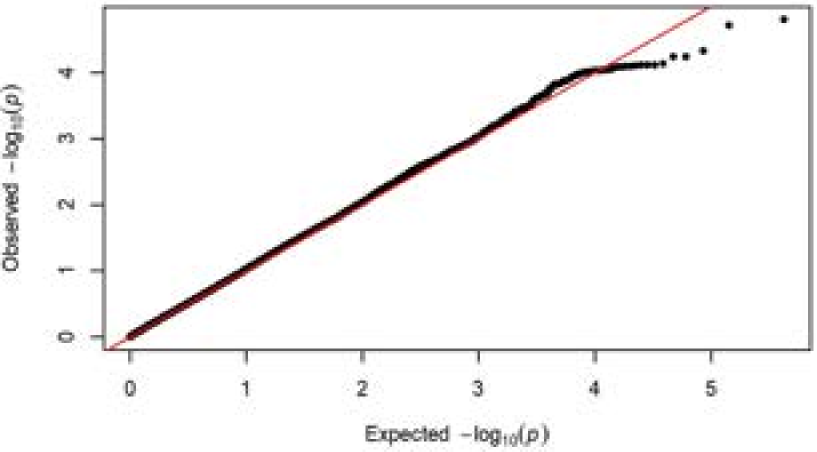

Q-Q Plot Response Bias Day 4 - Harlan 206 (n=758)

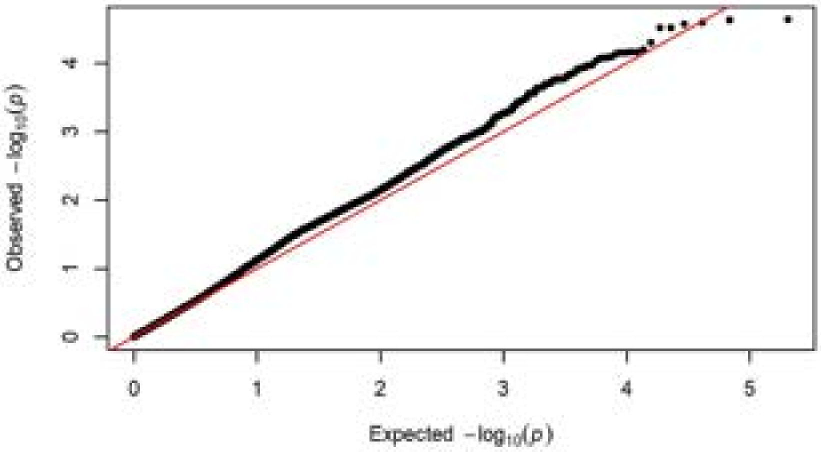

Q-Q Plot Response Bias Day 4 - Charles River P09 (n=294)

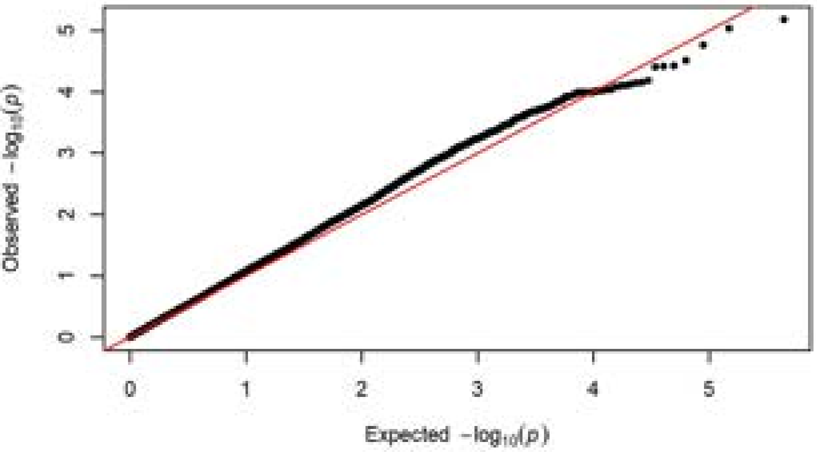

Q-Q Plot Response Bias Day 4 - Harlan 217 (n=351)

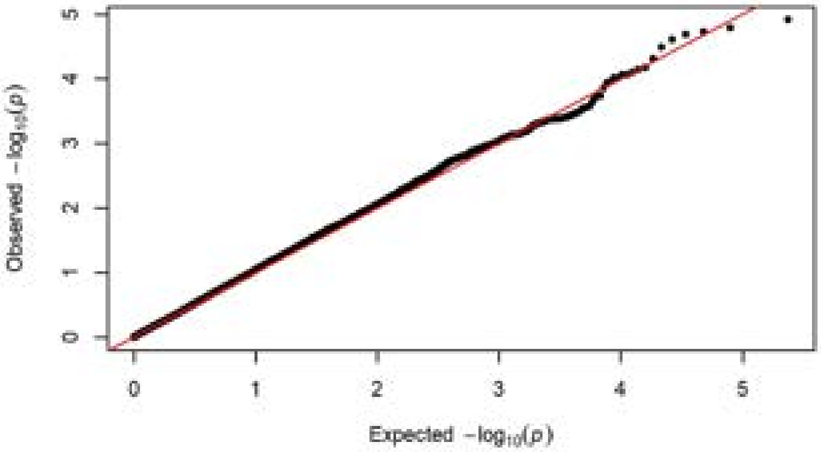

Q-Q Plot Response Bias Day 4 - Charles River C72 (n=358)

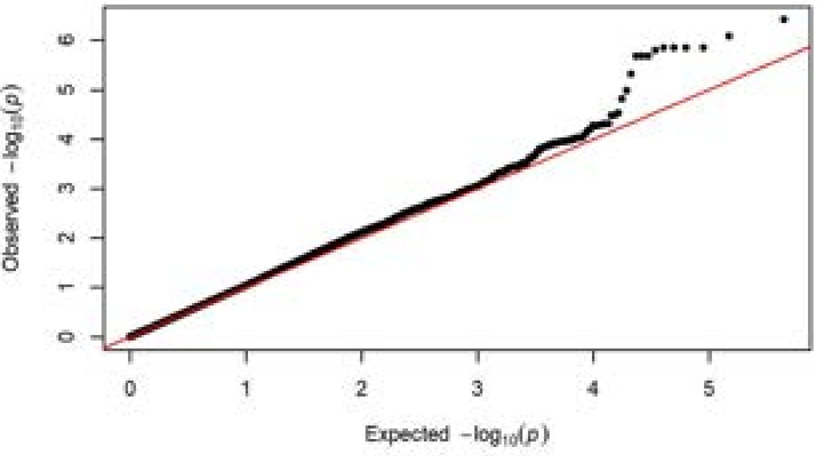

Q-Q Plot Response Bias Day 5 - Charles River R09-P3/7/10 (n=425)

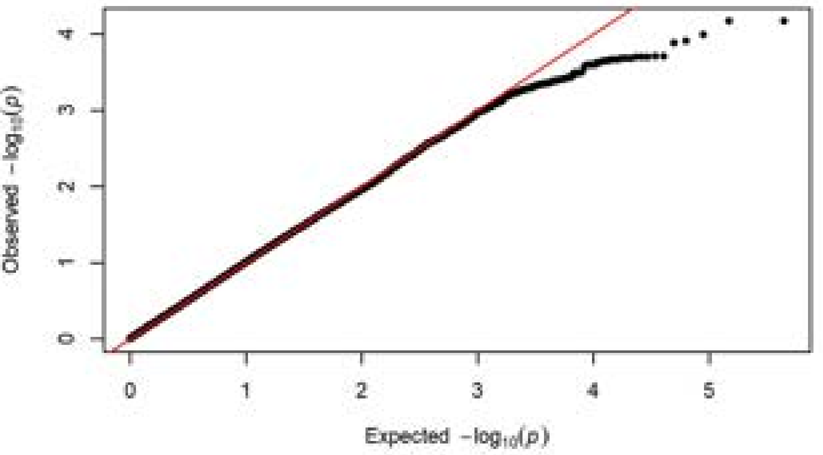

Q-Q Plot Response Bias Day 5 - Harlan 202A/C-208A (n=1099)

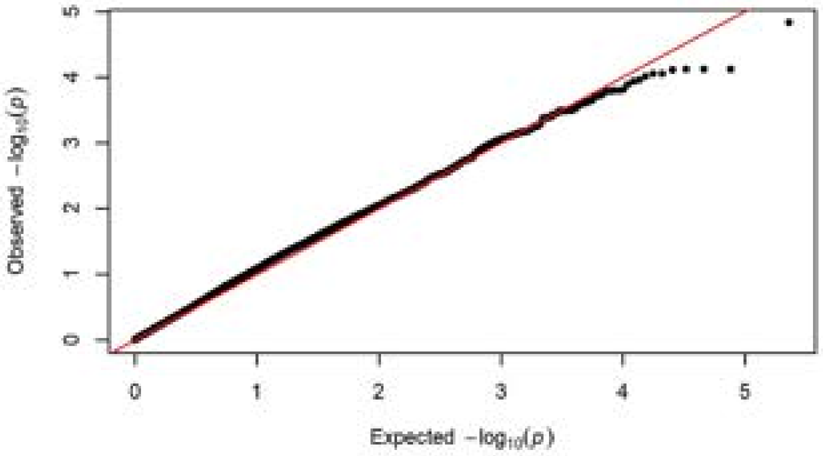

Q-Q Plot Response Bias Day 5 - Charles River R04 (n=650)

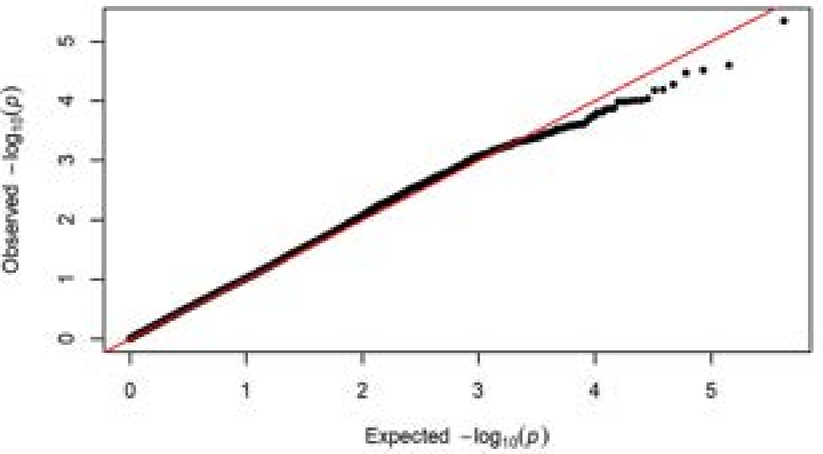

Q-Q Plot Response Bias Day 5 - Harlan 206 (n=757)

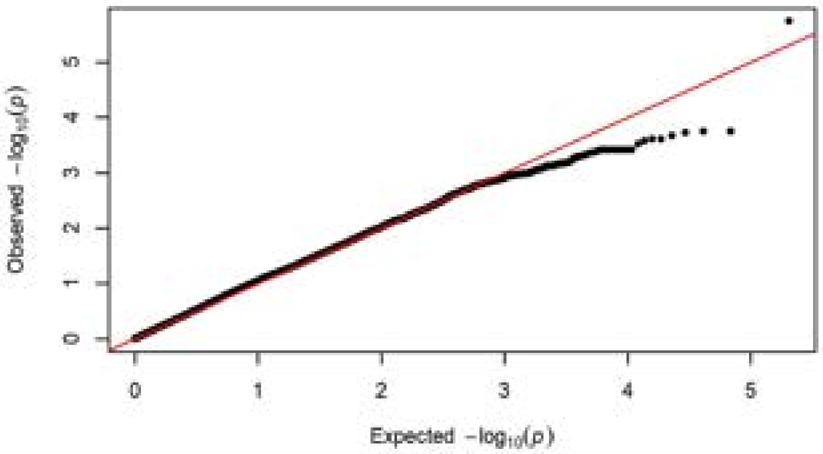

Q-Q Plot Response Bias Day 5 - Charles River P09 (n=293)

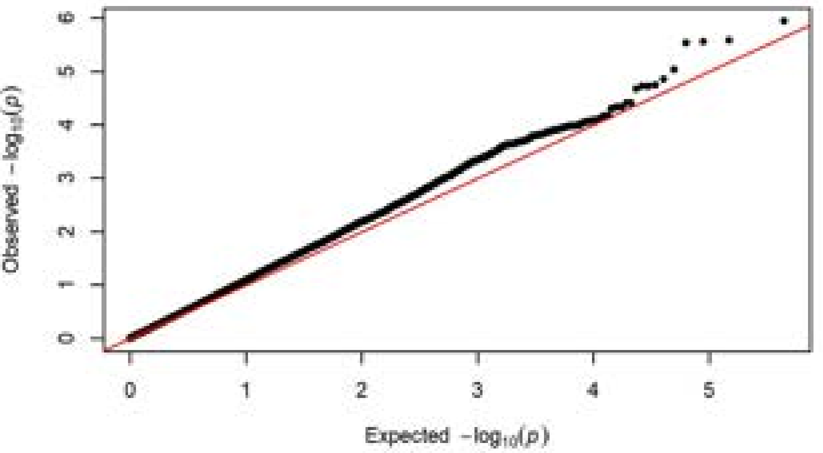

Q-Q Plot Response Bias Day 5 - Harlan 217 (n=351)

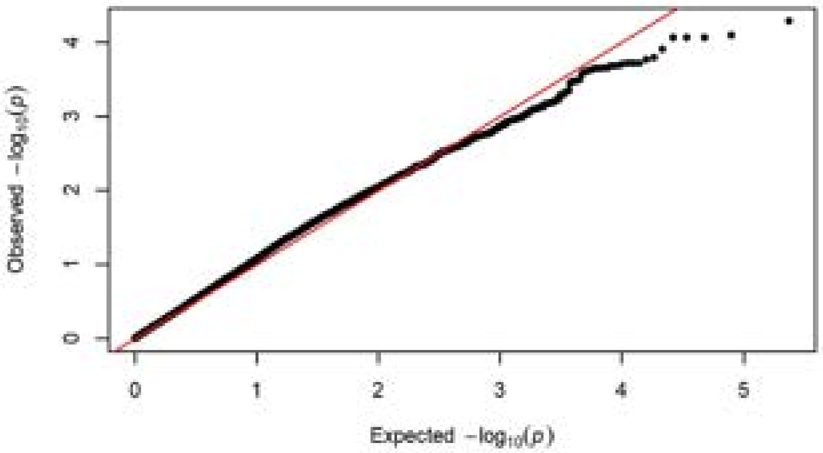

Q-Q Plot Response Bias Day 5 - Charles River C72 (n=358)

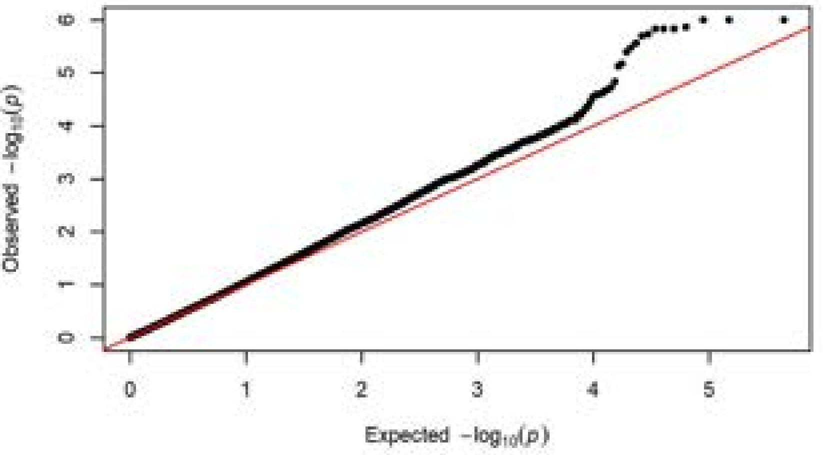

Supplement: S3 File — Each page contains Q-Q plots for the GWAS of a given day/metric for the seven subgroups. (PDF) [file pgen.1010234.s021.pdf]
