# Supplementary material for: Genetic characterization of outbred Sprague Dawley rats and utility for genome-wide association studies
Source: PLoS Genet. 2022 May 31;18(5):e1010234. doi: 10.1371/journal.pgen.1010234 (PMC9187121; doi:10.1371/journal.pgen.1010234)

Q-Q Plot Average Latency to Lever Press Day 1 - Meta-analysis of 7 Subgroups - 64k SNPs (n=3903)

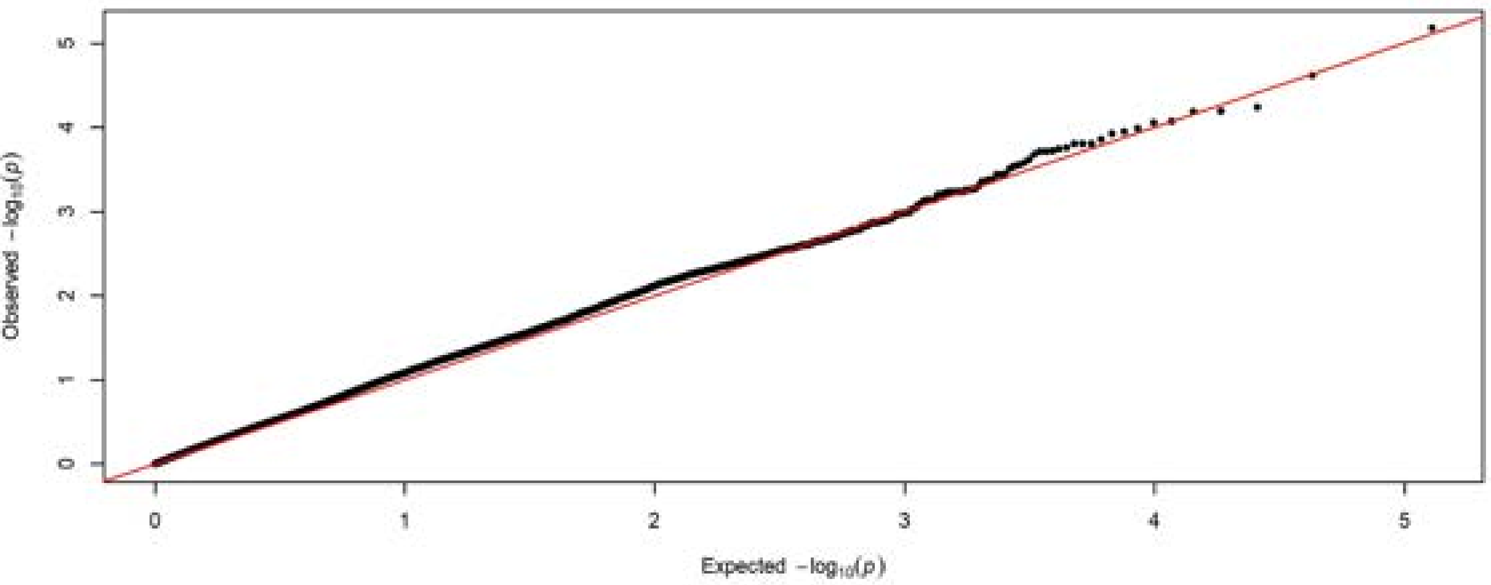

Q-Q Plot Average Latency to Lever Press Day 1 - Charles River 4 Subgroups - 198k SNPs (n=1728)

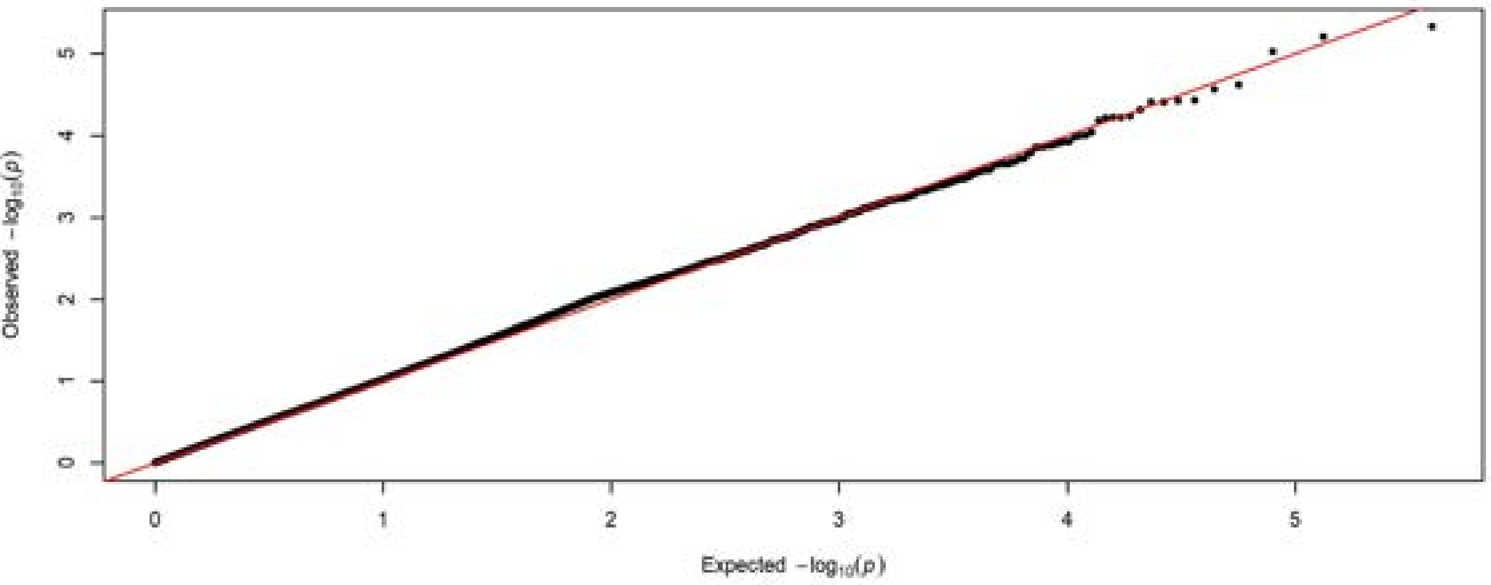

Q-Q Plot Average Latency to Lever Press Day 1 - Harlan 3 Subgroups - 83k SNPs (n=2175)

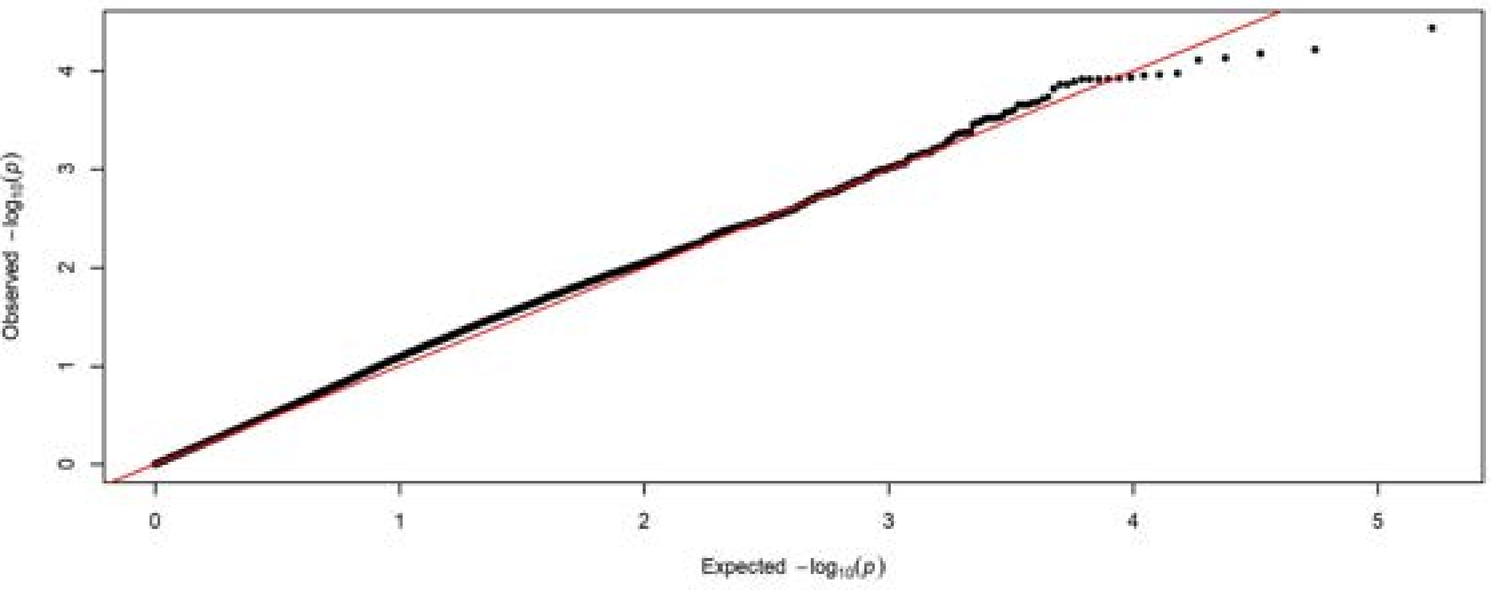

Q-Q Plot Average Latency to Lever Press Day 2 - Meta-analysis of 7 Subgroups - 64k SNPs (n=3934)

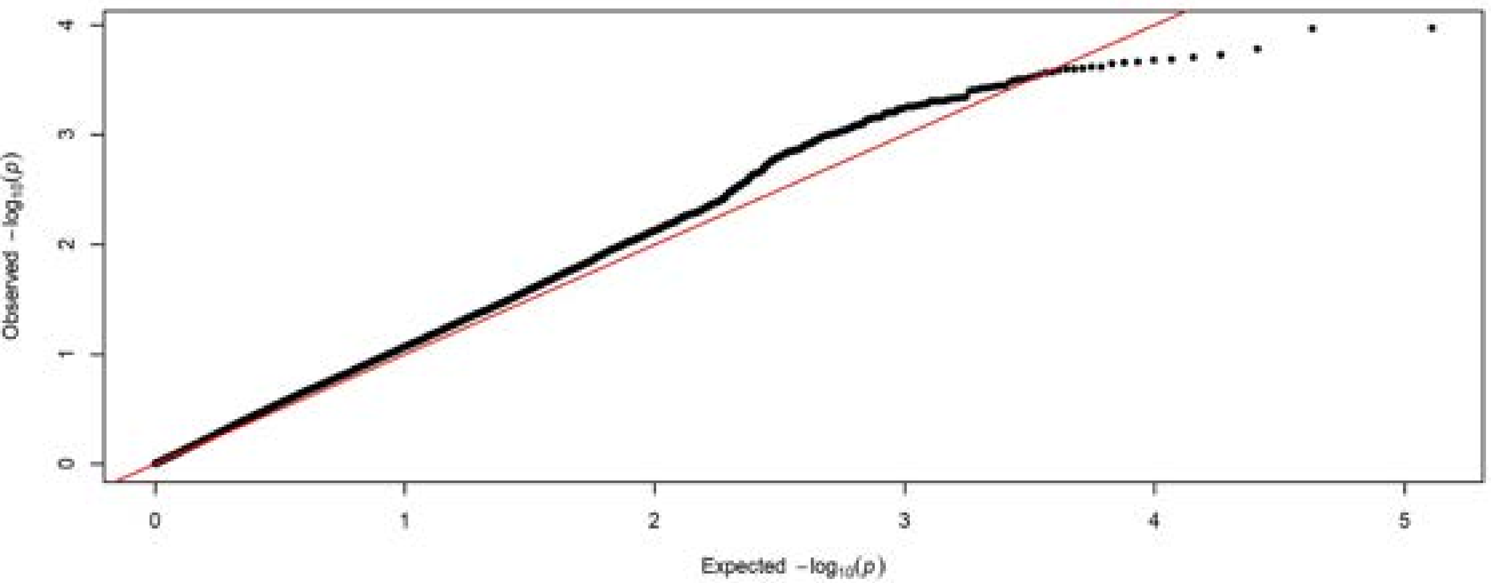

Q-Q Plot Average Latency to Lever Press Day 2 - Charles River 4 Subgroups - 198k SNPs (n=1726)

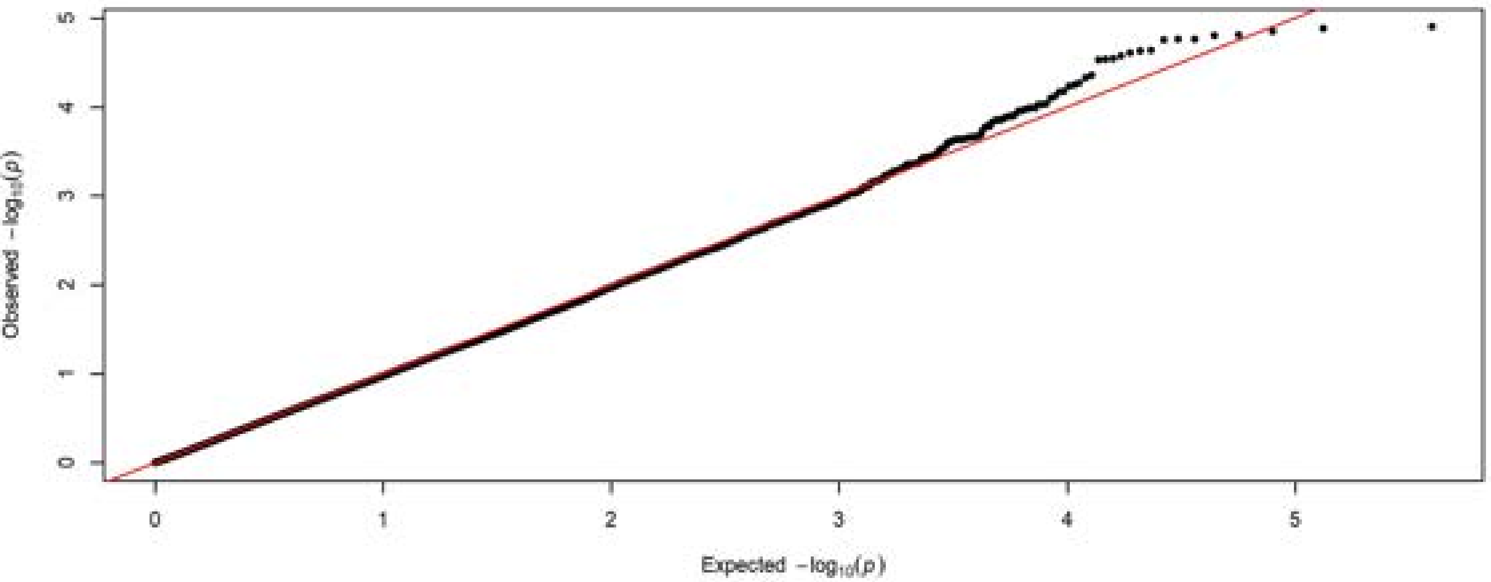

Q-Q Plot Average Latency to Lever Press Day 2 - Harlan 3 Subgroups - 83k SNPs (n=2208)

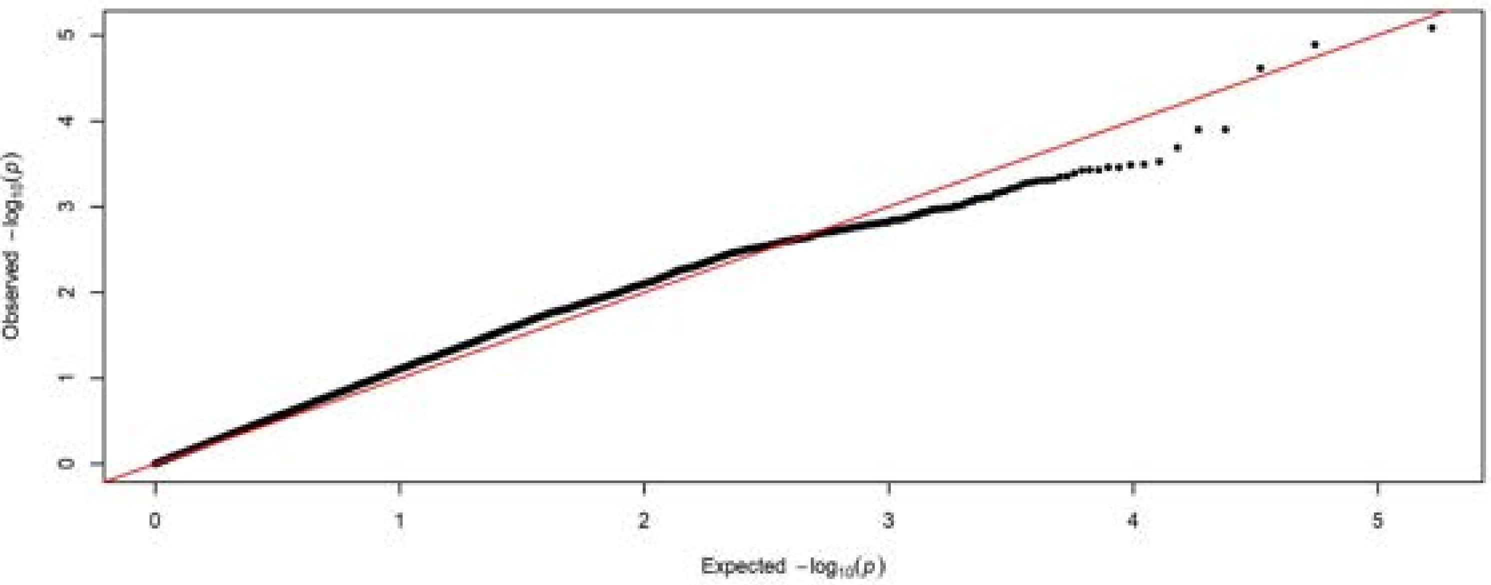

Q-Q Plot Average Latency to Lever Press Day 3 - Meta-analysis of 7 Subgroups - 64k SNPs (n=3932)

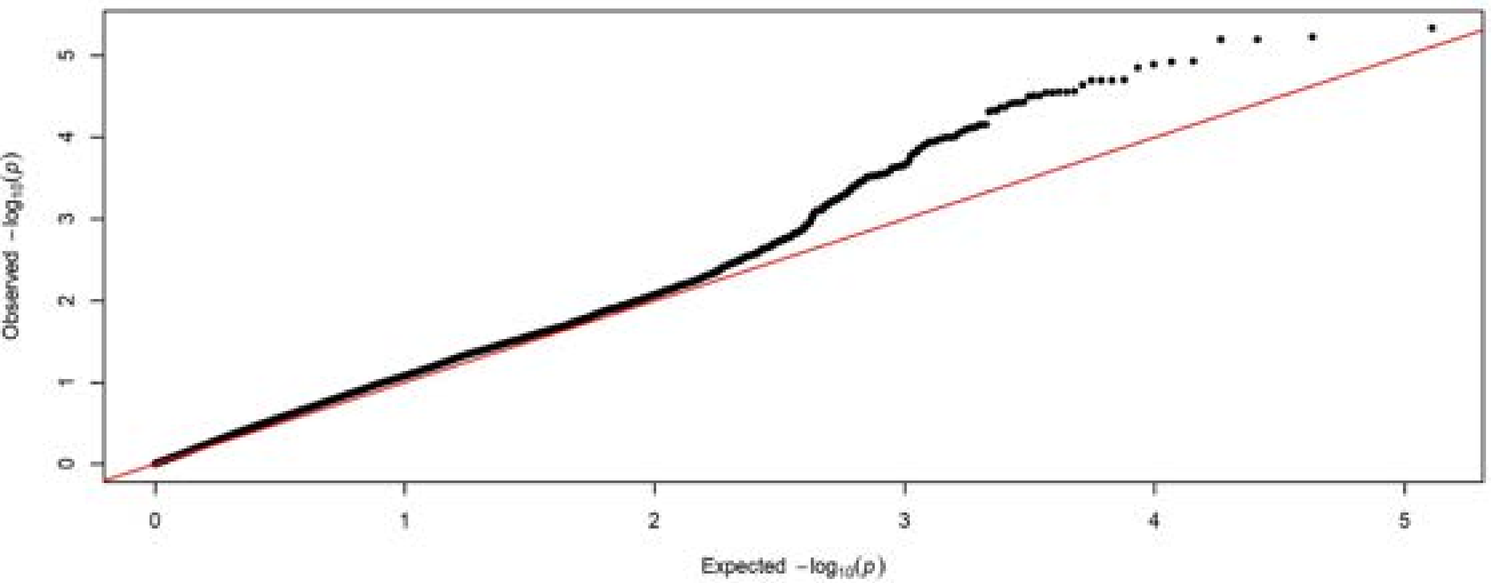

Q-Q Plot Average Latency to Lever Press Day 3 - Charles River 4 Subgroups - 198k SNPs (n=1727)

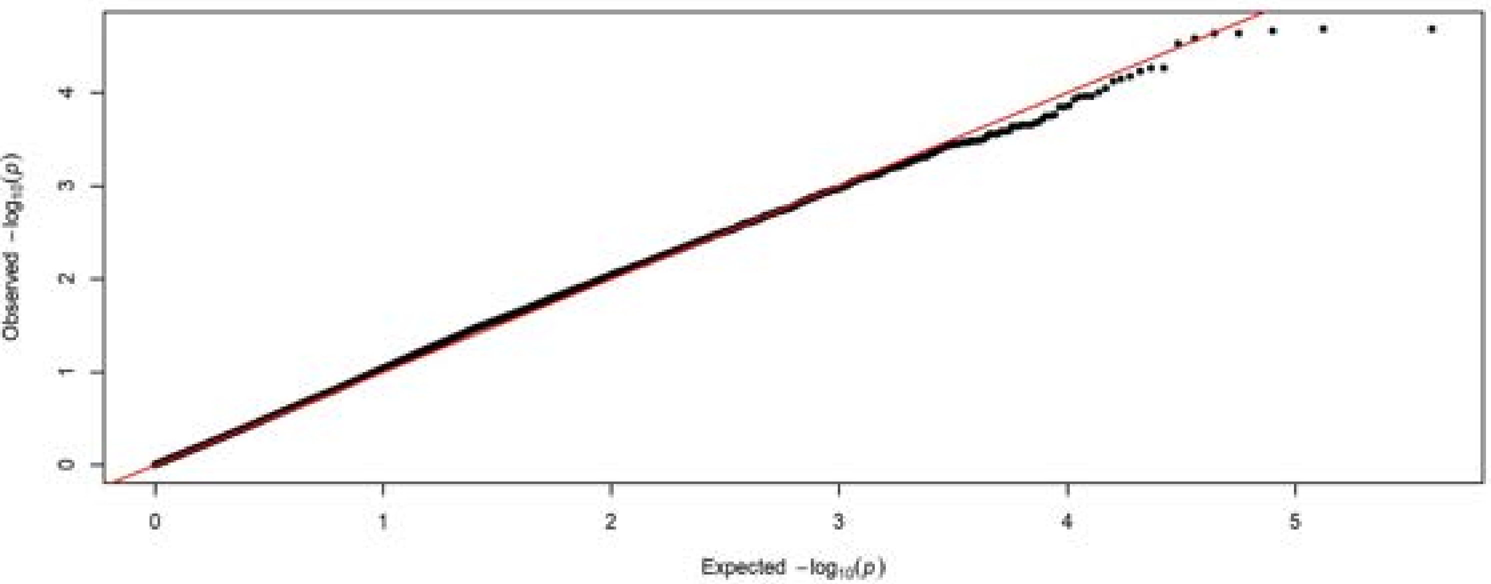

Q-Q Plot Average Latency to Lever Press Day 3 - Harlan 3 Subgroups - 83k SNPs (n=2205)

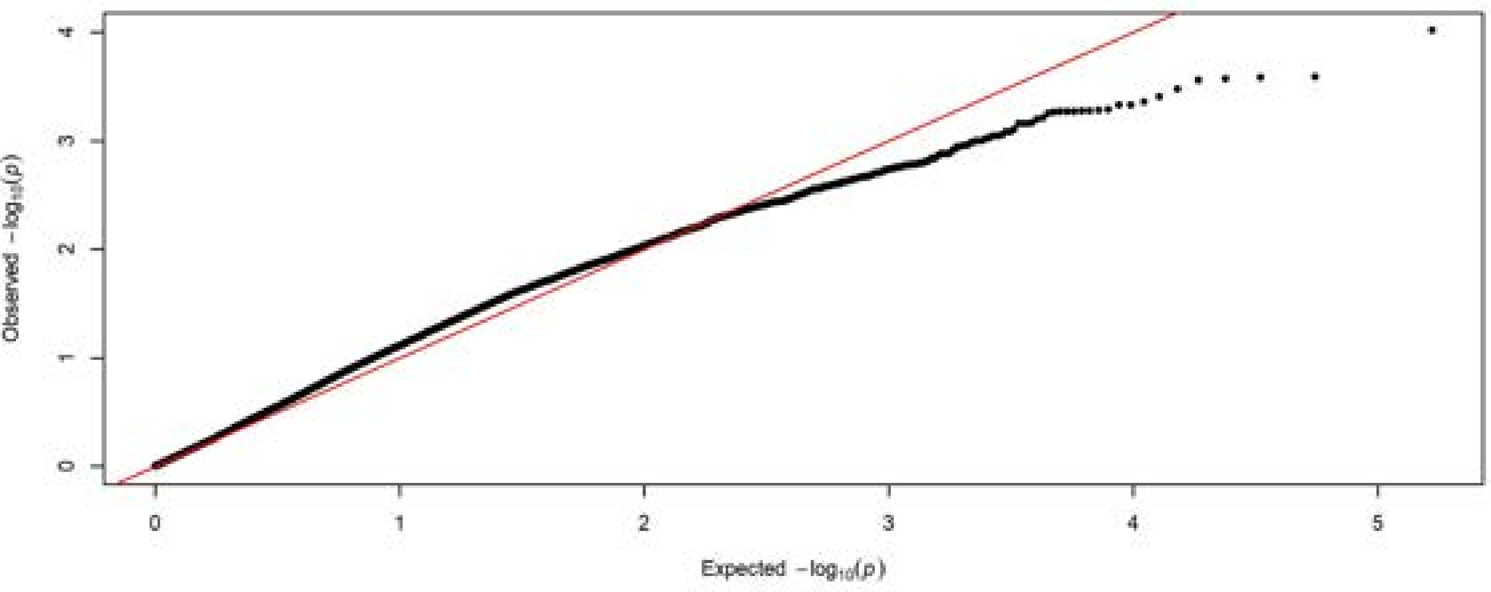

Q-Q Plot Average Latency to Lever Press Day 4 - Meta-analysis of 7 Subgroups - 64k SNPs (n=3936)

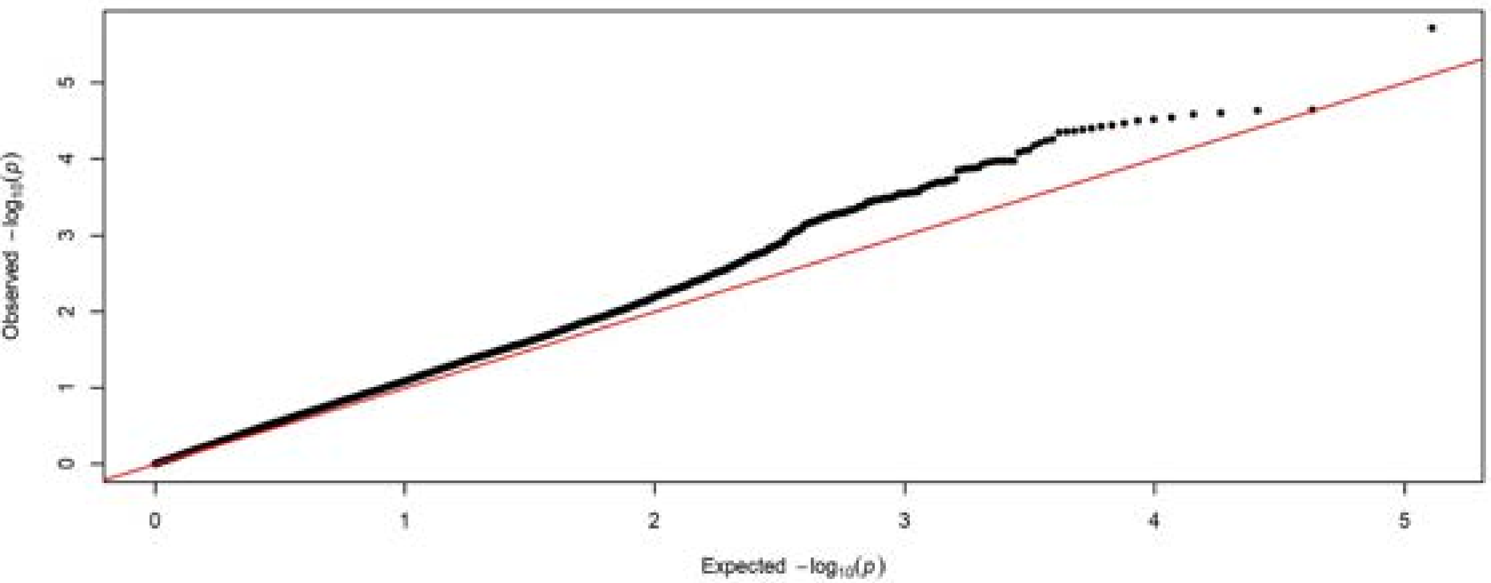

Q-Q Plot Average Latency to Lever Press Day 4 - Charles River 4 Subgroups - 198k SNPs (n=1728)

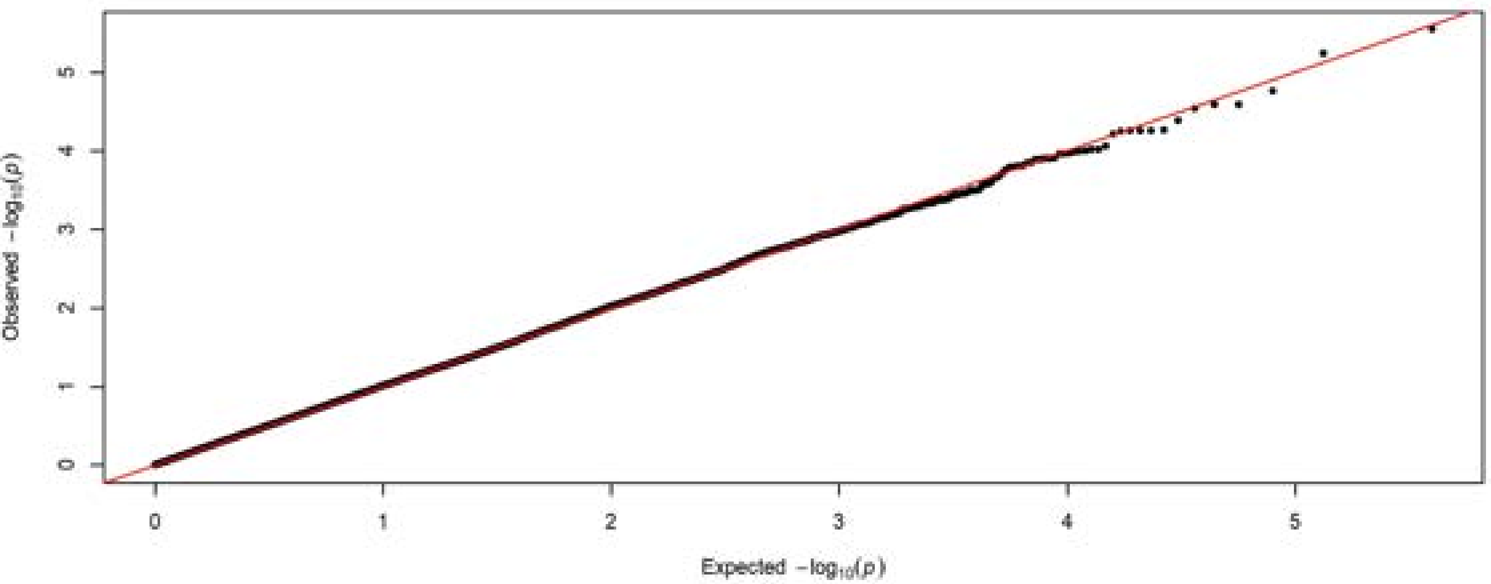

Q-Q Plot Average Latency to Lever Press Day 4 - Harlan 3 Subgroups - 83k SNPs (n=2208)

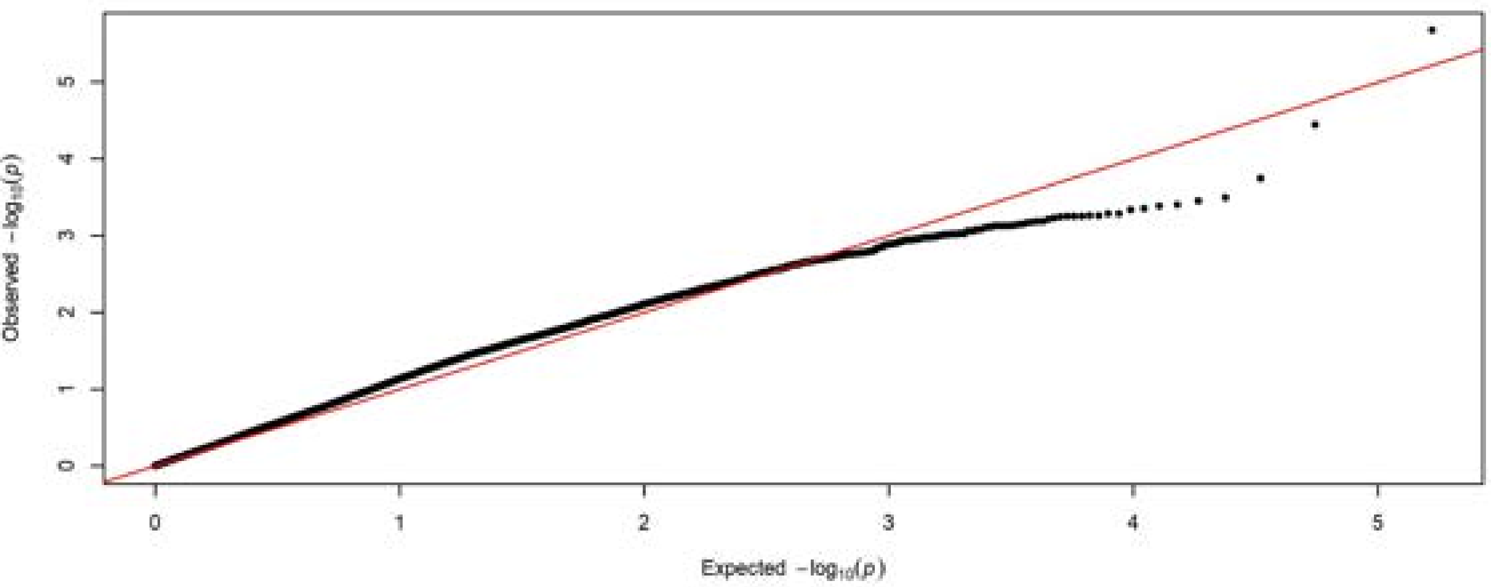

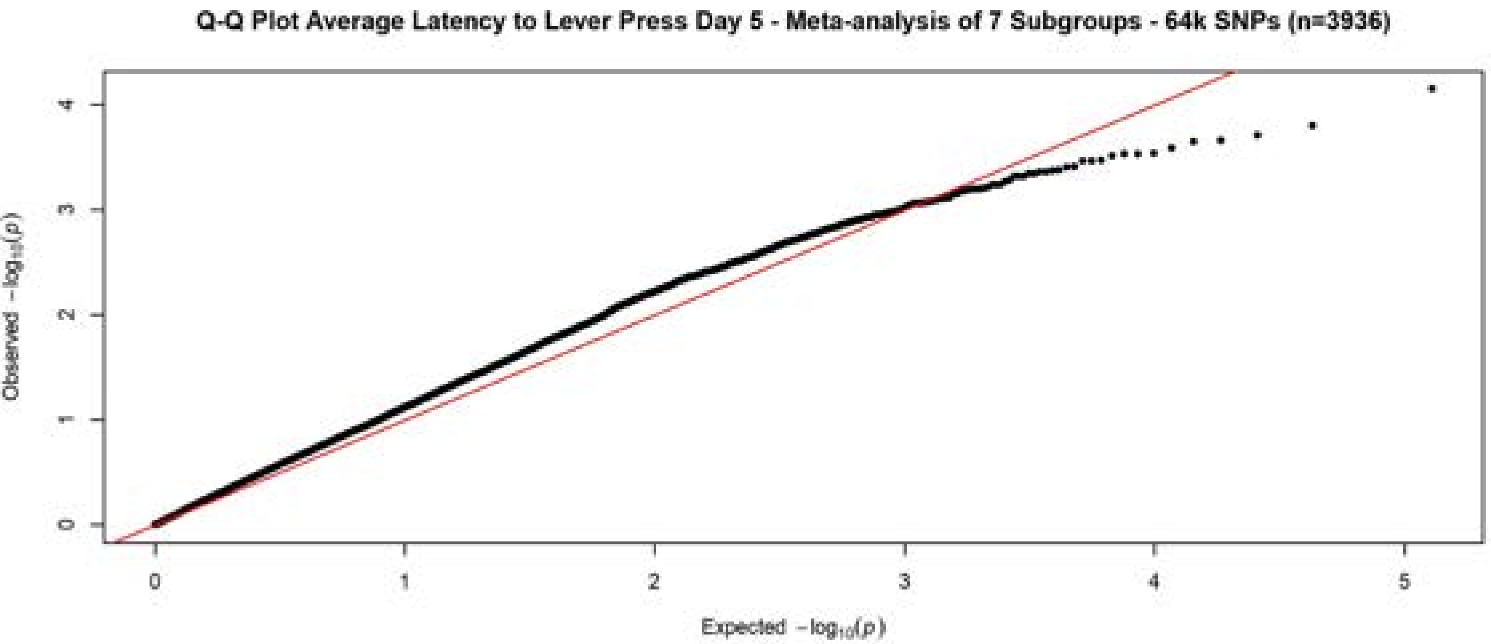
Q-Q Plot Average Latency to Lever Press Day 5 - Meta-analysis of 7 Subgroups - 64k SNPs (n=3936)
Observed −log10(p)
Expected −log10(p)

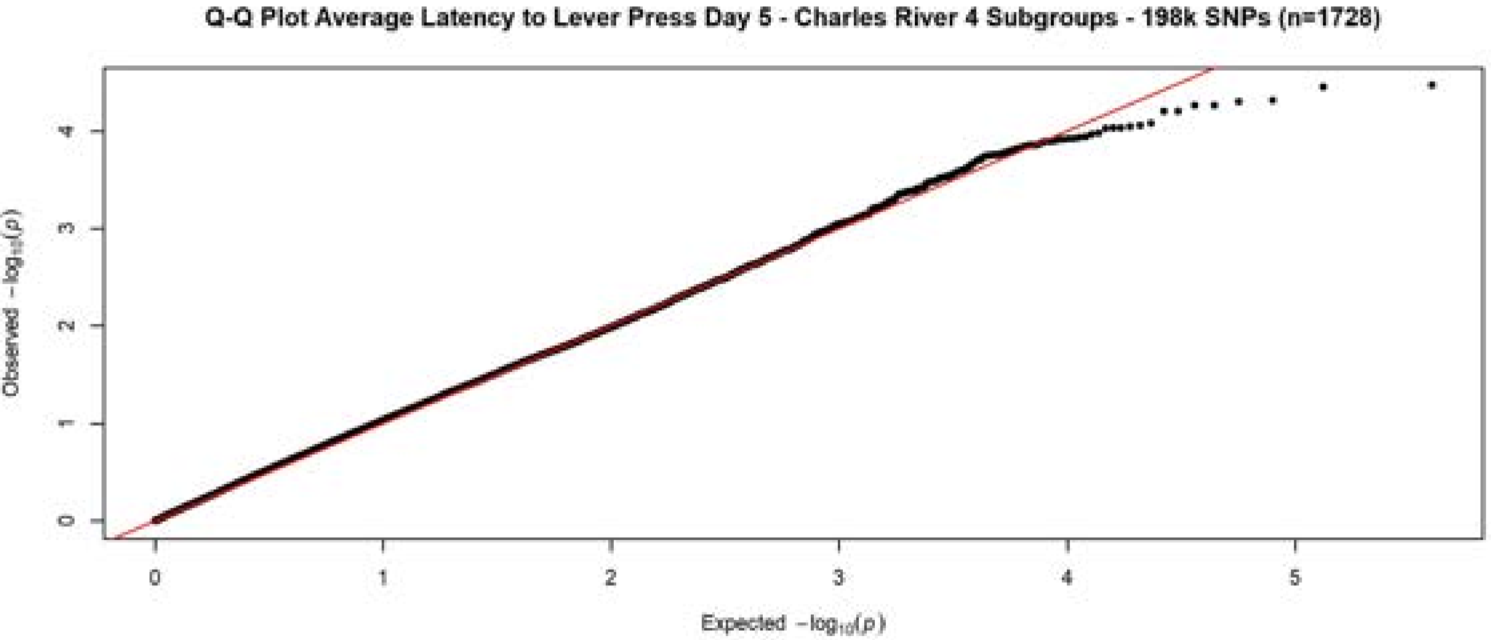
Q-Q Plot Average Latency to Lever Press Day 5 - Charles River 4 Subgroups - 198k SNPs (n=1728)
Observed −log10(p)
Expected −log10(p)

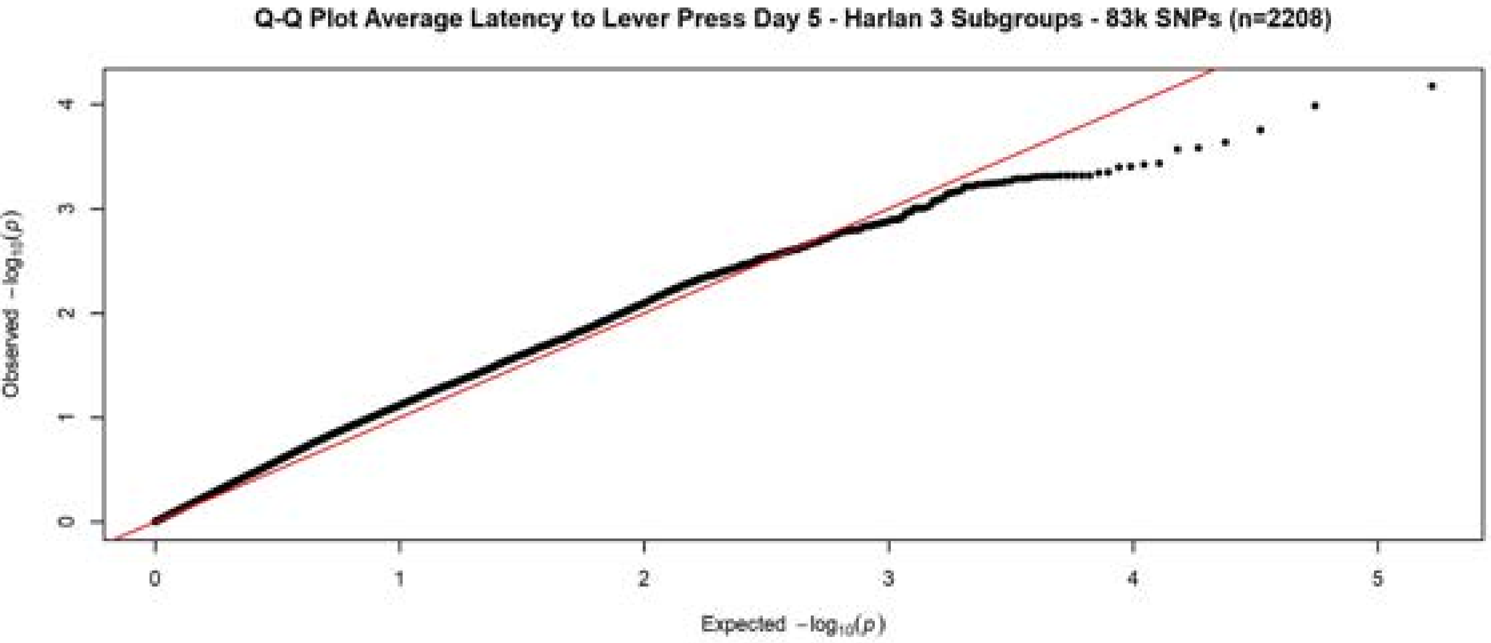
Q-Q Plot Average Latency to Lever Press Day 5 - Harlan 3 Subgroups - 83k SNPs (n=2208)
Observed −log10(p)
Expected −log10(p)

Q-Q Plot Average Latency to Magazine Entry Day 1 - Meta-analysis of 7 Subgroups - 64k SNPs (n=3903)

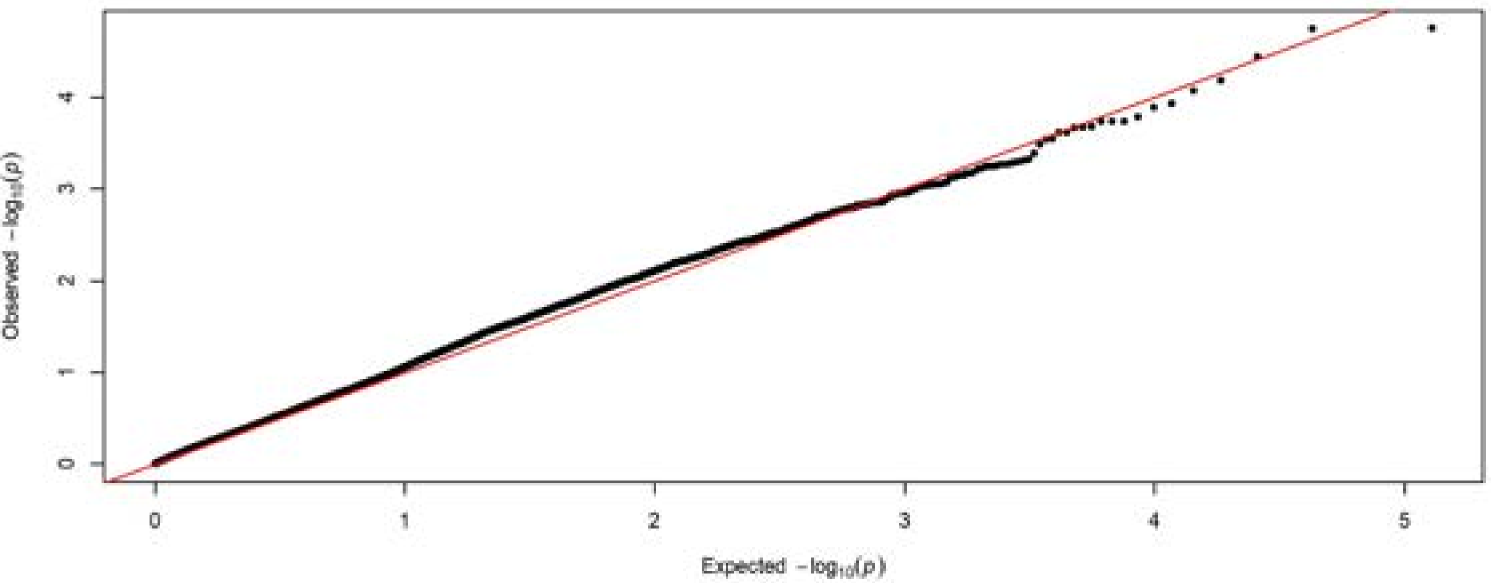

Q-Q Plot Average Latency to Magazine Entry Day 1 - Charles River 4 Subgroups - 198k SNPs (n=1728)

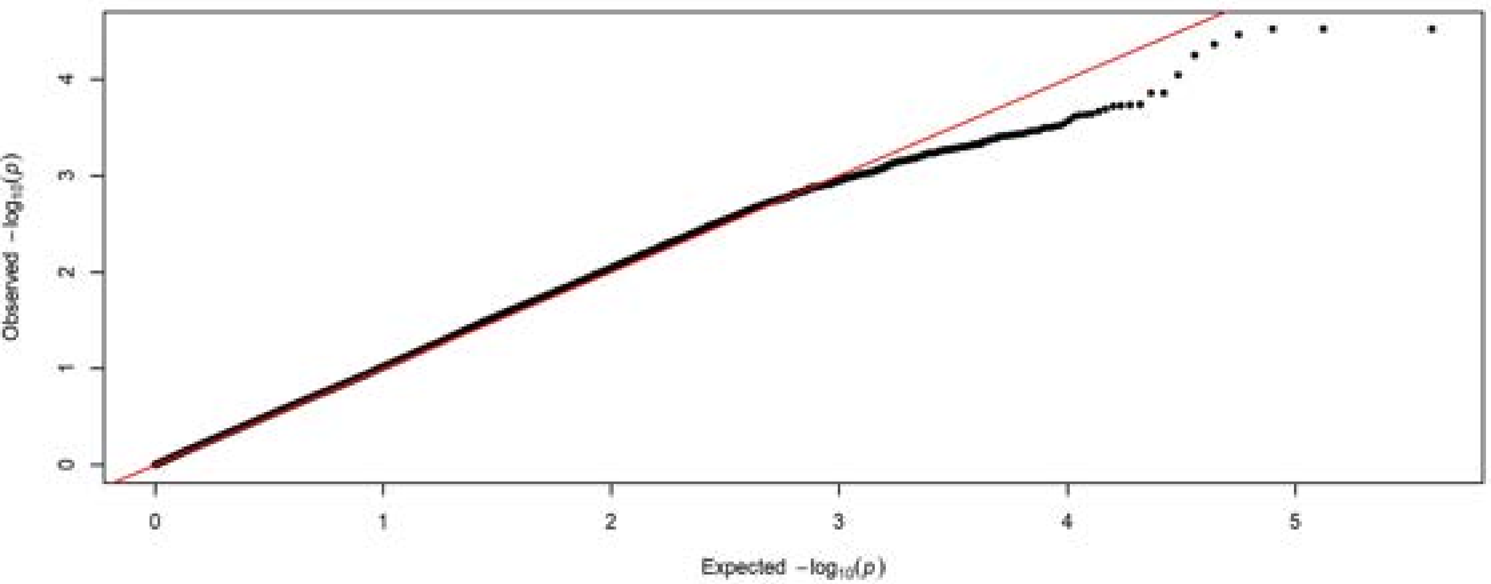

Q-Q Plot Average Latency to Magazine Entry Day 1 - Harlan 3 Subgroups - 83k SNPs (n=2175)

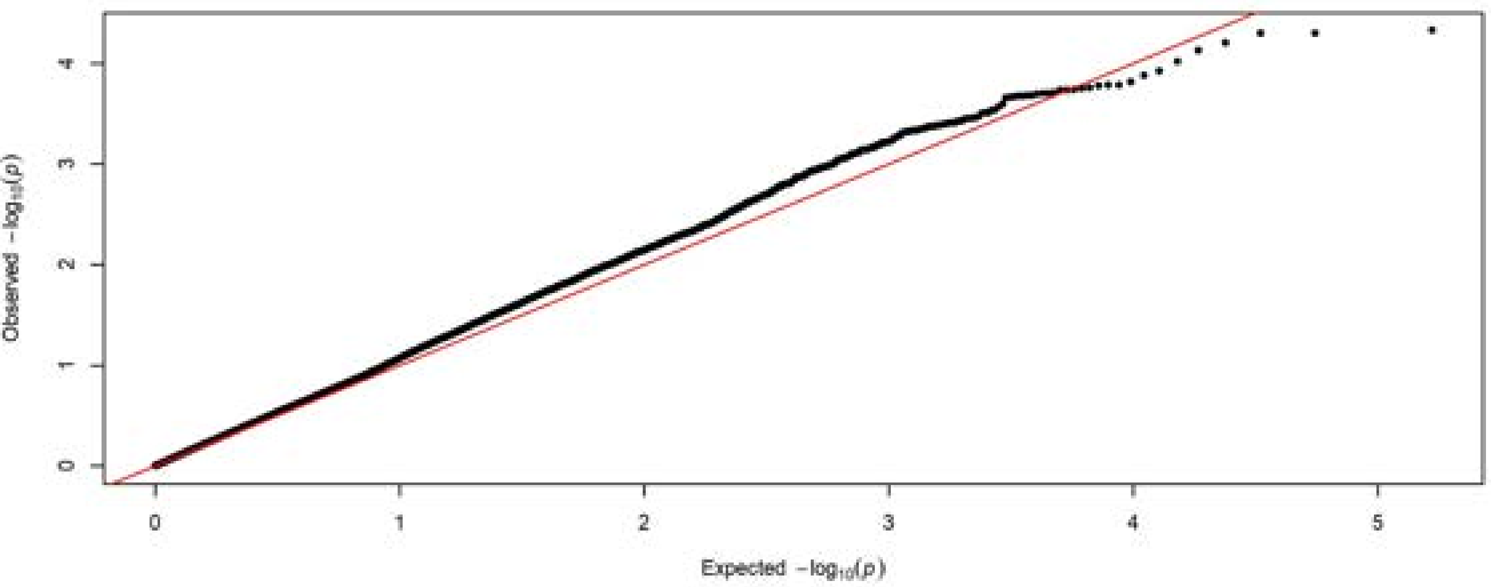

Q-Q Plot Average Latency to Magazine Entry Day 2 - Meta-analysis of 7 Subgroups - 64k SNPs (n=3934)

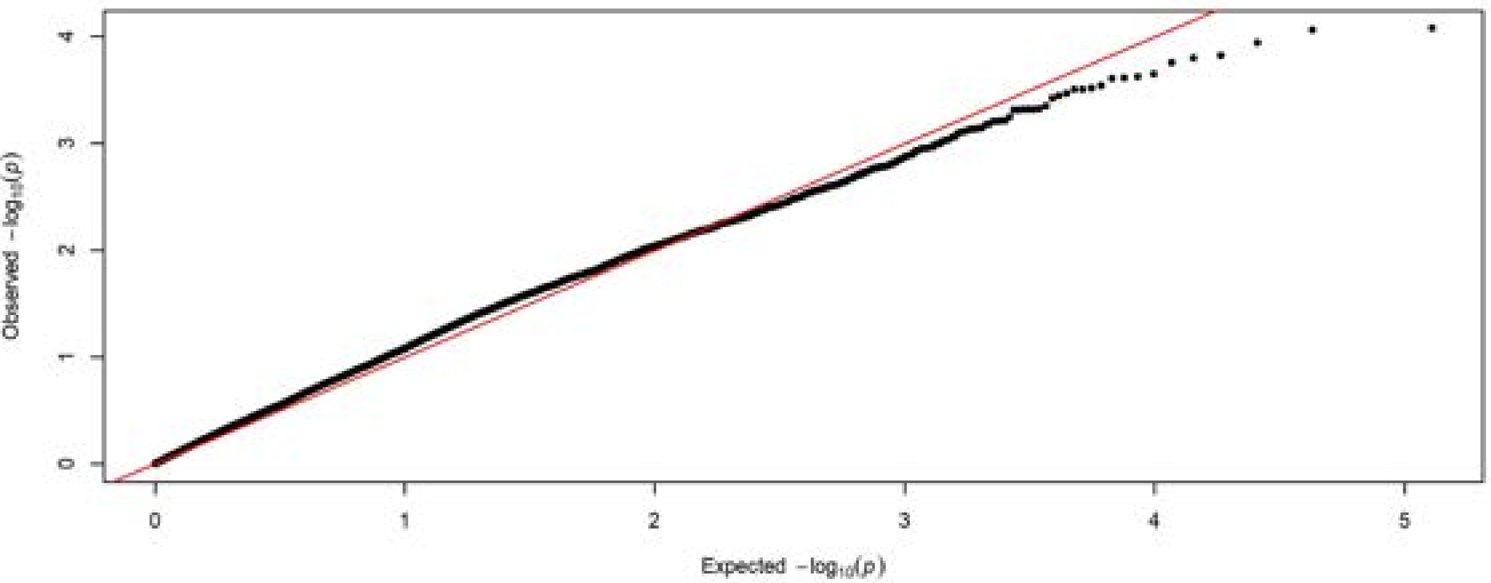

Q-Q Plot Average Latency to Magazine Entry Day 2 - Charles River 4 Subgroups - 198k SNPs (n=1726)

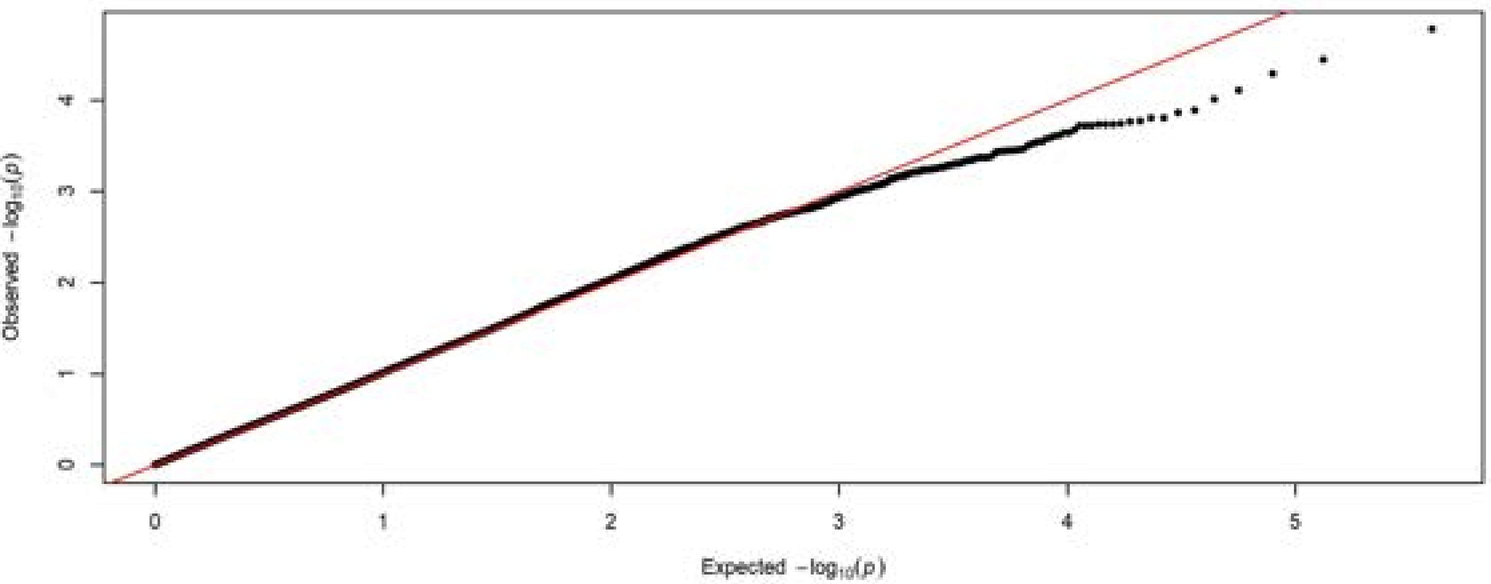

Q-Q Plot Average Latency to Magazine Entry Day 2 - Harlan 3 Subgroups - 83k SNPs (n=2208)

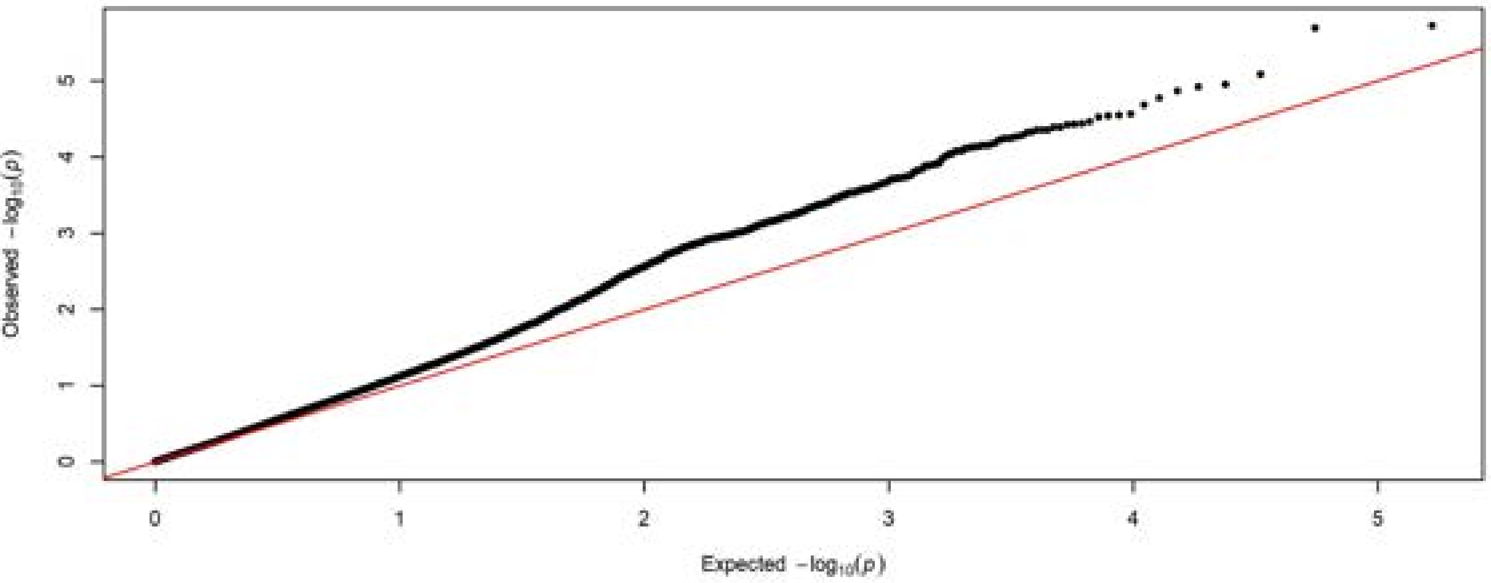

Q-Q Plot Average Latency to Magazine Entry Day 3 - Meta-analysis of 7 Subgroups - 64k SNPs (n=3932)

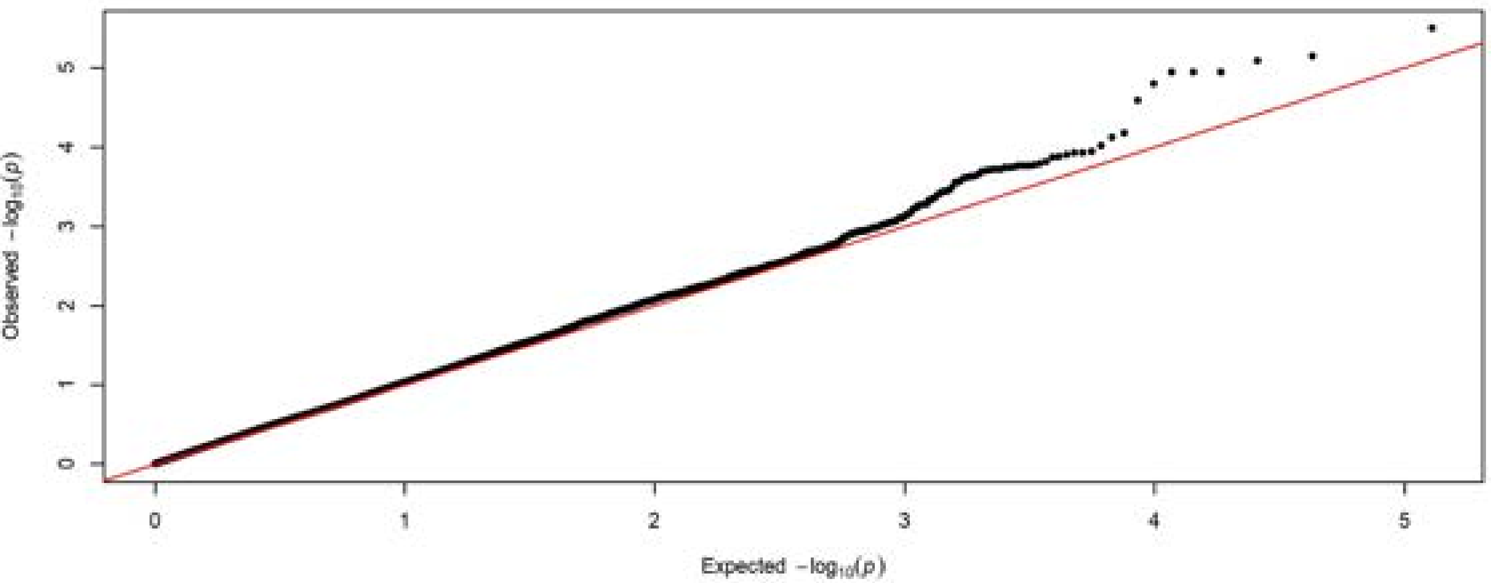

Q-Q Plot Average Latency to Magazine Entry Day 3 - Charles River 4 Subgroups - 198k SNPs (n=1727)

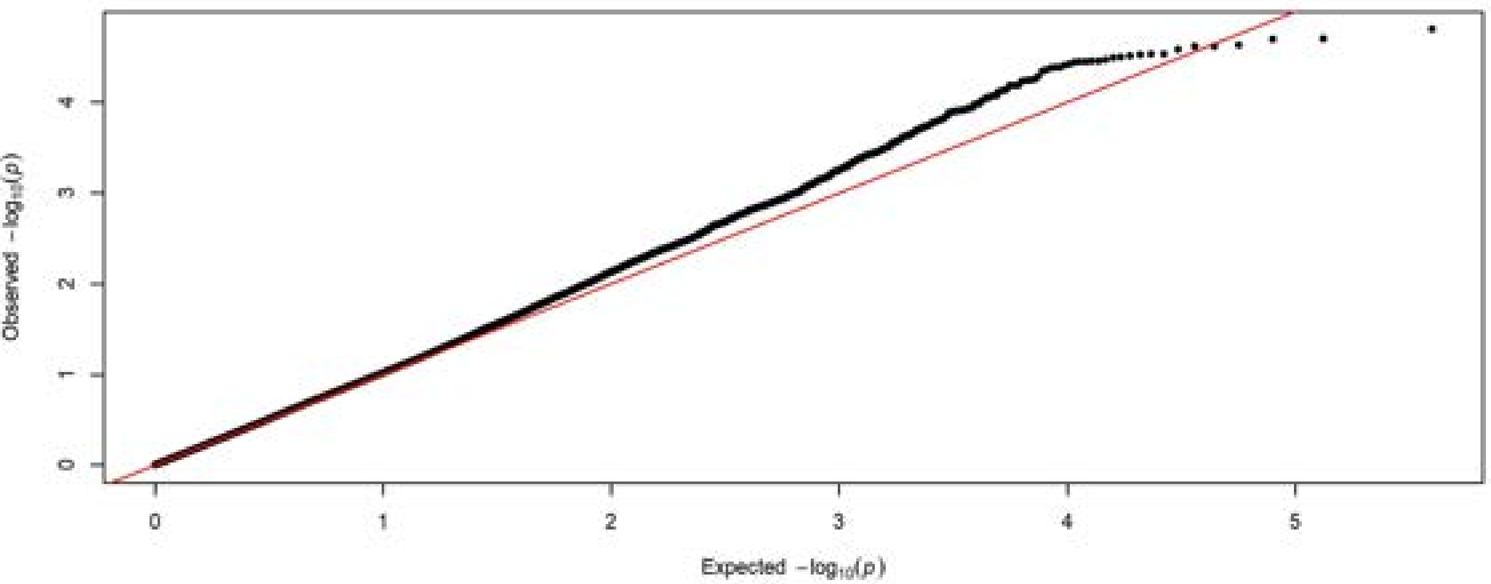

Q-Q Plot Average Latency to Magazine Entry Day 3 - Harlan 3 Subgroups - 83k SNPs (n=2205)

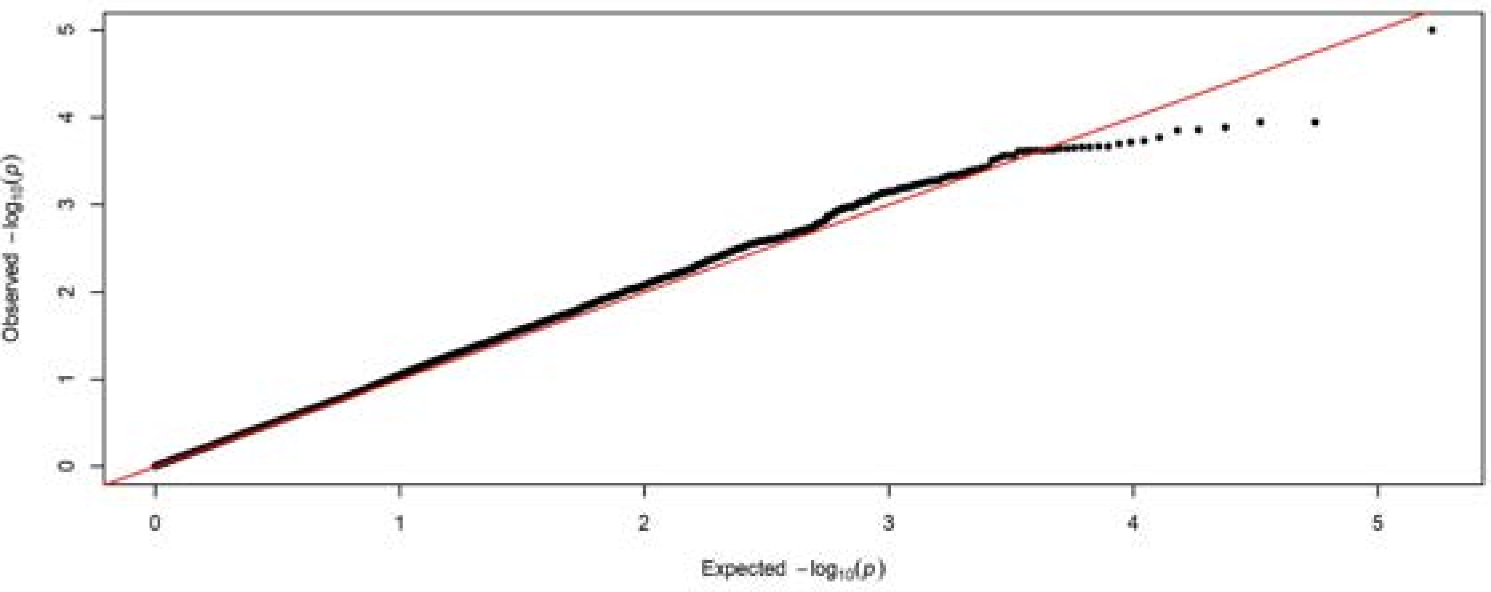

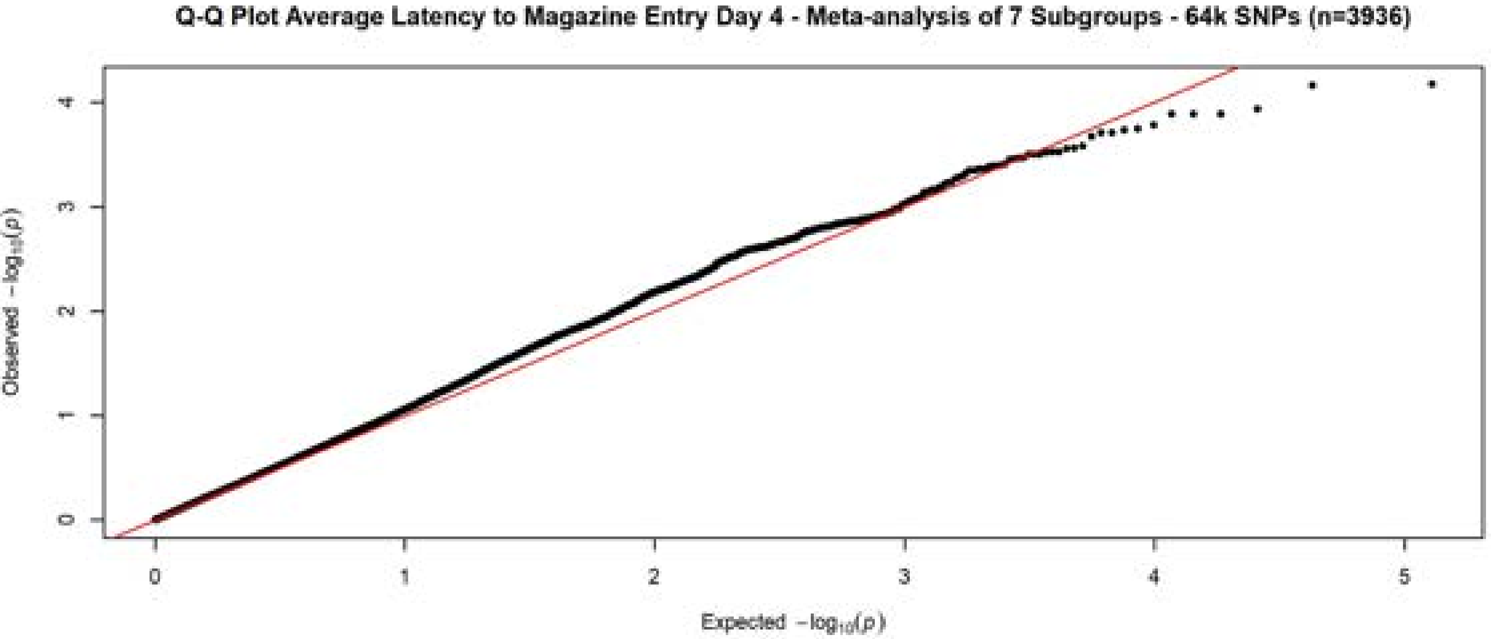
Q-Q Plot Average Latency to Magazine Entry Day 4 - Meta-analysis of 7 Subgroups - 64k SNPs (n=3936)
Observed −log10(p)
Expected −log10(p)

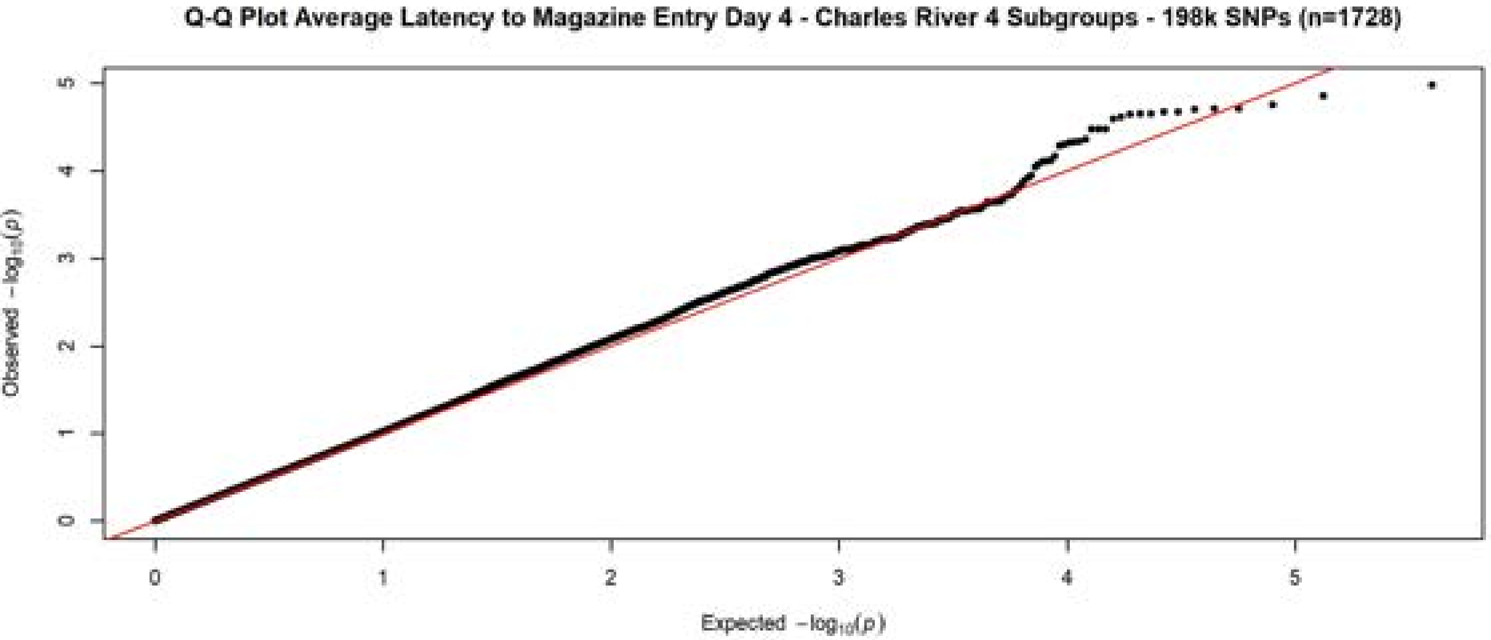
Q-Q Plot Average Latency to Magazine Entry Day 4 - Charles River 4 Subgroups - 198k SNPs (n=1728)
Observed −log10(p)
Expected −log10(p)

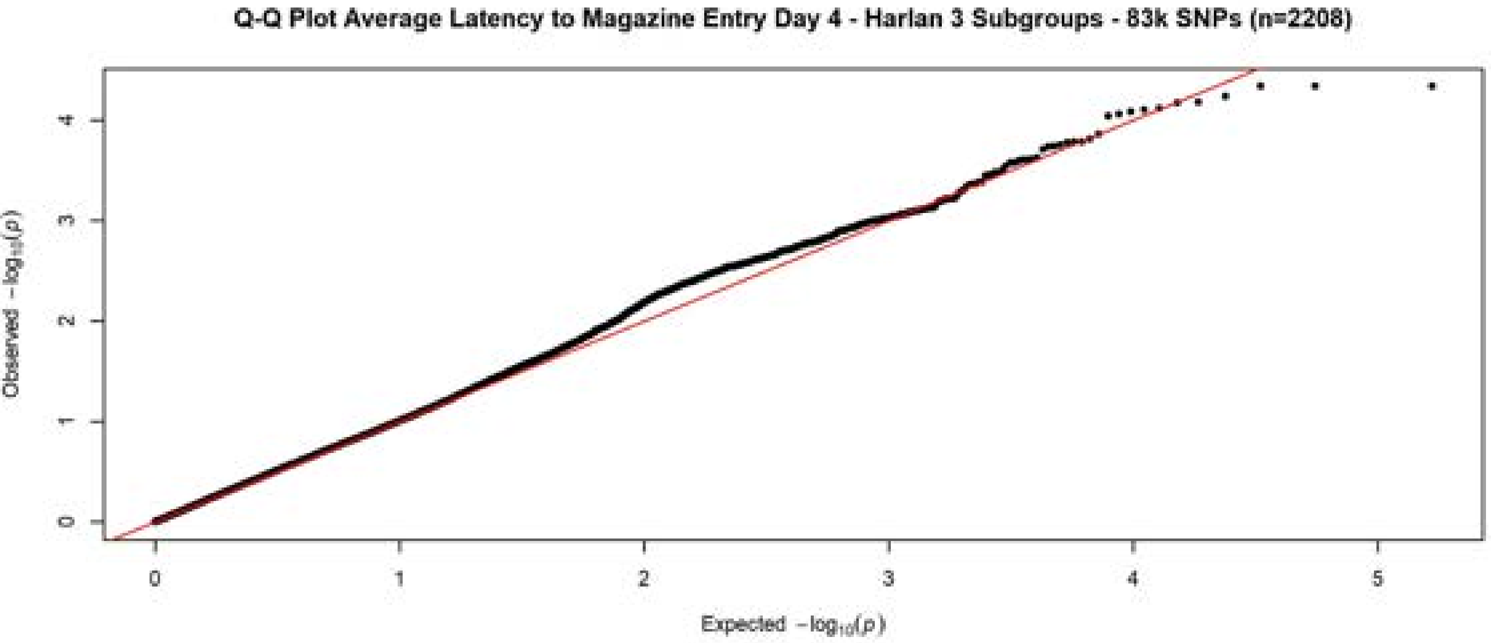
Q-Q Plot Average Latency to Magazine Entry Day 4 - Harlan 3 Subgroups - 83k SNPs (n=2208)
Observed −log10(p)
Expected −log10(p)

Q-Q Plot Average Latency to Magazine Entry Day 5 - Meta-analysis of 7 Subgroups - 64k SNPs (n=3936)

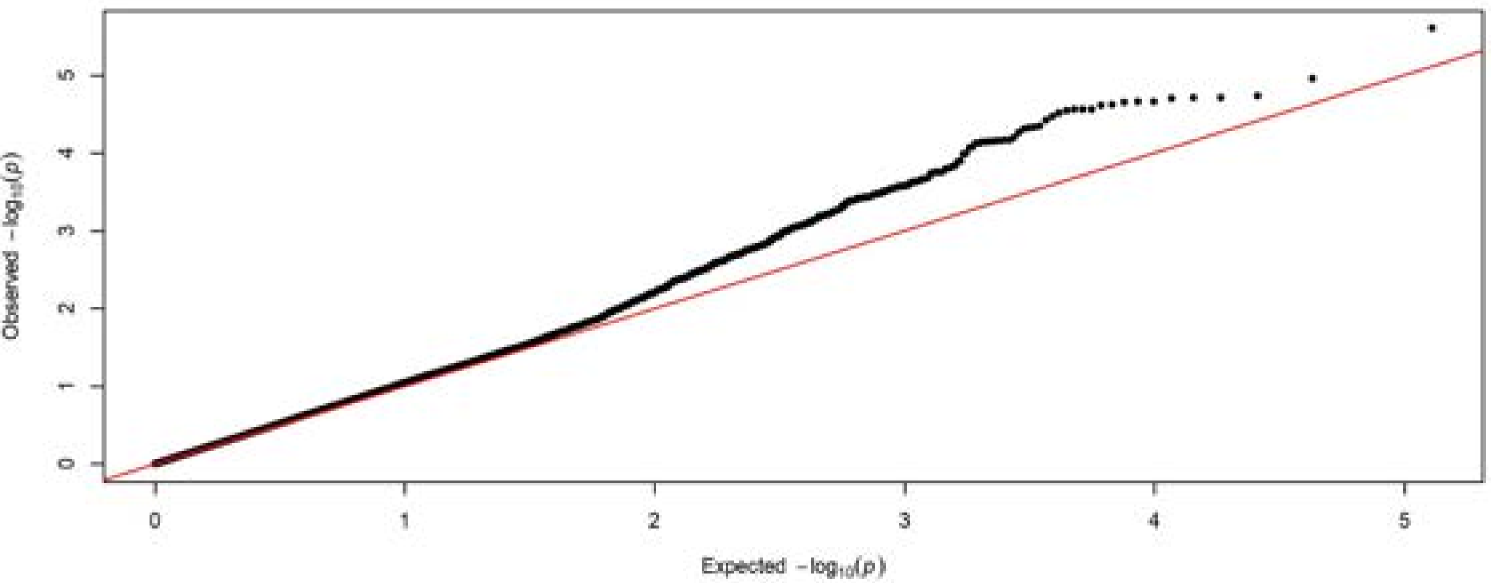

Q-Q Plot Average Latency to Magazine Entry Day 5 - Charles River 4 Subgroups - 198k SNPs (n=1728)

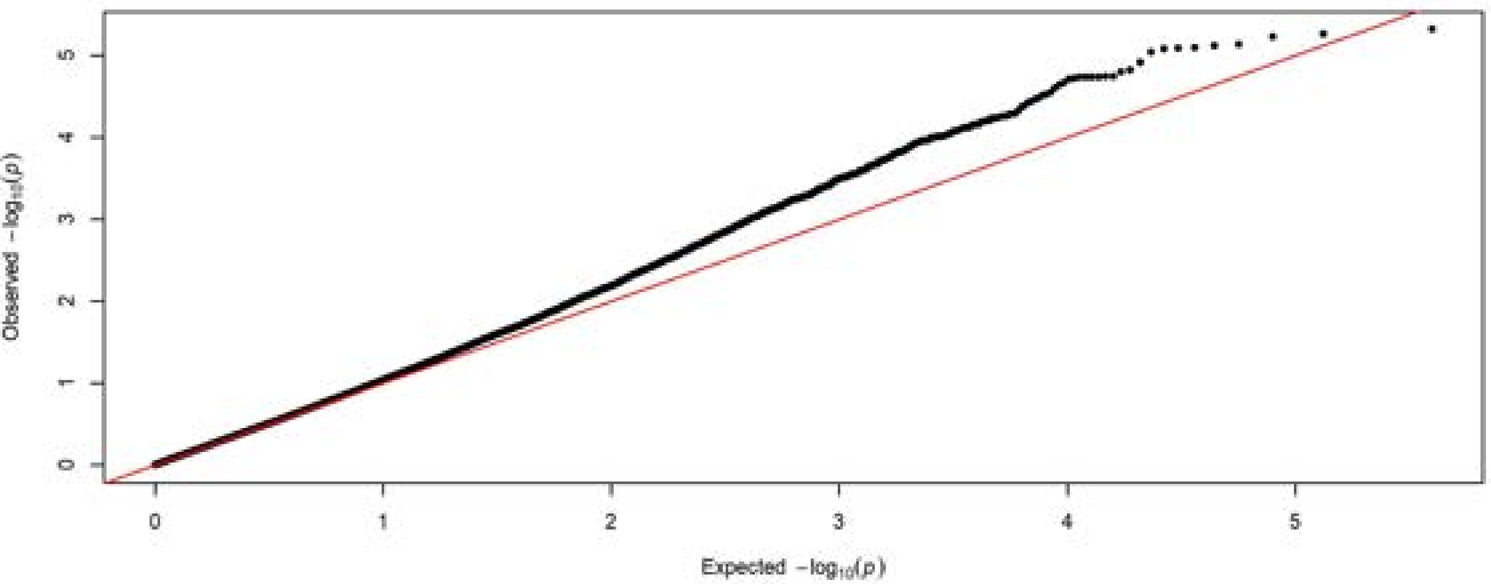

Q-Q Plot Average Latency to Magazine Entry Day 5 - Harlan 3 Subgroups - 83k SNPs (n=2208)

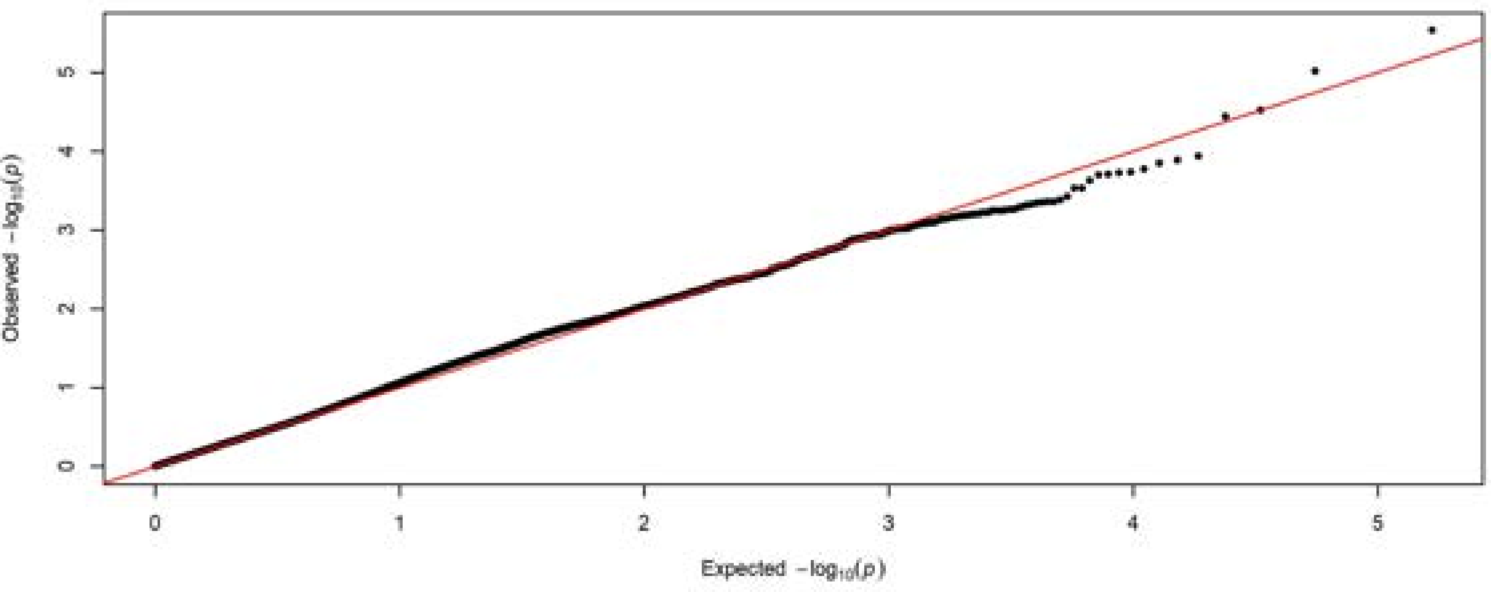

Q-Q Plot PavCA Index Score Day 1 - Meta-analysis of 7 Subgroups - 64k SNPs (n=3880)

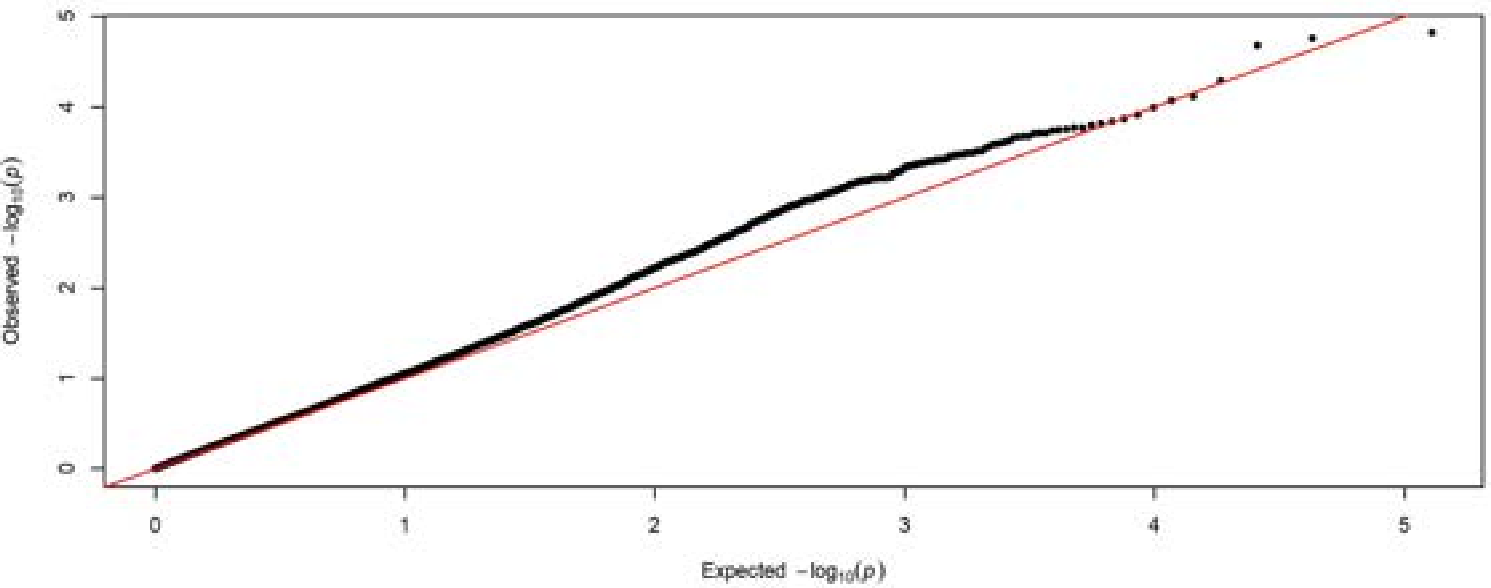

Q-Q Plot PavCA Index Score Day 1 - Charles River 4 Subgroups - 198k SNPs (n=1720)

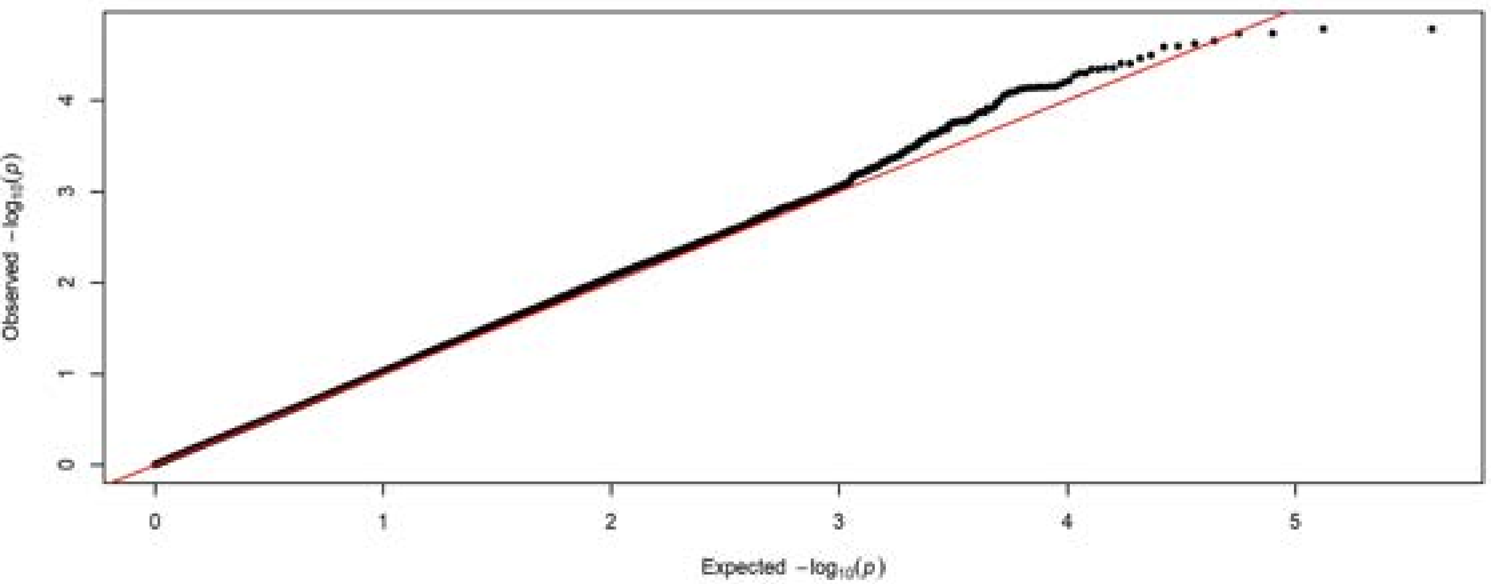

Q-Q Plot PavCA Index Score Day 1 - Harlan 3 Subgroups - 83k SNPs (n=2160)

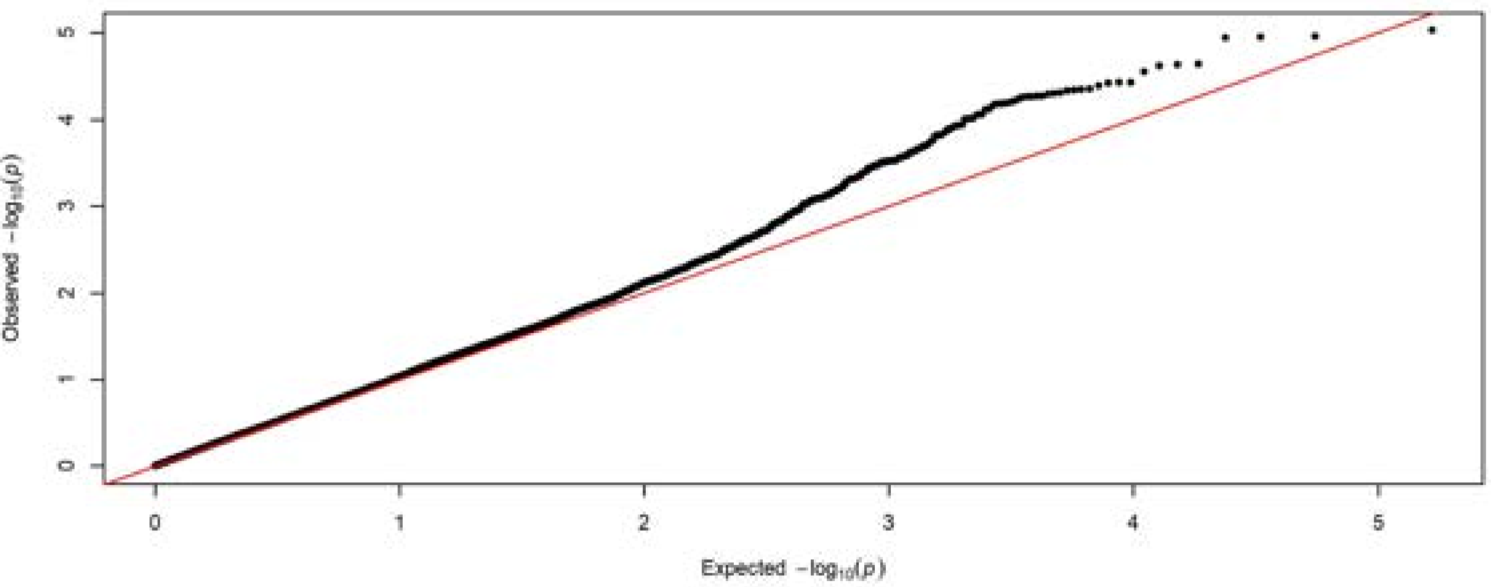

Q-Q Plot PavCA Index Score Day 2 - Meta-analysis of 7 Subgroups - 64k SNPs (n=3919)

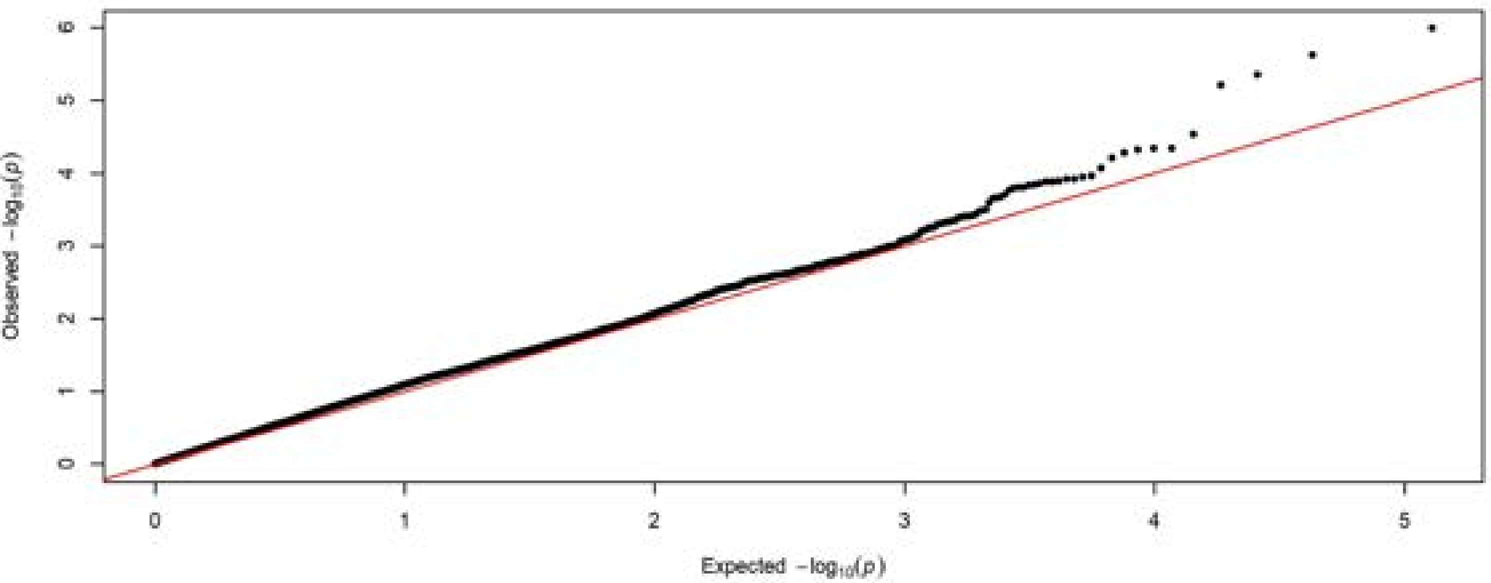

Q-Q Plot PavCA Index Score Day 2 - Charles River 4 Subgroups - 198k SNPs (n=1719)

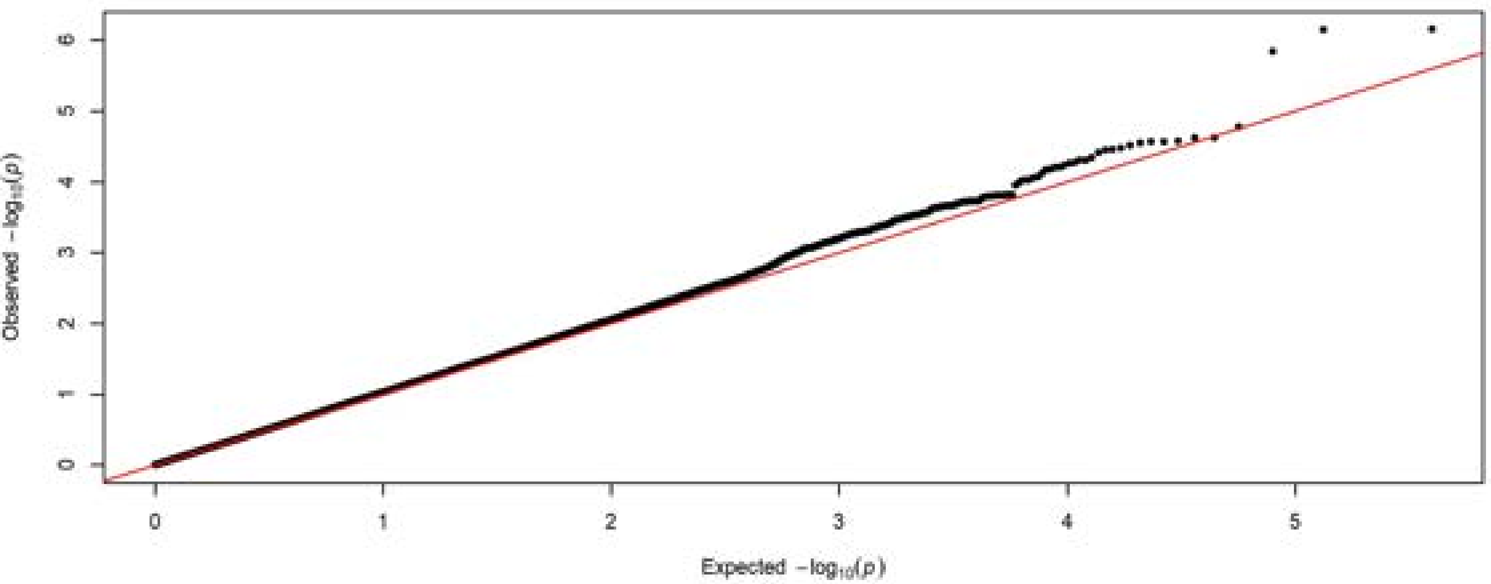

Q-Q Plot PavCA Index Score Day 2 - Harlan 3 Subgroups - 83k SNPs (n=2200)

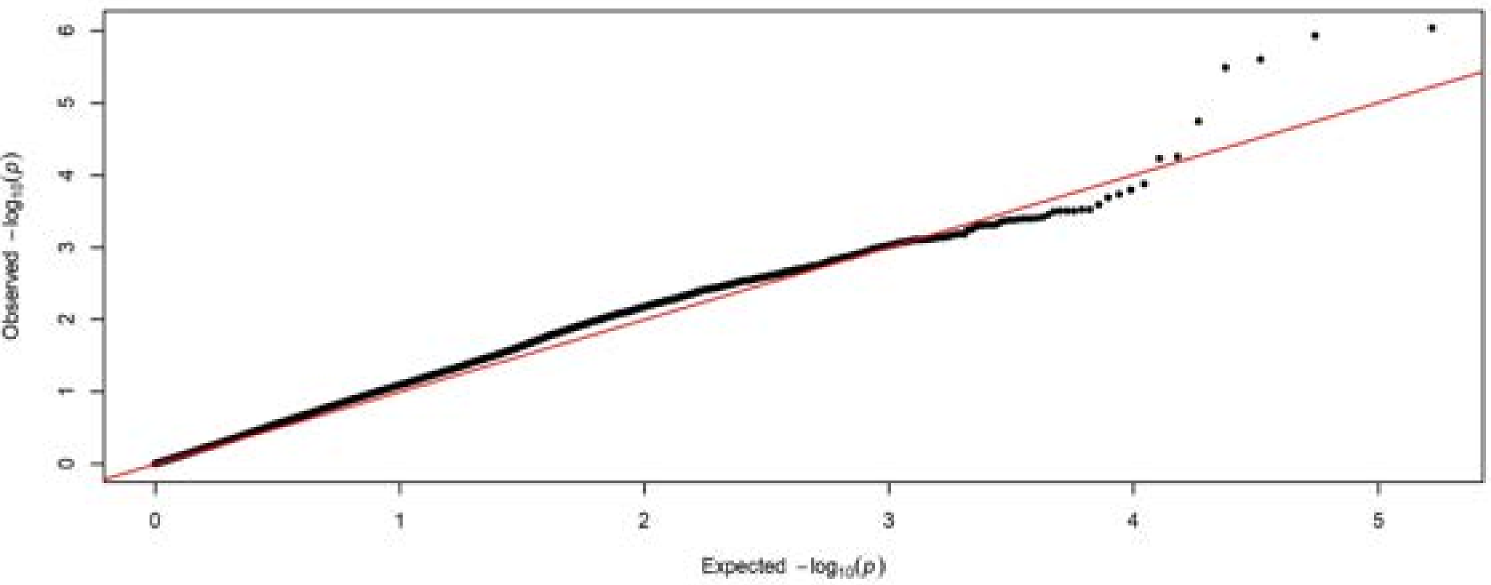

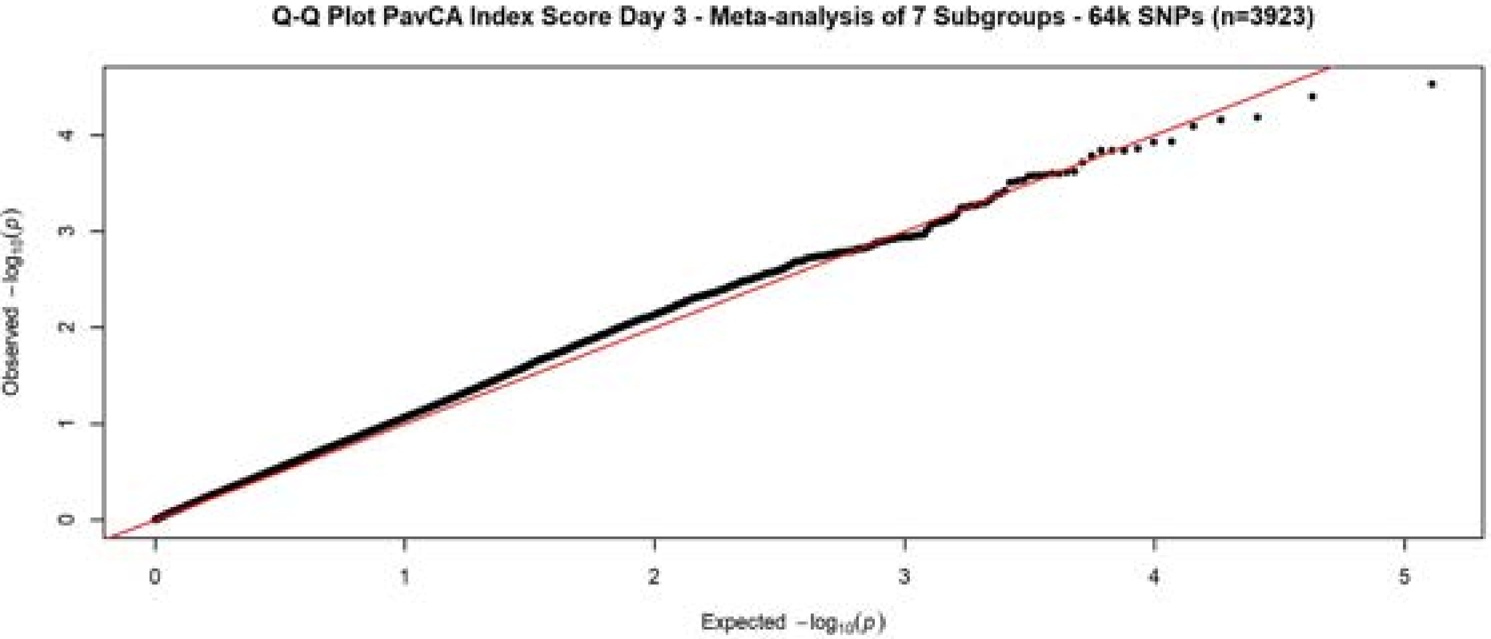
Q-Q Plot PavCA Index Score Day 3 - Meta-analysis of 7 Subgroups - 64k SNPs (n=3923)
Observed $-\log_{10}(p)$
Expected $-\log_{10}(p)$

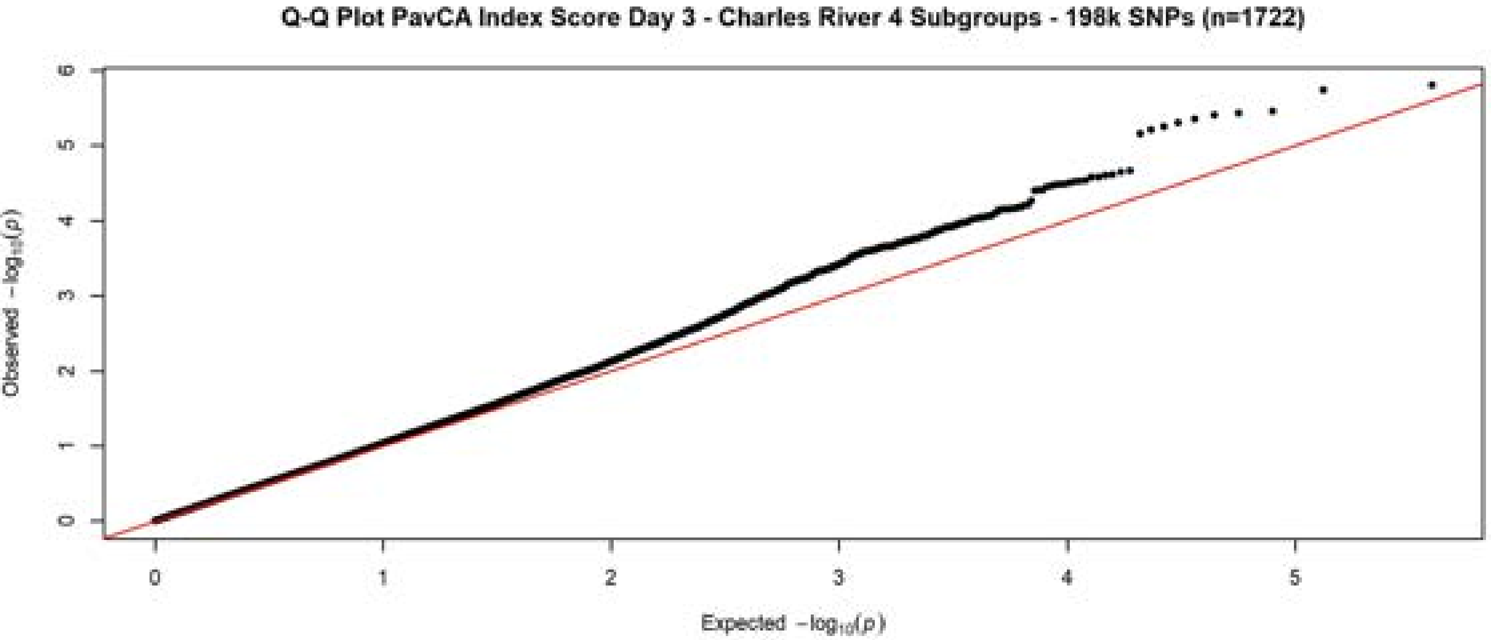
Q-Q Plot PavCA Index Score Day 3 - Charles River 4 Subgroups - 198k SNPs (n=1722)
Observed $-\log_{10}(p)$
Expected $-\log_{10}(p)$

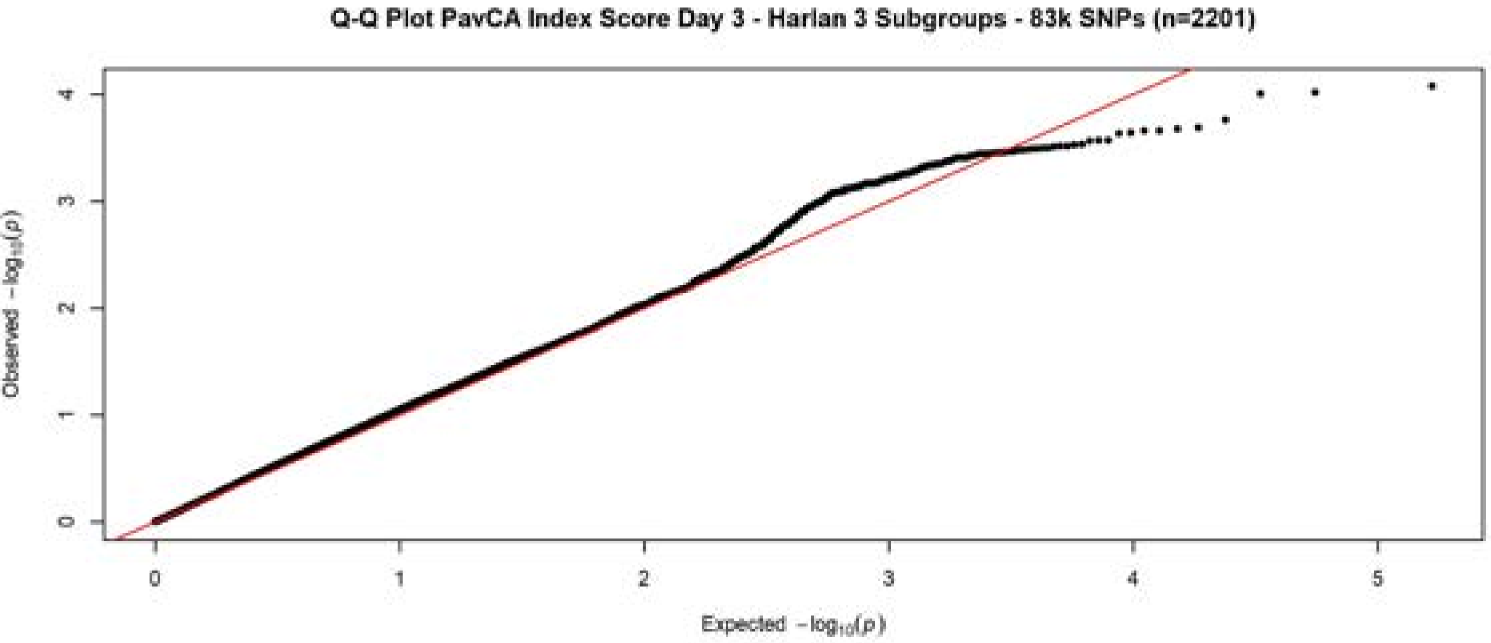
Q-Q Plot PavCA Index Score Day 3 - Harlan 3 Subgroups - 83k SNPs (n=2201)
Observed $-\log_{10}(p)$
Expected $-\log_{10}(p)$

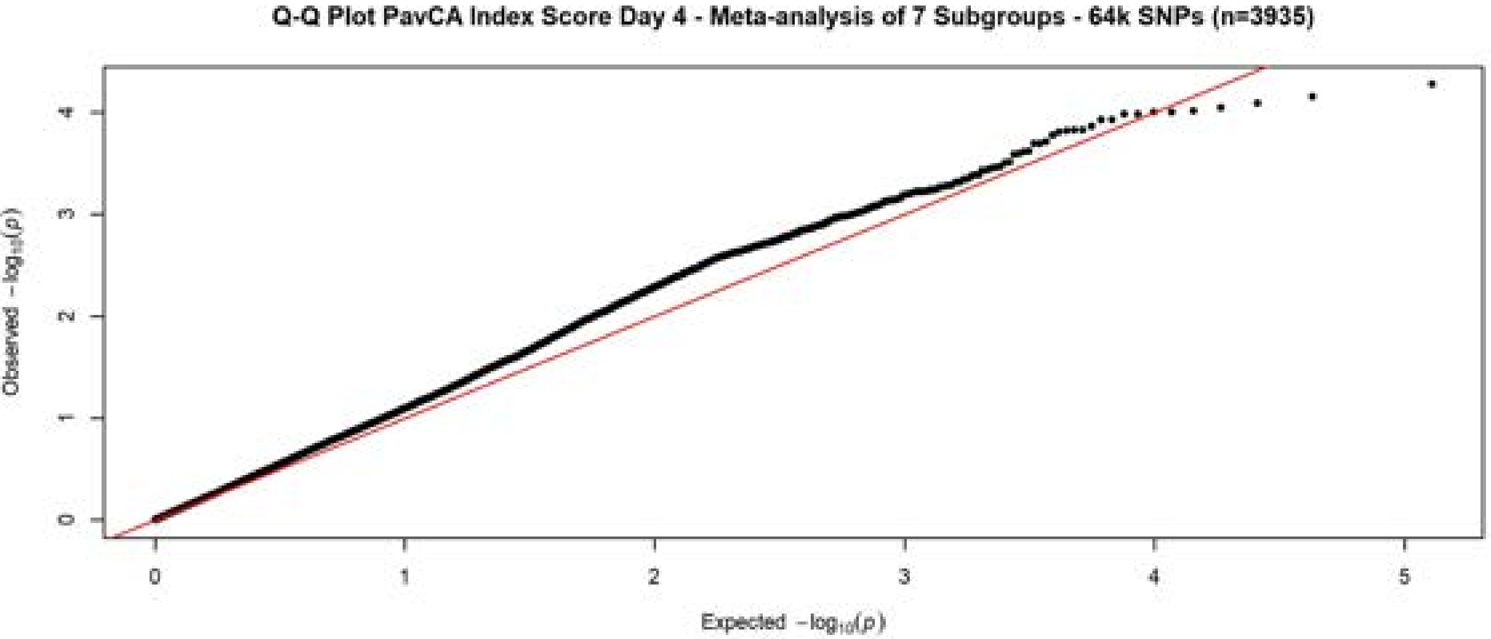
Q-Q Plot PavCA Index Score Day 4 - Meta-analysis of 7 Subgroups - 64k SNPs (n=3935)
Observed −log10(p)
Expected −log10(p)

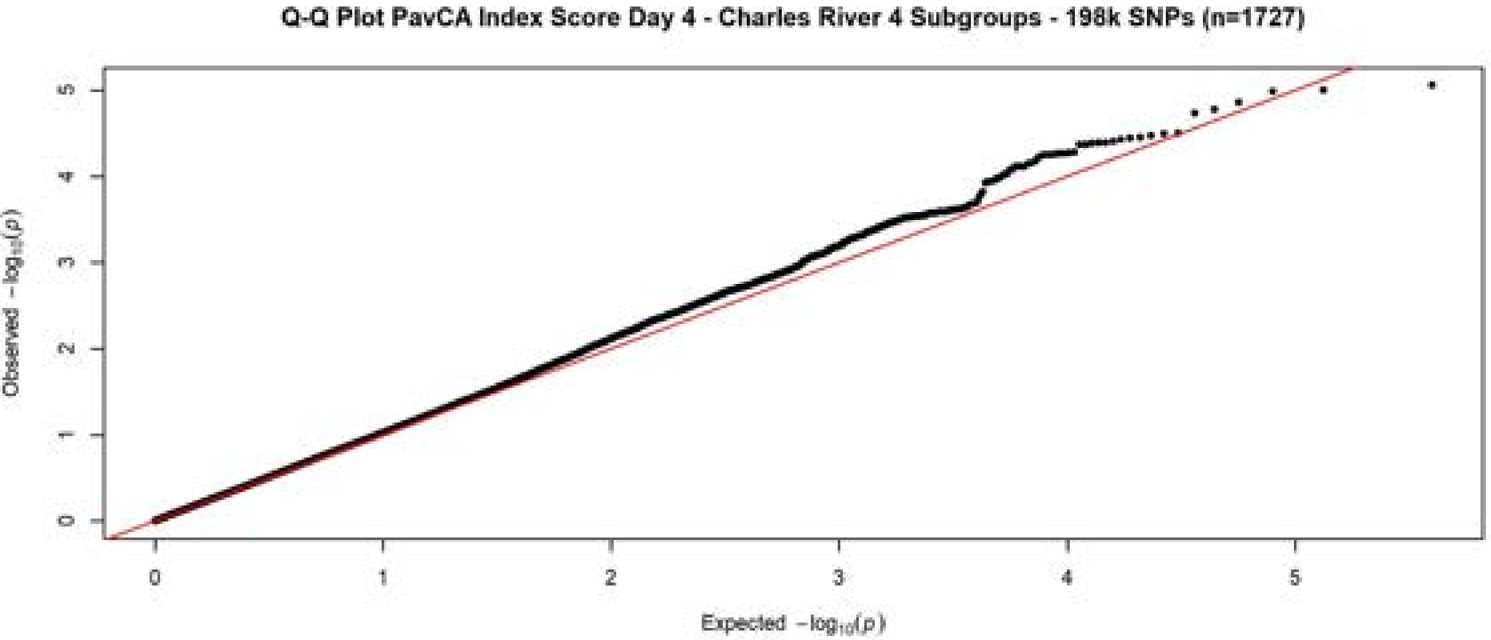
Q-Q Plot PavCA Index Score Day 4 - Charles River 4 Subgroups - 198k SNPs (n=1727)
Observed −log10(p)
Expected −log10(p)

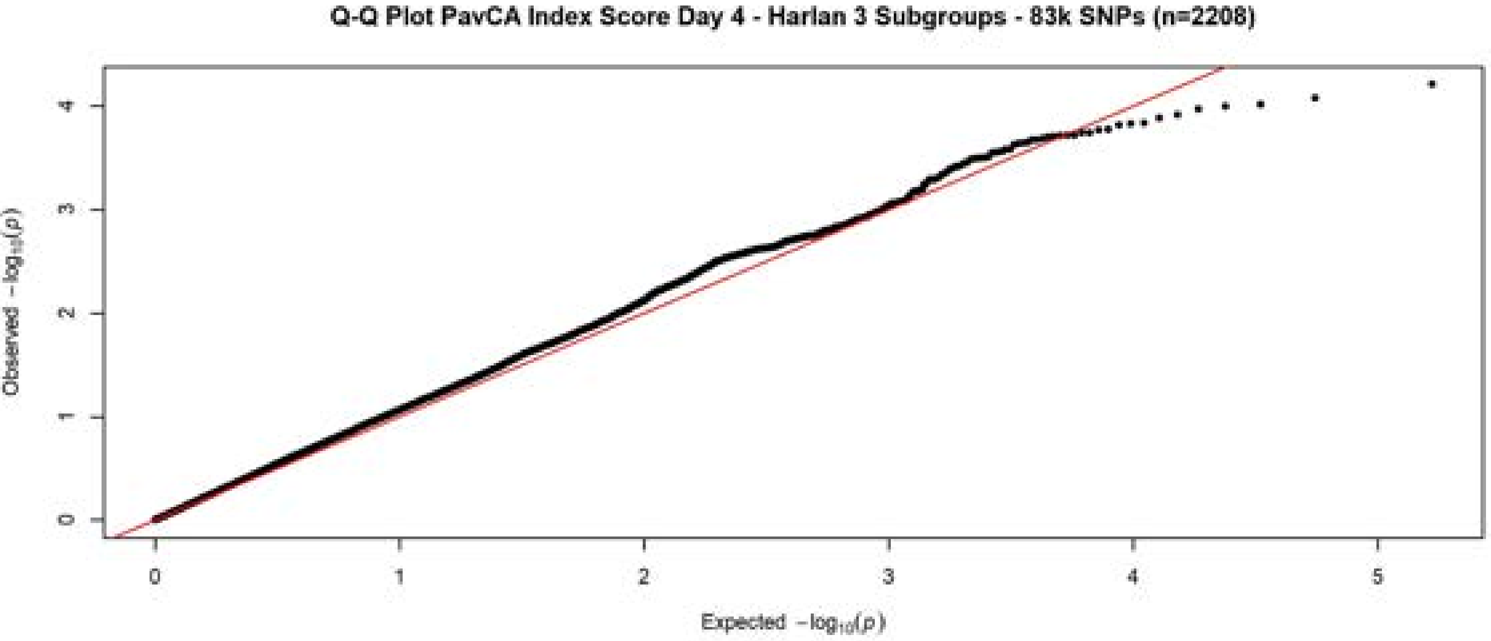
Q-Q Plot PavCA Index Score Day 4 - Harlan 3 Subgroups - 83k SNPs (n=2208)
Observed −log10(p)
Expected −log10(p)

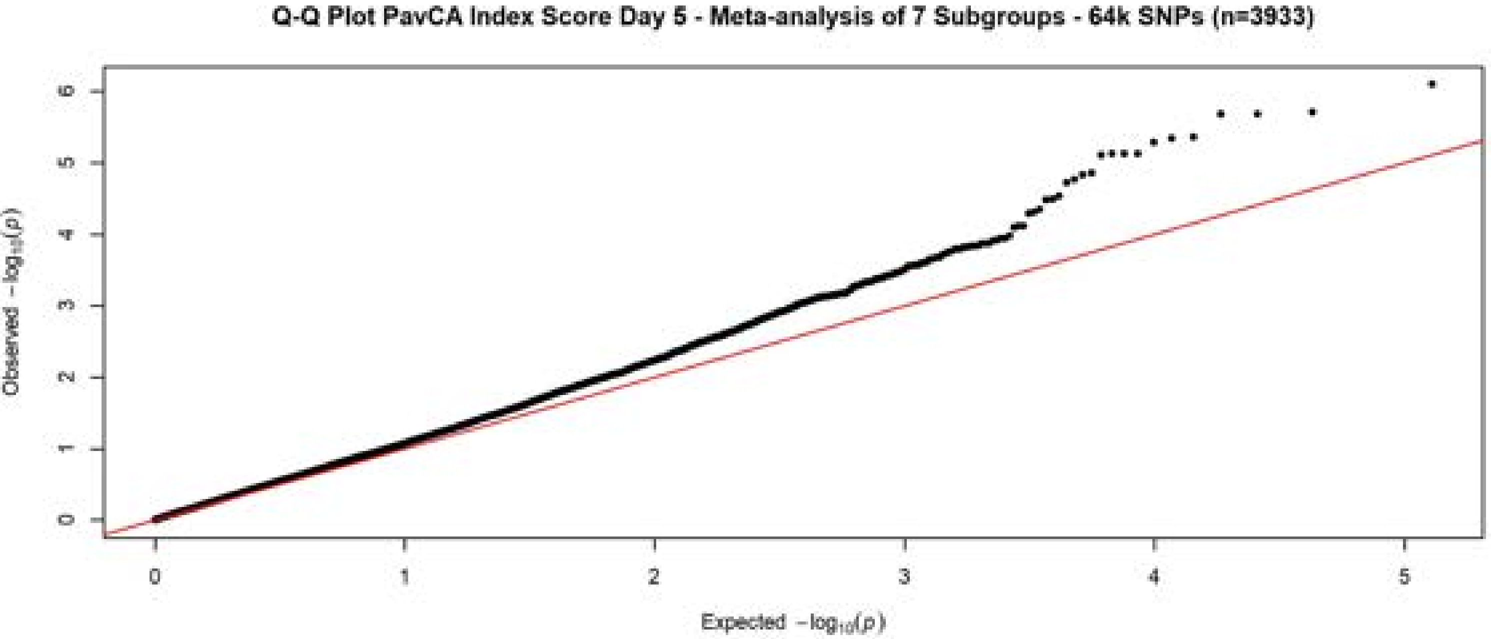
Q-Q Plot PavCA Index Score Day 5 - Meta-analysis of 7 Subgroups - 64k SNPs (n=3933)
Observed $-\log_{10}(p)$
Expected $-\log_{10}(p)$

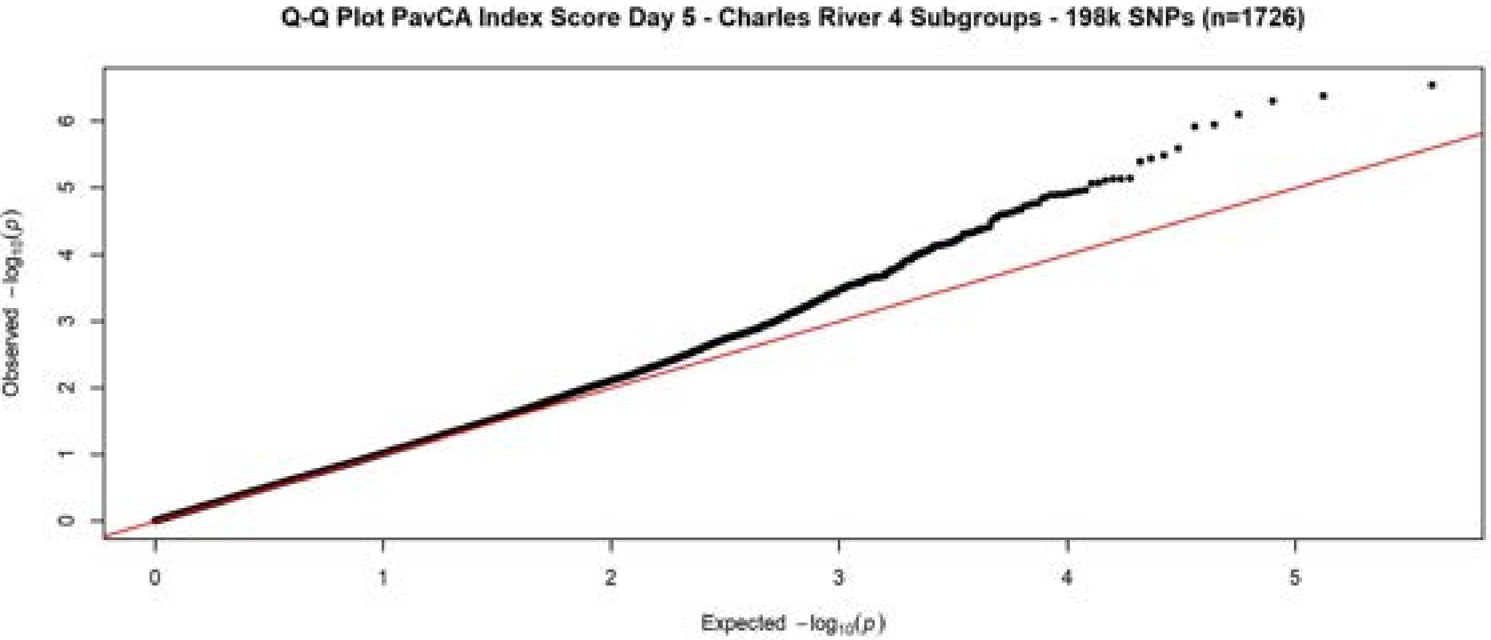
Q-Q Plot PavCA Index Score Day 5 - Charles River 4 Subgroups - 198k SNPs (n=1726)
Observed $-\log_{10}(p)$
Expected $-\log_{10}(p)$

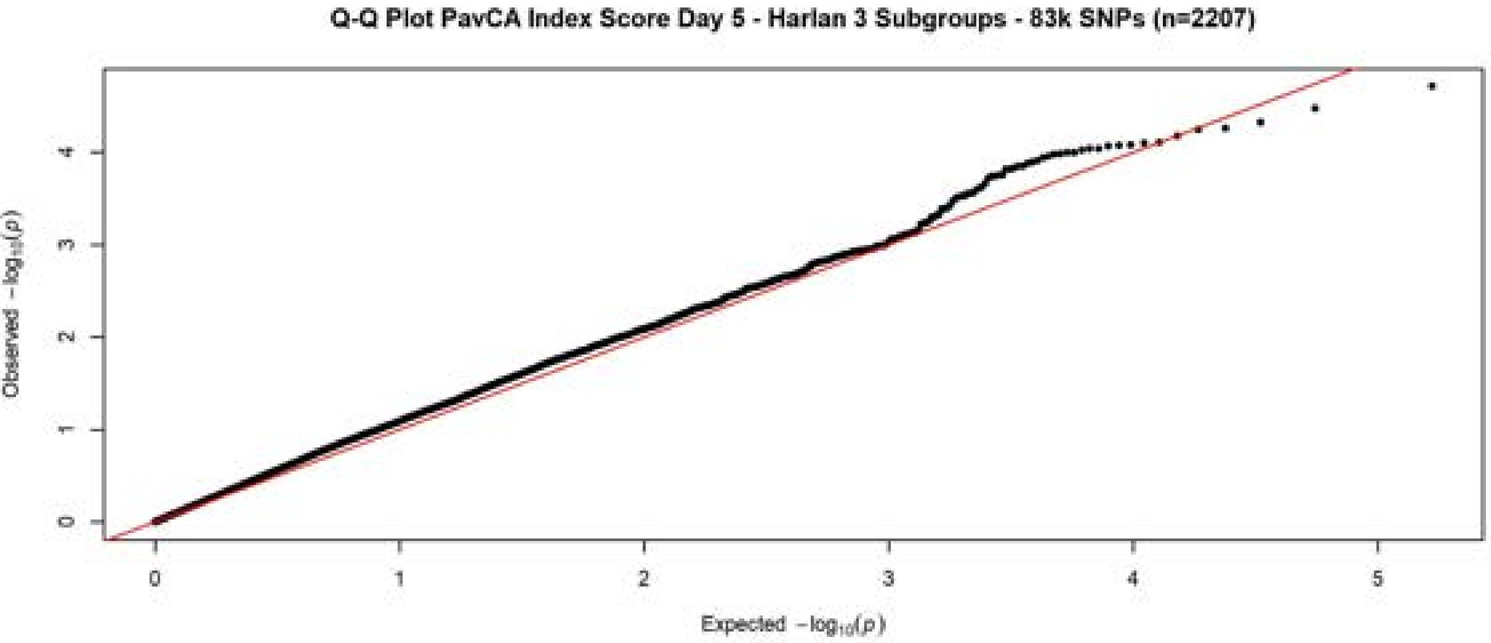
Q-Q Plot PavCA Index Score Day 5 - Harlan 3 Subgroups - 83k SNPs (n=2207)
Observed $-\log_{10}(p)$
Expected $-\log_{10}(p)$

Q-Q Plot Latency Score Day 1 - Meta-analysis of 7 Subgroups - 64k SNPs (n=3903)

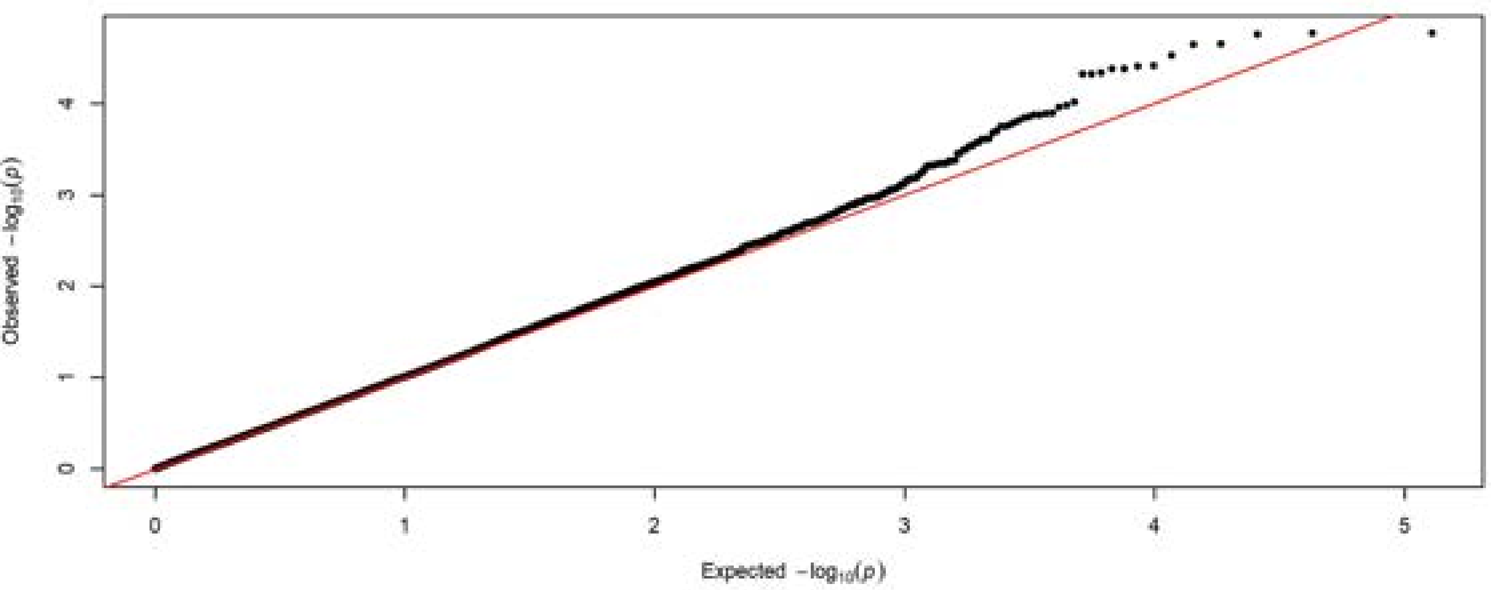

Q-Q Plot Latency Score Day 1 - Charles River 4 Subgroups - 198k SNPs (n=1728)

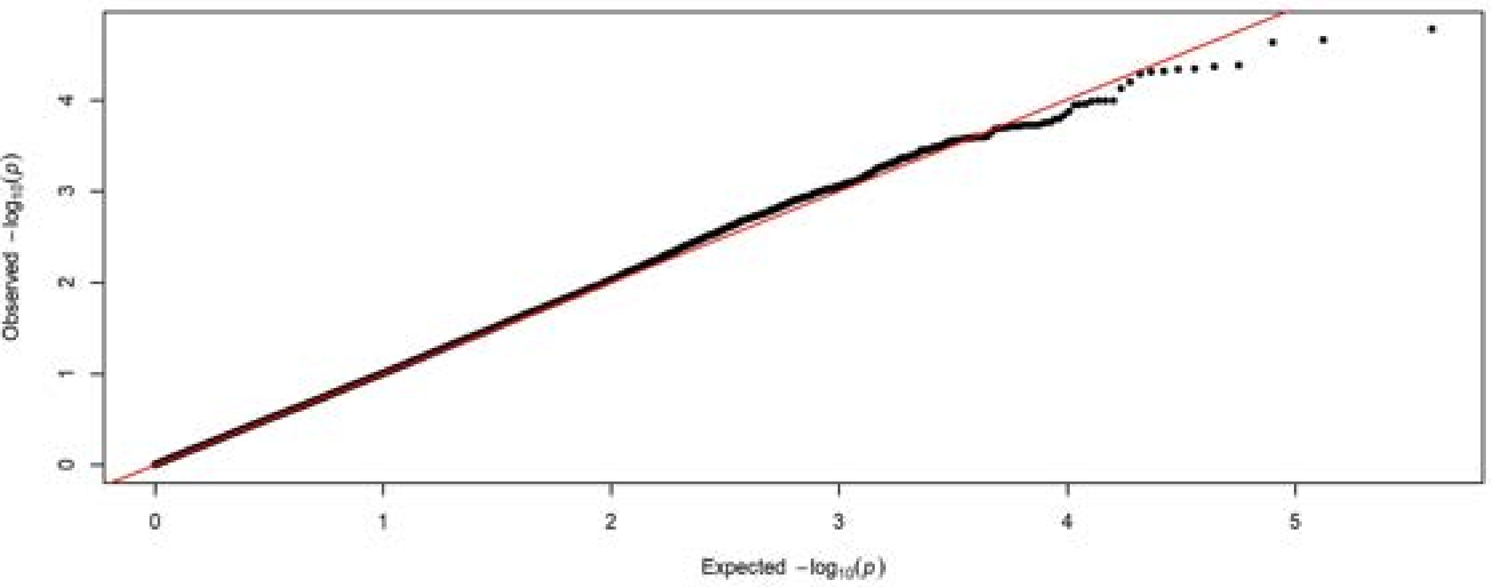

Q-Q Plot Latency Score Day 1 - Harlan 3 Subgroups - 83k SNPs (n=2175)

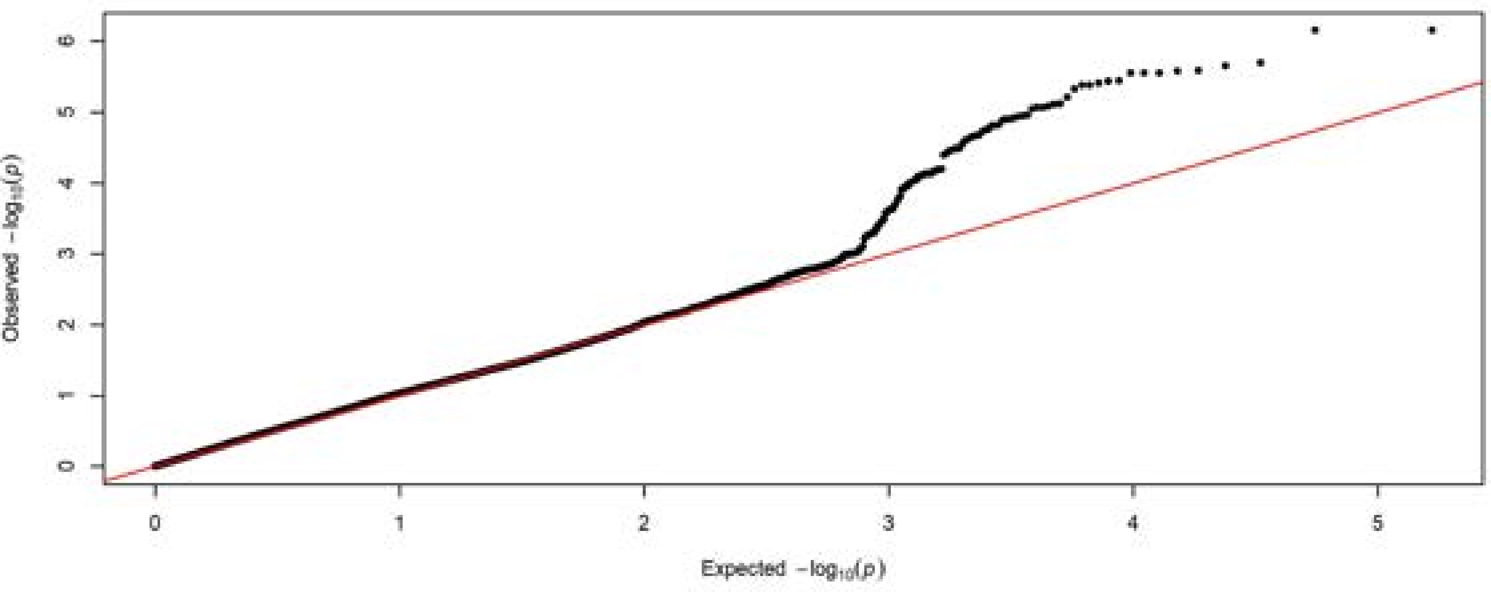

Q-Q Plot Latency Score Day 2 - Meta-analysis of 7 Subgroups - 64k SNPs (n=3934)

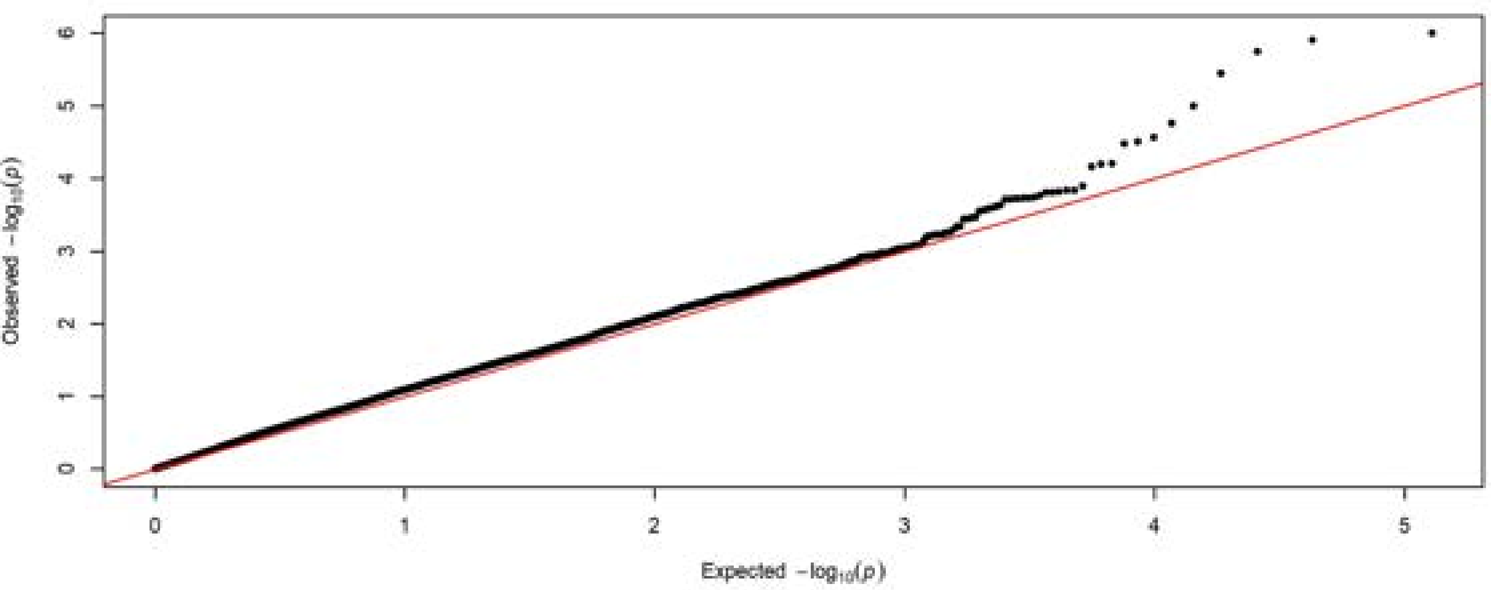

Q-Q Plot Latency Score Day 2 - Charles River 4 Subgroups - 198k SNPs (n=1726)

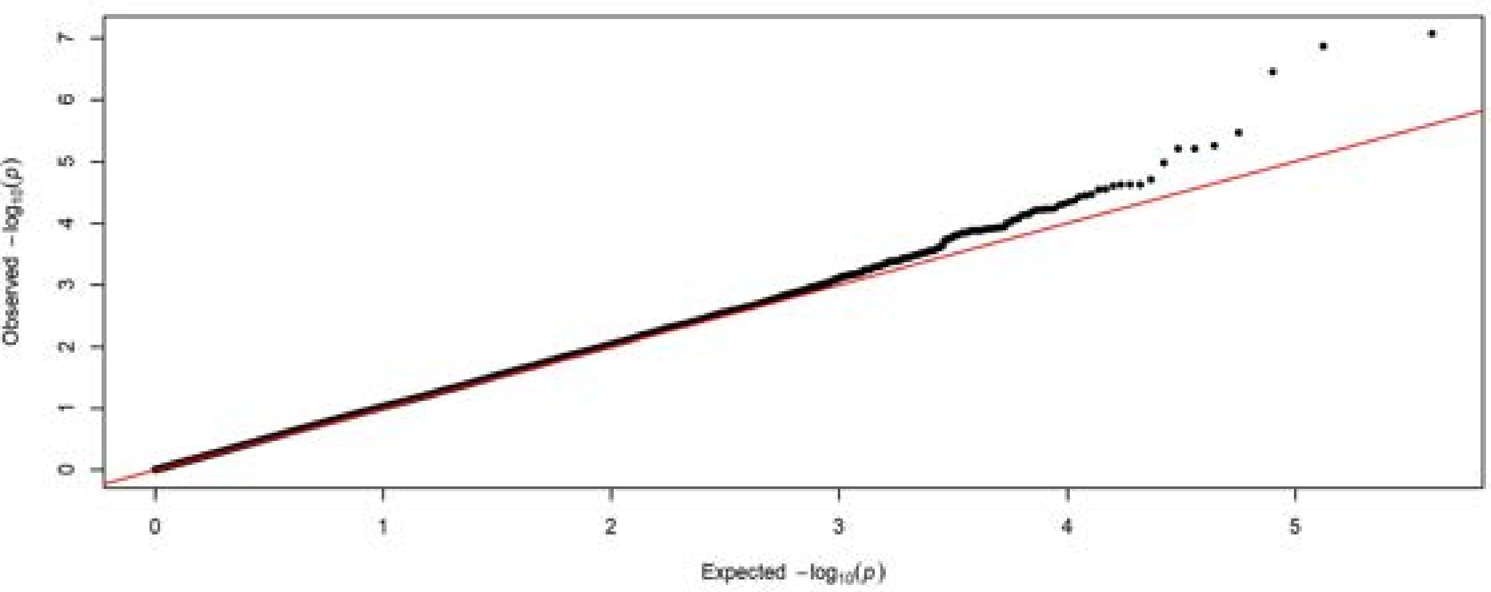

Q-Q Plot Latency Score Day 2 - Harlan 3 Subgroups - 83k SNPs (n=2208)

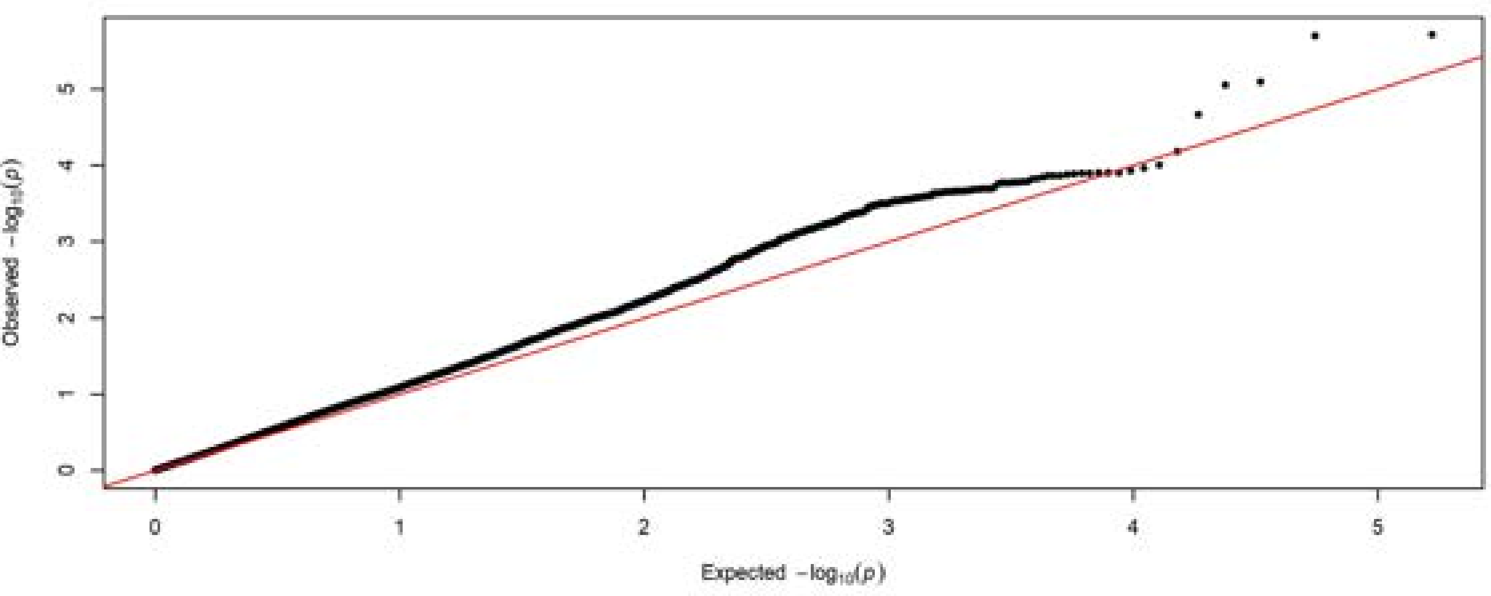

Q-Q Plot Latency Score Day 3 - Meta-analysis of 7 Subgroups - 64k SNPs (n=3932)

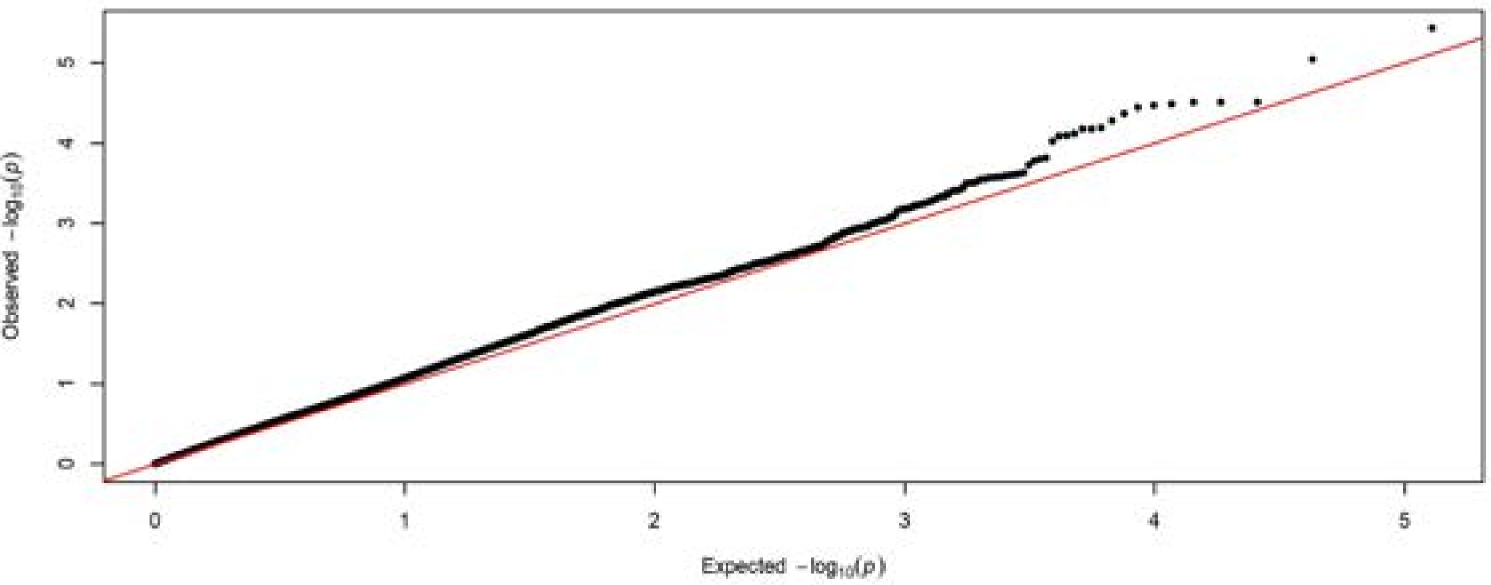

Q-Q Plot Latency Score Day 3 - Charles River 4 Subgroups - 198k SNPs (n=1727)

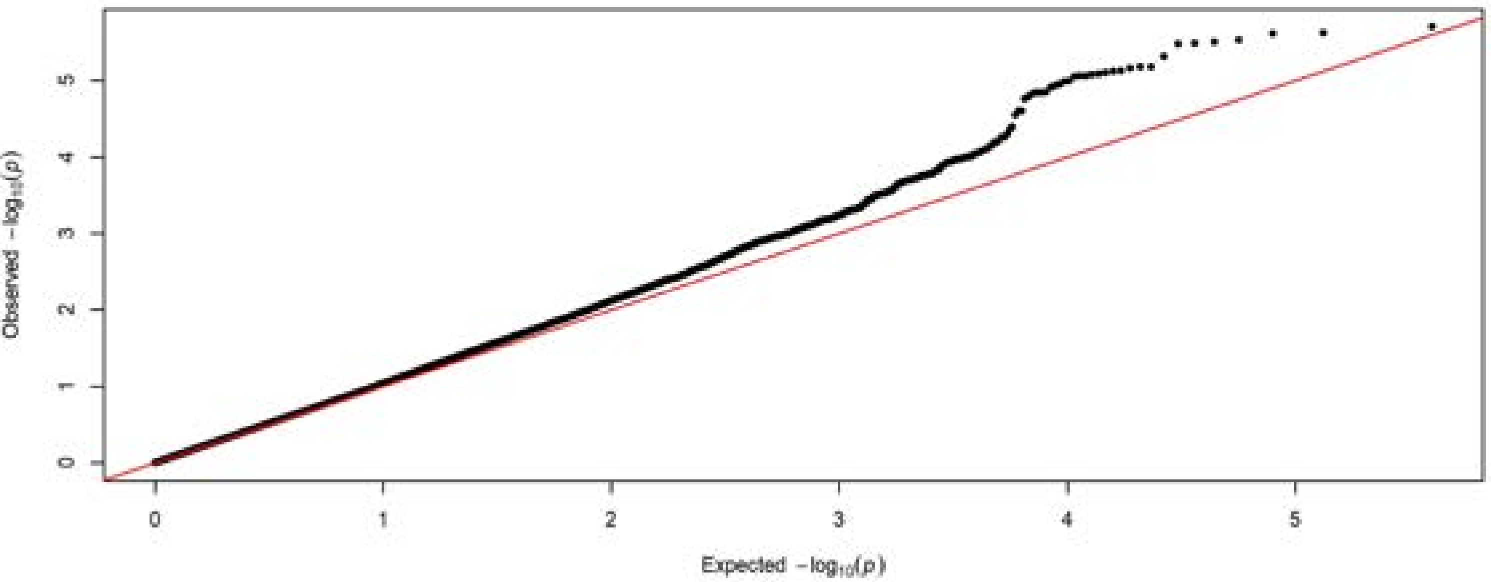

Q-Q Plot Latency Score Day 3 - Harlan 3 Subgroups - 83k SNPs (n=2205)

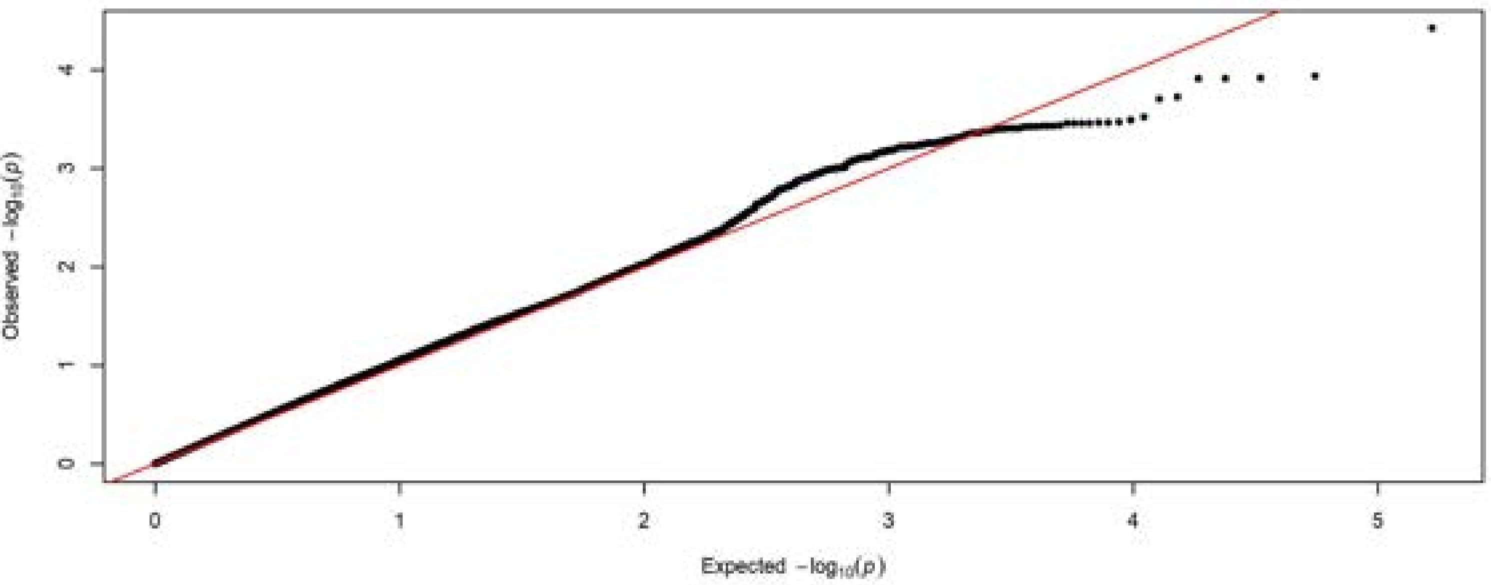

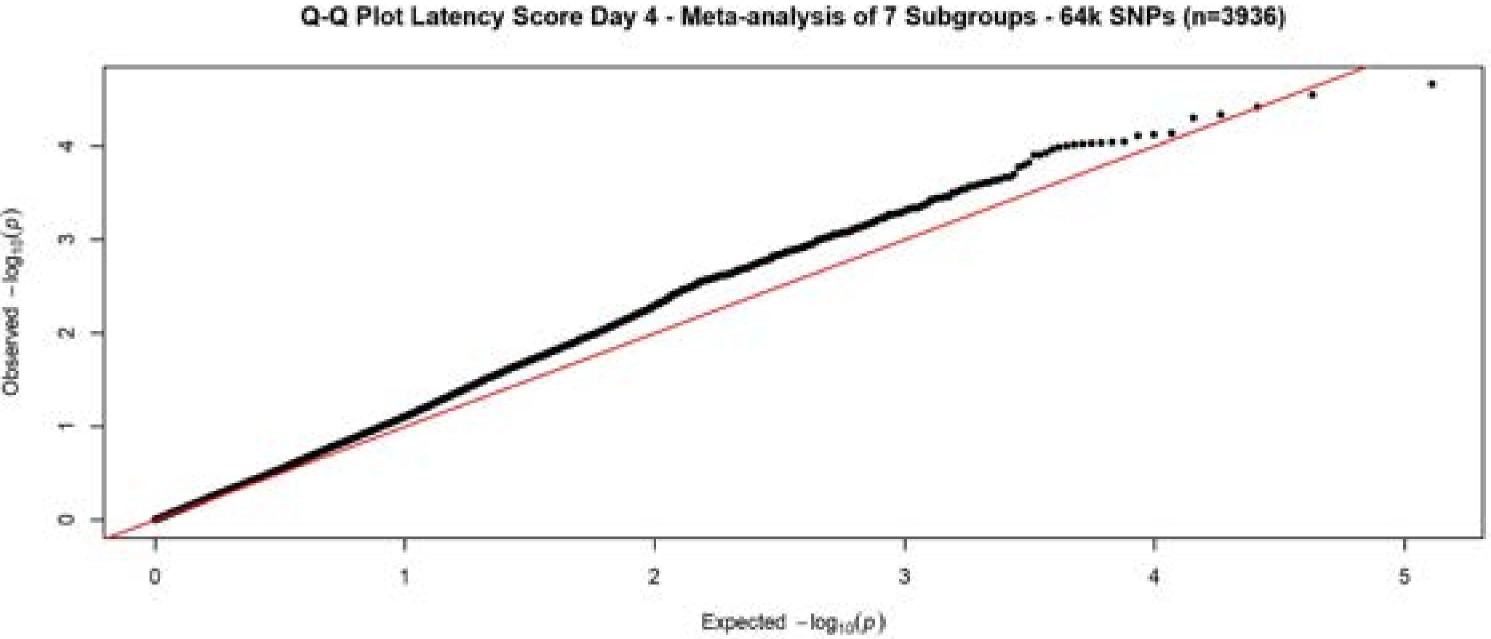
Q-Q Plot Latency Score Day 4 - Meta-analysis of 7 Subgroups - 64k SNPs (n=3936)
Observed $-\log_{10}(p)$
Expected $-\log_{10}(p)$

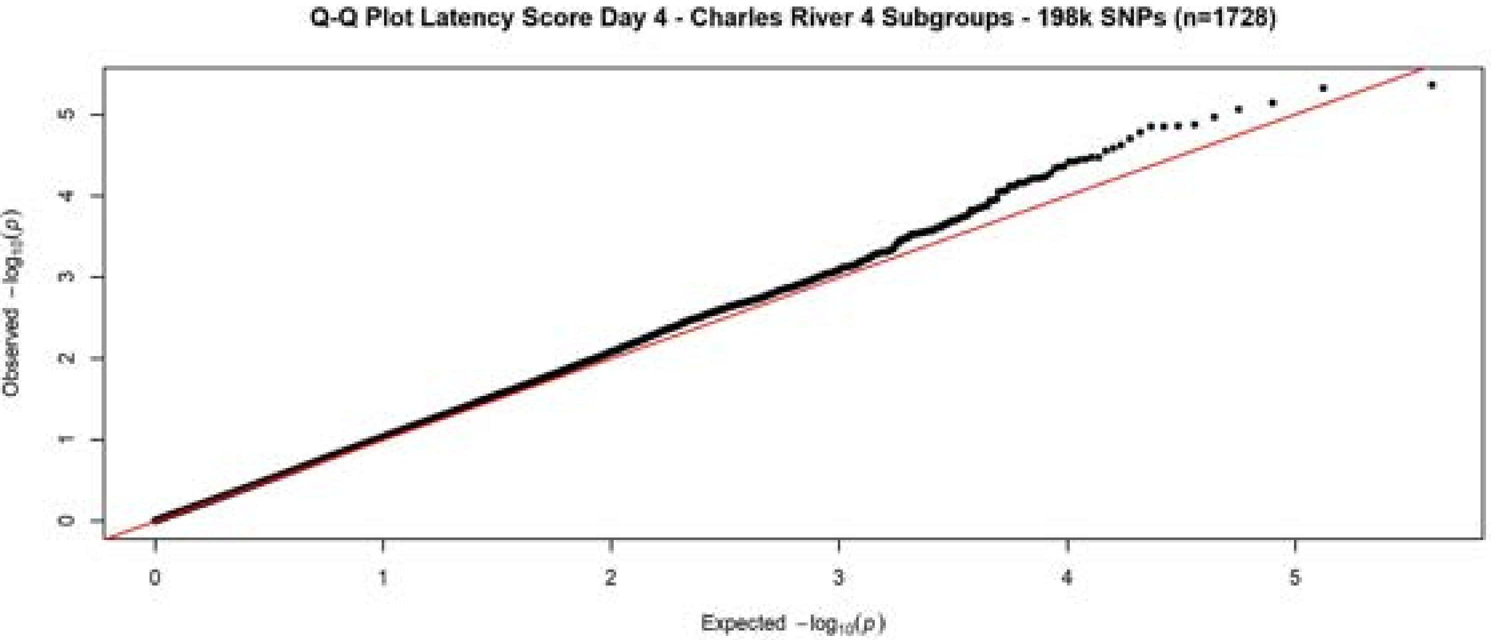
Q-Q Plot Latency Score Day 4 - Charles River 4 Subgroups - 198k SNPs (n=1728)
Observed $-\log_{10}(p)$
Expected $-\log_{10}(p)$

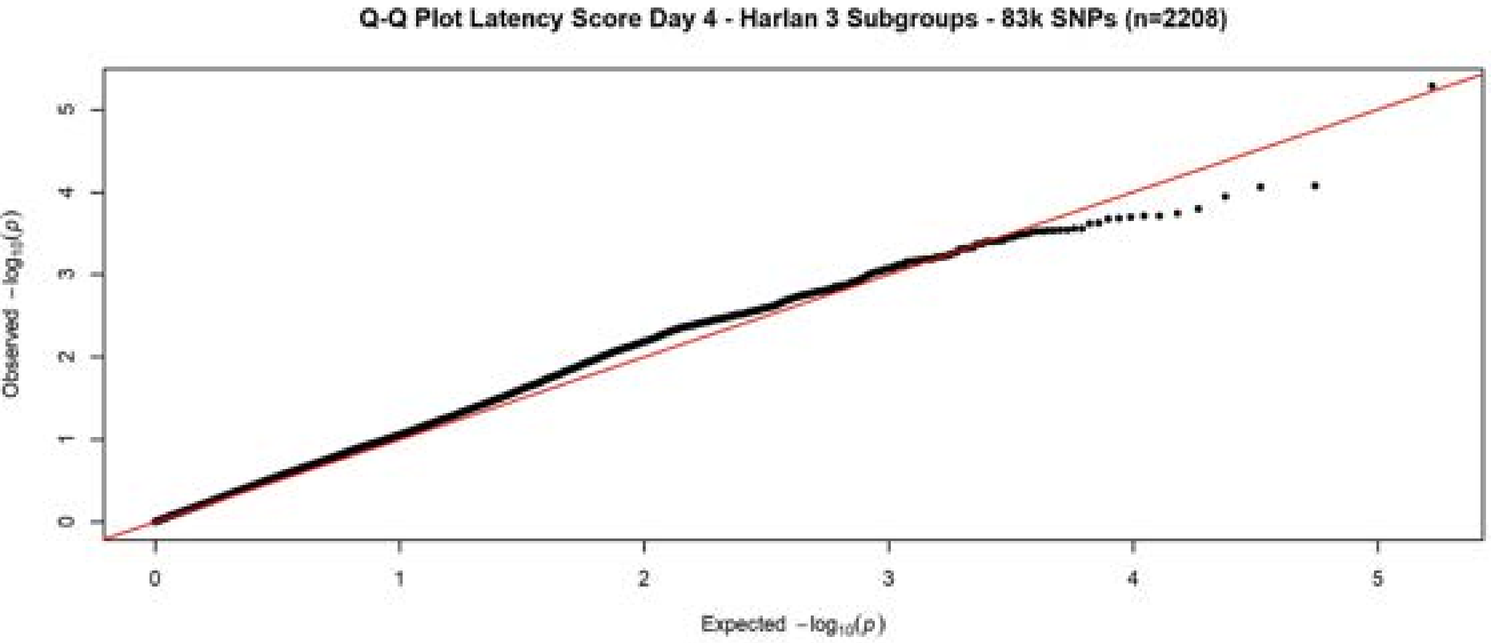
Q-Q Plot Latency Score Day 4 - Harlan 3 Subgroups - 83k SNPs (n=2208)
Observed $-\log_{10}(p)$
Expected $-\log_{10}(p)$

Q-Q Plot Latency Score Day 5 - Meta-analysis of 7 Subgroups - 64k SNPs (n=3936)

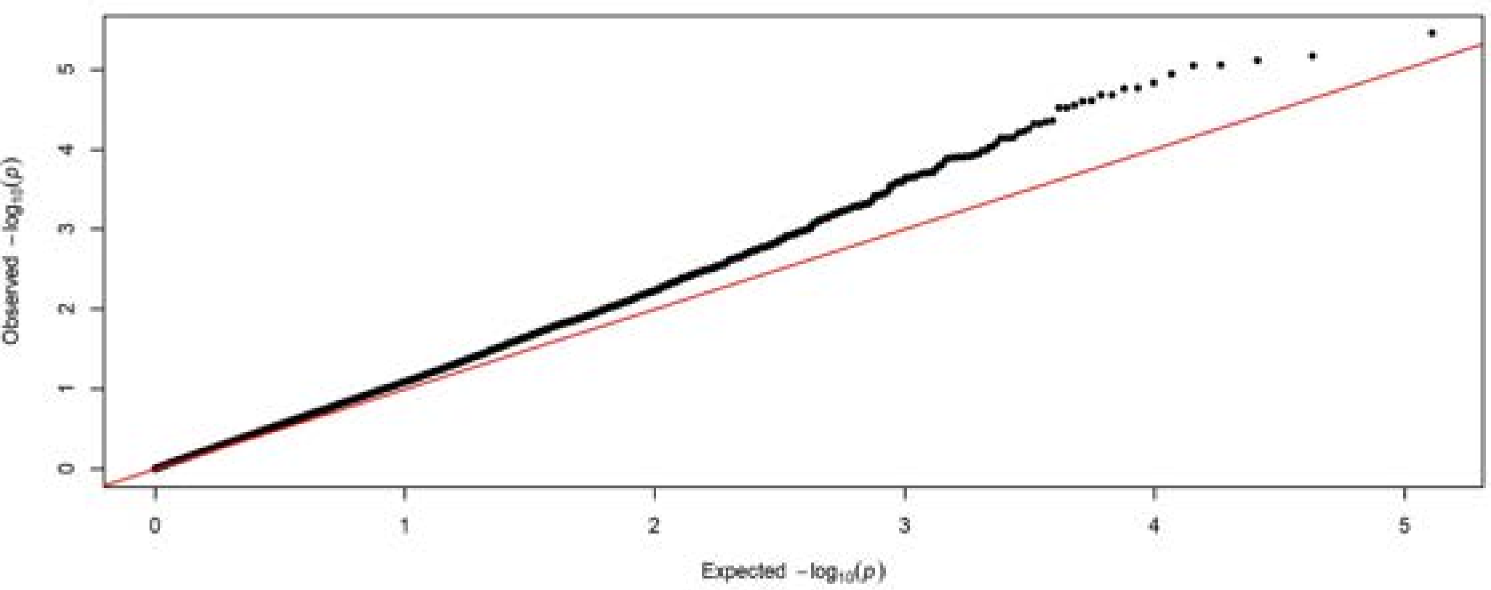

Q-Q Plot Latency Score Day 5 - Charles River 4 Subgroups - 198k SNPs (n=1728)

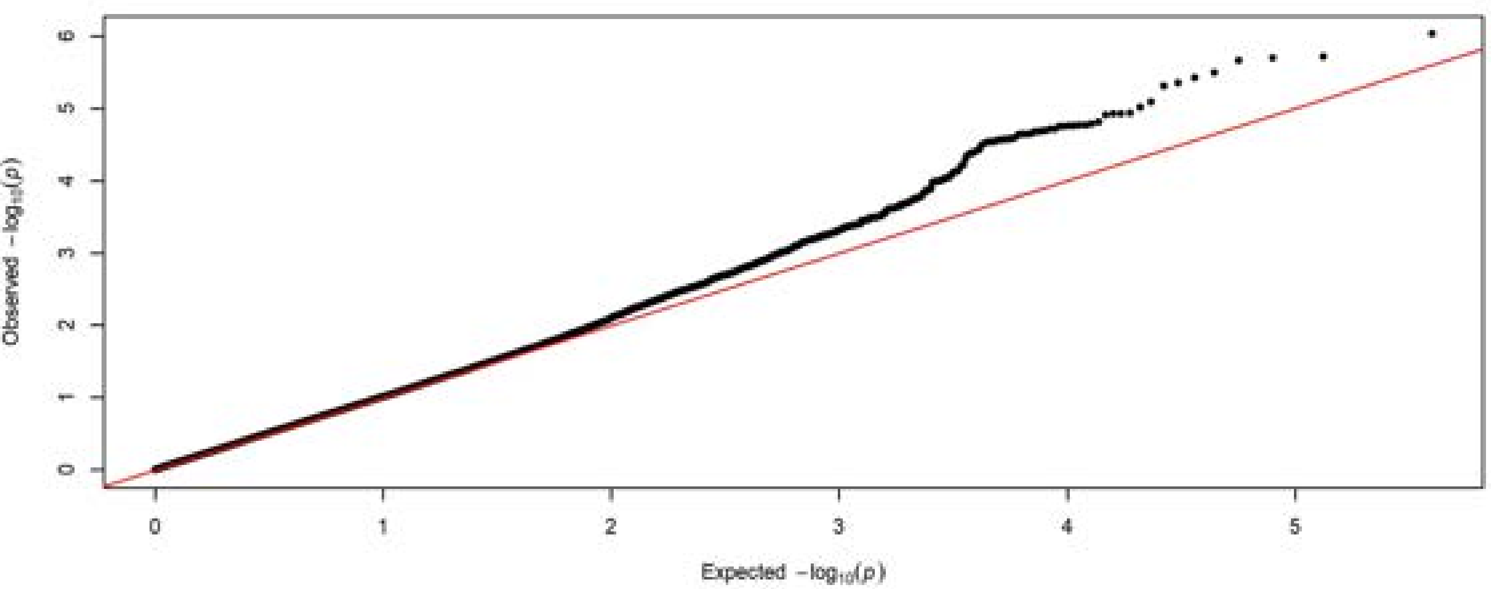

Q-Q Plot Latency Score Day 5 - Harlan 3 Subgroups - 83k SNPs (n=2208)

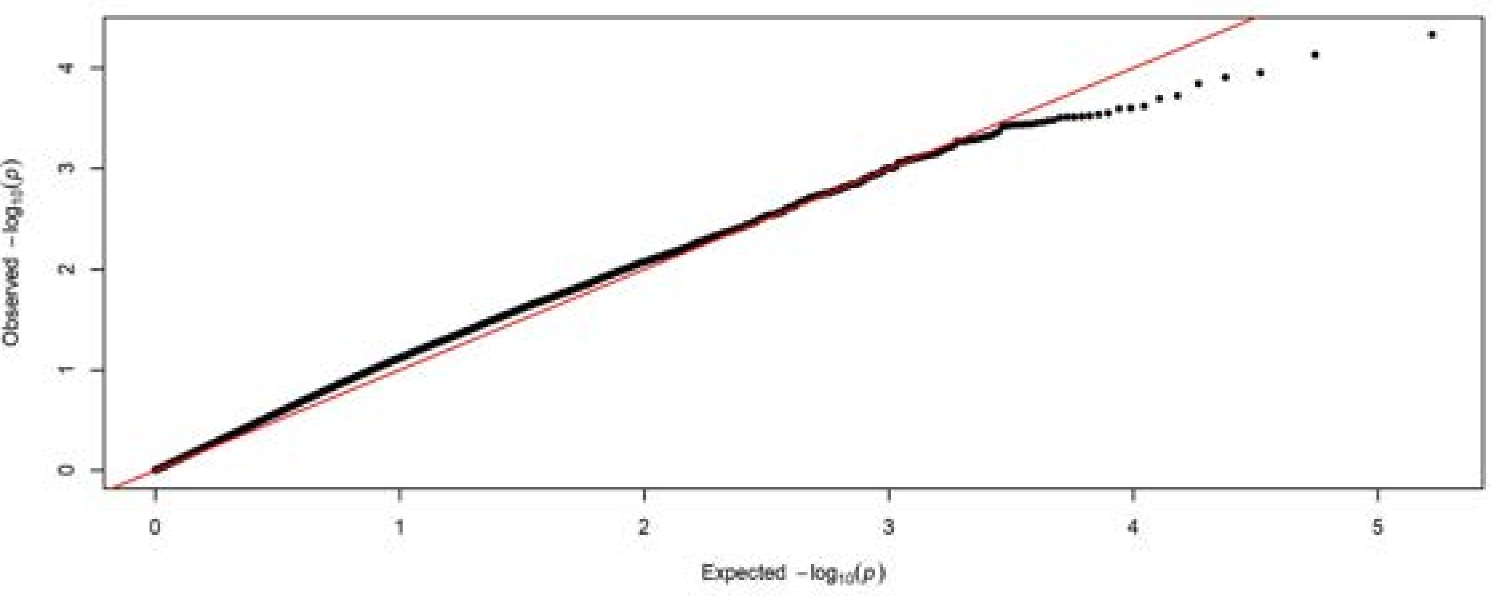

Q-Q Plot Lever Presses Day 1 - Meta-analysis of 7 Subgroups - 64k SNPs (n=3933)

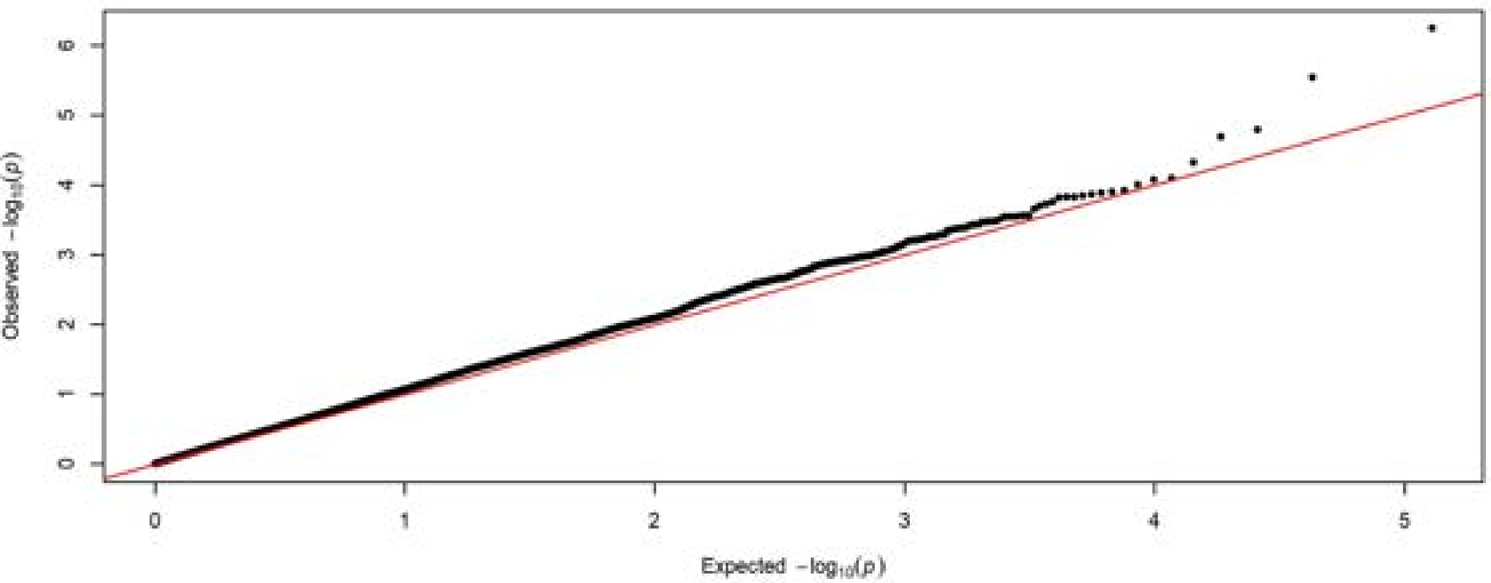

Q-Q Plot Lever Presses Day 1 - Charles River 4 Subgroups - 198k SNPs (n=1727)

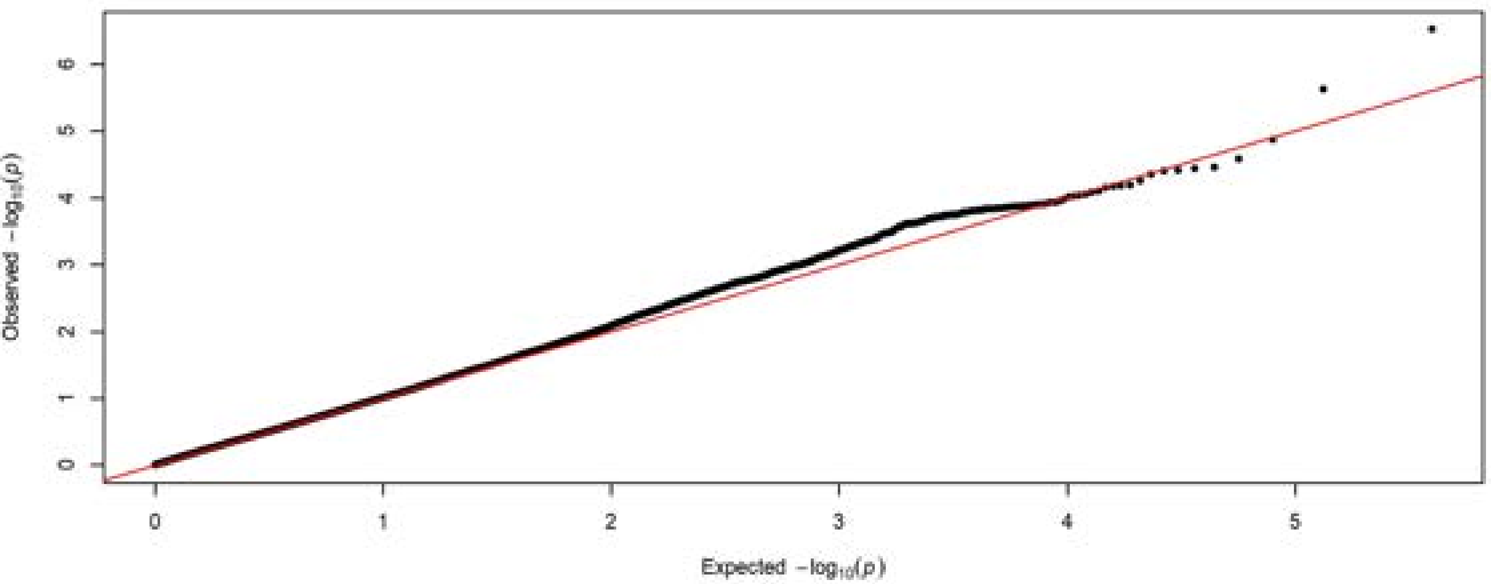

Q-Q Plot Lever Presses Day 1 - Harlan 3 Subgroups - 83k SNPs (n=2206)

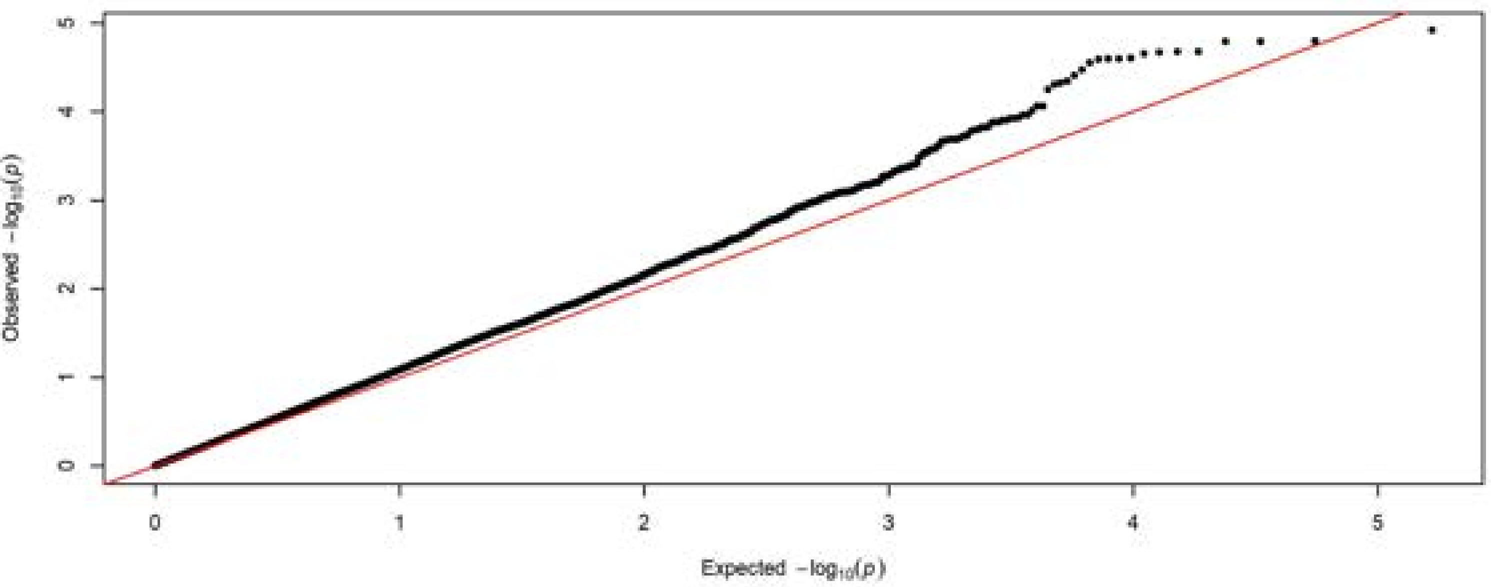

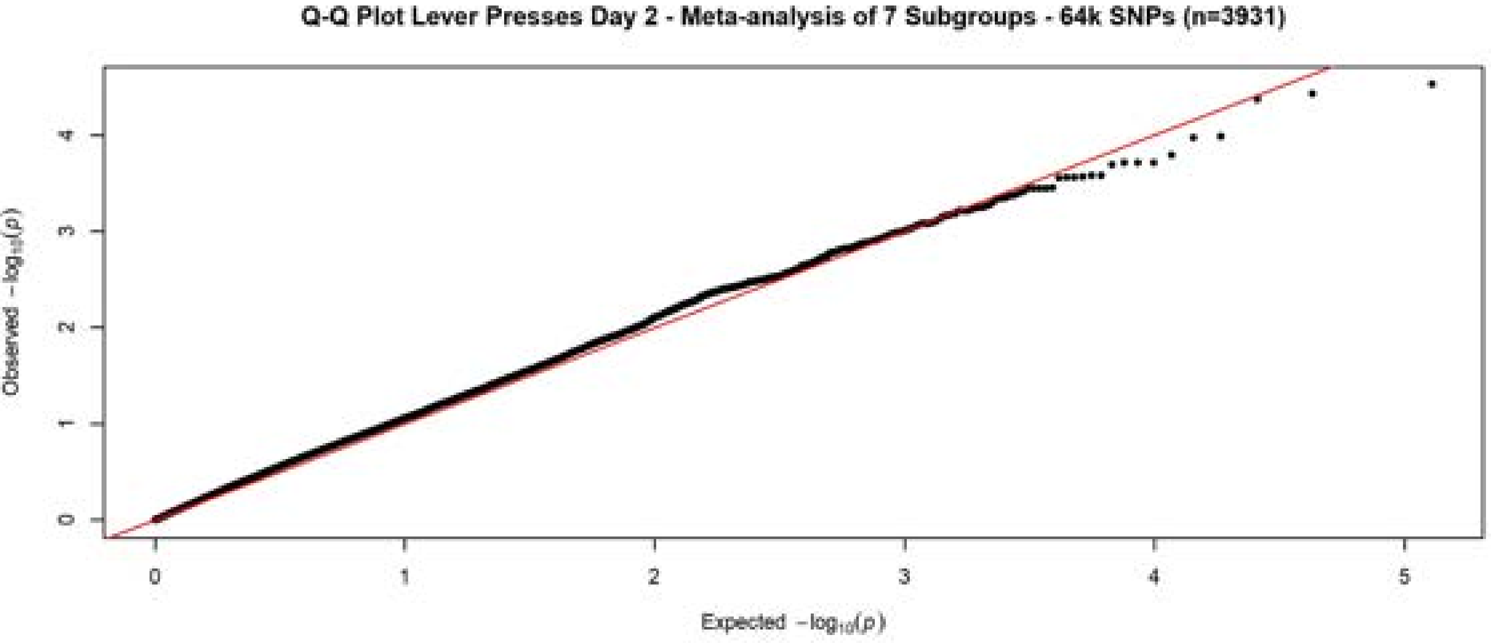

Q-Q Plot Lever Presses Day 2 - Meta-analysis of 7 Subgroups - 64k SNPs (n=3931)
Observed $-\log_{10}(p)$
Expected $-\log_{10}(p)$

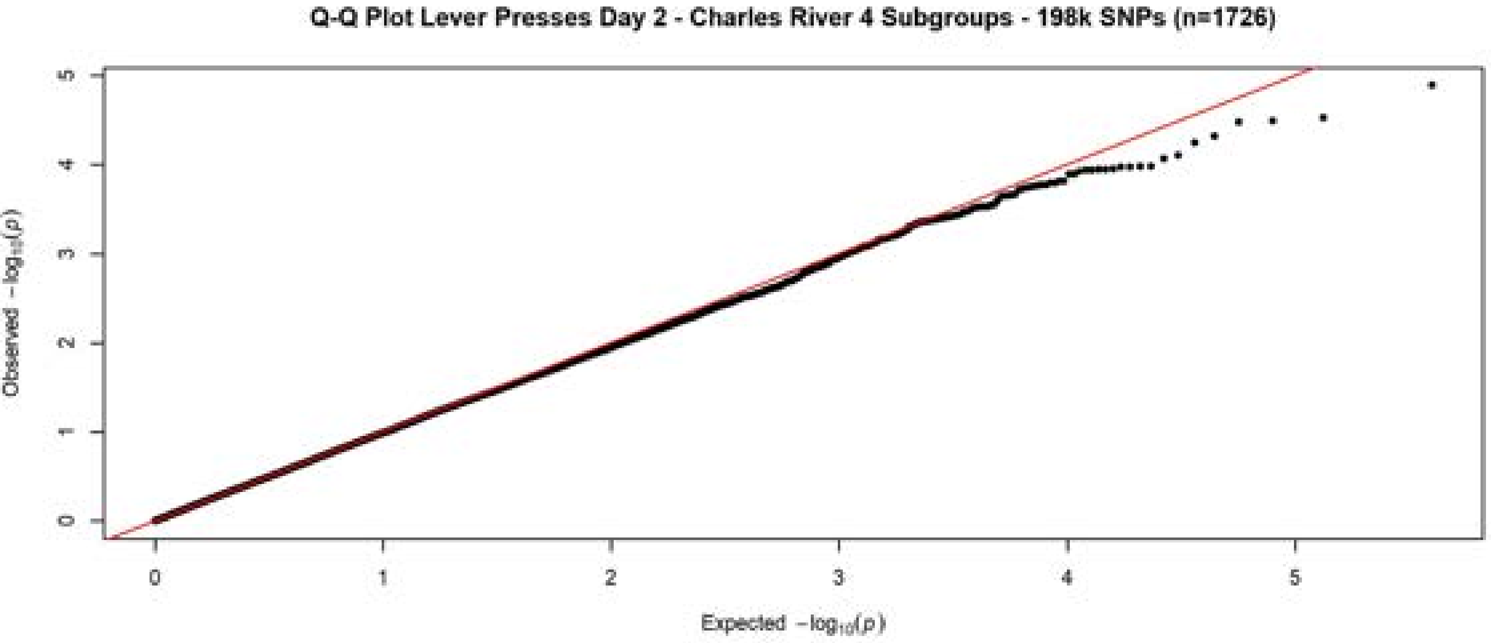

Q-Q Plot Lever Presses Day 2 - Charles River 4 Subgroups - 198k SNPs (n=1726)
Observed $-\log_{10}(p)$
Expected $-\log_{10}(p)$

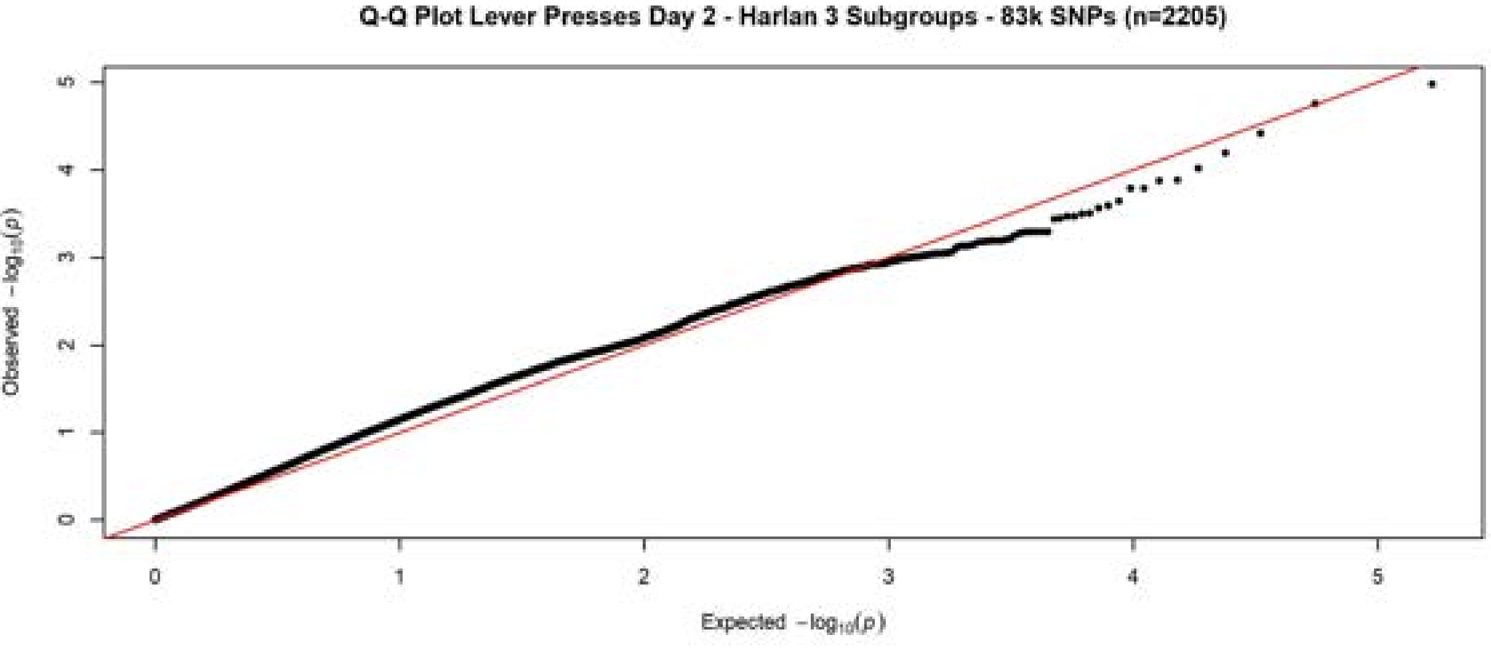

Q-Q Plot Lever Presses Day 2 - Harlan 3 Subgroups - 83k SNPs (n=2205)
Observed $-\log_{10}(p)$
Expected $-\log_{10}(p)$

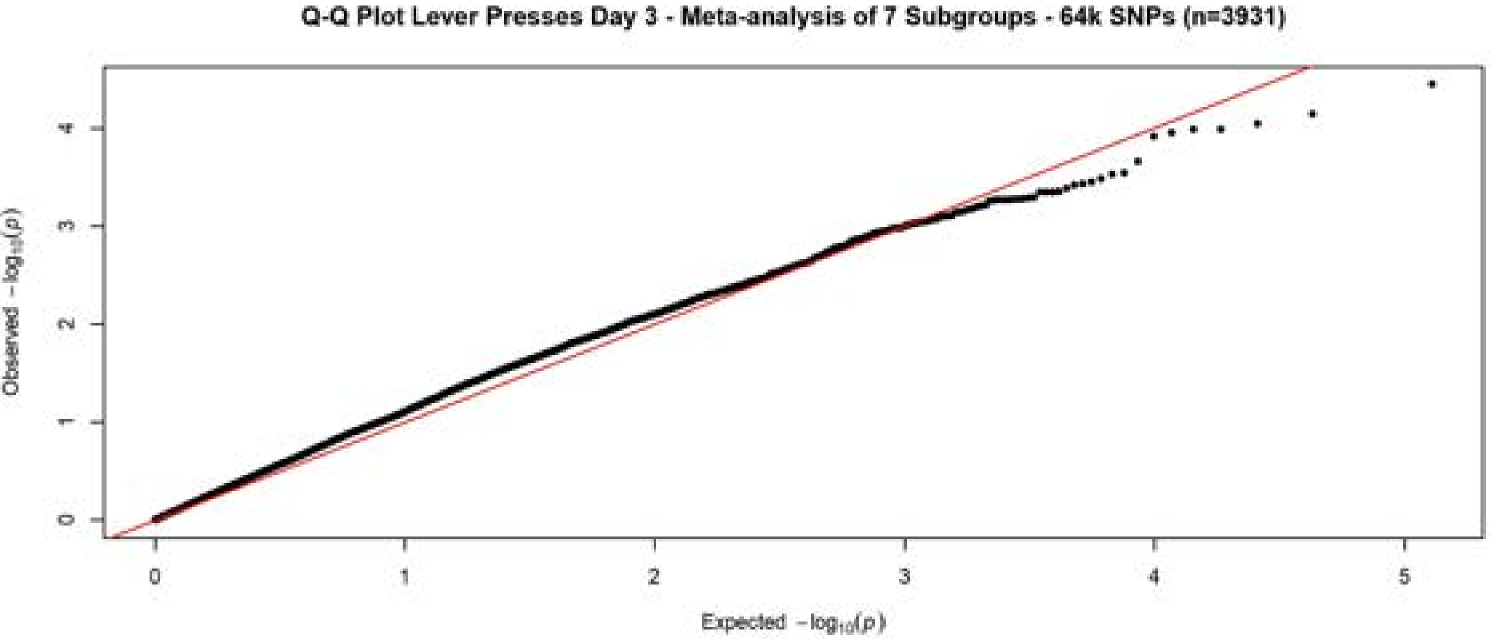
Q-Q Plot Lever Presses Day 3 - Meta-analysis of 7 Subgroups - 64k SNPs (n=3931)
Observed $-\log_{10}(p)$
Expected $-\log_{10}(p)$

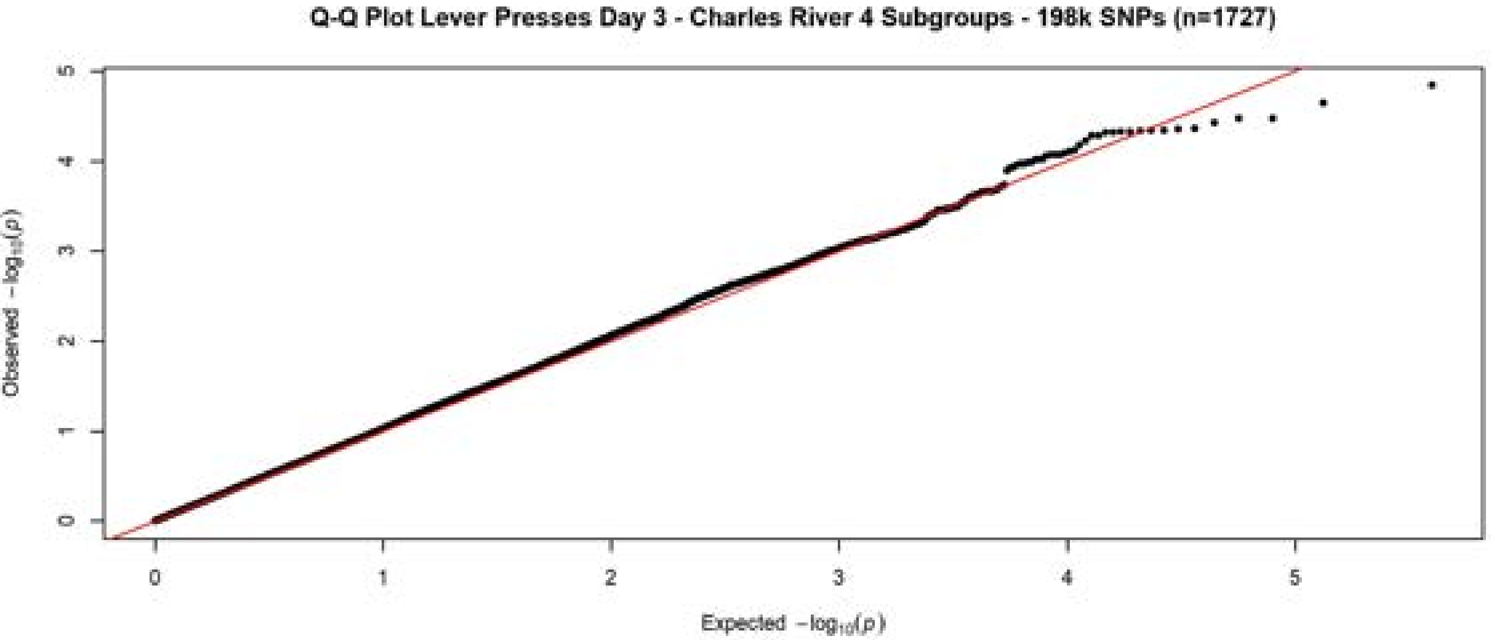
Q-Q Plot Lever Presses Day 3 - Charles River 4 Subgroups - 198k SNPs (n=1727)
Observed $-\log_{10}(p)$
Expected $-\log_{10}(p)$

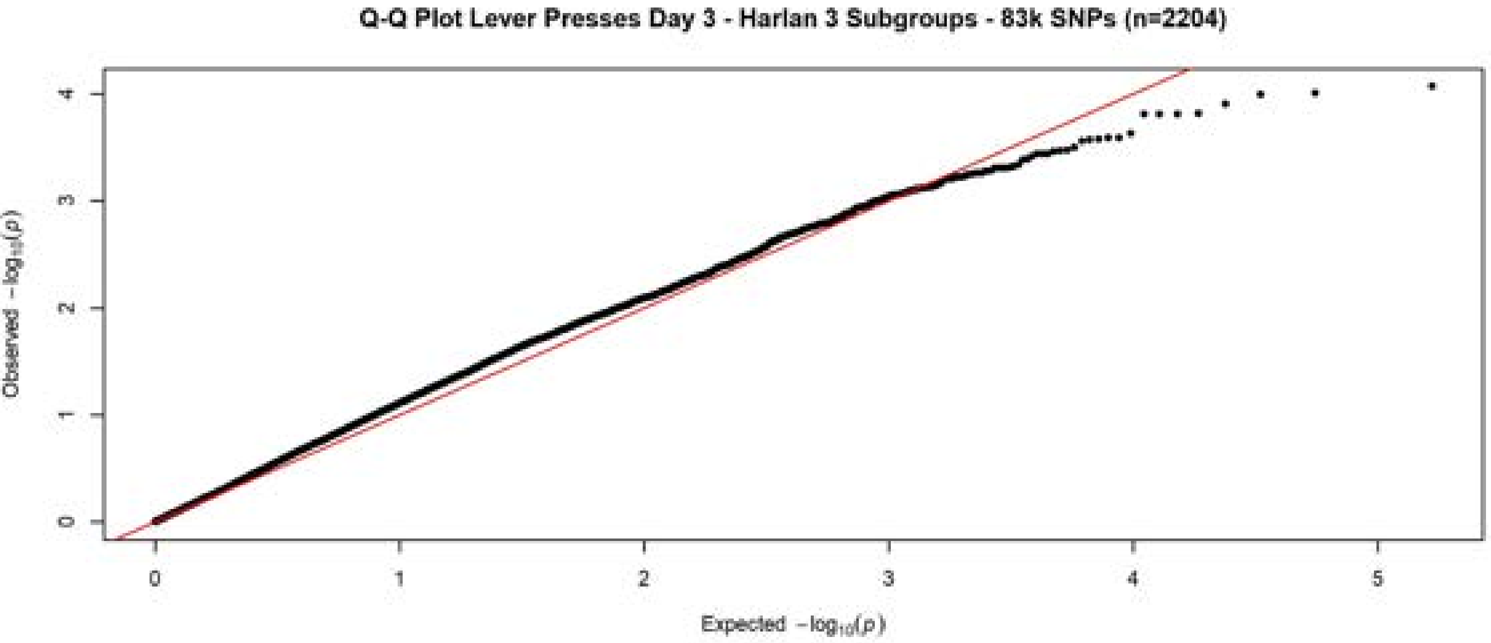
Q-Q Plot Lever Presses Day 3 - Harlan 3 Subgroups - 83k SNPs (n=2204)
Observed $-\log_{10}(p)$
Expected $-\log_{10}(p)$

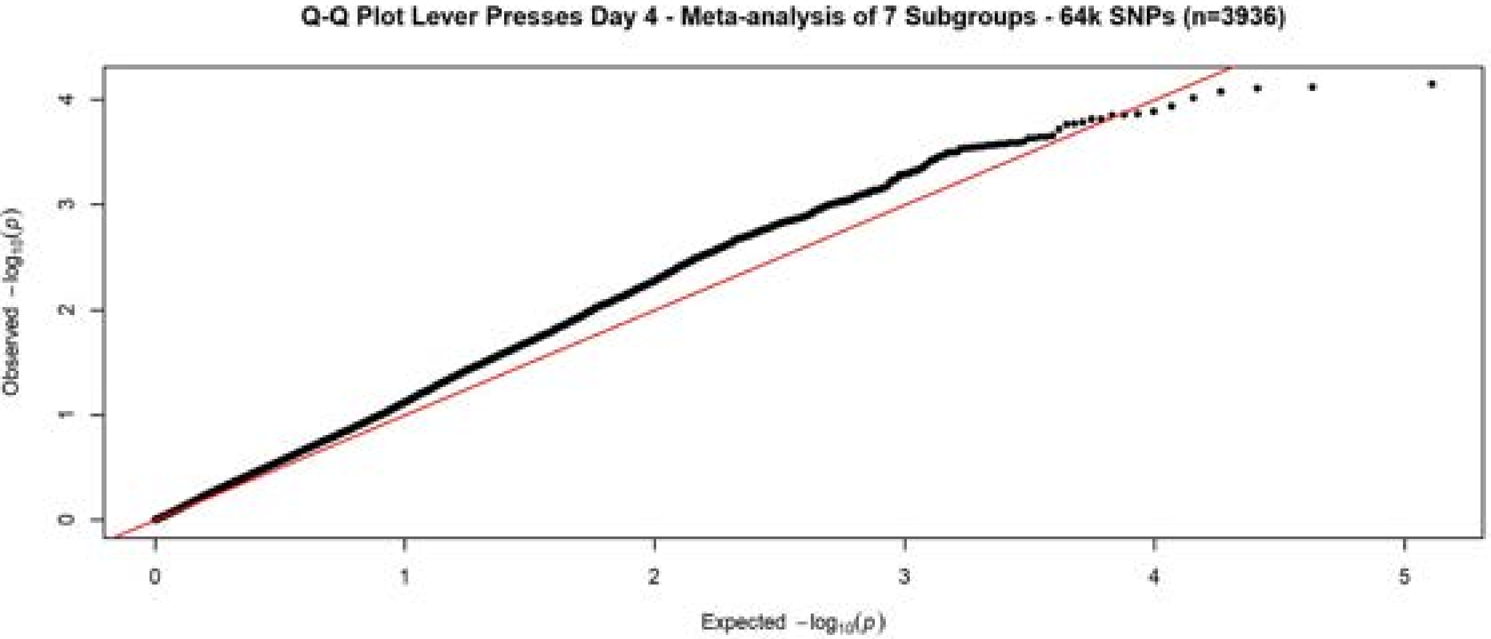

Q-Q Plot Lever Presses Day 4 - Meta-analysis of 7 Subgroups - 64k SNPs (n=3936)
Observed $-\log_{10}(p)$
Expected $-\log_{10}(p)$

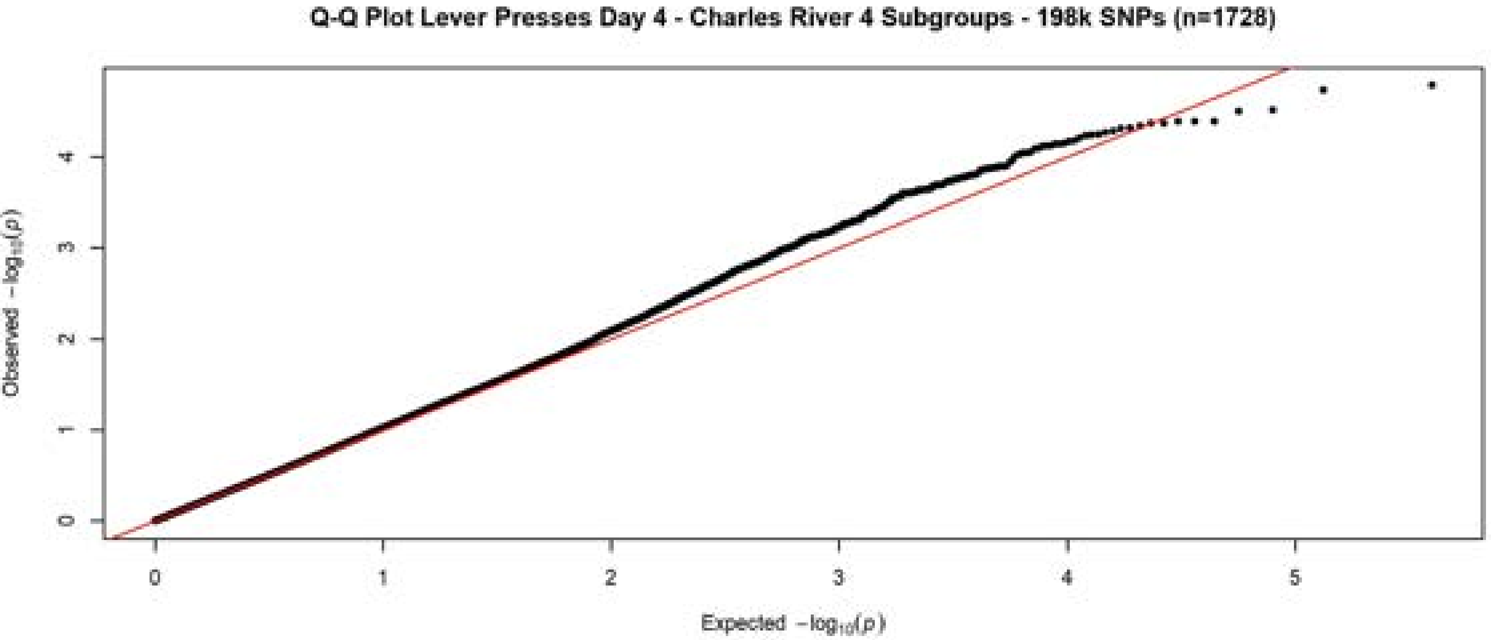

Q-Q Plot Lever Presses Day 4 - Charles River 4 Subgroups - 198k SNPs (n=1728)
Observed $-\log_{10}(p)$
Expected $-\log_{10}(p)$

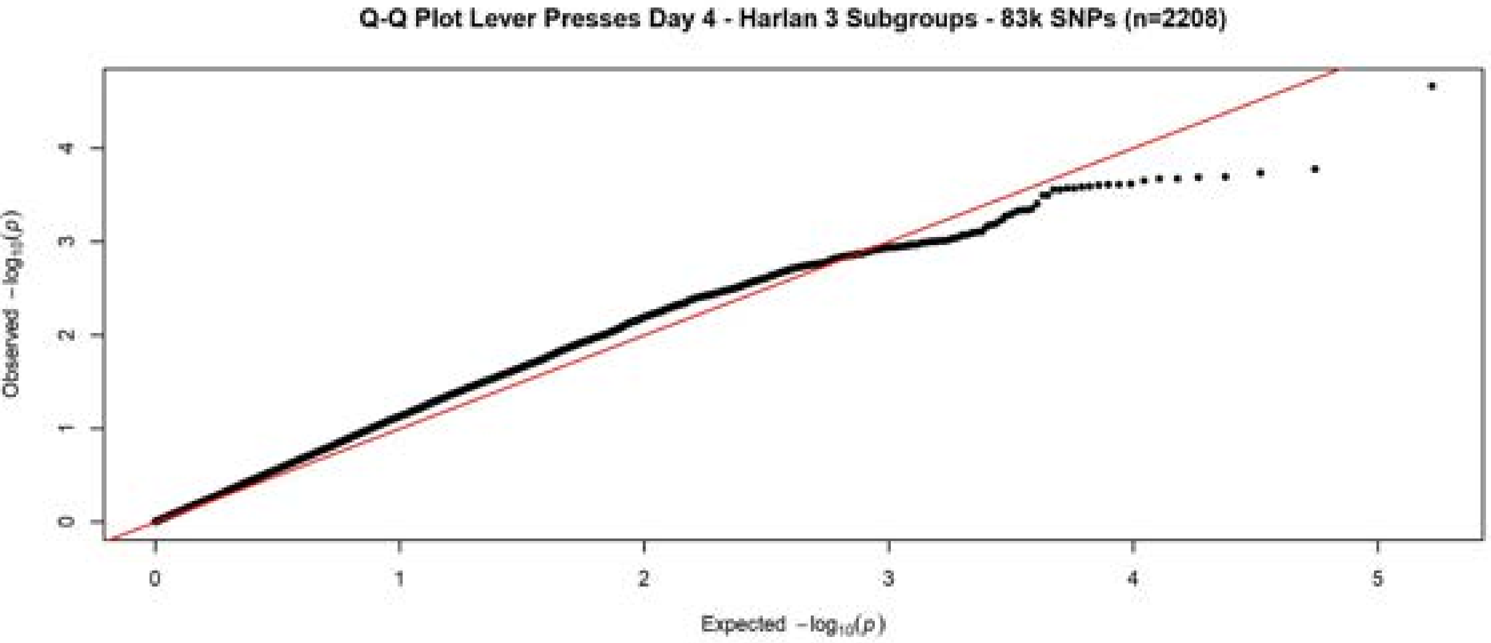

Q-Q Plot Lever Presses Day 4 - Harlan 3 Subgroups - 83k SNPs (n=2208)
Observed $-\log_{10}(p)$
Expected $-\log_{10}(p)$

Q-Q Plot Lever Presses Day 5 - Meta-analysis of 7 Subgroups - 64k SNPs (n=3936)

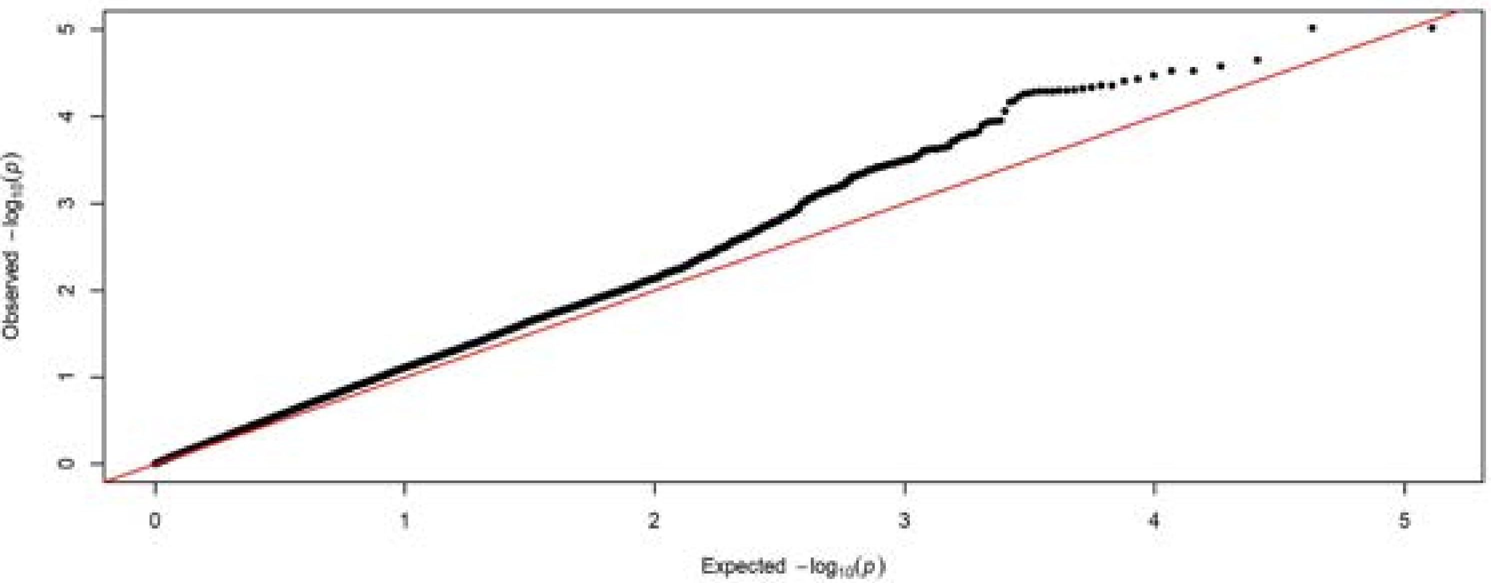

Q-Q Plot Lever Presses Day 5 - Charles River 4 Subgroups - 198k SNPs (n=1728)

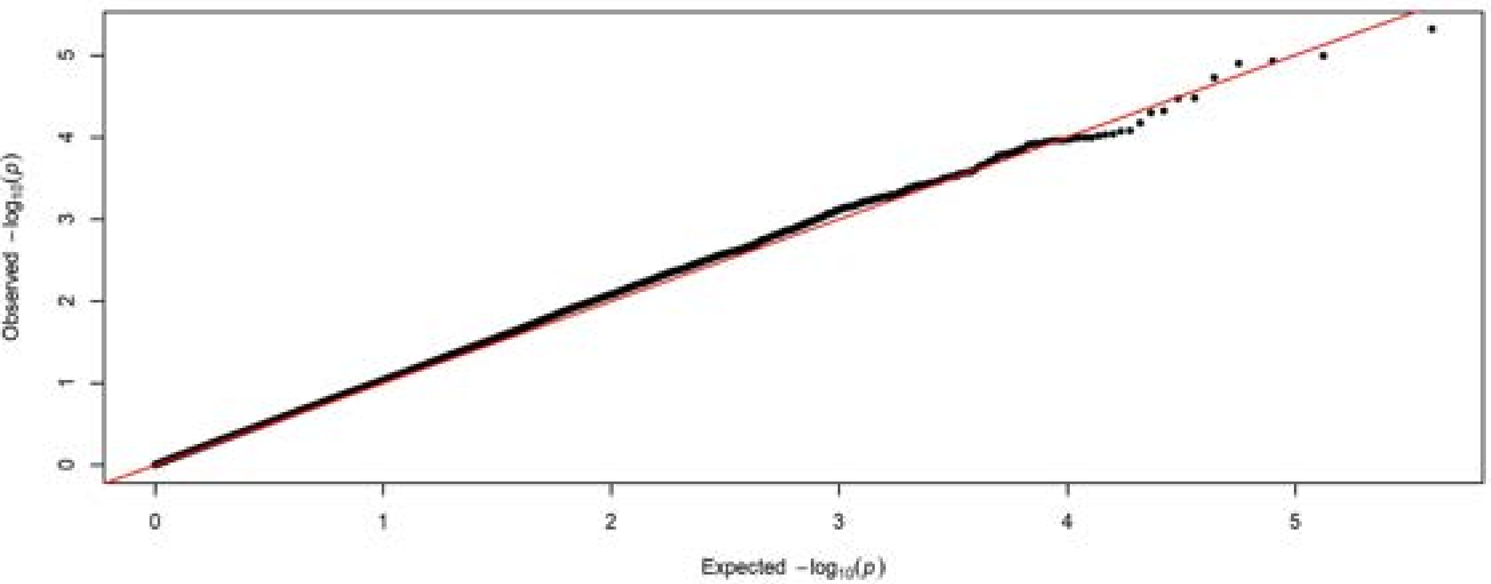

Q-Q Plot Lever Presses Day 5 - Harlan 3 Subgroups - 83k SNPs (n=2208)

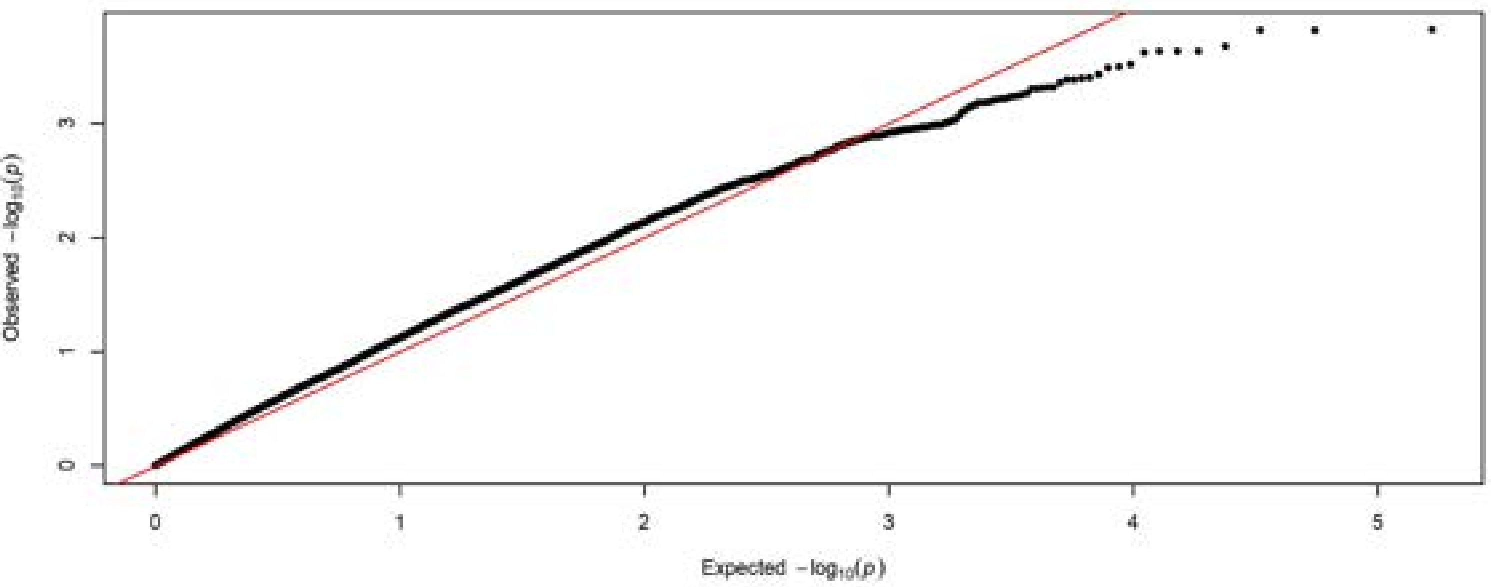

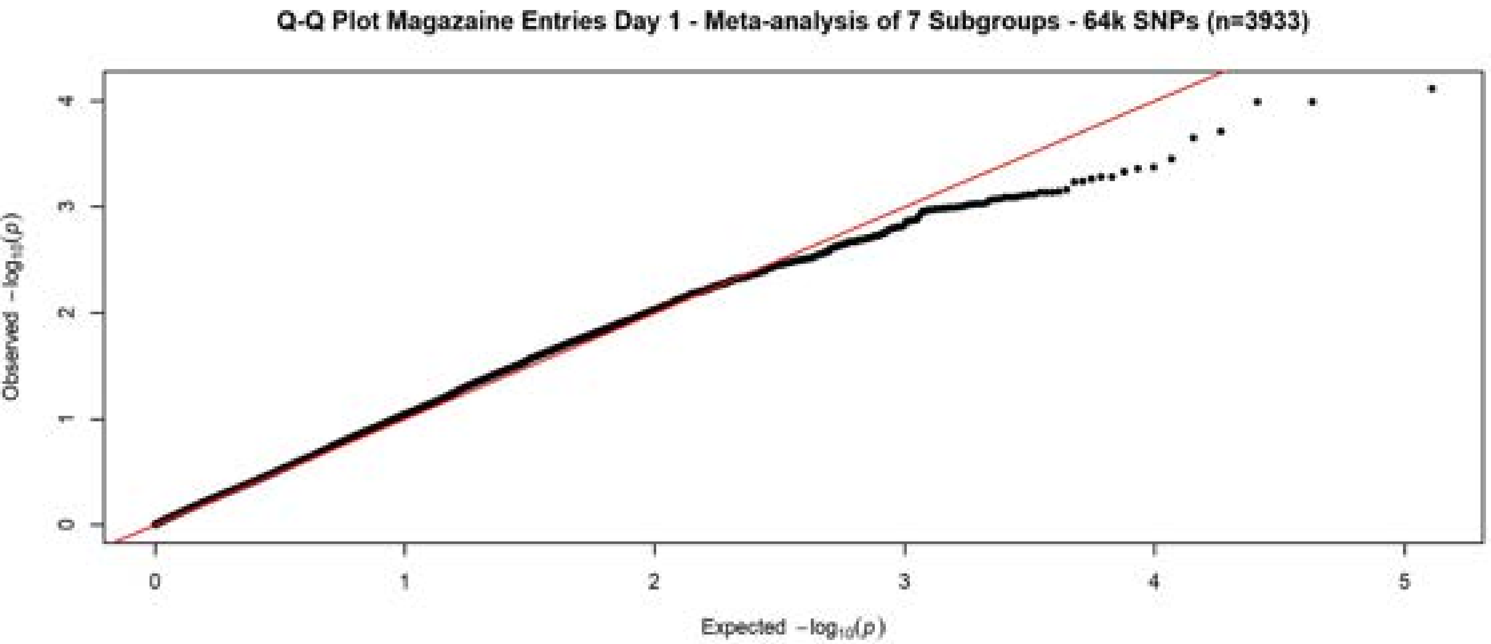
Q-Q Plot Magazaine Entries Day 1 - Meta-analysis of 7 Subgroups - 64k SNPs (n=3933)
Observed $-\log_{10}(p)$
Expected $-\log_{10}(p)$
0
1
2
3
4
5

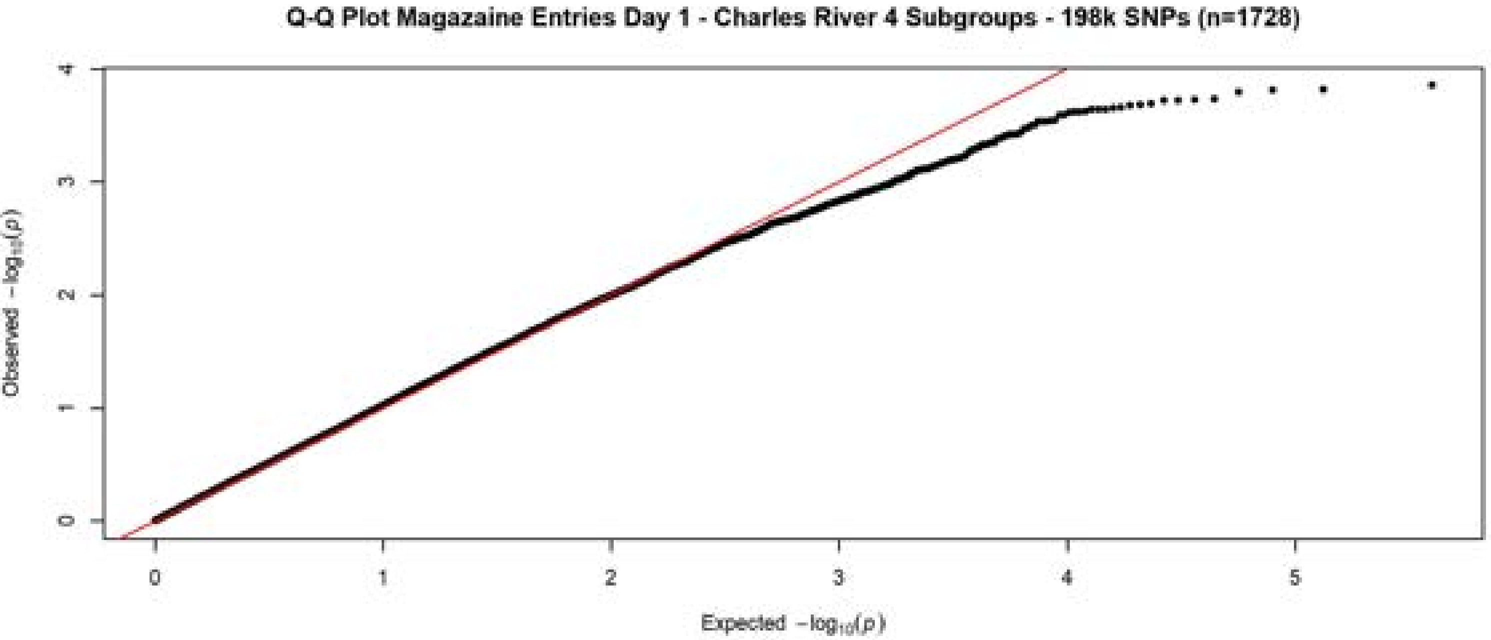
Q-Q Plot Magazaine Entries Day 1 - Charles River 4 Subgroups - 198k SNPs (n=1728)
Observed $-\log_{10}(p)$
Expected $-\log_{10}(p)$
0
1
2
3
4
5

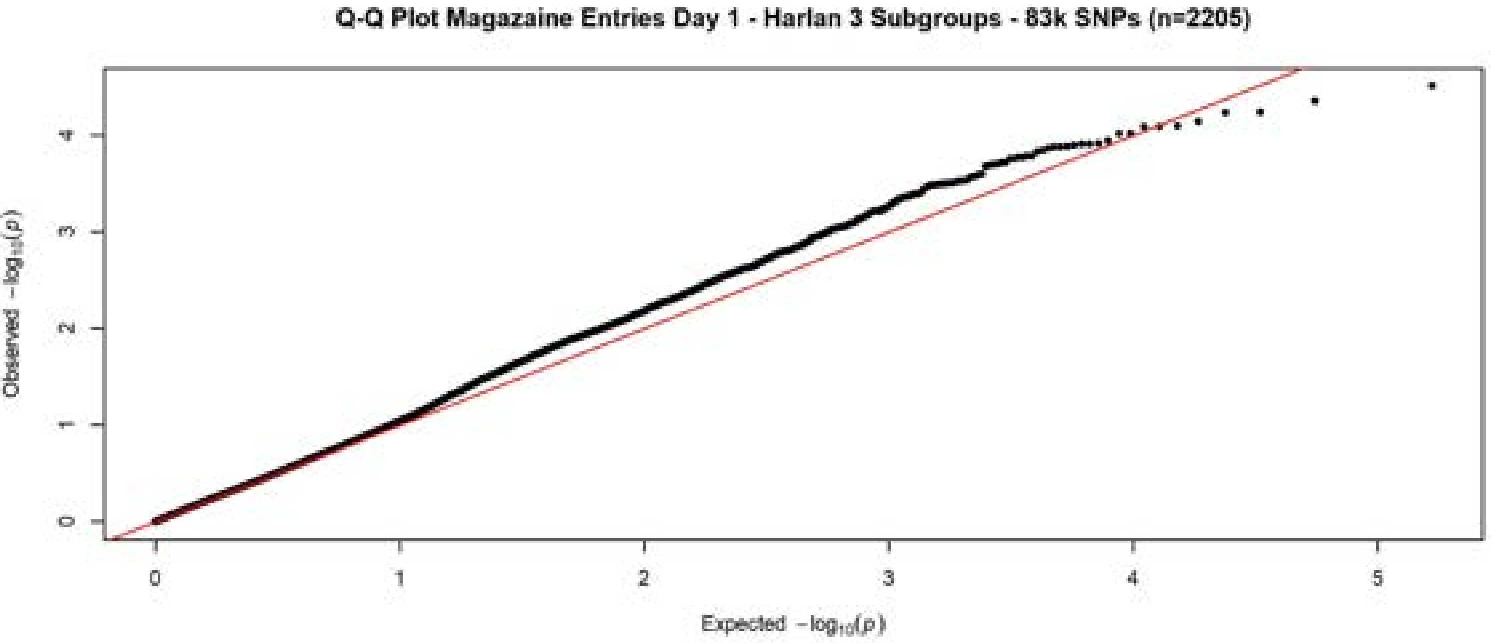
Q-Q Plot Magazaine Entries Day 1 - Harlan 3 Subgroups - 83k SNPs (n=2205)
Observed $-\log_{10}(p)$
Expected $-\log_{10}(p)$
0
1
2
3
4
5

Q-Q Plot Magazaine Entries Day 2 - Meta-analysis of 7 Subgroups - 64k SNPs (n=3933)

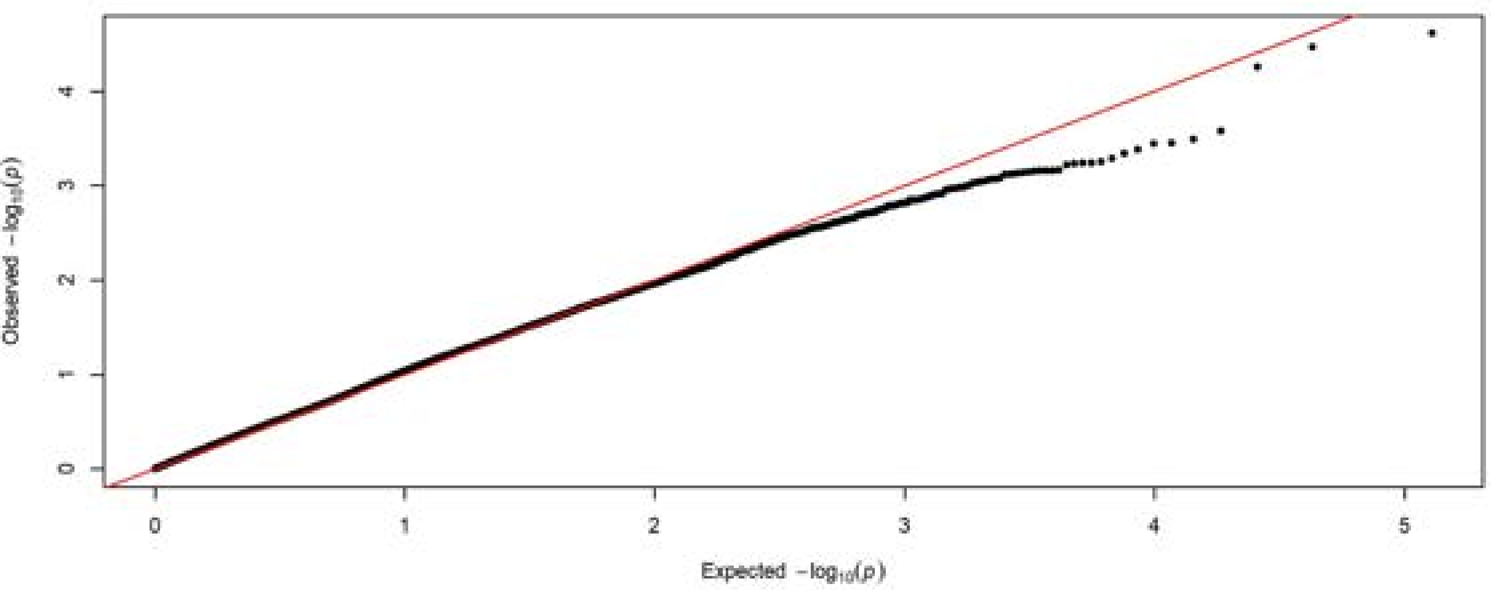

Q-Q Plot Magazaine Entries Day 2 - Charles River 4 Subgroups - 198k SNPs (n=1726)

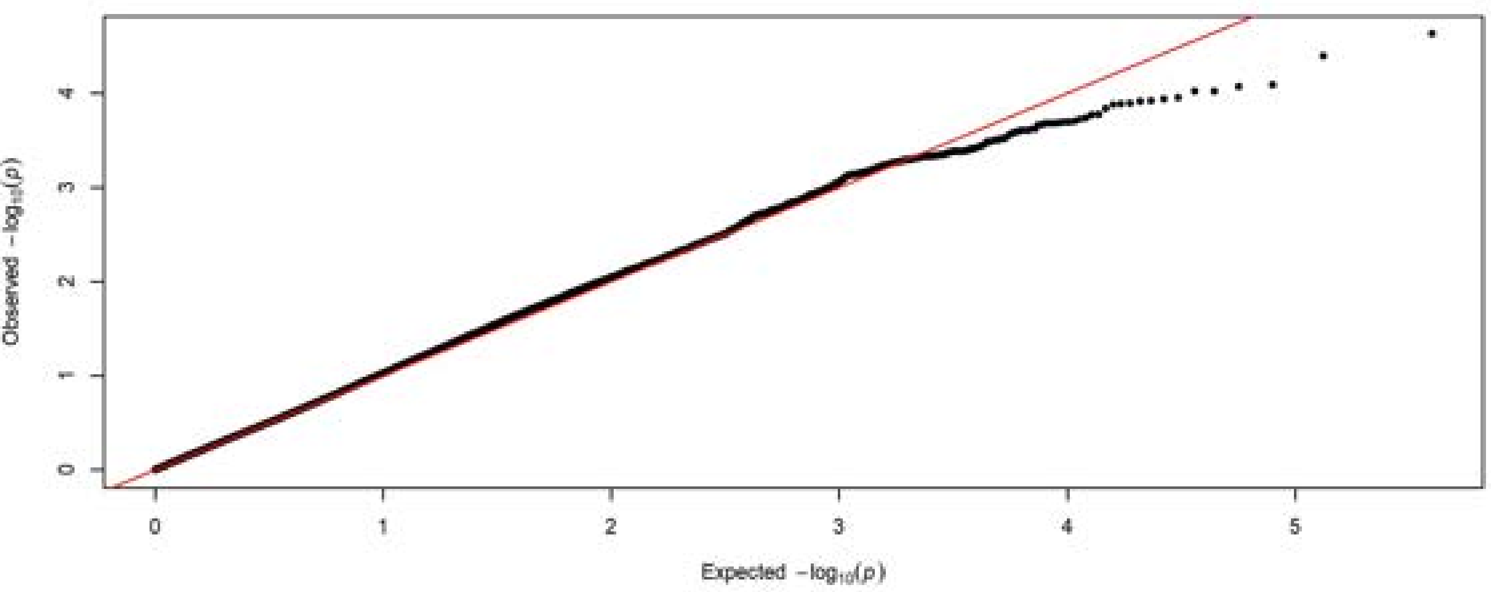

Q-Q Plot Magazaine Entries Day 2 - Harlan 3 Subgroups - 83k SNPs (n=2207)

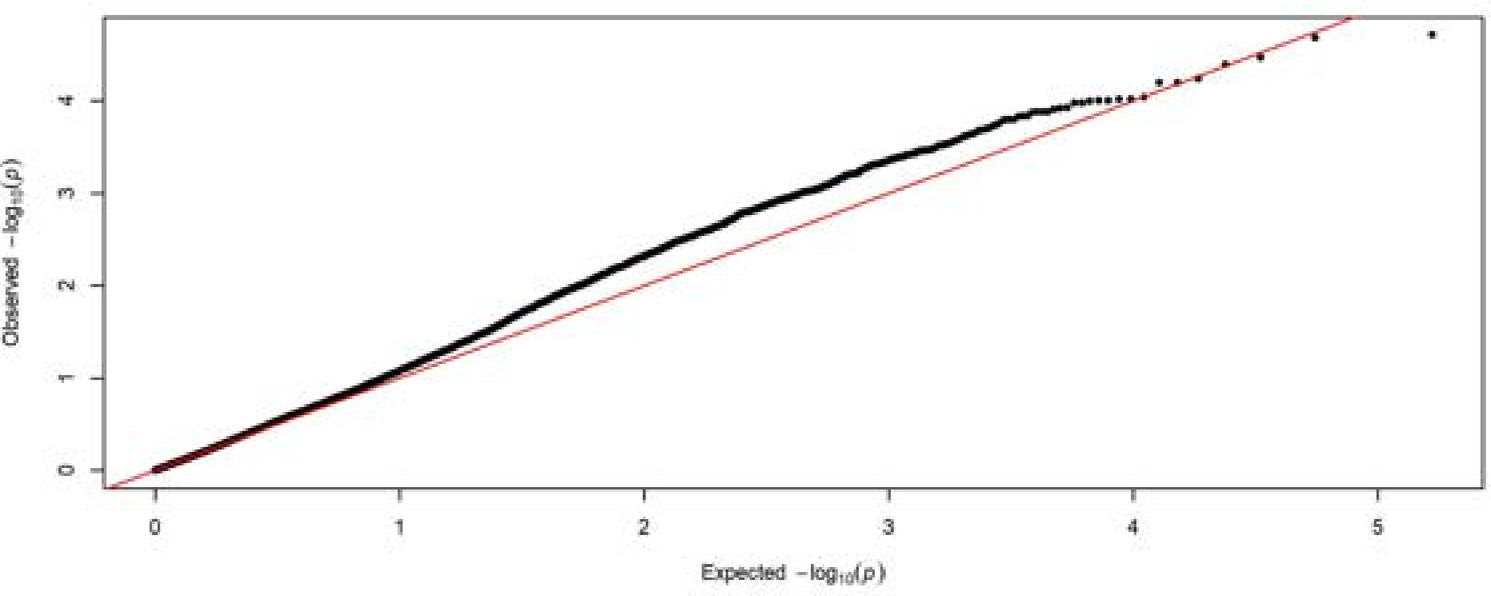

Q-Q Plot Magazaine Entries Day 3 - Meta-analysis of 7 Subgroups - 64k SNPs (n=3931)

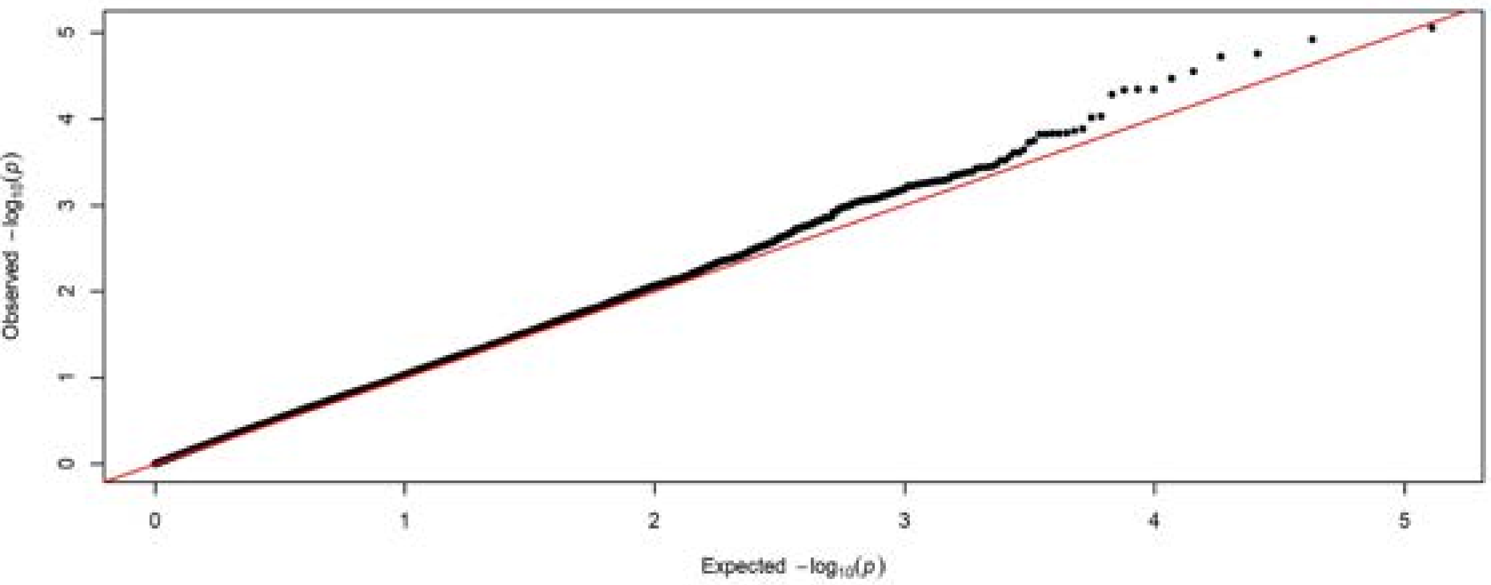

Q-Q Plot Magazaine Entries Day 3 - Charles River 4 Subgroups - 198k SNPs (n=1727)

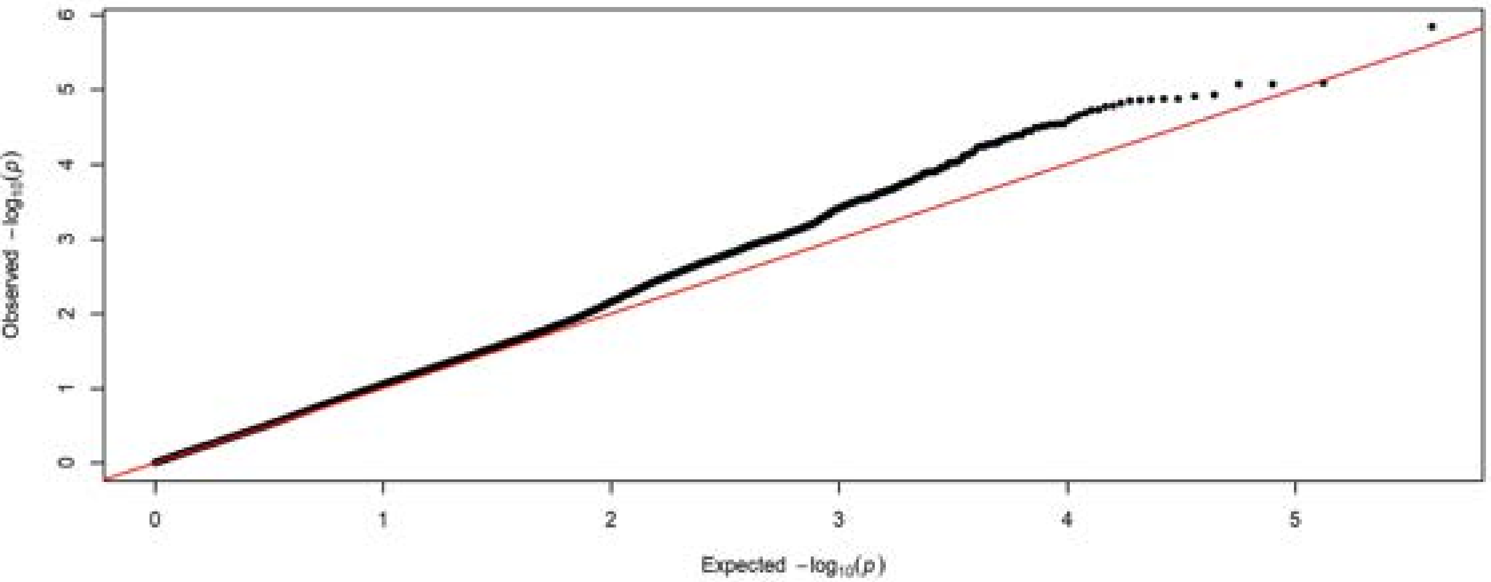

Q-Q Plot Magazaine Entries Day 3 - Harlan 3 Subgroups - 83k SNPs (n=2204)

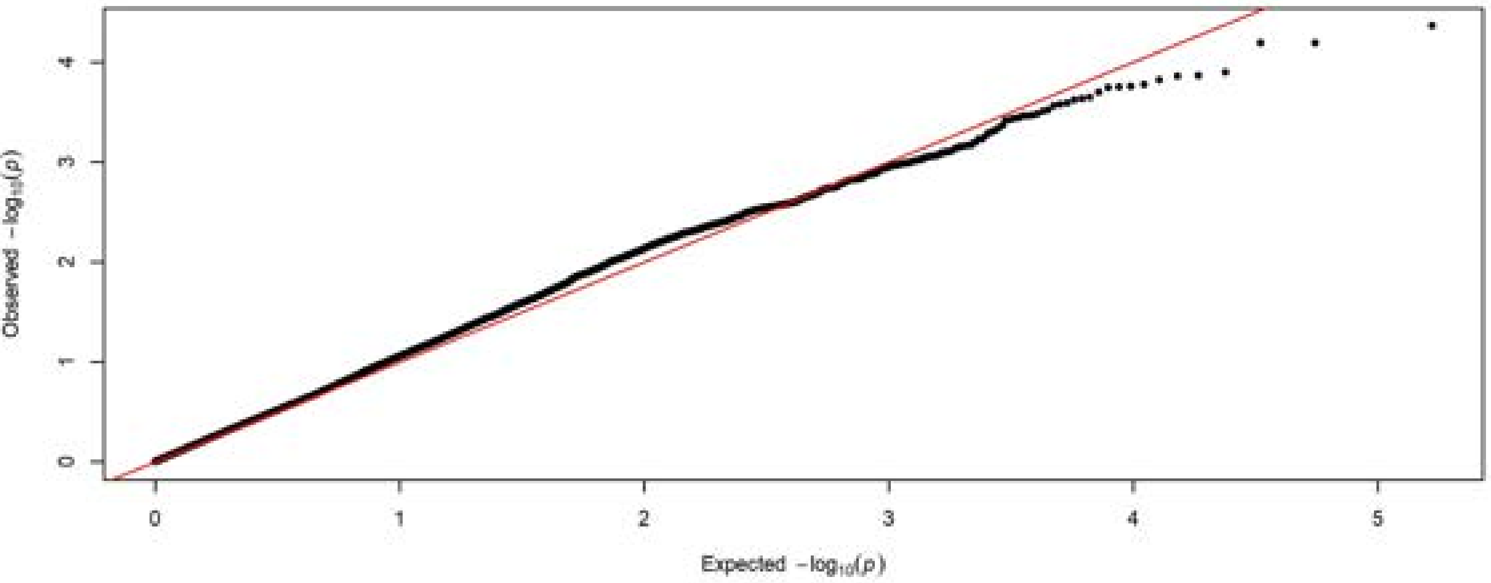

Q-Q Plot Magazaine Entries Day 4 - Meta-analysis of 7 Subgroups - 64k SNPs (n=3936)

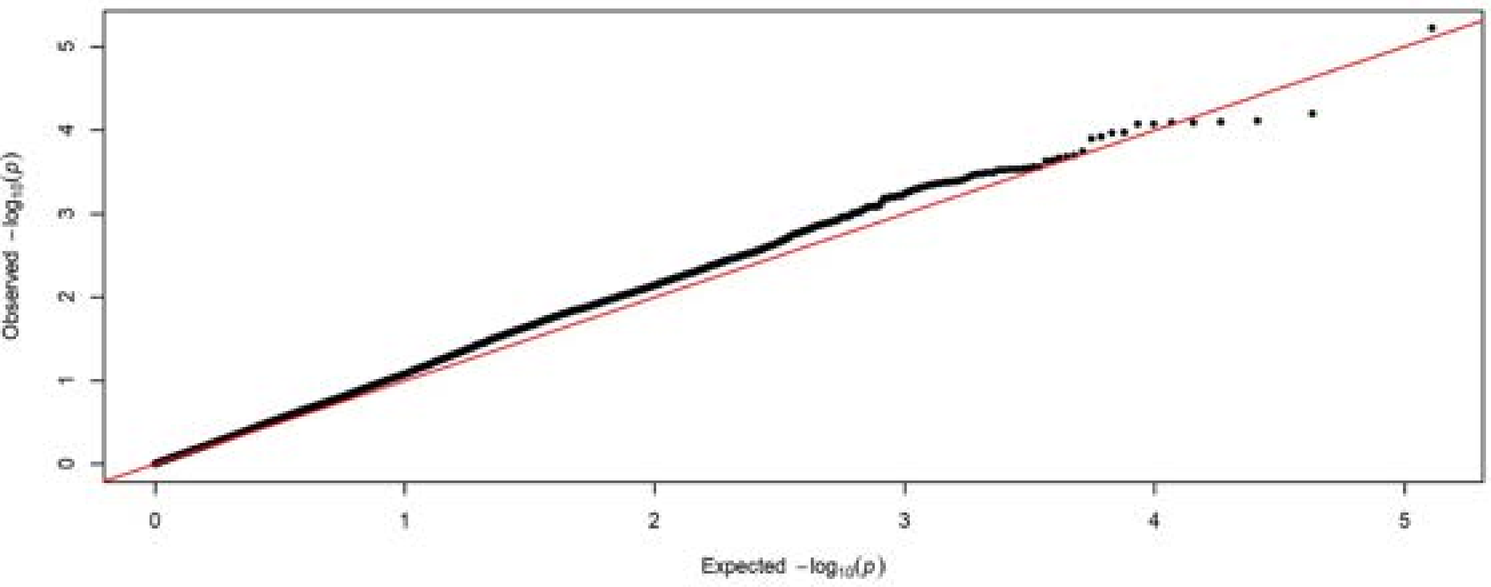

Q-Q Plot Magazaine Entries Day 4 - Charles River 4 Subgroups - 198k SNPs (n=1728)

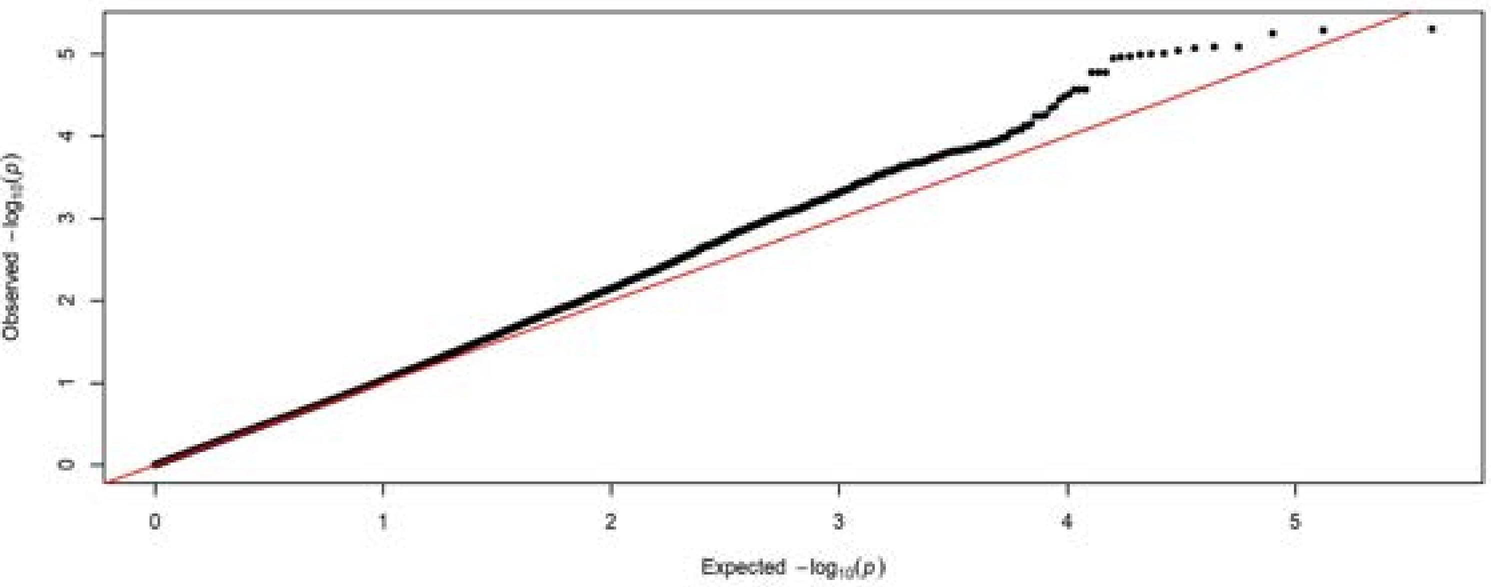

Q-Q Plot Magazaine Entries Day 4 - Harlan 3 Subgroups - 83k SNPs (n=2208)

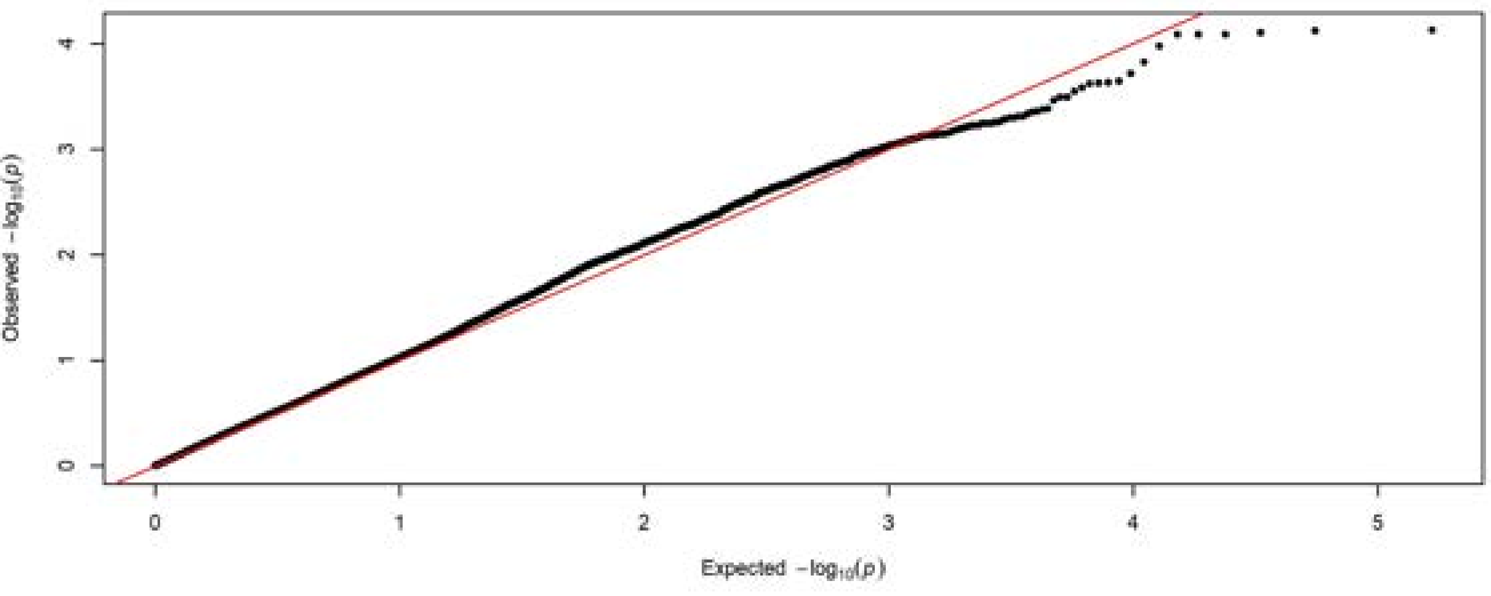

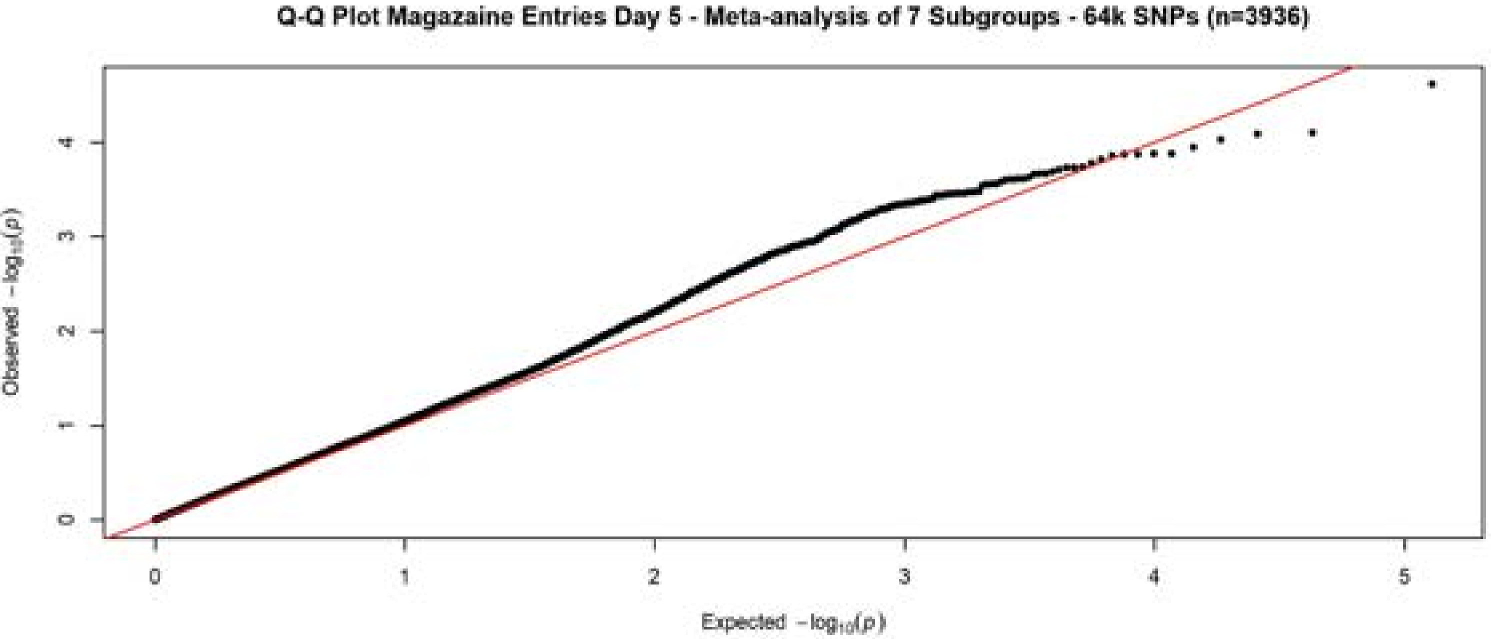
Q-Q Plot Magazaine Entries Day 5 - Meta-analysis of 7 Subgroups - 64k SNPs (n=3936)
Observed $-\log_{10}(p)$
Expected $-\log_{10}(p)$

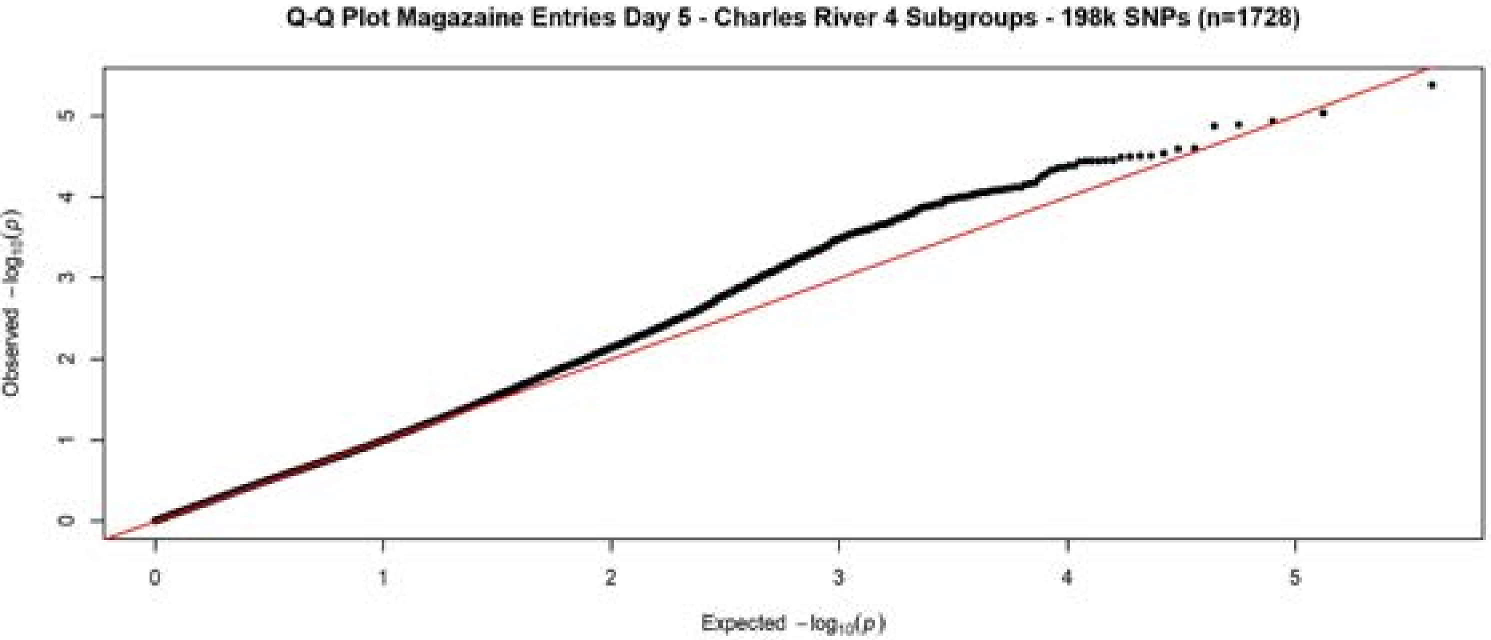
Q-Q Plot Magazaine Entries Day 5 - Charles River 4 Subgroups - 198k SNPs (n=1728)
Observed $-\log_{10}(p)$
Expected $-\log_{10}(p)$

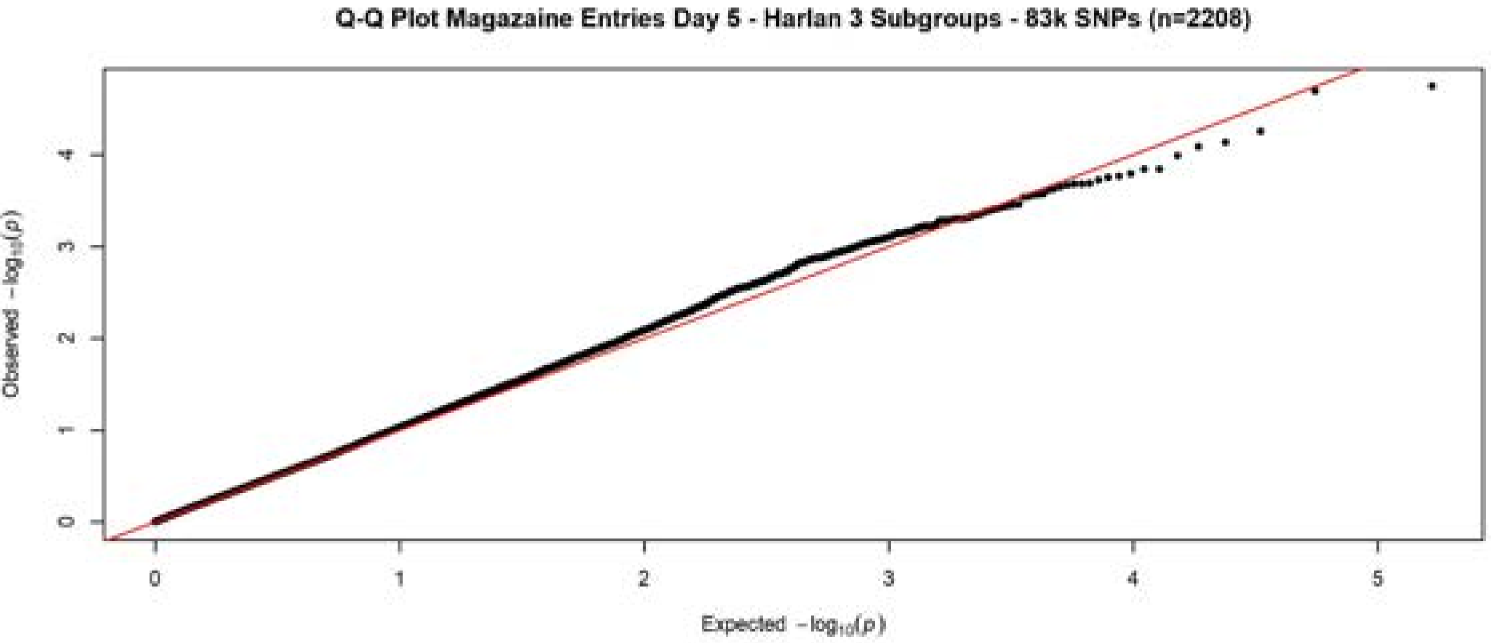
Q-Q Plot Magazaine Entries Day 5 - Harlan 3 Subgroups - 83k SNPs (n=2208)
Observed $-\log_{10}(p)$
Expected $-\log_{10}(p)$

Q-Q Plot Magazine Entries NCS Day 1 - Meta-analysis of 7 Subgroups - 64k SNPs (n=3933)

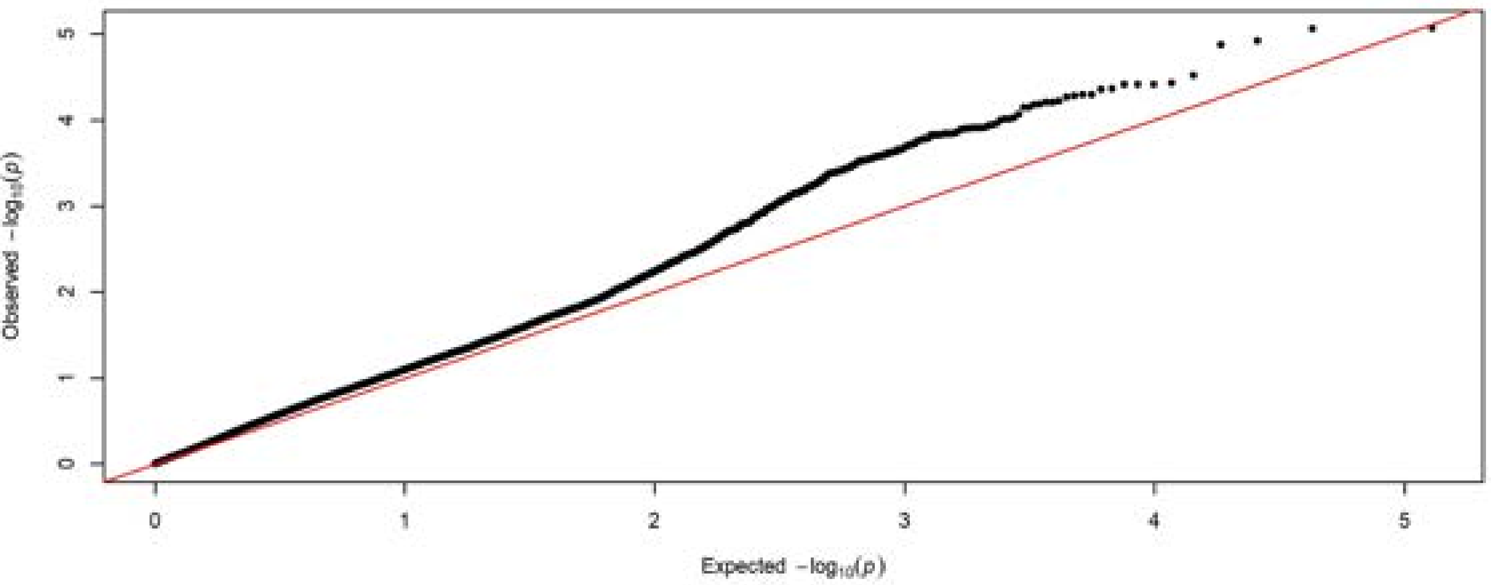

Q-Q Plot Magazine Entries NCS Day 1 - Charles River 4 Subgroups - 198k SNPs (n=1728)

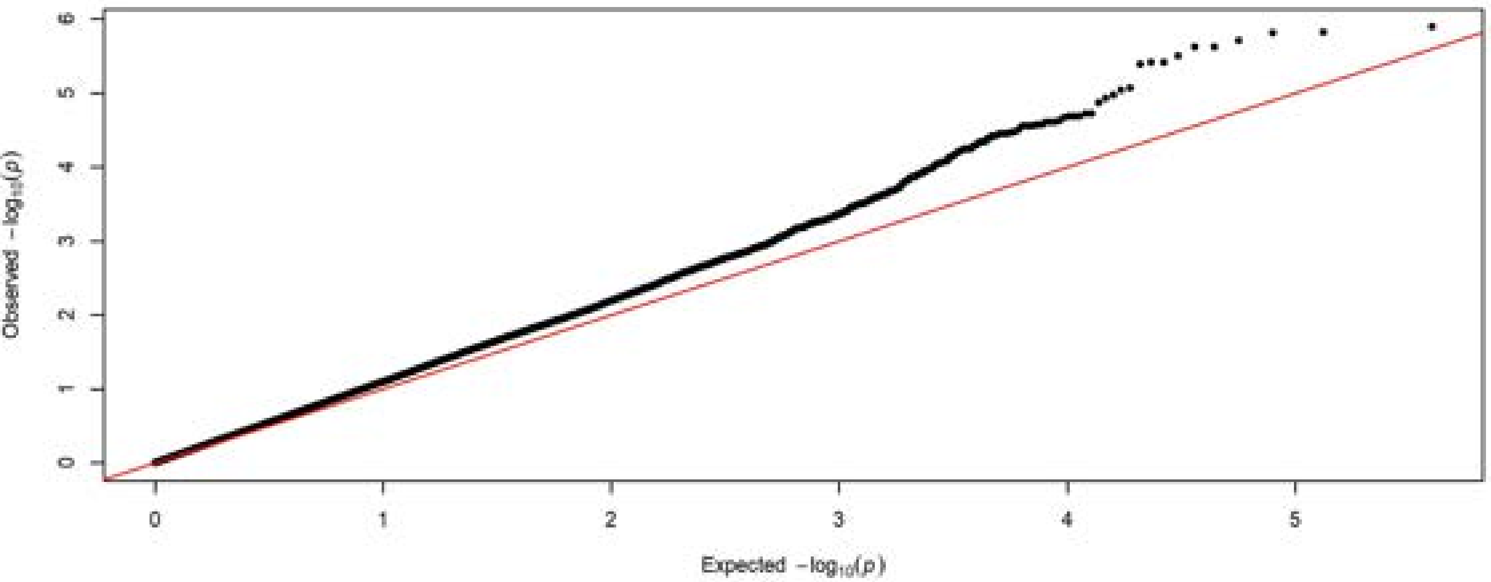

Q-Q Plot Magazine Entries NCS Day 1 - Harlan 3 Subgroups - 83k SNPs (n=2205)

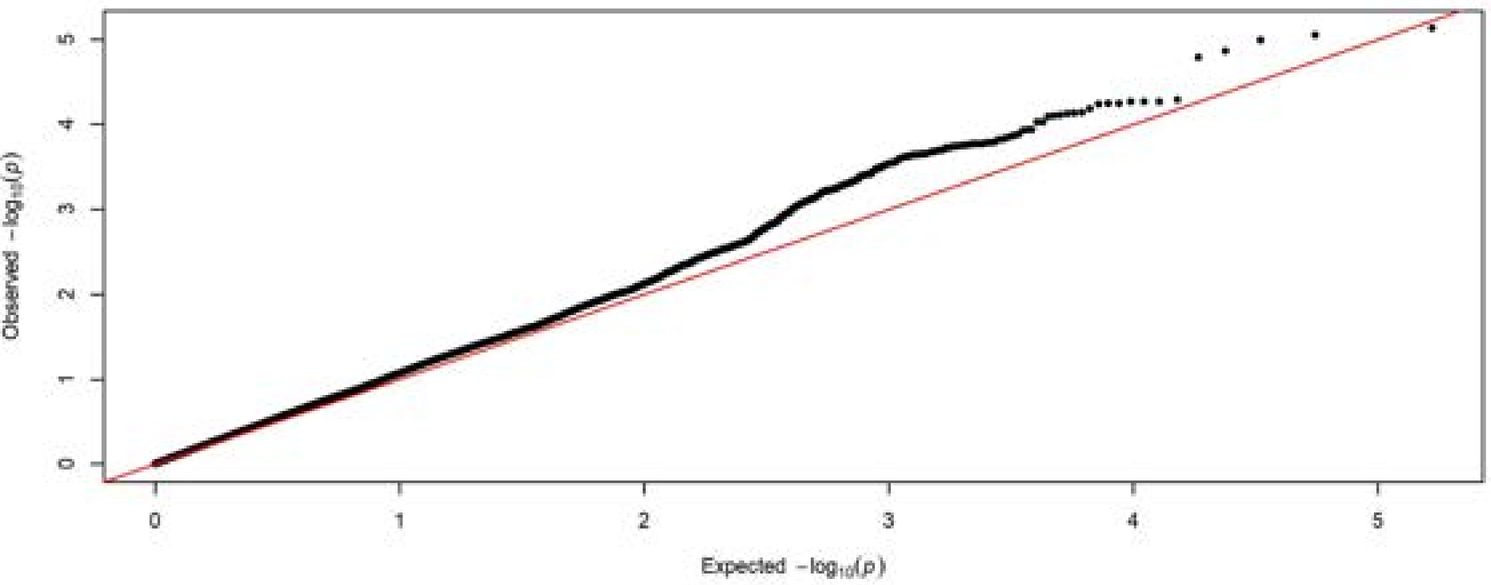

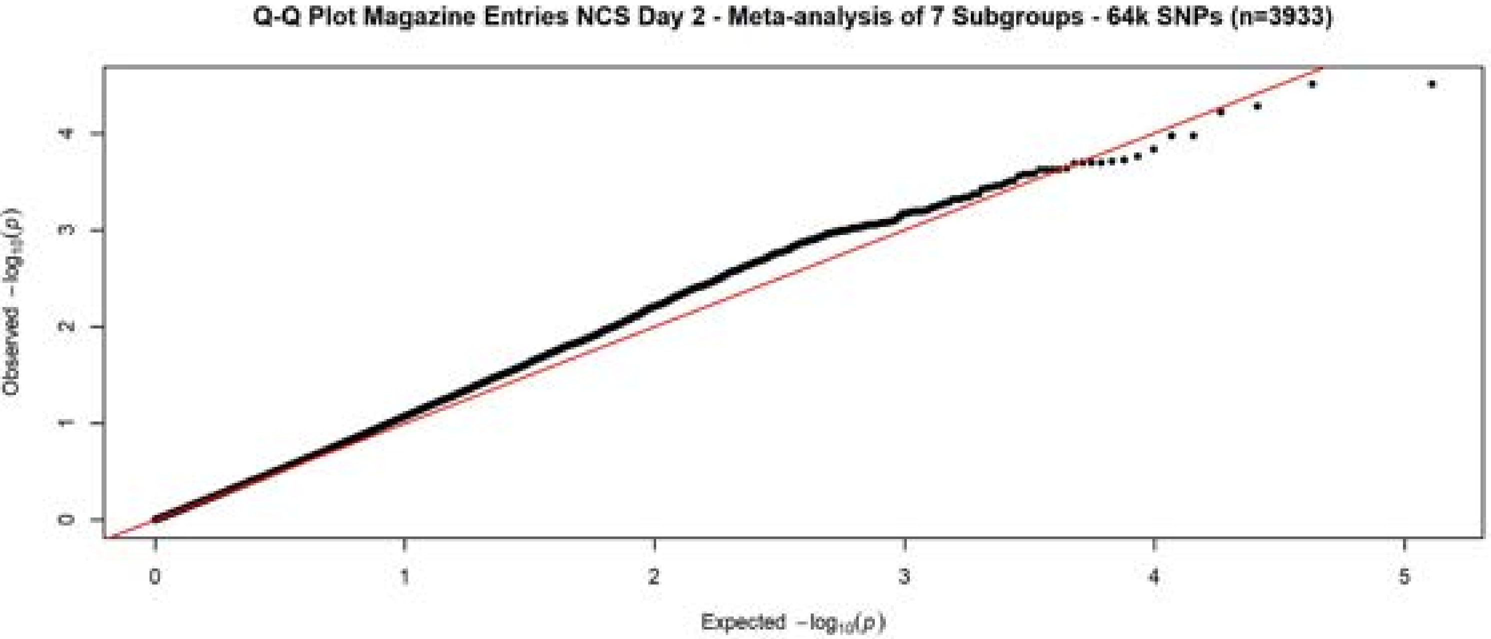
Q-Q Plot Magazine Entries NCS Day 2 - Meta-analysis of 7 Subgroups - 64k SNPs (n=3933)
Observed −log10(p)
Expected −log10(p)

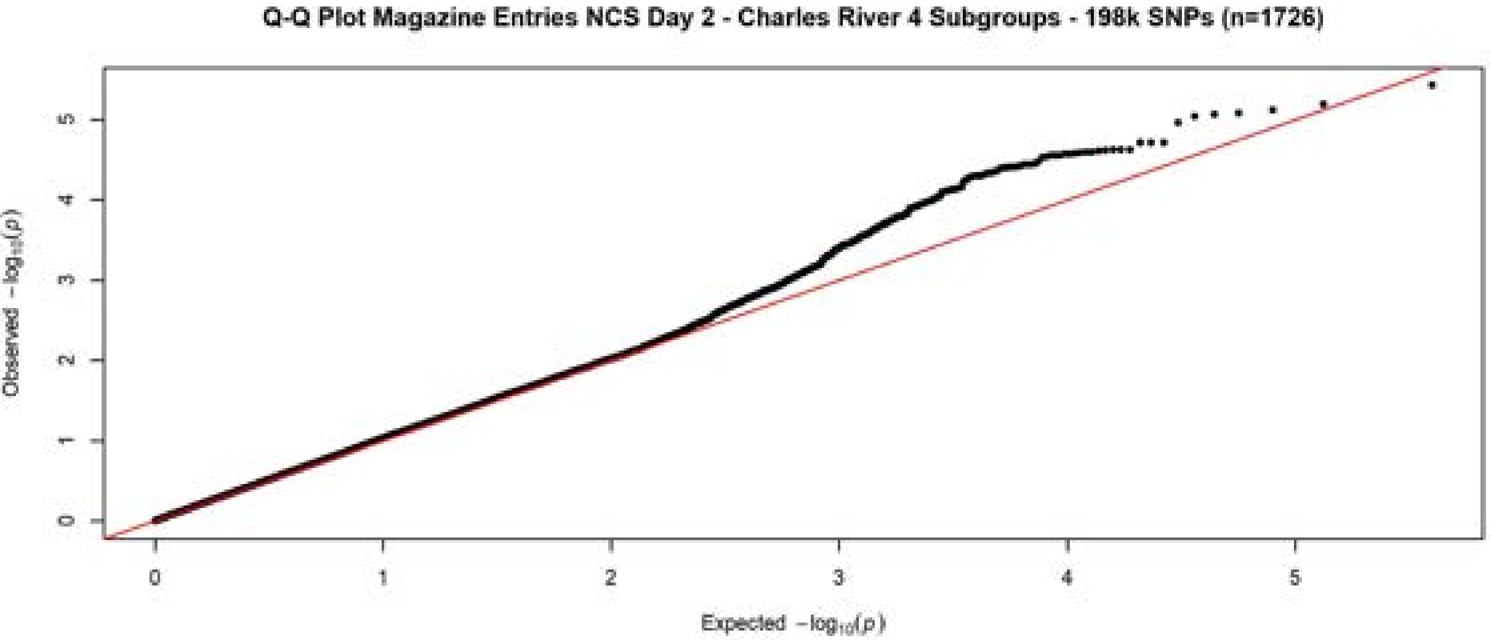
Q-Q Plot Magazine Entries NCS Day 2 - Charles River 4 Subgroups - 198k SNPs (n=1726)
Observed −log10(p)
Expected −log10(p)

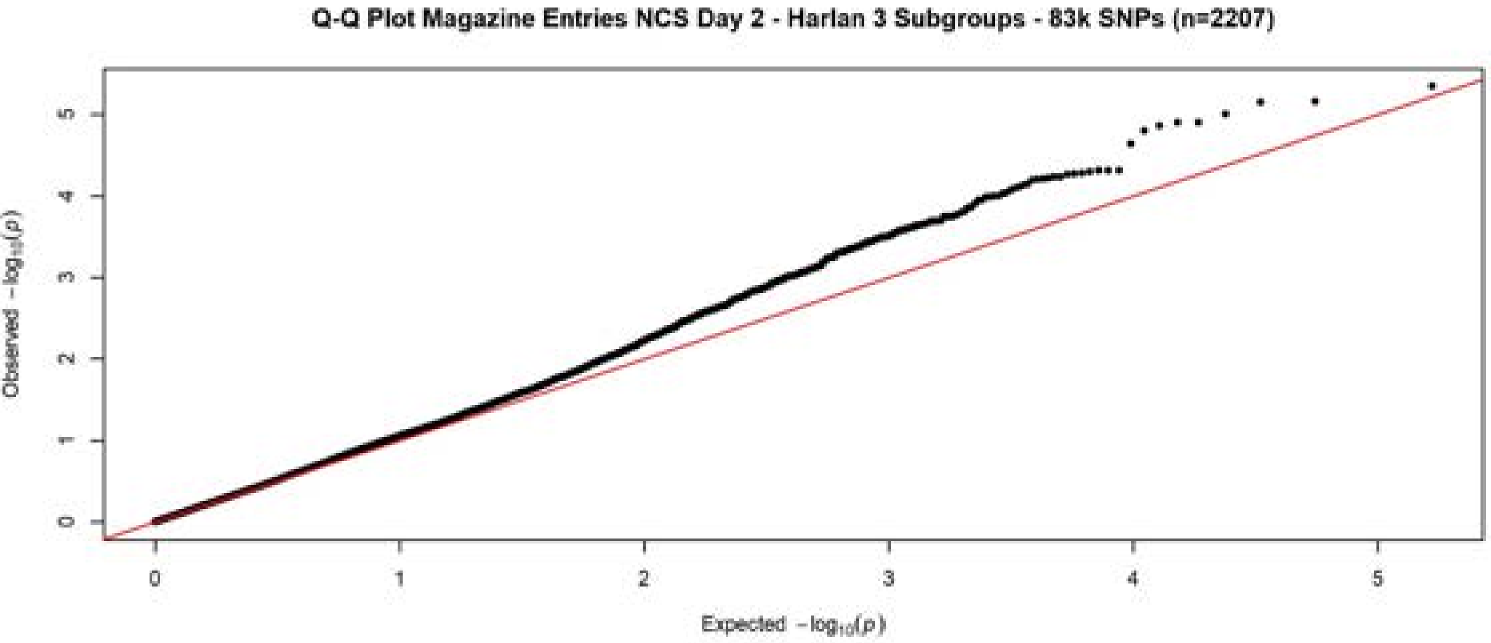
Q-Q Plot Magazine Entries NCS Day 2 - Harlan 3 Subgroups - 83k SNPs (n=2207)
Observed −log10(p)
Expected −log10(p)

Q-Q Plot Magazine Entries NCS Day 3 - Meta-analysis of 7 Subgroups - 64k SNPs (n=3931)

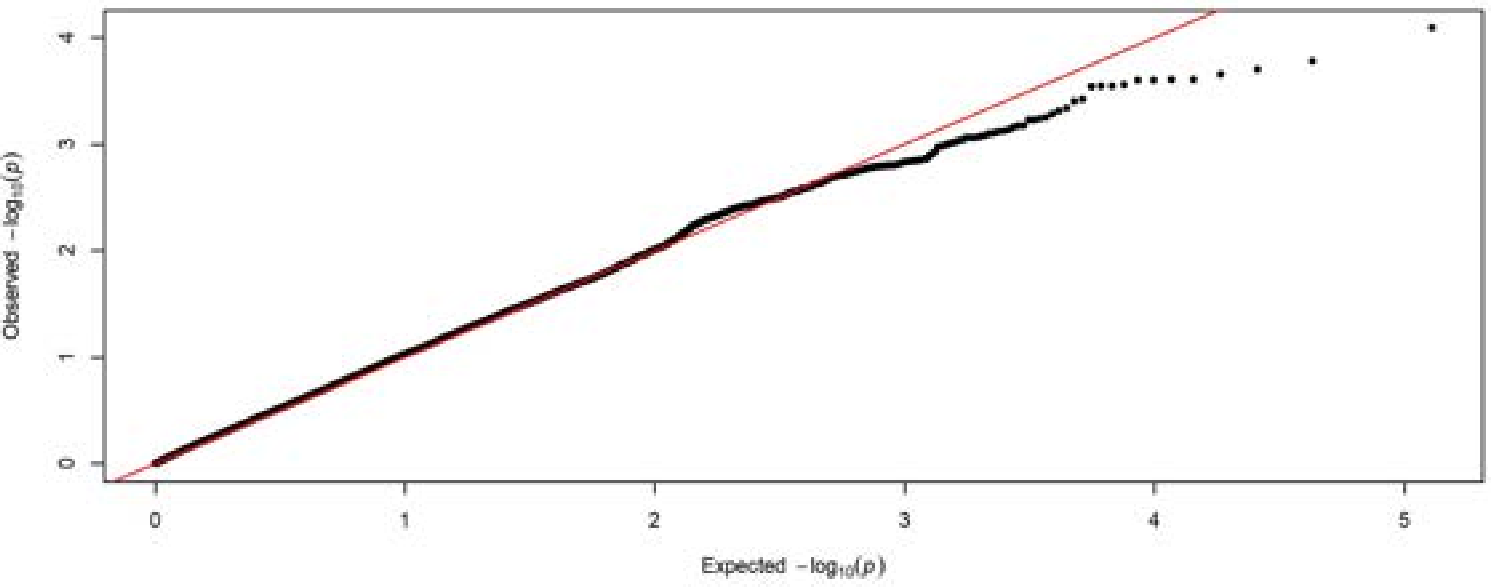

Q-Q Plot Magazine Entries NCS Day 3 - Charles River 4 Subgroups - 198k SNPs (n=1727)

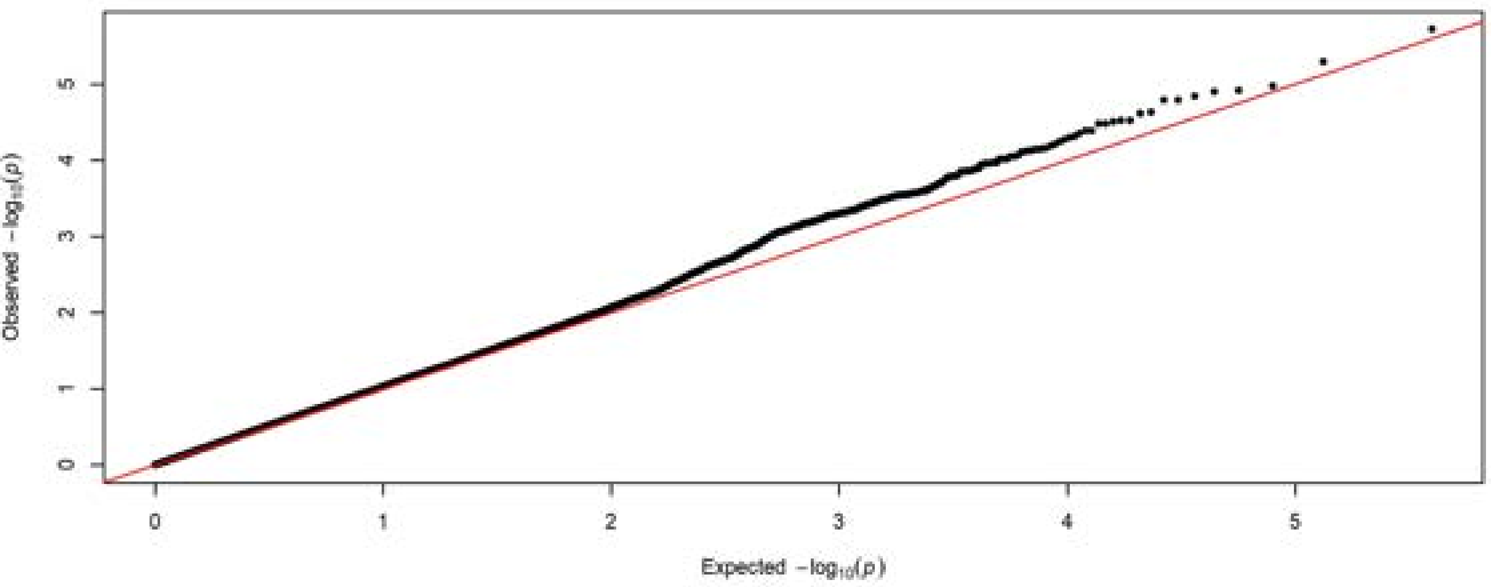

Q-Q Plot Magazine Entries NCS Day 3 - Harlan 3 Subgroups - 83k SNPs (n=2204)

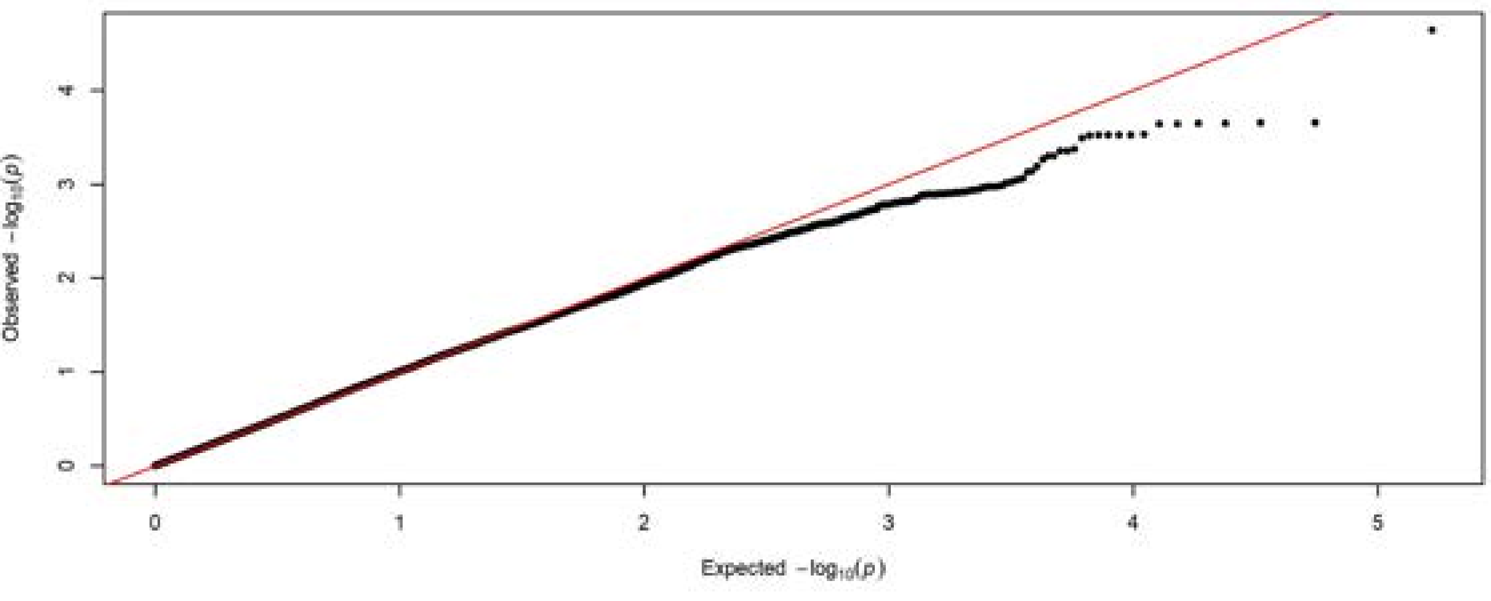

Q-Q Plot Magazine Entries NCS Day 4 - Meta-analysis of 7 Subgroups - 64k SNPs (n=3936)

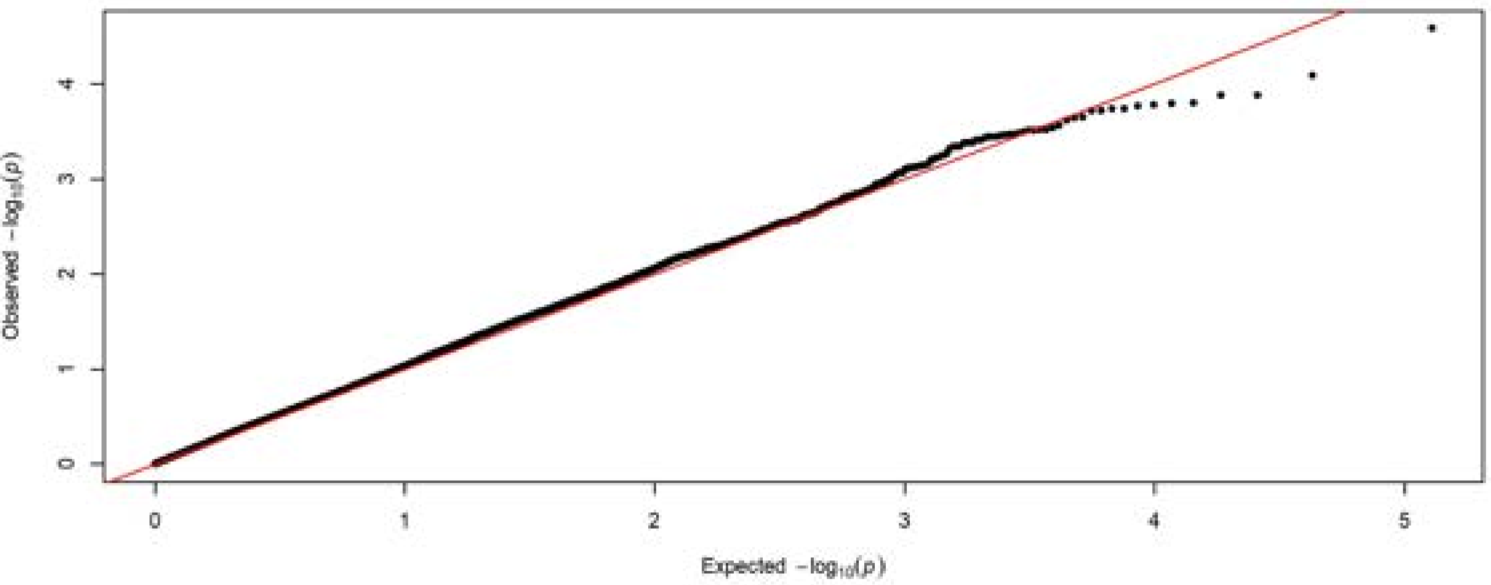

Q-Q Plot Magazine Entries NCS Day 4 - Charles River 4 Subgroups - 198k SNPs (n=1728)

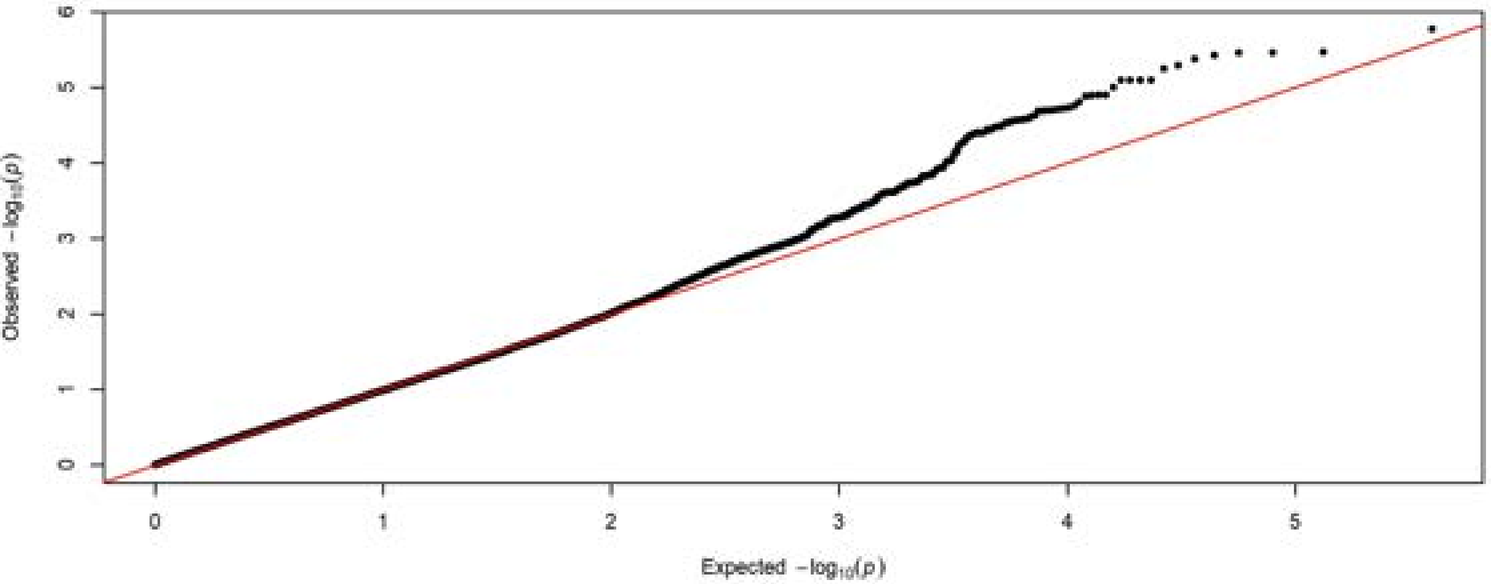

Q-Q Plot Magazine Entries NCS Day 4 - Harlan 3 Subgroups - 83k SNPs (n=2208)

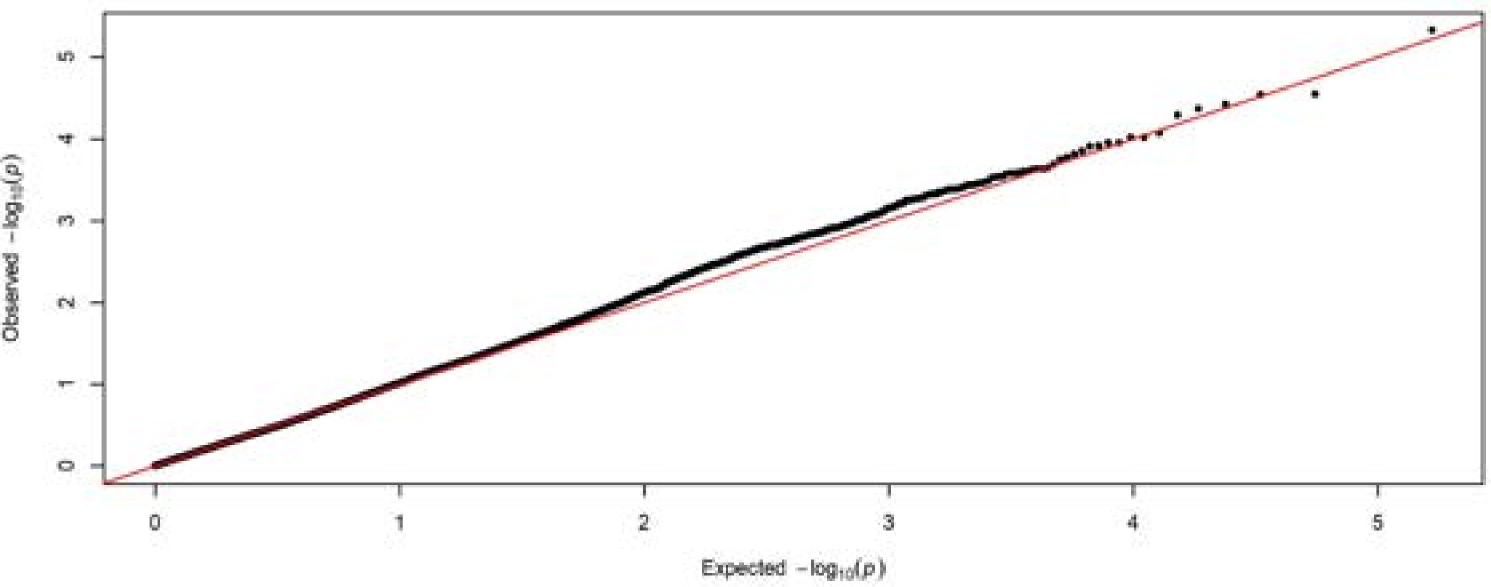

Q-Q Plot Magazine Entries NCS Day 5 - Meta-analysis of 7 Subgroups - 64k SNPs (n=3936)

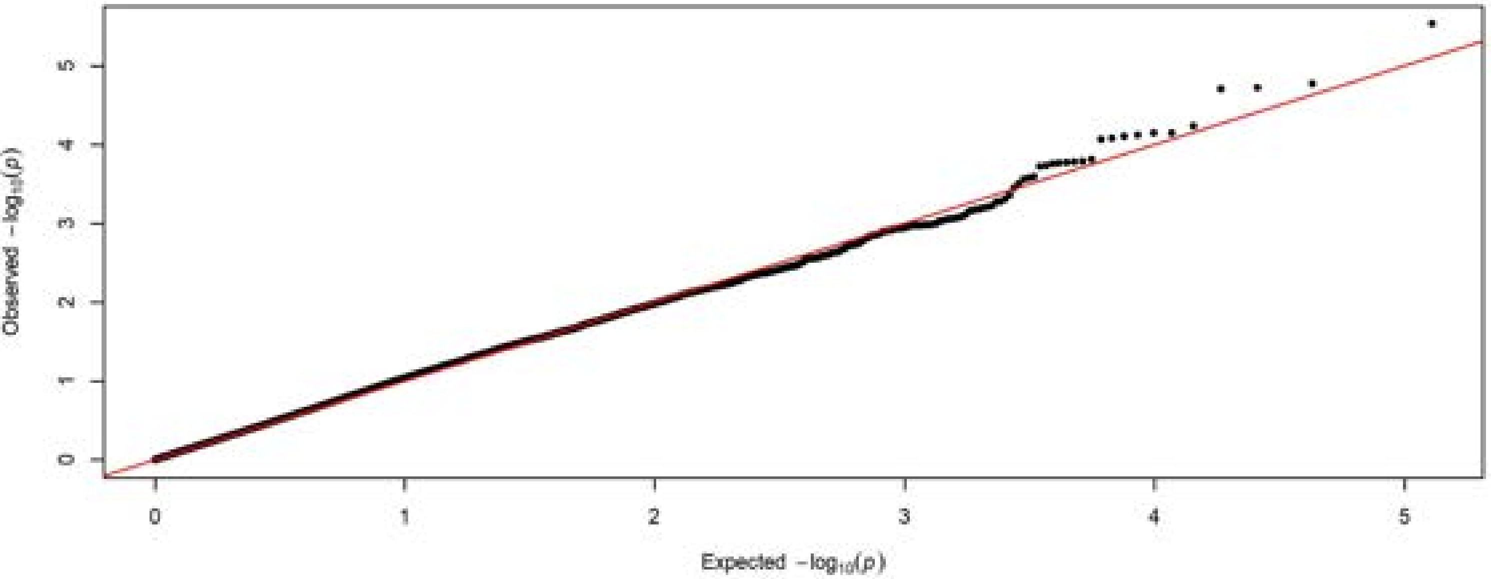

Q-Q Plot Magazine Entries NCS Day 5 - Charles River 4 Subgroups - 198k SNPs (n=1728)

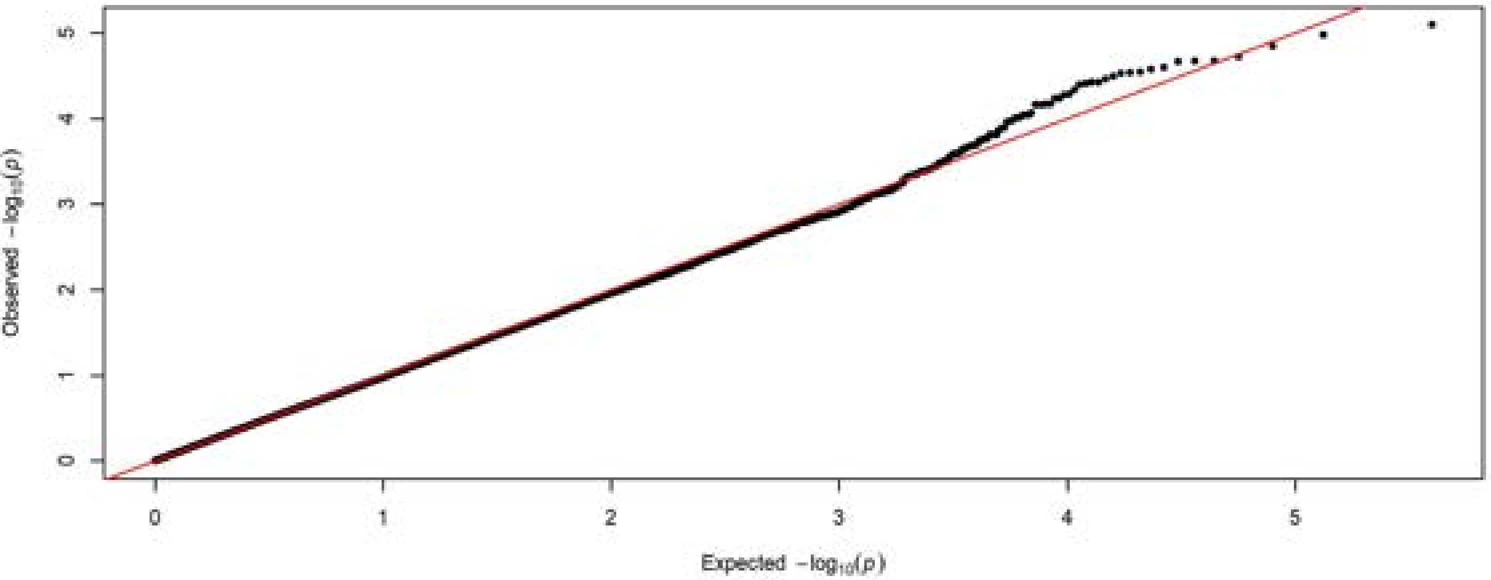

Q-Q Plot Magazine Entries NCS Day 5 - Harlan 3 Subgroups - 83k SNPs (n=2208)

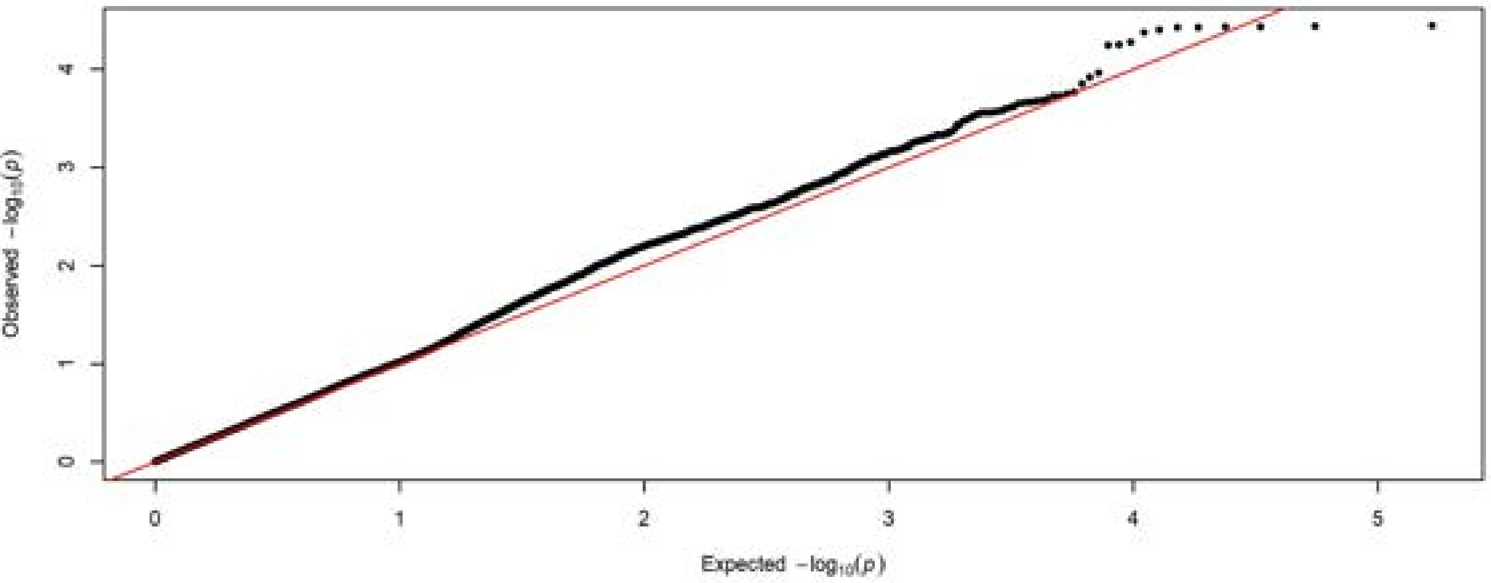

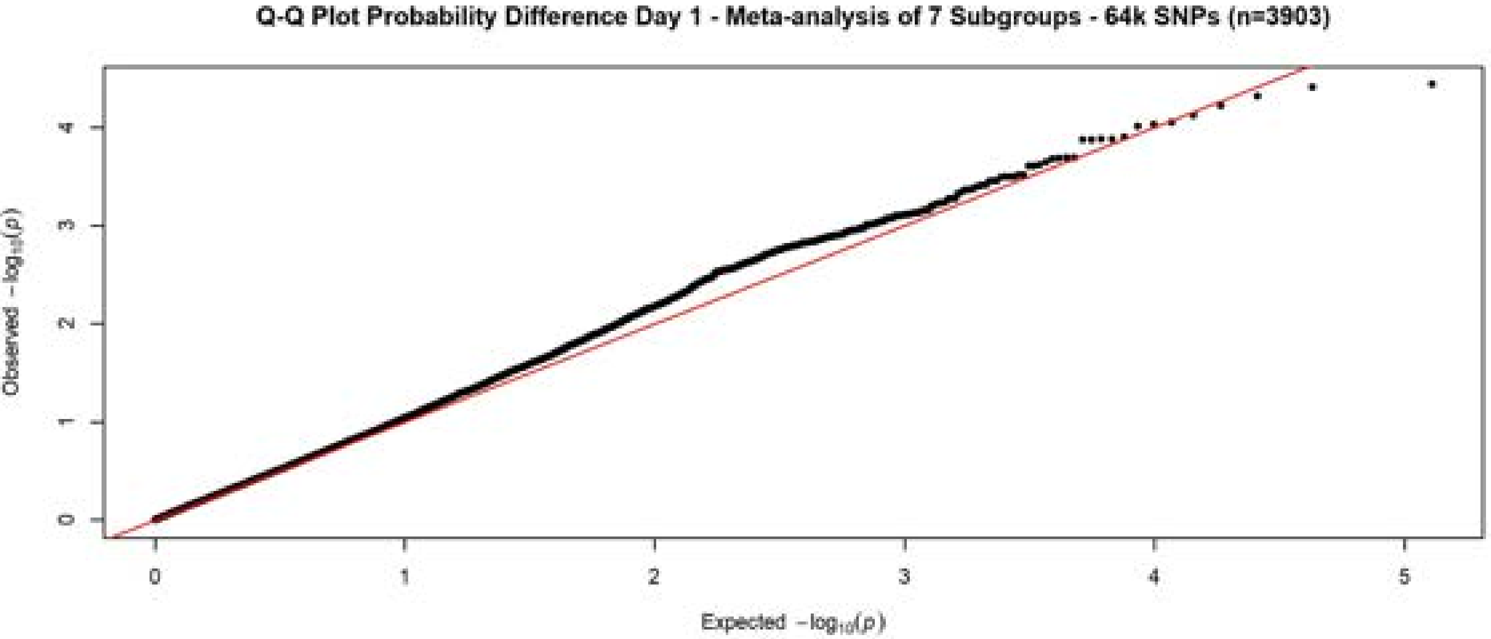
Q-Q Plot Probability Difference Day 1 - Meta-analysis of 7 Subgroups - 64k SNPs (n=3903)
Observed −log10(p)
Expected −log10(p)

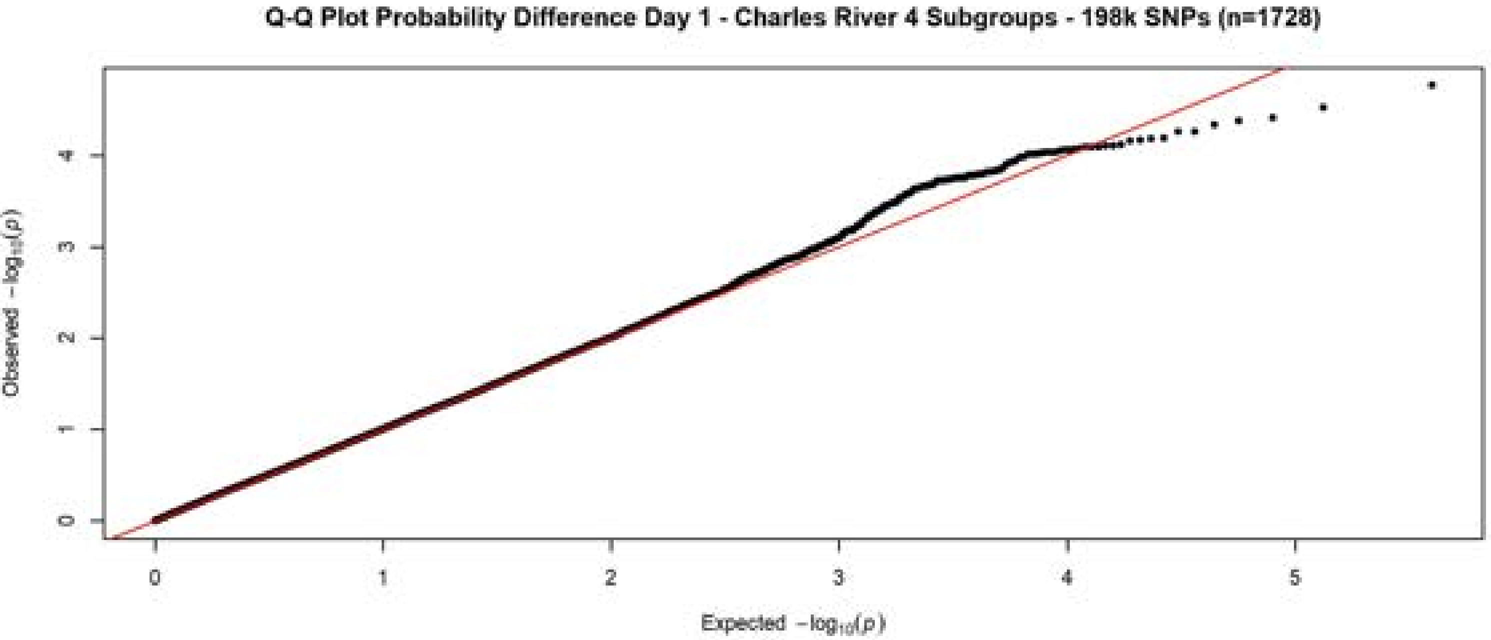
Q-Q Plot Probability Difference Day 1 - Charles River 4 Subgroups - 198k SNPs (n=1728)
Observed −log10(p)
Expected −log10(p)

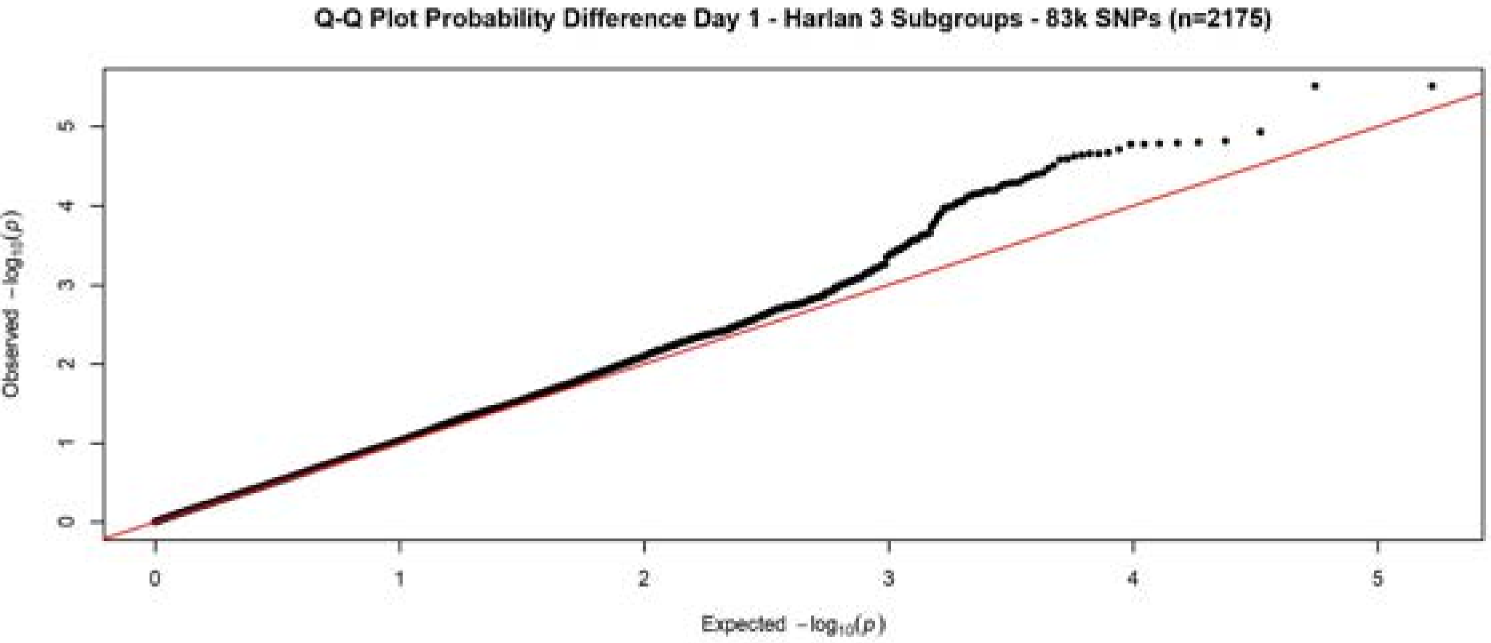
Q-Q Plot Probability Difference Day 1 - Harlan 3 Subgroups - 83k SNPs (n=2175)
Observed −log10(p)
Expected −log10(p)

Q-Q Plot Probability Difference Day 2 - Meta-analysis of 7 Subgroups - 64k SNPs (n=3934)

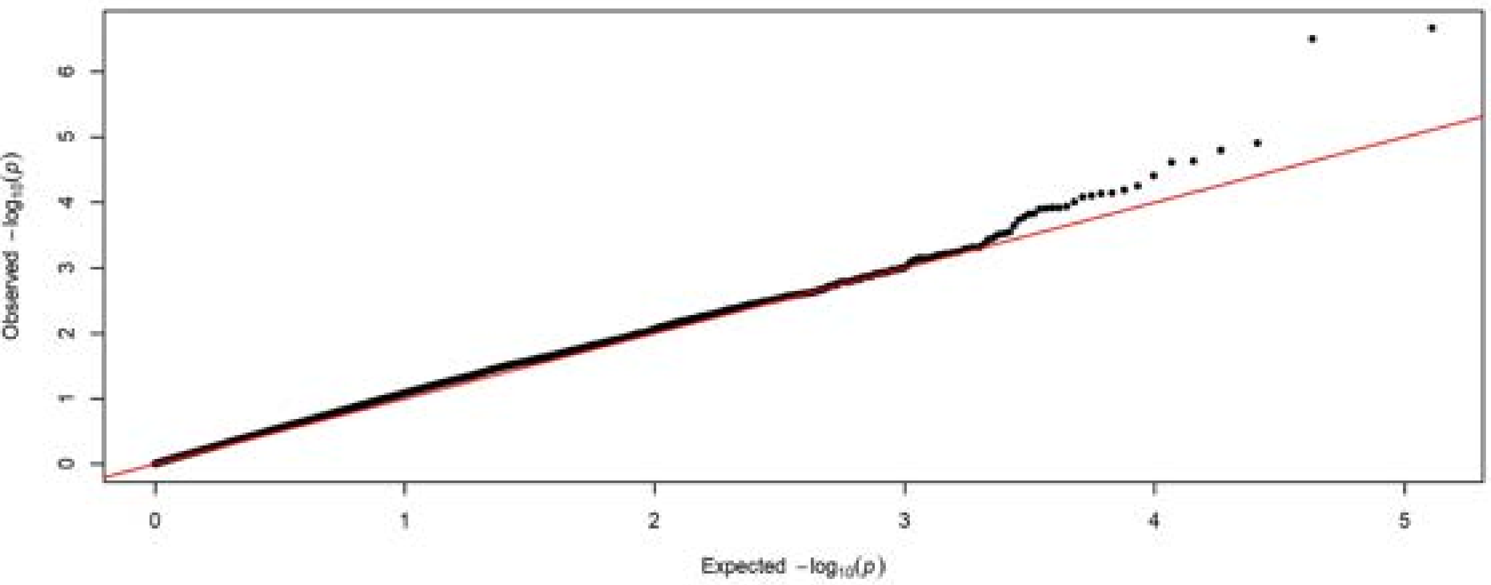

Q-Q Plot Probability Difference Day 2 - Charles River 4 Subgroups - 198k SNPs (n=1726)

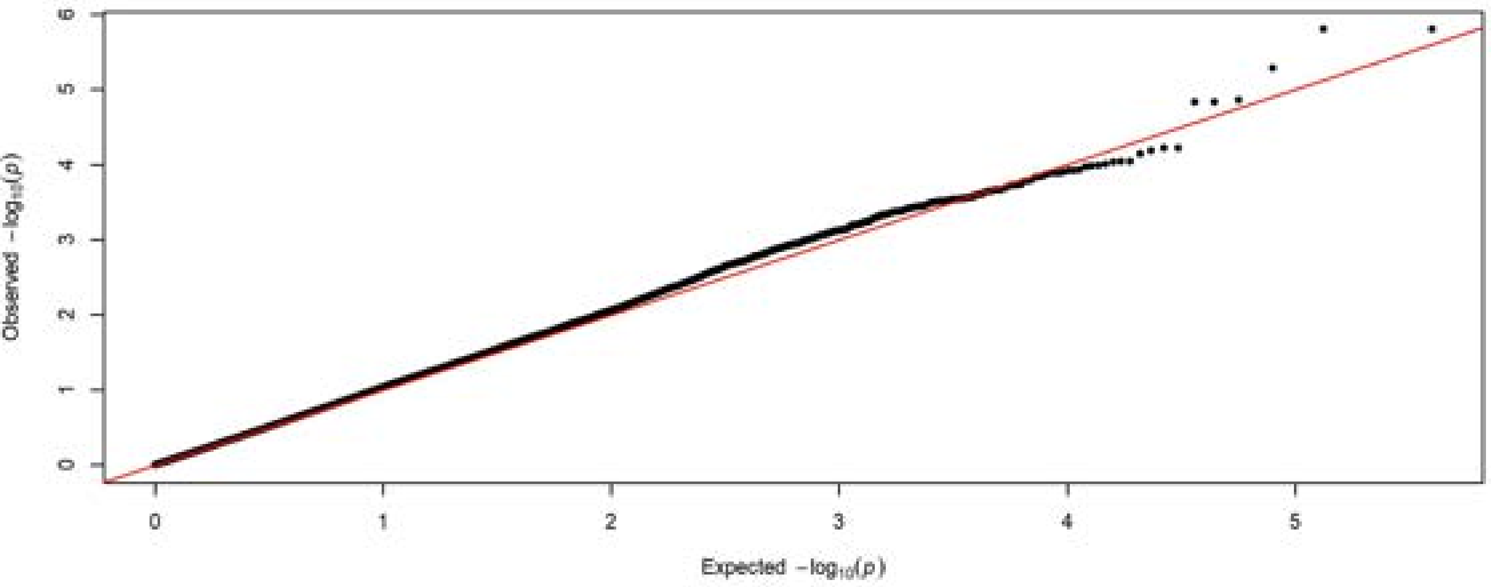

Q-Q Plot Probability Difference Day 2 - Harlan 3 Subgroups - 83k SNPs (n=2208)

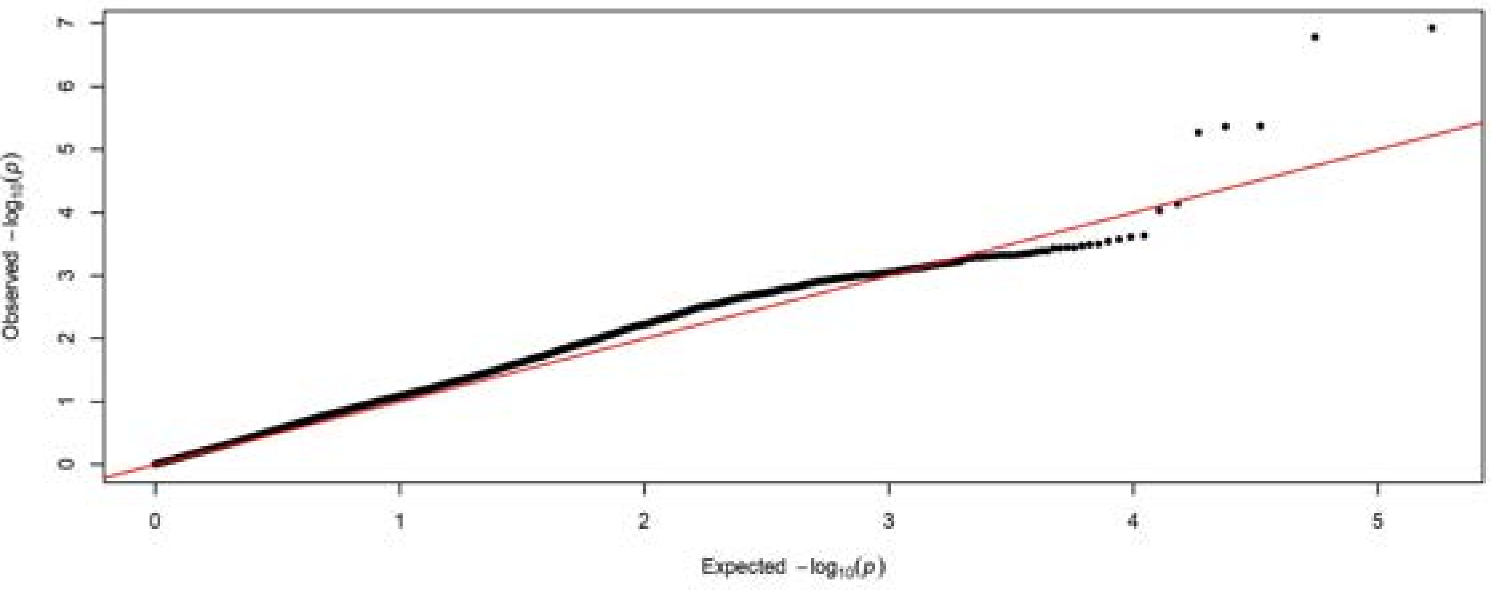

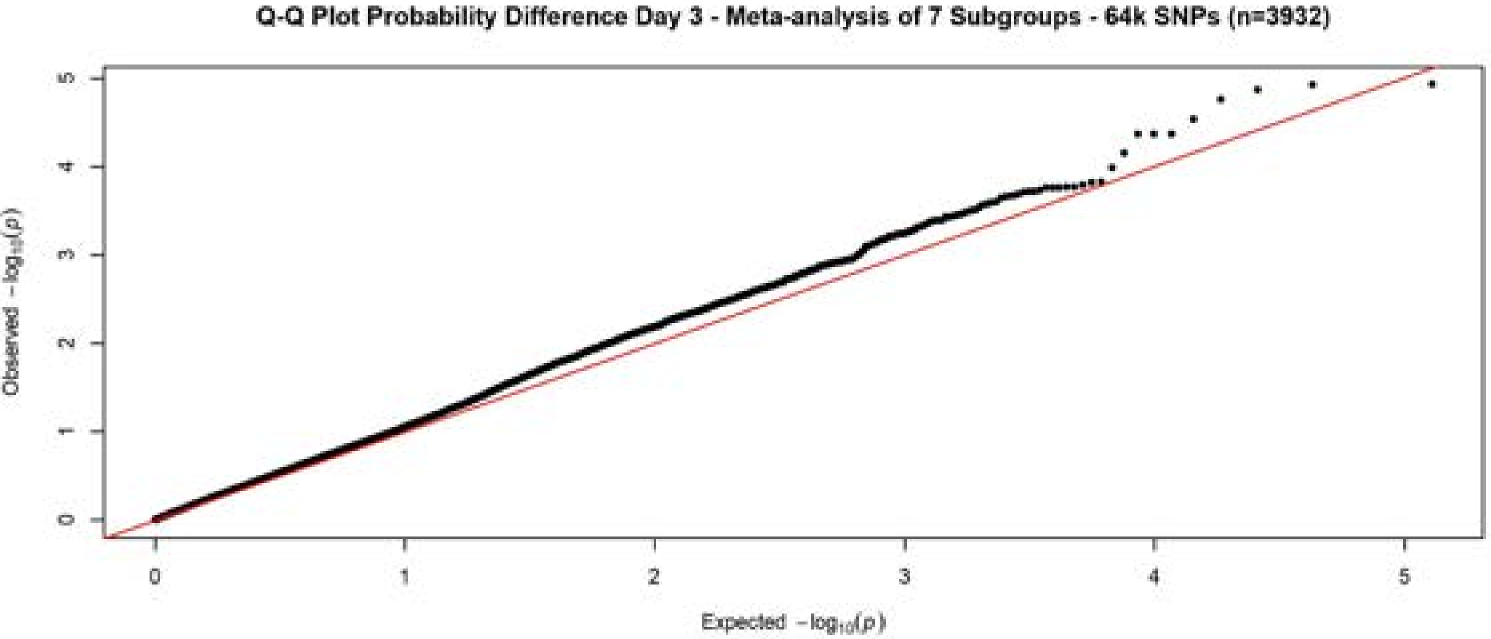
Q-Q Plot Probability Difference Day 3 - Meta-analysis of 7 Subgroups - 64k SNPs (n=3932)
Observed −log10(p)
Expected −log10(p)

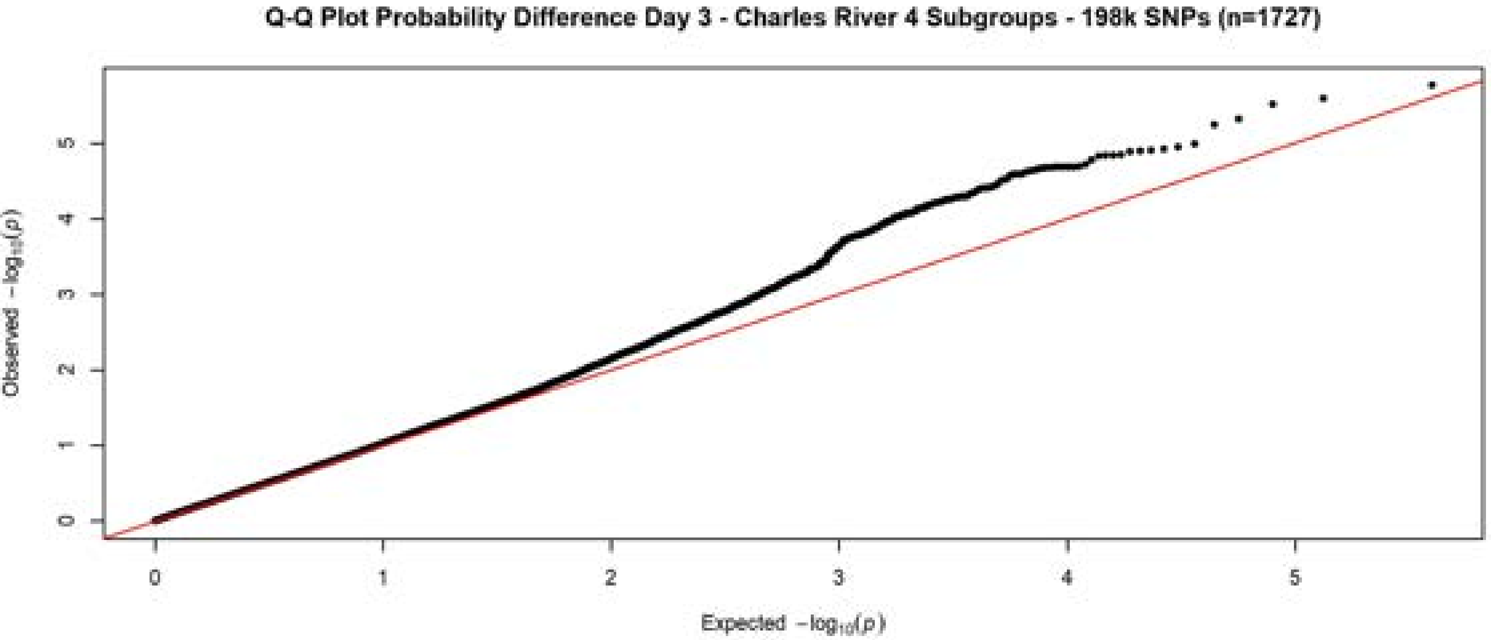
Q-Q Plot Probability Difference Day 3 - Charles River 4 Subgroups - 198k SNPs (n=1727)
Observed −log10(p)
Expected −log10(p)

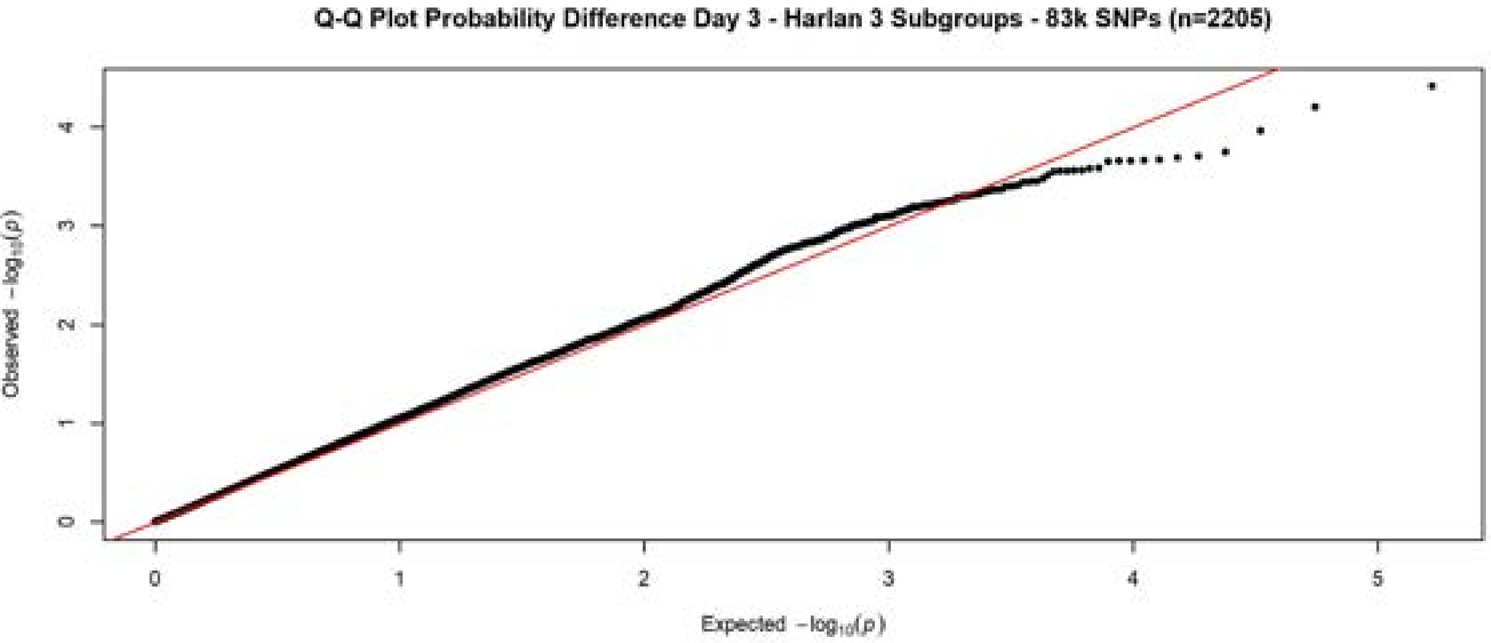
Q-Q Plot Probability Difference Day 3 - Harlan 3 Subgroups - 83k SNPs (n=2205)
Observed −log10(p)
Expected −log10(p)

Q-Q Plot Probability Difference Day 4 - Meta-analysis of 7 Subgroups - 64k SNPs (n=3936)

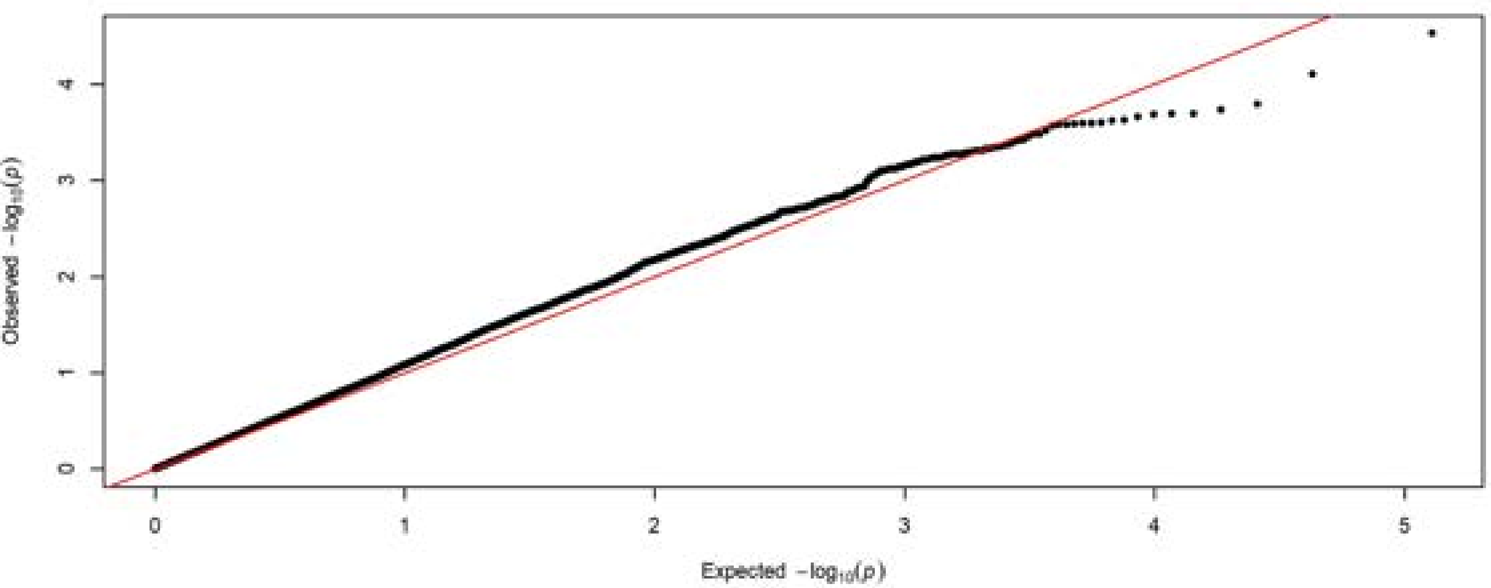

Q-Q Plot Probability Difference Day 4 - Charles River 4 Subgroups - 198k SNPs (n=1728)

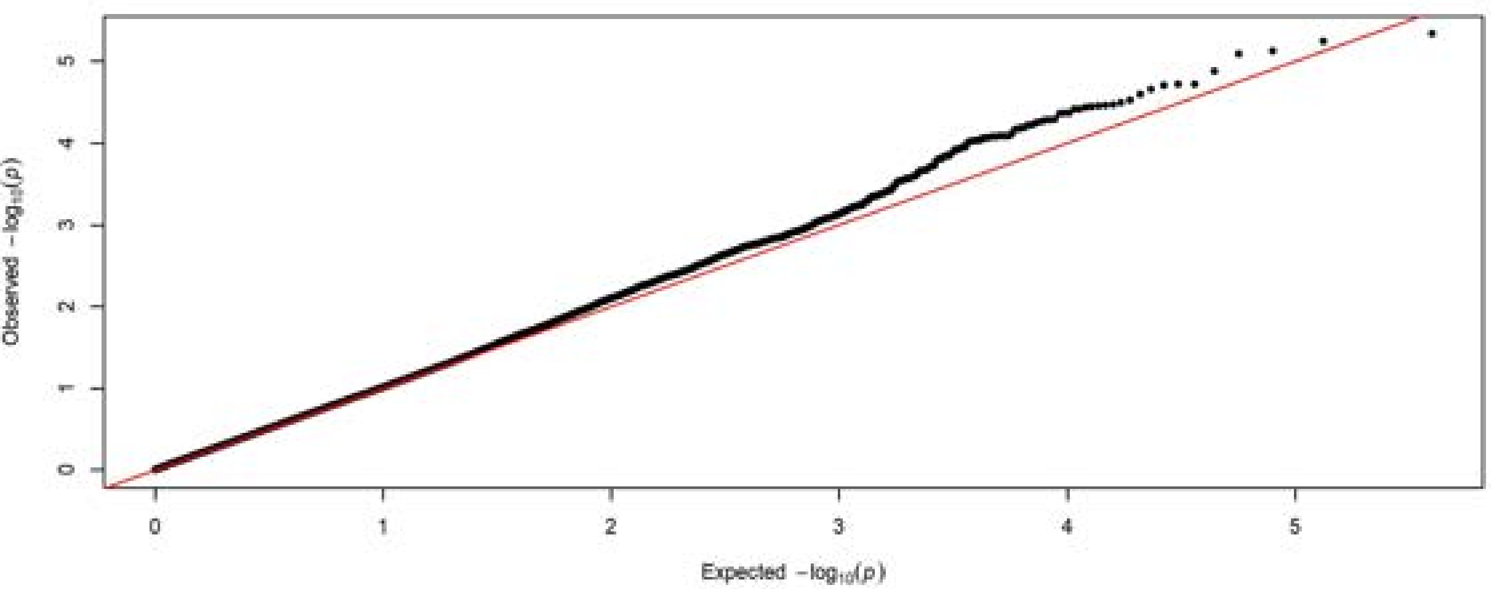

Q-Q Plot Probability Difference Day 4 - Harlan 3 Subgroups - 83k SNPs (n=2208)

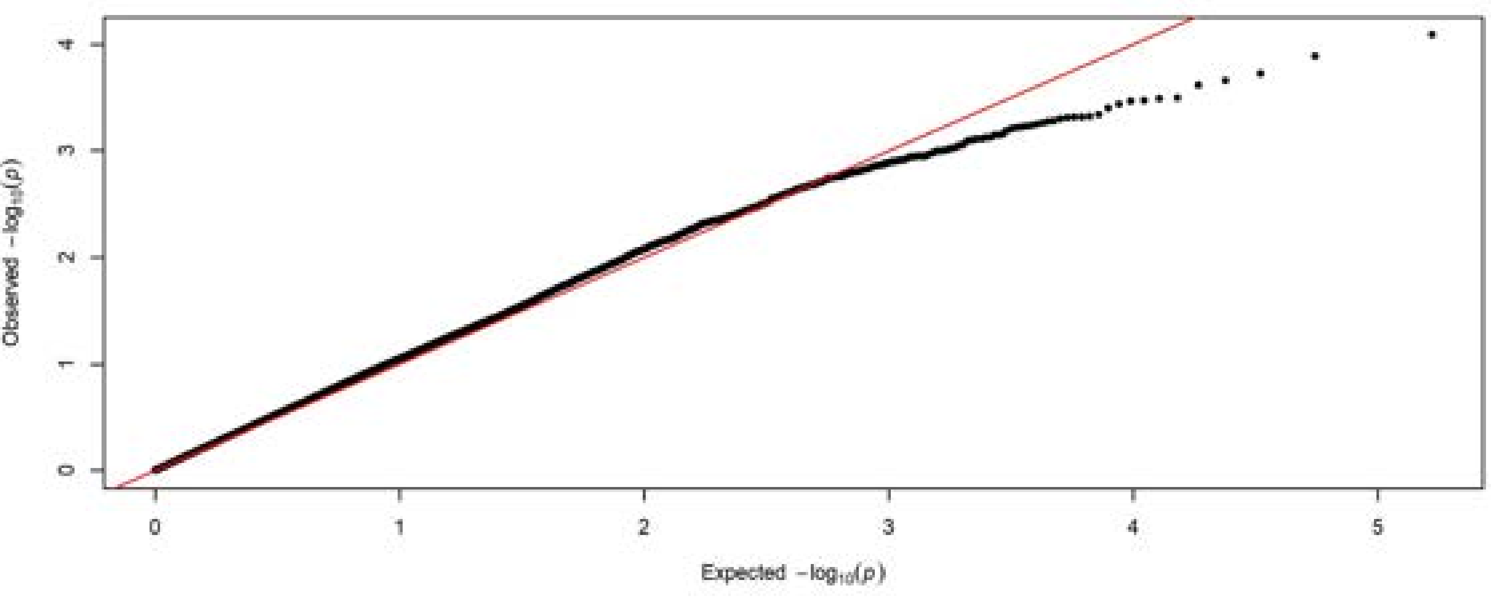

Q-Q Plot Probability Difference Day 5 - Meta-analysis of 7 Subgroups - 64k SNPs (n=3936)

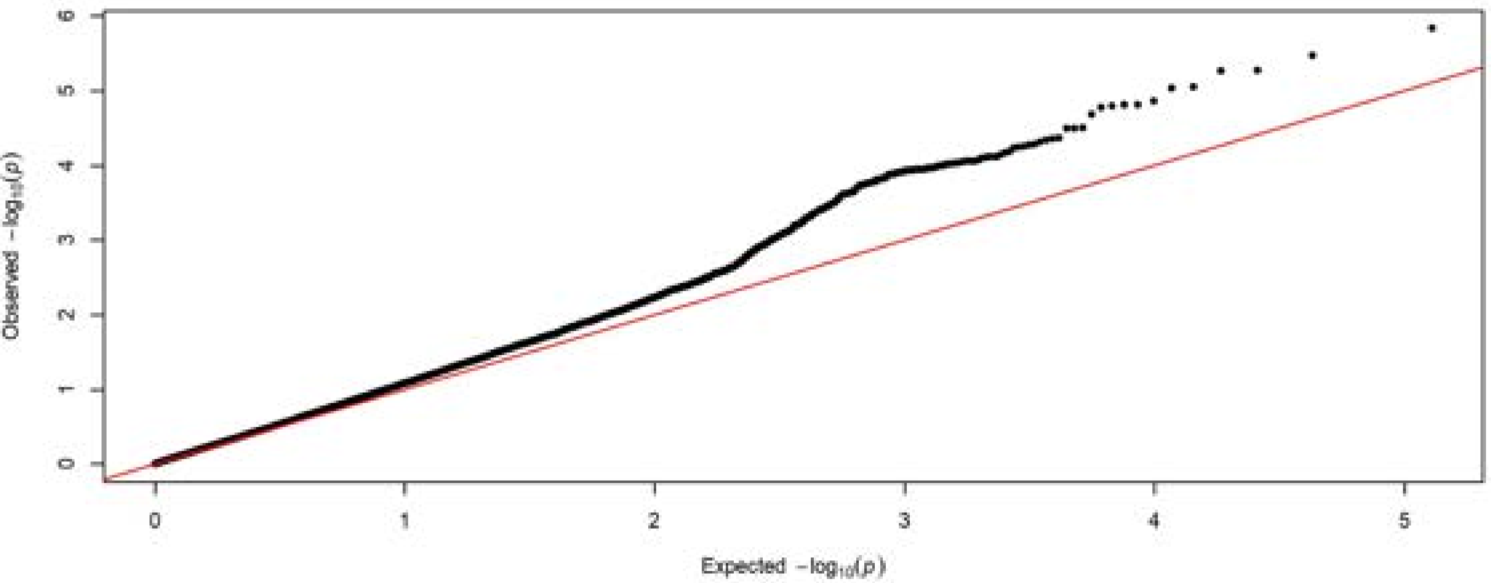

Q-Q Plot Probability Difference Day 5 - Charles River 4 Subgroups - 198k SNPs (n=1728)

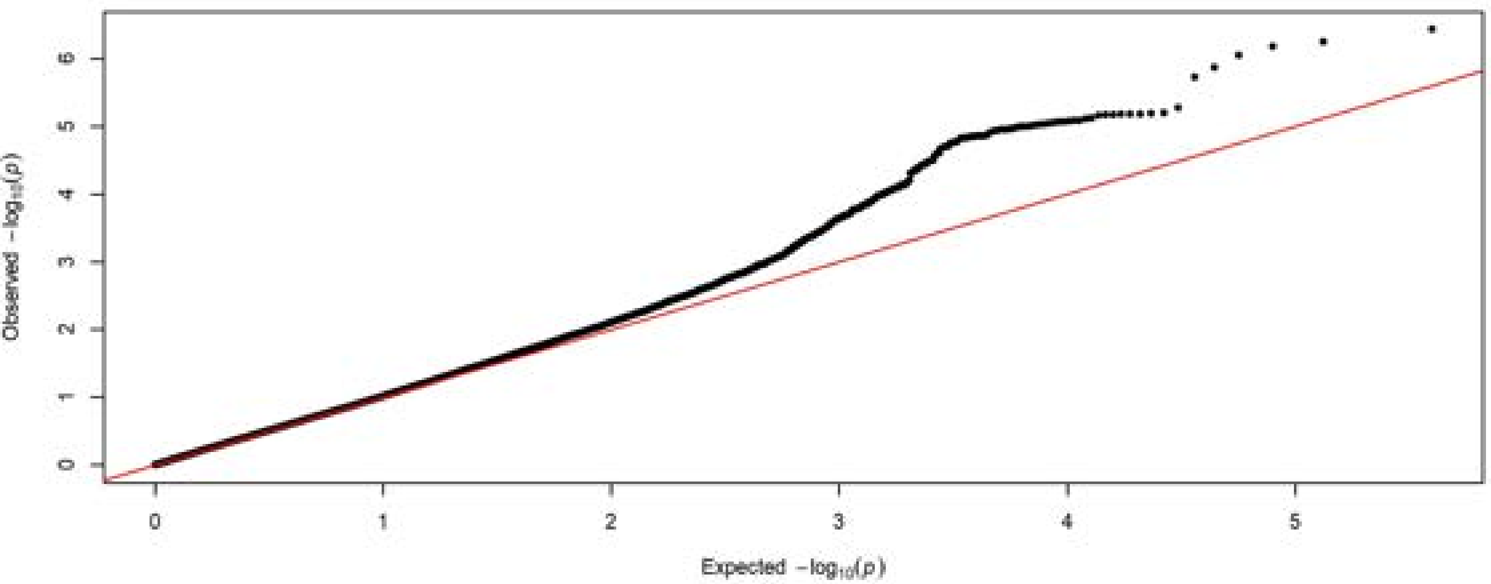

Q-Q Plot Probability Difference Day 5 - Harlan 3 Subgroups - 83k SNPs (n=2208)

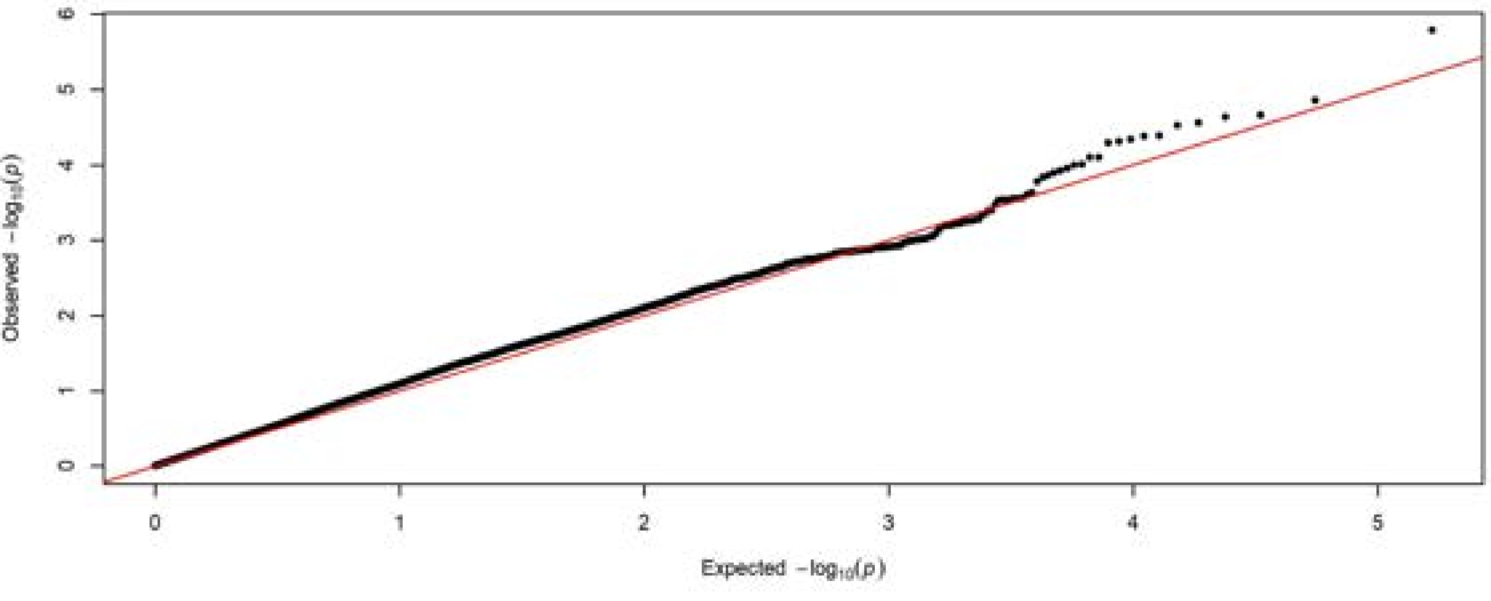

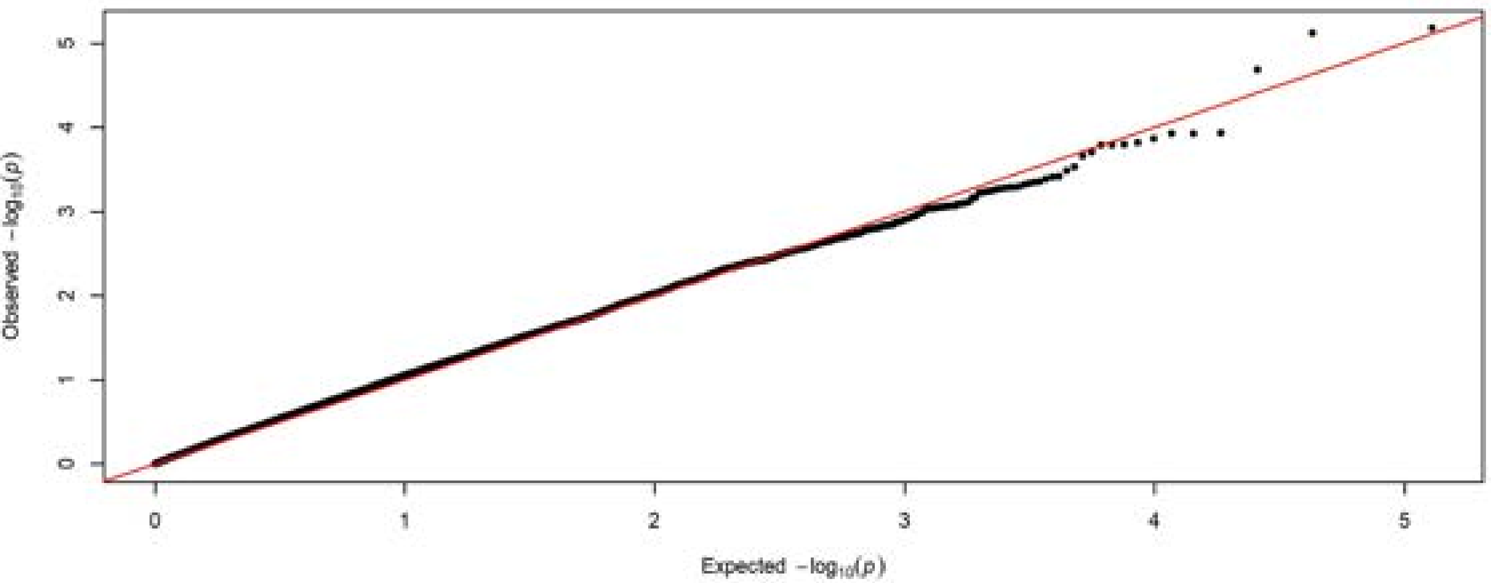

Q-Q Plot Probability of Lever Press Day 1 - Meta-analysis of 7 Subgroups - 64k SNPs (n=3903)

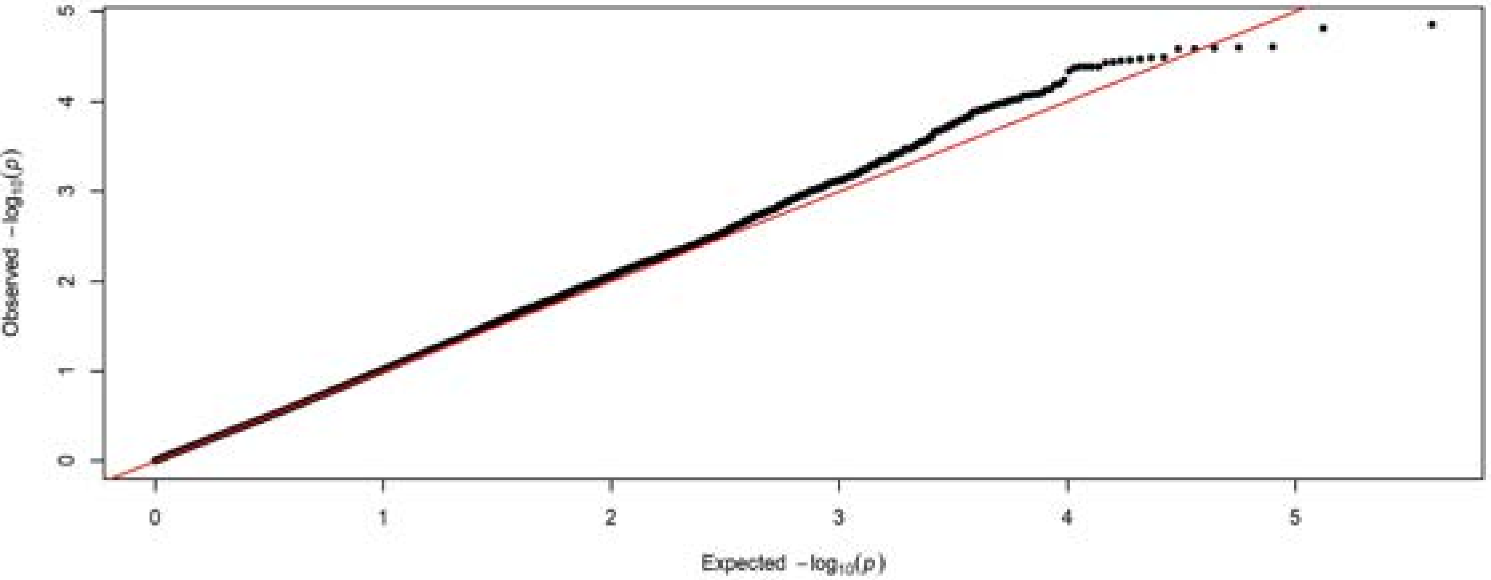

Q-Q Plot Probability of Lever Press Day 1 - Charles River 4 Subgroups - 198k SNPs (n=1728)

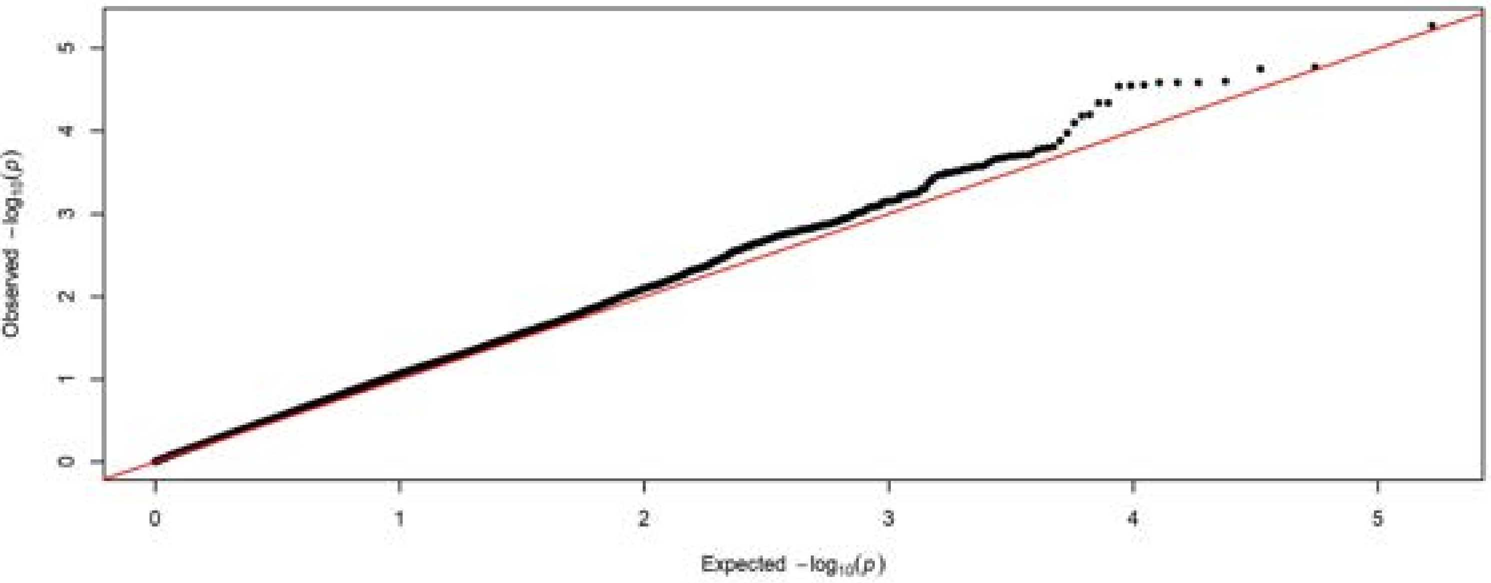

Q-Q Plot Probability of Lever Press Day 1 - Harlan 3 Subgroups - 83k SNPs (n=2175)

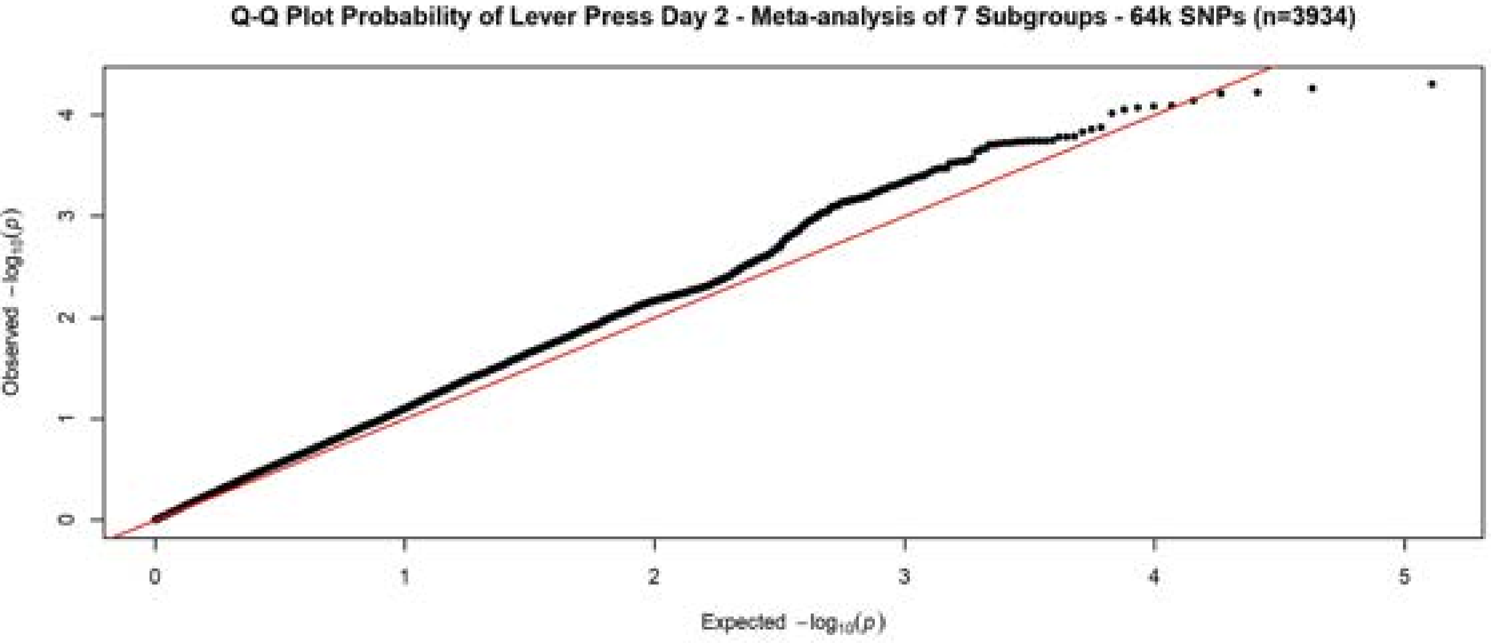
Q-Q Plot Probability of Lever Press Day 2 - Meta-analysis of 7 Subgroups - 64k SNPs (n=3934)
Observed $-\log_{10}(p)$
Expected $-\log_{10}(p)$
0
1
2
3
4
5

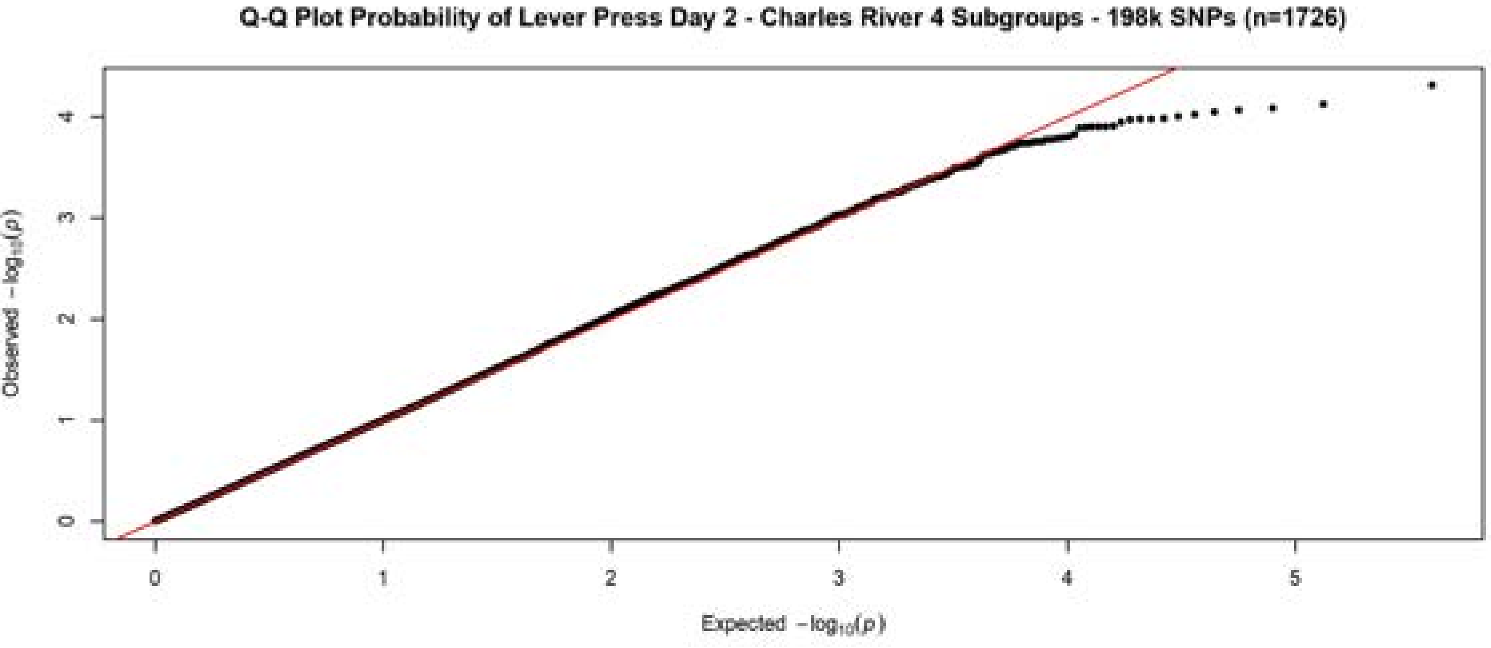
Q-Q Plot Probability of Lever Press Day 2 - Charles River 4 Subgroups - 198k SNPs (n=1726)
Observed $-\log_{10}(p)$
Expected $-\log_{10}(p)$
0
1
2
3
4
5

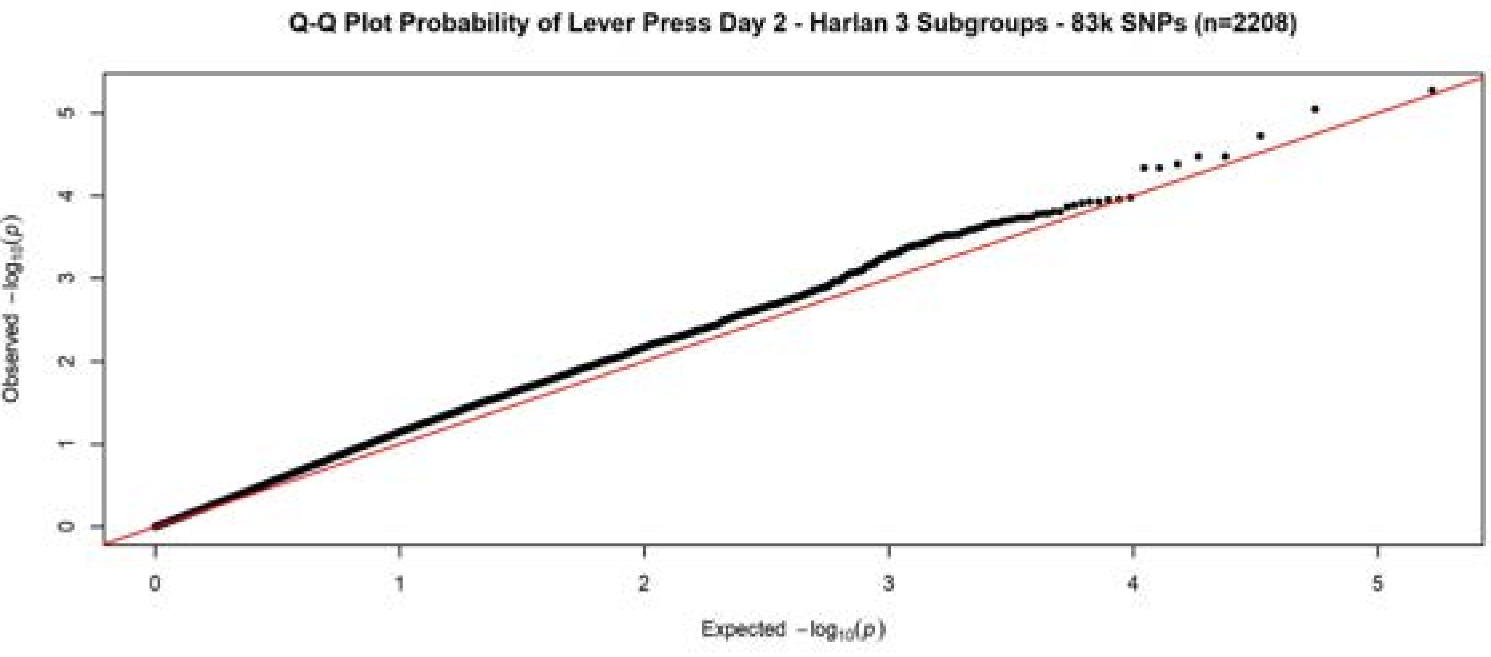
Q-Q Plot Probability of Lever Press Day 2 - Harlan 3 Subgroups - 83k SNPs (n=2208)
Observed $-\log_{10}(p)$
Expected $-\log_{10}(p)$
0
1
2
3
4
5

Q-Q Plot Probability of Lever Press Day 3 - Meta-analysis of 7 Subgroups - 64k SNPs (n=3932)

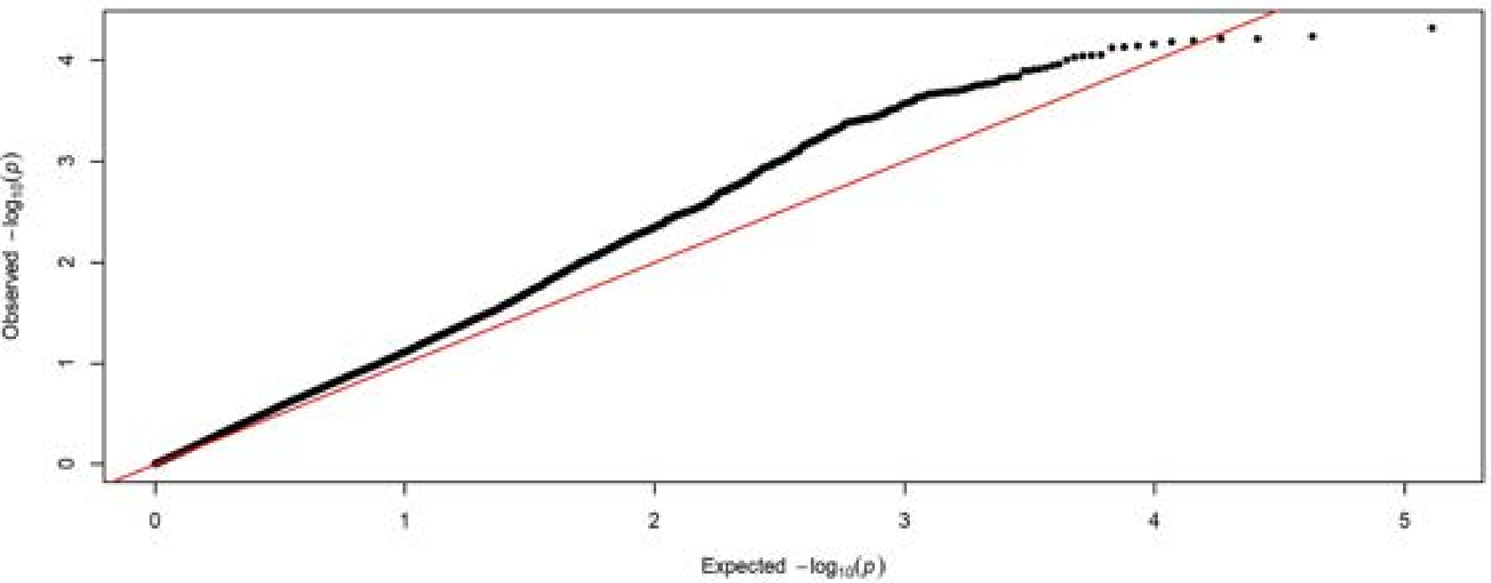

Q-Q Plot Probability of Lever Press Day 3 - Charles River 4 Subgroups - 198k SNPs (n=1727)

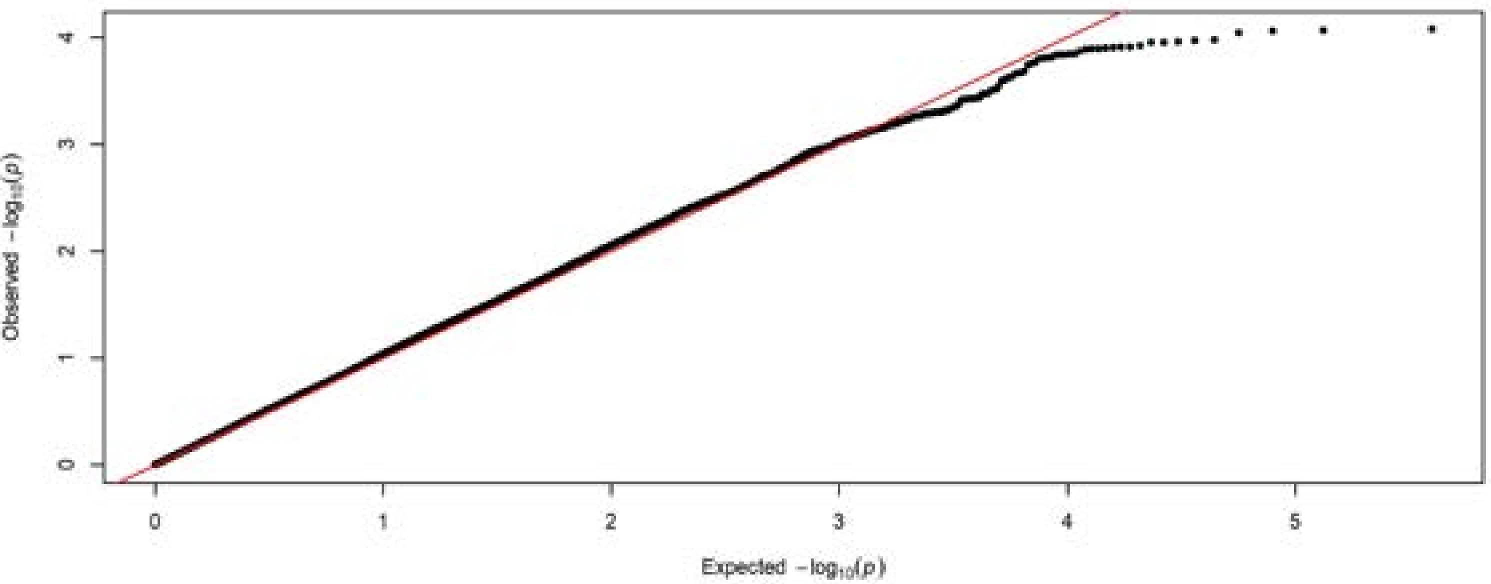

Q-Q Plot Probability of Lever Press Day 3 - Harlan 3 Subgroups - 83k SNPs (n=2205)

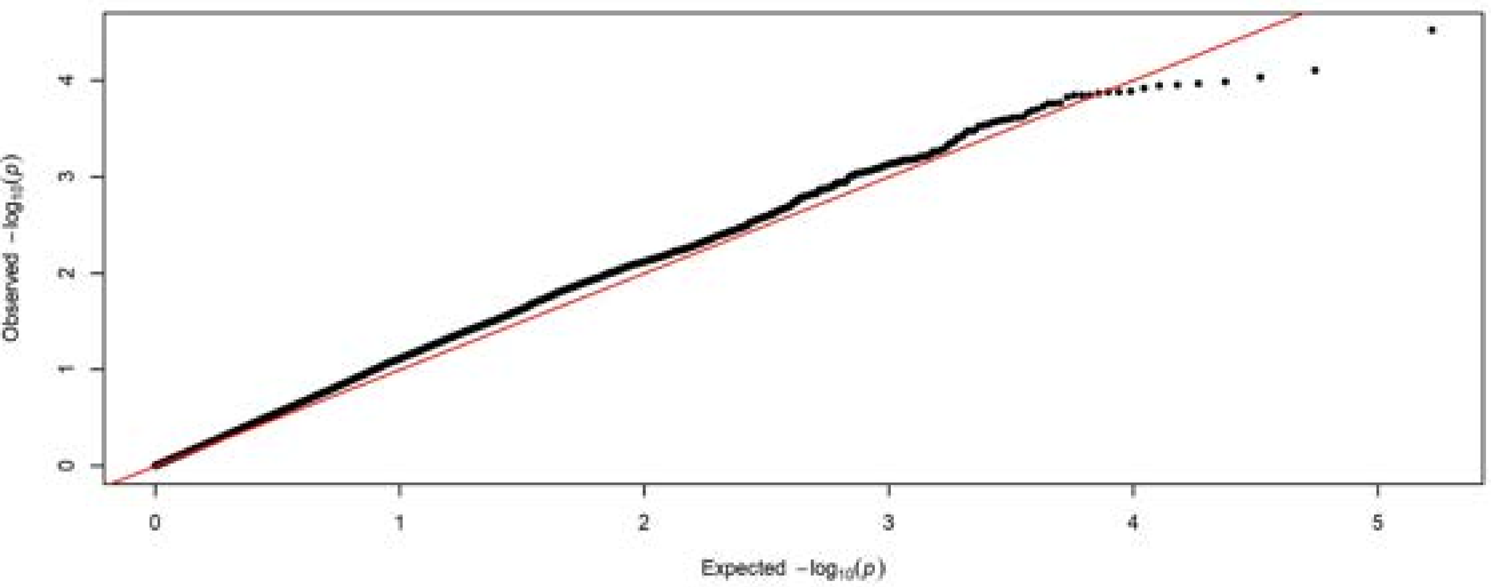

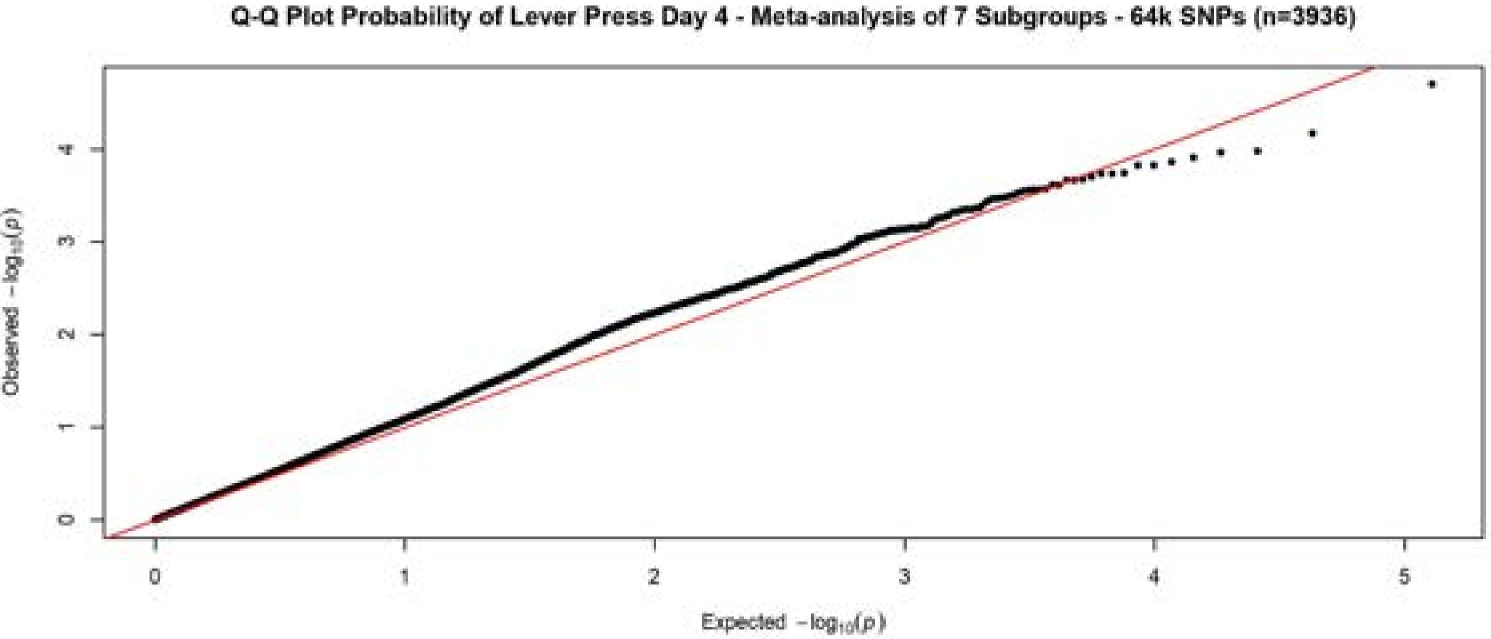
Q-Q Plot Probability of Lever Press Day 4 - Meta-analysis of 7 Subgroups - 64k SNPs (n=3936)
Observed −log10(p)
Expected −log10(p)
0
1
2
3
4
5

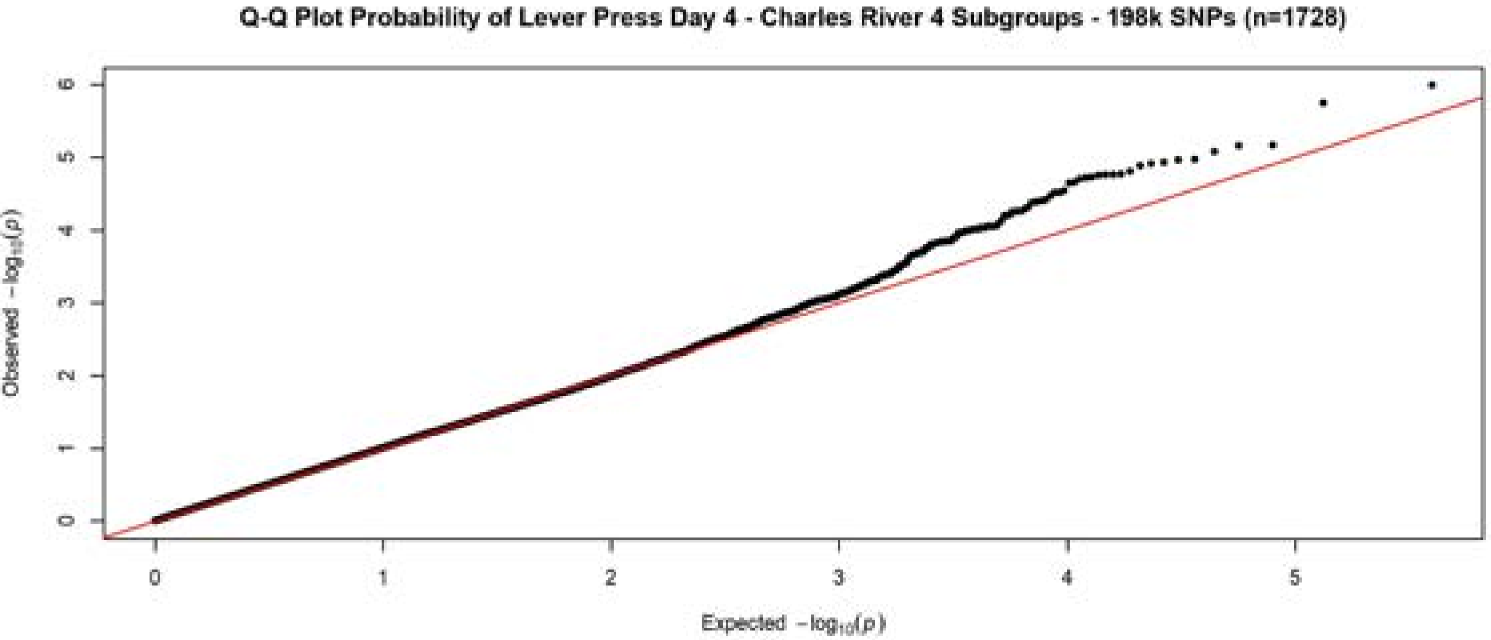
Q-Q Plot Probability of Lever Press Day 4 - Charles River 4 Subgroups - 198k SNPs (n=1728)
Observed −log10(p)
Expected −log10(p)
0
1
2
3
4
5
6

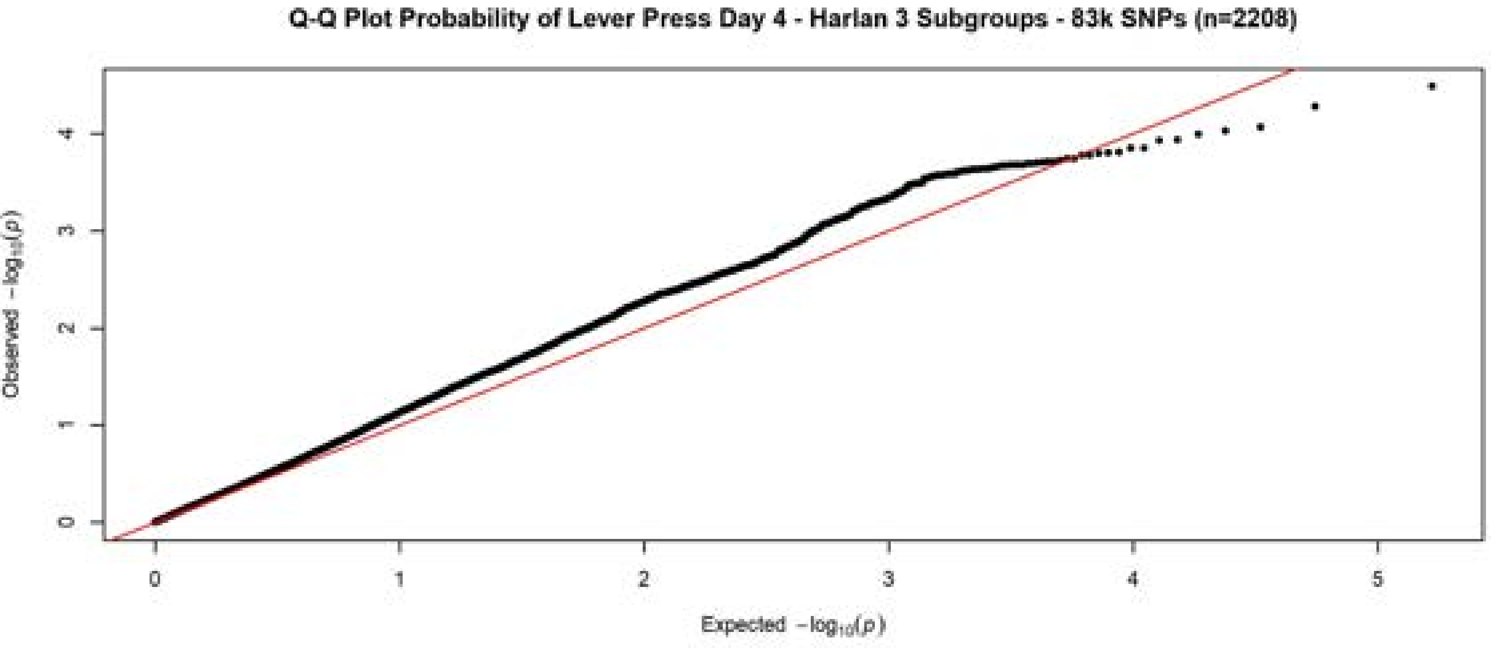
Q-Q Plot Probability of Lever Press Day 4 - Harlan 3 Subgroups - 83k SNPs (n=2208)
Observed −log10(p)
Expected −log10(p)
0
1
2
3
4
5

Q-Q Plot Probability of Lever Press Day 5 - Meta-analysis of 7 Subgroups - 64k SNPs (n=3936)

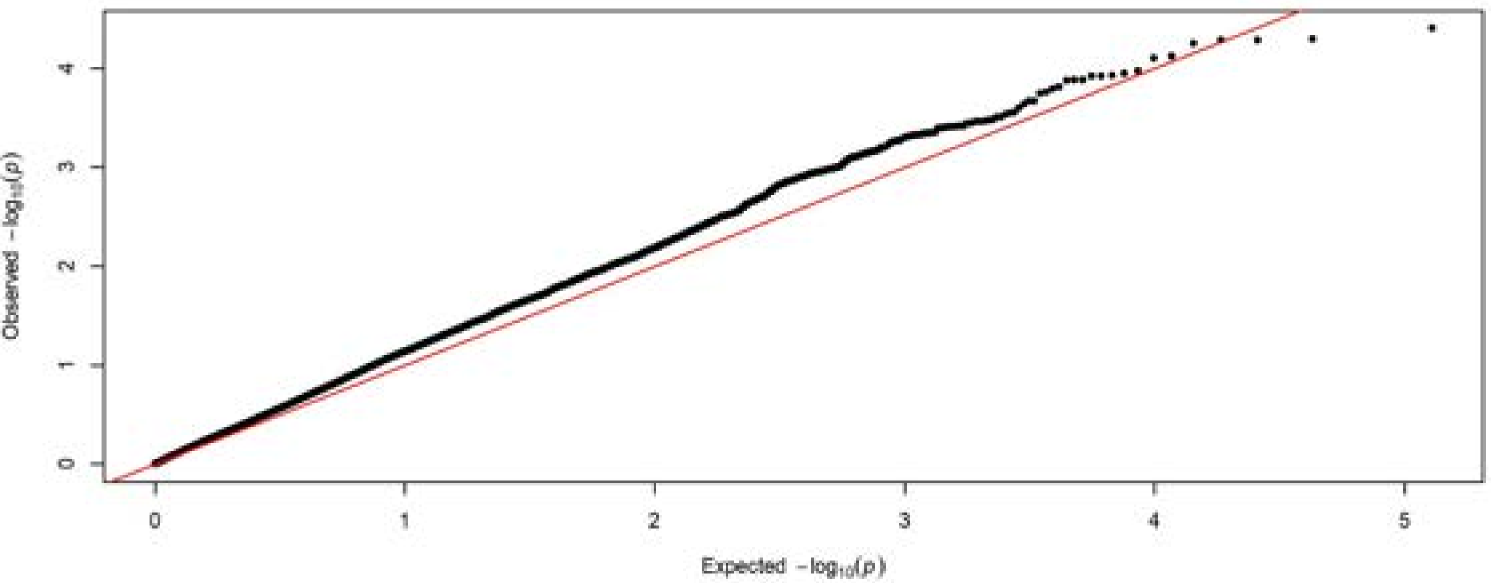

Q-Q Plot Probability of Lever Press Day 5 - Charles River 4 Subgroups - 198k SNPs (n=1728)

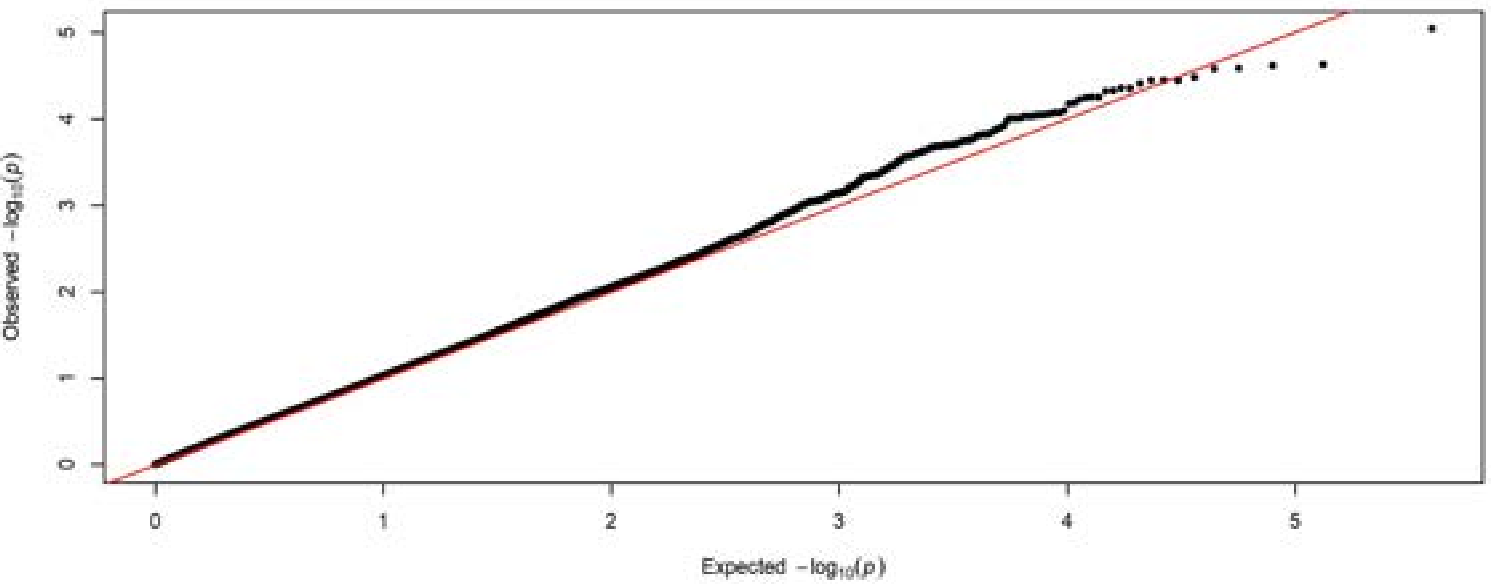

Q-Q Plot Probability of Lever Press Day 5 - Harlan 3 Subgroups - 83k SNPs (n=2208)

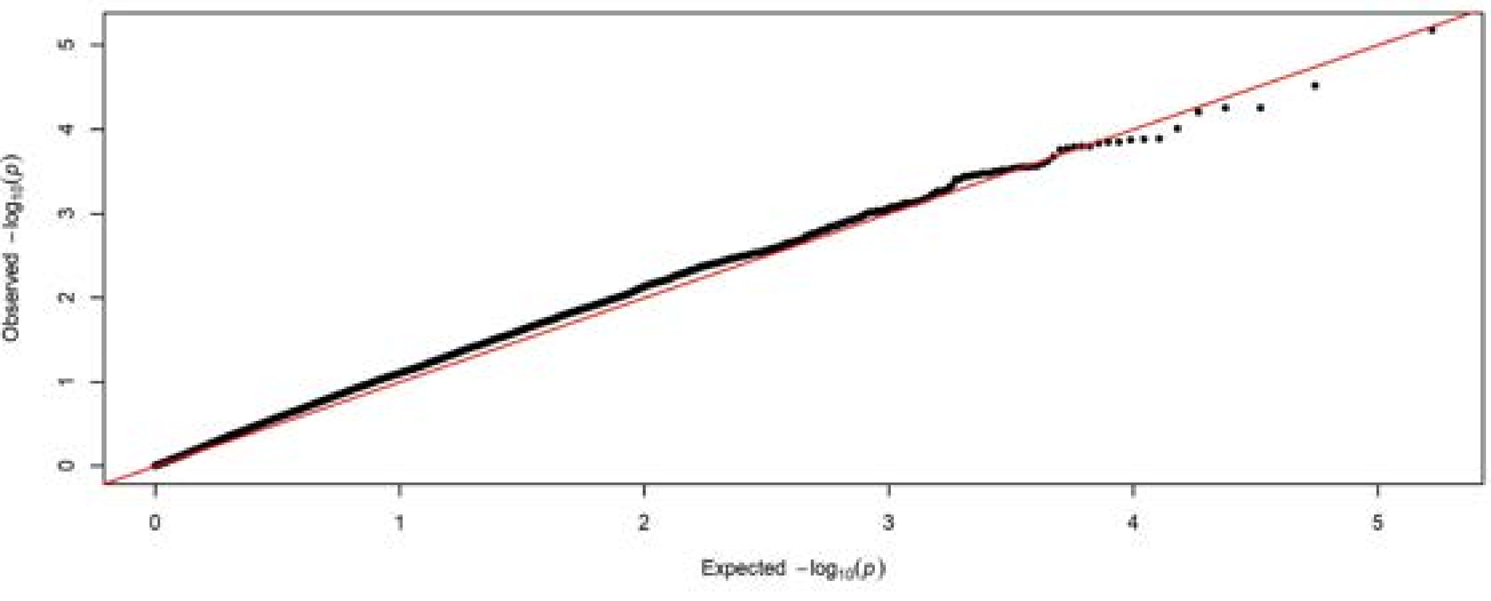

Q-Q Plot Probability of Magazine Entry Day 1 - Meta-analysis of 7 Subgroups - 64k SNPs (n=3903)

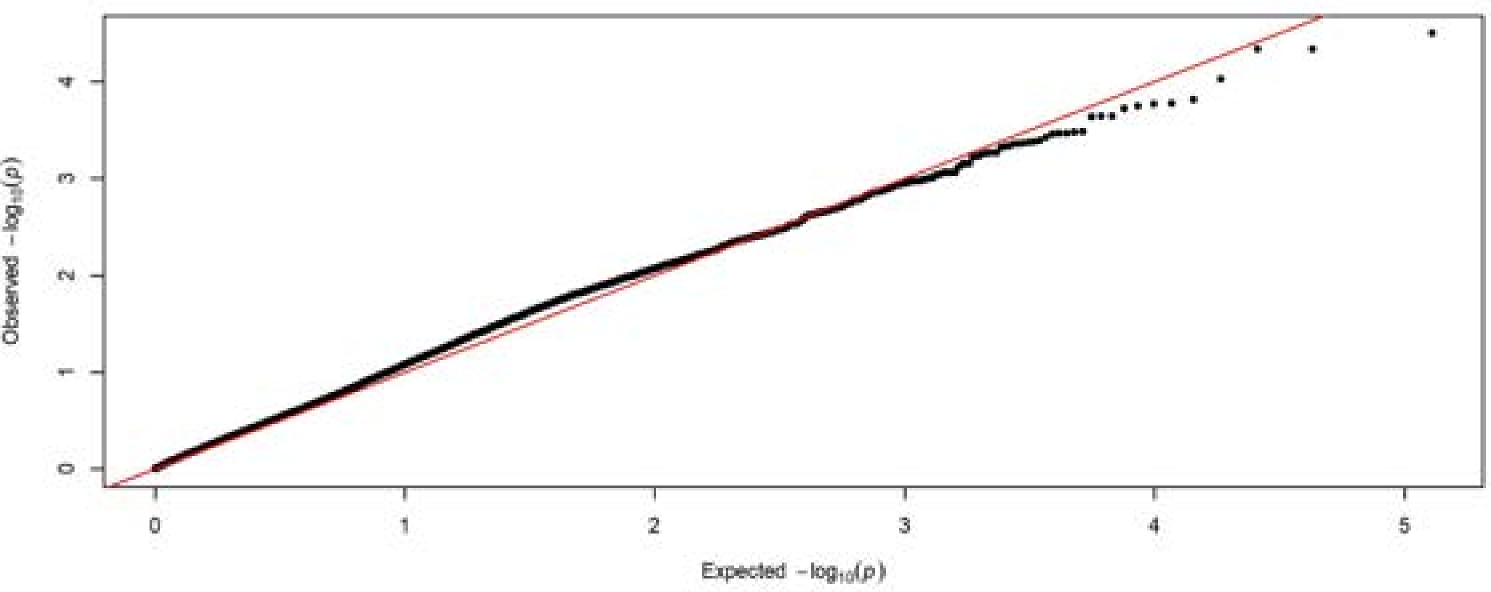

Q-Q Plot Probability of Magazine Entry Day 1 - Charles River 4 Subgroups - 198k SNPs (n=1728)

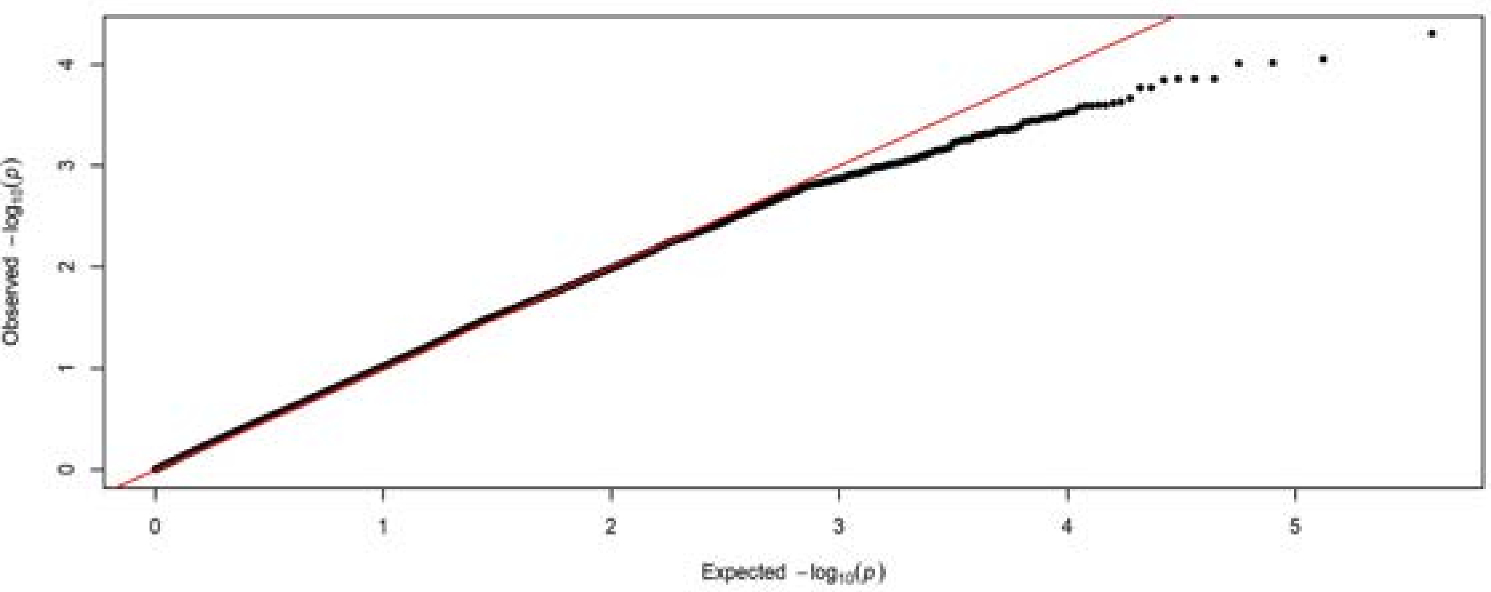

Q-Q Plot Probability of Magazine Entry Day 1 - Harlan 3 Subgroups - 83k SNPs (n=2175)

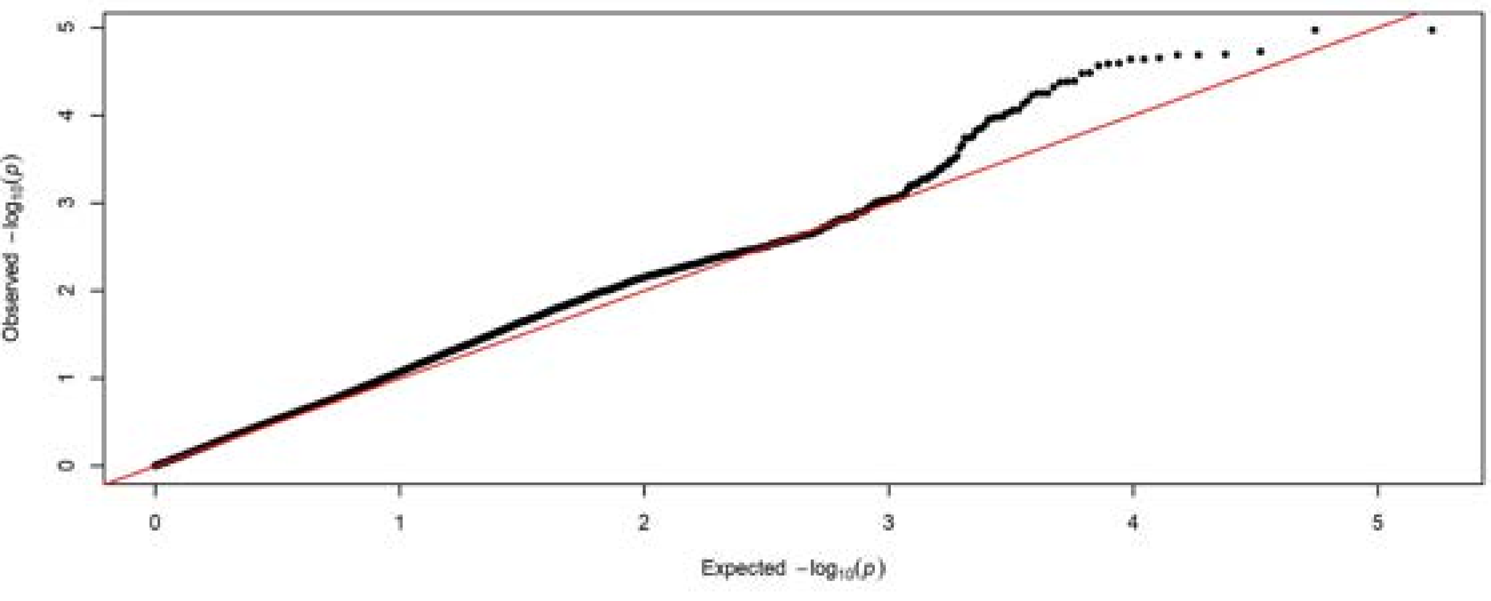

Q-Q Plot Probability of Magazine Entry Day 2 - Meta-analysis of 7 Subgroups - 64k SNPs (n=3934)

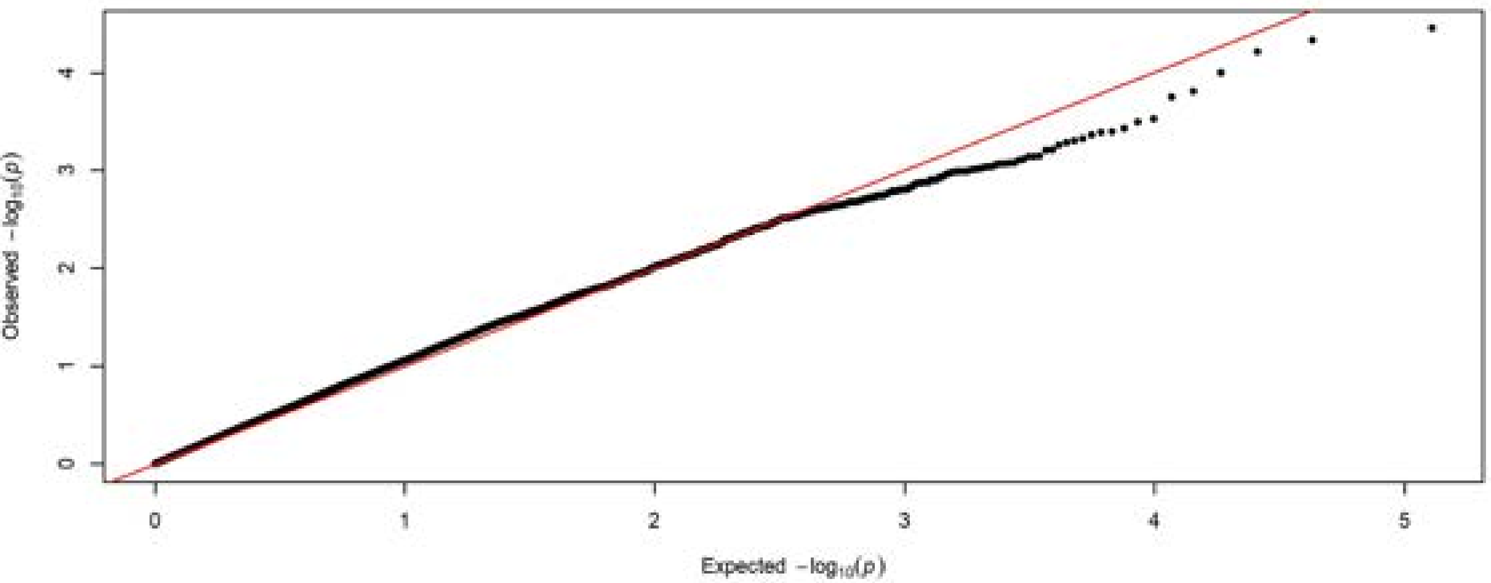

Q-Q Plot Probability of Magazine Entry Day 2 - Charles River 4 Subgroups - 198k SNPs (n=1726)

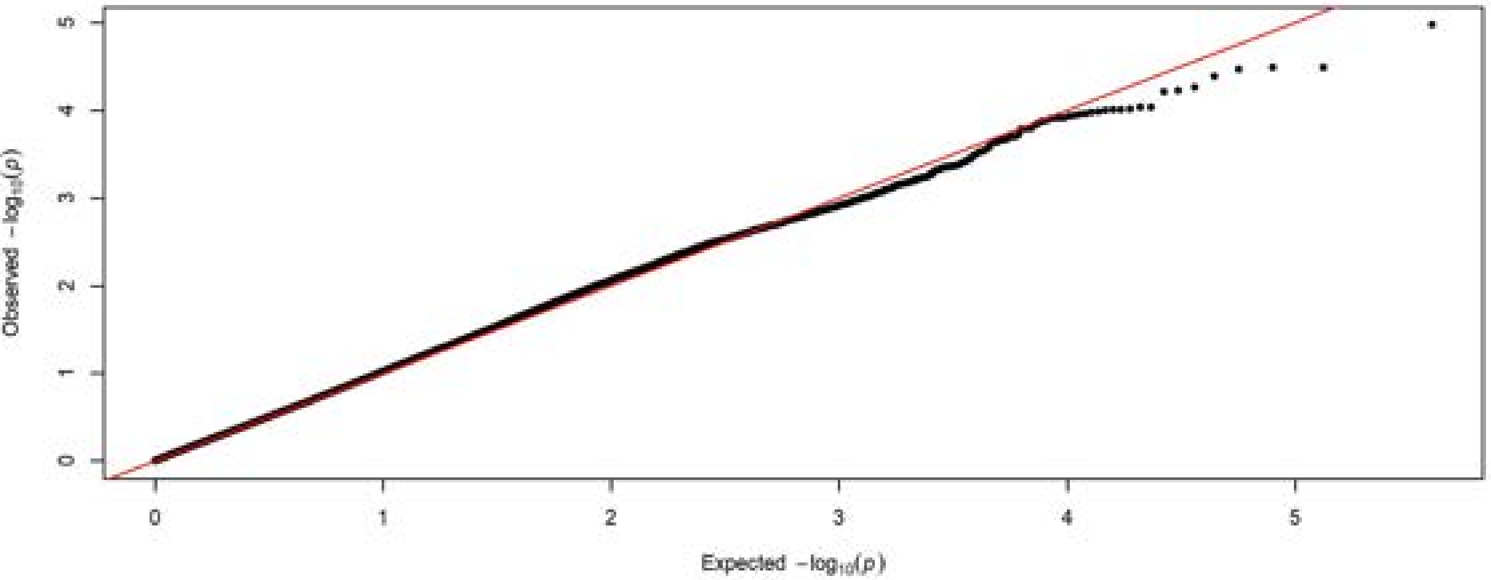

Q-Q Plot Probability of Magazine Entry Day 2 - Harlan 3 Subgroups - 83k SNPs (n=2208)

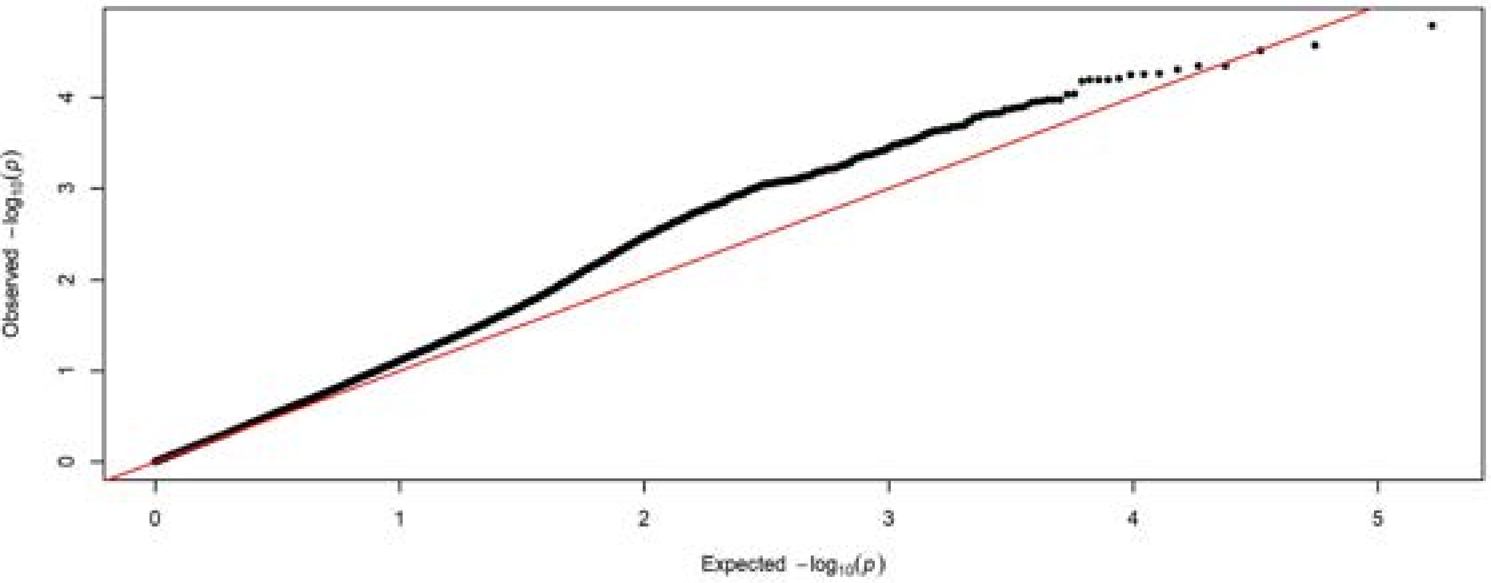

Q-Q Plot Probability of Magazine Entry Day 3 - Meta-analysis of 7 Subgroups - 64k SNPs (n=3932)

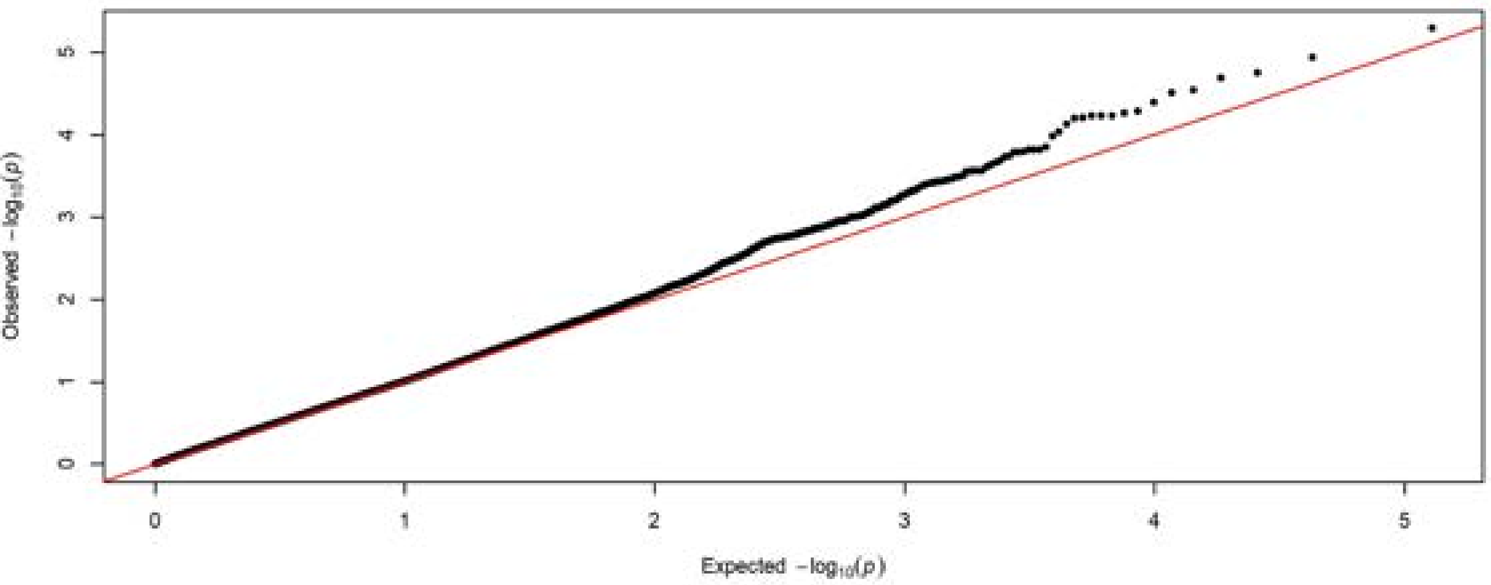

Q-Q Plot Probability of Magazine Entry Day 3 - Charles River 4 Subgroups - 198k SNPs (n=1727)

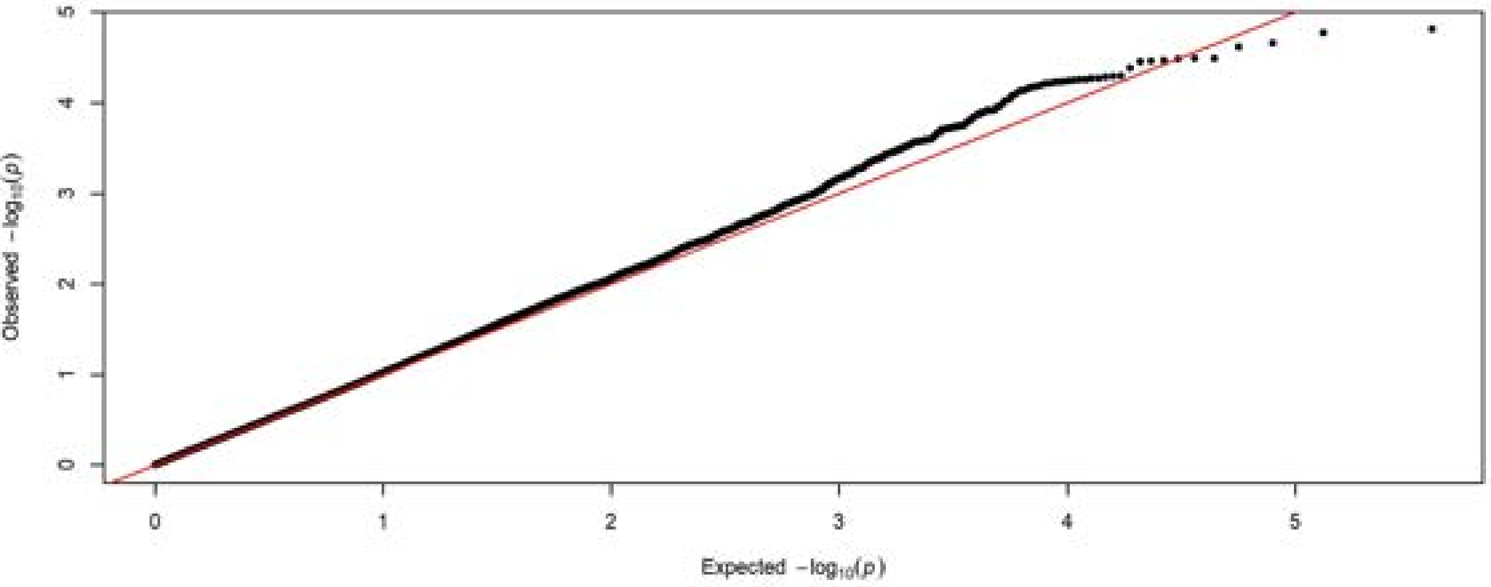

Q-Q Plot Probability of Magazine Entry Day 3 - Harlan 3 Subgroups - 83k SNPs (n=2205)

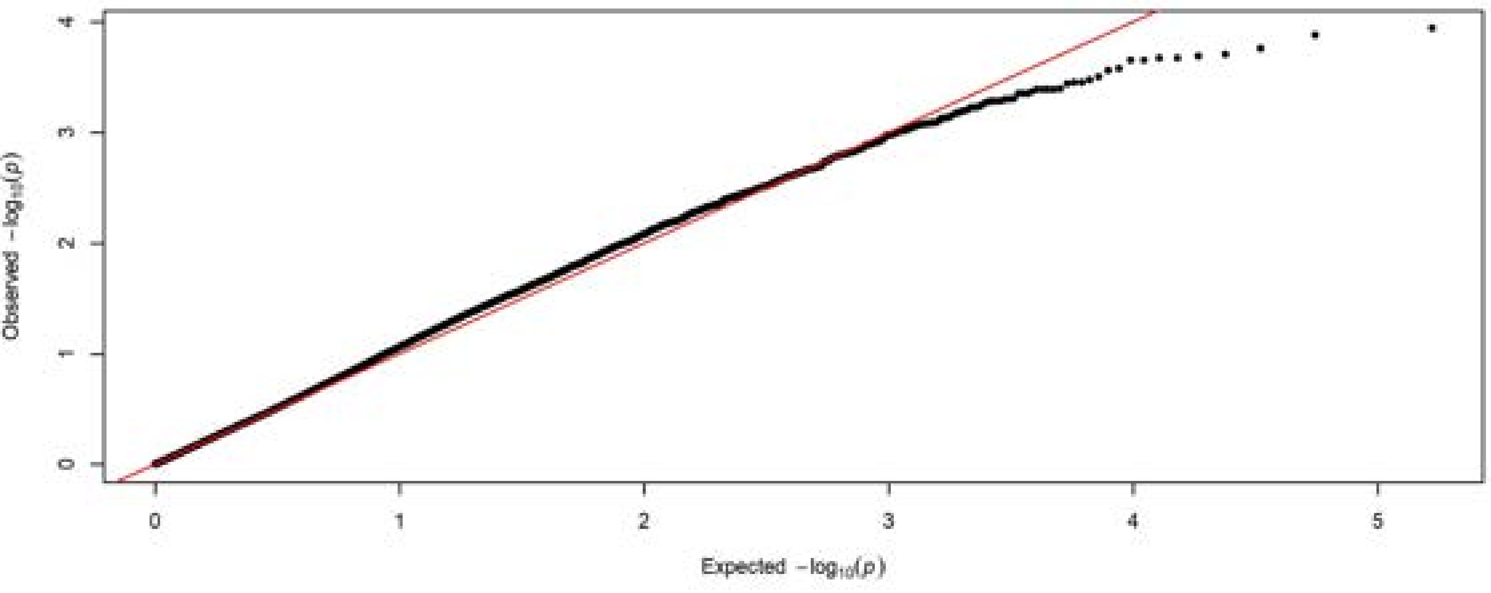

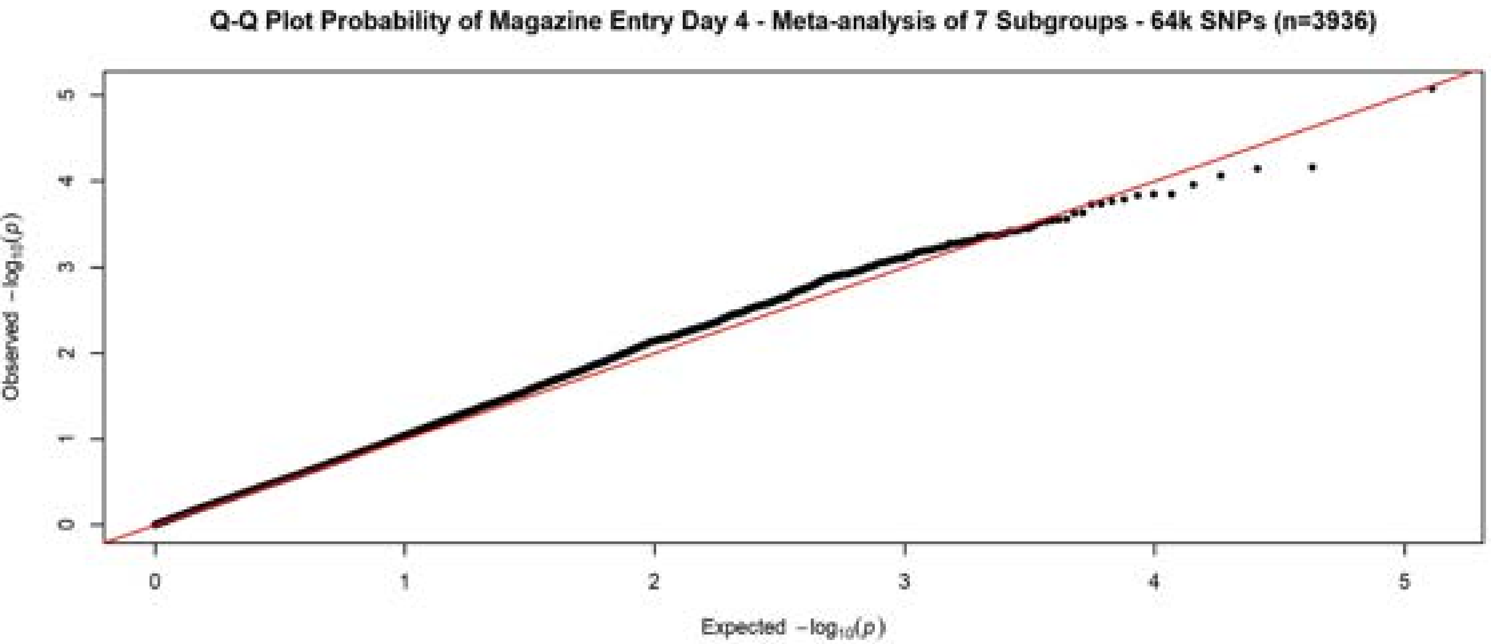
Q-Q Plot Probability of Magazine Entry Day 4 - Meta-analysis of 7 Subgroups - 64k SNPs (n=3936)
Observed $-\log_{10}(p)$
Expected $-\log_{10}(p)$
0
1
2
3
4
5

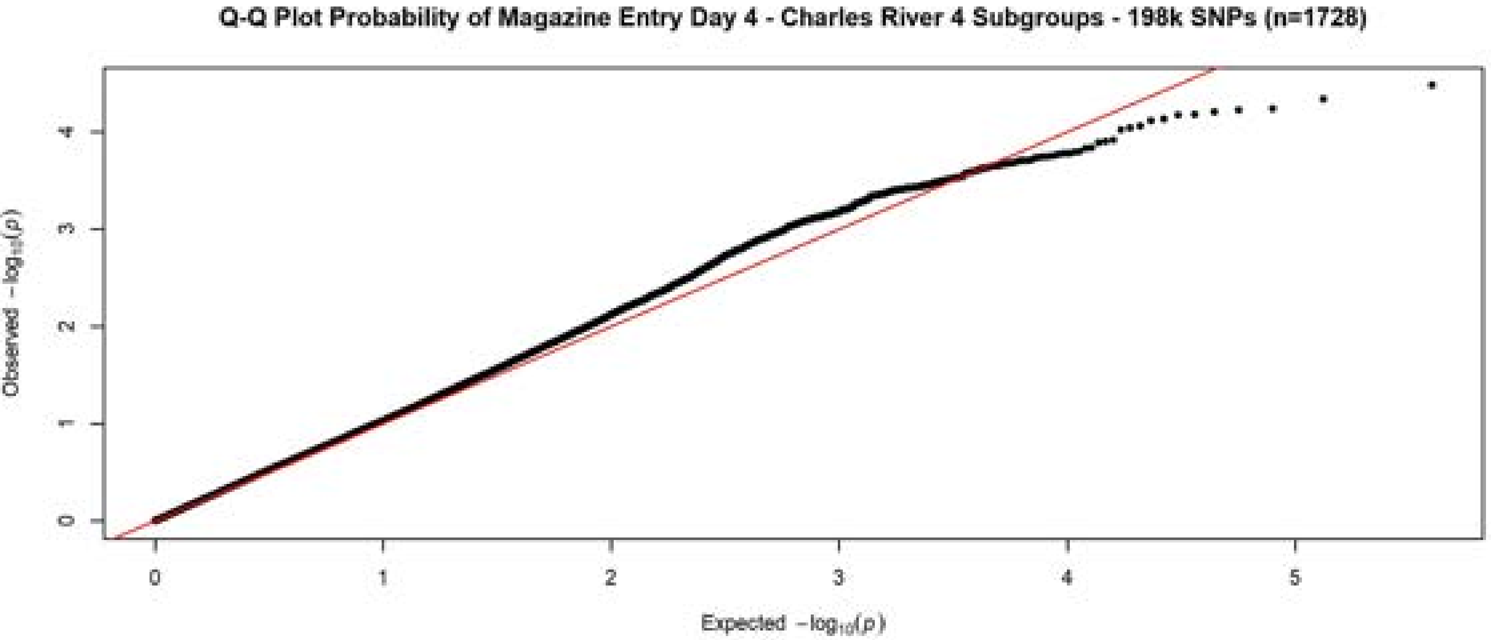
Q-Q Plot Probability of Magazine Entry Day 4 - Charles River 4 Subgroups - 198k SNPs (n=1728)
Observed $-\log_{10}(p)$
Expected $-\log_{10}(p)$
0
1
2
3
4
5

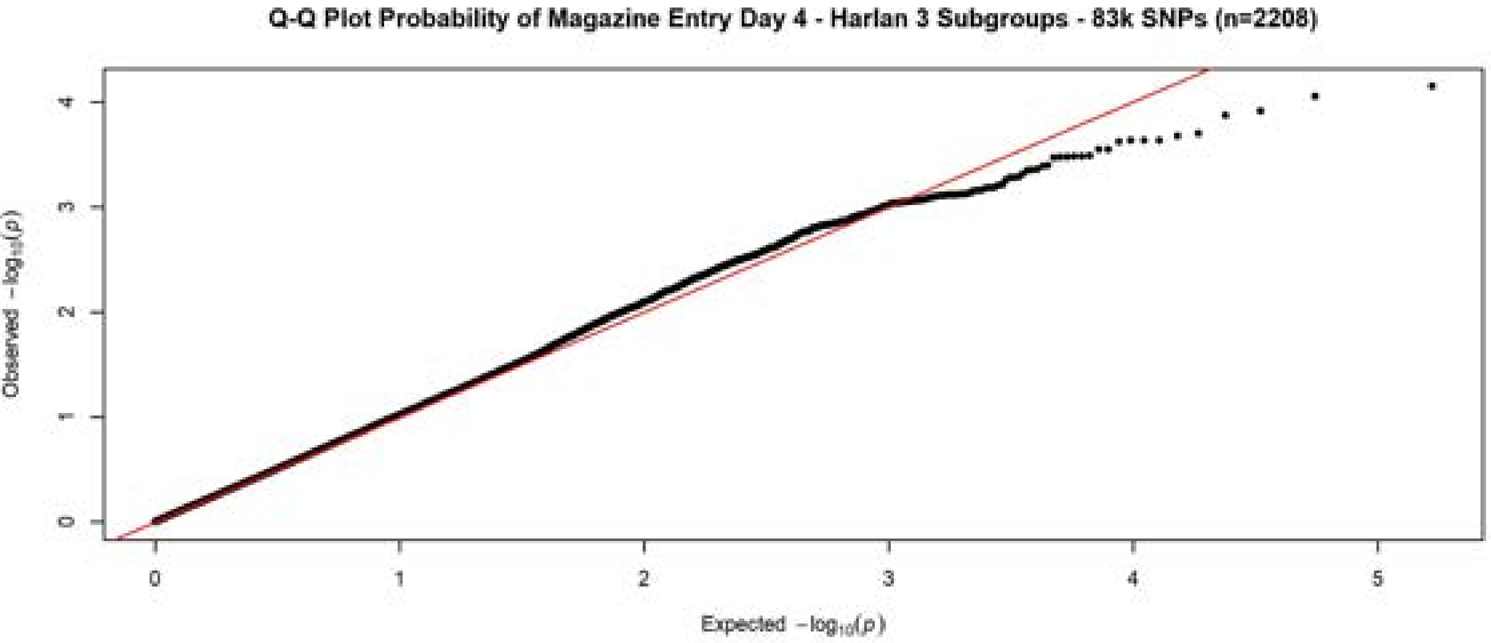
Q-Q Plot Probability of Magazine Entry Day 4 - Harlan 3 Subgroups - 83k SNPs (n=2208)
Observed $-\log_{10}(p)$
Expected $-\log_{10}(p)$
0
1
2
3
4
5

Q-Q Plot Probability of Magazine Entry Day 5 - Meta-analysis of 7 Subgroups - 64k SNPs (n=3936)

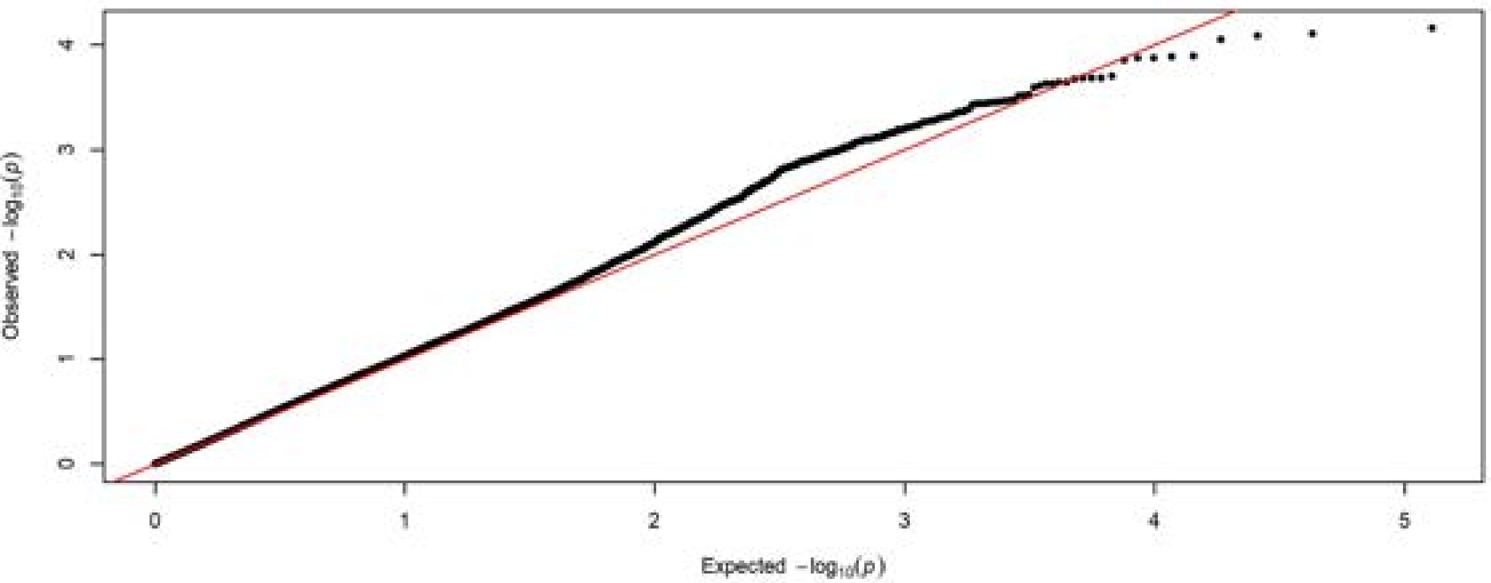

Q-Q Plot Probability of Magazine Entry Day 5 - Charles River 4 Subgroups - 198k SNPs (n=1728)

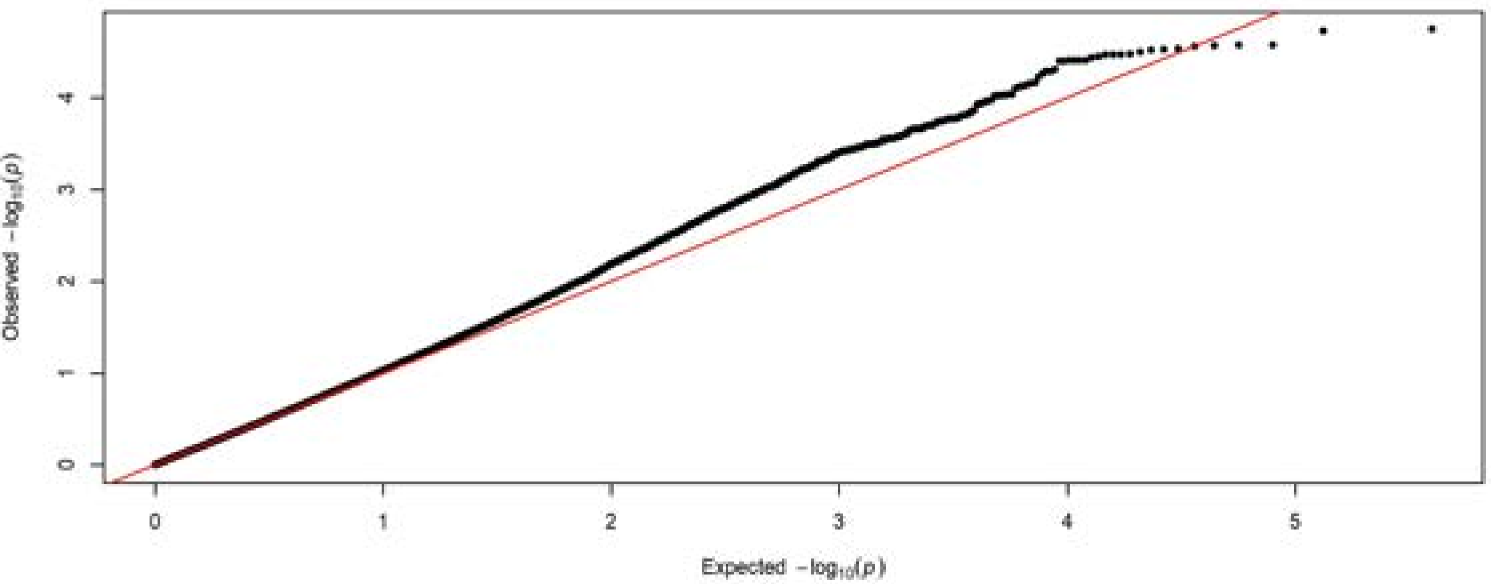

Q-Q Plot Probability of Magazine Entry Day 5 - Harlan 3 Subgroups - 83k SNPs (n=2208)

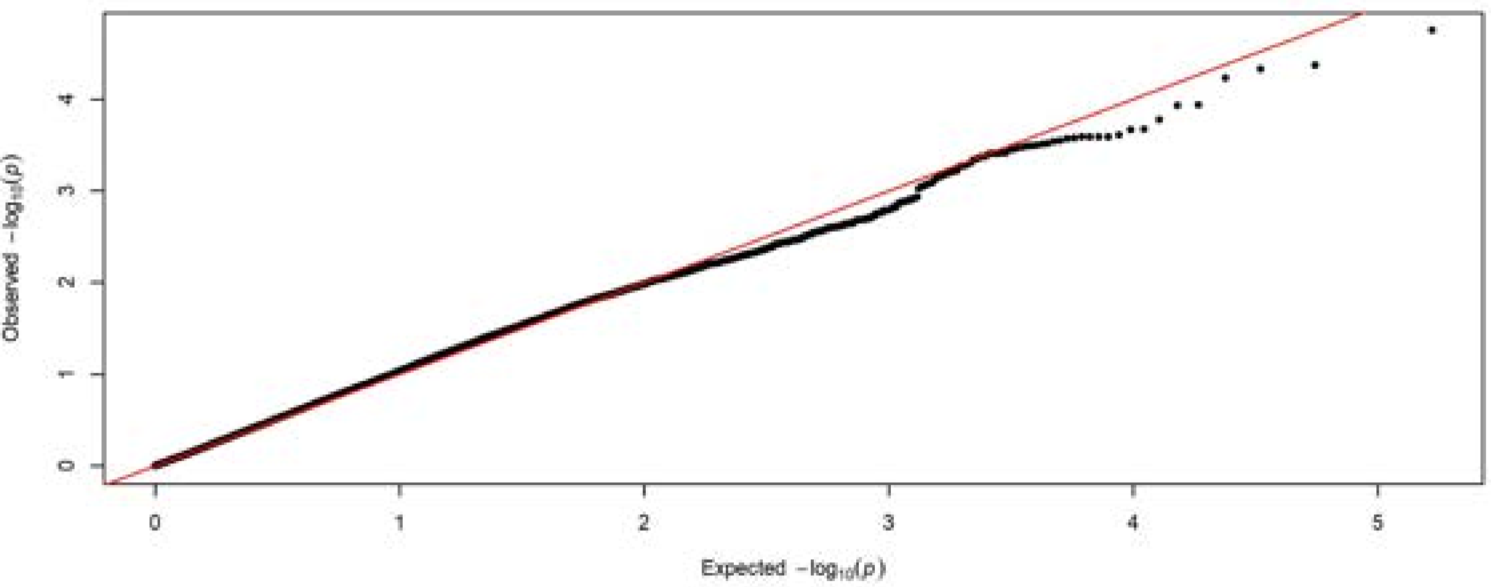

Q-Q Plot Response Bias Day 1 - Meta-analysis of 7 Subgroups - 64k SNPs (n=3912)

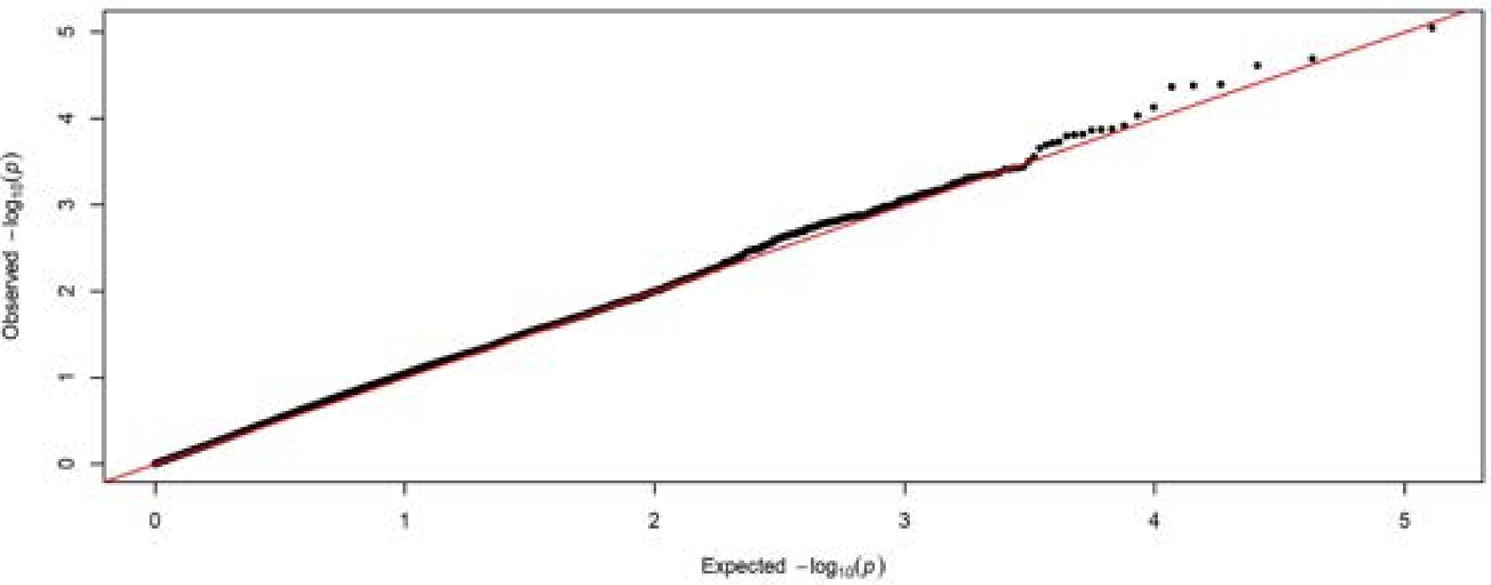

Q-Q Plot Response Bias Day 1 - Charles River 4 Subgroups - 198k SNPs (n=1720)

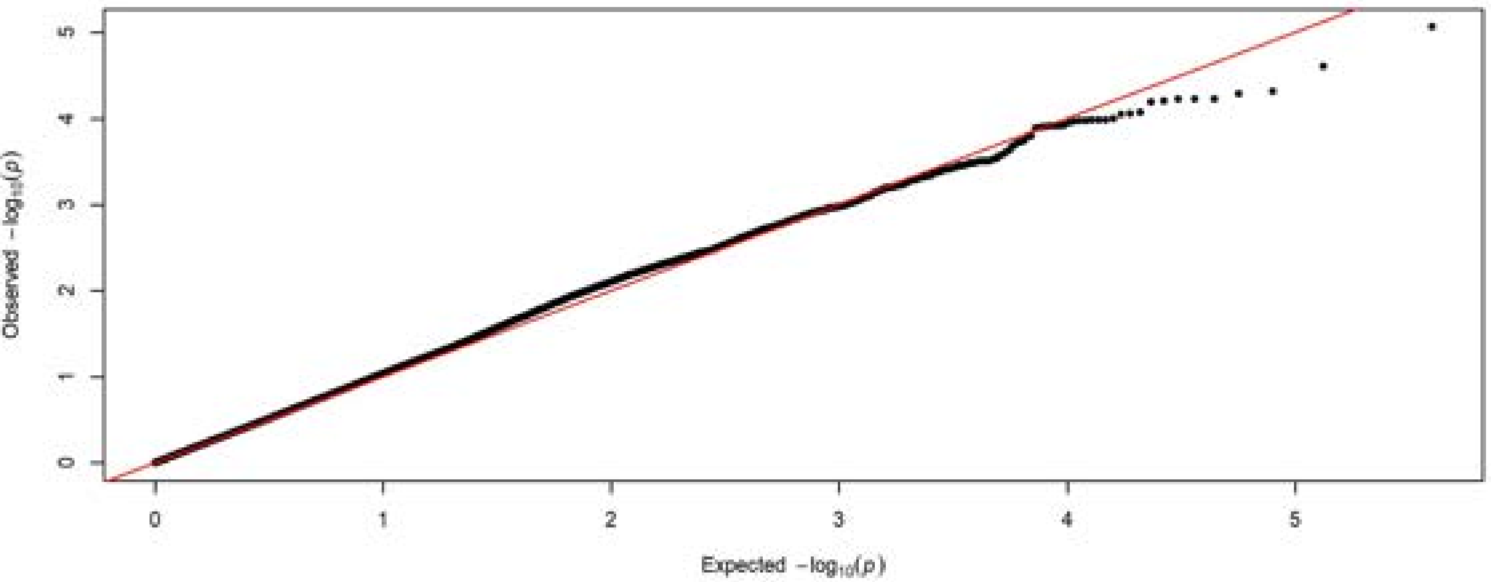

Q-Q Plot Response Bias Day 1 - Harlan 3 Subgroups - 83k SNPs (n=2192)

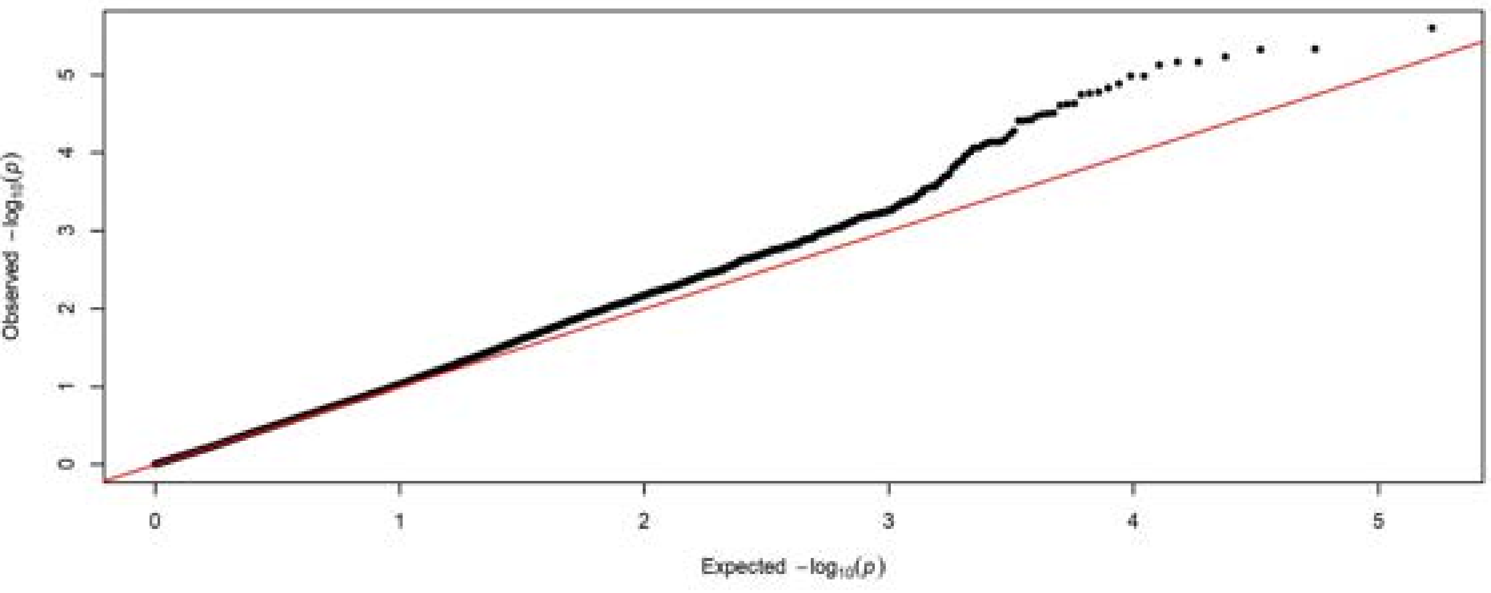

Q-Q Plot Response Bias Day 2 - Meta-analysis of 7 Subgroups - 64k SNPs (n=3919)

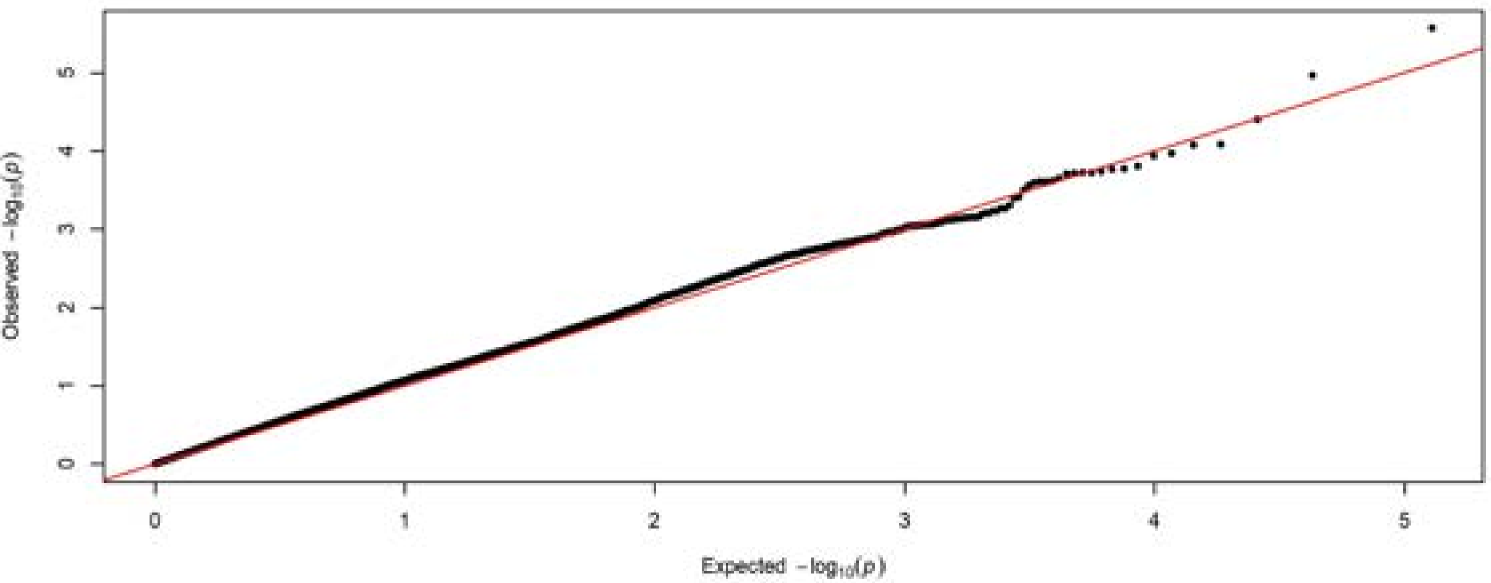

Q-Q Plot Response Bias Day 2 - Charles River 4 Subgroups - 198k SNPs (n=1719)

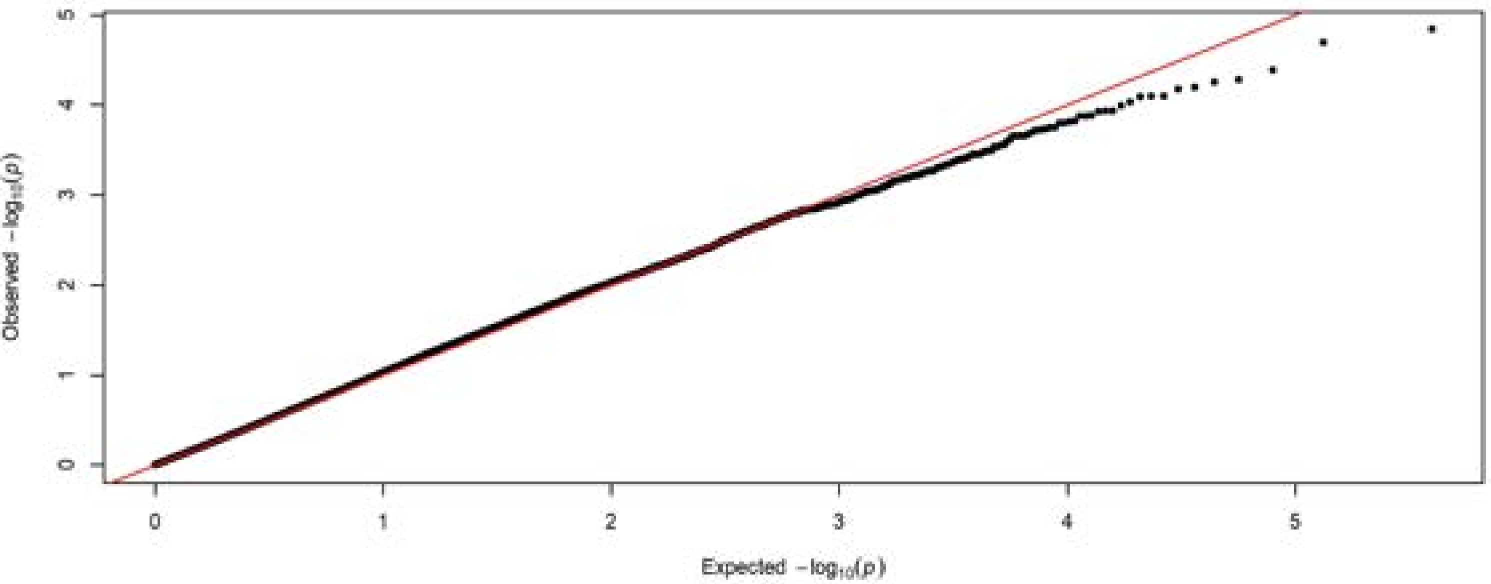

Q-Q Plot Response Bias Day 2 - Harlan 3 Subgroups - 83k SNPs (n=2200)

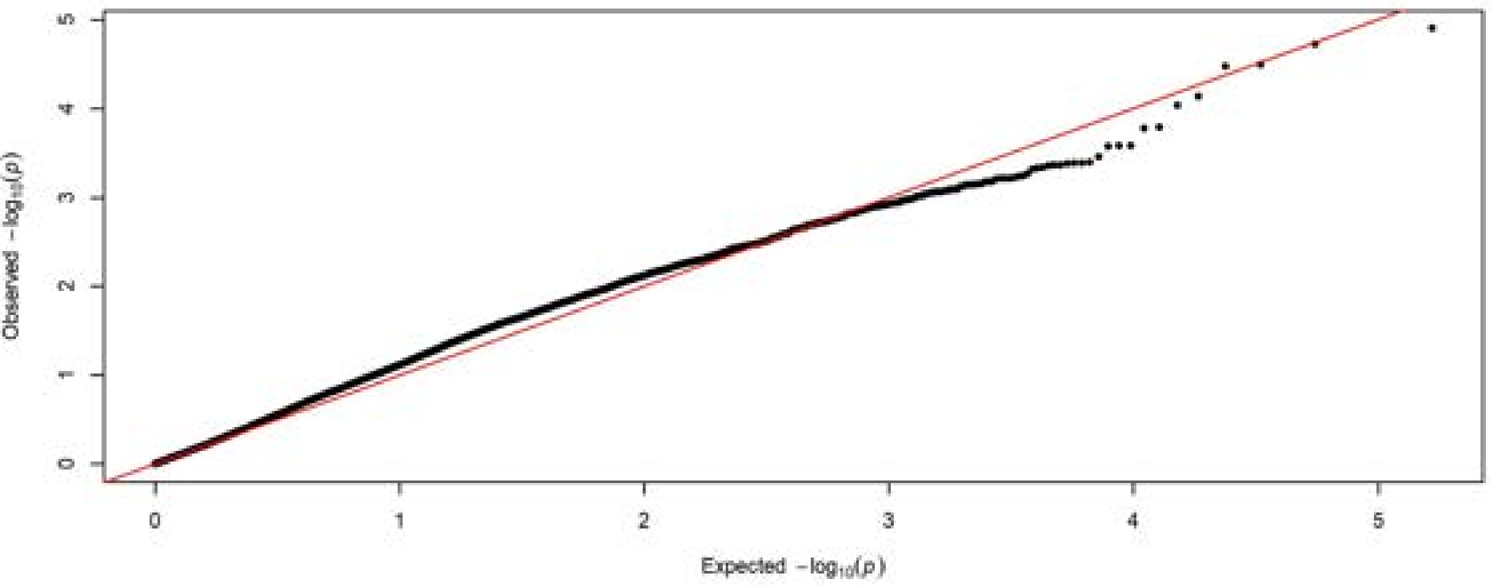

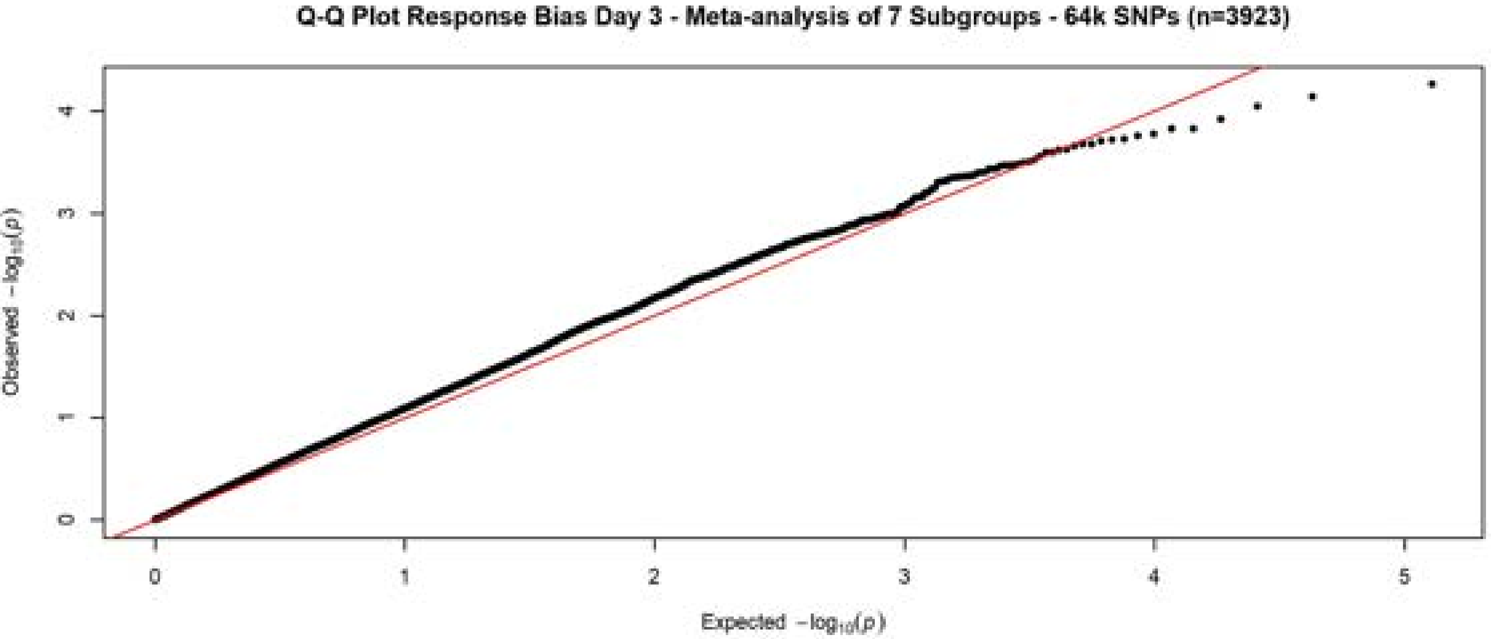
Q-Q Plot Response Bias Day 3 - Meta-analysis of 7 Subgroups - 64k SNPs (n=3923)
Observed $-\log_{10}(p)$
Expected $-\log_{10}(p)$

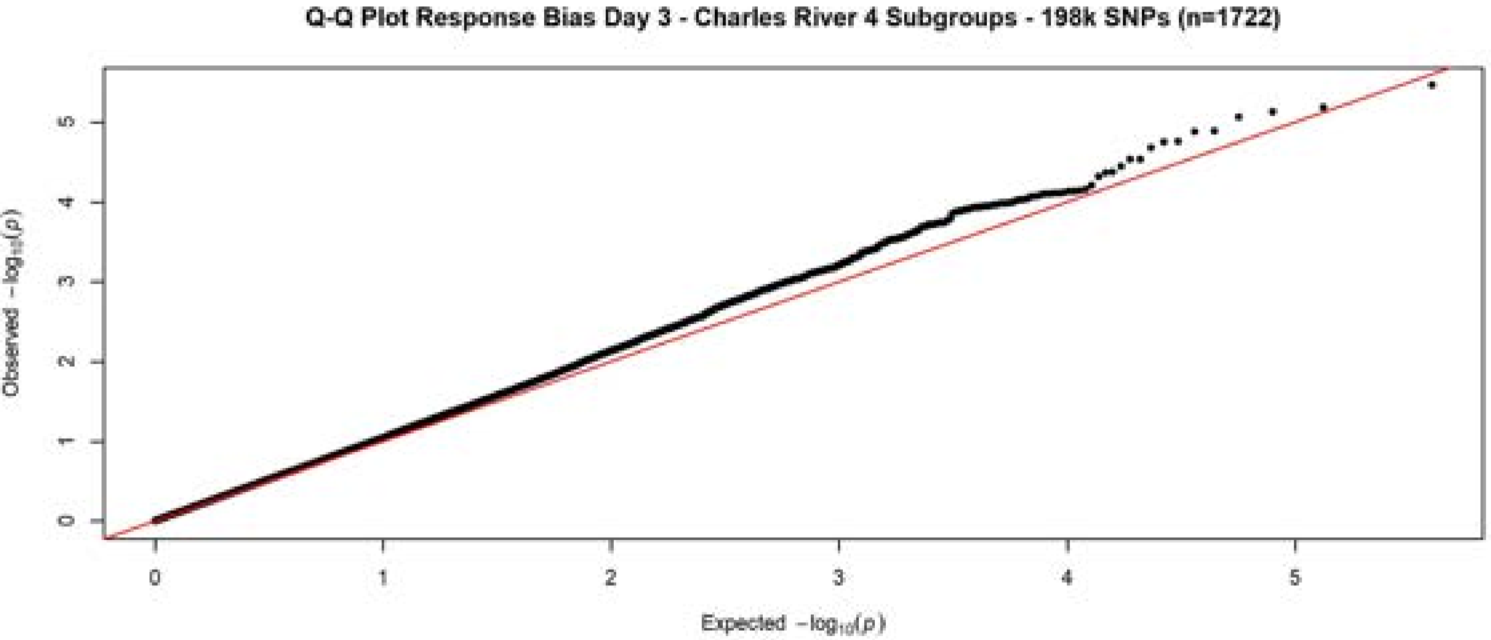
Q-Q Plot Response Bias Day 3 - Charles River 4 Subgroups - 198k SNPs (n=1722)
Observed $-\log_{10}(p)$
Expected $-\log_{10}(p)$

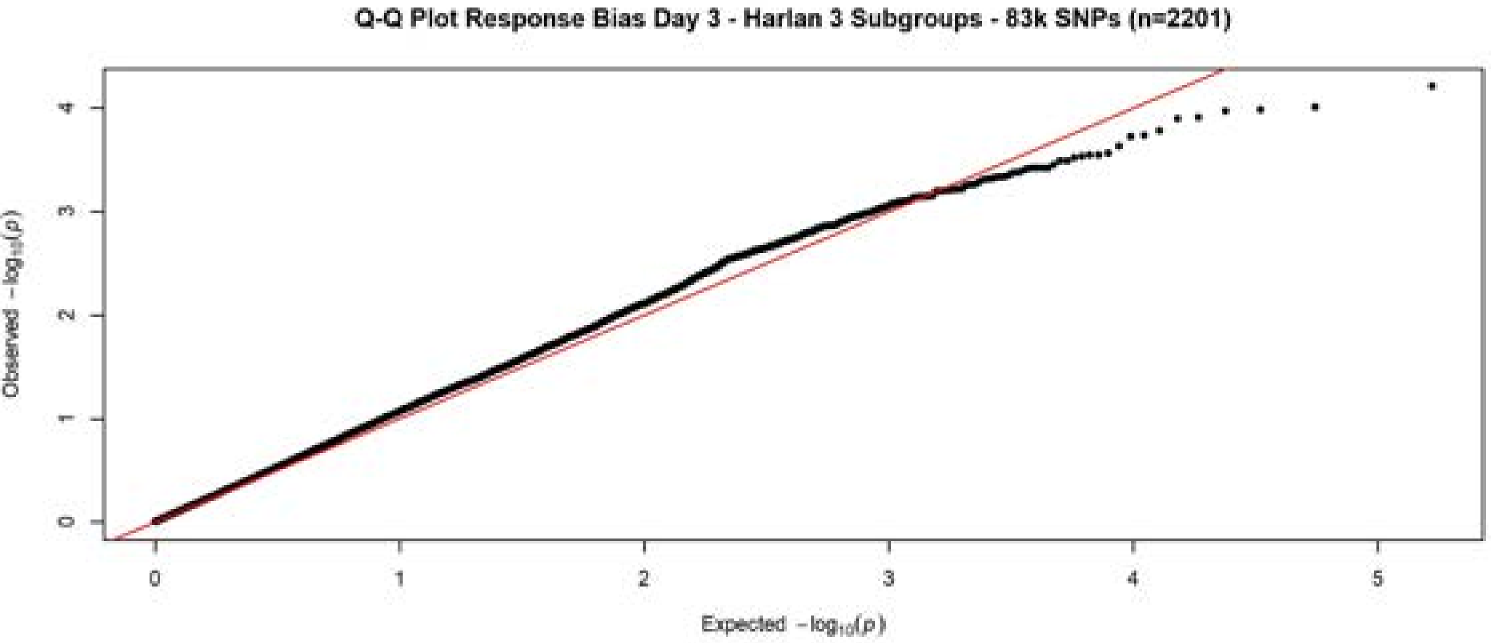
Q-Q Plot Response Bias Day 3 - Harlan 3 Subgroups - 83k SNPs (n=2201)
Observed $-\log_{10}(p)$
Expected $-\log_{10}(p)$

Q-Q Plot Response Bias Day 4 - Meta-analysis of 7 Subgroups - 64k SNPs (n=3935)

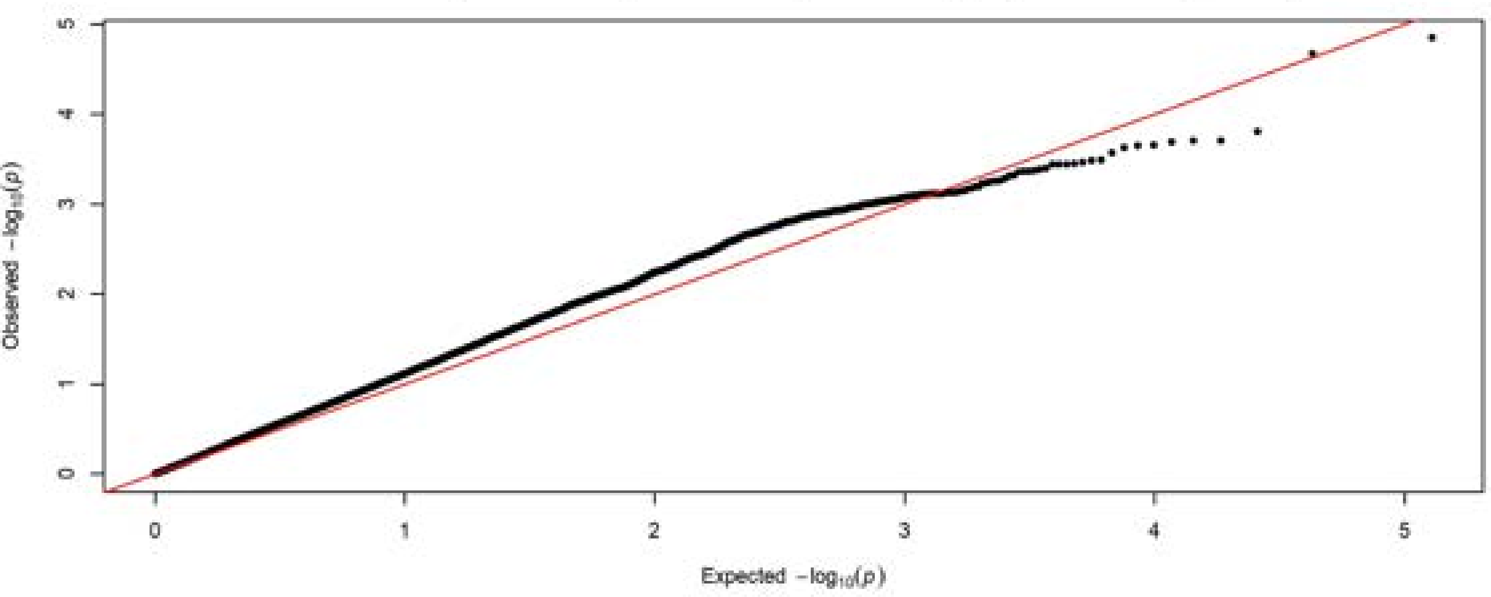

Q-Q Plot Response Bias Day 4 - Charles River 4 Subgroups - 198k SNPs (n=1727)

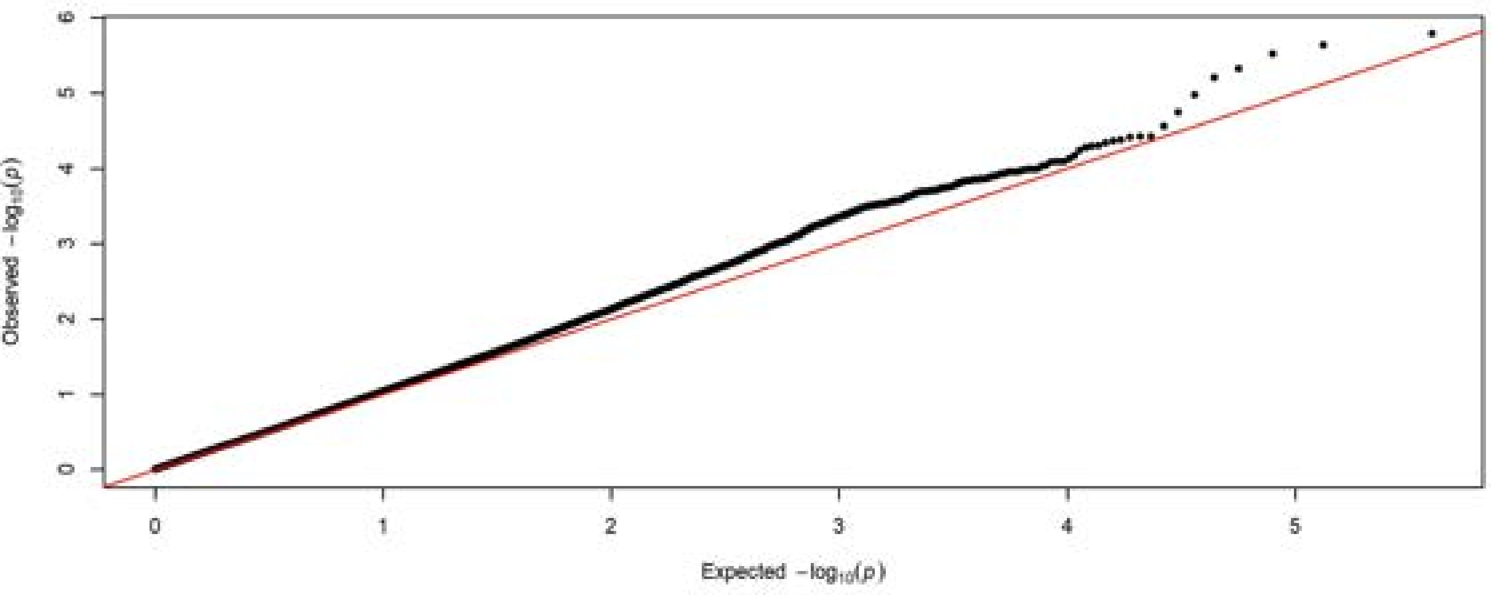

Q-Q Plot Response Bias Day 4 - Harlan 3 Subgroups - 83k SNPs (n=2208)

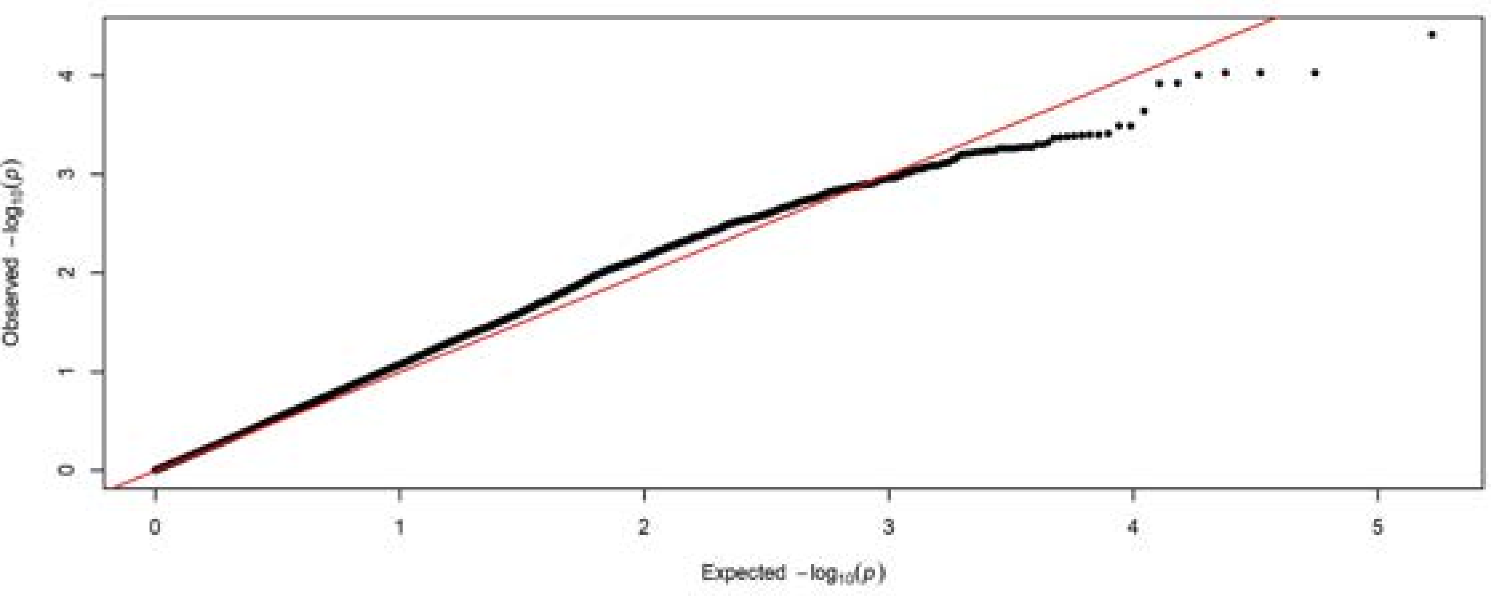

Q-Q Plot Response Bias Day 5 - Meta-analysis of 7 Subgroups - 64k SNPs (n=3933)

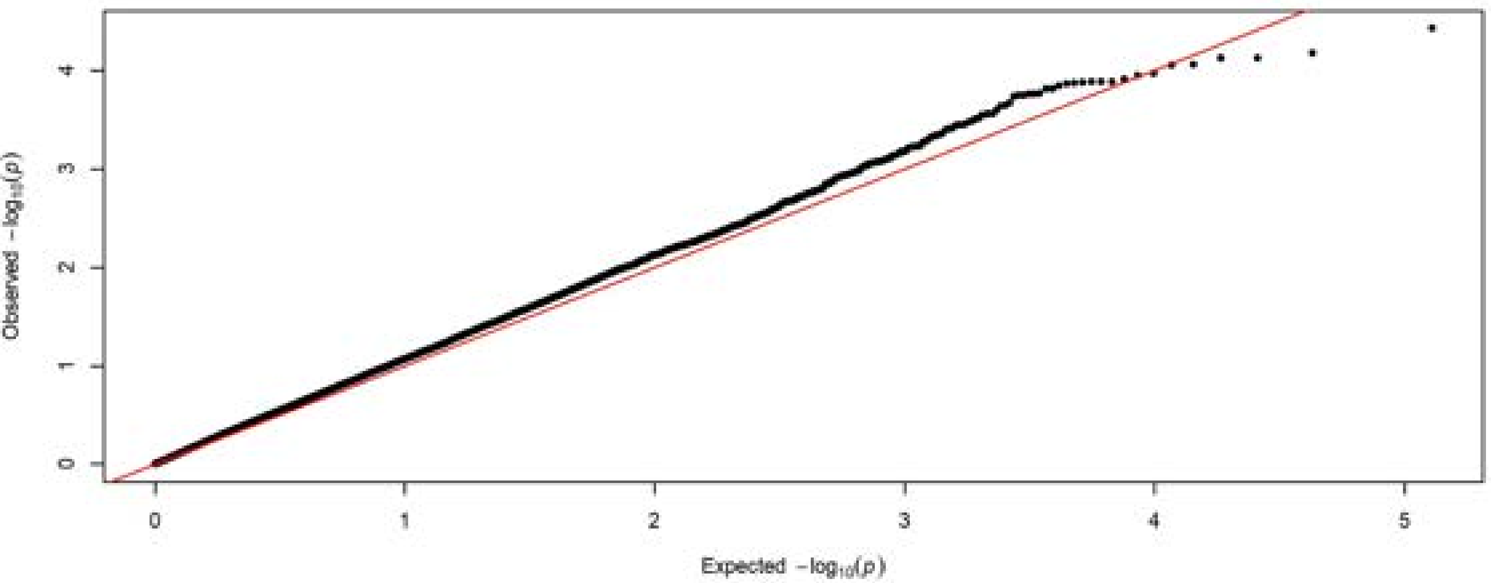

Q-Q Plot Response Bias Day 5 - Charles River 4 Subgroups - 198k SNPs (n=1726)

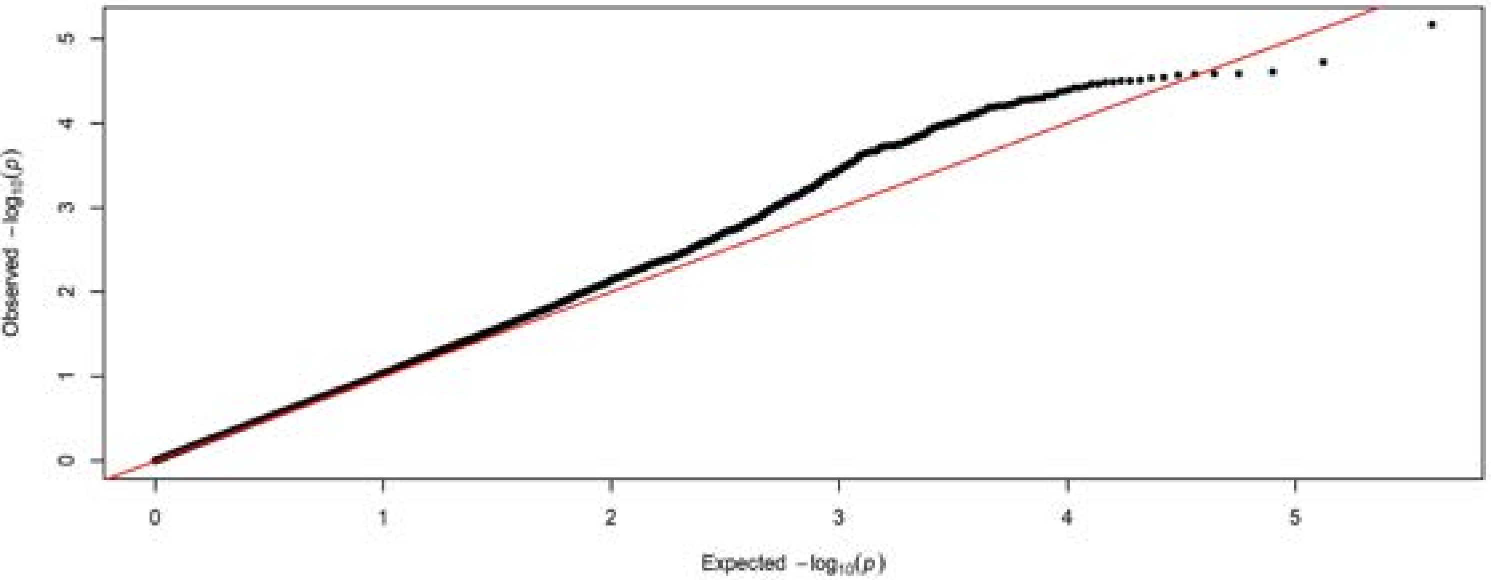

Q-Q Plot Response Bias Day 5 - Harlan 3 Subgroups - 83k SNPs (n=2207)

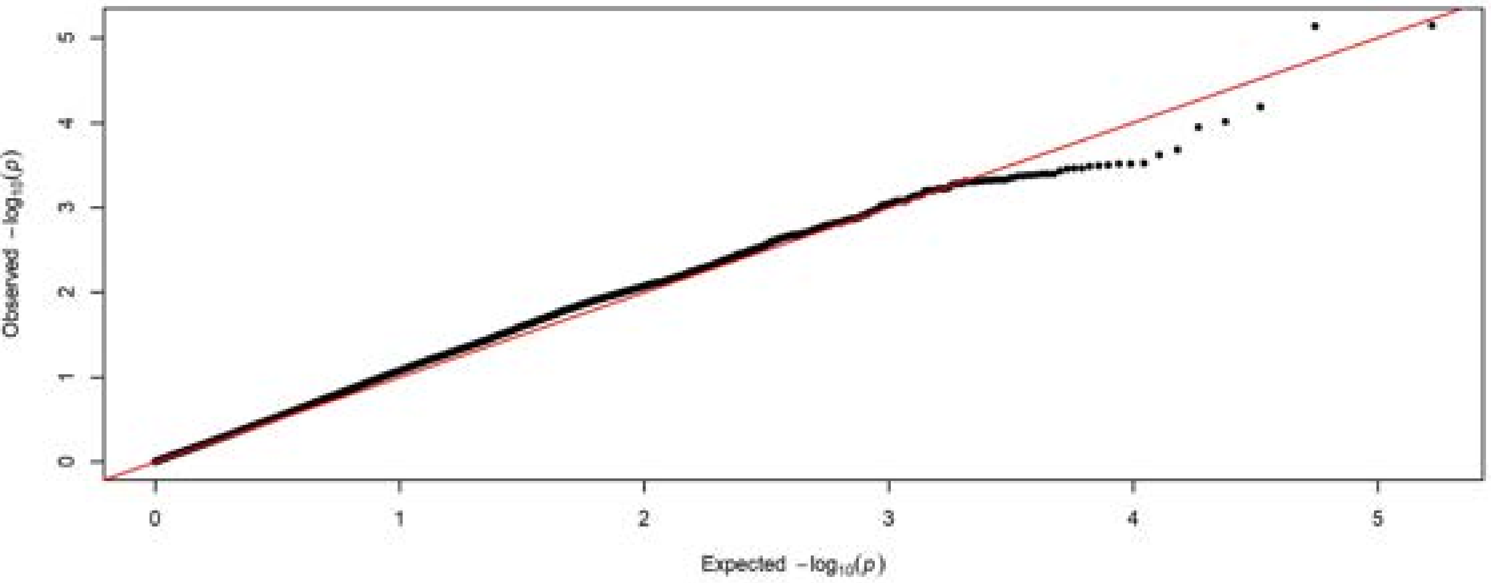

Supplement: S5 File — Each page contains Q-Q plots for the meta-analyses of the GWAS results for a given day/metric across (1) all seven subgroups, (2) Charles River subgroups, and (3) Harlan subgroups. (PDF) [file pgen.1010234.s023.pdf]
